# Pain Phenotypes and Pain Multimorbidity Among Medicare Beneficiaries With Cerebral Palsy

**DOI:** 10.1001/jamaneurol.2024.2443

**Published:** 2024-08-05

**Authors:** Mark D. Peterson, Kathryn Ashbaugh, Michael O’Leary, Mary Schmidt, Heidi Haapala, Neil Kamdar, Edward A. Hurvitz

**Affiliations:** 1 Department of Physical Medicine and Rehabilitation, Michigan Medicine, University of Michigan, Ann Arbor; 2 Institute for Healthcare Policy and Innovation, Michigan Medicine, University of Michigan, Ann Arbor; 3 Center for Population Health Sciences, Stanford University, California; 4 Cecil H. Sheps Center for Health Services Research, University of North Carolina at Chapel Hill

## Abstract

This cohort study assesses the association of pain phenotypes and pain multimorbidity with cerebral palsy subtypes among Medicare beneficiaries.

Pain is the most prevalent somatic symptom in cerebral palsy (CP) and has been reported in 70% to 75% of adults (aged ≥18 years) with CP.^1^ However, pain is perhaps the least understood and studied comorbidity of CP.^2,3,4^ Understanding phenotypes of pain among adults with CP is crucial for prescribing the most appropriate and effective pain management interventions that match treatments to underlying pain mechanisms. The objective of this study was to compare the prevalence of nociplastic, neuropathic, nociceptive, and mixed pain subtypes in adults with CP; describe combinations of pain subtypes; and assess their association with CP subtypes.

## Methods

This cohort study used Medicare fee-for-service research identifiable files from the 20% random sample from January 1, 2008, through December 31, 2020, to identify a cohort of patients with CP. We used data from the Master Beneficiary Summary File, Medicare Provider Analysis and Review, outpatient file, and carrier file to identify these patients. The University of Michigan institutional review board deemed this study exempt and waived the need for informed consent as data were retrospective and deidentified. The study followed the STROBE reporting guideline.

Patients with CP aged 18 years or older at enrollment were identified based on *ICD-9* and *ICD-10* codes. We excluded patients enrolled in Medicare Advantage or Part C or whose coverage lapsed from January 1, 2016, through December 31, 2020. We identified pain diagnoses using *ICD-10* codes^5^ (eTable in Supplement 1).

Counts and proportions of demographic information, including age, sex, race and ethnicity, census region, dual enrollment status, and CP subtype, were calculated for the entire cohort, along with prevalence of pain. Race and ethnicity data were from beneficiary claims and included to show that the sample is representative of the US population. All analyses were conducted between October 3, 2023, and April 24, 2024, using SAS, version 9.4 (SAS Institute Inc).

## Results

We identified 24 464 eligible adults with CP (mean [SD], age 48.9 [13.8] years, 46.7% female, 53.3% male) (Table 1). A total of 21 767 patients (89.0%) had 1 or more documented pain diagnoses, 2697 (11.0%) had no pain diagnosis, 3708 (15.2%) had a single pain condition throughout the study period, 18 059 (73.8%) exhibited pain multimorbidity (ie, ≥2 diagnoses), and 7259 (29.7%) had pain extreme multimorbidity (ie, ≥5 diagnoses). All nonmutually exclusive pain conditions are listed in Table 2.

**Table 1.** Descriptive Characteristics of Adults With Cerebral Palsy

Characteristic	No. (%)
No. of patients	24 464
Age group, y	
15-35	4902 (20.0)
36-55	11 190 (45.7)
56-75	7859 (32.1)
76-104	513 (2.1)
Sex	
Female	11 418 (46.7)
Male	13 046 (53.3)
Race and ethnicity	
American Indian or Alaska Native	221 (0.9)
Asian or Pacific Islander	345 (1.4)
Hispanic	1807 (7.4)
Non-Hispanic Black	3329 (13.6)
Non-Hispanic White	18 449 (75.4)
Unknown or other	313 (1.3)
Census region	
Midwest	6695 (27.4)
Northeast	5280 (21.6)
South	8247 (33.7)
West	4242 (17.3)
Dual enrollment status	
None	1550 (6.3)
Part	3785 (15.5)
Full	19 129 (78.2)
Cerebral palsy subtype	
Ataxic	627 (2.6)
Athetoid	784 (3.2)
Diplegia	2764 (11.3)
Hemiplegia	1632 (6.6)
Mixed	1286 (5.3)
Other or unspecified	12 459 (50.9)
Quadriplegia	4912 (20.1)

**Table 2.** Prevalence of Pain Diagnoses Among Adults With Cerebral Palsy (N = 24 464)

Pain category	No. of patients (%)
Arthritis joint other	17 501 (71.5)
Arthritis joint lower limb	13 578 (55.5)
Arthritis joint upper limb	8300 (33.9)
Back pain low back	6827 (27.9)
Back pain other or unspecified	6373 (26.1)
Arthritis joint spine and hips	6254 (25.6)
Headache	5240 (21.4)
Neck pain	4853 (19.8)
Noncardiac or musculoskeletal chest pain	3840 (15.7)
Neuralgia, neuritis, and radiculitis, unspecified	3596 (14.7)
Back pain mid back	2806 (11.5)
None	2697 (11.0)
Myalgia and myositis, unspecified (fibromyalgia)	1566 (6.4)
Functional bowel irritable bowel syndrome	1223 (5.0)
Type 2 diabetes with diabetic neuropathy	1060 (4.3)
Chronic fatigue	897 (3.7)
Migraine	782 (3.2)
Dyspepsia	674 (2.8)
Dyskinesia of esophagus	372 (1.5)
Occipital neuralgia	140 (0.6)
Endometriosis	117 (0.5)
Interstitial cystitis	99 (0.4)
Phantom limb syndrome with pain	32 (0.1)
Vulvodynia	21 (0.1)
Erythromelalgia	<11 (<0.1)^a^

^a^ Per Centers for Medicare & Medicaid Services reporting rules, no specific data can be provided for any cell with fewer than 11 individuals.

The distribution of patients by pain phenotype was 21 101 (86.3%) with any evidence of nociceptive pain, 11 213 (45.8%) with any evidence of nociplastic pain, and 4139 (16.9%) with any evidence of neuropathic pain. The distribution of patients across the cohort was 9499 (38.8%) with nociceptive pain only; 7538 (30.8%) with nociceptive and nociplastic pain; 3065 (12.5%) with neuropathic, nociceptive, and nociplastic pain; 999 (4.1%) with neuropathic and nociceptive pain; 591 (2.4%) with nociplastic pain only; 56 (0.2%) with neuropathic pain only; and 19 (0.1%) with neuropathic and nociplastic pain.

## Discussion

This cohort study is, to our knowledge, the largest to examine pain and its subtypes by topologic phenotype. Our finding of 89.0% pain prevalence underscores the importance of understanding and treating pain in adults with CP. Furthermore, 73.8% of the patients had more than 1 pain diagnosis, which increases the complexity of treatment decisions and highlights the necessity of understanding etiology to deliver effective treatment. Pain subtypes and combinations of subtypes were similar across all clinical presentations of CP. A limitation of this study is that sensitivity and specificity of using *ICD* codes for the identification of CP and CP subtypes are unknown. These findings provide crucial information for clinical and public health audiences, especially given the comparison to the general population, where chronic pain is estimated to be present in approximately 20% of adults.^6^

## Notes

Supplement 1. **eTable.** *International Classification of Diseases, Tenth Revision, Clinical Modification* (*ICD-10-CM*) for Identification of Pain Conditions in the Claim

Supplement 2.
Data Sharing Statement
