# Supplementary material for: Pain Phenotypes and Pain Multimorbidity Among Medicare Beneficiaries With Cerebral Palsy
Source: JAMA Neurol. 2024 Aug 5;81(9):1004–5. doi: 10.1001/jamaneurol.2024.2443 (PMC11385049; doi:10.1001/jamaneurol.2024.2443)
Supplement: Supplement 1. — eTable. International Classification of Diseases, Tenth Revision, Clinical Modification (ICD-10-CM) for Identification of Pain Conditions in the Claim [file jamaneurol-e242443-s001.pdf]

Supplemental Online Content

Peterson MD, Ashbaugh K, O'Leary M, et al. Pain phenotypes and pain multimorbidity among Medicare beneficiaries with cerebral palsy. *JAMA Neurol*. Published online August 5, 2024. doi:10.1001/jamaneurol.2024.2443

eTable. *International Classification of Diseases, Tenth Revision, Clinical Modification* (*ICD-10-CM*) for Identification of Pain Conditions in the Claim

This supplemental material has been provided by the authors to give readers additional information about their work.

© 2024 Peterson MD et al. *JAMA Neurology.*

eTable. *International Classification of Diseases, Tenth Revision, Clinical Modification* (*ICD-10-CM*) for Identification of Pain Conditions in the Claim

DX	Condition	Category	Pain Type
B022	Zoster with other nervous system involvement	Neuralgia, neuritis, and radiculitis, unspecified	Neuropathic Pain
E1140	Type 2 diabetes mellitus with diabetic neuropathy	Type 2 diabetes mellitus with diabetic neuropathy	Neuropathic Pain
G50	Disorders of trigeminal nerve	Neuralgia, neuritis, and radiculitis, unspecified	Neuropathic Pain
G546	Phantom limb syndrome with pain	Phantom limb syndrome with pain	Neuropathic Pain
G629	Polyneuropathy, unspecified	Neuralgia, neuritis, and radiculitis, unspecified	Neuropathic Pain
G63	Polyneuropathy in diseases classified elsewhere	Neuralgia, neuritis, and radiculitis, unspecified	Neuropathic Pain
G9009	Other idiopathic peripheral autonomic neuropathy	Neuralgia, neuritis, and radiculitis, unspecified	Neuropathic Pain
I7381	Erythromelalgia	Erythromelalgia	Neuropathic Pain
M5410	Radiculopathy, site unspecified	Neuralgia, neuritis, and radiculitis, unspecified	Neuropathic Pain
M5411	Radiculopathy, occipito-atlanto-axial region	Neuralgia, neuritis, and radiculitis, unspecified	Neuropathic Pain
M5412	Radiculopathy, cervical region	Neuralgia, neuritis, and radiculitis, unspecified	Neuropathic Pain
M5413	Radiculopathy, cervicothoracic region	Neuralgia, neuritis, and radiculitis, unspecified	Neuropathic Pain
M5414	Radiculopathy, thoracic region	Neuralgia, neuritis, and radiculitis, unspecified	Neuropathic Pain
M5415	Radiculopathy, thoracolumbar region	Neuralgia, neuritis, and radiculitis, unspecified	Neuropathic Pain
M5416	Radiculopathy, lumbar region	Neuralgia, neuritis, and radiculitis, unspecified	Neuropathic Pain
M5417	Radiculopathy, lumbosacral region	Neuralgia, neuritis, and radiculitis, unspecified	Neuropathic Pain
M5418	Radiculopathy, sacral and sacrococcygeal region	Neuralgia, neuritis, and radiculitis, unspecified	Neuropathic Pain
M792	Neuralgia and neuritis, unspecified	Neuralgia, neuritis, and radiculitis, unspecified	Neuropathic Pain
M00	Pyogenic arthritis	Arthritis Joint Other	Nociceptive Pain
M000	Staphylococcal arthritis and polyarthritis	Arthritis Joint Other	Nociceptive Pain
M0000	Staphylococcal arthritis, unspecified joint	Arthritis Joint Other	Nociceptive Pain
M0001	Staphylococcal arthritis, shoulder	Arthritis Joint Upper Limb	Nociceptive Pain
M00011	Staphylococcal arthritis, right shoulder	Arthritis Joint Upper Limb	Nociceptive Pain
M00012	Staphylococcal arthritis, left shoulder	Arthritis Joint Upper Limb	Nociceptive Pain
M00019	Staphylococcal arthritis, unspecified shoulder	Arthritis Joint Upper Limb	Nociceptive Pain
M0002	Staphylococcal arthritis, elbow	Arthritis Joint Upper Limb	Nociceptive Pain
M00021	Staphylococcal arthritis, right elbow	Arthritis Joint Upper Limb	Nociceptive Pain
M00022	Staphylococcal arthritis, left elbow	Arthritis Joint Upper Limb	Nociceptive Pain
M00029	Staphylococcal arthritis, unspecified elbow	Arthritis Joint Upper Limb	Nociceptive Pain
M0003	Staphylococcal arthritis, wrist	Arthritis Joint Upper Limb	Nociceptive Pain
M00031	Staphylococcal arthritis, right wrist	Arthritis Joint Upper Limb	Nociceptive Pain
M00032	Staphylococcal arthritis, left wrist	Arthritis Joint Upper Limb	Nociceptive Pain
M00039	Staphylococcal arthritis, unspecified wrist	Arthritis Joint Upper Limb	Nociceptive Pain
M0004	Staphylococcal arthritis, hand	Arthritis Joint Upper Limb	Nociceptive Pain
M00041	Staphylococcal arthritis, right hand	Arthritis Joint Upper Limb	Nociceptive Pain

© 2024 Peterson MD et al. *JAMA Neurology.*

M00042	Staphylococcal arthritis, left hand	Arthritis Joint Upper Limb	Nociceptive Pain
M00049	Staphylococcal arthritis, unspecified hand	Arthritis Joint Upper Limb	Nociceptive Pain
M0005	Staphylococcal arthritis, hip	Arthritis Joint Spine and Hips	Nociceptive Pain
M00051	Staphylococcal arthritis, right hip	Arthritis Joint Spine and Hips	Nociceptive Pain
M00052	Staphylococcal arthritis, left hip	Arthritis Joint Spine and Hips	Nociceptive Pain
M00059	Staphylococcal arthritis, unspecified hip	Arthritis Joint Spine and Hips	Nociceptive Pain
M0006	Staphylococcal arthritis, knee	Arthritis Joint Lower Limb	Nociceptive Pain
M00061	Staphylococcal arthritis, right knee	Arthritis Joint Lower Limb	Nociceptive Pain
M00062	Staphylococcal arthritis, left knee	Arthritis Joint Lower Limb	Nociceptive Pain
M00069	Staphylococcal arthritis, unspecified knee	Arthritis Joint Lower Limb	Nociceptive Pain
M0007	Staphylococcal arthritis, ankle and foot	Arthritis Joint Lower Limb	Nociceptive Pain
M00071	Staphylococcal arthritis, right ankle and foot	Arthritis Joint Lower Limb	Nociceptive Pain
M00072	Staphylococcal arthritis, left ankle and foot	Arthritis Joint Lower Limb	Nociceptive Pain
M00079	Staphylococcal arthritis, unspecified ankle and foot	Arthritis Joint Lower Limb	Nociceptive Pain
M0008	Staphylococcal arthritis, vertebrae	Arthritis Joint Spine and Hips	Nociceptive Pain
M0009	Staphylococcal polyarthritis	Arthritis Joint Other	Nociceptive Pain
M001	Pneumococcal arthritis and polyarthritis	Arthritis Joint Other	Nociceptive Pain
M0010	Pneumococcal arthritis, unspecified joint	Arthritis Joint Other	Nociceptive Pain
M0011	Pneumococcal arthritis, shoulder	Arthritis Joint Upper Limb	Nociceptive Pain
M00111	Pneumococcal arthritis, right shoulder	Arthritis Joint Upper Limb	Nociceptive Pain
M00112	Pneumococcal arthritis, left shoulder	Arthritis Joint Upper Limb	Nociceptive Pain
M00119	Pneumococcal arthritis, unspecified shoulder	Arthritis Joint Upper Limb	Nociceptive Pain
M0012	Pneumococcal arthritis, elbow	Arthritis Joint Upper Limb	Nociceptive Pain
M00121	Pneumococcal arthritis, right elbow	Arthritis Joint Upper Limb	Nociceptive Pain
M00122	Pneumococcal arthritis, left elbow	Arthritis Joint Upper Limb	Nociceptive Pain
M00129	Pneumococcal arthritis, unspecified elbow	Arthritis Joint Upper Limb	Nociceptive Pain
M0013	Pneumococcal arthritis, wrist	Arthritis Joint Upper Limb	Nociceptive Pain
M00131	Pneumococcal arthritis, right wrist	Arthritis Joint Upper Limb	Nociceptive Pain
M00132	Pneumococcal arthritis, left wrist	Arthritis Joint Upper Limb	Nociceptive Pain
M00139	Pneumococcal arthritis, unspecified wrist	Arthritis Joint Upper Limb	Nociceptive Pain
M0014	Pneumococcal arthritis, hand	Arthritis Joint Upper Limb	Nociceptive Pain
M00141	Pneumococcal arthritis, right hand	Arthritis Joint Upper Limb	Nociceptive Pain
M00142	Pneumococcal arthritis, left hand	Arthritis Joint Upper Limb	Nociceptive Pain
M00149	Pneumococcal arthritis, unspecified hand	Arthritis Joint Upper Limb	Nociceptive Pain
M0015	Pneumococcal arthritis, hip	Arthritis Joint Spine and Hips	Nociceptive Pain
M00151	Pneumococcal arthritis, right hip	Arthritis Joint Spine and Hips	Nociceptive Pain
M00152	Pneumococcal arthritis, left hip	Arthritis Joint Spine and Hips	Nociceptive Pain

© 2024 Peterson MD et al. *JAMA Neurology.*

M00159	Pneumococcal arthritis, unspecified hip	Arthritis Joint Spine and Hips	Nociceptive Pain
M0016	Pneumococcal arthritis, knee	Arthritis Joint Lower Limb	Nociceptive Pain
M00161	Pneumococcal arthritis, right knee	Arthritis Joint Lower Limb	Nociceptive Pain
M00162	Pneumococcal arthritis, left knee	Arthritis Joint Lower Limb	Nociceptive Pain
M00169	Pneumococcal arthritis, unspecified knee	Arthritis Joint Lower Limb	Nociceptive Pain
M0017	Pneumococcal arthritis, ankle and foot	Arthritis Joint Lower Limb	Nociceptive Pain
M00171	Pneumococcal arthritis, right ankle and foot	Arthritis Joint Lower Limb	Nociceptive Pain
M00172	Pneumococcal arthritis, left ankle and foot	Arthritis Joint Lower Limb	Nociceptive Pain
M00179	Pneumococcal arthritis, unspecified ankle and foot	Arthritis Joint Lower Limb	Nociceptive Pain
M0018	Pneumococcal arthritis, vertebrae	Arthritis Joint Spine and Hips	Nociceptive Pain
M0019	Pneumococcal polyarthritis	Arthritis Joint Other	Nociceptive Pain
M002	Other streptococcal arthritis and polyarthritis	Arthritis Joint Other	Nociceptive Pain
M0020	Other streptococcal arthritis, unspecified joint	Arthritis Joint Other	Nociceptive Pain
M0021	Other streptococcal arthritis, shoulder	Arthritis Joint Upper Limb	Nociceptive Pain
M00211	Other streptococcal arthritis, right shoulder	Arthritis Joint Upper Limb	Nociceptive Pain
M00212	Other streptococcal arthritis, left shoulder	Arthritis Joint Upper Limb	Nociceptive Pain
M00219	Other streptococcal arthritis, unspecified shoulder	Arthritis Joint Upper Limb	Nociceptive Pain
M0022	Other streptococcal arthritis, elbow	Arthritis Joint Upper Limb	Nociceptive Pain
M00221	Other streptococcal arthritis, right elbow	Arthritis Joint Upper Limb	Nociceptive Pain
M00222	Other streptococcal arthritis, left elbow	Arthritis Joint Upper Limb	Nociceptive Pain
M00229	Other streptococcal arthritis, unspecified elbow	Arthritis Joint Upper Limb	Nociceptive Pain
M0023	Other streptococcal arthritis, wrist	Arthritis Joint Upper Limb	Nociceptive Pain
M00231	Other streptococcal arthritis, right wrist	Arthritis Joint Upper Limb	Nociceptive Pain
M00232	Other streptococcal arthritis, left wrist	Arthritis Joint Upper Limb	Nociceptive Pain
M00239	Other streptococcal arthritis, unspecified wrist	Arthritis Joint Upper Limb	Nociceptive Pain
M0024	Other streptococcal arthritis, hand	Arthritis Joint Upper Limb	Nociceptive Pain
M00241	Other streptococcal arthritis, right hand	Arthritis Joint Upper Limb	Nociceptive Pain
M00242	Other streptococcal arthritis, left hand	Arthritis Joint Upper Limb	Nociceptive Pain
M00249	Other streptococcal arthritis, unspecified hand	Arthritis Joint Upper Limb	Nociceptive Pain
M0025	Other streptococcal arthritis, hip	Arthritis Joint Spine and Hips	Nociceptive Pain
M00251	Other streptococcal arthritis, right hip	Arthritis Joint Spine and Hips	Nociceptive Pain
M00252	Other streptococcal arthritis, left hip	Arthritis Joint Spine and Hips	Nociceptive Pain
M00259	Other streptococcal arthritis, unspecified hip	Arthritis Joint Spine and Hips	Nociceptive Pain
M0026	Other streptococcal arthritis, knee	Arthritis Joint Lower Limb	Nociceptive Pain
M00261	Other streptococcal arthritis, right knee	Arthritis Joint Lower Limb	Nociceptive Pain
M00262	Other streptococcal arthritis, left knee	Arthritis Joint Lower Limb	Nociceptive Pain
M00269	Other streptococcal arthritis, unspecified knee	Arthritis Joint Lower Limb	Nociceptive Pain

© 2024 Peterson MD et al. *JAMA Neurology.*

M0027	Other streptococcal arthritis, ankle and foot	Arthritis Joint Lower Limb	Nociceptive Pain
M00271	Other streptococcal arthritis, right ankle and foot	Arthritis Joint Lower Limb	Nociceptive Pain
M00272	Other streptococcal arthritis, left ankle and foot	Arthritis Joint Lower Limb	Nociceptive Pain
M00279	Other streptococcal arthritis, unspecified ankle and foot	Arthritis Joint Lower Limb	Nociceptive Pain
M0028	Other streptococcal arthritis, vertebrae	Arthritis Joint Spine and Hips	Nociceptive Pain
M0029	Other streptococcal polyarthritis	Arthritis Joint Other	Nociceptive Pain
M008	Arthritis and polyarthritis due to other bacteria	Arthritis Joint Other	Nociceptive Pain
M0080	Arthritis due to other bacteria, unspecified joint	Arthritis Joint Other	Nociceptive Pain
M0081	Arthritis due to other bacteria, shoulder	Arthritis Joint Upper Limb	Nociceptive Pain
M00811	Arthritis due to other bacteria, right shoulder	Arthritis Joint Upper Limb	Nociceptive Pain
M00812	Arthritis due to other bacteria, left shoulder	Arthritis Joint Upper Limb	Nociceptive Pain
M00819	Arthritis due to other bacteria, unspecified shoulder	Arthritis Joint Upper Limb	Nociceptive Pain
M0082	Arthritis due to other bacteria, elbow	Arthritis Joint Upper Limb	Nociceptive Pain
M00821	Arthritis due to other bacteria, right elbow	Arthritis Joint Upper Limb	Nociceptive Pain
M00822	Arthritis due to other bacteria, left elbow	Arthritis Joint Upper Limb	Nociceptive Pain
M00829	Arthritis due to other bacteria, unspecified elbow	Arthritis Joint Upper Limb	Nociceptive Pain
M0083	Arthritis due to other bacteria, wrist	Arthritis Joint Upper Limb	Nociceptive Pain
M00831	Arthritis due to other bacteria, right wrist	Arthritis Joint Upper Limb	Nociceptive Pain
M00832	Arthritis due to other bacteria, left wrist	Arthritis Joint Upper Limb	Nociceptive Pain
M00839	Arthritis due to other bacteria, unspecified wrist	Arthritis Joint Upper Limb	Nociceptive Pain
M0084	Arthritis due to other bacteria, hand	Arthritis Joint Upper Limb	Nociceptive Pain
M00841	Arthritis due to other bacteria, right hand	Arthritis Joint Upper Limb	Nociceptive Pain
M00842	Arthritis due to other bacteria, left hand	Arthritis Joint Upper Limb	Nociceptive Pain
M00849	Arthritis due to other bacteria, unspecified hand	Arthritis Joint Upper Limb	Nociceptive Pain
M0085	Arthritis due to other bacteria, hip	Arthritis Joint Spine and Hips	Nociceptive Pain
M00851	Arthritis due to other bacteria, right hip	Arthritis Joint Spine and Hips	Nociceptive Pain
M00852	Arthritis due to other bacteria, left hip	Arthritis Joint Spine and Hips	Nociceptive Pain
M00859	Arthritis due to other bacteria, unspecified hip	Arthritis Joint Spine and Hips	Nociceptive Pain
M0086	Arthritis due to other bacteria, knee	Arthritis Joint Lower Limb	Nociceptive Pain
M00861	Arthritis due to other bacteria, right knee	Arthritis Joint Lower Limb	Nociceptive Pain
M00862	Arthritis due to other bacteria, left knee	Arthritis Joint Lower Limb	Nociceptive Pain
M00869	Arthritis due to other bacteria, unspecified knee	Arthritis Joint Lower Limb	Nociceptive Pain
M0087	Arthritis due to other bacteria, ankle and foot	Arthritis Joint Lower Limb	Nociceptive Pain
M00871	Arthritis due to other bacteria, right ankle and foot	Arthritis Joint Lower Limb	Nociceptive Pain
M00872	Arthritis due to other bacteria, left ankle and foot	Arthritis Joint Lower Limb	Nociceptive Pain
M00879	Arthritis due to other bacteria, unspecified ankle and foot	Arthritis Joint Lower Limb	Nociceptive Pain
M0088	Arthritis due to other bacteria, vertebrae	Arthritis Joint Spine and Hips	Nociceptive Pain

© 2024 Peterson MD et al. *JAMA Neurology.*

M0089	Polyarthritis due to other bacteria	Arthritis Joint Other	Nociceptive Pain
M009	Pyogenic arthritis, unspecified	Arthritis Joint Other	Nociceptive Pain
M01	Direct infect of joint in infec/parastc dis classd elswhr	Arthritis Joint Other	Nociceptive Pain
M01X	Direct infct of joint in infec/parastc dis classd elswhr	Arthritis Joint Other	Nociceptive Pain
M01X0	Dir infct of unsp joint in infec/parastc dis classd elswhr	Arthritis Joint Other	Nociceptive Pain
M01X1	Direct infct of shldr jt in infec/parastc dis classd elswhr	Arthritis Joint Upper Limb	Nociceptive Pain
M01X11	Direct infct of r shldr in infec/parastc dis classd elswhr	Arthritis Joint Upper Limb	Nociceptive Pain
M01X12	Direct infct of l shldr in infec/parastc dis classd elswhr	Arthritis Joint Upper Limb	Nociceptive Pain
M01X19	Dir infct of unsp shldr in infec/parastc dis classd elswhr	Arthritis Joint Upper Limb	Nociceptive Pain
M01X2	Direct infct of elbow in infec/parastc dis classd elswhr	Arthritis Joint Upper Limb	Nociceptive Pain
M01X21	Direct infct of r elbow in infec/parastc dis classd elswhr	Arthritis Joint Upper Limb	Nociceptive Pain
M01X22	Direct infct of l elbow in infec/parastc dis classd elswhr	Arthritis Joint Upper Limb	Nociceptive Pain
M01X29	Dir infct of unsp elbow in infec/parastc dis classd elswhr	Arthritis Joint Upper Limb	Nociceptive Pain
M01X3	Direct infct of wrist in infec/parastc dis classd elswhr	Arthritis Joint Upper Limb	Nociceptive Pain
M01X31	Direct infct of r wrist in infec/parastc dis classd elswhr	Arthritis Joint Upper Limb	Nociceptive Pain
M01X32	Direct infct of l wrist in infec/parastc dis classd elswhr	Arthritis Joint Upper Limb	Nociceptive Pain
M01X39	Dir infct of unsp wrist in infec/parastc dis classd elswhr	Arthritis Joint Upper Limb	Nociceptive Pain
M01X4	Direct infct of hand in infec/parastc diseases classd elswhr	Arthritis Joint Upper Limb	Nociceptive Pain
M01X41	Direct infct of r hand in infec/parastc dis classd elswhr	Arthritis Joint Upper Limb	Nociceptive Pain
M01X42	Direct infct of l hand in infec/parastc dis classd elswhr	Arthritis Joint Upper Limb	Nociceptive Pain
M01X49	Direct infct of unsp hand in infec/parastc dis classd elswhr	Arthritis Joint Upper Limb	Nociceptive Pain
M01X5	Direct infct of hip in infec/parastc diseases classd elswhr	Arthritis Joint Spine and Hips	Nociceptive Pain
M01X51	Direct infct of r hip in infec/parastc dis classd elswhr	Arthritis Joint Spine and Hips	Nociceptive Pain
M01X52	Direct infct of left hip in infec/parastc dis classd elswhr	Arthritis Joint Spine and Hips	Nociceptive Pain
M01X59	Direct infct of unsp hip in infec/parastc dis classd elswhr	Arthritis Joint Spine and Hips	Nociceptive Pain
M01X6	Direct infct of knee in infec/parastc diseases classd elswhr	Arthritis Joint Lower Limb	Nociceptive Pain
M01X61	Direct infct of r knee in infec/parastc dis classd elswhr	Arthritis Joint Lower Limb	Nociceptive Pain
M01X62	Direct infct of l knee in infec/parastc dis classd elswhr	Arthritis Joint Lower Limb	Nociceptive Pain
M01X69	Direct infct of unsp knee in infec/parastc dis classd elswhr	Arthritis Joint Lower Limb	Nociceptive Pain
M01X7	Direct infct of ank/ft in infec/parastc dis classd elswhr	Arthritis Joint Lower Limb	Nociceptive Pain
M01X71	Dir infct of right ank/ft in infec/parastc dis classd elswhr	Arthritis Joint Lower Limb	Nociceptive Pain
M01X72	Dir infct of left ank/ft in infec/parastc dis classd elswhr	Arthritis Joint Lower Limb	Nociceptive Pain
M01X79	Dir infct of unsp ank/ft in infec/parastc dis classd elswhr	Arthritis Joint Lower Limb	Nociceptive Pain
M01X8	Direct infct of verteb in infec/parastc dis classd elswhr	Arthritis Joint Other	Nociceptive Pain
M01X9	Dir infct of mult joints in infec/parastc dis classd elswhr	Arthritis Joint Other	Nociceptive Pain
M02	Postinfective and reactive arthropathies	Arthritis Joint Other	Nociceptive Pain
M020	Arthropathy following intestinal bypass	Arthritis Joint Other	Nociceptive Pain

© 2024 Peterson MD et al. *JAMA Neurology.*

M0200	Arthropathy following intestinal bypass, unspecified site	Arthritis Joint Other	Nociceptive Pain
M0201	Arthropathy following intestinal bypass, shoulder	Arthritis Joint Upper Limb	Nociceptive Pain
M02011	Arthropathy following intestinal bypass, right shoulder	Arthritis Joint Upper Limb	Nociceptive Pain
M02012	Arthropathy following intestinal bypass, left shoulder	Arthritis Joint Upper Limb	Nociceptive Pain
M02019	Arthropathy following intestinal bypass, unsp shoulder	Arthritis Joint Upper Limb	Nociceptive Pain
M0202	Arthropathy following intestinal bypass, elbow	Arthritis Joint Upper Limb	Nociceptive Pain
M02021	Arthropathy following intestinal bypass, right elbow	Arthritis Joint Upper Limb	Nociceptive Pain
M02022	Arthropathy following intestinal bypass, left elbow	Arthritis Joint Upper Limb	Nociceptive Pain
M02029	Arthropathy following intestinal bypass, unspecified elbow	Arthritis Joint Upper Limb	Nociceptive Pain
M0203	Arthropathy following intestinal bypass, wrist	Arthritis Joint Upper Limb	Nociceptive Pain
M02031	Arthropathy following intestinal bypass, right wrist	Arthritis Joint Upper Limb	Nociceptive Pain
M02032	Arthropathy following intestinal bypass, left wrist	Arthritis Joint Upper Limb	Nociceptive Pain
M02039	Arthropathy following intestinal bypass, unspecified wrist	Arthritis Joint Upper Limb	Nociceptive Pain
M0204	Arthropathy following intestinal bypass, hand	Arthritis Joint Upper Limb	Nociceptive Pain
M02041	Arthropathy following intestinal bypass, right hand	Arthritis Joint Upper Limb	Nociceptive Pain
M02042	Arthropathy following intestinal bypass, left hand	Arthritis Joint Upper Limb	Nociceptive Pain
M02049	Arthropathy following intestinal bypass, unspecified hand	Arthritis Joint Upper Limb	Nociceptive Pain
M0205	Arthropathy following intestinal bypass, hip	Arthritis Joint Spine and Hips	Nociceptive Pain
M02051	Arthropathy following intestinal bypass, right hip	Arthritis Joint Spine and Hips	Nociceptive Pain
M02052	Arthropathy following intestinal bypass, left hip	Arthritis Joint Spine and Hips	Nociceptive Pain
M02059	Arthropathy following intestinal bypass, unspecified hip	Arthritis Joint Spine and Hips	Nociceptive Pain
M0206	Arthropathy following intestinal bypass, knee	Arthritis Joint Lower Limb	Nociceptive Pain
M02061	Arthropathy following intestinal bypass, right knee	Arthritis Joint Lower Limb	Nociceptive Pain
M02062	Arthropathy following intestinal bypass, left knee	Arthritis Joint Lower Limb	Nociceptive Pain
M02069	Arthropathy following intestinal bypass, unspecified knee	Arthritis Joint Lower Limb	Nociceptive Pain
M0207	Arthropathy following intestinal bypass, ankle and foot	Arthritis Joint Lower Limb	Nociceptive Pain
M02071	Arthropathy following intestinal bypass, right ank/ft	Arthritis Joint Lower Limb	Nociceptive Pain
M02072	Arthropathy following intestinal bypass, left ankle and foot	Arthritis Joint Lower Limb	Nociceptive Pain
M02079	Arthropathy following intestinal bypass, unsp ankle and foot	Arthritis Joint Lower Limb	Nociceptive Pain
M0208	Arthropathy following intestinal bypass, vertebrae	Arthritis Joint Spine and Hips	Nociceptive Pain
M0209	Arthropathy following intestinal bypass, multiple sites	Arthritis Joint Other	Nociceptive Pain
M021	Postdysenteric arthropathy	Arthritis Joint Other	Nociceptive Pain
M0210	Postdysenteric arthropathy, unspecified site	Arthritis Joint Other	Nociceptive Pain
M0211	Postdysenteric arthropathy, shoulder	Arthritis Joint Upper Limb	Nociceptive Pain
M02111	Postdysenteric arthropathy, right shoulder	Arthritis Joint Upper Limb	Nociceptive Pain
M02112	Postdysenteric arthropathy, left shoulder	Arthritis Joint Upper Limb	Nociceptive Pain
M02119	Postdysenteric arthropathy, unspecified shoulder	Arthritis Joint Upper Limb	Nociceptive Pain

© 2024 Peterson MD et al. *JAMA Neurology.*

M0212	Postdysenteric arthropathy, elbow	Arthritis Joint Upper Limb	Nociceptive Pain
M02121	Postdysenteric arthropathy, right elbow	Arthritis Joint Upper Limb	Nociceptive Pain
M02122	Postdysenteric arthropathy, left elbow	Arthritis Joint Upper Limb	Nociceptive Pain
M02129	Postdysenteric arthropathy, unspecified elbow	Arthritis Joint Upper Limb	Nociceptive Pain
M0213	Postdysenteric arthropathy, wrist	Arthritis Joint Upper Limb	Nociceptive Pain
M02131	Postdysenteric arthropathy, right wrist	Arthritis Joint Upper Limb	Nociceptive Pain
M02132	Postdysenteric arthropathy, left wrist	Arthritis Joint Upper Limb	Nociceptive Pain
M02139	Postdysenteric arthropathy, unspecified wrist	Arthritis Joint Upper Limb	Nociceptive Pain
M0214	Postdysenteric arthropathy, hand	Arthritis Joint Upper Limb	Nociceptive Pain
M02141	Postdysenteric arthropathy, right hand	Arthritis Joint Upper Limb	Nociceptive Pain
M02142	Postdysenteric arthropathy, left hand	Arthritis Joint Upper Limb	Nociceptive Pain
M02149	Postdysenteric arthropathy, unspecified hand	Arthritis Joint Upper Limb	Nociceptive Pain
M0215	Postdysenteric arthropathy, hip	Arthritis Joint Spine and Hips	Nociceptive Pain
M02151	Postdysenteric arthropathy, right hip	Arthritis Joint Spine and Hips	Nociceptive Pain
M02152	Postdysenteric arthropathy, left hip	Arthritis Joint Spine and Hips	Nociceptive Pain
M02159	Postdysenteric arthropathy, unspecified hip	Arthritis Joint Spine and Hips	Nociceptive Pain
M0216	Postdysenteric arthropathy, knee	Arthritis Joint Lower Limb	Nociceptive Pain
M02161	Postdysenteric arthropathy, right knee	Arthritis Joint Lower Limb	Nociceptive Pain
M02162	Postdysenteric arthropathy, left knee	Arthritis Joint Lower Limb	Nociceptive Pain
M02169	Postdysenteric arthropathy, unspecified knee	Arthritis Joint Lower Limb	Nociceptive Pain
M0217	Postdysenteric arthropathy, ankle and foot	Arthritis Joint Lower Limb	Nociceptive Pain
M02171	Postdysenteric arthropathy, right ankle and foot	Arthritis Joint Lower Limb	Nociceptive Pain
M02172	Postdysenteric arthropathy, left ankle and foot	Arthritis Joint Lower Limb	Nociceptive Pain
M02179	Postdysenteric arthropathy, unspecified ankle and foot	Arthritis Joint Lower Limb	Nociceptive Pain
M0218	Postdysenteric arthropathy, vertebrae	Arthritis Joint Spine and Hips	Nociceptive Pain
M0219	Postdysenteric arthropathy, multiple sites	Arthritis Joint Other	Nociceptive Pain
M022	Postimmunization arthropathy	Arthritis Joint Other	Nociceptive Pain
M0220	Postimmunization arthropathy, unspecified site	Arthritis Joint Other	Nociceptive Pain
M0221	Postimmunization arthropathy, shoulder	Arthritis Joint Upper Limb	Nociceptive Pain
M02211	Postimmunization arthropathy, right shoulder	Arthritis Joint Upper Limb	Nociceptive Pain
M02212	Postimmunization arthropathy, left shoulder	Arthritis Joint Upper Limb	Nociceptive Pain
M02219	Postimmunization arthropathy, unspecified shoulder	Arthritis Joint Upper Limb	Nociceptive Pain
M0222	Postimmunization arthropathy, elbow	Arthritis Joint Upper Limb	Nociceptive Pain
M02221	Postimmunization arthropathy, right elbow	Arthritis Joint Upper Limb	Nociceptive Pain
M02222	Postimmunization arthropathy, left elbow	Arthritis Joint Upper Limb	Nociceptive Pain
M02229	Postimmunization arthropathy, unspecified elbow	Arthritis Joint Upper Limb	Nociceptive Pain
M0223	Postimmunization arthropathy, wrist	Arthritis Joint Upper Limb	Nociceptive Pain

© 2024 Peterson MD et al. *JAMA Neurology.*

M02231	Postimmunization arthropathy, right wrist	Arthritis Joint Upper Limb	Nociceptive Pain
M02232	Postimmunization arthropathy, left wrist	Arthritis Joint Upper Limb	Nociceptive Pain
M02239	Postimmunization arthropathy, unspecified wrist	Arthritis Joint Upper Limb	Nociceptive Pain
M0224	Postimmunization arthropathy, hand	Arthritis Joint Upper Limb	Nociceptive Pain
M02241	Postimmunization arthropathy, right hand	Arthritis Joint Upper Limb	Nociceptive Pain
M02242	Postimmunization arthropathy, left hand	Arthritis Joint Upper Limb	Nociceptive Pain
M02249	Postimmunization arthropathy, unspecified hand	Arthritis Joint Upper Limb	Nociceptive Pain
M0225	Postimmunization arthropathy, hip	Arthritis Joint Spine and Hips	Nociceptive Pain
M02251	Postimmunization arthropathy, right hip	Arthritis Joint Spine and Hips	Nociceptive Pain
M02252	Postimmunization arthropathy, left hip	Arthritis Joint Spine and Hips	Nociceptive Pain
M02259	Postimmunization arthropathy, unspecified hip	Arthritis Joint Spine and Hips	Nociceptive Pain
M0226	Postimmunization arthropathy, knee	Arthritis Joint Lower Limb	Nociceptive Pain
M02261	Postimmunization arthropathy, right knee	Arthritis Joint Lower Limb	Nociceptive Pain
M02262	Postimmunization arthropathy, left knee	Arthritis Joint Lower Limb	Nociceptive Pain
M02269	Postimmunization arthropathy, unspecified knee	Arthritis Joint Lower Limb	Nociceptive Pain
M0227	Postimmunization arthropathy, ankle and foot	Arthritis Joint Lower Limb	Nociceptive Pain
M02271	Postimmunization arthropathy, right ankle and foot	Arthritis Joint Lower Limb	Nociceptive Pain
M02272	Postimmunization arthropathy, left ankle and foot	Arthritis Joint Lower Limb	Nociceptive Pain
M02279	Postimmunization arthropathy, unspecified ankle and foot	Arthritis Joint Lower Limb	Nociceptive Pain
M0228	Postimmunization arthropathy, vertebrae	Arthritis Joint Spine and Hips	Nociceptive Pain
M0229	Postimmunization arthropathy, multiple sites	Arthritis Joint Other	Nociceptive Pain
M023	Reiter's disease	Arthritis Joint Other	Nociceptive Pain
M0230	Reiter's disease, unspecified site	Arthritis Joint Other	Nociceptive Pain
M0231	Reiter's disease, shoulder	Arthritis Joint Upper Limb	Nociceptive Pain
M02311	Reiter's disease, right shoulder	Arthritis Joint Upper Limb	Nociceptive Pain
M02312	Reiter's disease, left shoulder	Arthritis Joint Upper Limb	Nociceptive Pain
M02319	Reiter's disease, unspecified shoulder	Arthritis Joint Upper Limb	Nociceptive Pain
M0232	Reiter's disease, elbow	Arthritis Joint Upper Limb	Nociceptive Pain
M02321	Reiter's disease, right elbow	Arthritis Joint Upper Limb	Nociceptive Pain
M02322	Reiter's disease, left elbow	Arthritis Joint Upper Limb	Nociceptive Pain
M02329	Reiter's disease, unspecified elbow	Arthritis Joint Upper Limb	Nociceptive Pain
M0233	Reiter's disease, wrist	Arthritis Joint Upper Limb	Nociceptive Pain
M02331	Reiter's disease, right wrist	Arthritis Joint Upper Limb	Nociceptive Pain
M02332	Reiter's disease, left wrist	Arthritis Joint Upper Limb	Nociceptive Pain
M02339	Reiter's disease, unspecified wrist	Arthritis Joint Upper Limb	Nociceptive Pain
M0234	Reiter's disease, hand	Arthritis Joint Upper Limb	Nociceptive Pain
M02341	Reiter's disease, right hand	Arthritis Joint Upper Limb	Nociceptive Pain

© 2024 Peterson MD et al. *JAMA Neurology.*

M02342	Reiter's disease, left hand	Arthritis Joint Upper Limb	Nociceptive Pain
M02349	Reiter's disease, unspecified hand	Arthritis Joint Upper Limb	Nociceptive Pain
M0235	Reiter's disease, hip	Arthritis Joint Spine and Hips	Nociceptive Pain
M02351	Reiter's disease, right hip	Arthritis Joint Spine and Hips	Nociceptive Pain
M02352	Reiter's disease, left hip	Arthritis Joint Spine and Hips	Nociceptive Pain
M02359	Reiter's disease, unspecified hip	Arthritis Joint Spine and Hips	Nociceptive Pain
M0236	Reiter's disease, knee	Arthritis Joint Lower Limb	Nociceptive Pain
M02361	Reiter's disease, right knee	Arthritis Joint Lower Limb	Nociceptive Pain
M02362	Reiter's disease, left knee	Arthritis Joint Lower Limb	Nociceptive Pain
M02369	Reiter's disease, unspecified knee	Arthritis Joint Lower Limb	Nociceptive Pain
M0237	Reiter's disease, ankle and foot	Arthritis Joint Lower Limb	Nociceptive Pain
M02371	Reiter's disease, right ankle and foot	Arthritis Joint Lower Limb	Nociceptive Pain
M02372	Reiter's disease, left ankle and foot	Arthritis Joint Lower Limb	Nociceptive Pain
M02379	Reiter's disease, unspecified ankle and foot	Arthritis Joint Lower Limb	Nociceptive Pain
M0238	Reiter's disease, vertebrae	Arthritis Joint Spine and Hips	Nociceptive Pain
M0239	Reiter's disease, multiple sites	Arthritis Joint Other	Nociceptive Pain
M028	Other reactive arthropathies	Arthritis Joint Other	Nociceptive Pain
M0280	Other reactive arthropathies, unspecified site	Arthritis Joint Other	Nociceptive Pain
M0281	Other reactive arthropathies, shoulder	Arthritis Joint Upper Limb	Nociceptive Pain
M02811	Other reactive arthropathies, right shoulder	Arthritis Joint Upper Limb	Nociceptive Pain
M02812	Other reactive arthropathies, left shoulder	Arthritis Joint Upper Limb	Nociceptive Pain
M02819	Other reactive arthropathies, unspecified shoulder	Arthritis Joint Upper Limb	Nociceptive Pain
M0282	Other reactive arthropathies, elbow	Arthritis Joint Upper Limb	Nociceptive Pain
M02821	Other reactive arthropathies, right elbow	Arthritis Joint Upper Limb	Nociceptive Pain
M02822	Other reactive arthropathies, left elbow	Arthritis Joint Upper Limb	Nociceptive Pain
M02829	Other reactive arthropathies, unspecified elbow	Arthritis Joint Upper Limb	Nociceptive Pain
M0283	Other reactive arthropathies, wrist	Arthritis Joint Upper Limb	Nociceptive Pain
M02831	Other reactive arthropathies, right wrist	Arthritis Joint Upper Limb	Nociceptive Pain
M02832	Other reactive arthropathies, left wrist	Arthritis Joint Upper Limb	Nociceptive Pain
M02839	Other reactive arthropathies, unspecified wrist	Arthritis Joint Upper Limb	Nociceptive Pain
M0284	Other reactive arthropathies, hand	Arthritis Joint Upper Limb	Nociceptive Pain
M02841	Other reactive arthropathies, right hand	Arthritis Joint Upper Limb	Nociceptive Pain
M02842	Other reactive arthropathies, left hand	Arthritis Joint Upper Limb	Nociceptive Pain
M02849	Other reactive arthropathies, unspecified hand	Arthritis Joint Upper Limb	Nociceptive Pain
M0285	Other reactive arthropathies, hip	Arthritis Joint Spine and Hips	Nociceptive Pain
M02851	Other reactive arthropathies, right hip	Arthritis Joint Spine and Hips	Nociceptive Pain
M02852	Other reactive arthropathies, left hip	Arthritis Joint Spine and Hips	Nociceptive Pain

© 2024 Peterson MD et al. *JAMA Neurology.*

M02859	Other reactive arthropathies, unspecified hip	Arthritis Joint Spine and Hips	Nociceptive Pain
M0286	Other reactive arthropathies, knee	Arthritis Joint Lower Limb	Nociceptive Pain
M02861	Other reactive arthropathies, right knee	Arthritis Joint Lower Limb	Nociceptive Pain
M02862	Other reactive arthropathies, left knee	Arthritis Joint Lower Limb	Nociceptive Pain
M02869	Other reactive arthropathies, unspecified knee	Arthritis Joint Lower Limb	Nociceptive Pain
M0287	Other reactive arthropathies, ankle and foot	Arthritis Joint Lower Limb	Nociceptive Pain
M02871	Other reactive arthropathies, right ankle and foot	Arthritis Joint Lower Limb	Nociceptive Pain
M02872	Other reactive arthropathies, left ankle and foot	Arthritis Joint Lower Limb	Nociceptive Pain
M02879	Other reactive arthropathies, unspecified ankle and foot	Arthritis Joint Lower Limb	Nociceptive Pain
M0288	Other reactive arthropathies, vertebrae	Arthritis Joint Spine and Hips	Nociceptive Pain
M0289	Other reactive arthropathies, multiple sites	Arthritis Joint Other	Nociceptive Pain
M029	Reactive arthropathy, unspecified	Arthritis Joint Other	Nociceptive Pain
M05	Rheumatoid arthritis with rheumatoid factor	Arthritis Joint Other	Nociceptive Pain
M050	Felty's syndrome	Arthritis Joint Other	Nociceptive Pain
M0500	Felty's syndrome, unspecified site	Arthritis Joint Other	Nociceptive Pain
M0501	Felty's syndrome, shoulder	Arthritis Joint Upper Limb	Nociceptive Pain
M05011	Felty's syndrome, right shoulder	Arthritis Joint Upper Limb	Nociceptive Pain
M05012	Felty's syndrome, left shoulder	Arthritis Joint Upper Limb	Nociceptive Pain
M05019	Felty's syndrome, unspecified shoulder	Arthritis Joint Upper Limb	Nociceptive Pain
M0502	Felty's syndrome, elbow	Arthritis Joint Upper Limb	Nociceptive Pain
M05021	Felty's syndrome, right elbow	Arthritis Joint Upper Limb	Nociceptive Pain
M05022	Felty's syndrome, left elbow	Arthritis Joint Upper Limb	Nociceptive Pain
M05029	Felty's syndrome, unspecified elbow	Arthritis Joint Upper Limb	Nociceptive Pain
M0503	Felty's syndrome, wrist	Arthritis Joint Upper Limb	Nociceptive Pain
M05031	Felty's syndrome, right wrist	Arthritis Joint Upper Limb	Nociceptive Pain
M05032	Felty's syndrome, left wrist	Arthritis Joint Upper Limb	Nociceptive Pain
M05039	Felty's syndrome, unspecified wrist	Arthritis Joint Upper Limb	Nociceptive Pain
M0504	Felty's syndrome, hand	Arthritis Joint Upper Limb	Nociceptive Pain
M05041	Felty's syndrome, right hand	Arthritis Joint Upper Limb	Nociceptive Pain
M05042	Felty's syndrome, left hand	Arthritis Joint Upper Limb	Nociceptive Pain
M05049	Felty's syndrome, unspecified hand	Arthritis Joint Upper Limb	Nociceptive Pain
M0505	Felty's syndrome, hip	Arthritis Joint Spine and Hips	Nociceptive Pain
M05051	Felty's syndrome, right hip	Arthritis Joint Spine and Hips	Nociceptive Pain
M05052	Felty's syndrome, left hip	Arthritis Joint Spine and Hips	Nociceptive Pain
M05059	Felty's syndrome, unspecified hip	Arthritis Joint Spine and Hips	Nociceptive Pain
M0506	Felty's syndrome, knee	Arthritis Joint Lower Limb	Nociceptive Pain
M05061	Felty's syndrome, right knee	Arthritis Joint Lower Limb	Nociceptive Pain

© 2024 Peterson MD et al. *JAMA Neurology.*

M05062	Felty's syndrome, left knee	Arthritis Joint Lower Limb	Nociceptive Pain
M05069	Felty's syndrome, unspecified knee	Arthritis Joint Lower Limb	Nociceptive Pain
M0507	Felty's syndrome, ankle and foot	Arthritis Joint Lower Limb	Nociceptive Pain
M05071	Felty's syndrome, right ankle and foot	Arthritis Joint Lower Limb	Nociceptive Pain
M05072	Felty's syndrome, left ankle and foot	Arthritis Joint Lower Limb	Nociceptive Pain
M05079	Felty's syndrome, unspecified ankle and foot	Arthritis Joint Lower Limb	Nociceptive Pain
M0509	Felty's syndrome, multiple sites	Arthritis Joint Other	Nociceptive Pain
M051	Rheumatoid lung disease with rheumatoid arthritis	Arthritis Joint Other	Nociceptive Pain
M0510	Rheumatoid lung disease w rheumatoid arthritis of unsp site	Arthritis Joint Other	Nociceptive Pain
M0511	Rheumatoid lung disease w rheumatoid arthritis of shoulder	Arthritis Joint Upper Limb	Nociceptive Pain
M05111	Rheumatoid lung disease w rheumatoid arthritis of r shoulder	Arthritis Joint Upper Limb	Nociceptive Pain
M05112	Rheumatoid lung disease w rheumatoid arthritis of l shoulder	Arthritis Joint Upper Limb	Nociceptive Pain
M05119	Rheu lung disease w rheumatoid arthritis of unsp shoulder	Arthritis Joint Upper Limb	Nociceptive Pain
M0512	Rheumatoid lung disease with rheumatoid arthritis of elbow	Arthritis Joint Upper Limb	Nociceptive Pain
M05121	Rheumatoid lung disease w rheumatoid arthritis of r elbow	Arthritis Joint Upper Limb	Nociceptive Pain
M05122	Rheumatoid lung disease w rheumatoid arthritis of left elbow	Arthritis Joint Upper Limb	Nociceptive Pain
M05129	Rheumatoid lung disease w rheumatoid arthritis of unsp elbow	Arthritis Joint Upper Limb	Nociceptive Pain
M0513	Rheumatoid lung disease with rheumatoid arthritis of wrist	Arthritis Joint Upper Limb	Nociceptive Pain
M05131	Rheumatoid lung disease w rheumatoid arthritis of r wrist	Arthritis Joint Upper Limb	Nociceptive Pain
M05132	Rheumatoid lung disease w rheumatoid arthritis of left wrist	Arthritis Joint Upper Limb	Nociceptive Pain
M05139	Rheumatoid lung disease w rheumatoid arthritis of unsp wrist	Arthritis Joint Upper Limb	Nociceptive Pain
M0514	Rheumatoid lung disease with rheumatoid arthritis of hand	Arthritis Joint Upper Limb	Nociceptive Pain
M05141	Rheumatoid lung disease w rheumatoid arthritis of right hand	Arthritis Joint Upper Limb	Nociceptive Pain
M05142	Rheumatoid lung disease w rheumatoid arthritis of left hand	Arthritis Joint Upper Limb	Nociceptive Pain
M05149	Rheumatoid lung disease w rheumatoid arthritis of unsp hand	Arthritis Joint Upper Limb	Nociceptive Pain
M0515	Rheumatoid lung disease with rheumatoid arthritis of hip	Arthritis Joint Spine and Hips	Nociceptive Pain
M05151	Rheumatoid lung disease w rheumatoid arthritis of right hip	Arthritis Joint Spine and Hips	Nociceptive Pain
M05152	Rheumatoid lung disease w rheumatoid arthritis of left hip	Arthritis Joint Spine and Hips	Nociceptive Pain
M05159	Rheumatoid lung disease w rheumatoid arthritis of unsp hip	Arthritis Joint Spine and Hips	Nociceptive Pain
M0516	Rheumatoid lung disease with rheumatoid arthritis of knee	Arthritis Joint Lower Limb	Nociceptive Pain
M05161	Rheumatoid lung disease w rheumatoid arthritis of right knee	Arthritis Joint Lower Limb	Nociceptive Pain
M05162	Rheumatoid lung disease w rheumatoid arthritis of left knee	Arthritis Joint Lower Limb	Nociceptive Pain
M05169	Rheumatoid lung disease w rheumatoid arthritis of unsp knee	Arthritis Joint Lower Limb	Nociceptive Pain
M0517	Rheumatoid lung disease w rheumatoid arthritis of ank/ft	Arthritis Joint Lower Limb	Nociceptive Pain
M05171	Rheu lung disease w rheumatoid arthritis of right ank/ft	Arthritis Joint Lower Limb	Nociceptive Pain
M05172	Rheu lung disease w rheumatoid arthritis of left ank/ft	Arthritis Joint Lower Limb	Nociceptive Pain
M05179	Rheu lung disease w rheumatoid arthritis of unsp ank/ft	Arthritis Joint Lower Limb	Nociceptive Pain

© 2024 Peterson MD et al. *JAMA Neurology.*

M0519	Rheumatoid lung disease w rheumatoid arthritis mult site	Arthritis Joint Other	Nociceptive Pain
M052	Rheumatoid vasculitis with rheumatoid arthritis	Arthritis Joint Other	Nociceptive Pain
M0520	Rheumatoid vasculitis with rheumatoid arthritis of unsp site	Arthritis Joint Other	Nociceptive Pain
M0521	Rheumatoid vasculitis with rheumatoid arthritis of shoulder	Arthritis Joint Upper Limb	Nociceptive Pain
M05211	Rheumatoid vasculitis w rheumatoid arthritis of r shoulder	Arthritis Joint Upper Limb	Nociceptive Pain
M05212	Rheumatoid vasculitis w rheumatoid arthritis of l shoulder	Arthritis Joint Upper Limb	Nociceptive Pain
M05219	Rheu vasculitis w rheumatoid arthritis of unsp shoulder	Arthritis Joint Upper Limb	Nociceptive Pain
M0522	Rheumatoid vasculitis with rheumatoid arthritis of elbow	Arthritis Joint Upper Limb	Nociceptive Pain
M05221	Rheumatoid vasculitis w rheumatoid arthritis of right elbow	Arthritis Joint Upper Limb	Nociceptive Pain
M05222	Rheumatoid vasculitis w rheumatoid arthritis of left elbow	Arthritis Joint Upper Limb	Nociceptive Pain
M05229	Rheumatoid vasculitis w rheumatoid arthritis of unsp elbow	Arthritis Joint Upper Limb	Nociceptive Pain
M0523	Rheumatoid vasculitis with rheumatoid arthritis of wrist	Arthritis Joint Upper Limb	Nociceptive Pain
M05231	Rheumatoid vasculitis w rheumatoid arthritis of right wrist	Arthritis Joint Upper Limb	Nociceptive Pain
M05232	Rheumatoid vasculitis w rheumatoid arthritis of left wrist	Arthritis Joint Upper Limb	Nociceptive Pain
M05239	Rheumatoid vasculitis w rheumatoid arthritis of unsp wrist	Arthritis Joint Upper Limb	Nociceptive Pain
M0524	Rheumatoid vasculitis with rheumatoid arthritis of hand	Arthritis Joint Upper Limb	Nociceptive Pain
M05241	Rheumatoid vasculitis w rheumatoid arthritis of right hand	Arthritis Joint Upper Limb	Nociceptive Pain
M05242	Rheumatoid vasculitis with rheumatoid arthritis of left hand	Arthritis Joint Upper Limb	Nociceptive Pain
M05249	Rheumatoid vasculitis with rheumatoid arthritis of unsp hand	Arthritis Joint Upper Limb	Nociceptive Pain
M0525	Rheumatoid vasculitis with rheumatoid arthritis of hip	Arthritis Joint Spine and Hips	Nociceptive Pain
M05251	Rheumatoid vasculitis with rheumatoid arthritis of right hip	Arthritis Joint Spine and Hips	Nociceptive Pain
M05252	Rheumatoid vasculitis with rheumatoid arthritis of left hip	Arthritis Joint Spine and Hips	Nociceptive Pain
M05259	Rheumatoid vasculitis with rheumatoid arthritis of unsp hip	Arthritis Joint Spine and Hips	Nociceptive Pain
M0526	Rheumatoid vasculitis with rheumatoid arthritis of knee	Arthritis Joint Lower Limb	Nociceptive Pain
M05261	Rheumatoid vasculitis w rheumatoid arthritis of right knee	Arthritis Joint Lower Limb	Nociceptive Pain
M05262	Rheumatoid vasculitis with rheumatoid arthritis of left knee	Arthritis Joint Lower Limb	Nociceptive Pain
M05269	Rheumatoid vasculitis with rheumatoid arthritis of unsp knee	Arthritis Joint Lower Limb	Nociceptive Pain
M0527	Rheumatoid vasculitis w rheumatoid arthritis of ank/ft	Arthritis Joint Lower Limb	Nociceptive Pain
M05271	Rheumatoid vasculitis w rheumatoid arthritis of right ank/ft	Arthritis Joint Lower Limb	Nociceptive Pain
M05272	Rheumatoid vasculitis w rheumatoid arthritis of left ank/ft	Arthritis Joint Lower Limb	Nociceptive Pain
M05279	Rheumatoid vasculitis w rheumatoid arthritis of unsp ank/ft	Arthritis Joint Lower Limb	Nociceptive Pain
M0529	Rheumatoid vasculitis w rheumatoid arthritis mult site	Arthritis Joint Other	Nociceptive Pain
M053	Rheumatoid heart disease with rheumatoid arthritis	Arthritis Joint Other	Nociceptive Pain
M0530	Rheumatoid heart disease w rheumatoid arthritis of unsp site	Arthritis Joint Other	Nociceptive Pain
M0531	Rheumatoid heart disease w rheumatoid arthritis of shoulder	Arthritis Joint Upper Limb	Nociceptive Pain
M05311	Rheu heart disease w rheumatoid arthritis of r shoulder	Arthritis Joint Upper Limb	Nociceptive Pain
M05312	Rheu heart disease w rheumatoid arthritis of l shoulder	Arthritis Joint Upper Limb	Nociceptive Pain

© 2024 Peterson MD et al. *JAMA Neurology.*

M05319	Rheu heart disease w rheumatoid arthritis of unsp shoulder	Arthritis Joint Upper Limb	Nociceptive Pain
M0532	Rheumatoid heart disease with rheumatoid arthritis of elbow	Arthritis Joint Upper Limb	Nociceptive Pain
M05321	Rheumatoid heart disease w rheumatoid arthritis of r elbow	Arthritis Joint Upper Limb	Nociceptive Pain
M05322	Rheumatoid heart disease w rheumatoid arthritis of l elbow	Arthritis Joint Upper Limb	Nociceptive Pain
M05329	Rheu heart disease w rheumatoid arthritis of unsp elbow	Arthritis Joint Upper Limb	Nociceptive Pain
M0533	Rheumatoid heart disease with rheumatoid arthritis of wrist	Arthritis Joint Upper Limb	Nociceptive Pain
M05331	Rheumatoid heart disease w rheumatoid arthritis of r wrist	Arthritis Joint Upper Limb	Nociceptive Pain
M05332	Rheumatoid heart disease w rheumatoid arthritis of l wrist	Arthritis Joint Upper Limb	Nociceptive Pain
M05339	Rheu heart disease w rheumatoid arthritis of unsp wrist	Arthritis Joint Upper Limb	Nociceptive Pain
M0534	Rheumatoid heart disease with rheumatoid arthritis of hand	Arthritis Joint Upper Limb	Nociceptive Pain
M05341	Rheu heart disease w rheumatoid arthritis of right hand	Arthritis Joint Upper Limb	Nociceptive Pain
M05342	Rheumatoid heart disease w rheumatoid arthritis of left hand	Arthritis Joint Upper Limb	Nociceptive Pain
M05349	Rheumatoid heart disease w rheumatoid arthritis of unsp hand	Arthritis Joint Upper Limb	Nociceptive Pain
M0535	Rheumatoid heart disease with rheumatoid arthritis of hip	Arthritis Joint Spine and Hips	Nociceptive Pain
M05351	Rheumatoid heart disease w rheumatoid arthritis of right hip	Arthritis Joint Spine and Hips	Nociceptive Pain
M05352	Rheumatoid heart disease w rheumatoid arthritis of left hip	Arthritis Joint Spine and Hips	Nociceptive Pain
M05359	Rheumatoid heart disease w rheumatoid arthritis of unsp hip	Arthritis Joint Spine and Hips	Nociceptive Pain
M0536	Rheumatoid heart disease with rheumatoid arthritis of knee	Arthritis Joint Lower Limb	Nociceptive Pain
M05361	Rheu heart disease w rheumatoid arthritis of right knee	Arthritis Joint Lower Limb	Nociceptive Pain
M05362	Rheumatoid heart disease w rheumatoid arthritis of left knee	Arthritis Joint Lower Limb	Nociceptive Pain
M05369	Rheumatoid heart disease w rheumatoid arthritis of unsp knee	Arthritis Joint Lower Limb	Nociceptive Pain
M0537	Rheumatoid heart disease w rheumatoid arthritis of ank/ft	Arthritis Joint Lower Limb	Nociceptive Pain
M05371	Rheu heart disease w rheumatoid arthritis of right ank/ft	Arthritis Joint Lower Limb	Nociceptive Pain
M05372	Rheu heart disease w rheumatoid arthritis of left ank/ft	Arthritis Joint Lower Limb	Nociceptive Pain
M05379	Rheu heart disease w rheumatoid arthritis of unsp ank/ft	Arthritis Joint Lower Limb	Nociceptive Pain
M0539	Rheumatoid heart disease w rheumatoid arthritis mult site	Arthritis Joint Other	Nociceptive Pain
M054	Rheumatoid myopathy with rheumatoid arthritis	Arthritis Joint Other	Nociceptive Pain
M0540	Rheumatoid myopathy with rheumatoid arthritis of unsp site	Arthritis Joint Other	Nociceptive Pain
M0541	Rheumatoid myopathy with rheumatoid arthritis of shoulder	Arthritis Joint Upper Limb	Nociceptive Pain
M05411	Rheumatoid myopathy w rheumatoid arthritis of right shoulder	Arthritis Joint Upper Limb	Nociceptive Pain
M05412	Rheumatoid myopathy w rheumatoid arthritis of left shoulder	Arthritis Joint Upper Limb	Nociceptive Pain
M05419	Rheumatoid myopathy w rheumatoid arthritis of unsp shoulder	Arthritis Joint Upper Limb	Nociceptive Pain
M0542	Rheumatoid myopathy with rheumatoid arthritis of elbow	Arthritis Joint Upper Limb	Nociceptive Pain
M05421	Rheumatoid myopathy with rheumatoid arthritis of right elbow	Arthritis Joint Upper Limb	Nociceptive Pain
M05422	Rheumatoid myopathy with rheumatoid arthritis of left elbow	Arthritis Joint Upper Limb	Nociceptive Pain

© 2024 Peterson MD et al. *JAMA Neurology.*

M05429	Rheumatoid myopathy with rheumatoid arthritis of unsp elbow	Arthritis Joint Upper Limb	Nociceptive Pain
M0543	Rheumatoid myopathy with rheumatoid arthritis of wrist	Arthritis Joint Upper Limb	Nociceptive Pain
M05431	Rheumatoid myopathy with rheumatoid arthritis of right wrist	Arthritis Joint Upper Limb	Nociceptive Pain
M05432	Rheumatoid myopathy with rheumatoid arthritis of left wrist	Arthritis Joint Upper Limb	Nociceptive Pain
M05439	Rheumatoid myopathy with rheumatoid arthritis of unsp wrist	Arthritis Joint Upper Limb	Nociceptive Pain
M0544	Rheumatoid myopathy with rheumatoid arthritis of hand	Arthritis Joint Upper Limb	Nociceptive Pain
M05441	Rheumatoid myopathy with rheumatoid arthritis of right hand	Arthritis Joint Upper Limb	Nociceptive Pain
M05442	Rheumatoid myopathy with rheumatoid arthritis of left hand	Arthritis Joint Upper Limb	Nociceptive Pain
M05449	Rheumatoid myopathy with rheumatoid arthritis of unsp hand	Arthritis Joint Upper Limb	Nociceptive Pain
M0545	Rheumatoid myopathy with rheumatoid arthritis of hip	Arthritis Joint Spine and Hips	Nociceptive Pain
M05451	Rheumatoid myopathy with rheumatoid arthritis of right hip	Arthritis Joint Spine and Hips	Nociceptive Pain
M05452	Rheumatoid myopathy with rheumatoid arthritis of left hip	Arthritis Joint Spine and Hips	Nociceptive Pain
M05459	Rheumatoid myopathy with rheumatoid arthritis of unsp hip	Arthritis Joint Spine and Hips	Nociceptive Pain
M0546	Rheumatoid myopathy with rheumatoid arthritis of knee	Arthritis Joint Lower Limb	Nociceptive Pain
M05461	Rheumatoid myopathy with rheumatoid arthritis of right knee	Arthritis Joint Lower Limb	Nociceptive Pain
M05462	Rheumatoid myopathy with rheumatoid arthritis of left knee	Arthritis Joint Lower Limb	Nociceptive Pain
M05469	Rheumatoid myopathy with rheumatoid arthritis of unsp knee	Arthritis Joint Lower Limb	Nociceptive Pain
M0547	Rheumatoid myopathy w rheumatoid arthritis of ankle and foot	Arthritis Joint Lower Limb	Nociceptive Pain
M05471	Rheumatoid myopathy w rheumatoid arthritis of right ank/ft	Arthritis Joint Lower Limb	Nociceptive Pain
M05472	Rheumatoid myopathy w rheumatoid arthritis of left ank/ft	Arthritis Joint Lower Limb	Nociceptive Pain
M05479	Rheumatoid myopathy w rheumatoid arthritis of unsp ank/ft	Arthritis Joint Lower Limb	Nociceptive Pain
M0549	Rheumatoid myopathy w rheumatoid arthritis of multiple sites	Arthritis Joint Other	Nociceptive Pain
M055	Rheumatoid polyneuropathy with rheumatoid arthritis	Arthritis Joint Other	Nociceptive Pain
M0550	Rheumatoid polyneurop w rheumatoid arthritis of unsp site	Arthritis Joint Other	Nociceptive Pain
M0551	Rheumatoid polyneuropathy w rheumatoid arthritis of shoulder	Arthritis Joint Upper Limb	Nociceptive Pain
M05511	Rheumatoid polyneurop w rheumatoid arthritis of r shoulder	Arthritis Joint Upper Limb	Nociceptive Pain
M05512	Rheumatoid polyneurop w rheumatoid arthritis of l shoulder	Arthritis Joint Upper Limb	Nociceptive Pain
M05519	Rheu polyneurop w rheumatoid arthritis of unsp shoulder	Arthritis Joint Upper Limb	Nociceptive Pain
M0552	Rheumatoid polyneuropathy with rheumatoid arthritis of elbow	Arthritis Joint Upper Limb	Nociceptive Pain
M05521	Rheumatoid polyneurop w rheumatoid arthritis of right elbow	Arthritis Joint Upper Limb	Nociceptive Pain
M05522	Rheumatoid polyneurop w rheumatoid arthritis of left elbow	Arthritis Joint Upper Limb	Nociceptive Pain
M05529	Rheumatoid polyneurop w rheumatoid arthritis of unsp elbow	Arthritis Joint Upper Limb	Nociceptive Pain
M0553	Rheumatoid polyneuropathy with rheumatoid arthritis of wrist	Arthritis Joint Upper Limb	Nociceptive Pain
M05531	Rheumatoid polyneurop w rheumatoid arthritis of right wrist	Arthritis Joint Upper Limb	Nociceptive Pain

© 2024 Peterson MD et al. *JAMA Neurology.*

M05532	Rheumatoid polyneurop w rheumatoid arthritis of left wrist	Arthritis Joint Upper Limb	Nociceptive Pain
M05539	Rheumatoid polyneurop w rheumatoid arthritis of unsp wrist	Arthritis Joint Upper Limb	Nociceptive Pain
M0554	Rheumatoid polyneuropathy with rheumatoid arthritis of hand	Arthritis Joint Upper Limb	Nociceptive Pain
M05541	Rheumatoid polyneurop w rheumatoid arthritis of right hand	Arthritis Joint Upper Limb	Nociceptive Pain
M05542	Rheumatoid polyneurop w rheumatoid arthritis of left hand	Arthritis Joint Upper Limb	Nociceptive Pain
M05549	Rheumatoid polyneurop w rheumatoid arthritis of unsp hand	Arthritis Joint Upper Limb	Nociceptive Pain
M0555	Rheumatoid polyneuropathy with rheumatoid arthritis of hip	Arthritis Joint Spine and Hips	Nociceptive Pain
M05551	Rheumatoid polyneurop w rheumatoid arthritis of right hip	Arthritis Joint Spine and Hips	Nociceptive Pain
M05552	Rheumatoid polyneuropathy w rheumatoid arthritis of left hip	Arthritis Joint Spine and Hips	Nociceptive Pain
M05559	Rheumatoid polyneuropathy w rheumatoid arthritis of unsp hip	Arthritis Joint Spine and Hips	Nociceptive Pain
M0556	Rheumatoid polyneuropathy with rheumatoid arthritis of knee	Arthritis Joint Lower Limb	Nociceptive Pain
M05561	Rheumatoid polyneurop w rheumatoid arthritis of right knee	Arthritis Joint Lower Limb	Nociceptive Pain
M05562	Rheumatoid polyneurop w rheumatoid arthritis of left knee	Arthritis Joint Lower Limb	Nociceptive Pain
M05569	Rheumatoid polyneurop w rheumatoid arthritis of unsp knee	Arthritis Joint Lower Limb	Nociceptive Pain
M0557	Rheumatoid polyneuropathy w rheumatoid arthritis of ank/ft	Arthritis Joint Lower Limb	Nociceptive Pain
M05571	Rheumatoid polyneurop w rheumatoid arthritis of right ank/ft	Arthritis Joint Lower Limb	Nociceptive Pain
M05572	Rheumatoid polyneurop w rheumatoid arthritis of left ank/ft	Arthritis Joint Lower Limb	Nociceptive Pain
M05579	Rheumatoid polyneurop w rheumatoid arthritis of unsp ank/ft	Arthritis Joint Lower Limb	Nociceptive Pain
M0559	Rheumatoid polyneuropathy w rheumatoid arthritis mult site	Arthritis Joint Other	Nociceptive Pain
M056	Rheumatoid arthritis w involvement of oth organs and systems	Arthritis Joint Other	Nociceptive Pain
M0560	Rheu arthritis of unsp site w involv of organs and systems	Arthritis Joint Other	Nociceptive Pain
M0561	Rheu arthritis of shoulder w involv of organs and systems	Arthritis Joint Upper Limb	Nociceptive Pain
M05611	Rheu arthritis of r shoulder w involv of organs and systems	Arthritis Joint Upper Limb	Nociceptive Pain
M05612	Rheu arthritis of l shoulder w involv of organs and systems	Arthritis Joint Upper Limb	Nociceptive Pain
M05619	Rheu arthrit of unsp shoulder w involv of organs and systems	Arthritis Joint Upper Limb	Nociceptive Pain
M0562	Rheumatoid arthritis of elbow w involv of organs and systems	Arthritis Joint Upper Limb	Nociceptive Pain
M05621	Rheu arthritis of r elbow w involv of organs and systems	Arthritis Joint Upper Limb	Nociceptive Pain
M05622	Rheu arthritis of l elbow w involv of organs and systems	Arthritis Joint Upper Limb	Nociceptive Pain
M05629	Rheu arthritis of unsp elbow w involv of organs and systems	Arthritis Joint Upper Limb	Nociceptive Pain
M0563	Rheumatoid arthritis of wrist w involv of organs and systems	Arthritis Joint Upper Limb	Nociceptive Pain
M05631	Rheu arthritis of r wrist w involv of organs and systems	Arthritis Joint Upper Limb	Nociceptive Pain
M05632	Rheu arthritis of l wrist w involv of organs and systems	Arthritis Joint Upper Limb	Nociceptive Pain
M05639	Rheu arthritis of unsp wrist w involv of organs and systems	Arthritis Joint Upper Limb	Nociceptive Pain
M0564	Rheumatoid arthritis of hand w involv of organs and systems	Arthritis Joint Upper Limb	Nociceptive Pain
M05641	Rheu arthritis of right hand w involv of organs and systems	Arthritis Joint Upper Limb	Nociceptive Pain
M05642	Rheu arthritis of left hand w involv of organs and systems	Arthritis Joint Upper Limb	Nociceptive Pain

© 2024 Peterson MD et al. *JAMA Neurology.*

M05649	Rheu arthritis of unsp hand w involv of organs and systems	Arthritis Joint Upper Limb	Nociceptive Pain
M0565	Rheumatoid arthritis of hip w involv of organs and systems	Arthritis Joint Spine and Hips	Nociceptive Pain
M05651	Rheu arthritis of right hip w involv of organs and systems	Arthritis Joint Spine and Hips	Nociceptive Pain
M05652	Rheu arthritis of left hip w involv of organs and systems	Arthritis Joint Spine and Hips	Nociceptive Pain
M05659	Rheu arthritis of unsp hip w involv of organs and systems	Arthritis Joint Spine and Hips	Nociceptive Pain
M0566	Rheumatoid arthritis of knee w involv of organs and systems	Arthritis Joint Lower Limb	Nociceptive Pain
M05661	Rheu arthritis of right knee w involv of organs and systems	Arthritis Joint Lower Limb	Nociceptive Pain
M05662	Rheu arthritis of left knee w involv of organs and systems	Arthritis Joint Lower Limb	Nociceptive Pain
M05669	Rheu arthritis of unsp knee w involv of organs and systems	Arthritis Joint Lower Limb	Nociceptive Pain
M0567	Rheu arthritis of ank/ft w involv of organs and systems	Arthritis Joint Lower Limb	Nociceptive Pain
M05671	Rheu arthrit of right ank/ft w involv of organs and systems	Arthritis Joint Lower Limb	Nociceptive Pain
M05672	Rheu arthritis of left ank/ft w involv of organs and systems	Arthritis Joint Lower Limb	Nociceptive Pain
M05679	Rheu arthritis of unsp ank/ft w involv of organs and systems	Arthritis Joint Lower Limb	Nociceptive Pain
M0569	Rheu arthritis mult site w involv of organs and systems	Arthritis Joint Other	Nociceptive Pain
M057	Rheumatoid arthritis w rheumatoid factor w/o org/sys involv	Arthritis Joint Other	Nociceptive Pain
M0570	Rheu arthritis w rheu factor of unsp site w/o org/sys involv	Arthritis Joint Other	Nociceptive Pain
M0571	Rheu arthritis w rheu factor of shoulder w/o org/sys involv	Arthritis Joint Upper Limb	Nociceptive Pain
M05711	Rheu arthrit w rheu factor of r shoulder w/o org/sys involv	Arthritis Joint Upper Limb	Nociceptive Pain
M05712	Rheu arthrit w rheu factor of l shoulder w/o org/sys involv	Arthritis Joint Upper Limb	Nociceptive Pain
M05719	Rheu arthrit w rheu factor of unsp shldr w/o org/sys involv	Arthritis Joint Upper Limb	Nociceptive Pain
M0572	Rheu arthritis w rheu factor of elbow w/o org/sys involv	Arthritis Joint Upper Limb	Nociceptive Pain
M05721	Rheu arthritis w rheu factor of r elbow w/o org/sys involv	Arthritis Joint Upper Limb	Nociceptive Pain
M05722	Rheu arthritis w rheu factor of l elbow w/o org/sys involv	Arthritis Joint Upper Limb	Nociceptive Pain
M05729	Rheu arthrit w rheu factor of unsp elbow w/o org/sys involv	Arthritis Joint Upper Limb	Nociceptive Pain
M0573	Rheu arthritis w rheu factor of wrist w/o org/sys involv	Arthritis Joint Upper Limb	Nociceptive Pain
M05731	Rheu arthritis w rheu factor of r wrist w/o org/sys involv	Arthritis Joint Upper Limb	Nociceptive Pain
M05732	Rheu arthritis w rheu factor of l wrist w/o org/sys involv	Arthritis Joint Upper Limb	Nociceptive Pain
M05739	Rheu arthrit w rheu factor of unsp wrist w/o org/sys involv	Arthritis Joint Upper Limb	Nociceptive Pain
M0574	Rheu arthritis w rheu factor of hand w/o org/sys involv	Arthritis Joint Upper Limb	Nociceptive Pain
M05741	Rheu arthritis w rheu factor of r hand w/o org/sys involv	Arthritis Joint Upper Limb	Nociceptive Pain
M05742	Rheu arthritis w rheu factor of left hand w/o org/sys involv	Arthritis Joint Upper Limb	Nociceptive Pain
M05749	Rheu arthritis w rheu factor of unsp hand w/o org/sys involv	Arthritis Joint Upper Limb	Nociceptive Pain
M0575	Rheu arthritis w rheumatoid factor of hip w/o org/sys involv	Arthritis Joint Spine and Hips	Nociceptive Pain
M05751	Rheu arthritis w rheu factor of right hip w/o org/sys involv	Arthritis Joint Spine and Hips	Nociceptive Pain
M05752	Rheu arthritis w rheu factor of left hip w/o org/sys involv	Arthritis Joint Spine and Hips	Nociceptive Pain
M05759	Rheu arthritis w rheu factor of unsp hip w/o org/sys involv	Arthritis Joint Spine and Hips	Nociceptive Pain
M0576	Rheu arthritis w rheu factor of knee w/o org/sys involv	Arthritis Joint Lower Limb	Nociceptive Pain

© 2024 Peterson MD et al. *JAMA Neurology.*

M05761	Rheu arthritis w rheu factor of r knee w/o org/sys involv	Arthritis Joint Lower Limb	Nociceptive Pain
M05762	Rheu arthritis w rheu factor of left knee w/o org/sys involv	Arthritis Joint Lower Limb	Nociceptive Pain
M05769	Rheu arthritis w rheu factor of unsp knee w/o org/sys involv	Arthritis Joint Lower Limb	Nociceptive Pain
M0577	Rheu arthritis w rheu factor of ank/ft w/o org/sys involv	Arthritis Joint Lower Limb	Nociceptive Pain
M05771	Rheu arthrit w rheu fctr of right ank/ft w/o org/sys involv	Arthritis Joint Lower Limb	Nociceptive Pain
M05772	Rheu arthrit w rheu factor of left ank/ft w/o org/sys involv	Arthritis Joint Lower Limb	Nociceptive Pain
M05779	Rheu arthrit w rheu factor of unsp ank/ft w/o org/sys involv	Arthritis Joint Lower Limb	Nociceptive Pain
M0579	Rheu arthritis w rheu factor mult site w/o org/sys involv	Arthritis Joint Other	Nociceptive Pain
M057A	Rheu arthrit with rheu factor of oth site w/o org/sys invl	Arthritis Joint Other	Nociceptive Pain
M058	Other rheumatoid arthritis with rheumatoid factor	Arthritis Joint Other	Nociceptive Pain
M0580	Oth rheumatoid arthritis with rheumatoid factor of unsp site	Arthritis Joint Other	Nociceptive Pain
M0581	Oth rheumatoid arthritis with rheumatoid factor of shoulder	Arthritis Joint Upper Limb	Nociceptive Pain
M05811	Oth rheumatoid arthritis w rheumatoid factor of r shoulder	Arthritis Joint Upper Limb	Nociceptive Pain
M05812	Oth rheumatoid arthritis w rheumatoid factor of l shoulder	Arthritis Joint Upper Limb	Nociceptive Pain
M05819	Oth rheu arthritis w rheumatoid factor of unsp shoulder	Arthritis Joint Upper Limb	Nociceptive Pain
M0582	Other rheumatoid arthritis with rheumatoid factor of elbow	Arthritis Joint Upper Limb	Nociceptive Pain
M05821	Oth rheumatoid arthritis w rheumatoid factor of right elbow	Arthritis Joint Upper Limb	Nociceptive Pain
M05822	Oth rheumatoid arthritis w rheumatoid factor of left elbow	Arthritis Joint Upper Limb	Nociceptive Pain
M05829	Oth rheumatoid arthritis w rheumatoid factor of unsp elbow	Arthritis Joint Upper Limb	Nociceptive Pain
M0583	Other rheumatoid arthritis with rheumatoid factor of wrist	Arthritis Joint Upper Limb	Nociceptive Pain
M05831	Oth rheumatoid arthritis w rheumatoid factor of right wrist	Arthritis Joint Upper Limb	Nociceptive Pain
M05832	Oth rheumatoid arthritis w rheumatoid factor of left wrist	Arthritis Joint Upper Limb	Nociceptive Pain
M05839	Oth rheumatoid arthritis w rheumatoid factor of unsp wrist	Arthritis Joint Upper Limb	Nociceptive Pain
M0584	Other rheumatoid arthritis with rheumatoid factor of hand	Arthritis Joint Upper Limb	Nociceptive Pain
M05841	Oth rheumatoid arthritis w rheumatoid factor of right hand	Arthritis Joint Upper Limb	Nociceptive Pain
M05842	Oth rheumatoid arthritis with rheumatoid factor of left hand	Arthritis Joint Upper Limb	Nociceptive Pain
M05849	Oth rheumatoid arthritis with rheumatoid factor of unsp hand	Arthritis Joint Upper Limb	Nociceptive Pain
M0585	Other rheumatoid arthritis with rheumatoid factor of hip	Arthritis Joint Spine and Hips	Nociceptive Pain
M05851	Oth rheumatoid arthritis with rheumatoid factor of right hip	Arthritis Joint Spine and Hips	Nociceptive Pain
M05852	Oth rheumatoid arthritis with rheumatoid factor of left hip	Arthritis Joint Spine and Hips	Nociceptive Pain
M05859	Oth rheumatoid arthritis with rheumatoid factor of unsp hip	Arthritis Joint Spine and Hips	Nociceptive Pain
M0586	Other rheumatoid arthritis with rheumatoid factor of knee	Arthritis Joint Lower Limb	Nociceptive Pain
M05861	Oth rheumatoid arthritis w rheumatoid factor of right knee	Arthritis Joint Lower Limb	Nociceptive Pain
M05862	Oth rheumatoid arthritis with rheumatoid factor of left knee	Arthritis Joint Lower Limb	Nociceptive Pain
M05869	Oth rheumatoid arthritis with rheumatoid factor of unsp knee	Arthritis Joint Lower Limb	Nociceptive Pain
M0587	Oth rheumatoid arthritis w rheumatoid factor of ank/ft	Arthritis Joint Lower Limb	Nociceptive Pain
M05871	Oth rheumatoid arthritis w rheumatoid factor of right ank/ft	Arthritis Joint Lower Limb	Nociceptive Pain

© 2024 Peterson MD et al. *JAMA Neurology.*

M05872	Oth rheumatoid arthritis w rheumatoid factor of left ank/ft	Arthritis Joint Lower Limb	Nociceptive Pain
M05879	Oth rheumatoid arthritis w rheumatoid factor of unsp ank/ft	Arthritis Joint Lower Limb	Nociceptive Pain
M0589	Oth rheumatoid arthritis w rheumatoid factor mult site	Arthritis Joint Other	Nociceptive Pain
M058A	Other rheu arthritis with rheumatoid factor of oth site	Arthritis Joint Other	Nociceptive Pain
M059	Rheumatoid arthritis with rheumatoid factor, unspecified	Arthritis Joint Other	Nociceptive Pain
M06	Other rheumatoid arthritis	Arthritis Joint Other	Nociceptive Pain
M060	Rheumatoid arthritis without rheumatoid factor	Arthritis Joint Other	Nociceptive Pain
M0600	Rheumatoid arthritis without rheumatoid factor, unsp site	Arthritis Joint Other	Nociceptive Pain
M0601	Rheumatoid arthritis without rheumatoid factor, shoulder	Arthritis Joint Upper Limb	Nociceptive Pain
M06011	Rheumatoid arthritis w/o rheumatoid factor, right shoulder	Arthritis Joint Upper Limb	Nociceptive Pain
M06012	Rheumatoid arthritis w/o rheumatoid factor, left shoulder	Arthritis Joint Upper Limb	Nociceptive Pain
M06019	Rheumatoid arthritis w/o rheumatoid factor, unsp shoulder	Arthritis Joint Upper Limb	Nociceptive Pain
M0602	Rheumatoid arthritis without rheumatoid factor, elbow	Arthritis Joint Upper Limb	Nociceptive Pain
M06021	Rheumatoid arthritis without rheumatoid factor, right elbow	Arthritis Joint Upper Limb	Nociceptive Pain
M06022	Rheumatoid arthritis without rheumatoid factor, left elbow	Arthritis Joint Upper Limb	Nociceptive Pain
M06029	Rheumatoid arthritis without rheumatoid factor, unsp elbow	Arthritis Joint Upper Limb	Nociceptive Pain
M0603	Rheumatoid arthritis without rheumatoid factor, wrist	Arthritis Joint Upper Limb	Nociceptive Pain
M06031	Rheumatoid arthritis without rheumatoid factor, right wrist	Arthritis Joint Upper Limb	Nociceptive Pain
M06032	Rheumatoid arthritis without rheumatoid factor, left wrist	Arthritis Joint Upper Limb	Nociceptive Pain
M06039	Rheumatoid arthritis without rheumatoid factor, unsp wrist	Arthritis Joint Upper Limb	Nociceptive Pain
M0604	Rheumatoid arthritis without rheumatoid factor, hand	Arthritis Joint Upper Limb	Nociceptive Pain
M06041	Rheumatoid arthritis without rheumatoid factor, right hand	Arthritis Joint Upper Limb	Nociceptive Pain
M06042	Rheumatoid arthritis without rheumatoid factor, left hand	Arthritis Joint Upper Limb	Nociceptive Pain
M06049	Rheumatoid arthritis without rheumatoid factor, unsp hand	Arthritis Joint Upper Limb	Nociceptive Pain
M0605	Rheumatoid arthritis without rheumatoid factor, hip	Arthritis Joint Spine and Hips	Nociceptive Pain
M06051	Rheumatoid arthritis without rheumatoid factor, right hip	Arthritis Joint Spine and Hips	Nociceptive Pain
M06052	Rheumatoid arthritis without rheumatoid factor, left hip	Arthritis Joint Spine and Hips	Nociceptive Pain
M06059	Rheumatoid arthritis without rheumatoid factor, unsp hip	Arthritis Joint Spine and Hips	Nociceptive Pain
M0606	Rheumatoid arthritis without rheumatoid factor, knee	Arthritis Joint Lower Limb	Nociceptive Pain
M06061	Rheumatoid arthritis without rheumatoid factor, right knee	Arthritis Joint Lower Limb	Nociceptive Pain
M06062	Rheumatoid arthritis without rheumatoid factor, left knee	Arthritis Joint Lower Limb	Nociceptive Pain
M06069	Rheumatoid arthritis without rheumatoid factor, unsp knee	Arthritis Joint Lower Limb	Nociceptive Pain
M0607	Rheumatoid arthritis w/o rheumatoid factor, ankle and foot	Arthritis Joint Lower Limb	Nociceptive Pain
M06071	Rheumatoid arthritis w/o rheumatoid factor, right ank/ft	Arthritis Joint Lower Limb	Nociceptive Pain
M06072	Rheumatoid arthritis w/o rheumatoid factor, left ank/ft	Arthritis Joint Lower Limb	Nociceptive Pain
M06079	Rheumatoid arthritis w/o rheumatoid factor, unsp ank/ft	Arthritis Joint Lower Limb	Nociceptive Pain
M0608	Rheumatoid arthritis without rheumatoid factor, vertebrae	Arthritis Joint Spine and Hips	Nociceptive Pain

© 2024 Peterson MD et al. *JAMA Neurology.*

M0609	Rheumatoid arthritis w/o rheumatoid factor, multiple sites	Arthritis Joint Other	Nociceptive Pain
M060A	Rheumatoid arthritis without rheumatoid factor, oth site	Arthritis Joint Other	Nociceptive Pain
M061	Adult-onset Still's disease	Arthritis Joint Other	Nociceptive Pain
M062	Rheumatoid bursitis	Arthritis Joint Other	Nociceptive Pain
M0620	Rheumatoid bursitis, unspecified site	Arthritis Joint Other	Nociceptive Pain
M0621	Rheumatoid bursitis, shoulder	Arthritis Joint Upper Limb	Nociceptive Pain
M06211	Rheumatoid bursitis, right shoulder	Arthritis Joint Upper Limb	Nociceptive Pain
M06212	Rheumatoid bursitis, left shoulder	Arthritis Joint Upper Limb	Nociceptive Pain
M06219	Rheumatoid bursitis, unspecified shoulder	Arthritis Joint Upper Limb	Nociceptive Pain
M0622	Rheumatoid bursitis, elbow	Arthritis Joint Upper Limb	Nociceptive Pain
M06221	Rheumatoid bursitis, right elbow	Arthritis Joint Upper Limb	Nociceptive Pain
M06222	Rheumatoid bursitis, left elbow	Arthritis Joint Upper Limb	Nociceptive Pain
M06229	Rheumatoid bursitis, unspecified elbow	Arthritis Joint Upper Limb	Nociceptive Pain
M0623	Rheumatoid bursitis, wrist	Arthritis Joint Upper Limb	Nociceptive Pain
M06231	Rheumatoid bursitis, right wrist	Arthritis Joint Upper Limb	Nociceptive Pain
M06232	Rheumatoid bursitis, left wrist	Arthritis Joint Upper Limb	Nociceptive Pain
M06239	Rheumatoid bursitis, unspecified wrist	Arthritis Joint Upper Limb	Nociceptive Pain
M0624	Rheumatoid bursitis, hand	Arthritis Joint Upper Limb	Nociceptive Pain
M06241	Rheumatoid bursitis, right hand	Arthritis Joint Upper Limb	Nociceptive Pain
M06242	Rheumatoid bursitis, left hand	Arthritis Joint Upper Limb	Nociceptive Pain
M06249	Rheumatoid bursitis, unspecified hand	Arthritis Joint Upper Limb	Nociceptive Pain
M0625	Rheumatoid bursitis, hip	Arthritis Joint Spine and Hips	Nociceptive Pain
M06251	Rheumatoid bursitis, right hip	Arthritis Joint Spine and Hips	Nociceptive Pain
M06252	Rheumatoid bursitis, left hip	Arthritis Joint Spine and Hips	Nociceptive Pain
M06259	Rheumatoid bursitis, unspecified hip	Arthritis Joint Spine and Hips	Nociceptive Pain
M0626	Rheumatoid bursitis, knee	Arthritis Joint Lower Limb	Nociceptive Pain
M06261	Rheumatoid bursitis, right knee	Arthritis Joint Lower Limb	Nociceptive Pain
M06262	Rheumatoid bursitis, left knee	Arthritis Joint Lower Limb	Nociceptive Pain
M06269	Rheumatoid bursitis, unspecified knee	Arthritis Joint Lower Limb	Nociceptive Pain
M0627	Rheumatoid bursitis, ankle and foot	Arthritis Joint Lower Limb	Nociceptive Pain
M06271	Rheumatoid bursitis, right ankle and foot	Arthritis Joint Lower Limb	Nociceptive Pain
M06272	Rheumatoid bursitis, left ankle and foot	Arthritis Joint Lower Limb	Nociceptive Pain
M06279	Rheumatoid bursitis, unspecified ankle and foot	Arthritis Joint Lower Limb	Nociceptive Pain
M0628	Rheumatoid bursitis, vertebrae	Arthritis Joint Spine and Hips	Nociceptive Pain
M0629	Rheumatoid bursitis, multiple sites	Arthritis Joint Other	Nociceptive Pain
M063	Rheumatoid nodule	Arthritis Joint Other	Nociceptive Pain
M0630	Rheumatoid nodule, unspecified site	Arthritis Joint Other	Nociceptive Pain

© 2024 Peterson MD et al. *JAMA Neurology.*

M0631	Rheumatoid nodule, shoulder	Arthritis Joint Upper Limb	Nociceptive Pain
M06311	Rheumatoid nodule, right shoulder	Arthritis Joint Upper Limb	Nociceptive Pain
M06312	Rheumatoid nodule, left shoulder	Arthritis Joint Upper Limb	Nociceptive Pain
M06319	Rheumatoid nodule, unspecified shoulder	Arthritis Joint Upper Limb	Nociceptive Pain
M0632	Rheumatoid nodule, elbow	Arthritis Joint Upper Limb	Nociceptive Pain
M06321	Rheumatoid nodule, right elbow	Arthritis Joint Upper Limb	Nociceptive Pain
M06322	Rheumatoid nodule, left elbow	Arthritis Joint Upper Limb	Nociceptive Pain
M06329	Rheumatoid nodule, unspecified elbow	Arthritis Joint Upper Limb	Nociceptive Pain
M0633	Rheumatoid nodule, wrist	Arthritis Joint Upper Limb	Nociceptive Pain
M06331	Rheumatoid nodule, right wrist	Arthritis Joint Upper Limb	Nociceptive Pain
M06332	Rheumatoid nodule, left wrist	Arthritis Joint Upper Limb	Nociceptive Pain
M06339	Rheumatoid nodule, unspecified wrist	Arthritis Joint Upper Limb	Nociceptive Pain
M0634	Rheumatoid nodule, hand	Arthritis Joint Upper Limb	Nociceptive Pain
M06341	Rheumatoid nodule, right hand	Arthritis Joint Upper Limb	Nociceptive Pain
M06342	Rheumatoid nodule, left hand	Arthritis Joint Upper Limb	Nociceptive Pain
M06349	Rheumatoid nodule, unspecified hand	Arthritis Joint Upper Limb	Nociceptive Pain
M0635	Rheumatoid nodule, hip	Arthritis Joint Spine and Hips	Nociceptive Pain
M06351	Rheumatoid nodule, right hip	Arthritis Joint Spine and Hips	Nociceptive Pain
M06352	Rheumatoid nodule, left hip	Arthritis Joint Spine and Hips	Nociceptive Pain
M06359	Rheumatoid nodule, unspecified hip	Arthritis Joint Spine and Hips	Nociceptive Pain
M0636	Rheumatoid nodule, knee	Arthritis Joint Lower Limb	Nociceptive Pain
M06361	Rheumatoid nodule, right knee	Arthritis Joint Lower Limb	Nociceptive Pain
M06362	Rheumatoid nodule, left knee	Arthritis Joint Lower Limb	Nociceptive Pain
M06369	Rheumatoid nodule, unspecified knee	Arthritis Joint Lower Limb	Nociceptive Pain
M0637	Rheumatoid nodule, ankle and foot	Arthritis Joint Lower Limb	Nociceptive Pain
M06371	Rheumatoid nodule, right ankle and foot	Arthritis Joint Lower Limb	Nociceptive Pain
M06372	Rheumatoid nodule, left ankle and foot	Arthritis Joint Lower Limb	Nociceptive Pain
M06379	Rheumatoid nodule, unspecified ankle and foot	Arthritis Joint Lower Limb	Nociceptive Pain
M0638	Rheumatoid nodule, vertebrae	Arthritis Joint Spine and Hips	Nociceptive Pain
M0639	Rheumatoid nodule, multiple sites	Arthritis Joint Other	Nociceptive Pain
M064	Inflammatory polyarthropathy	Arthritis Joint Other	Nociceptive Pain
M068	Other specified rheumatoid arthritis	Arthritis Joint Other	Nociceptive Pain
M0680	Other specified rheumatoid arthritis, unspecified site	Arthritis Joint Other	Nociceptive Pain
M0681	Other specified rheumatoid arthritis, shoulder	Arthritis Joint Upper Limb	Nociceptive Pain
M06811	Other specified rheumatoid arthritis, right shoulder	Arthritis Joint Upper Limb	Nociceptive Pain
M06812	Other specified rheumatoid arthritis, left shoulder	Arthritis Joint Upper Limb	Nociceptive Pain
M06819	Other specified rheumatoid arthritis, unspecified shoulder	Arthritis Joint Upper Limb	Nociceptive Pain

© 2024 Peterson MD et al. *JAMA Neurology.*

M0682	Other specified rheumatoid arthritis, elbow	Arthritis Joint Upper Limb	Nociceptive Pain
M06821	Other specified rheumatoid arthritis, right elbow	Arthritis Joint Upper Limb	Nociceptive Pain
M06822	Other specified rheumatoid arthritis, left elbow	Arthritis Joint Upper Limb	Nociceptive Pain
M06829	Other specified rheumatoid arthritis, unspecified elbow	Arthritis Joint Upper Limb	Nociceptive Pain
M0683	Other specified rheumatoid arthritis, wrist	Arthritis Joint Upper Limb	Nociceptive Pain
M06831	Other specified rheumatoid arthritis, right wrist	Arthritis Joint Upper Limb	Nociceptive Pain
M06832	Other specified rheumatoid arthritis, left wrist	Arthritis Joint Upper Limb	Nociceptive Pain
M06839	Other specified rheumatoid arthritis, unspecified wrist	Arthritis Joint Upper Limb	Nociceptive Pain
M0684	Other specified rheumatoid arthritis, hand	Arthritis Joint Upper Limb	Nociceptive Pain
M06841	Other specified rheumatoid arthritis, right hand	Arthritis Joint Upper Limb	Nociceptive Pain
M06842	Other specified rheumatoid arthritis, left hand	Arthritis Joint Upper Limb	Nociceptive Pain
M06849	Other specified rheumatoid arthritis, unspecified hand	Arthritis Joint Upper Limb	Nociceptive Pain
M0685	Other specified rheumatoid arthritis, hip	Arthritis Joint Spine and Hips	Nociceptive Pain
M06851	Other specified rheumatoid arthritis, right hip	Arthritis Joint Spine and Hips	Nociceptive Pain
M06852	Other specified rheumatoid arthritis, left hip	Arthritis Joint Spine and Hips	Nociceptive Pain
M06859	Other specified rheumatoid arthritis, unspecified hip	Arthritis Joint Spine and Hips	Nociceptive Pain
M0686	Other specified rheumatoid arthritis, knee	Arthritis Joint Lower Limb	Nociceptive Pain
M06861	Other specified rheumatoid arthritis, right knee	Arthritis Joint Lower Limb	Nociceptive Pain
M06862	Other specified rheumatoid arthritis, left knee	Arthritis Joint Lower Limb	Nociceptive Pain
M06869	Other specified rheumatoid arthritis, unspecified knee	Arthritis Joint Lower Limb	Nociceptive Pain
M0687	Other specified rheumatoid arthritis, ankle and foot	Arthritis Joint Lower Limb	Nociceptive Pain
M06871	Other specified rheumatoid arthritis, right ankle and foot	Arthritis Joint Lower Limb	Nociceptive Pain
M06872	Other specified rheumatoid arthritis, left ankle and foot	Arthritis Joint Lower Limb	Nociceptive Pain
M06879	Oth rheumatoid arthritis, unspecified ankle and foot	Arthritis Joint Lower Limb	Nociceptive Pain
M0688	Other specified rheumatoid arthritis, vertebrae	Arthritis Joint Spine and Hips	Nociceptive Pain
M0689	Other specified rheumatoid arthritis, multiple sites	Arthritis Joint Other	Nociceptive Pain
M068A	Other specified rheumatoid arthritis, other specified site	Arthritis Joint Other	Nociceptive Pain
M069	Rheumatoid arthritis, unspecified	Arthritis Joint Other	Nociceptive Pain
M08	Juvenile arthritis	Arthritis Joint Other	Nociceptive Pain
M080	Unspecified juvenile rheumatoid arthritis	Arthritis Joint Other	Nociceptive Pain
M0800	Unsp juvenile rheumatoid arthritis of unspecified site	Arthritis Joint Other	Nociceptive Pain
M0801	Unspecified juvenile rheumatoid arthritis, shoulder	Arthritis Joint Upper Limb	Nociceptive Pain
M08011	Unspecified juvenile rheumatoid arthritis, right shoulder	Arthritis Joint Upper Limb	Nociceptive Pain
M08012	Unspecified juvenile rheumatoid arthritis, left shoulder	Arthritis Joint Upper Limb	Nociceptive Pain
M08019	Unsp juvenile rheumatoid arthritis, unspecified shoulder	Arthritis Joint Upper Limb	Nociceptive Pain
M0802	Unspecified juvenile rheumatoid arthritis of elbow	Arthritis Joint Upper Limb	Nociceptive Pain
M08021	Unspecified juvenile rheumatoid arthritis, right elbow	Arthritis Joint Upper Limb	Nociceptive Pain

© 2024 Peterson MD et al. *JAMA Neurology.*

M08022	Unspecified juvenile rheumatoid arthritis, left elbow	Arthritis Joint Upper Limb	Nociceptive Pain
M08029	Unspecified juvenile rheumatoid arthritis, unspecified elbow	Arthritis Joint Upper Limb	Nociceptive Pain
M0803	Unspecified juvenile rheumatoid arthritis, wrist	Arthritis Joint Upper Limb	Nociceptive Pain
M08031	Unspecified juvenile rheumatoid arthritis, right wrist	Arthritis Joint Upper Limb	Nociceptive Pain
M08032	Unspecified juvenile rheumatoid arthritis, left wrist	Arthritis Joint Upper Limb	Nociceptive Pain
M08039	Unspecified juvenile rheumatoid arthritis, unspecified wrist	Arthritis Joint Upper Limb	Nociceptive Pain
M0804	Unspecified juvenile rheumatoid arthritis, hand	Arthritis Joint Upper Limb	Nociceptive Pain
M08041	Unspecified juvenile rheumatoid arthritis, right hand	Arthritis Joint Upper Limb	Nociceptive Pain
M08042	Unspecified juvenile rheumatoid arthritis, left hand	Arthritis Joint Upper Limb	Nociceptive Pain
M08049	Unspecified juvenile rheumatoid arthritis, unspecified hand	Arthritis Joint Upper Limb	Nociceptive Pain
M0805	Unspecified juvenile rheumatoid arthritis, hip	Arthritis Joint Spine and Hips	Nociceptive Pain
M08051	Unspecified juvenile rheumatoid arthritis, right hip	Arthritis Joint Spine and Hips	Nociceptive Pain
M08052	Unspecified juvenile rheumatoid arthritis, left hip	Arthritis Joint Spine and Hips	Nociceptive Pain
M08059	Unspecified juvenile rheumatoid arthritis, unspecified hip	Arthritis Joint Spine and Hips	Nociceptive Pain
M0806	Unspecified juvenile rheumatoid arthritis, knee	Arthritis Joint Lower Limb	Nociceptive Pain
M08061	Unspecified juvenile rheumatoid arthritis, right knee	Arthritis Joint Lower Limb	Nociceptive Pain
M08062	Unspecified juvenile rheumatoid arthritis, left knee	Arthritis Joint Lower Limb	Nociceptive Pain
M08069	Unspecified juvenile rheumatoid arthritis, unspecified knee	Arthritis Joint Lower Limb	Nociceptive Pain
M0807	Unspecified juvenile rheumatoid arthritis, ankle and foot	Arthritis Joint Lower Limb	Nociceptive Pain
M08071	Unsp juvenile rheumatoid arthritis, right ankle and foot	Arthritis Joint Lower Limb	Nociceptive Pain
M08072	Unsp juvenile rheumatoid arthritis, left ankle and foot	Arthritis Joint Lower Limb	Nociceptive Pain
M08079	Unsp juvenile rheumatoid arthritis, unsp ankle and foot	Arthritis Joint Lower Limb	Nociceptive Pain
M0808	Unspecified juvenile rheumatoid arthritis, vertebrae	Arthritis Joint Spine and Hips	Nociceptive Pain
M0809	Unspecified juvenile rheumatoid arthritis, multiple sites	Arthritis Joint Other	Nociceptive Pain
M080A	Unspecified juvenile rheumatoid arthritis, oth site	Arthritis Joint Other	Nociceptive Pain
M081	Juvenile ankylosing spondylitis	Arthritis Joint Other	Nociceptive Pain
M082	Juvenile rheumatoid arthritis with systemic onset	Arthritis Joint Other	Nociceptive Pain
M0820	Juvenile rheumatoid arthritis with systemic onset, unsp site	Arthritis Joint Other	Nociceptive Pain
M0821	Juvenile rheumatoid arthritis with systemic onset, shoulder	Arthritis Joint Upper Limb	Nociceptive Pain
M08211	Juvenile rheumatoid arthritis w systemic onset, r shoulder	Arthritis Joint Upper Limb	Nociceptive Pain
M08212	Juvenile rheumatoid arthritis w systemic onset, l shoulder	Arthritis Joint Upper Limb	Nociceptive Pain
M08219	Juvenile rheu arthritis w systemic onset, unsp shoulder	Arthritis Joint Upper Limb	Nociceptive Pain
M0822	Juvenile rheumatoid arthritis with systemic onset, elbow	Arthritis Joint Upper Limb	Nociceptive Pain
M08221	Juvenile rheumatoid arthritis w systemic onset, right elbow	Arthritis Joint Upper Limb	Nociceptive Pain
M08222	Juvenile rheumatoid arthritis w systemic onset, left elbow	Arthritis Joint Upper Limb	Nociceptive Pain
M08229	Juvenile rheumatoid arthritis w systemic onset, unsp elbow	Arthritis Joint Upper Limb	Nociceptive Pain
M0823	Juvenile rheumatoid arthritis with systemic onset, wrist	Arthritis Joint Upper Limb	Nociceptive Pain

© 2024 Peterson MD et al. *JAMA Neurology.*

M08231	Juvenile rheumatoid arthritis w systemic onset, right wrist	Arthritis Joint Upper Limb	Nociceptive Pain
M08232	Juvenile rheumatoid arthritis w systemic onset, left wrist	Arthritis Joint Upper Limb	Nociceptive Pain
M08239	Juvenile rheumatoid arthritis w systemic onset, unsp wrist	Arthritis Joint Upper Limb	Nociceptive Pain
M0824	Juvenile rheumatoid arthritis with systemic onset, hand	Arthritis Joint Upper Limb	Nociceptive Pain
M08241	Juvenile rheumatoid arthritis w systemic onset, right hand	Arthritis Joint Upper Limb	Nociceptive Pain
M08242	Juvenile rheumatoid arthritis with systemic onset, left hand	Arthritis Joint Upper Limb	Nociceptive Pain
M08249	Juvenile rheumatoid arthritis with systemic onset, unsp hand	Arthritis Joint Upper Limb	Nociceptive Pain
M0825	Juvenile rheumatoid arthritis with systemic onset, hip	Arthritis Joint Spine and Hips	Nociceptive Pain
M08251	Juvenile rheumatoid arthritis with systemic onset, right hip	Arthritis Joint Spine and Hips	Nociceptive Pain
M08252	Juvenile rheumatoid arthritis with systemic onset, left hip	Arthritis Joint Spine and Hips	Nociceptive Pain
M08259	Juvenile rheumatoid arthritis with systemic onset, unsp hip	Arthritis Joint Spine and Hips	Nociceptive Pain
M0826	Juvenile rheumatoid arthritis with systemic onset, knee	Arthritis Joint Lower Limb	Nociceptive Pain
M08261	Juvenile rheumatoid arthritis w systemic onset, right knee	Arthritis Joint Lower Limb	Nociceptive Pain
M08262	Juvenile rheumatoid arthritis with systemic onset, left knee	Arthritis Joint Lower Limb	Nociceptive Pain
M08269	Juvenile rheumatoid arthritis with systemic onset, unsp knee	Arthritis Joint Lower Limb	Nociceptive Pain
M0827	Juvenile rheumatoid arthritis w systemic onset, ank/ft	Arthritis Joint Lower Limb	Nociceptive Pain
M08271	Juvenile rheumatoid arthritis w systemic onset, right ank/ft	Arthritis Joint Lower Limb	Nociceptive Pain
M08272	Juvenile rheumatoid arthritis w systemic onset, left ank/ft	Arthritis Joint Lower Limb	Nociceptive Pain
M08279	Juvenile rheumatoid arthritis w systemic onset, unsp ank/ft	Arthritis Joint Lower Limb	Nociceptive Pain
M0828	Juvenile rheumatoid arthritis with systemic onset, vertebrae	Arthritis Joint Spine and Hips	Nociceptive Pain
M0829	Juvenile rheu arthritis w systemic onset, multiple sites	Arthritis Joint Other	Nociceptive Pain
M082A	Juvenile rheumatoid arthritis with systemic onset, oth site	Arthritis Joint Other	Nociceptive Pain
M083	Juvenile rheumatoid polyarthritis (seronegative)	Arthritis Joint Other	Nociceptive Pain
M084	Pauciarticular juvenile rheumatoid arthritis	Arthritis Joint Other	Nociceptive Pain
M0840	Pauciarticular juvenile rheumatoid arthritis, unsp site	Arthritis Joint Other	Nociceptive Pain
M0841	Pauciarticular juvenile rheumatoid arthritis, shoulder	Arthritis Joint Upper Limb	Nociceptive Pain
M08411	Pauciarticular juvenile rheumatoid arthritis, right shoulder	Arthritis Joint Upper Limb	Nociceptive Pain
M08412	Pauciarticular juvenile rheumatoid arthritis, left shoulder	Arthritis Joint Upper Limb	Nociceptive Pain
M08419	Pauciarticular juvenile rheumatoid arthritis, unsp shoulder	Arthritis Joint Upper Limb	Nociceptive Pain
M0842	Pauciarticular juvenile rheumatoid arthritis, elbow	Arthritis Joint Upper Limb	Nociceptive Pain
M08421	Pauciarticular juvenile rheumatoid arthritis, right elbow	Arthritis Joint Upper Limb	Nociceptive Pain
M08422	Pauciarticular juvenile rheumatoid arthritis, left elbow	Arthritis Joint Upper Limb	Nociceptive Pain
M08429	Pauciarticular juvenile rheumatoid arthritis, unsp elbow	Arthritis Joint Upper Limb	Nociceptive Pain
M0843	Pauciarticular juvenile rheumatoid arthritis, wrist	Arthritis Joint Upper Limb	Nociceptive Pain
M08431	Pauciarticular juvenile rheumatoid arthritis, right wrist	Arthritis Joint Upper Limb	Nociceptive Pain
M08432	Pauciarticular juvenile rheumatoid arthritis, left wrist	Arthritis Joint Upper Limb	Nociceptive Pain
M08439	Pauciarticular juvenile rheumatoid arthritis, unsp wrist	Arthritis Joint Upper Limb	Nociceptive Pain

© 2024 Peterson MD et al. *JAMA Neurology.*

M0844	Pauciarticular juvenile rheumatoid arthritis, hand	Arthritis Joint Upper Limb	Nociceptive Pain
M08441	Pauciarticular juvenile rheumatoid arthritis, right hand	Arthritis Joint Upper Limb	Nociceptive Pain
M08442	Pauciarticular juvenile rheumatoid arthritis, left hand	Arthritis Joint Upper Limb	Nociceptive Pain
M08449	Pauciarticular juvenile rheumatoid arthritis, unsp hand	Arthritis Joint Upper Limb	Nociceptive Pain
M0845	Pauciarticular juvenile rheumatoid arthritis, hip	Arthritis Joint Spine and Hips	Nociceptive Pain
M08451	Pauciarticular juvenile rheumatoid arthritis, right hip	Arthritis Joint Spine and Hips	Nociceptive Pain
M08452	Pauciarticular juvenile rheumatoid arthritis, left hip	Arthritis Joint Spine and Hips	Nociceptive Pain
M08459	Pauciarticular juvenile rheumatoid arthritis, unsp hip	Arthritis Joint Spine and Hips	Nociceptive Pain
M0846	Pauciarticular juvenile rheumatoid arthritis, knee	Arthritis Joint Lower Limb	Nociceptive Pain
M08461	Pauciarticular juvenile rheumatoid arthritis, right knee	Arthritis Joint Lower Limb	Nociceptive Pain
M08462	Pauciarticular juvenile rheumatoid arthritis, left knee	Arthritis Joint Lower Limb	Nociceptive Pain
M08469	Pauciarticular juvenile rheumatoid arthritis, unsp knee	Arthritis Joint Lower Limb	Nociceptive Pain
M0847	Pauciarticular juvenile rheumatoid arthritis, ankle and foot	Arthritis Joint Lower Limb	Nociceptive Pain
M08471	Pauciarticular juvenile rheumatoid arthritis, right ank/ft	Arthritis Joint Lower Limb	Nociceptive Pain
M08472	Pauciarticular juvenile rheumatoid arthritis, left ank/ft	Arthritis Joint Lower Limb	Nociceptive Pain
M08479	Pauciarticular juvenile rheumatoid arthritis, unsp ank/ft	Arthritis Joint Lower Limb	Nociceptive Pain
M0848	Pauciarticular juvenile rheumatoid arthritis, vertebrae	Arthritis Joint Spine and Hips	Nociceptive Pain
M084A	Pauciarticular juvenile rheumatoid arthritis, oth site	Arthritis Joint Other	Nociceptive Pain
M088	Other juvenile arthritis	Arthritis Joint Other	Nociceptive Pain
M0880	Other juvenile arthritis, unspecified site	Arthritis Joint Other	Nociceptive Pain
M0881	Other juvenile arthritis, shoulder	Arthritis Joint Upper Limb	Nociceptive Pain
M08811	Other juvenile arthritis, right shoulder	Arthritis Joint Upper Limb	Nociceptive Pain
M08812	Other juvenile arthritis, left shoulder	Arthritis Joint Upper Limb	Nociceptive Pain
M08819	Other juvenile arthritis, unspecified shoulder	Arthritis Joint Upper Limb	Nociceptive Pain
M0882	Other juvenile arthritis, elbow	Arthritis Joint Upper Limb	Nociceptive Pain
M08821	Other juvenile arthritis, right elbow	Arthritis Joint Upper Limb	Nociceptive Pain
M08822	Other juvenile arthritis, left elbow	Arthritis Joint Upper Limb	Nociceptive Pain
M08829	Other juvenile arthritis, unspecified elbow	Arthritis Joint Upper Limb	Nociceptive Pain
M0883	Other juvenile arthritis, wrist	Arthritis Joint Upper Limb	Nociceptive Pain
M08831	Other juvenile arthritis, right wrist	Arthritis Joint Upper Limb	Nociceptive Pain
M08832	Other juvenile arthritis, left wrist	Arthritis Joint Upper Limb	Nociceptive Pain
M08839	Other juvenile arthritis, unspecified wrist	Arthritis Joint Upper Limb	Nociceptive Pain
M0884	Other juvenile arthritis, hand	Arthritis Joint Upper Limb	Nociceptive Pain
M08841	Other juvenile arthritis, right hand	Arthritis Joint Upper Limb	Nociceptive Pain
M08842	Other juvenile arthritis, left hand	Arthritis Joint Upper Limb	Nociceptive Pain
M08849	Other juvenile arthritis, unspecified hand	Arthritis Joint Upper Limb	Nociceptive Pain
M0885	Other juvenile arthritis, hip	Arthritis Joint Spine and Hips	Nociceptive Pain

© 2024 Peterson MD et al. *JAMA Neurology.*

M08851	Other juvenile arthritis, right hip	Arthritis Joint Spine and Hips	Nociceptive Pain
M08852	Other juvenile arthritis, left hip	Arthritis Joint Spine and Hips	Nociceptive Pain
M08859	Other juvenile arthritis, unspecified hip	Arthritis Joint Spine and Hips	Nociceptive Pain
M0886	Other juvenile arthritis, knee	Arthritis Joint Lower Limb	Nociceptive Pain
M08861	Other juvenile arthritis, right knee	Arthritis Joint Lower Limb	Nociceptive Pain
M08862	Other juvenile arthritis, left knee	Arthritis Joint Lower Limb	Nociceptive Pain
M08869	Other juvenile arthritis, unspecified knee	Arthritis Joint Lower Limb	Nociceptive Pain
M0887	Other juvenile arthritis, ankle and foot	Arthritis Joint Lower Limb	Nociceptive Pain
M08871	Other juvenile arthritis, right ankle and foot	Arthritis Joint Lower Limb	Nociceptive Pain
M08872	Other juvenile arthritis, left ankle and foot	Arthritis Joint Lower Limb	Nociceptive Pain
M08879	Other juvenile arthritis, unspecified ankle and foot	Arthritis Joint Lower Limb	Nociceptive Pain
M0888	Other juvenile arthritis, other specified site	Arthritis Joint Other	Nociceptive Pain
M0889	Other juvenile arthritis, multiple sites	Arthritis Joint Other	Nociceptive Pain
M089	Juvenile arthritis, unspecified	Arthritis Joint Other	Nociceptive Pain
M0890	Juvenile arthritis, unspecified, unspecified site	Arthritis Joint Other	Nociceptive Pain
M0891	Juvenile arthritis, unspecified, shoulder	Arthritis Joint Upper Limb	Nociceptive Pain
M08911	Juvenile arthritis, unspecified, right shoulder	Arthritis Joint Upper Limb	Nociceptive Pain
M08912	Juvenile arthritis, unspecified, left shoulder	Arthritis Joint Upper Limb	Nociceptive Pain
M08919	Juvenile arthritis, unspecified, unspecified shoulder	Arthritis Joint Upper Limb	Nociceptive Pain
M0892	Juvenile arthritis, unspecified, elbow	Arthritis Joint Upper Limb	Nociceptive Pain
M08921	Juvenile arthritis, unspecified, right elbow	Arthritis Joint Upper Limb	Nociceptive Pain
M08922	Juvenile arthritis, unspecified, left elbow	Arthritis Joint Upper Limb	Nociceptive Pain
M08929	Juvenile arthritis, unspecified, unspecified elbow	Arthritis Joint Upper Limb	Nociceptive Pain
M0893	Juvenile arthritis, unspecified, wrist	Arthritis Joint Upper Limb	Nociceptive Pain
M08931	Juvenile arthritis, unspecified, right wrist	Arthritis Joint Upper Limb	Nociceptive Pain
M08932	Juvenile arthritis, unspecified, left wrist	Arthritis Joint Upper Limb	Nociceptive Pain
M08939	Juvenile arthritis, unspecified, unspecified wrist	Arthritis Joint Upper Limb	Nociceptive Pain
M0894	Juvenile arthritis, unspecified, hand	Arthritis Joint Upper Limb	Nociceptive Pain
M08941	Juvenile arthritis, unspecified, right hand	Arthritis Joint Upper Limb	Nociceptive Pain
M08942	Juvenile arthritis, unspecified, left hand	Arthritis Joint Upper Limb	Nociceptive Pain
M08949	Juvenile arthritis, unspecified, unspecified hand	Arthritis Joint Upper Limb	Nociceptive Pain
M0895	Juvenile arthritis, unspecified, hip	Arthritis Joint Spine and Hips	Nociceptive Pain
M08951	Juvenile arthritis, unspecified, right hip	Arthritis Joint Spine and Hips	Nociceptive Pain
M08952	Juvenile arthritis, unspecified, left hip	Arthritis Joint Spine and Hips	Nociceptive Pain
M08959	Juvenile arthritis, unspecified, unspecified hip	Arthritis Joint Spine and Hips	Nociceptive Pain
M0896	Juvenile arthritis, unspecified, knee	Arthritis Joint Lower Limb	Nociceptive Pain
M08961	Juvenile arthritis, unspecified, right knee	Arthritis Joint Lower Limb	Nociceptive Pain

© 2024 Peterson MD et al. *JAMA Neurology.*

M08962	Juvenile arthritis, unspecified, left knee	Arthritis Joint Lower Limb	Nociceptive Pain
M08969	Juvenile arthritis, unspecified, unspecified knee	Arthritis Joint Lower Limb	Nociceptive Pain
M0897	Juvenile arthritis, unspecified, ankle and foot	Arthritis Joint Lower Limb	Nociceptive Pain
M08971	Juvenile arthritis, unspecified, right ankle and foot	Arthritis Joint Lower Limb	Nociceptive Pain
M08972	Juvenile arthritis, unspecified, left ankle and foot	Arthritis Joint Lower Limb	Nociceptive Pain
M08979	Juvenile arthritis, unspecified, unspecified ankle and foot	Arthritis Joint Lower Limb	Nociceptive Pain
M0898	Juvenile arthritis, unspecified, vertebrae	Arthritis Joint Spine and Hips	Nociceptive Pain
M0899	Juvenile arthritis, unspecified, multiple sites	Arthritis Joint Other	Nociceptive Pain
M089A	Juvenile arthritis, unspecified, other specified site	Arthritis Joint Other	Nociceptive Pain
M11	Other crystal arthropathies	Arthritis Joint Other	Nociceptive Pain
M110	Hydroxyapatite deposition disease	Arthritis Joint Other	Nociceptive Pain
M1100	Hydroxyapatite deposition disease, unspecified site	Arthritis Joint Other	Nociceptive Pain
M1101	Hydroxyapatite deposition disease, shoulder	Arthritis Joint Upper Limb	Nociceptive Pain
M11011	Hydroxyapatite deposition disease, right shoulder	Arthritis Joint Upper Limb	Nociceptive Pain
M11012	Hydroxyapatite deposition disease, left shoulder	Arthritis Joint Upper Limb	Nociceptive Pain
M11019	Hydroxyapatite deposition disease, unspecified shoulder	Arthritis Joint Upper Limb	Nociceptive Pain
M1102	Hydroxyapatite deposition disease, elbow	Arthritis Joint Upper Limb	Nociceptive Pain
M11021	Hydroxyapatite deposition disease, right elbow	Arthritis Joint Upper Limb	Nociceptive Pain
M11022	Hydroxyapatite deposition disease, left elbow	Arthritis Joint Upper Limb	Nociceptive Pain
M11029	Hydroxyapatite deposition disease, unspecified elbow	Arthritis Joint Upper Limb	Nociceptive Pain
M1103	Hydroxyapatite deposition disease, wrist	Arthritis Joint Upper Limb	Nociceptive Pain
M11031	Hydroxyapatite deposition disease, right wrist	Arthritis Joint Upper Limb	Nociceptive Pain
M11032	Hydroxyapatite deposition disease, left wrist	Arthritis Joint Upper Limb	Nociceptive Pain
M11039	Hydroxyapatite deposition disease, unspecified wrist	Arthritis Joint Upper Limb	Nociceptive Pain
M1104	Hydroxyapatite deposition disease, hand	Arthritis Joint Upper Limb	Nociceptive Pain
M11041	Hydroxyapatite deposition disease, right hand	Arthritis Joint Upper Limb	Nociceptive Pain
M11042	Hydroxyapatite deposition disease, left hand	Arthritis Joint Upper Limb	Nociceptive Pain
M11049	Hydroxyapatite deposition disease, unspecified hand	Arthritis Joint Upper Limb	Nociceptive Pain
M1105	Hydroxyapatite deposition disease, hip	Arthritis Joint Spine and Hips	Nociceptive Pain
M11051	Hydroxyapatite deposition disease, right hip	Arthritis Joint Spine and Hips	Nociceptive Pain
M11052	Hydroxyapatite deposition disease, left hip	Arthritis Joint Spine and Hips	Nociceptive Pain
M11059	Hydroxyapatite deposition disease, unspecified hip	Arthritis Joint Spine and Hips	Nociceptive Pain
M1106	Hydroxyapatite deposition disease, knee	Arthritis Joint Lower Limb	Nociceptive Pain
M11061	Hydroxyapatite deposition disease, right knee	Arthritis Joint Lower Limb	Nociceptive Pain
M11062	Hydroxyapatite deposition disease, left knee	Arthritis Joint Lower Limb	Nociceptive Pain
M11069	Hydroxyapatite deposition disease, unspecified knee	Arthritis Joint Lower Limb	Nociceptive Pain
M1107	Hydroxyapatite deposition disease, ankle and foot	Arthritis Joint Lower Limb	Nociceptive Pain

© 2024 Peterson MD et al. *JAMA Neurology.*

M11071	Hydroxyapatite deposition disease, right ankle and foot	Arthritis Joint Lower Limb	Nociceptive Pain
M11072	Hydroxyapatite deposition disease, left ankle and foot	Arthritis Joint Lower Limb	Nociceptive Pain
M11079	Hydroxyapatite deposition disease, unsp ankle and foot	Arthritis Joint Lower Limb	Nociceptive Pain
M1108	Hydroxyapatite deposition disease, vertebrae	Arthritis Joint Spine and Hips	Nociceptive Pain
M1109	Hydroxyapatite deposition disease, multiple sites	Arthritis Joint Other	Nociceptive Pain
M111	Familial chondrocalcinosis	Arthritis Joint Other	Nociceptive Pain
M1110	Familial chondrocalcinosis, unspecified site	Arthritis Joint Other	Nociceptive Pain
M1111	Familial chondrocalcinosis, shoulder	Arthritis Joint Upper Limb	Nociceptive Pain
M11111	Familial chondrocalcinosis, right shoulder	Arthritis Joint Upper Limb	Nociceptive Pain
M11112	Familial chondrocalcinosis, left shoulder	Arthritis Joint Upper Limb	Nociceptive Pain
M11119	Familial chondrocalcinosis, unspecified shoulder	Arthritis Joint Upper Limb	Nociceptive Pain
M1112	Familial chondrocalcinosis, elbow	Arthritis Joint Upper Limb	Nociceptive Pain
M11121	Familial chondrocalcinosis, right elbow	Arthritis Joint Upper Limb	Nociceptive Pain
M11122	Familial chondrocalcinosis, left elbow	Arthritis Joint Upper Limb	Nociceptive Pain
M11129	Familial chondrocalcinosis, unspecified elbow	Arthritis Joint Upper Limb	Nociceptive Pain
M1113	Familial chondrocalcinosis, wrist	Arthritis Joint Upper Limb	Nociceptive Pain
M11131	Familial chondrocalcinosis, right wrist	Arthritis Joint Upper Limb	Nociceptive Pain
M11132	Familial chondrocalcinosis, left wrist	Arthritis Joint Upper Limb	Nociceptive Pain
M11139	Familial chondrocalcinosis, unspecified wrist	Arthritis Joint Upper Limb	Nociceptive Pain
M1114	Familial chondrocalcinosis, hand	Arthritis Joint Upper Limb	Nociceptive Pain
M11141	Familial chondrocalcinosis, right hand	Arthritis Joint Upper Limb	Nociceptive Pain
M11142	Familial chondrocalcinosis, left hand	Arthritis Joint Upper Limb	Nociceptive Pain
M11149	Familial chondrocalcinosis, unspecified hand	Arthritis Joint Upper Limb	Nociceptive Pain
M1115	Familial chondrocalcinosis, hip	Arthritis Joint Spine and Hips	Nociceptive Pain
M11151	Familial chondrocalcinosis, right hip	Arthritis Joint Spine and Hips	Nociceptive Pain
M11152	Familial chondrocalcinosis, left hip	Arthritis Joint Spine and Hips	Nociceptive Pain
M11159	Familial chondrocalcinosis, unspecified hip	Arthritis Joint Spine and Hips	Nociceptive Pain
M1116	Familial chondrocalcinosis, knee	Arthritis Joint Lower Limb	Nociceptive Pain
M11161	Familial chondrocalcinosis, right knee	Arthritis Joint Lower Limb	Nociceptive Pain
M11162	Familial chondrocalcinosis, left knee	Arthritis Joint Lower Limb	Nociceptive Pain
M11169	Familial chondrocalcinosis, unspecified knee	Arthritis Joint Lower Limb	Nociceptive Pain
M1117	Familial chondrocalcinosis, ankle and foot	Arthritis Joint Lower Limb	Nociceptive Pain
M11171	Familial chondrocalcinosis, right ankle and foot	Arthritis Joint Lower Limb	Nociceptive Pain
M11172	Familial chondrocalcinosis, left ankle and foot	Arthritis Joint Lower Limb	Nociceptive Pain
M11179	Familial chondrocalcinosis, unspecified ankle and foot	Arthritis Joint Lower Limb	Nociceptive Pain
M1118	Familial chondrocalcinosis, vertebrae	Arthritis Joint Spine and Hips	Nociceptive Pain
M1119	Familial chondrocalcinosis, multiple sites	Arthritis Joint Other	Nociceptive Pain

© 2024 Peterson MD et al. *JAMA Neurology.*

M112	Other chondrocalcinosis	Arthritis Joint Other	Nociceptive Pain
M1120	Other chondrocalcinosis, unspecified site	Arthritis Joint Other	Nociceptive Pain
M1121	Other chondrocalcinosis, shoulder	Arthritis Joint Upper Limb	Nociceptive Pain
M11211	Other chondrocalcinosis, right shoulder	Arthritis Joint Upper Limb	Nociceptive Pain
M11212	Other chondrocalcinosis, left shoulder	Arthritis Joint Upper Limb	Nociceptive Pain
M11219	Other chondrocalcinosis, unspecified shoulder	Arthritis Joint Upper Limb	Nociceptive Pain
M1122	Other chondrocalcinosis, elbow	Arthritis Joint Upper Limb	Nociceptive Pain
M11221	Other chondrocalcinosis, right elbow	Arthritis Joint Upper Limb	Nociceptive Pain
M11222	Other chondrocalcinosis, left elbow	Arthritis Joint Upper Limb	Nociceptive Pain
M11229	Other chondrocalcinosis, unspecified elbow	Arthritis Joint Upper Limb	Nociceptive Pain
M1123	Other chondrocalcinosis, wrist	Arthritis Joint Upper Limb	Nociceptive Pain
M11231	Other chondrocalcinosis, right wrist	Arthritis Joint Upper Limb	Nociceptive Pain
M11232	Other chondrocalcinosis, left wrist	Arthritis Joint Upper Limb	Nociceptive Pain
M11239	Other chondrocalcinosis, unspecified wrist	Arthritis Joint Upper Limb	Nociceptive Pain
M1124	Other chondrocalcinosis, hand	Arthritis Joint Upper Limb	Nociceptive Pain
M11241	Other chondrocalcinosis, right hand	Arthritis Joint Upper Limb	Nociceptive Pain
M11242	Other chondrocalcinosis, left hand	Arthritis Joint Upper Limb	Nociceptive Pain
M11249	Other chondrocalcinosis, unspecified hand	Arthritis Joint Upper Limb	Nociceptive Pain
M1125	Other chondrocalcinosis, hip	Arthritis Joint Spine and Hips	Nociceptive Pain
M11251	Other chondrocalcinosis, right hip	Arthritis Joint Spine and Hips	Nociceptive Pain
M11252	Other chondrocalcinosis, left hip	Arthritis Joint Spine and Hips	Nociceptive Pain
M11259	Other chondrocalcinosis, unspecified hip	Arthritis Joint Spine and Hips	Nociceptive Pain
M1126	Other chondrocalcinosis, knee	Arthritis Joint Lower Limb	Nociceptive Pain
M11261	Other chondrocalcinosis, right knee	Arthritis Joint Lower Limb	Nociceptive Pain
M11262	Other chondrocalcinosis, left knee	Arthritis Joint Lower Limb	Nociceptive Pain
M11269	Other chondrocalcinosis, unspecified knee	Arthritis Joint Lower Limb	Nociceptive Pain
M1127	Other chondrocalcinosis, ankle and foot	Arthritis Joint Lower Limb	Nociceptive Pain
M11271	Other chondrocalcinosis, right ankle and foot	Arthritis Joint Lower Limb	Nociceptive Pain
M11272	Other chondrocalcinosis, left ankle and foot	Arthritis Joint Lower Limb	Nociceptive Pain
M11279	Other chondrocalcinosis, unspecified ankle and foot	Arthritis Joint Lower Limb	Nociceptive Pain
M1128	Other chondrocalcinosis, vertebrae	Arthritis Joint Spine and Hips	Nociceptive Pain
M1129	Other chondrocalcinosis, multiple sites	Arthritis Joint Other	Nociceptive Pain
M118	Other specified crystal arthropathies	Arthritis Joint Other	Nociceptive Pain
M1180	Other specified crystal arthropathies, unspecified site	Arthritis Joint Other	Nociceptive Pain
M1181	Other specified crystal arthropathies, shoulder	Arthritis Joint Upper Limb	Nociceptive Pain
M11811	Other specified crystal arthropathies, right shoulder	Arthritis Joint Upper Limb	Nociceptive Pain
M11812	Other specified crystal arthropathies, left shoulder	Arthritis Joint Upper Limb	Nociceptive Pain

© 2024 Peterson MD et al. *JAMA Neurology.*

M11819	Other specified crystal arthropathies, unspecified shoulder	Arthritis Joint Upper Limb	Nociceptive Pain
M1182	Other specified crystal arthropathies, elbow	Arthritis Joint Upper Limb	Nociceptive Pain
M11821	Other specified crystal arthropathies, right elbow	Arthritis Joint Upper Limb	Nociceptive Pain
M11822	Other specified crystal arthropathies, left elbow	Arthritis Joint Upper Limb	Nociceptive Pain
M11829	Other specified crystal arthropathies, unspecified elbow	Arthritis Joint Upper Limb	Nociceptive Pain
M1183	Other specified crystal arthropathies, wrist	Arthritis Joint Upper Limb	Nociceptive Pain
M11831	Other specified crystal arthropathies, right wrist	Arthritis Joint Upper Limb	Nociceptive Pain
M11832	Other specified crystal arthropathies, left wrist	Arthritis Joint Upper Limb	Nociceptive Pain
M11839	Other specified crystal arthropathies, unspecified wrist	Arthritis Joint Upper Limb	Nociceptive Pain
M1184	Other specified crystal arthropathies, hand	Arthritis Joint Upper Limb	Nociceptive Pain
M11841	Other specified crystal arthropathies, right hand	Arthritis Joint Upper Limb	Nociceptive Pain
M11842	Other specified crystal arthropathies, left hand	Arthritis Joint Upper Limb	Nociceptive Pain
M11849	Other specified crystal arthropathies, unspecified hand	Arthritis Joint Upper Limb	Nociceptive Pain
M1185	Other specified crystal arthropathies, hip	Arthritis Joint Spine and Hips	Nociceptive Pain
M11851	Other specified crystal arthropathies, right hip	Arthritis Joint Spine and Hips	Nociceptive Pain
M11852	Other specified crystal arthropathies, left hip	Arthritis Joint Spine and Hips	Nociceptive Pain
M11859	Other specified crystal arthropathies, unspecified hip	Arthritis Joint Spine and Hips	Nociceptive Pain
M1186	Other specified crystal arthropathies, knee	Arthritis Joint Lower Limb	Nociceptive Pain
M11861	Other specified crystal arthropathies, right knee	Arthritis Joint Lower Limb	Nociceptive Pain
M11862	Other specified crystal arthropathies, left knee	Arthritis Joint Lower Limb	Nociceptive Pain
M11869	Other specified crystal arthropathies, unspecified knee	Arthritis Joint Lower Limb	Nociceptive Pain
M1187	Other specified crystal arthropathies, ankle and foot	Arthritis Joint Lower Limb	Nociceptive Pain
M11871	Other specified crystal arthropathies, right ankle and foot	Arthritis Joint Lower Limb	Nociceptive Pain
M11872	Other specified crystal arthropathies, left ankle and foot	Arthritis Joint Lower Limb	Nociceptive Pain
M11879	Oth crystal arthropathies, unspecified ankle and foot	Arthritis Joint Lower Limb	Nociceptive Pain
M1188	Other specified crystal arthropathies, vertebrae	Arthritis Joint Spine and Hips	Nociceptive Pain
M1189	Other specified crystal arthropathies, multiple sites	Arthritis Joint Other	Nociceptive Pain
M119	Crystal arthropathy, unspecified	Arthritis Joint Other	Nociceptive Pain
M12	Other and unspecified arthropathy	Arthritis Joint Other	Nociceptive Pain
M120	Chronic postrheumatic arthropathy [Jaccoud]	Arthritis Joint Other	Nociceptive Pain
M1200	Chronic postrheumatic arthropathy, unspecified site	Arthritis Joint Other	Nociceptive Pain
M1201	Chronic postrheumatic arthropathy [Jaccoud], shoulder	Arthritis Joint Upper Limb	Nociceptive Pain
M12011	Chronic postrheumatic arthropathy [Jaccoud], right shoulder	Arthritis Joint Upper Limb	Nociceptive Pain
M12012	Chronic postrheumatic arthropathy [Jaccoud], left shoulder	Arthritis Joint Upper Limb	Nociceptive Pain
M12019	Chronic postrheumatic arthropathy, unspecified shoulder	Arthritis Joint Upper Limb	Nociceptive Pain
M1202	Chronic postrheumatic arthropathy [Jaccoud], elbow	Arthritis Joint Upper Limb	Nociceptive Pain
M12021	Chronic postrheumatic arthropathy [Jaccoud], right elbow	Arthritis Joint Upper Limb	Nociceptive Pain

© 2024 Peterson MD et al. *JAMA Neurology.*

M12022	Chronic postrheumatic arthropathy [Jaccoud], left elbow	Arthritis Joint Upper Limb	Nociceptive Pain
M12029	Chronic postrheumatic arthropathy, unspecified elbow	Arthritis Joint Upper Limb	Nociceptive Pain
M1203	Chronic postrheumatic arthropathy [Jaccoud], wrist	Arthritis Joint Upper Limb	Nociceptive Pain
M12031	Chronic postrheumatic arthropathy [Jaccoud], right wrist	Arthritis Joint Upper Limb	Nociceptive Pain
M12032	Chronic postrheumatic arthropathy [Jaccoud], left wrist	Arthritis Joint Upper Limb	Nociceptive Pain
M12039	Chronic postrheumatic arthropathy, unspecified wrist	Arthritis Joint Upper Limb	Nociceptive Pain
M1204	Chronic postrheumatic arthropathy [Jaccoud], hand	Arthritis Joint Upper Limb	Nociceptive Pain
M12041	Chronic postrheumatic arthropathy [Jaccoud], right hand	Arthritis Joint Upper Limb	Nociceptive Pain
M12042	Chronic postrheumatic arthropathy [Jaccoud], left hand	Arthritis Joint Upper Limb	Nociceptive Pain
M12049	Chronic postrheumatic arthropathy, unspecified hand	Arthritis Joint Upper Limb	Nociceptive Pain
M1205	Chronic postrheumatic arthropathy [Jaccoud], hip	Arthritis Joint Spine and Hips	Nociceptive Pain
M12051	Chronic postrheumatic arthropathy [Jaccoud], right hip	Arthritis Joint Spine and Hips	Nociceptive Pain
M12052	Chronic postrheumatic arthropathy [Jaccoud], left hip	Arthritis Joint Spine and Hips	Nociceptive Pain
M12059	Chronic postrheumatic arthropathy [Jaccoud], unspecified hip	Arthritis Joint Spine and Hips	Nociceptive Pain
M1206	Chronic postrheumatic arthropathy [Jaccoud], knee	Arthritis Joint Lower Limb	Nociceptive Pain
M12061	Chronic postrheumatic arthropathy [Jaccoud], right knee	Arthritis Joint Lower Limb	Nociceptive Pain
M12062	Chronic postrheumatic arthropathy [Jaccoud], left knee	Arthritis Joint Lower Limb	Nociceptive Pain
M12069	Chronic postrheumatic arthropathy, unspecified knee	Arthritis Joint Lower Limb	Nociceptive Pain
M1207	Chronic postrheumatic arthropathy [Jaccoud], ankle and foot	Arthritis Joint Lower Limb	Nociceptive Pain
M12071	Chronic postrheumatic arthropathy, right ankle and foot	Arthritis Joint Lower Limb	Nociceptive Pain
M12072	Chronic postrheumatic arthropathy, left ankle and foot	Arthritis Joint Lower Limb	Nociceptive Pain
M12079	Chronic postrheumatic arthropathy, unsp ankle and foot	Arthritis Joint Lower Limb	Nociceptive Pain
M1208	Chronic postrheumatic arthropathy, other specified site	Arthritis Joint Other	Nociceptive Pain
M1209	Chronic postrheumatic arthropathy [Jaccoud], multiple sites	Arthritis Joint Other	Nociceptive Pain
M121	Kaschin-Beck disease	Arthritis Joint Other	Nociceptive Pain
M1210	Kaschin-Beck disease, unspecified site	Arthritis Joint Other	Nociceptive Pain
M1211	Kaschin-Beck disease, shoulder	Arthritis Joint Upper Limb	Nociceptive Pain
M12111	Kaschin-Beck disease, right shoulder	Arthritis Joint Upper Limb	Nociceptive Pain
M12112	Kaschin-Beck disease, left shoulder	Arthritis Joint Upper Limb	Nociceptive Pain
M12119	Kaschin-Beck disease, unspecified shoulder	Arthritis Joint Upper Limb	Nociceptive Pain
M1212	Kaschin-Beck disease, elbow	Arthritis Joint Upper Limb	Nociceptive Pain
M12121	Kaschin-Beck disease, right elbow	Arthritis Joint Upper Limb	Nociceptive Pain
M12122	Kaschin-Beck disease, left elbow	Arthritis Joint Upper Limb	Nociceptive Pain
M12129	Kaschin-Beck disease, unspecified elbow	Arthritis Joint Upper Limb	Nociceptive Pain
M1213	Kaschin-Beck disease, wrist	Arthritis Joint Upper Limb	Nociceptive Pain
M12131	Kaschin-Beck disease, right wrist	Arthritis Joint Upper Limb	Nociceptive Pain
M12132	Kaschin-Beck disease, left wrist	Arthritis Joint Upper Limb	Nociceptive Pain

© 2024 Peterson MD et al. *JAMA Neurology.*

M12139	Kaschin-Beck disease, unspecified wrist	Arthritis Joint Upper Limb	Nociceptive Pain
M1214	Kaschin-Beck disease, hand	Arthritis Joint Upper Limb	Nociceptive Pain
M12141	Kaschin-Beck disease, right hand	Arthritis Joint Upper Limb	Nociceptive Pain
M12142	Kaschin-Beck disease, left hand	Arthritis Joint Upper Limb	Nociceptive Pain
M12149	Kaschin-Beck disease, unspecified hand	Arthritis Joint Upper Limb	Nociceptive Pain
M1215	Kaschin-Beck disease, hip	Arthritis Joint Spine and Hips	Nociceptive Pain
M12151	Kaschin-Beck disease, right hip	Arthritis Joint Spine and Hips	Nociceptive Pain
M12152	Kaschin-Beck disease, left hip	Arthritis Joint Spine and Hips	Nociceptive Pain
M12159	Kaschin-Beck disease, unspecified hip	Arthritis Joint Spine and Hips	Nociceptive Pain
M1216	Kaschin-Beck disease, knee	Arthritis Joint Lower Limb	Nociceptive Pain
M12161	Kaschin-Beck disease, right knee	Arthritis Joint Lower Limb	Nociceptive Pain
M12162	Kaschin-Beck disease, left knee	Arthritis Joint Lower Limb	Nociceptive Pain
M12169	Kaschin-Beck disease, unspecified knee	Arthritis Joint Lower Limb	Nociceptive Pain
M1217	Kaschin-Beck disease, ankle and foot	Arthritis Joint Lower Limb	Nociceptive Pain
M12171	Kaschin-Beck disease, right ankle and foot	Arthritis Joint Lower Limb	Nociceptive Pain
M12172	Kaschin-Beck disease, left ankle and foot	Arthritis Joint Lower Limb	Nociceptive Pain
M12179	Kaschin-Beck disease, unspecified ankle and foot	Arthritis Joint Lower Limb	Nociceptive Pain
M1218	Kaschin-Beck disease, vertebrae	Arthritis Joint Spine and Hips	Nociceptive Pain
M1219	Kaschin-Beck disease, multiple sites	Arthritis Joint Other	Nociceptive Pain
M122	Villonodular synovitis (pigmented)	Arthritis Joint Other	Nociceptive Pain
M1220	Villonodular synovitis (pigmented), unspecified site	Arthritis Joint Other	Nociceptive Pain
M1221	Villonodular synovitis (pigmented), shoulder	Arthritis Joint Upper Limb	Nociceptive Pain
M12211	Villonodular synovitis (pigmented), right shoulder	Arthritis Joint Upper Limb	Nociceptive Pain
M12212	Villonodular synovitis (pigmented), left shoulder	Arthritis Joint Upper Limb	Nociceptive Pain
M12219	Villonodular synovitis (pigmented), unspecified shoulder	Arthritis Joint Upper Limb	Nociceptive Pain
M1222	Villonodular synovitis (pigmented), elbow	Arthritis Joint Upper Limb	Nociceptive Pain
M12221	Villonodular synovitis (pigmented), right elbow	Arthritis Joint Upper Limb	Nociceptive Pain
M12222	Villonodular synovitis (pigmented), left elbow	Arthritis Joint Upper Limb	Nociceptive Pain
M12229	Villonodular synovitis (pigmented), unspecified elbow	Arthritis Joint Upper Limb	Nociceptive Pain
M1223	Villonodular synovitis (pigmented), wrist	Arthritis Joint Upper Limb	Nociceptive Pain
M12231	Villonodular synovitis (pigmented), right wrist	Arthritis Joint Upper Limb	Nociceptive Pain
M12232	Villonodular synovitis (pigmented), left wrist	Arthritis Joint Upper Limb	Nociceptive Pain
M12239	Villonodular synovitis (pigmented), unspecified wrist	Arthritis Joint Upper Limb	Nociceptive Pain
M1224	Villonodular synovitis (pigmented), hand	Arthritis Joint Upper Limb	Nociceptive Pain
M12241	Villonodular synovitis (pigmented), right hand	Arthritis Joint Upper Limb	Nociceptive Pain
M12242	Villonodular synovitis (pigmented), left hand	Arthritis Joint Upper Limb	Nociceptive Pain
M12249	Villonodular synovitis (pigmented), unspecified hand	Arthritis Joint Upper Limb	Nociceptive Pain

© 2024 Peterson MD et al. *JAMA Neurology.*

M1225	Villonodular synovitis (pigmented), hip	Arthritis Joint Spine and Hips	Nociceptive Pain
M12251	Villonodular synovitis (pigmented), right hip	Arthritis Joint Spine and Hips	Nociceptive Pain
M12252	Villonodular synovitis (pigmented), left hip	Arthritis Joint Spine and Hips	Nociceptive Pain
M12259	Villonodular synovitis (pigmented), unspecified hip	Arthritis Joint Spine and Hips	Nociceptive Pain
M1226	Villonodular synovitis (pigmented), knee	Arthritis Joint Lower Limb	Nociceptive Pain
M12261	Villonodular synovitis (pigmented), right knee	Arthritis Joint Lower Limb	Nociceptive Pain
M12262	Villonodular synovitis (pigmented), left knee	Arthritis Joint Lower Limb	Nociceptive Pain
M12269	Villonodular synovitis (pigmented), unspecified knee	Arthritis Joint Lower Limb	Nociceptive Pain
M1227	Villonodular synovitis (pigmented), ankle and foot	Arthritis Joint Lower Limb	Nociceptive Pain
M12271	Villonodular synovitis (pigmented), right ankle and foot	Arthritis Joint Lower Limb	Nociceptive Pain
M12272	Villonodular synovitis (pigmented), left ankle and foot	Arthritis Joint Lower Limb	Nociceptive Pain
M12279	Villonodular synovitis (pigmented), unsp ankle and foot	Arthritis Joint Lower Limb	Nociceptive Pain
M1228	Villonodular synovitis (pigmented), other specified site	Arthritis Joint Other	Nociceptive Pain
M1229	Villonodular synovitis (pigmented), multiple sites	Arthritis Joint Other	Nociceptive Pain
M123	Palindromic rheumatism	Arthritis Joint Other	Nociceptive Pain
M1230	Palindromic rheumatism, unspecified site	Arthritis Joint Other	Nociceptive Pain
M1231	Palindromic rheumatism, shoulder	Arthritis Joint Upper Limb	Nociceptive Pain
M12311	Palindromic rheumatism, right shoulder	Arthritis Joint Upper Limb	Nociceptive Pain
M12312	Palindromic rheumatism, left shoulder	Arthritis Joint Upper Limb	Nociceptive Pain
M12319	Palindromic rheumatism, unspecified shoulder	Arthritis Joint Upper Limb	Nociceptive Pain
M1232	Palindromic rheumatism, elbow	Arthritis Joint Upper Limb	Nociceptive Pain
M12321	Palindromic rheumatism, right elbow	Arthritis Joint Upper Limb	Nociceptive Pain
M12322	Palindromic rheumatism, left elbow	Arthritis Joint Upper Limb	Nociceptive Pain
M12329	Palindromic rheumatism, unspecified elbow	Arthritis Joint Upper Limb	Nociceptive Pain
M1233	Palindromic rheumatism, wrist	Arthritis Joint Upper Limb	Nociceptive Pain
M12331	Palindromic rheumatism, right wrist	Arthritis Joint Upper Limb	Nociceptive Pain
M12332	Palindromic rheumatism, left wrist	Arthritis Joint Upper Limb	Nociceptive Pain
M12339	Palindromic rheumatism, unspecified wrist	Arthritis Joint Upper Limb	Nociceptive Pain
M1234	Palindromic rheumatism, hand	Arthritis Joint Upper Limb	Nociceptive Pain
M12341	Palindromic rheumatism, right hand	Arthritis Joint Upper Limb	Nociceptive Pain
M12342	Palindromic rheumatism, left hand	Arthritis Joint Upper Limb	Nociceptive Pain
M12349	Palindromic rheumatism, unspecified hand	Arthritis Joint Upper Limb	Nociceptive Pain
M1235	Palindromic rheumatism, hip	Arthritis Joint Spine and Hips	Nociceptive Pain
M12351	Palindromic rheumatism, right hip	Arthritis Joint Spine and Hips	Nociceptive Pain
M12352	Palindromic rheumatism, left hip	Arthritis Joint Spine and Hips	Nociceptive Pain
M12359	Palindromic rheumatism, unspecified hip	Arthritis Joint Spine and Hips	Nociceptive Pain
M1236	Palindromic rheumatism, knee	Arthritis Joint Lower Limb	Nociceptive Pain

© 2024 Peterson MD et al. *JAMA Neurology.*

M12361	Palindromic rheumatism, right knee	Arthritis Joint Lower Limb	Nociceptive Pain
M12362	Palindromic rheumatism, left knee	Arthritis Joint Lower Limb	Nociceptive Pain
M12369	Palindromic rheumatism, unspecified knee	Arthritis Joint Lower Limb	Nociceptive Pain
M1237	Palindromic rheumatism, ankle and foot	Arthritis Joint Lower Limb	Nociceptive Pain
M12371	Palindromic rheumatism, right ankle and foot	Arthritis Joint Lower Limb	Nociceptive Pain
M12372	Palindromic rheumatism, left ankle and foot	Arthritis Joint Lower Limb	Nociceptive Pain
M12379	Palindromic rheumatism, unspecified ankle and foot	Arthritis Joint Lower Limb	Nociceptive Pain
M1238	Palindromic rheumatism, other specified site	Arthritis Joint Other	Nociceptive Pain
M1239	Palindromic rheumatism, multiple sites	Arthritis Joint Other	Nociceptive Pain
M124	Intermittent hydrarthrosis	Arthritis Joint Other	Nociceptive Pain
M1240	Intermittent hydrarthrosis, unspecified site	Arthritis Joint Other	Nociceptive Pain
M1241	Intermittent hydrarthrosis, shoulder	Arthritis Joint Upper Limb	Nociceptive Pain
M12411	Intermittent hydrarthrosis, right shoulder	Arthritis Joint Upper Limb	Nociceptive Pain
M12412	Intermittent hydrarthrosis, left shoulder	Arthritis Joint Upper Limb	Nociceptive Pain
M12419	Intermittent hydrarthrosis, unspecified shoulder	Arthritis Joint Upper Limb	Nociceptive Pain
M1242	Intermittent hydrarthrosis, elbow	Arthritis Joint Upper Limb	Nociceptive Pain
M12421	Intermittent hydrarthrosis, right elbow	Arthritis Joint Upper Limb	Nociceptive Pain
M12422	Intermittent hydrarthrosis, left elbow	Arthritis Joint Upper Limb	Nociceptive Pain
M12429	Intermittent hydrarthrosis, unspecified elbow	Arthritis Joint Upper Limb	Nociceptive Pain
M1243	Intermittent hydrarthrosis, wrist	Arthritis Joint Upper Limb	Nociceptive Pain
M12431	Intermittent hydrarthrosis, right wrist	Arthritis Joint Upper Limb	Nociceptive Pain
M12432	Intermittent hydrarthrosis, left wrist	Arthritis Joint Upper Limb	Nociceptive Pain
M12439	Intermittent hydrarthrosis, unspecified wrist	Arthritis Joint Upper Limb	Nociceptive Pain
M1244	Intermittent hydrarthrosis, hand	Arthritis Joint Upper Limb	Nociceptive Pain
M12441	Intermittent hydrarthrosis, right hand	Arthritis Joint Upper Limb	Nociceptive Pain
M12442	Intermittent hydrarthrosis, left hand	Arthritis Joint Upper Limb	Nociceptive Pain
M12449	Intermittent hydrarthrosis, unspecified hand	Arthritis Joint Upper Limb	Nociceptive Pain
M1245	Intermittent hydrarthrosis, hip	Arthritis Joint Spine and Hips	Nociceptive Pain
M12451	Intermittent hydrarthrosis, right hip	Arthritis Joint Spine and Hips	Nociceptive Pain
M12452	Intermittent hydrarthrosis, left hip	Arthritis Joint Spine and Hips	Nociceptive Pain
M12459	Intermittent hydrarthrosis, unspecified hip	Arthritis Joint Spine and Hips	Nociceptive Pain
M1246	Intermittent hydrarthrosis, knee	Arthritis Joint Lower Limb	Nociceptive Pain
M12461	Intermittent hydrarthrosis, right knee	Arthritis Joint Lower Limb	Nociceptive Pain
M12462	Intermittent hydrarthrosis, left knee	Arthritis Joint Lower Limb	Nociceptive Pain
M12469	Intermittent hydrarthrosis, unspecified knee	Arthritis Joint Lower Limb	Nociceptive Pain
M1247	Intermittent hydrarthrosis, ankle and foot	Arthritis Joint Lower Limb	Nociceptive Pain
M12471	Intermittent hydrarthrosis, right ankle and foot	Arthritis Joint Lower Limb	Nociceptive Pain

© 2024 Peterson MD et al. *JAMA Neurology.*

M12472	Intermittent hydrarthrosis, left ankle and foot	Arthritis Joint Lower Limb	Nociceptive Pain
M12479	Intermittent hydrarthrosis, unspecified ankle and foot	Arthritis Joint Lower Limb	Nociceptive Pain
M1248	Intermittent hydrarthrosis, other site	Arthritis Joint Other	Nociceptive Pain
M1249	Intermittent hydrarthrosis, multiple sites	Arthritis Joint Other	Nociceptive Pain
M125	Traumatic arthropathy	Arthritis Joint Other	Nociceptive Pain
M1250	Traumatic arthropathy, unspecified site	Arthritis Joint Other	Nociceptive Pain
M1251	Traumatic arthropathy, shoulder	Arthritis Joint Upper Limb	Nociceptive Pain
M12511	Traumatic arthropathy, right shoulder	Arthritis Joint Upper Limb	Nociceptive Pain
M12512	Traumatic arthropathy, left shoulder	Arthritis Joint Upper Limb	Nociceptive Pain
M12519	Traumatic arthropathy, unspecified shoulder	Arthritis Joint Upper Limb	Nociceptive Pain
M1252	Traumatic arthropathy, elbow	Arthritis Joint Upper Limb	Nociceptive Pain
M12521	Traumatic arthropathy, right elbow	Arthritis Joint Upper Limb	Nociceptive Pain
M12522	Traumatic arthropathy, left elbow	Arthritis Joint Upper Limb	Nociceptive Pain
M12529	Traumatic arthropathy, unspecified elbow	Arthritis Joint Upper Limb	Nociceptive Pain
M1253	Traumatic arthropathy, wrist	Arthritis Joint Upper Limb	Nociceptive Pain
M12531	Traumatic arthropathy, right wrist	Arthritis Joint Upper Limb	Nociceptive Pain
M12532	Traumatic arthropathy, left wrist	Arthritis Joint Upper Limb	Nociceptive Pain
M12539	Traumatic arthropathy, unspecified wrist	Arthritis Joint Upper Limb	Nociceptive Pain
M1254	Traumatic arthropathy, hand	Arthritis Joint Upper Limb	Nociceptive Pain
M12541	Traumatic arthropathy, right hand	Arthritis Joint Upper Limb	Nociceptive Pain
M12542	Traumatic arthropathy, left hand	Arthritis Joint Upper Limb	Nociceptive Pain
M12549	Traumatic arthropathy, unspecified hand	Arthritis Joint Upper Limb	Nociceptive Pain
M1255	Traumatic arthropathy, hip	Arthritis Joint Spine and Hips	Nociceptive Pain
M12551	Traumatic arthropathy, right hip	Arthritis Joint Spine and Hips	Nociceptive Pain
M12552	Traumatic arthropathy, left hip	Arthritis Joint Spine and Hips	Nociceptive Pain
M12559	Traumatic arthropathy, unspecified hip	Arthritis Joint Spine and Hips	Nociceptive Pain
M1256	Traumatic arthropathy, knee	Arthritis Joint Lower Limb	Nociceptive Pain
M12561	Traumatic arthropathy, right knee	Arthritis Joint Lower Limb	Nociceptive Pain
M12562	Traumatic arthropathy, left knee	Arthritis Joint Lower Limb	Nociceptive Pain
M12569	Traumatic arthropathy, unspecified knee	Arthritis Joint Lower Limb	Nociceptive Pain
M1257	Traumatic arthropathy, ankle and foot	Arthritis Joint Lower Limb	Nociceptive Pain
M12571	Traumatic arthropathy, right ankle and foot	Arthritis Joint Lower Limb	Nociceptive Pain
M12572	Traumatic arthropathy, left ankle and foot	Arthritis Joint Lower Limb	Nociceptive Pain
M12579	Traumatic arthropathy, unspecified ankle and foot	Arthritis Joint Lower Limb	Nociceptive Pain
M1258	Traumatic arthropathy, other specified site	Arthritis Joint Other	Nociceptive Pain
M1259	Traumatic arthropathy, multiple sites	Arthritis Joint Other	Nociceptive Pain
M128	Other specific arthropathies, not elsewhere classified	Arthritis Joint Other	Nociceptive Pain

© 2024 Peterson MD et al. *JAMA Neurology.*

M1280	Oth specific arthropathies, NEC, unsp site	Arthritis Joint Other	Nociceptive Pain
M1281	Oth specific arthropathies, NEC, shoulder	Arthritis Joint Upper Limb	Nociceptive Pain
M12811	Oth specific arthropathies, NEC, right shoulder	Arthritis Joint Upper Limb	Nociceptive Pain
M12812	Oth specific arthropathies, NEC, left shoulder	Arthritis Joint Upper Limb	Nociceptive Pain
M12819	Oth specific arthropathies, NEC, unsp shoulder	Arthritis Joint Upper Limb	Nociceptive Pain
M1282	Oth specific arthropathies, not elsewhere classified, elbow	Arthritis Joint Upper Limb	Nociceptive Pain
M12821	Oth specific arthropathies, NEC, right elbow	Arthritis Joint Upper Limb	Nociceptive Pain
M12822	Oth specific arthropathies, NEC, left elbow	Arthritis Joint Upper Limb	Nociceptive Pain
M12829	Oth specific arthropathies, NEC, unsp elbow	Arthritis Joint Upper Limb	Nociceptive Pain
M1283	Oth specific arthropathies, not elsewhere classified, wrist	Arthritis Joint Upper Limb	Nociceptive Pain
M12831	Oth specific arthropathies, NEC, right wrist	Arthritis Joint Upper Limb	Nociceptive Pain
M12832	Oth specific arthropathies, NEC, left wrist	Arthritis Joint Upper Limb	Nociceptive Pain
M12839	Oth specific arthropathies, NEC, unsp wrist	Arthritis Joint Upper Limb	Nociceptive Pain
M1284	Other specific arthropathies, not elsewhere classified, hand	Arthritis Joint Upper Limb	Nociceptive Pain
M12841	Oth specific arthropathies, NEC, right hand	Arthritis Joint Upper Limb	Nociceptive Pain
M12842	Oth specific arthropathies, NEC, left hand	Arthritis Joint Upper Limb	Nociceptive Pain
M12849	Oth specific arthropathies, NEC, unsp hand	Arthritis Joint Upper Limb	Nociceptive Pain
M1285	Other specific arthropathies, not elsewhere classified, hip	Arthritis Joint Spine and Hips	Nociceptive Pain
M12851	Oth specific arthropathies, NEC, right hip	Arthritis Joint Spine and Hips	Nociceptive Pain
M12852	Oth specific arthropathies, NEC, left hip	Arthritis Joint Spine and Hips	Nociceptive Pain
M12859	Oth specific arthropathies, NEC, unsp hip	Arthritis Joint Spine and Hips	Nociceptive Pain
M1286	Other specific arthropathies, not elsewhere classified, knee	Arthritis Joint Lower Limb	Nociceptive Pain
M12861	Oth specific arthropathies, NEC, right knee	Arthritis Joint Lower Limb	Nociceptive Pain
M12862	Oth specific arthropathies, NEC, left knee	Arthritis Joint Lower Limb	Nociceptive Pain
M12869	Oth specific arthropathies, NEC, unsp knee	Arthritis Joint Lower Limb	Nociceptive Pain
M1287	Oth specific arthropathies, NEC, ankle and foot	Arthritis Joint Lower Limb	Nociceptive Pain
M12871	Oth specific arthropathies, NEC, right ankle and foot	Arthritis Joint Lower Limb	Nociceptive Pain
M12872	Oth specific arthropathies, NEC, left ankle and foot	Arthritis Joint Lower Limb	Nociceptive Pain
M12879	Oth specific arthropathies, NEC, unsp ankle and foot	Arthritis Joint Lower Limb	Nociceptive Pain
M1288	Oth specific arthropathies, NEC, oth site	Arthritis Joint Other	Nociceptive Pain
M1289	Oth specific arthropathies, NEC, multiple sites	Arthritis Joint Other	Nociceptive Pain
M129	Arthropathy, unspecified	Arthritis Joint Other	Nociceptive Pain
M13	Other arthritis	Arthritis Joint Other	Nociceptive Pain
M130	Polyarthritis, unspecified	Arthritis Joint Other	Nociceptive Pain
M131	Monoarthritis, not elsewhere classified	Arthritis Joint Other	Nociceptive Pain
M1310	Monoarthritis, not elsewhere classified, unspecified site	Arthritis Joint Other	Nociceptive Pain
M1311	Monoarthritis, not elsewhere classified, shoulder	Arthritis Joint Upper Limb	Nociceptive Pain

© 2024 Peterson MD et al. *JAMA Neurology.*

M13111	Monoarthritis, not elsewhere classified, right shoulder	Arthritis Joint Upper Limb	Nociceptive Pain
M13112	Monoarthritis, not elsewhere classified, left shoulder	Arthritis Joint Upper Limb	Nociceptive Pain
M13119	Monoarthritis, not elsewhere classified, unsp shoulder	Arthritis Joint Upper Limb	Nociceptive Pain
M1312	Monoarthritis, not elsewhere classified, elbow	Arthritis Joint Upper Limb	Nociceptive Pain
M13121	Monoarthritis, not elsewhere classified, right elbow	Arthritis Joint Upper Limb	Nociceptive Pain
M13122	Monoarthritis, not elsewhere classified, left elbow	Arthritis Joint Upper Limb	Nociceptive Pain
M13129	Monoarthritis, not elsewhere classified, unspecified elbow	Arthritis Joint Upper Limb	Nociceptive Pain
M1313	Monoarthritis, not elsewhere classified, wrist	Arthritis Joint Upper Limb	Nociceptive Pain
M13131	Monoarthritis, not elsewhere classified, right wrist	Arthritis Joint Upper Limb	Nociceptive Pain
M13132	Monoarthritis, not elsewhere classified, left wrist	Arthritis Joint Upper Limb	Nociceptive Pain
M13139	Monoarthritis, not elsewhere classified, unspecified wrist	Arthritis Joint Upper Limb	Nociceptive Pain
M1314	Monoarthritis, not elsewhere classified, hand	Arthritis Joint Upper Limb	Nociceptive Pain
M13141	Monoarthritis, not elsewhere classified, right hand	Arthritis Joint Upper Limb	Nociceptive Pain
M13142	Monoarthritis, not elsewhere classified, left hand	Arthritis Joint Upper Limb	Nociceptive Pain
M13149	Monoarthritis, not elsewhere classified, unspecified hand	Arthritis Joint Upper Limb	Nociceptive Pain
M1315	Monoarthritis, not elsewhere classified, hip	Arthritis Joint Spine and Hips	Nociceptive Pain
M13151	Monoarthritis, not elsewhere classified, right hip	Arthritis Joint Spine and Hips	Nociceptive Pain
M13152	Monoarthritis, not elsewhere classified, left hip	Arthritis Joint Spine and Hips	Nociceptive Pain
M13159	Monoarthritis, not elsewhere classified, unspecified hip	Arthritis Joint Spine and Hips	Nociceptive Pain
M1316	Monoarthritis, not elsewhere classified, knee	Arthritis Joint Lower Limb	Nociceptive Pain
M13161	Monoarthritis, not elsewhere classified, right knee	Arthritis Joint Lower Limb	Nociceptive Pain
M13162	Monoarthritis, not elsewhere classified, left knee	Arthritis Joint Lower Limb	Nociceptive Pain
M13169	Monoarthritis, not elsewhere classified, unspecified knee	Arthritis Joint Lower Limb	Nociceptive Pain
M1317	Monoarthritis, not elsewhere classified, ankle and foot	Arthritis Joint Lower Limb	Nociceptive Pain
M13171	Monoarthritis, NEC, right ankle and foot	Arthritis Joint Lower Limb	Nociceptive Pain
M13172	Monoarthritis, not elsewhere classified, left ankle and foot	Arthritis Joint Lower Limb	Nociceptive Pain
M13179	Monoarthritis, not elsewhere classified, unsp ankle and foot	Arthritis Joint Lower Limb	Nociceptive Pain
M138	Other specified arthritis	Arthritis Joint Other	Nociceptive Pain
M1380	Other specified arthritis, unspecified site	Arthritis Joint Other	Nociceptive Pain
M1381	Other specified arthritis, shoulder	Arthritis Joint Upper Limb	Nociceptive Pain
M13811	Other specified arthritis, right shoulder	Arthritis Joint Upper Limb	Nociceptive Pain
M13812	Other specified arthritis, left shoulder	Arthritis Joint Upper Limb	Nociceptive Pain
M13819	Other specified arthritis, unspecified shoulder	Arthritis Joint Upper Limb	Nociceptive Pain
M1382	Other specified arthritis, elbow	Arthritis Joint Upper Limb	Nociceptive Pain
M13821	Other specified arthritis, right elbow	Arthritis Joint Upper Limb	Nociceptive Pain
M13822	Other specified arthritis, left elbow	Arthritis Joint Upper Limb	Nociceptive Pain
M13829	Other specified arthritis, unspecified elbow	Arthritis Joint Upper Limb	Nociceptive Pain

© 2024 Peterson MD et al. *JAMA Neurology.*

M1383	Other specified arthritis, wrist	Arthritis Joint Upper Limb	Nociceptive Pain
M13831	Other specified arthritis, right wrist	Arthritis Joint Upper Limb	Nociceptive Pain
M13832	Other specified arthritis, left wrist	Arthritis Joint Upper Limb	Nociceptive Pain
M13839	Other specified arthritis, unspecified wrist	Arthritis Joint Upper Limb	Nociceptive Pain
M1384	Other specified arthritis, hand	Arthritis Joint Upper Limb	Nociceptive Pain
M13841	Other specified arthritis, right hand	Arthritis Joint Upper Limb	Nociceptive Pain
M13842	Other specified arthritis, left hand	Arthritis Joint Upper Limb	Nociceptive Pain
M13849	Other specified arthritis, unspecified hand	Arthritis Joint Upper Limb	Nociceptive Pain
M1385	Other specified arthritis, hip	Arthritis Joint Spine and Hips	Nociceptive Pain
M13851	Other specified arthritis, right hip	Arthritis Joint Spine and Hips	Nociceptive Pain
M13852	Other specified arthritis, left hip	Arthritis Joint Spine and Hips	Nociceptive Pain
M13859	Other specified arthritis, unspecified hip	Arthritis Joint Spine and Hips	Nociceptive Pain
M1386	Other specified arthritis, knee	Arthritis Joint Lower Limb	Nociceptive Pain
M13861	Other specified arthritis, right knee	Arthritis Joint Lower Limb	Nociceptive Pain
M13862	Other specified arthritis, left knee	Arthritis Joint Lower Limb	Nociceptive Pain
M13869	Other specified arthritis, unspecified knee	Arthritis Joint Lower Limb	Nociceptive Pain
M1387	Other specified arthritis, ankle and foot	Arthritis Joint Lower Limb	Nociceptive Pain
M13871	Other specified arthritis, right ankle and foot	Arthritis Joint Lower Limb	Nociceptive Pain
M13872	Other specified arthritis, left ankle and foot	Arthritis Joint Lower Limb	Nociceptive Pain
M13879	Other specified arthritis, unspecified ankle and foot	Arthritis Joint Lower Limb	Nociceptive Pain
M1388	Other specified arthritis, other site	Arthritis Joint Other	Nociceptive Pain
M1389	Other specified arthritis, multiple sites	Arthritis Joint Other	Nociceptive Pain
M14	Arthropathies in other diseases classified elsewhere	Arthritis Joint Other	Nociceptive Pain
M146	Charcot's joint	Arthritis Joint Other	Nociceptive Pain
M1460	Charcot's joint, unspecified site	Arthritis Joint Other	Nociceptive Pain
M1461	Charcot's joint, shoulder	Arthritis Joint Upper Limb	Nociceptive Pain
M14611	Charcot's joint, right shoulder	Arthritis Joint Upper Limb	Nociceptive Pain
M14612	Charcot's joint, left shoulder	Arthritis Joint Upper Limb	Nociceptive Pain
M14619	Charcot's joint, unspecified shoulder	Arthritis Joint Upper Limb	Nociceptive Pain
M1462	Charcot's joint, elbow	Arthritis Joint Upper Limb	Nociceptive Pain
M14621	Charcot's joint, right elbow	Arthritis Joint Upper Limb	Nociceptive Pain
M14622	Charcot's joint, left elbow	Arthritis Joint Upper Limb	Nociceptive Pain
M14629	Charcot's joint, unspecified elbow	Arthritis Joint Upper Limb	Nociceptive Pain
M1463	Charcot's joint, wrist	Arthritis Joint Upper Limb	Nociceptive Pain
M14631	Charcot's joint, right wrist	Arthritis Joint Upper Limb	Nociceptive Pain
M14632	Charcot's joint, left wrist	Arthritis Joint Upper Limb	Nociceptive Pain
M14639	Charcot's joint, unspecified wrist	Arthritis Joint Upper Limb	Nociceptive Pain

© 2024 Peterson MD et al. *JAMA Neurology.*

M1464	Charcot's joint, hand	Arthritis Joint Upper Limb	Nociceptive Pain
M14641	Charcot's joint, right hand	Arthritis Joint Upper Limb	Nociceptive Pain
M14642	Charcot's joint, left hand	Arthritis Joint Upper Limb	Nociceptive Pain
M14649	Charcot's joint, unspecified hand	Arthritis Joint Upper Limb	Nociceptive Pain
M1465	Charcot's joint, hip	Arthritis Joint Spine and Hips	Nociceptive Pain
M14651	Charcot's joint, right hip	Arthritis Joint Spine and Hips	Nociceptive Pain
M14652	Charcot's joint, left hip	Arthritis Joint Spine and Hips	Nociceptive Pain
M14659	Charcot's joint, unspecified hip	Arthritis Joint Spine and Hips	Nociceptive Pain
M1466	Charcot's joint, knee	Arthritis Joint Lower Limb	Nociceptive Pain
M14661	Charcot's joint, right knee	Arthritis Joint Lower Limb	Nociceptive Pain
M14662	Charcot's joint, left knee	Arthritis Joint Lower Limb	Nociceptive Pain
M14669	Charcot's joint, unspecified knee	Arthritis Joint Lower Limb	Nociceptive Pain
M1467	Charcot's joint, ankle and foot	Arthritis Joint Lower Limb	Nociceptive Pain
M14671	Charcot's joint, right ankle and foot	Arthritis Joint Lower Limb	Nociceptive Pain
M14672	Charcot's joint, left ankle and foot	Arthritis Joint Lower Limb	Nociceptive Pain
M14679	Charcot's joint, unspecified ankle and foot	Arthritis Joint Lower Limb	Nociceptive Pain
M1468	Charcot's joint, vertebrae	Arthritis Joint Spine and Hips	Nociceptive Pain
M1469	Charcot's joint, multiple sites	Arthritis Joint Other	Nociceptive Pain
M148	Arthropathies in oth diseases classified elsewhere	Arthritis Joint Other	Nociceptive Pain
M1480	Arthropathies in oth diseases classd elswhr, unsp site	Arthritis Joint Other	Nociceptive Pain
M1481	Arthropathies in oth diseases classified elsewhere, shoulder	Arthritis Joint Upper Limb	Nociceptive Pain
M14811	Arthropathies in oth diseases classd elswhr, right shoulder	Arthritis Joint Upper Limb	Nociceptive Pain
M14812	Arthropathies in oth diseases classd elswhr, left shoulder	Arthritis Joint Upper Limb	Nociceptive Pain
M14819	Arthropathies in oth diseases classd elswhr, unsp shoulder	Arthritis Joint Upper Limb	Nociceptive Pain
M1482	Arthropathies in oth diseases classified elsewhere, elbow	Arthritis Joint Upper Limb	Nociceptive Pain
M14821	Arthropathies in oth diseases classd elswhr, right elbow	Arthritis Joint Upper Limb	Nociceptive Pain
M14822	Arthropathies in oth diseases classd elswhr, left elbow	Arthritis Joint Upper Limb	Nociceptive Pain
M14829	Arthropathies in oth diseases classd elswhr, unsp elbow	Arthritis Joint Upper Limb	Nociceptive Pain
M1483	Arthropathies in oth diseases classified elsewhere, wrist	Arthritis Joint Upper Limb	Nociceptive Pain
M14831	Arthropathies in oth diseases classd elswhr, right wrist	Arthritis Joint Upper Limb	Nociceptive Pain
M14832	Arthropathies in oth diseases classd elswhr, left wrist	Arthritis Joint Upper Limb	Nociceptive Pain
M14839	Arthropathies in oth diseases classd elswhr, unsp wrist	Arthritis Joint Upper Limb	Nociceptive Pain
M1484	Arthropathies in oth diseases classified elsewhere, hand	Arthritis Joint Upper Limb	Nociceptive Pain
M14841	Arthropathies in oth diseases classd elswhr, right hand	Arthritis Joint Upper Limb	Nociceptive Pain
M14842	Arthropathies in oth diseases classd elswhr, left hand	Arthritis Joint Upper Limb	Nociceptive Pain
M14849	Arthropathies in oth diseases classd elswhr, unsp hand	Arthritis Joint Upper Limb	Nociceptive Pain
M1485	Arthropathies in oth diseases classified elsewhere, hip	Arthritis Joint Spine and Hips	Nociceptive Pain

© 2024 Peterson MD et al. *JAMA Neurology.*

M14851	Arthropathies in oth diseases classd elswhr, right hip	Arthritis Joint Spine and Hips	Nociceptive Pain
M14852	Arthropathies in oth diseases classified elsewhere, left hip	Arthritis Joint Spine and Hips	Nociceptive Pain
M14859	Arthropathies in oth diseases classified elsewhere, unsp hip	Arthritis Joint Spine and Hips	Nociceptive Pain
M1486	Arthropathies in oth diseases classified elsewhere, knee	Arthritis Joint Lower Limb	Nociceptive Pain
M14861	Arthropathies in oth diseases classd elswhr, right knee	Arthritis Joint Lower Limb	Nociceptive Pain
M14862	Arthropathies in oth diseases classd elswhr, left knee	Arthritis Joint Lower Limb	Nociceptive Pain
M14869	Arthropathies in oth diseases classd elswhr, unsp knee	Arthritis Joint Lower Limb	Nociceptive Pain
M1487	Arthropathies in oth diseases classd elswhr, ankle and foot	Arthritis Joint Lower Limb	Nociceptive Pain
M14871	Arthropathies in oth diseases classd elswhr, right ank/ft	Arthritis Joint Lower Limb	Nociceptive Pain
M14872	Arthropathies in oth diseases classd elswhr, left ank/ft	Arthritis Joint Lower Limb	Nociceptive Pain
M14879	Arthropathies in oth diseases classd elswhr, unsp ank/ft	Arthritis Joint Lower Limb	Nociceptive Pain
M1488	Arthropathies in oth diseases classd elswhr, vertebrae	Arthritis Joint Spine and Hips	Nociceptive Pain
M1489	Arthropathies in oth diseases classd elswhr, multiple sites	Arthritis Joint Other	Nociceptive Pain
M15	Polyosteoarthritis	Arthritis Joint Other	Nociceptive Pain
M150	Primary generalized (osteo)arthritis	Arthritis Joint Other	Nociceptive Pain
M151	Heberden's nodes (with arthropathy)	Arthritis Joint Other	Nociceptive Pain
M152	Bouchard's nodes (with arthropathy)	Arthritis Joint Other	Nociceptive Pain
M153	Secondary multiple arthritis	Arthritis Joint Other	Nociceptive Pain
M154	Erosive (osteo)arthritis	Arthritis Joint Other	Nociceptive Pain
M158	Other polyosteoarthritis	Arthritis Joint Other	Nociceptive Pain
M159	Polyosteoarthritis, unspecified	Arthritis Joint Other	Nociceptive Pain
M16	Osteoarthritis of hip	Arthritis Joint Spine and Hips	Nociceptive Pain
M160	Bilateral primary osteoarthritis of hip	Arthritis Joint Spine and Hips	Nociceptive Pain
M161	Unilateral primary osteoarthritis of hip	Arthritis Joint Spine and Hips	Nociceptive Pain
M1610	Unilateral primary osteoarthritis, unspecified hip	Arthritis Joint Spine and Hips	Nociceptive Pain
M1611	Unilateral primary osteoarthritis, right hip	Arthritis Joint Spine and Hips	Nociceptive Pain
M1612	Unilateral primary osteoarthritis, left hip	Arthritis Joint Spine and Hips	Nociceptive Pain
M162	Bilateral osteoarthritis resulting from hip dysplasia	Arthritis Joint Spine and Hips	Nociceptive Pain
M163	Unilateral osteoarthritis resulting from hip dysplasia	Arthritis Joint Spine and Hips	Nociceptive Pain
M1630	Unilateral osteoarth resulting from hip dysplasia, unsp hip	Arthritis Joint Spine and Hips	Nociceptive Pain
M1631	Unilateral osteoarth resulting from hip dysplasia, right hip	Arthritis Joint Spine and Hips	Nociceptive Pain
M1632	Unilateral osteoarth resulting from hip dysplasia, left hip	Arthritis Joint Spine and Hips	Nociceptive Pain
M164	Bilateral post-traumatic osteoarthritis of hip	Arthritis Joint Spine and Hips	Nociceptive Pain
M165	Unilateral post-traumatic osteoarthritis of hip	Arthritis Joint Spine and Hips	Nociceptive Pain
M1650	Unilateral post-traumatic osteoarthritis, unspecified hip	Arthritis Joint Spine and Hips	Nociceptive Pain
M1651	Unilateral post-traumatic osteoarthritis, right hip	Arthritis Joint Spine and Hips	Nociceptive Pain
M1652	Unilateral post-traumatic osteoarthritis, left hip	Arthritis Joint Spine and Hips	Nociceptive Pain

© 2024 Peterson MD et al. *JAMA Neurology.*

M166	Other bilateral secondary osteoarthritis of hip	Arthritis Joint Spine and Hips	Nociceptive Pain
M167	Other unilateral secondary osteoarthritis of hip	Arthritis Joint Spine and Hips	Nociceptive Pain
M169	Osteoarthritis of hip, unspecified	Arthritis Joint Spine and Hips	Nociceptive Pain
M17	Osteoarthritis of knee	Arthritis Joint Lower Limb	Nociceptive Pain
M170	Bilateral primary osteoarthritis of knee	Arthritis Joint Lower Limb	Nociceptive Pain
M171	Unilateral primary osteoarthritis of knee	Arthritis Joint Lower Limb	Nociceptive Pain
M1710	Unilateral primary osteoarthritis, unspecified knee	Arthritis Joint Lower Limb	Nociceptive Pain
M1711	Unilateral primary osteoarthritis, right knee	Arthritis Joint Lower Limb	Nociceptive Pain
M1712	Unilateral primary osteoarthritis, left knee	Arthritis Joint Lower Limb	Nociceptive Pain
M172	Bilateral post-traumatic osteoarthritis of knee	Arthritis Joint Lower Limb	Nociceptive Pain
M173	Unilateral post-traumatic osteoarthritis of knee	Arthritis Joint Lower Limb	Nociceptive Pain
M1730	Unilateral post-traumatic osteoarthritis, unspecified knee	Arthritis Joint Lower Limb	Nociceptive Pain
M1731	Unilateral post-traumatic osteoarthritis, right knee	Arthritis Joint Lower Limb	Nociceptive Pain
M1732	Unilateral post-traumatic osteoarthritis, left knee	Arthritis Joint Lower Limb	Nociceptive Pain
M174	Other bilateral secondary osteoarthritis of knee	Arthritis Joint Lower Limb	Nociceptive Pain
M175	Other unilateral secondary osteoarthritis of knee	Arthritis Joint Lower Limb	Nociceptive Pain
M179	Osteoarthritis of knee, unspecified	Arthritis Joint Lower Limb	Nociceptive Pain
M18	Osteoarthritis of first carpometacarpal joint	Arthritis Joint Upper Limb	Nociceptive Pain
M180	Bilateral primary osteoarth of first carpometacarp joints	Arthritis Joint Upper Limb	Nociceptive Pain
M181	Unilateral primary osteoarth of first carpometacarp joint	Arthritis Joint Upper Limb	Nociceptive Pain
M1810	Unil prim osteoarth of first carpometacarp joint, unsp hand	Arthritis Joint Upper Limb	Nociceptive Pain
M1811	Unil primary osteoarth of first carpometacarp joint, r hand	Arthritis Joint Upper Limb	Nociceptive Pain
M1812	Unil primary osteoarth of first carpometacarp joint, l hand	Arthritis Joint Upper Limb	Nociceptive Pain
M182	Bi post-trauma osteoarth of first carpometacarp joints	Arthritis Joint Upper Limb	Nociceptive Pain
M183	Unil post-trauma osteoarth of first carpometacarp joint	Arthritis Joint Upper Limb	Nociceptive Pain
M1830	Unil post-trauma osteoarth of 1st carpometacarp jt,unsp hand	Arthritis Joint Upper Limb	Nociceptive Pain
M1831	Unil post-trauma osteoarth of 1st carpometacarp jt, r hand	Arthritis Joint Upper Limb	Nociceptive Pain
M1832	Unil post-trauma osteoarth of 1st carpometacarp jt, l hand	Arthritis Joint Upper Limb	Nociceptive Pain
M184	Oth bi secondary osteoarth of first carpometacarp joints	Arthritis Joint Upper Limb	Nociceptive Pain
M185	Oth unil secondary osteoarth of first carpometacarp joint	Arthritis Joint Upper Limb	Nociceptive Pain
M1850	Oth unil sec osteoarth of 1st carpometacarp joint, unsp hand	Arthritis Joint Upper Limb	Nociceptive Pain
M1851	Oth unil sec osteoarth of first carpometacarp joint, r hand	Arthritis Joint Upper Limb	Nociceptive Pain
M1852	Oth unil sec osteoarth of first carpometacarp joint, l hand	Arthritis Joint Upper Limb	Nociceptive Pain
M189	Osteoarthritis of first carpometacarpal joint, unspecified	Arthritis Joint Upper Limb	Nociceptive Pain
M19	Other and unspecified osteoarthritis	Arthritis Joint Other	Nociceptive Pain
M190	Primary osteoarthritis of other joints	Arthritis Joint Other	Nociceptive Pain
M1901	Primary osteoarthritis, shoulder	Arthritis Joint Upper Limb	Nociceptive Pain

© 2024 Peterson MD et al. *JAMA Neurology.*

M19011	Primary osteoarthritis, right shoulder	Arthritis Joint Upper Limb	Nociceptive Pain
M19012	Primary osteoarthritis, left shoulder	Arthritis Joint Upper Limb	Nociceptive Pain
M19019	Primary osteoarthritis, unspecified shoulder	Arthritis Joint Upper Limb	Nociceptive Pain
M1902	Primary osteoarthritis, elbow	Arthritis Joint Upper Limb	Nociceptive Pain
M19021	Primary osteoarthritis, right elbow	Arthritis Joint Upper Limb	Nociceptive Pain
M19022	Primary osteoarthritis, left elbow	Arthritis Joint Upper Limb	Nociceptive Pain
M19029	Primary osteoarthritis, unspecified elbow	Arthritis Joint Upper Limb	Nociceptive Pain
M1903	Primary osteoarthritis, wrist	Arthritis Joint Upper Limb	Nociceptive Pain
M19031	Primary osteoarthritis, right wrist	Arthritis Joint Upper Limb	Nociceptive Pain
M19032	Primary osteoarthritis, left wrist	Arthritis Joint Upper Limb	Nociceptive Pain
M19039	Primary osteoarthritis, unspecified wrist	Arthritis Joint Upper Limb	Nociceptive Pain
M1904	Primary osteoarthritis, hand	Arthritis Joint Upper Limb	Nociceptive Pain
M19041	Primary osteoarthritis, right hand	Arthritis Joint Upper Limb	Nociceptive Pain
M19042	Primary osteoarthritis, left hand	Arthritis Joint Upper Limb	Nociceptive Pain
M19049	Primary osteoarthritis, unspecified hand	Arthritis Joint Upper Limb	Nociceptive Pain
M1907	Primary osteoarthritis ankle and foot	Arthritis Joint Lower Limb	Nociceptive Pain
M19071	Primary osteoarthritis, right ankle and foot	Arthritis Joint Lower Limb	Nociceptive Pain
M19072	Primary osteoarthritis, left ankle and foot	Arthritis Joint Lower Limb	Nociceptive Pain
M19079	Primary osteoarthritis, unspecified ankle and foot	Arthritis Joint Lower Limb	Nociceptive Pain
M1909	Primary osteoarthritis, other specified site	Arthritis Joint Other	Nociceptive Pain
M191	Post-traumatic osteoarthritis of other joints	Arthritis Joint Other	Nociceptive Pain
M1911	Post-traumatic osteoarthritis, shoulder	Arthritis Joint Upper Limb	Nociceptive Pain
M19111	Post-traumatic osteoarthritis, right shoulder	Arthritis Joint Upper Limb	Nociceptive Pain
M19112	Post-traumatic osteoarthritis, left shoulder	Arthritis Joint Upper Limb	Nociceptive Pain
M19119	Post-traumatic osteoarthritis, unspecified shoulder	Arthritis Joint Upper Limb	Nociceptive Pain
M1912	Post-traumatic osteoarthritis, elbow	Arthritis Joint Upper Limb	Nociceptive Pain
M19121	Post-traumatic osteoarthritis, right elbow	Arthritis Joint Upper Limb	Nociceptive Pain
M19122	Post-traumatic osteoarthritis, left elbow	Arthritis Joint Upper Limb	Nociceptive Pain
M19129	Post-traumatic osteoarthritis, unspecified elbow	Arthritis Joint Upper Limb	Nociceptive Pain
M1913	Post-traumatic osteoarthritis, wrist	Arthritis Joint Upper Limb	Nociceptive Pain
M19131	Post-traumatic osteoarthritis, right wrist	Arthritis Joint Upper Limb	Nociceptive Pain
M19132	Post-traumatic osteoarthritis, left wrist	Arthritis Joint Upper Limb	Nociceptive Pain
M19139	Post-traumatic osteoarthritis, unspecified wrist	Arthritis Joint Upper Limb	Nociceptive Pain
M1914	Post-traumatic osteoarthritis, hand	Arthritis Joint Upper Limb	Nociceptive Pain
M19141	Post-traumatic osteoarthritis, right hand	Arthritis Joint Upper Limb	Nociceptive Pain
M19142	Post-traumatic osteoarthritis, left hand	Arthritis Joint Upper Limb	Nociceptive Pain
M19149	Post-traumatic osteoarthritis, unspecified hand	Arthritis Joint Upper Limb	Nociceptive Pain

© 2024 Peterson MD et al. *JAMA Neurology.*

M1917	Post-traumatic osteoarthritis, ankle and foot	Arthritis Joint Lower Limb	Nociceptive Pain
M19171	Post-traumatic osteoarthritis, right ankle and foot	Arthritis Joint Lower Limb	Nociceptive Pain
M19172	Post-traumatic osteoarthritis, left ankle and foot	Arthritis Joint Lower Limb	Nociceptive Pain
M19179	Post-traumatic osteoarthritis, unspecified ankle and foot	Arthritis Joint Lower Limb	Nociceptive Pain
M1919	Post-traumatic osteoarthritis, other specified site	Arthritis Joint Other	Nociceptive Pain
M192	Secondary osteoarthritis of other joints	Arthritis Joint Other	Nociceptive Pain
M1921	Secondary osteoarthritis, shoulder	Arthritis Joint Upper Limb	Nociceptive Pain
M19211	Secondary osteoarthritis, right shoulder	Arthritis Joint Upper Limb	Nociceptive Pain
M19212	Secondary osteoarthritis, left shoulder	Arthritis Joint Upper Limb	Nociceptive Pain
M19219	Secondary osteoarthritis, unspecified shoulder	Arthritis Joint Upper Limb	Nociceptive Pain
M1922	Secondary osteoarthritis, elbow	Arthritis Joint Upper Limb	Nociceptive Pain
M19221	Secondary osteoarthritis, right elbow	Arthritis Joint Upper Limb	Nociceptive Pain
M19222	Secondary osteoarthritis, left elbow	Arthritis Joint Upper Limb	Nociceptive Pain
M19229	Secondary osteoarthritis, unspecified elbow	Arthritis Joint Upper Limb	Nociceptive Pain
M1923	Secondary osteoarthritis, wrist	Arthritis Joint Upper Limb	Nociceptive Pain
M19231	Secondary osteoarthritis, right wrist	Arthritis Joint Upper Limb	Nociceptive Pain
M19232	Secondary osteoarthritis, left wrist	Arthritis Joint Upper Limb	Nociceptive Pain
M19239	Secondary osteoarthritis, unspecified wrist	Arthritis Joint Upper Limb	Nociceptive Pain
M1924	Secondary osteoarthritis, hand	Arthritis Joint Upper Limb	Nociceptive Pain
M19241	Secondary osteoarthritis, right hand	Arthritis Joint Upper Limb	Nociceptive Pain
M19242	Secondary osteoarthritis, left hand	Arthritis Joint Upper Limb	Nociceptive Pain
M19249	Secondary osteoarthritis, unspecified hand	Arthritis Joint Upper Limb	Nociceptive Pain
M1927	Secondary osteoarthritis, ankle and foot	Arthritis Joint Lower Limb	Nociceptive Pain
M19271	Secondary osteoarthritis, right ankle and foot	Arthritis Joint Lower Limb	Nociceptive Pain
M19272	Secondary osteoarthritis, left ankle and foot	Arthritis Joint Lower Limb	Nociceptive Pain
M19279	Secondary osteoarthritis, unspecified ankle and foot	Arthritis Joint Lower Limb	Nociceptive Pain
M1929	Secondary osteoarthritis, other specified site	Arthritis Joint Other	Nociceptive Pain
M199	Osteoarthritis, unspecified site	Arthritis Joint Other	Nociceptive Pain
M1990	Unspecified osteoarthritis, unspecified site	Arthritis Joint Other	Nociceptive Pain
M1991	Primary osteoarthritis, unspecified site	Arthritis Joint Other	Nociceptive Pain
M1992	Post-traumatic osteoarthritis, unspecified site	Arthritis Joint Other	Nociceptive Pain
M1993	Secondary osteoarthritis, unspecified site	Arthritis Joint Other	Nociceptive Pain
M20	Acquired deformities of fingers and toes	Arthritis Joint Other	Nociceptive Pain
M200	Deformity of finger(s)	Arthritis Joint Other	Nociceptive Pain
M2000	Unspecified deformity of finger(s)	Arthritis Joint Other	Nociceptive Pain
M20001	Unspecified deformity of right finger(s)	Arthritis Joint Other	Nociceptive Pain
M20002	Unspecified deformity of left finger(s)	Arthritis Joint Upper Limb	Nociceptive Pain

© 2024 Peterson MD et al. *JAMA Neurology.*

M20009	Unspecified deformity of unspecified finger(s)	Arthritis Joint Upper Limb	Nociceptive Pain
M2001	Mallet finger	Arthritis Joint Upper Limb	Nociceptive Pain
M20011	Mallet finger of right finger(s)	Arthritis Joint Upper Limb	Nociceptive Pain
M20012	Mallet finger of left finger(s)	Arthritis Joint Upper Limb	Nociceptive Pain
M20019	Mallet finger of unspecified finger(s)	Arthritis Joint Upper Limb	Nociceptive Pain
M2002	Boutonniere deformity	Arthritis Joint Upper Limb	Nociceptive Pain
M20021	Boutonniere deformity of right finger(s)	Arthritis Joint Upper Limb	Nociceptive Pain
M20022	Boutonniere deformity of left finger(s)	Arthritis Joint Upper Limb	Nociceptive Pain
M20029	Boutonniere deformity of unspecified finger(s)	Arthritis Joint Upper Limb	Nociceptive Pain
M2003	Swan-neck deformity	Arthritis Joint Upper Limb	Nociceptive Pain
M20031	Swan-neck deformity of right finger(s)	Arthritis Joint Upper Limb	Nociceptive Pain
M20032	Swan-neck deformity of left finger(s)	Arthritis Joint Upper Limb	Nociceptive Pain
M20039	Swan-neck deformity of unspecified finger(s)	Arthritis Joint Upper Limb	Nociceptive Pain
M2009	Other deformity of finger(s)	Arthritis Joint Upper Limb	Nociceptive Pain
M20091	Other deformity of right finger(s)	Arthritis Joint Upper Limb	Nociceptive Pain
M20092	Other deformity of left finger(s)	Arthritis Joint Upper Limb	Nociceptive Pain
M20099	Other deformity of finger(s), unspecified finger(s)	Arthritis Joint Upper Limb	Nociceptive Pain
M201	Hallux valgus (acquired)	Arthritis Joint Lower Limb	Nociceptive Pain
M2010	Hallux valgus (acquired), unspecified foot	Arthritis Joint Lower Limb	Nociceptive Pain
M2011	Hallux valgus (acquired), right foot	Arthritis Joint Lower Limb	Nociceptive Pain
M2012	Hallux valgus (acquired), left foot	Arthritis Joint Lower Limb	Nociceptive Pain
M202	Hallux rigidus	Arthritis Joint Lower Limb	Nociceptive Pain
M2020	Hallux rigidus, unspecified foot	Arthritis Joint Lower Limb	Nociceptive Pain
M2021	Hallux rigidus, right foot	Arthritis Joint Lower Limb	Nociceptive Pain
M2022	Hallux rigidus, left foot	Arthritis Joint Lower Limb	Nociceptive Pain
M203	Hallux varus (acquired)	Arthritis Joint Lower Limb	Nociceptive Pain
M2030	Hallux varus (acquired), unspecified foot	Arthritis Joint Lower Limb	Nociceptive Pain
M2031	Hallux varus (acquired), right foot	Arthritis Joint Lower Limb	Nociceptive Pain
M2032	Hallux varus (acquired), left foot	Arthritis Joint Lower Limb	Nociceptive Pain
M204	Other hammer toe(s) (acquired)	Arthritis Joint Lower Limb	Nociceptive Pain
M2040	Other hammer toe(s) (acquired), unspecified foot	Arthritis Joint Lower Limb	Nociceptive Pain
M2041	Other hammer toe(s) (acquired), right foot	Arthritis Joint Lower Limb	Nociceptive Pain
M2042	Other hammer toe(s) (acquired), left foot	Arthritis Joint Lower Limb	Nociceptive Pain
M205	Other deformities of toe(s) (acquired)	Arthritis Joint Lower Limb	Nociceptive Pain
M205X	Other deformities of toe(s) (acquired)	Arthritis Joint Lower Limb	Nociceptive Pain
M205X1	Other deformities of toe(s) (acquired), right foot	Arthritis Joint Lower Limb	Nociceptive Pain
M205X2	Other deformities of toe(s) (acquired), left foot	Arthritis Joint Lower Limb	Nociceptive Pain

© 2024 Peterson MD et al. *JAMA Neurology.*

M205X9	Other deformities of toe(s) (acquired), unspecified foot	Arthritis Joint Lower Limb	Nociceptive Pain
M206	Acquired deformities of toe(s), unspecified	Arthritis Joint Lower Limb	Nociceptive Pain
M2060	Acquired deformities of toe(s), unsp, unspecified foot	Arthritis Joint Lower Limb	Nociceptive Pain
M2061	Acquired deformities of toe(s), unspecified, right foot	Arthritis Joint Lower Limb	Nociceptive Pain
M2062	Acquired deformities of toe(s), unspecified, left foot	Arthritis Joint Lower Limb	Nociceptive Pain
M21	Other acquired deformities of limbs	Arthritis Joint Other	Nociceptive Pain
M210	Valgus deformity, not elsewhere classified	Arthritis Joint Other	Nociceptive Pain
M2100	Valgus deformity, not elsewhere classified, unspecified site	Arthritis Joint Other	Nociceptive Pain
M2102	Valgus deformity, not elsewhere classified, elbow	Arthritis Joint Upper Limb	Nociceptive Pain
M21021	Valgus deformity, not elsewhere classified, right elbow	Arthritis Joint Upper Limb	Nociceptive Pain
M21022	Valgus deformity, not elsewhere classified, left elbow	Arthritis Joint Upper Limb	Nociceptive Pain
M21029	Valgus deformity, not elsewhere classified, unsp elbow	Arthritis Joint Upper Limb	Nociceptive Pain
M2105	Valgus deformity, not elsewhere classified, hip	Arthritis Joint Spine and Hips	Nociceptive Pain
M21051	Valgus deformity, not elsewhere classified, right hip	Arthritis Joint Spine and Hips	Nociceptive Pain
M21052	Valgus deformity, not elsewhere classified, left hip	Arthritis Joint Spine and Hips	Nociceptive Pain
M21059	Valgus deformity, not elsewhere classified, unspecified hip	Arthritis Joint Spine and Hips	Nociceptive Pain
M2106	Valgus deformity, not elsewhere classified, knee	Arthritis Joint Lower Limb	Nociceptive Pain
M21061	Valgus deformity, not elsewhere classified, right knee	Arthritis Joint Lower Limb	Nociceptive Pain
M21062	Valgus deformity, not elsewhere classified, left knee	Arthritis Joint Lower Limb	Nociceptive Pain
M21069	Valgus deformity, not elsewhere classified, unspecified knee	Arthritis Joint Lower Limb	Nociceptive Pain
M2107	Valgus deformity, not elsewhere classified, ankle	Arthritis Joint Lower Limb	Nociceptive Pain
M21071	Valgus deformity, not elsewhere classified, right ankle	Arthritis Joint Lower Limb	Nociceptive Pain
M21072	Valgus deformity, not elsewhere classified, left ankle	Arthritis Joint Lower Limb	Nociceptive Pain
M21079	Valgus deformity, not elsewhere classified, unsp ankle	Arthritis Joint Lower Limb	Nociceptive Pain
M211	Varus deformity, not elsewhere classified	Arthritis Joint Other	Nociceptive Pain
M2110	Varus deformity, not elsewhere classified, unspecified site	Arthritis Joint Other	Nociceptive Pain
M2112	Varus deformity, not elsewhere classified, elbow	Arthritis Joint Upper Limb	Nociceptive Pain
M21121	Varus deformity, not elsewhere classified, right elbow	Arthritis Joint Upper Limb	Nociceptive Pain
M21122	Varus deformity, not elsewhere classified, left elbow	Arthritis Joint Upper Limb	Nociceptive Pain
M21129	Varus deformity, not elsewhere classified, unspecified elbow	Arthritis Joint Upper Limb	Nociceptive Pain
M2115	Varus deformity, not elsewhere classified, hip	Arthritis Joint Spine and Hips	Nociceptive Pain
M21151	Varus deformity, not elsewhere classified, right hip	Arthritis Joint Spine and Hips	Nociceptive Pain
M21152	Varus deformity, not elsewhere classified, left hip	Arthritis Joint Spine and Hips	Nociceptive Pain
M21159	Varus deformity, not elsewhere classified, unspecified	Arthritis Joint Other	Nociceptive Pain
M2116	Varus deformity, not elsewhere classified, knee	Arthritis Joint Lower Limb	Nociceptive Pain
M21161	Varus deformity, not elsewhere classified, right knee	Arthritis Joint Lower Limb	Nociceptive Pain
M21162	Varus deformity, not elsewhere classified, left knee	Arthritis Joint Lower Limb	Nociceptive Pain

© 2024 Peterson MD et al. *JAMA Neurology.*

M21169	Varus deformity, not elsewhere classified, unspecified knee	Arthritis Joint Lower Limb	Nociceptive Pain
M2117	Varus deformity, not elsewhere classified, ankle	Arthritis Joint Lower Limb	Nociceptive Pain
M21171	Varus deformity, not elsewhere classified, right ankle	Arthritis Joint Lower Limb	Nociceptive Pain
M21172	Varus deformity, not elsewhere classified, left ankle	Arthritis Joint Lower Limb	Nociceptive Pain
M21179	Varus deformity, not elsewhere classified, unspecified ankle	Arthritis Joint Lower Limb	Nociceptive Pain
M212	Flexion deformity	Arthritis Joint Other	Nociceptive Pain
M2120	Flexion deformity, unspecified site	Arthritis Joint Other	Nociceptive Pain
M2121	Flexion deformity, shoulder	Arthritis Joint Upper Limb	Nociceptive Pain
M21211	Flexion deformity, right shoulder	Arthritis Joint Upper Limb	Nociceptive Pain
M21212	Flexion deformity, left shoulder	Arthritis Joint Upper Limb	Nociceptive Pain
M21219	Flexion deformity, unspecified shoulder	Arthritis Joint Upper Limb	Nociceptive Pain
M2122	Flexion deformity, elbow	Arthritis Joint Upper Limb	Nociceptive Pain
M21221	Flexion deformity, right elbow	Arthritis Joint Upper Limb	Nociceptive Pain
M21222	Flexion deformity, left elbow	Arthritis Joint Upper Limb	Nociceptive Pain
M21229	Flexion deformity, unspecified elbow	Arthritis Joint Upper Limb	Nociceptive Pain
M2123	Flexion deformity, wrist	Arthritis Joint Upper Limb	Nociceptive Pain
M21231	Flexion deformity, right wrist	Arthritis Joint Upper Limb	Nociceptive Pain
M21232	Flexion deformity, left wrist	Arthritis Joint Upper Limb	Nociceptive Pain
M21239	Flexion deformity, unspecified wrist	Arthritis Joint Upper Limb	Nociceptive Pain
M2124	Flexion deformity, finger joints	Arthritis Joint Upper Limb	Nociceptive Pain
M21241	Flexion deformity, right finger joints	Arthritis Joint Upper Limb	Nociceptive Pain
M21242	Flexion deformity, left finger joints	Arthritis Joint Upper Limb	Nociceptive Pain
M21249	Flexion deformity, unspecified finger joints	Arthritis Joint Upper Limb	Nociceptive Pain
M2125	Flexion deformity, hip	Arthritis Joint Spine and Hips	Nociceptive Pain
M21251	Flexion deformity, right hip	Arthritis Joint Spine and Hips	Nociceptive Pain
M21252	Flexion deformity, left hip	Arthritis Joint Spine and Hips	Nociceptive Pain
M21259	Flexion deformity, unspecified hip	Arthritis Joint Spine and Hips	Nociceptive Pain
M2126	Flexion deformity, knee	Arthritis Joint Lower Limb	Nociceptive Pain
M21261	Flexion deformity, right knee	Arthritis Joint Lower Limb	Nociceptive Pain
M21262	Flexion deformity, left knee	Arthritis Joint Lower Limb	Nociceptive Pain
M21269	Flexion deformity, unspecified knee	Arthritis Joint Lower Limb	Nociceptive Pain
M2127	Flexion deformity, ankle and toes	Arthritis Joint Lower Limb	Nociceptive Pain
M21271	Flexion deformity, right ankle and toes	Arthritis Joint Lower Limb	Nociceptive Pain
M21272	Flexion deformity, left ankle and toes	Arthritis Joint Lower Limb	Nociceptive Pain
M21279	Flexion deformity, unspecified ankle and toes	Arthritis Joint Lower Limb	Nociceptive Pain
M213	Wrist or foot drop (acquired)	Arthritis Joint Other	Nociceptive Pain
M2133	Wrist drop (acquired)	Arthritis Joint Upper Limb	Nociceptive Pain

© 2024 Peterson MD et al. *JAMA Neurology.*

M21331	Wrist drop, right wrist	Arthritis Joint Upper Limb	Nociceptive Pain
M21332	Wrist drop, left wrist	Arthritis Joint Upper Limb	Nociceptive Pain
M21339	Wrist drop, unspecified wrist	Arthritis Joint Upper Limb	Nociceptive Pain
M2137	Foot drop (acquired)	Arthritis Joint Lower Limb	Nociceptive Pain
M21371	Foot drop, right foot	Arthritis Joint Lower Limb	Nociceptive Pain
M21372	Foot drop, left foot	Arthritis Joint Lower Limb	Nociceptive Pain
M21379	Foot drop, unspecified foot	Arthritis Joint Lower Limb	Nociceptive Pain
M214	Flat foot [pes planus] (acquired)	Arthritis Joint Lower Limb	Nociceptive Pain
M2140	Flat foot [pes planus] (acquired), unspecified foot	Arthritis Joint Lower Limb	Nociceptive Pain
M2141	Flat foot [pes planus] (acquired), right foot	Arthritis Joint Lower Limb	Nociceptive Pain
M2142	Flat foot [pes planus] (acquired), left foot	Arthritis Joint Lower Limb	Nociceptive Pain
M215	Acquired clawhand, clubhand, clawfoot and clubfoot	Arthritis Joint Other	Nociceptive Pain
M2151	Acquired clawhand	Arthritis Joint Upper Limb	Nociceptive Pain
M21511	Acquired clawhand, right hand	Arthritis Joint Upper Limb	Nociceptive Pain
M21512	Acquired clawhand, left hand	Arthritis Joint Upper Limb	Nociceptive Pain
M21519	Acquired clawhand, unspecified hand	Arthritis Joint Upper Limb	Nociceptive Pain
M2152	Acquired clubhand	Arthritis Joint Upper Limb	Nociceptive Pain
M21521	Acquired clubhand, right hand	Arthritis Joint Upper Limb	Nociceptive Pain
M21522	Acquired clubhand, left hand	Arthritis Joint Upper Limb	Nociceptive Pain
M21529	Acquired clubhand, unspecified hand	Arthritis Joint Upper Limb	Nociceptive Pain
M2153	Acquired clawfoot	Arthritis Joint Lower Limb	Nociceptive Pain
M21531	Acquired clawfoot, right foot	Arthritis Joint Lower Limb	Nociceptive Pain
M21532	Acquired clawfoot, left foot	Arthritis Joint Lower Limb	Nociceptive Pain
M21539	Acquired clawfoot, unspecified foot	Arthritis Joint Lower Limb	Nociceptive Pain
M2154	Acquired clubfoot	Arthritis Joint Lower Limb	Nociceptive Pain
M21541	Acquired clubfoot, right foot	Arthritis Joint Lower Limb	Nociceptive Pain
M21542	Acquired clubfoot, left foot	Arthritis Joint Lower Limb	Nociceptive Pain
M21549	Acquired clubfoot, unspecified foot	Arthritis Joint Lower Limb	Nociceptive Pain
M216	Other acquired deformities of foot	Arthritis Joint Lower Limb	Nociceptive Pain
M2161	Bunion	Arthritis Joint Lower Limb	Nociceptive Pain
M21611	Bunion of right foot	Arthritis Joint Lower Limb	Nociceptive Pain
M21612	Bunion of left foot	Arthritis Joint Lower Limb	Nociceptive Pain
M21619	Bunion of unspecified foot	Arthritis Joint Lower Limb	Nociceptive Pain
M2162	Bunionette	Arthritis Joint Lower Limb	Nociceptive Pain
M21621	Bunionette of right foot	Arthritis Joint Lower Limb	Nociceptive Pain
M21622	Bunionette of left foot	Arthritis Joint Lower Limb	Nociceptive Pain
M21629	Bunionette of unspecified foot	Arthritis Joint Lower Limb	Nociceptive Pain

© 2024 Peterson MD et al. *JAMA Neurology.*

M216X	Other acquired deformities of foot	Arthritis Joint Lower Limb	Nociceptive Pain
M216X1	Other acquired deformities of right foot	Arthritis Joint Lower Limb	Nociceptive Pain
M216X2	Other acquired deformities of left foot	Arthritis Joint Lower Limb	Nociceptive Pain
M216X9	Other acquired deformities of unspecified foot	Arthritis Joint Lower Limb	Nociceptive Pain
M217	Unequal limb length (acquired)	Arthritis Joint Other	Nociceptive Pain
M2170	Unequal limb length (acquired), unspecified site	Arthritis Joint Other	Nociceptive Pain
M2172	Unequal limb length (acquired), humerus	Arthritis Joint Upper Limb	Nociceptive Pain
M21721	Unequal limb length (acquired), right humerus	Arthritis Joint Upper Limb	Nociceptive Pain
M21722	Unequal limb length (acquired), left humerus	Arthritis Joint Upper Limb	Nociceptive Pain
M21729	Unequal limb length (acquired), unspecified humerus	Arthritis Joint Upper Limb	Nociceptive Pain
M2173	Unequal limb length (acquired), ulna and radius	Arthritis Joint Upper Limb	Nociceptive Pain
M21731	Unequal limb length (acquired), right ulna	Arthritis Joint Upper Limb	Nociceptive Pain
M21732	Unequal limb length (acquired), left ulna	Arthritis Joint Upper Limb	Nociceptive Pain
M21733	Unequal limb length (acquired), right radius	Arthritis Joint Upper Limb	Nociceptive Pain
M21734	Unequal limb length (acquired), left radius	Arthritis Joint Upper Limb	Nociceptive Pain
M21739	Unequal limb length (acquired), unspecified ulna and radius	Arthritis Joint Upper Limb	Nociceptive Pain
M2175	Unequal limb length (acquired), femur	Arthritis Joint Lower Limb	Nociceptive Pain
M21751	Unequal limb length (acquired), right femur	Arthritis Joint Lower Limb	Nociceptive Pain
M21752	Unequal limb length (acquired), left femur	Arthritis Joint Lower Limb	Nociceptive Pain
M21759	Unequal limb length (acquired), unspecified femur	Arthritis Joint Lower Limb	Nociceptive Pain
M2176	Unequal limb length (acquired), tibia and fibula	Arthritis Joint Lower Limb	Nociceptive Pain
M21761	Unequal limb length (acquired), right tibia	Arthritis Joint Lower Limb	Nociceptive Pain
M21762	Unequal limb length (acquired), left tibia	Arthritis Joint Lower Limb	Nociceptive Pain
M21763	Unequal limb length (acquired), right fibula	Arthritis Joint Lower Limb	Nociceptive Pain
M21764	Unequal limb length (acquired), left fibula	Arthritis Joint Lower Limb	Nociceptive Pain
M21769	Unequal limb length (acquired), unspecified tibia and fibula	Arthritis Joint Lower Limb	Nociceptive Pain
M218	Other specified acquired deformities of limbs	Arthritis Joint Other	Nociceptive Pain
M2180	Other specified acquired deformities of unspecified limb	Arthritis Joint Other	Nociceptive Pain
M2182	Other specified acquired deformities of upper arm	Arthritis Joint Upper Limb	Nociceptive Pain
M21821	Other specified acquired deformities of right upper arm	Arthritis Joint Upper Limb	Nociceptive Pain
M21822	Other specified acquired deformities of left upper arm	Arthritis Joint Upper Limb	Nociceptive Pain
M21829	Oth acquired deformities of unspecified upper arm	Arthritis Joint Upper Limb	Nociceptive Pain
M2183	Other specified acquired deformities of forearm	Arthritis Joint Upper Limb	Nociceptive Pain
M21831	Other specified acquired deformities of right forearm	Arthritis Joint Upper Limb	Nociceptive Pain
M21832	Other specified acquired deformities of left forearm	Arthritis Joint Upper Limb	Nociceptive Pain
M21839	Other specified acquired deformities of unspecified forearm	Arthritis Joint Upper Limb	Nociceptive Pain
M2185	Other specified acquired deformities of thigh	Arthritis Joint Lower Limb	Nociceptive Pain

© 2024 Peterson MD et al. *JAMA Neurology.*

M21851	Other specified acquired deformities of right thigh	Arthritis Joint Lower Limb	Nociceptive Pain
M21852	Other specified acquired deformities of left thigh	Arthritis Joint Lower Limb	Nociceptive Pain
M21859	Other specified acquired deformities of unspecified thigh	Arthritis Joint Lower Limb	Nociceptive Pain
M2186	Other specified acquired deformities of lower leg	Arthritis Joint Lower Limb	Nociceptive Pain
M21861	Other specified acquired deformities of right lower leg	Arthritis Joint Lower Limb	Nociceptive Pain
M21862	Other specified acquired deformities of left lower leg	Arthritis Joint Lower Limb	Nociceptive Pain
M21869	Oth acquired deformities of unspecified lower leg	Arthritis Joint Lower Limb	Nociceptive Pain
M219	Unspecified acquired deformity of limb and hand	Arthritis Joint Upper Limb	Nociceptive Pain
M2190	Unspecified acquired deformity of unspecified limb	Arthritis Joint Other	Nociceptive Pain
M2192	Unspecified acquired deformity of upper arm	Arthritis Joint Upper Limb	Nociceptive Pain
M21921	Unspecified acquired deformity of right upper arm	Arthritis Joint Upper Limb	Nociceptive Pain
M21922	Unspecified acquired deformity of left upper arm	Arthritis Joint Upper Limb	Nociceptive Pain
M21929	Unspecified acquired deformity of unspecified upper arm	Arthritis Joint Upper Limb	Nociceptive Pain
M2193	Unspecified acquired deformity of forearm	Arthritis Joint Upper Limb	Nociceptive Pain
M21931	Unspecified acquired deformity of right forearm	Arthritis Joint Upper Limb	Nociceptive Pain
M21932	Unspecified acquired deformity of left forearm	Arthritis Joint Upper Limb	Nociceptive Pain
M21939	Unspecified acquired deformity of unspecified forearm	Arthritis Joint Upper Limb	Nociceptive Pain
M2194	Unspecified acquired deformity of hand	Arthritis Joint Upper Limb	Nociceptive Pain
M21941	Unspecified acquired deformity of hand, right hand	Arthritis Joint Upper Limb	Nociceptive Pain
M21942	Unspecified acquired deformity of hand, left hand	Arthritis Joint Upper Limb	Nociceptive Pain
M21949	Unspecified acquired deformity of hand, unspecified hand	Arthritis Joint Upper Limb	Nociceptive Pain
M2195	Unspecified acquired deformity of thigh	Arthritis Joint Lower Limb	Nociceptive Pain
M21951	Unspecified acquired deformity of right thigh	Arthritis Joint Lower Limb	Nociceptive Pain
M21952	Unspecified acquired deformity of left thigh	Arthritis Joint Lower Limb	Nociceptive Pain
M21959	Unspecified acquired deformity of unspecified thigh	Arthritis Joint Lower Limb	Nociceptive Pain
M2196	Unspecified acquired deformity of lower leg	Arthritis Joint Lower Limb	Nociceptive Pain
M21961	Unspecified acquired deformity of right lower leg	Arthritis Joint Lower Limb	Nociceptive Pain
M21962	Unspecified acquired deformity of left lower leg	Arthritis Joint Lower Limb	Nociceptive Pain
M21969	Unspecified acquired deformity of unspecified lower leg	Arthritis Joint Lower Limb	Nociceptive Pain
M22	Disorder of patella	Arthritis Joint Lower Limb	Nociceptive Pain
M220	Recurrent dislocation of patella	Arthritis Joint Lower Limb	Nociceptive Pain
M2200	Recurrent dislocation of patella, unspecified knee	Arthritis Joint Lower Limb	Nociceptive Pain
M2201	Recurrent dislocation of patella, right knee	Arthritis Joint Lower Limb	Nociceptive Pain
M2202	Recurrent dislocation of patella, left knee	Arthritis Joint Lower Limb	Nociceptive Pain
M221	Recurrent subluxation of patella	Arthritis Joint Lower Limb	Nociceptive Pain
M2210	Recurrent subluxation of patella, unspecified knee	Arthritis Joint Lower Limb	Nociceptive Pain
M2211	Recurrent subluxation of patella, right knee	Arthritis Joint Lower Limb	Nociceptive Pain

© 2024 Peterson MD et al. *JAMA Neurology.*

M2212	Recurrent subluxation of patella, left knee	Arthritis Joint Lower Limb	Nociceptive Pain
M222	Patellofemoral disorders	Arthritis Joint Lower Limb	Nociceptive Pain
M222X	Patellofemoral disorders	Arthritis Joint Lower Limb	Nociceptive Pain
M222X1	Patellofemoral disorders, right knee	Arthritis Joint Lower Limb	Nociceptive Pain
M222X2	Patellofemoral disorders, left knee	Arthritis Joint Lower Limb	Nociceptive Pain
M222X9	Patellofemoral disorders, unspecified knee	Arthritis Joint Lower Limb	Nociceptive Pain
M223	Other derangements of patella	Arthritis Joint Lower Limb	Nociceptive Pain
M223X	Other derangements of patella	Arthritis Joint Lower Limb	Nociceptive Pain
M223X1	Other derangements of patella, right knee	Arthritis Joint Lower Limb	Nociceptive Pain
M223X2	Other derangements of patella, left knee	Arthritis Joint Lower Limb	Nociceptive Pain
M223X9	Other derangements of patella, unspecified knee	Arthritis Joint Lower Limb	Nociceptive Pain
M224	Chondromalacia patellae	Arthritis Joint Lower Limb	Nociceptive Pain
M2240	Chondromalacia patellae, unspecified knee	Arthritis Joint Lower Limb	Nociceptive Pain
M2241	Chondromalacia patellae, right knee	Arthritis Joint Lower Limb	Nociceptive Pain
M2242	Chondromalacia patellae, left knee	Arthritis Joint Lower Limb	Nociceptive Pain
M228	Other disorders of patella	Arthritis Joint Lower Limb	Nociceptive Pain
M228X	Other disorders of patella	Arthritis Joint Lower Limb	Nociceptive Pain
M228X1	Other disorders of patella, right knee	Arthritis Joint Lower Limb	Nociceptive Pain
M228X2	Other disorders of patella, left knee	Arthritis Joint Lower Limb	Nociceptive Pain
M228X9	Other disorders of patella, unspecified knee	Arthritis Joint Lower Limb	Nociceptive Pain
M229	Unspecified disorder of patella	Arthritis Joint Lower Limb	Nociceptive Pain
M2290	Unspecified disorder of patella, unspecified knee	Arthritis Joint Lower Limb	Nociceptive Pain
M2291	Unspecified disorder of patella, right knee	Arthritis Joint Lower Limb	Nociceptive Pain
M2292	Unspecified disorder of patella, left knee	Arthritis Joint Lower Limb	Nociceptive Pain
M23	Internal derangement of knee	Arthritis Joint Lower Limb	Nociceptive Pain
M230	Cystic meniscus	Arthritis Joint Lower Limb	Nociceptive Pain
M2300	Cystic meniscus, unspecified meniscus	Arthritis Joint Lower Limb	Nociceptive Pain
M23000	Cystic meniscus, unspecified lateral meniscus, right knee	Arthritis Joint Lower Limb	Nociceptive Pain
M23001	Cystic meniscus, unspecified lateral meniscus, left knee	Arthritis Joint Lower Limb	Nociceptive Pain
M23002	Cystic meniscus, unsp lateral meniscus, unspecified knee	Arthritis Joint Lower Limb	Nociceptive Pain
M23003	Cystic meniscus, unspecified medial meniscus, right knee	Arthritis Joint Lower Limb	Nociceptive Pain
M23004	Cystic meniscus, unspecified medial meniscus, left knee	Arthritis Joint Lower Limb	Nociceptive Pain
M23005	Cystic meniscus, unsp medial meniscus, unspecified knee	Arthritis Joint Lower Limb	Nociceptive Pain
M23006	Cystic meniscus, unspecified meniscus, right knee	Arthritis Joint Lower Limb	Nociceptive Pain
M23007	Cystic meniscus, unspecified meniscus, left knee	Arthritis Joint Lower Limb	Nociceptive Pain
M23009	Cystic meniscus, unspecified meniscus, unspecified knee	Arthritis Joint Lower Limb	Nociceptive Pain
M2301	Cystic meniscus, anterior horn of medial meniscus	Arthritis Joint Lower Limb	Nociceptive Pain

© 2024 Peterson MD et al. *JAMA Neurology.*

M23011	Cystic meniscus, anterior horn of medial meniscus, r knee	Arthritis Joint Lower Limb	Nociceptive Pain
M23012	Cystic meniscus, anterior horn of medial meniscus, left knee	Arthritis Joint Lower Limb	Nociceptive Pain
M23019	Cystic meniscus, anterior horn of medial meniscus, unsp knee	Arthritis Joint Lower Limb	Nociceptive Pain
M2302	Cystic meniscus, posterior horn of medial meniscus	Arthritis Joint Lower Limb	Nociceptive Pain
M23021	Cystic meniscus, posterior horn of medial meniscus, r knee	Arthritis Joint Lower Limb	Nociceptive Pain
M23022	Cystic meniscus, posterior horn of medial meniscus, l knee	Arthritis Joint Lower Limb	Nociceptive Pain
M23029	Cystic meniscus, post horn of medial meniscus, unsp knee	Arthritis Joint Lower Limb	Nociceptive Pain
M2303	Cystic meniscus, other medial meniscus	Arthritis Joint Lower Limb	Nociceptive Pain
M23031	Cystic meniscus, other medial meniscus, right knee	Arthritis Joint Lower Limb	Nociceptive Pain
M23032	Cystic meniscus, other medial meniscus, left knee	Arthritis Joint Lower Limb	Nociceptive Pain
M23039	Cystic meniscus, other medial meniscus, unspecified knee	Arthritis Joint Lower Limb	Nociceptive Pain
M2304	Cystic meniscus, anterior horn of lateral meniscus	Arthritis Joint Lower Limb	Nociceptive Pain
M23041	Cystic meniscus, anterior horn of lat mensc, right knee	Arthritis Joint Lower Limb	Nociceptive Pain
M23042	Cystic meniscus, anterior horn of lat mensc, left knee	Arthritis Joint Lower Limb	Nociceptive Pain
M23049	Cystic meniscus, anterior horn of lat mensc, unsp knee	Arthritis Joint Lower Limb	Nociceptive Pain
M2305	Cystic meniscus, posterior horn of lateral meniscus	Arthritis Joint Lower Limb	Nociceptive Pain
M23051	Cystic meniscus, posterior horn of lat mensc, right knee	Arthritis Joint Lower Limb	Nociceptive Pain
M23052	Cystic meniscus, posterior horn of lat mensc, left knee	Arthritis Joint Lower Limb	Nociceptive Pain
M23059	Cystic meniscus, posterior horn of lat mensc, unsp knee	Arthritis Joint Lower Limb	Nociceptive Pain
M2306	Cystic meniscus, other lateral meniscus	Arthritis Joint Lower Limb	Nociceptive Pain
M23061	Cystic meniscus, other lateral meniscus, right knee	Arthritis Joint Lower Limb	Nociceptive Pain
M23062	Cystic meniscus, other lateral meniscus, left knee	Arthritis Joint Lower Limb	Nociceptive Pain
M23069	Cystic meniscus, other lateral meniscus, unspecified knee	Arthritis Joint Lower Limb	Nociceptive Pain
M232	Derangement of meniscus due to old tear or injury	Arthritis Joint Lower Limb	Nociceptive Pain
M2320	Derangement of unsp meniscus due to old tear or injury	Arthritis Joint Lower Limb	Nociceptive Pain
M23200	Derang of unsp lat mensc due to old tear/inj, right knee	Arthritis Joint Lower Limb	Nociceptive Pain
M23201	Derangement of unsp lat mensc due to old tear/inj, left knee	Arthritis Joint Lower Limb	Nociceptive Pain
M23202	Derangement of unsp lat mensc due to old tear/inj, unsp knee	Arthritis Joint Lower Limb	Nociceptive Pain
M23203	Derang of unsp medial meniscus due to old tear/inj, r knee	Arthritis Joint Lower Limb	Nociceptive Pain
M23204	Derang of unsp medial meniscus due to old tear/inj, l knee	Arthritis Joint Lower Limb	Nociceptive Pain
M23205	Derang of unsp medial mensc due to old tear/inj, unsp knee	Arthritis Joint Lower Limb	Nociceptive Pain
M23206	Derangement of unsp meniscus due to old tear/inj, right knee	Arthritis Joint Lower Limb	Nociceptive Pain
M23207	Derangement of unsp meniscus due to old tear/inj, left knee	Arthritis Joint Lower Limb	Nociceptive Pain
M23209	Derangement of unsp meniscus due to old tear/inj, unsp knee	Arthritis Joint Lower Limb	Nociceptive Pain
M2321	Derang of ant horn of medial meniscus due to old tear/inj	Arthritis Joint Lower Limb	Nociceptive Pain
M23211	Derang of ant horn of medial mensc d/t old tear/inj, r knee	Arthritis Joint Lower Limb	Nociceptive Pain
M23212	Derang of ant horn of medial mensc d/t old tear/inj, l knee	Arthritis Joint Lower Limb	Nociceptive Pain

© 2024 Peterson MD et al. *JAMA Neurology.*

M23219	Derang of ant horn of med mensc d/t old tear/inj, unsp knee	Arthritis Joint Lower Limb	Nociceptive Pain
M2322	Derang of post horn of medial meniscus due to old tear/inj	Arthritis Joint Lower Limb	Nociceptive Pain
M23221	Derang of post horn of medial mensc d/t old tear/inj, r knee	Arthritis Joint Lower Limb	Nociceptive Pain
M23222	Derang of post horn of medial mensc d/t old tear/inj, l knee	Arthritis Joint Lower Limb	Nociceptive Pain
M23229	Derang of post horn of med mensc d/t old tear/inj, unsp knee	Arthritis Joint Lower Limb	Nociceptive Pain
M2323	Derangement of oth medial meniscus due to old tear or injury	Arthritis Joint Lower Limb	Nociceptive Pain
M23231	Derang of medial meniscus due to old tear/inj, right knee	Arthritis Joint Lower Limb	Nociceptive Pain
M23232	Derang of medial meniscus due to old tear/inj, left knee	Arthritis Joint Lower Limb	Nociceptive Pain
M23239	Derang of medial meniscus due to old tear/inj, unsp knee	Arthritis Joint Lower Limb	Nociceptive Pain
M2324	Derang of anterior horn of lat mensc due to old tear/inj	Arthritis Joint Lower Limb	Nociceptive Pain
M23241	Derang of ant horn of lat mensc due to old tear/inj, r knee	Arthritis Joint Lower Limb	Nociceptive Pain
M23242	Derang of ant horn of lat mensc due to old tear/inj, l knee	Arthritis Joint Lower Limb	Nociceptive Pain
M23249	Derang of ant horn of lat mensc d/t old tear/inj, unsp knee	Arthritis Joint Lower Limb	Nociceptive Pain
M2325	Derang of posterior horn of lat mensc due to old tear/inj	Arthritis Joint Lower Limb	Nociceptive Pain
M23251	Derang of post horn of lat mensc due to old tear/inj, r knee	Arthritis Joint Lower Limb	Nociceptive Pain
M23252	Derang of post horn of lat mensc due to old tear/inj, l knee	Arthritis Joint Lower Limb	Nociceptive Pain
M23259	Derang of post horn of lat mensc d/t old tear/inj, unsp knee	Arthritis Joint Lower Limb	Nociceptive Pain
M2326	Derangement of lateral meniscus due to old tear or injury	Arthritis Joint Lower Limb	Nociceptive Pain
M23261	Derangement of lat mensc due to old tear/inj, right knee	Arthritis Joint Lower Limb	Nociceptive Pain
M23262	Derangement of lat mensc due to old tear/inj, left knee	Arthritis Joint Lower Limb	Nociceptive Pain
M23269	Derangement of lat mensc due to old tear/inj, unsp knee	Arthritis Joint Lower Limb	Nociceptive Pain
M233	Other meniscus derangements	Arthritis Joint Lower Limb	Nociceptive Pain
M2330	Other meniscus derangements, unspecified meniscus	Arthritis Joint Lower Limb	Nociceptive Pain
M23300	Oth meniscus derangements, unsp lateral meniscus, right knee	Arthritis Joint Lower Limb	Nociceptive Pain
M23301	Oth meniscus derangements, unsp lateral meniscus, left knee	Arthritis Joint Lower Limb	Nociceptive Pain
M23302	Oth meniscus derangements, unsp lateral meniscus, unsp knee	Arthritis Joint Lower Limb	Nociceptive Pain
M23303	Oth meniscus derangements, unsp medial meniscus, right knee	Arthritis Joint Lower Limb	Nociceptive Pain
M23304	Other meniscus derangements, unsp medial meniscus, left knee	Arthritis Joint Lower Limb	Nociceptive Pain
M23305	Other meniscus derangements, unsp medial meniscus, unsp knee	Arthritis Joint Lower Limb	Nociceptive Pain
M23306	Other meniscus derangements, unsp meniscus, right knee	Arthritis Joint Lower Limb	Nociceptive Pain
M23307	Other meniscus derangements, unspecified meniscus, left knee	Arthritis Joint Lower Limb	Nociceptive Pain
M23309	Other meniscus derangements, unsp meniscus, unspecified knee	Arthritis Joint Lower Limb	Nociceptive Pain
M2331	Oth meniscus derangements, anterior horn of medial meniscus	Arthritis Joint Lower Limb	Nociceptive Pain
M23311	Oth meniscus derang, ant horn of medial meniscus, r knee	Arthritis Joint Lower Limb	Nociceptive Pain
M23312	Oth meniscus derang, ant horn of medial meniscus, l knee	Arthritis Joint Lower Limb	Nociceptive Pain

© 2024 Peterson MD et al. *JAMA Neurology.*

M23319	Oth meniscus derang, ant horn of medial meniscus, unsp knee	Arthritis Joint Lower Limb	Nociceptive Pain
M2332	Oth meniscus derangements, posterior horn of medial meniscus	Arthritis Joint Lower Limb	Nociceptive Pain
M23321	Oth meniscus derang, post horn of medial meniscus, r knee	Arthritis Joint Lower Limb	Nociceptive Pain
M23322	Oth meniscus derang, post horn of medial meniscus, l knee	Arthritis Joint Lower Limb	Nociceptive Pain
M23329	Oth meniscus derang, post horn of medial meniscus, unsp knee	Arthritis Joint Lower Limb	Nociceptive Pain
M2333	Other meniscus derangements, other medial meniscus	Arthritis Joint Lower Limb	Nociceptive Pain
M23331	Oth meniscus derangements, other medial meniscus, right knee	Arthritis Joint Lower Limb	Nociceptive Pain
M23332	Oth meniscus derangements, other medial meniscus, left knee	Arthritis Joint Lower Limb	Nociceptive Pain
M23339	Oth meniscus derangements, other medial meniscus, unsp knee	Arthritis Joint Lower Limb	Nociceptive Pain
M2334	Oth meniscus derangements, anterior horn of lateral meniscus	Arthritis Joint Lower Limb	Nociceptive Pain
M23341	Oth meniscus derang, anterior horn of lat mensc, right knee	Arthritis Joint Lower Limb	Nociceptive Pain
M23342	Oth meniscus derang, anterior horn of lat mensc, left knee	Arthritis Joint Lower Limb	Nociceptive Pain
M23349	Oth meniscus derang, anterior horn of lat mensc, unsp knee	Arthritis Joint Lower Limb	Nociceptive Pain
M2335	Oth meniscus derangements, posterior horn of lat mensc	Arthritis Joint Lower Limb	Nociceptive Pain
M23351	Oth meniscus derang, posterior horn of lat mensc, right knee	Arthritis Joint Lower Limb	Nociceptive Pain
M23352	Oth meniscus derang, posterior horn of lat mensc, left knee	Arthritis Joint Lower Limb	Nociceptive Pain
M23359	Oth meniscus derang, posterior horn of lat mensc, unsp knee	Arthritis Joint Lower Limb	Nociceptive Pain
M2336	Other meniscus derangements, other lateral meniscus	Arthritis Joint Lower Limb	Nociceptive Pain
M23361	Oth meniscus derangements, oth lateral meniscus, right knee	Arthritis Joint Lower Limb	Nociceptive Pain
M23362	Oth meniscus derangements, other lateral meniscus, left knee	Arthritis Joint Lower Limb	Nociceptive Pain
M23369	Oth meniscus derangements, other lateral meniscus, unsp knee	Arthritis Joint Lower Limb	Nociceptive Pain
M234	Loose body in knee	Arthritis Joint Lower Limb	Nociceptive Pain
M2340	Loose body in knee, unspecified knee	Arthritis Joint Lower Limb	Nociceptive Pain
M2341	Loose body in knee, right knee	Arthritis Joint Lower Limb	Nociceptive Pain
M2342	Loose body in knee, left knee	Arthritis Joint Lower Limb	Nociceptive Pain
M235	Chronic instability of knee	Arthritis Joint Lower Limb	Nociceptive Pain
M2350	Chronic instability of knee, unspecified knee	Arthritis Joint Lower Limb	Nociceptive Pain
M2351	Chronic instability of knee, right knee	Arthritis Joint Lower Limb	Nociceptive Pain
M2352	Chronic instability of knee, left knee	Arthritis Joint Lower Limb	Nociceptive Pain
M236	Other spontaneous disruption of ligament(s) of knee	Arthritis Joint Lower Limb	Nociceptive Pain
M2360	Other spontaneous disruption of unspecified ligament of knee	Arthritis Joint Lower Limb	Nociceptive Pain
M23601	Other spontaneous disruption of unsp ligament of right knee	Arthritis Joint Lower Limb	Nociceptive Pain
M23602	Other spontaneous disruption of unsp ligament of left knee	Arthritis Joint Lower Limb	Nociceptive Pain
M23609	Other spontaneous disruption of unsp ligament of unsp knee	Arthritis Joint Lower Limb	Nociceptive Pain

© 2024 Peterson MD et al. *JAMA Neurology.*

M2361	Oth spon disruption of anterior cruciate ligament of knee	Arthritis Joint Lower Limb	Nociceptive Pain
M23611	Oth spon disrupt of anterior cruciate ligament of right knee	Arthritis Joint Lower Limb	Nociceptive Pain
M23612	Oth spon disrupt of anterior cruciate ligament of left knee	Arthritis Joint Lower Limb	Nociceptive Pain
M23619	Oth spon disrupt of anterior cruciate ligament of unsp knee	Arthritis Joint Lower Limb	Nociceptive Pain
M2362	Oth spon disruption of posterior cruciate ligament of knee	Arthritis Joint Lower Limb	Nociceptive Pain
M23621	Oth spon disrupt of posterior cruciate ligament of r knee	Arthritis Joint Lower Limb	Nociceptive Pain
M23622	Oth spon disrupt of posterior cruciate ligament of left knee	Arthritis Joint Lower Limb	Nociceptive Pain
M23629	Oth spon disrupt of posterior cruciate ligament of unsp knee	Arthritis Joint Lower Limb	Nociceptive Pain
M2363	Oth spon disruption of medial collateral ligament of knee	Arthritis Joint Lower Limb	Nociceptive Pain
M23631	Oth spon disruption of medial collat ligament of right knee	Arthritis Joint Lower Limb	Nociceptive Pain
M23632	Oth spon disruption of medial collat ligament of left knee	Arthritis Joint Lower Limb	Nociceptive Pain
M23639	Oth spon disruption of medial collat ligament of unsp knee	Arthritis Joint Lower Limb	Nociceptive Pain
M2364	Oth spon disruption of lateral collateral ligament of knee	Arthritis Joint Lower Limb	Nociceptive Pain
M23641	Oth spon disruption of lateral collat ligament of right knee	Arthritis Joint Lower Limb	Nociceptive Pain
M23642	Oth spon disruption of lateral collat ligament of left knee	Arthritis Joint Lower Limb	Nociceptive Pain
M23649	Oth spon disruption of lateral collat ligament of unsp knee	Arthritis Joint Lower Limb	Nociceptive Pain
M2367	Other spontaneous disruption of capsular ligament of knee	Arthritis Joint Lower Limb	Nociceptive Pain
M23671	Oth spon disruption of capsular ligament of right knee	Arthritis Joint Lower Limb	Nociceptive Pain
M23672	Oth spontaneous disruption of capsular ligament of left knee	Arthritis Joint Lower Limb	Nociceptive Pain
M23679	Oth spontaneous disruption of capsular ligament of unsp knee	Arthritis Joint Lower Limb	Nociceptive Pain
M238	Other internal derangements of knee	Arthritis Joint Lower Limb	Nociceptive Pain
M238X	Other internal derangements of knee	Arthritis Joint Lower Limb	Nociceptive Pain
M238X1	Other internal derangements of right knee	Arthritis Joint Lower Limb	Nociceptive Pain
M238X2	Other internal derangements of left knee	Arthritis Joint Lower Limb	Nociceptive Pain
M238X9	Other internal derangements of unspecified knee	Arthritis Joint Lower Limb	Nociceptive Pain
M239	Unspecified internal derangement of knee	Arthritis Joint Lower Limb	Nociceptive Pain
M2390	Unspecified internal derangement of unspecified knee	Arthritis Joint Lower Limb	Nociceptive Pain
M2391	Unspecified internal derangement of right knee	Arthritis Joint Lower Limb	Nociceptive Pain
M2392	Unspecified internal derangement of left knee	Arthritis Joint Lower Limb	Nociceptive Pain
M24	Other specific joint derangements	Arthritis Joint Other	Nociceptive Pain
M240	Loose body in joint	Arthritis Joint Other	Nociceptive Pain
M2400	Loose body in unspecified joint	Arthritis Joint Other	Nociceptive Pain
M2401	Loose body in shoulder	Arthritis Joint Upper Limb	Nociceptive Pain
M24011	Loose body in right shoulder	Arthritis Joint Upper Limb	Nociceptive Pain
M24012	Loose body in left shoulder	Arthritis Joint Upper Limb	Nociceptive Pain
M24019	Loose body in unspecified shoulder	Arthritis Joint Upper Limb	Nociceptive Pain
M2402	Loose body in elbow	Arthritis Joint Upper Limb	Nociceptive Pain

© 2024 Peterson MD et al. *JAMA Neurology.*

M24021	Loose body in right elbow	Arthritis Joint Upper Limb	Nociceptive Pain
M24022	Loose body in left elbow	Arthritis Joint Upper Limb	Nociceptive Pain
M24029	Loose body in unspecified elbow	Arthritis Joint Upper Limb	Nociceptive Pain
M2403	Loose body in wrist	Arthritis Joint Upper Limb	Nociceptive Pain
M24031	Loose body in right wrist	Arthritis Joint Upper Limb	Nociceptive Pain
M24032	Loose body in left wrist	Arthritis Joint Upper Limb	Nociceptive Pain
M24039	Loose body in unspecified wrist	Arthritis Joint Upper Limb	Nociceptive Pain
M2404	Loose body in finger joints	Arthritis Joint Upper Limb	Nociceptive Pain
M24041	Loose body in right finger joint(s)	Arthritis Joint Upper Limb	Nociceptive Pain
M24042	Loose body in left finger joint(s)	Arthritis Joint Upper Limb	Nociceptive Pain
M24049	Loose body in unspecified finger joint(s)	Arthritis Joint Upper Limb	Nociceptive Pain
M2405	Loose body in hip	Arthritis Joint Spine and Hips	Nociceptive Pain
M24051	Loose body in right hip	Arthritis Joint Spine and Hips	Nociceptive Pain
M24052	Loose body in left hip	Arthritis Joint Spine and Hips	Nociceptive Pain
M24059	Loose body in unspecified hip	Arthritis Joint Spine and Hips	Nociceptive Pain
M2407	Loose body in ankle and toe joints	Arthritis Joint Lower Limb	Nociceptive Pain
M24071	Loose body in right ankle	Arthritis Joint Lower Limb	Nociceptive Pain
M24072	Loose body in left ankle	Arthritis Joint Lower Limb	Nociceptive Pain
M24073	Loose body in unspecified ankle	Arthritis Joint Lower Limb	Nociceptive Pain
M24074	Loose body in right toe joint(s)	Arthritis Joint Lower Limb	Nociceptive Pain
M24075	Loose body in left toe joint(s)	Arthritis Joint Lower Limb	Nociceptive Pain
M24076	Loose body in unspecified toe joints	Arthritis Joint Lower Limb	Nociceptive Pain
M2408	Loose body, other site	Arthritis Joint Other	Nociceptive Pain
M241	Other articular cartilage disorders	Arthritis Joint Other	Nociceptive Pain
M2410	Other articular cartilage disorders, unspecified site	Arthritis Joint Other	Nociceptive Pain
M2411	Other articular cartilage disorders, shoulder	Arthritis Joint Upper Limb	Nociceptive Pain
M24111	Other articular cartilage disorders, right shoulder	Arthritis Joint Upper Limb	Nociceptive Pain
M24112	Other articular cartilage disorders, left shoulder	Arthritis Joint Upper Limb	Nociceptive Pain
M24119	Other articular cartilage disorders, unspecified shoulder	Arthritis Joint Upper Limb	Nociceptive Pain
M2412	Other articular cartilage disorders, elbow	Arthritis Joint Upper Limb	Nociceptive Pain
M24121	Other articular cartilage disorders, right elbow	Arthritis Joint Upper Limb	Nociceptive Pain
M24122	Other articular cartilage disorders, left elbow	Arthritis Joint Upper Limb	Nociceptive Pain
M24129	Other articular cartilage disorders, unspecified elbow	Arthritis Joint Upper Limb	Nociceptive Pain
M2413	Other articular cartilage disorders, wrist	Arthritis Joint Upper Limb	Nociceptive Pain
M24131	Other articular cartilage disorders, right wrist	Arthritis Joint Upper Limb	Nociceptive Pain
M24132	Other articular cartilage disorders, left wrist	Arthritis Joint Upper Limb	Nociceptive Pain
M24139	Other articular cartilage disorders, unspecified wrist	Arthritis Joint Upper Limb	Nociceptive Pain

© 2024 Peterson MD et al. *JAMA Neurology.*

M2414	Other articular cartilage disorders, hand	Arthritis Joint Upper Limb	Nociceptive Pain
M24141	Other articular cartilage disorders, right hand	Arthritis Joint Upper Limb	Nociceptive Pain
M24142	Other articular cartilage disorders, left hand	Arthritis Joint Upper Limb	Nociceptive Pain
M24149	Other articular cartilage disorders, unspecified hand	Arthritis Joint Upper Limb	Nociceptive Pain
M2415	Other articular cartilage disorders, hip	Arthritis Joint Spine and Hips	Nociceptive Pain
M24151	Other articular cartilage disorders, right hip	Arthritis Joint Spine and Hips	Nociceptive Pain
M24152	Other articular cartilage disorders, left hip	Arthritis Joint Spine and Hips	Nociceptive Pain
M24159	Other articular cartilage disorders, unspecified hip	Arthritis Joint Spine and Hips	Nociceptive Pain
M2417	Other articular cartilage disorders, ankle and foot	Arthritis Joint Lower Limb	Nociceptive Pain
M24171	Other articular cartilage disorders, right ankle	Arthritis Joint Lower Limb	Nociceptive Pain
M24172	Other articular cartilage disorders, left ankle	Arthritis Joint Lower Limb	Nociceptive Pain
M24173	Other articular cartilage disorders, unspecified ankle	Arthritis Joint Lower Limb	Nociceptive Pain
M24174	Other articular cartilage disorders, right foot	Arthritis Joint Lower Limb	Nociceptive Pain
M24175	Other articular cartilage disorders, left foot	Arthritis Joint Lower Limb	Nociceptive Pain
M24176	Other articular cartilage disorders, unspecified foot	Arthritis Joint Lower Limb	Nociceptive Pain
M2419	Other articular cartilage disorders, other specified site	Arthritis Joint Other	Nociceptive Pain
M242	Disorder of ligament	Arthritis Joint Other	Nociceptive Pain
M2420	Disorder of ligament, unspecified site	Arthritis Joint Other	Nociceptive Pain
M2421	Disorder of ligament, shoulder	Arthritis Joint Upper Limb	Nociceptive Pain
M24211	Disorder of ligament, right shoulder	Arthritis Joint Upper Limb	Nociceptive Pain
M24212	Disorder of ligament, left shoulder	Arthritis Joint Upper Limb	Nociceptive Pain
M24219	Disorder of ligament, unspecified shoulder	Arthritis Joint Upper Limb	Nociceptive Pain
M2422	Disorder of ligament, elbow	Arthritis Joint Upper Limb	Nociceptive Pain
M24221	Disorder of ligament, right elbow	Arthritis Joint Upper Limb	Nociceptive Pain
M24222	Disorder of ligament, left elbow	Arthritis Joint Upper Limb	Nociceptive Pain
M24229	Disorder of ligament, unspecified elbow	Arthritis Joint Upper Limb	Nociceptive Pain
M2423	Disorder of ligament, wrist	Arthritis Joint Upper Limb	Nociceptive Pain
M24231	Disorder of ligament, right wrist	Arthritis Joint Upper Limb	Nociceptive Pain
M24232	Disorder of ligament, left wrist	Arthritis Joint Upper Limb	Nociceptive Pain
M24239	Disorder of ligament, unspecified wrist	Arthritis Joint Upper Limb	Nociceptive Pain
M2424	Disorder of ligament, hand	Arthritis Joint Upper Limb	Nociceptive Pain
M24241	Disorder of ligament, right hand	Arthritis Joint Upper Limb	Nociceptive Pain
M24242	Disorder of ligament, left hand	Arthritis Joint Upper Limb	Nociceptive Pain
M24249	Disorder of ligament, unspecified hand	Arthritis Joint Upper Limb	Nociceptive Pain
M2425	Disorder of ligament, hip	Arthritis Joint Spine and Hips	Nociceptive Pain
M24251	Disorder of ligament, right hip	Arthritis Joint Spine and Hips	Nociceptive Pain
M24252	Disorder of ligament, left hip	Arthritis Joint Spine and Hips	Nociceptive Pain

© 2024 Peterson MD et al. *JAMA Neurology.*

M24259	Disorder of ligament, unspecified hip	Arthritis Joint Spine and Hips	Nociceptive Pain
M2427	Disorder of ligament, ankle and foot	Arthritis Joint Lower Limb	Nociceptive Pain
M24271	Disorder of ligament, right ankle	Arthritis Joint Lower Limb	Nociceptive Pain
M24272	Disorder of ligament, left ankle	Arthritis Joint Lower Limb	Nociceptive Pain
M24273	Disorder of ligament, unspecified ankle	Arthritis Joint Lower Limb	Nociceptive Pain
M24274	Disorder of ligament, right foot	Arthritis Joint Lower Limb	Nociceptive Pain
M24275	Disorder of ligament, left foot	Arthritis Joint Lower Limb	Nociceptive Pain
M24276	Disorder of ligament, unspecified foot	Arthritis Joint Lower Limb	Nociceptive Pain
M2428	Disorder of ligament, vertebrae	Arthritis Joint Spine and Hips	Nociceptive Pain
M2429	Disorder of ligament, other specified site	Arthritis Joint Other	Nociceptive Pain
M243	Pathological dislocation of joint, not elsewhere classified	Arthritis Joint Other	Nociceptive Pain
M2430	Pathological dislocation of unsp joint, NEC	Arthritis Joint Other	Nociceptive Pain
M2431	Pathological dislocation of shoulder, NEC	Arthritis Joint Upper Limb	Nociceptive Pain
M24311	Pathological dislocation of right shoulder, NEC	Arthritis Joint Upper Limb	Nociceptive Pain
M24312	Pathological dislocation of left shoulder, NEC	Arthritis Joint Upper Limb	Nociceptive Pain
M24319	Pathological dislocation of unsp shoulder, NEC	Arthritis Joint Upper Limb	Nociceptive Pain
M2432	Pathological dislocation of elbow, not elsewhere classified	Arthritis Joint Upper Limb	Nociceptive Pain
M24321	Pathological dislocation of right elbow, NEC	Arthritis Joint Upper Limb	Nociceptive Pain
M24322	Pathological dislocation of left elbow, NEC	Arthritis Joint Upper Limb	Nociceptive Pain
M24329	Pathological dislocation of unsp elbow, NEC	Arthritis Joint Upper Limb	Nociceptive Pain
M2433	Pathological dislocation of wrist, not elsewhere classified	Arthritis Joint Upper Limb	Nociceptive Pain
M24331	Pathological dislocation of right wrist, NEC	Arthritis Joint Upper Limb	Nociceptive Pain
M24332	Pathological dislocation of left wrist, NEC	Arthritis Joint Upper Limb	Nociceptive Pain
M24339	Pathological dislocation of unsp wrist, NEC	Arthritis Joint Upper Limb	Nociceptive Pain
M2434	Pathological dislocation of hand, not elsewhere classified	Arthritis Joint Upper Limb	Nociceptive Pain
M24341	Pathological dislocation of right hand, NEC	Arthritis Joint Upper Limb	Nociceptive Pain
M24342	Pathological dislocation of left hand, NEC	Arthritis Joint Upper Limb	Nociceptive Pain
M24349	Pathological dislocation of unsp hand, NEC	Arthritis Joint Upper Limb	Nociceptive Pain
M2435	Pathological dislocation of hip, not elsewhere classified	Arthritis Joint Spine and Hips	Nociceptive Pain
M24351	Pathological dislocation of right hip, NEC	Arthritis Joint Spine and Hips	Nociceptive Pain
M24352	Pathological dislocation of left hip, NEC	Arthritis Joint Spine and Hips	Nociceptive Pain
M24359	Pathological dislocation of unsp hip, NEC	Arthritis Joint Spine and Hips	Nociceptive Pain
M2436	Pathological dislocation of knee, not elsewhere classified	Arthritis Joint Lower Limb	Nociceptive Pain
M24361	Pathological dislocation of right knee, NEC	Arthritis Joint Lower Limb	Nociceptive Pain
M24362	Pathological dislocation of left knee, NEC	Arthritis Joint Lower Limb	Nociceptive Pain
M24369	Pathological dislocation of unsp knee, NEC	Arthritis Joint Lower Limb	Nociceptive Pain
M2437	Pathological dislocation of ankle and foot, NEC	Arthritis Joint Lower Limb	Nociceptive Pain

© 2024 Peterson MD et al. *JAMA Neurology.*

M24371	Pathological dislocation of right ankle, NEC	Arthritis Joint Lower Limb	Nociceptive Pain
M24372	Pathological dislocation of left ankle, NEC	Arthritis Joint Lower Limb	Nociceptive Pain
M24373	Pathological dislocation of unsp ankle, NEC	Arthritis Joint Lower Limb	Nociceptive Pain
M24374	Pathological dislocation of right foot, NEC	Arthritis Joint Lower Limb	Nociceptive Pain
M24375	Pathological dislocation of left foot, NEC	Arthritis Joint Lower Limb	Nociceptive Pain
M24376	Pathological dislocation of unsp foot, NEC	Arthritis Joint Lower Limb	Nociceptive Pain
M2439	Pathological dislocation of other specified joint, NEC	Arthritis Joint Other	Nociceptive Pain
M244	Recurrent dislocation of joint	Arthritis Joint Other	Nociceptive Pain
M2440	Recurrent dislocation, unspecified joint	Arthritis Joint Other	Nociceptive Pain
M2441	Recurrent dislocation, shoulder	Arthritis Joint Upper Limb	Nociceptive Pain
M24411	Recurrent dislocation, right shoulder	Arthritis Joint Upper Limb	Nociceptive Pain
M24412	Recurrent dislocation, left shoulder	Arthritis Joint Upper Limb	Nociceptive Pain
M24419	Recurrent dislocation, unspecified shoulder	Arthritis Joint Upper Limb	Nociceptive Pain
M2442	Recurrent dislocation, elbow	Arthritis Joint Upper Limb	Nociceptive Pain
M24421	Recurrent dislocation, right elbow	Arthritis Joint Upper Limb	Nociceptive Pain
M24422	Recurrent dislocation, left elbow	Arthritis Joint Upper Limb	Nociceptive Pain
M24429	Recurrent dislocation, unspecified elbow	Arthritis Joint Upper Limb	Nociceptive Pain
M2443	Recurrent dislocation, wrist	Arthritis Joint Upper Limb	Nociceptive Pain
M24431	Recurrent dislocation, right wrist	Arthritis Joint Upper Limb	Nociceptive Pain
M24432	Recurrent dislocation, left wrist	Arthritis Joint Upper Limb	Nociceptive Pain
M24439	Recurrent dislocation, unspecified wrist	Arthritis Joint Upper Limb	Nociceptive Pain
M2444	Recurrent dislocation, hand and finger(s)	Arthritis Joint Upper Limb	Nociceptive Pain
M24441	Recurrent dislocation, right hand	Arthritis Joint Upper Limb	Nociceptive Pain
M24442	Recurrent dislocation, left hand	Arthritis Joint Upper Limb	Nociceptive Pain
M24443	Recurrent dislocation, unspecified hand	Arthritis Joint Upper Limb	Nociceptive Pain
M24444	Recurrent dislocation, right finger	Arthritis Joint Upper Limb	Nociceptive Pain
M24445	Recurrent dislocation, left finger	Arthritis Joint Upper Limb	Nociceptive Pain
M24446	Recurrent dislocation, unspecified finger	Arthritis Joint Upper Limb	Nociceptive Pain
M2445	Recurrent dislocation, hip	Arthritis Joint Spine and Hips	Nociceptive Pain
M24451	Recurrent dislocation, right hip	Arthritis Joint Spine and Hips	Nociceptive Pain
M24452	Recurrent dislocation, left hip	Arthritis Joint Spine and Hips	Nociceptive Pain
M24459	Recurrent dislocation, unspecified hip	Arthritis Joint Spine and Hips	Nociceptive Pain
M2446	Recurrent dislocation, knee	Arthritis Joint Lower Limb	Nociceptive Pain
M24461	Recurrent dislocation, right knee	Arthritis Joint Lower Limb	Nociceptive Pain
M24462	Recurrent dislocation, left knee	Arthritis Joint Lower Limb	Nociceptive Pain
M24469	Recurrent dislocation, unspecified knee	Arthritis Joint Lower Limb	Nociceptive Pain
M2447	Recurrent dislocation, ankle, foot and toes	Arthritis Joint Lower Limb	Nociceptive Pain

© 2024 Peterson MD et al. *JAMA Neurology.*

M24471	Recurrent dislocation, right ankle	Arthritis Joint Lower Limb	Nociceptive Pain
M24472	Recurrent dislocation, left ankle	Arthritis Joint Lower Limb	Nociceptive Pain
M24473	Recurrent dislocation, unspecified ankle	Arthritis Joint Lower Limb	Nociceptive Pain
M24474	Recurrent dislocation, right foot	Arthritis Joint Lower Limb	Nociceptive Pain
M24475	Recurrent dislocation, left foot	Arthritis Joint Lower Limb	Nociceptive Pain
M24476	Recurrent dislocation, unspecified foot	Arthritis Joint Lower Limb	Nociceptive Pain
M24477	Recurrent dislocation, right toe(s)	Arthritis Joint Lower Limb	Nociceptive Pain
M24478	Recurrent dislocation, left toe(s)	Arthritis Joint Lower Limb	Nociceptive Pain
M24479	Recurrent dislocation, unspecified toe(s)	Arthritis Joint Lower Limb	Nociceptive Pain
M2449	Recurrent dislocation, other specified joint	Arthritis Joint Other	Nociceptive Pain
M245	Contracture of joint	Arthritis Joint Other	Nociceptive Pain
M2450	Contracture, unspecified joint	Arthritis Joint Other	Nociceptive Pain
M2451	Contracture, shoulder	Arthritis Joint Upper Limb	Nociceptive Pain
M24511	Contracture, right shoulder	Arthritis Joint Upper Limb	Nociceptive Pain
M24512	Contracture, left shoulder	Arthritis Joint Upper Limb	Nociceptive Pain
M24519	Contracture, unspecified shoulder	Arthritis Joint Upper Limb	Nociceptive Pain
M2452	Contracture, elbow	Arthritis Joint Upper Limb	Nociceptive Pain
M24521	Contracture, right elbow	Arthritis Joint Upper Limb	Nociceptive Pain
M24522	Contracture, left elbow	Arthritis Joint Upper Limb	Nociceptive Pain
M24529	Contracture, unspecified elbow	Arthritis Joint Upper Limb	Nociceptive Pain
M2453	Contracture, wrist	Arthritis Joint Upper Limb	Nociceptive Pain
M24531	Contracture, right wrist	Arthritis Joint Upper Limb	Nociceptive Pain
M24532	Contracture, left wrist	Arthritis Joint Upper Limb	Nociceptive Pain
M24539	Contracture, unspecified wrist	Arthritis Joint Upper Limb	Nociceptive Pain
M2454	Contracture, hand	Arthritis Joint Upper Limb	Nociceptive Pain
M24541	Contracture, right hand	Arthritis Joint Upper Limb	Nociceptive Pain
M24542	Contracture, left hand	Arthritis Joint Upper Limb	Nociceptive Pain
M24549	Contracture, unspecified hand	Arthritis Joint Upper Limb	Nociceptive Pain
M2455	Contracture, hip	Arthritis Joint Spine and Hips	Nociceptive Pain
M24551	Contracture, right hip	Arthritis Joint Spine and Hips	Nociceptive Pain
M24552	Contracture, left hip	Arthritis Joint Spine and Hips	Nociceptive Pain
M24559	Contracture, unspecified hip	Arthritis Joint Spine and Hips	Nociceptive Pain
M2456	Contracture, knee	Arthritis Joint Lower Limb	Nociceptive Pain
M24561	Contracture, right knee	Arthritis Joint Lower Limb	Nociceptive Pain
M24562	Contracture, left knee	Arthritis Joint Lower Limb	Nociceptive Pain
M24569	Contracture, unspecified knee	Arthritis Joint Lower Limb	Nociceptive Pain
M2457	Contracture, ankle and foot	Arthritis Joint Lower Limb	Nociceptive Pain

© 2024 Peterson MD et al. *JAMA Neurology.*

M24571	Contracture, right ankle	Arthritis Joint Lower Limb	Nociceptive Pain
M24572	Contracture, left ankle	Arthritis Joint Lower Limb	Nociceptive Pain
M24573	Contracture, unspecified ankle	Arthritis Joint Lower Limb	Nociceptive Pain
M24574	Contracture, right foot	Arthritis Joint Lower Limb	Nociceptive Pain
M24575	Contracture, left foot	Arthritis Joint Lower Limb	Nociceptive Pain
M24576	Contracture, unspecified foot	Arthritis Joint Lower Limb	Nociceptive Pain
M2459	Contracture, other specified joint	Arthritis Joint Other	Nociceptive Pain
M246	Ankylosis of joint	Arthritis Joint Other	Nociceptive Pain
M2460	Ankylosis, unspecified joint	Arthritis Joint Other	Nociceptive Pain
M2461	Ankylosis, shoulder	Arthritis Joint Upper Limb	Nociceptive Pain
M24611	Ankylosis, right shoulder	Arthritis Joint Upper Limb	Nociceptive Pain
M24612	Ankylosis, left shoulder	Arthritis Joint Upper Limb	Nociceptive Pain
M24619	Ankylosis, unspecified shoulder	Arthritis Joint Upper Limb	Nociceptive Pain
M2462	Ankylosis, elbow	Arthritis Joint Upper Limb	Nociceptive Pain
M24621	Ankylosis, right elbow	Arthritis Joint Upper Limb	Nociceptive Pain
M24622	Ankylosis, left elbow	Arthritis Joint Upper Limb	Nociceptive Pain
M24629	Ankylosis, unspecified elbow	Arthritis Joint Upper Limb	Nociceptive Pain
M2463	Ankylosis, wrist	Arthritis Joint Upper Limb	Nociceptive Pain
M24631	Ankylosis, right wrist	Arthritis Joint Upper Limb	Nociceptive Pain
M24632	Ankylosis, left wrist	Arthritis Joint Upper Limb	Nociceptive Pain
M24639	Ankylosis, unspecified wrist	Arthritis Joint Upper Limb	Nociceptive Pain
M2464	Ankylosis, hand	Arthritis Joint Upper Limb	Nociceptive Pain
M24641	Ankylosis, right hand	Arthritis Joint Upper Limb	Nociceptive Pain
M24642	Ankylosis, left hand	Arthritis Joint Upper Limb	Nociceptive Pain
M24649	Ankylosis, unspecified hand	Arthritis Joint Upper Limb	Nociceptive Pain
M2465	Ankylosis, hip	Arthritis Joint Spine and Hips	Nociceptive Pain
M24651	Ankylosis, right hip	Arthritis Joint Spine and Hips	Nociceptive Pain
M24652	Ankylosis, left hip	Arthritis Joint Spine and Hips	Nociceptive Pain
M24659	Ankylosis, unspecified hip	Arthritis Joint Spine and Hips	Nociceptive Pain
M2466	Ankylosis, knee	Arthritis Joint Lower Limb	Nociceptive Pain
M24661	Ankylosis, right knee	Arthritis Joint Lower Limb	Nociceptive Pain
M24662	Ankylosis, left knee	Arthritis Joint Lower Limb	Nociceptive Pain
M24669	Ankylosis, unspecified knee	Arthritis Joint Lower Limb	Nociceptive Pain
M2467	Ankylosis, ankle and foot	Arthritis Joint Lower Limb	Nociceptive Pain
M24671	Ankylosis, right ankle	Arthritis Joint Lower Limb	Nociceptive Pain
M24672	Ankylosis, left ankle	Arthritis Joint Lower Limb	Nociceptive Pain
M24673	Ankylosis, unspecified ankle	Arthritis Joint Lower Limb	Nociceptive Pain

© 2024 Peterson MD et al. *JAMA Neurology.*

M24674	Ankylosis, right foot	Arthritis Joint Lower Limb	Nociceptive Pain
M24675	Ankylosis, left foot	Arthritis Joint Lower Limb	Nociceptive Pain
M24676	Ankylosis, unspecified foot	Arthritis Joint Lower Limb	Nociceptive Pain
M2469	Ankylosis, other specified joint	Arthritis Joint Other	Nociceptive Pain
M247	Protrusio acetabuli	Arthritis Joint Other	Nociceptive Pain
M248	Other specific joint derangements, not elsewhere classified	Arthritis Joint Other	Nociceptive Pain
M2480	Oth specific joint derangements of unsp joint, NEC	Arthritis Joint Other	Nociceptive Pain
M2481	Oth specific joint derangements of shoulder, NEC	Arthritis Joint Upper Limb	Nociceptive Pain
M24811	Oth specific joint derangements of right shoulder, NEC	Arthritis Joint Upper Limb	Nociceptive Pain
M24812	Oth specific joint derangements of left shoulder, NEC	Arthritis Joint Upper Limb	Nociceptive Pain
M24819	Oth specific joint derangements of unsp shoulder, NEC	Arthritis Joint Upper Limb	Nociceptive Pain
M2482	Oth specific joint derangements of elbow, NEC	Arthritis Joint Upper Limb	Nociceptive Pain
M24821	Oth specific joint derangements of right elbow, NEC	Arthritis Joint Upper Limb	Nociceptive Pain
M24822	Oth specific joint derangements of left elbow, NEC	Arthritis Joint Upper Limb	Nociceptive Pain
M24829	Oth specific joint derangements of unsp elbow, NEC	Arthritis Joint Upper Limb	Nociceptive Pain
M2483	Oth specific joint derangements of wrist, NEC	Arthritis Joint Upper Limb	Nociceptive Pain
M24831	Oth specific joint derangements of right wrist, NEC	Arthritis Joint Upper Limb	Nociceptive Pain
M24832	Oth specific joint derangements of left wrist, NEC	Arthritis Joint Upper Limb	Nociceptive Pain
M24839	Oth specific joint derangements of unsp wrist, NEC	Arthritis Joint Upper Limb	Nociceptive Pain
M2484	Oth specific joint derangements of hand, NEC	Arthritis Joint Upper Limb	Nociceptive Pain
M24841	Oth specific joint derangements of right hand, NEC	Arthritis Joint Upper Limb	Nociceptive Pain
M24842	Oth specific joint derangements of left hand, NEC	Arthritis Joint Upper Limb	Nociceptive Pain
M24849	Oth specific joint derangements of unsp hand, NEC	Arthritis Joint Upper Limb	Nociceptive Pain
M2485	Oth specific joint derangements of hip, NEC	Arthritis Joint Spine and Hips	Nociceptive Pain
M24851	Oth specific joint derangements of right hip, NEC	Arthritis Joint Spine and Hips	Nociceptive Pain
M24852	Oth specific joint derangements of left hip, NEC	Arthritis Joint Spine and Hips	Nociceptive Pain
M24859	Oth specific joint derangements of unsp hip, NEC	Arthritis Joint Spine and Hips	Nociceptive Pain
M2487	Oth specific joint derangements of ankle and foot, NEC	Arthritis Joint Lower Limb	Nociceptive Pain
M24871	Oth specific joint derangements of right ankle, NEC	Arthritis Joint Lower Limb	Nociceptive Pain
M24872	Oth specific joint derangements of left ankle, NEC	Arthritis Joint Lower Limb	Nociceptive Pain
M24873	Oth specific joint derangements of unsp ankle, NEC	Arthritis Joint Lower Limb	Nociceptive Pain
M24874	Oth specific joint derangements of right foot, NEC	Arthritis Joint Lower Limb	Nociceptive Pain
M24875	Oth specific joint derangements left foot, NEC	Arthritis Joint Lower Limb	Nociceptive Pain
M24876	Oth specific joint derangements of unsp foot, NEC	Arthritis Joint Lower Limb	Nociceptive Pain
M2489	Other specific joint derangement of oth joint, NEC	Arthritis Joint Other	Nociceptive Pain
M249	Joint derangement, unspecified	Arthritis Joint Other	Nociceptive Pain
M25	Other joint disorder, not elsewhere classified	Arthritis Joint Other	Nociceptive Pain

© 2024 Peterson MD et al. *JAMA Neurology.*

M250	Hemarthrosis	Arthritis Joint Other	Nociceptive Pain
M2500	Hemarthrosis, unspecified joint	Arthritis Joint Other	Nociceptive Pain
M2501	Hemarthrosis, shoulder	Arthritis Joint Upper Limb	Nociceptive Pain
M25011	Hemarthrosis, right shoulder	Arthritis Joint Upper Limb	Nociceptive Pain
M25012	Hemarthrosis, left shoulder	Arthritis Joint Upper Limb	Nociceptive Pain
M25019	Hemarthrosis, unspecified shoulder	Arthritis Joint Upper Limb	Nociceptive Pain
M2502	Hemarthrosis, elbow	Arthritis Joint Upper Limb	Nociceptive Pain
M25021	Hemarthrosis, right elbow	Arthritis Joint Upper Limb	Nociceptive Pain
M25022	Hemarthrosis, left elbow	Arthritis Joint Upper Limb	Nociceptive Pain
M25029	Hemarthrosis, unspecified elbow	Arthritis Joint Upper Limb	Nociceptive Pain
M2503	Hemarthrosis, wrist	Arthritis Joint Upper Limb	Nociceptive Pain
M25031	Hemarthrosis, right wrist	Arthritis Joint Upper Limb	Nociceptive Pain
M25032	Hemarthrosis, left wrist	Arthritis Joint Upper Limb	Nociceptive Pain
M25039	Hemarthrosis, unspecified wrist	Arthritis Joint Upper Limb	Nociceptive Pain
M2504	Hemarthrosis, hand	Arthritis Joint Upper Limb	Nociceptive Pain
M25041	Hemarthrosis, right hand	Arthritis Joint Upper Limb	Nociceptive Pain
M25042	Hemarthrosis, left hand	Arthritis Joint Upper Limb	Nociceptive Pain
M25049	Hemarthrosis, unspecified hand	Arthritis Joint Upper Limb	Nociceptive Pain
M2505	Hemarthrosis, hip	Arthritis Joint Spine and Hips	Nociceptive Pain
M25051	Hemarthrosis, right hip	Arthritis Joint Spine and Hips	Nociceptive Pain
M25052	Hemarthrosis, left hip	Arthritis Joint Spine and Hips	Nociceptive Pain
M25059	Hemarthrosis, unspecified hip	Arthritis Joint Spine and Hips	Nociceptive Pain
M2506	Hemarthrosis, knee	Arthritis Joint Lower Limb	Nociceptive Pain
M25061	Hemarthrosis, right knee	Arthritis Joint Lower Limb	Nociceptive Pain
M25062	Hemarthrosis, left knee	Arthritis Joint Lower Limb	Nociceptive Pain
M25069	Hemarthrosis, unspecified knee	Arthritis Joint Lower Limb	Nociceptive Pain
M2507	Hemarthrosis, ankle and foot	Arthritis Joint Lower Limb	Nociceptive Pain
M25071	Hemarthrosis, right ankle	Arthritis Joint Lower Limb	Nociceptive Pain
M25072	Hemarthrosis, left ankle	Arthritis Joint Lower Limb	Nociceptive Pain
M25073	Hemarthrosis, unspecified ankle	Arthritis Joint Lower Limb	Nociceptive Pain
M25074	Hemarthrosis, right foot	Arthritis Joint Lower Limb	Nociceptive Pain
M25075	Hemarthrosis, left foot	Arthritis Joint Lower Limb	Nociceptive Pain
M25076	Hemarthrosis, unspecified foot	Arthritis Joint Lower Limb	Nociceptive Pain
M2508	Hemarthrosis, other specified site	Arthritis Joint Other	Nociceptive Pain
M251	Fistula of joint	Arthritis Joint Other	Nociceptive Pain
M2510	Fistula, unspecified joint	Arthritis Joint Other	Nociceptive Pain
M2511	Fistula, shoulder	Arthritis Joint Upper Limb	Nociceptive Pain

© 2024 Peterson MD et al. *JAMA Neurology.*

M25111	Fistula, right shoulder	Arthritis Joint Upper Limb	Nociceptive Pain
M25112	Fistula, left shoulder	Arthritis Joint Upper Limb	Nociceptive Pain
M25119	Fistula, unspecified shoulder	Arthritis Joint Upper Limb	Nociceptive Pain
M2512	Fistula, elbow	Arthritis Joint Upper Limb	Nociceptive Pain
M25121	Fistula, right elbow	Arthritis Joint Upper Limb	Nociceptive Pain
M25122	Fistula, left elbow	Arthritis Joint Upper Limb	Nociceptive Pain
M25129	Fistula, unspecified elbow	Arthritis Joint Upper Limb	Nociceptive Pain
M2513	Fistula, wrist	Arthritis Joint Upper Limb	Nociceptive Pain
M25131	Fistula, right wrist	Arthritis Joint Upper Limb	Nociceptive Pain
M25132	Fistula, left wrist	Arthritis Joint Upper Limb	Nociceptive Pain
M25139	Fistula, unspecified wrist	Arthritis Joint Upper Limb	Nociceptive Pain
M2514	Fistula, hand	Arthritis Joint Upper Limb	Nociceptive Pain
M25141	Fistula, right hand	Arthritis Joint Upper Limb	Nociceptive Pain
M25142	Fistula, left hand	Arthritis Joint Upper Limb	Nociceptive Pain
M25149	Fistula, unspecified hand	Arthritis Joint Upper Limb	Nociceptive Pain
M2515	Fistula, hip	Arthritis Joint Spine and Hips	Nociceptive Pain
M25151	Fistula, right hip	Arthritis Joint Spine and Hips	Nociceptive Pain
M25152	Fistula, left hip	Arthritis Joint Spine and Hips	Nociceptive Pain
M25159	Fistula, unspecified hip	Arthritis Joint Spine and Hips	Nociceptive Pain
M2516	Fistula, knee	Arthritis Joint Lower Limb	Nociceptive Pain
M25161	Fistula, right knee	Arthritis Joint Lower Limb	Nociceptive Pain
M25162	Fistula, left knee	Arthritis Joint Lower Limb	Nociceptive Pain
M25169	Fistula, unspecified knee	Arthritis Joint Lower Limb	Nociceptive Pain
M2517	Fistula, ankle and foot	Arthritis Joint Lower Limb	Nociceptive Pain
M25171	Fistula, right ankle	Arthritis Joint Lower Limb	Nociceptive Pain
M25172	Fistula, left ankle	Arthritis Joint Lower Limb	Nociceptive Pain
M25173	Fistula, unspecified ankle	Arthritis Joint Lower Limb	Nociceptive Pain
M25174	Fistula, right foot	Arthritis Joint Lower Limb	Nociceptive Pain
M25175	Fistula, left foot	Arthritis Joint Lower Limb	Nociceptive Pain
M25176	Fistula, unspecified foot	Arthritis Joint Lower Limb	Nociceptive Pain
M2518	Fistula, other specified site	Arthritis Joint Other	Nociceptive Pain
M252	Flail joint	Arthritis Joint Other	Nociceptive Pain
M2520	Flail joint, unspecified joint	Arthritis Joint Other	Nociceptive Pain
M2521	Flail joint, shoulder	Arthritis Joint Upper Limb	Nociceptive Pain
M25211	Flail joint, right shoulder	Arthritis Joint Upper Limb	Nociceptive Pain
M25212	Flail joint, left shoulder	Arthritis Joint Upper Limb	Nociceptive Pain
M25219	Flail joint, unspecified shoulder	Arthritis Joint Upper Limb	Nociceptive Pain

© 2024 Peterson MD et al. *JAMA Neurology.*

M2522	Flail joint, elbow	Arthritis Joint Upper Limb	Nociceptive Pain
M25221	Flail joint, right elbow	Arthritis Joint Upper Limb	Nociceptive Pain
M25222	Flail joint, left elbow	Arthritis Joint Upper Limb	Nociceptive Pain
M25229	Flail joint, unspecified elbow	Arthritis Joint Upper Limb	Nociceptive Pain
M2523	Flail joint, wrist	Arthritis Joint Upper Limb	Nociceptive Pain
M25231	Flail joint, right wrist	Arthritis Joint Upper Limb	Nociceptive Pain
M25232	Flail joint, left wrist	Arthritis Joint Upper Limb	Nociceptive Pain
M25239	Flail joint, unspecified wrist	Arthritis Joint Upper Limb	Nociceptive Pain
M2524	Flail joint, hand	Arthritis Joint Upper Limb	Nociceptive Pain
M25241	Flail joint, right hand	Arthritis Joint Upper Limb	Nociceptive Pain
M25242	Flail joint, left hand	Arthritis Joint Upper Limb	Nociceptive Pain
M25249	Flail joint, unspecified hand	Arthritis Joint Upper Limb	Nociceptive Pain
M2525	Flail joint, hip	Arthritis Joint Spine and Hips	Nociceptive Pain
M25251	Flail joint, right hip	Arthritis Joint Spine and Hips	Nociceptive Pain
M25252	Flail joint, left hip	Arthritis Joint Spine and Hips	Nociceptive Pain
M25259	Flail joint, unspecified hip	Arthritis Joint Spine and Hips	Nociceptive Pain
M2526	Flail joint, knee	Arthritis Joint Lower Limb	Nociceptive Pain
M25261	Flail joint, right knee	Arthritis Joint Lower Limb	Nociceptive Pain
M25262	Flail joint, left knee	Arthritis Joint Lower Limb	Nociceptive Pain
M25269	Flail joint, unspecified knee	Arthritis Joint Lower Limb	Nociceptive Pain
M2527	Flail joint, ankle and foot	Arthritis Joint Lower Limb	Nociceptive Pain
M25271	Flail joint, right ankle and foot	Arthritis Joint Lower Limb	Nociceptive Pain
M25272	Flail joint, left ankle and foot	Arthritis Joint Lower Limb	Nociceptive Pain
M25279	Flail joint, unspecified ankle and foot	Arthritis Joint Lower Limb	Nociceptive Pain
M2528	Flail joint, other site	Arthritis Joint Other	Nociceptive Pain
M253	Other instability of joint	Arthritis Joint Other	Nociceptive Pain
M2530	Other instability, unspecified joint	Arthritis Joint Other	Nociceptive Pain
M2531	Other instability, shoulder	Arthritis Joint Upper Limb	Nociceptive Pain
M25311	Other instability, right shoulder	Arthritis Joint Upper Limb	Nociceptive Pain
M25312	Other instability, left shoulder	Arthritis Joint Upper Limb	Nociceptive Pain
M25319	Other instability, unspecified shoulder	Arthritis Joint Upper Limb	Nociceptive Pain
M2532	Other instability, elbow	Arthritis Joint Upper Limb	Nociceptive Pain
M25321	Other instability, right elbow	Arthritis Joint Upper Limb	Nociceptive Pain
M25322	Other instability, left elbow	Arthritis Joint Upper Limb	Nociceptive Pain
M25329	Other instability, unspecified elbow	Arthritis Joint Upper Limb	Nociceptive Pain
M2533	Other instability, wrist	Arthritis Joint Upper Limb	Nociceptive Pain
M25331	Other instability, right wrist	Arthritis Joint Upper Limb	Nociceptive Pain

© 2024 Peterson MD et al. *JAMA Neurology.*

M25332	Other instability, left wrist	Arthritis Joint Upper Limb	Nociceptive Pain
M25339	Other instability, unspecified wrist	Arthritis Joint Upper Limb	Nociceptive Pain
M2534	Other instability, hand	Arthritis Joint Upper Limb	Nociceptive Pain
M25341	Other instability, right hand	Arthritis Joint Upper Limb	Nociceptive Pain
M25342	Other instability, left hand	Arthritis Joint Upper Limb	Nociceptive Pain
M25349	Other instability, unspecified hand	Arthritis Joint Upper Limb	Nociceptive Pain
M2535	Other instability, hip	Arthritis Joint Spine and Hips	Nociceptive Pain
M25351	Other instability, right hip	Arthritis Joint Spine and Hips	Nociceptive Pain
M25352	Other instability, left hip	Arthritis Joint Spine and Hips	Nociceptive Pain
M25359	Other instability, unspecified hip	Arthritis Joint Spine and Hips	Nociceptive Pain
M2536	Other instability, knee	Arthritis Joint Lower Limb	Nociceptive Pain
M25361	Other instability, right knee	Arthritis Joint Lower Limb	Nociceptive Pain
M25362	Other instability, left knee	Arthritis Joint Lower Limb	Nociceptive Pain
M25369	Other instability, unspecified knee	Arthritis Joint Lower Limb	Nociceptive Pain
M2537	Other instability, ankle and foot	Arthritis Joint Lower Limb	Nociceptive Pain
M25371	Other instability, right ankle	Arthritis Joint Lower Limb	Nociceptive Pain
M25372	Other instability, left ankle	Arthritis Joint Lower Limb	Nociceptive Pain
M25373	Other instability, unspecified ankle	Arthritis Joint Lower Limb	Nociceptive Pain
M25374	Other instability, right foot	Arthritis Joint Lower Limb	Nociceptive Pain
M25375	Other instability, left foot	Arthritis Joint Lower Limb	Nociceptive Pain
M25376	Other instability, unspecified foot	Arthritis Joint Lower Limb	Nociceptive Pain
M2539	Other instability, other specified joint	Arthritis Joint Other	Nociceptive Pain
M254	Effusion of joint	Arthritis Joint Other	Nociceptive Pain
M2540	Effusion, unspecified joint	Arthritis Joint Other	Nociceptive Pain
M2541	Effusion, shoulder	Arthritis Joint Upper Limb	Nociceptive Pain
M25411	Effusion, right shoulder	Arthritis Joint Upper Limb	Nociceptive Pain
M25412	Effusion, left shoulder	Arthritis Joint Upper Limb	Nociceptive Pain
M25419	Effusion, unspecified shoulder	Arthritis Joint Upper Limb	Nociceptive Pain
M2542	Effusion, elbow	Arthritis Joint Upper Limb	Nociceptive Pain
M25421	Effusion, right elbow	Arthritis Joint Upper Limb	Nociceptive Pain
M25422	Effusion, left elbow	Arthritis Joint Upper Limb	Nociceptive Pain
M25429	Effusion, unspecified elbow	Arthritis Joint Upper Limb	Nociceptive Pain
M2543	Effusion, wrist	Arthritis Joint Upper Limb	Nociceptive Pain
M25431	Effusion, right wrist	Arthritis Joint Upper Limb	Nociceptive Pain
M25432	Effusion, left wrist	Arthritis Joint Upper Limb	Nociceptive Pain
M25439	Effusion, unspecified wrist	Arthritis Joint Upper Limb	Nociceptive Pain
M2544	Effusion, hand	Arthritis Joint Upper Limb	Nociceptive Pain

© 2024 Peterson MD et al. *JAMA Neurology.*

M25441	Effusion, right hand	Arthritis Joint Upper Limb	Nociceptive Pain
M25442	Effusion, left hand	Arthritis Joint Upper Limb	Nociceptive Pain
M25449	Effusion, unspecified hand	Arthritis Joint Upper Limb	Nociceptive Pain
M2545	Effusion, hip	Arthritis Joint Spine and Hips	Nociceptive Pain
M25451	Effusion, right hip	Arthritis Joint Spine and Hips	Nociceptive Pain
M25452	Effusion, left hip	Arthritis Joint Spine and Hips	Nociceptive Pain
M25459	Effusion, unspecified hip	Arthritis Joint Spine and Hips	Nociceptive Pain
M2546	Effusion, knee	Arthritis Joint Lower Limb	Nociceptive Pain
M25461	Effusion, right knee	Arthritis Joint Lower Limb	Nociceptive Pain
M25462	Effusion, left knee	Arthritis Joint Lower Limb	Nociceptive Pain
M25469	Effusion, unspecified knee	Arthritis Joint Lower Limb	Nociceptive Pain
M2547	Effusion, ankle and foot	Arthritis Joint Lower Limb	Nociceptive Pain
M25471	Effusion, right ankle	Arthritis Joint Lower Limb	Nociceptive Pain
M25472	Effusion, left ankle	Arthritis Joint Lower Limb	Nociceptive Pain
M25473	Effusion, unspecified ankle	Arthritis Joint Lower Limb	Nociceptive Pain
M25474	Effusion, right foot	Arthritis Joint Lower Limb	Nociceptive Pain
M25475	Effusion, left foot	Arthritis Joint Lower Limb	Nociceptive Pain
M25476	Effusion, unspecified foot	Arthritis Joint Lower Limb	Nociceptive Pain
M2548	Effusion, other site	Arthritis Joint Other	Nociceptive Pain
M255	Pain in joint	Arthritis Joint Other	Nociceptive Pain
M2550	Pain in unspecified joint	Arthritis Joint Other	Nociceptive Pain
M2551	Pain in shoulder	Arthritis Joint Upper Limb	Nociceptive Pain
M25511	Pain in right shoulder	Arthritis Joint Upper Limb	Nociceptive Pain
M25512	Pain in left shoulder	Arthritis Joint Upper Limb	Nociceptive Pain
M25519	Pain in unspecified shoulder	Arthritis Joint Upper Limb	Nociceptive Pain
M2552	Pain in elbow	Arthritis Joint Upper Limb	Nociceptive Pain
M25521	Pain in right elbow	Arthritis Joint Upper Limb	Nociceptive Pain
M25522	Pain in left elbow	Arthritis Joint Upper Limb	Nociceptive Pain
M25529	Pain in unspecified elbow	Arthritis Joint Upper Limb	Nociceptive Pain
M2553	Pain in wrist	Arthritis Joint Upper Limb	Nociceptive Pain
M25531	Pain in right wrist	Arthritis Joint Upper Limb	Nociceptive Pain
M25532	Pain in left wrist	Arthritis Joint Upper Limb	Nociceptive Pain
M25539	Pain in unspecified wrist	Arthritis Joint Upper Limb	Nociceptive Pain
M2554	Pain in joints of hand	Arthritis Joint Upper Limb	Nociceptive Pain
M25541	Pain in joints of right hand	Arthritis Joint Upper Limb	Nociceptive Pain
M25542	Pain in joints of left hand	Arthritis Joint Upper Limb	Nociceptive Pain
M25549	Pain in joints of unspecified hand	Arthritis Joint Upper Limb	Nociceptive Pain

© 2024 Peterson MD et al. *JAMA Neurology.*

M2555	Pain in hip	Arthritis Joint Spine and Hips	Nociceptive Pain
M25551	Pain in right hip	Arthritis Joint Spine and Hips	Nociceptive Pain
M25552	Pain in left hip	Arthritis Joint Spine and Hips	Nociceptive Pain
M25559	Pain in unspecified hip	Arthritis Joint Spine and Hips	Nociceptive Pain
M2556	Pain in knee	Arthritis Joint Lower Limb	Nociceptive Pain
M25561	Pain in right knee	Arthritis Joint Lower Limb	Nociceptive Pain
M25562	Pain in left knee	Arthritis Joint Lower Limb	Nociceptive Pain
M25569	Pain in unspecified knee	Arthritis Joint Lower Limb	Nociceptive Pain
M2557	Pain in ankle and joints of foot	Arthritis Joint Lower Limb	Nociceptive Pain
M25571	Pain in right ankle and joints of right foot	Arthritis Joint Lower Limb	Nociceptive Pain
M25572	Pain in left ankle and joints of left foot	Arthritis Joint Lower Limb	Nociceptive Pain
M25579	Pain in unspecified ankle and joints of unspecified foot	Arthritis Joint Lower Limb	Nociceptive Pain
M2559	Pain in other specified joint	Arthritis Joint Other	Nociceptive Pain
M256	Stiffness of joint, not elsewhere classified	Arthritis Joint Other	Nociceptive Pain
M2560	Stiffness of unspecified joint, not elsewhere classified	Arthritis Joint Other	Nociceptive Pain
M2561	Stiffness of shoulder, not elsewhere classified	Arthritis Joint Upper Limb	Nociceptive Pain
M25611	Stiffness of right shoulder, not elsewhere classified	Arthritis Joint Upper Limb	Nociceptive Pain
M25612	Stiffness of left shoulder, not elsewhere classified	Arthritis Joint Upper Limb	Nociceptive Pain
M25619	Stiffness of unspecified shoulder, not elsewhere classified	Arthritis Joint Upper Limb	Nociceptive Pain
M2562	Stiffness of elbow, not elsewhere classified	Arthritis Joint Upper Limb	Nociceptive Pain
M25621	Stiffness of right elbow, not elsewhere classified	Arthritis Joint Upper Limb	Nociceptive Pain
M25622	Stiffness of left elbow, not elsewhere classified	Arthritis Joint Upper Limb	Nociceptive Pain
M25629	Stiffness of unspecified elbow, not elsewhere classified	Arthritis Joint Upper Limb	Nociceptive Pain
M2563	Stiffness of wrist, not elsewhere classified	Arthritis Joint Upper Limb	Nociceptive Pain
M25631	Stiffness of right wrist, not elsewhere classified	Arthritis Joint Upper Limb	Nociceptive Pain
M25632	Stiffness of left wrist, not elsewhere classified	Arthritis Joint Upper Limb	Nociceptive Pain
M25639	Stiffness of unspecified wrist, not elsewhere classified	Arthritis Joint Upper Limb	Nociceptive Pain
M2564	Stiffness of hand, not elsewhere classified	Arthritis Joint Upper Limb	Nociceptive Pain
M25641	Stiffness of right hand, not elsewhere classified	Arthritis Joint Upper Limb	Nociceptive Pain
M25642	Stiffness of left hand, not elsewhere classified	Arthritis Joint Upper Limb	Nociceptive Pain
M25649	Stiffness of unspecified hand, not elsewhere classified	Arthritis Joint Upper Limb	Nociceptive Pain
M2565	Stiffness of hip, not elsewhere classified	Arthritis Joint Spine and Hips	Nociceptive Pain
M25651	Stiffness of right hip, not elsewhere classified	Arthritis Joint Spine and Hips	Nociceptive Pain
M25652	Stiffness of left hip, not elsewhere classified	Arthritis Joint Spine and Hips	Nociceptive Pain
M25659	Stiffness of unspecified hip, not elsewhere classified	Arthritis Joint Spine and Hips	Nociceptive Pain
M2566	Stiffness of knee, not elsewhere classified	Arthritis Joint Lower Limb	Nociceptive Pain
M25661	Stiffness of right knee, not elsewhere classified	Arthritis Joint Lower Limb	Nociceptive Pain

© 2024 Peterson MD et al. *JAMA Neurology.*

M25662	Stiffness of left knee, not elsewhere classified	Arthritis Joint Lower Limb	Nociceptive Pain
M25669	Stiffness of unspecified knee, not elsewhere classified	Arthritis Joint Lower Limb	Nociceptive Pain
M2567	Stiffness of ankle and foot, not elsewhere classified	Arthritis Joint Lower Limb	Nociceptive Pain
M25671	Stiffness of right ankle, not elsewhere classified	Arthritis Joint Lower Limb	Nociceptive Pain
M25672	Stiffness of left ankle, not elsewhere classified	Arthritis Joint Lower Limb	Nociceptive Pain
M25673	Stiffness of unspecified ankle, not elsewhere classified	Arthritis Joint Lower Limb	Nociceptive Pain
M25674	Stiffness of right foot, not elsewhere classified	Arthritis Joint Lower Limb	Nociceptive Pain
M25675	Stiffness of left foot, not elsewhere classified	Arthritis Joint Lower Limb	Nociceptive Pain
M25676	Stiffness of unspecified foot, not elsewhere classified	Arthritis Joint Lower Limb	Nociceptive Pain
M2569	Stiffness of other specified joint, not elsewhere classified	Arthritis Joint Other	Nociceptive Pain
M257	Osteophyte	Arthritis Joint Other	Nociceptive Pain
M2570	Osteophyte, unspecified joint	Arthritis Joint Other	Nociceptive Pain
M2571	Osteophyte, shoulder	Arthritis Joint Upper Limb	Nociceptive Pain
M25711	Osteophyte, right shoulder	Arthritis Joint Upper Limb	Nociceptive Pain
M25712	Osteophyte, left shoulder	Arthritis Joint Upper Limb	Nociceptive Pain
M25719	Osteophyte, unspecified shoulder	Arthritis Joint Upper Limb	Nociceptive Pain
M2572	Osteophyte, elbow	Arthritis Joint Upper Limb	Nociceptive Pain
M25721	Osteophyte, right elbow	Arthritis Joint Upper Limb	Nociceptive Pain
M25722	Osteophyte, left elbow	Arthritis Joint Upper Limb	Nociceptive Pain
M25729	Osteophyte, unspecified elbow	Arthritis Joint Upper Limb	Nociceptive Pain
M2573	Osteophyte, wrist	Arthritis Joint Upper Limb	Nociceptive Pain
M25731	Osteophyte, right wrist	Arthritis Joint Upper Limb	Nociceptive Pain
M25732	Osteophyte, left wrist	Arthritis Joint Upper Limb	Nociceptive Pain
M25739	Osteophyte, unspecified wrist	Arthritis Joint Upper Limb	Nociceptive Pain
M2574	Osteophyte, hand	Arthritis Joint Upper Limb	Nociceptive Pain
M25741	Osteophyte, right hand	Arthritis Joint Upper Limb	Nociceptive Pain
M25742	Osteophyte, left hand	Arthritis Joint Upper Limb	Nociceptive Pain
M25749	Osteophyte, unspecified hand	Arthritis Joint Upper Limb	Nociceptive Pain
M2575	Osteophyte, hip	Arthritis Joint Spine and Hips	Nociceptive Pain
M25751	Osteophyte, right hip	Arthritis Joint Spine and Hips	Nociceptive Pain
M25752	Osteophyte, left hip	Arthritis Joint Spine and Hips	Nociceptive Pain
M25759	Osteophyte, unspecified hip	Arthritis Joint Spine and Hips	Nociceptive Pain
M2576	Osteophyte, knee	Arthritis Joint Lower Limb	Nociceptive Pain
M25761	Osteophyte, right knee	Arthritis Joint Lower Limb	Nociceptive Pain
M25762	Osteophyte, left knee	Arthritis Joint Lower Limb	Nociceptive Pain
M25769	Osteophyte, unspecified knee	Arthritis Joint Lower Limb	Nociceptive Pain
M2577	Osteophyte, ankle and foot	Arthritis Joint Lower Limb	Nociceptive Pain

© 2024 Peterson MD et al. *JAMA Neurology.*

M25771	Osteophyte, right ankle	Arthritis Joint Lower Limb	Nociceptive Pain
M25772	Osteophyte, left ankle	Arthritis Joint Lower Limb	Nociceptive Pain
M25773	Osteophyte, unspecified ankle	Arthritis Joint Lower Limb	Nociceptive Pain
M25774	Osteophyte, right foot	Arthritis Joint Lower Limb	Nociceptive Pain
M25775	Osteophyte, left foot	Arthritis Joint Lower Limb	Nociceptive Pain
M25776	Osteophyte, unspecified foot	Arthritis Joint Lower Limb	Nociceptive Pain
M2578	Osteophyte, vertebrae	Arthritis Joint Spine and Hips	Nociceptive Pain
M258	Other specified joint disorders	Arthritis Joint Other	Nociceptive Pain
M2580	Other specified joint disorders, unspecified joint	Arthritis Joint Other	Nociceptive Pain
M2581	Other specified joint disorders, shoulder	Arthritis Joint Upper Limb	Nociceptive Pain
M25811	Other specified joint disorders, right shoulder	Arthritis Joint Upper Limb	Nociceptive Pain
M25812	Other specified joint disorders, left shoulder	Arthritis Joint Upper Limb	Nociceptive Pain
M25819	Other specified joint disorders, unspecified shoulder	Arthritis Joint Upper Limb	Nociceptive Pain
M2582	Other specified joint disorders, elbow	Arthritis Joint Upper Limb	Nociceptive Pain
M25821	Other specified joint disorders, right elbow	Arthritis Joint Upper Limb	Nociceptive Pain
M25822	Other specified joint disorders, left elbow	Arthritis Joint Upper Limb	Nociceptive Pain
M25829	Other specified joint disorders, unspecified elbow	Arthritis Joint Upper Limb	Nociceptive Pain
M2583	Other specified joint disorders, wrist	Arthritis Joint Upper Limb	Nociceptive Pain
M25831	Other specified joint disorders, right wrist	Arthritis Joint Upper Limb	Nociceptive Pain
M25832	Other specified joint disorders, left wrist	Arthritis Joint Upper Limb	Nociceptive Pain
M25839	Other specified joint disorders, unspecified wrist	Arthritis Joint Upper Limb	Nociceptive Pain
M2584	Other specified joint disorders, hand	Arthritis Joint Upper Limb	Nociceptive Pain
M25841	Other specified joint disorders, right hand	Arthritis Joint Upper Limb	Nociceptive Pain
M25842	Other specified joint disorders, left hand	Arthritis Joint Upper Limb	Nociceptive Pain
M25849	Other specified joint disorders, unspecified hand	Arthritis Joint Upper Limb	Nociceptive Pain
M2585	Other specified joint disorders, hip	Arthritis Joint Spine and Hips	Nociceptive Pain
M25851	Other specified joint disorders, right hip	Arthritis Joint Spine and Hips	Nociceptive Pain
M25852	Other specified joint disorders, left hip	Arthritis Joint Spine and Hips	Nociceptive Pain
M25859	Other specified joint disorders, unspecified hip	Arthritis Joint Spine and Hips	Nociceptive Pain
M2586	Other specified joint disorders, knee	Arthritis Joint Lower Limb	Nociceptive Pain
M25861	Other specified joint disorders, right knee	Arthritis Joint Lower Limb	Nociceptive Pain
M25862	Other specified joint disorders, left knee	Arthritis Joint Lower Limb	Nociceptive Pain
M25869	Other specified joint disorders, unspecified knee	Arthritis Joint Lower Limb	Nociceptive Pain
M2587	Other specified joint disorders, ankle and foot	Arthritis Joint Lower Limb	Nociceptive Pain
M25871	Other specified joint disorders, right ankle and foot	Arthritis Joint Lower Limb	Nociceptive Pain
M25872	Other specified joint disorders, left ankle and foot	Arthritis Joint Lower Limb	Nociceptive Pain
M25879	Other specified joint disorders, unspecified ankle and foot	Arthritis Joint Lower Limb	Nociceptive Pain

© 2024 Peterson MD et al. *JAMA Neurology.*

M259	Joint disorder, unspecified	Arthritis Joint Other	Nociceptive Pain
M32	Systemic lupus erythematosus (SLE)	Arthritis Joint Other	Nociceptive Pain
M320	Drug-induced systemic lupus erythematosus	Arthritis Joint Other	Nociceptive Pain
M321	Systemic lupus erythematosus w organ or system involvement	Arthritis Joint Other	Nociceptive Pain
M3210	Systemic lupus erythematosus, organ or system involv unsp	Arthritis Joint Other	Nociceptive Pain
M3211	Endocarditis in systemic lupus erythematosus	Arthritis Joint Other	Nociceptive Pain
M3212	Pericarditis in systemic lupus erythematosus	Arthritis Joint Other	Nociceptive Pain
M3213	Lung involvement in systemic lupus erythematosus	Arthritis Joint Other	Nociceptive Pain
M3214	Glomerular disease in systemic lupus erythematosus	Arthritis Joint Other	Nociceptive Pain
M3215	Tubulo-interstitial nephrop in sys lupus erythematosus	Arthritis Joint Other	Nociceptive Pain
M3219	Oth organ or system involv in systemic lupus erythematosus	Arthritis Joint Other	Nociceptive Pain
M328	Other forms of systemic lupus erythematosus	Arthritis Joint Other	Nociceptive Pain
M329	Systemic lupus erythematosus, unspecified	Arthritis Joint Other	Nociceptive Pain
M33	Dermatopolymyositis	Arthritis Joint Other	Nociceptive Pain
M330	Juvenile dermatomyositis	Arthritis Joint Other	Nociceptive Pain
M3300	Juvenile dermatomyositis, organ involvement unspecified	Arthritis Joint Other	Nociceptive Pain
M3301	Juvenile dermatomyositis with respiratory involvement	Arthritis Joint Other	Nociceptive Pain
M3302	Juvenile dermatomyositis with myopathy	Arthritis Joint Other	Nociceptive Pain
M3303	Juvenile dermatomyositis without myopathy	Arthritis Joint Other	Nociceptive Pain
M3309	Juvenile dermatomyositis with other organ involvement	Arthritis Joint Other	Nociceptive Pain
M331	Other dermatomyositis	Arthritis Joint Other	Nociceptive Pain
M3310	Other dermatomyositis, organ involvement unspecified	Arthritis Joint Other	Nociceptive Pain
M3311	Other dermatomyositis with respiratory involvement	Arthritis Joint Other	Nociceptive Pain
M3312	Other dermatomyositis with myopathy	Arthritis Joint Other	Nociceptive Pain
M3313	Other dermatomyositis without myopathy	Arthritis Joint Other	Nociceptive Pain
M3319	Other dermatomyositis with other organ involvement	Arthritis Joint Other	Nociceptive Pain
M332	Polymyositis	Arthritis Joint Other	Nociceptive Pain
M3320	Polymyositis, organ involvement unspecified	Arthritis Joint Other	Nociceptive Pain
M3321	Polymyositis with respiratory involvement	Arthritis Joint Other	Nociceptive Pain
M3322	Polymyositis with myopathy	Arthritis Joint Other	Nociceptive Pain
M3329	Polymyositis with other organ involvement	Arthritis Joint Other	Nociceptive Pain
M339	Dermatopolymyositis, unspecified	Arthritis Joint Other	Nociceptive Pain
M3390	Dermatopolymyositis, unsp, organ involvement unspecified	Arthritis Joint Other	Nociceptive Pain
M3391	Dermatopolymyositis, unsp with respiratory involvement	Arthritis Joint Other	Nociceptive Pain
M3392	Dermatopolymyositis, unspecified with myopathy	Arthritis Joint Other	Nociceptive Pain
M3393	Dermatopolymyositis, unspecified without myopathy	Arthritis Joint Other	Nociceptive Pain
M3399	Dermatopolymyositis, unsp with other organ involvement	Arthritis Joint Other	Nociceptive Pain

© 2024 Peterson MD et al. *JAMA Neurology.*

M34	Systemic sclerosis [scleroderma]	Arthritis Joint Other	Nociceptive Pain
M340	Progressive systemic sclerosis	Arthritis Joint Other	Nociceptive Pain
M341	CR(E)ST syndrome	Arthritis Joint Other	Nociceptive Pain
M342	Systemic sclerosis induced by drug and chemical	Arthritis Joint Other	Nociceptive Pain
M348	Other forms of systemic sclerosis	Arthritis Joint Other	Nociceptive Pain
M3481	Systemic sclerosis with lung involvement	Arthritis Joint Other	Nociceptive Pain
M3482	Systemic sclerosis with myopathy	Arthritis Joint Other	Nociceptive Pain
M3483	Systemic sclerosis with polyneuropathy	Arthritis Joint Other	Nociceptive Pain
M3489	Other systemic sclerosis	Arthritis Joint Other	Nociceptive Pain
M349	Systemic sclerosis, unspecified	Arthritis Joint Other	Nociceptive Pain
M351	Other overlap syndromes	Arthritis Joint Other	Nociceptive Pain
M352	Behcet's disease	Arthritis Joint Other	Nociceptive Pain
M353	Polymyalgia rheumatica	Arthritis Joint Other	Nociceptive Pain
M354	Diffuse (eosinophilic) fasciitis	Arthritis Joint Other	Nociceptive Pain
M355	Multifocal fibrosclerosis	Arthritis Joint Other	Nociceptive Pain
M356	Relapsing panniculitis [Weber-Christian]	Arthritis Joint Other	Nociceptive Pain
M357	Hypermobility syndrome	Arthritis Joint Other	Nociceptive Pain
M358	Other specified systemic involvement of connective tissue	Arthritis Joint Other	Nociceptive Pain
M3581	Multisystem inflammatory syndrome	Arthritis Joint Other	Nociceptive Pain
M3589	Other specified systemic involvement of connective tissue	Arthritis Joint Other	Nociceptive Pain
M359	Systemic involvement of connective tissue, unspecified	Arthritis Joint Other	Nociceptive Pain
M36	Systemic disorders of conn tiss in diseases classd elswhr	Arthritis Joint Other	Nociceptive Pain
M360	Dermato(poly)myositis in neoplastic disease	Arthritis Joint Other	Nociceptive Pain
M361	Arthropathy in neoplastic disease	Arthritis Joint Other	Nociceptive Pain
M362	Hemophilic arthropathy	Arthritis Joint Other	Nociceptive Pain
M363	Arthropathy in other blood disorders	Arthritis Joint Other	Nociceptive Pain
M364	Arthropathy in hypersensitivity reactions classd elswhr	Arthritis Joint Other	Nociceptive Pain
M368	Systemic disord of conn tiss in oth diseases classd elswhr	Arthritis Joint Other	Nociceptive Pain
M400	Postural kyphosis	Back Pain Other/Unspecified	Nociceptive Pain
M401	Other secondary kyphosis	Arthritis Joint Spine and Hips	Nociceptive Pain
M402	Other and unspecified kyphosis	Back Pain Other/Unspecified	Nociceptive Pain
M404	Postural lordosis	Arthritis Joint Other	Nociceptive Pain
M405	Lordosis, unspecified	Arthritis Joint Other	Nociceptive Pain
M4100	Infantile idiopathic scoliosis, site unspecified	Back Pain Other/Unspecified	Nociceptive Pain
M4102	Infantile idiopathic scoliosis, cervical region	Neck Pain	Nociceptive Pain
M4103	Infantile idiopathic scoliosis, cervicothoracic region	Neck Pain	Nociceptive Pain
M4104	Infantile idiopathic scoliosis, thoracic region	Back Pain Mid Back	Nociceptive Pain

© 2024 Peterson MD et al. *JAMA Neurology.*

M4105	Infantile idiopathic scoliosis, thoracolumbar region	Back Pain Mid Back	Nociceptive Pain
M4106	Infantile idiopathic scoliosis, lumbar region	Back Pain Low Back	Nociceptive Pain
M4107	Infantile idiopathic scoliosis, lumbosacral region	Back Pain Low Back	Nociceptive Pain
M4108	Infantile idiopathic scoliosis, sacr/sacrocygl region	Back Pain Low Back	Nociceptive Pain
M4120	Other idiopathic scoliosis, site unspecified	Back Pain Other/Unspecified	Nociceptive Pain
M4122	Other idiopathic scoliosis, cervical region	Neck Pain	Nociceptive Pain
M4123	Other idiopathic scoliosis, cervicothoracic region	Neck Pain	Nociceptive Pain
M4124	Other idiopathic scoliosis, thoracic region	Back Pain Mid Back	Nociceptive Pain
M4125	Other idiopathic scoliosis, thoracolumbar region	Back Pain Mid Back	Nociceptive Pain
M4126	Other idiopathic scoliosis, lumbar region	Back Pain Low Back	Nociceptive Pain
M4127	Other idiopathic scoliosis, lumbosacral region	Back Pain Low Back	Nociceptive Pain
M413	Thoracogenic scoliosis	Back Pain Mid Back	Nociceptive Pain
M414	Neuromuscular scoliosis	Arthritis Joint Other	Nociceptive Pain
M415	Other secondary scoliosis	Arthritis Joint Other	Nociceptive Pain
M4180	Other forms of scoliosis, site unspecified	Back Pain Other/Unspecified	Nociceptive Pain
M4182	Other forms of scoliosis, cervical region	Neck Pain	Nociceptive Pain
M4183	Other forms of scoliosis, cervicothoracic region	Neck Pain	Nociceptive Pain
M4184	Other forms of scoliosis, thoracic region	Back Pain Mid Back	Nociceptive Pain
M4185	Other forms of scoliosis, thoracolumbar region	Back Pain Mid Back	Nociceptive Pain
M4186	Other forms of scoliosis, lumbar region	Back Pain Low Back	Nociceptive Pain
M4187	Other forms of scoliosis, lumbosacral region	Back Pain Low Back	Nociceptive Pain
M419	Scoliosis, unspecified	Back Pain Other/Unspecified	Nociceptive Pain
M42	Spinal osteochondrosis	Arthritis Joint Other	Nociceptive Pain
M420	Juvenile osteochondrosis of spine	Arthritis Joint Spine and Hips	Nociceptive Pain
M4200	Juvenile osteochondrosis of spine, site unspecified	Arthritis Joint Spine and Hips	Nociceptive Pain
M4201	Juvenile osteochondrosis of spine, occipt-atlan-ax region	Arthritis Joint Spine and Hips	Nociceptive Pain
M4202	Juvenile osteochondrosis of spine, cervical region	Arthritis Joint Spine and Hips	Nociceptive Pain
M4203	Juvenile osteochondrosis of spine, cervicothoracic region	Arthritis Joint Spine and Hips	Nociceptive Pain
M4204	Juvenile osteochondrosis of spine, thoracic region	Arthritis Joint Spine and Hips	Nociceptive Pain
M4205	Juvenile osteochondrosis of spine, thoracolumbar region	Arthritis Joint Spine and Hips	Nociceptive Pain
M4206	Juvenile osteochondrosis of spine, lumbar region	Arthritis Joint Spine and Hips	Nociceptive Pain
M4207	Juvenile osteochondrosis of spine, lumbosacral region	Arthritis Joint Spine and Hips	Nociceptive Pain
M4208	Juvenile osteochondrosis of spine, sacr/sacrocygl region	Arthritis Joint Spine and Hips	Nociceptive Pain
M4209	Juvenile osteochondrosis of spine, multiple sites in spine	Arthritis Joint Spine and Hips	Nociceptive Pain
M421	Adult osteochondrosis of spine	Arthritis Joint Spine and Hips	Nociceptive Pain
M4210	Adult osteochondrosis of spine, site unspecified	Arthritis Joint Spine and Hips	Nociceptive Pain
M4211	Adult osteochondrosis of spine, occipt-atlan-ax region	Arthritis Joint Spine and Hips	Nociceptive Pain

© 2024 Peterson MD et al. *JAMA Neurology.*

M4212	Adult osteochondrosis of spine, cervical region	Arthritis Joint Spine and Hips	Nociceptive Pain
M4213	Adult osteochondrosis of spine, cervicothoracic region	Arthritis Joint Spine and Hips	Nociceptive Pain
M4214	Adult osteochondrosis of spine, thoracic region	Arthritis Joint Spine and Hips	Nociceptive Pain
M4215	Adult osteochondrosis of spine, thoracolumbar region	Arthritis Joint Spine and Hips	Nociceptive Pain
M4216	Adult osteochondrosis of spine, lumbar region	Arthritis Joint Spine and Hips	Nociceptive Pain
M4217	Adult osteochondrosis of spine, lumbosacral region	Arthritis Joint Spine and Hips	Nociceptive Pain
M4218	Adult osteochondrosis of spine, sacr/sacrocygl region	Arthritis Joint Spine and Hips	Nociceptive Pain
M4219	Adult osteochondrosis of spine, multiple sites in spine	Arthritis Joint Spine and Hips	Nociceptive Pain
M429	Spinal osteochondrosis, unspecified	Arthritis Joint Other	Nociceptive Pain
M4300	Spondylolysis, site unspecified	Back Pain Other/Unspecified	Nociceptive Pain
M4301	Spondylolysis, occipito-atlanto-axial region	Neck Pain	Nociceptive Pain
M4302	Spondylolysis, cervical region	Neck Pain	Nociceptive Pain
M4303	Spondylolysis, cervicothoracic region	Neck Pain	Nociceptive Pain
M4304	Spondylolysis, thoracic region	Back Pain Mid Back	Nociceptive Pain
M4305	Spondylolysis, thoracolumbar region	Back Pain Mid Back	Nociceptive Pain
M4306	Spondylolysis, lumbar region	Back Pain Low Back	Nociceptive Pain
M4307	Spondylolysis, lumbosacral region	Back Pain Low Back	Nociceptive Pain
M4308	Spondylolysis, sacral and sacrococcygeal region	Back Pain Low Back	Nociceptive Pain
M4309	Spondylolysis, multiple sites in spine	Back Pain Other/Unspecified	Nociceptive Pain
M4310	Spondylolisthesis, site unspecified	Back Pain Other/Unspecified	Nociceptive Pain
M4311	Spondylolisthesis, occipito-atlanto-axial region	Neck Pain	Nociceptive Pain
M4312	Spondylolisthesis, cervical region	Neck Pain	Nociceptive Pain
M4313	Spondylolisthesis, cervicothoracic region	Neck Pain	Nociceptive Pain
M4314	Spondylolisthesis, thoracic region	Back Pain Mid Back	Nociceptive Pain
M4315	Spondylolisthesis, thoracolumbar region	Back Pain Mid Back	Nociceptive Pain
M4316	Spondylolisthesis, lumbar region	Back Pain Low Back	Nociceptive Pain
M4317	Spondylolisthesis, lumbosacral region	Back Pain Low Back	Nociceptive Pain
M4318	Spondylolisthesis, sacral and sacrococcygeal region	Back Pain Low Back	Nociceptive Pain
M4319	Spondylolisthesis, multiple sites in spine	Back Pain Other/Unspecified	Nociceptive Pain
M4327	Fusion of spine, lumbosacral region	Back Pain Low Back	Nociceptive Pain
M4328	Fusion of spine, sacral and sacrococcygeal region	Back Pain Low Back	Nociceptive Pain
M438	Other specified deforming dorsopathies	Arthritis Joint Other	Nociceptive Pain
M439	Deforming dorsopathy, unspecified	Arthritis Joint Other	Nociceptive Pain
M46	Other inflammatory spondylopathies	Neck Pain	Nociceptive Pain
M4640	Discitis, unspecified, site unspecified	Back Pain Other/Unspecified	Nociceptive Pain
M4644	Discitis, unspecified, thoracic region	Back Pain Mid Back	Nociceptive Pain
M4645	Discitis, unspecified, thoracolumbar region	Back Pain Mid Back	Nociceptive Pain

© 2024 Peterson MD et al. *JAMA Neurology.*

M4646	Discitis, unspecified, lumbar region	Back Pain Low Back	Nociceptive Pain
M4647	Discitis, unspecified, lumbosacral region	Back Pain Low Back	Nociceptive Pain
M4648	Discitis, unspecified, sacral and sacrococcygeal region	Back Pain Low Back	Nociceptive Pain
M4710	Other spondylosis with myelopathy, site unspecified	Back Pain Other/Unspecified	Nociceptive Pain
M4711	Oth spondylosis w myelopathy, occipito-atlanto-axial region	Neck Pain	Nociceptive Pain
M4712	Other spondylosis with myelopathy, cervical region	Neck Pain	Nociceptive Pain
M4713	Other spondylosis with myelopathy, cervicothoracic region	Neck Pain	Nociceptive Pain
M4714	Other spondylosis with myelopathy, thoracic region	Back Pain Mid Back	Nociceptive Pain
M4715	Other spondylosis with myelopathy, thoracolumbar region	Back Pain Mid Back	Nociceptive Pain
M4716	Other spondylosis with myelopathy, lumbar region	Back Pain Low Back	Nociceptive Pain
M47814	Spondylosis w/o myelopathy or radiculopathy, thoracic region	Back Pain Mid Back	Nociceptive Pain
M47815	Spondyls w/o myelopathy or radiculopathy, thoracolum region	Back Pain Mid Back	Nociceptive Pain
M47816	Spondylosis w/o myelopathy or radiculopathy, lumbar region	Back Pain Low Back	Nociceptive Pain
M47817	Spondyls w/o myelopathy or radiculopathy, lumbosacr region	Back Pain Low Back	Nociceptive Pain
M47818	Spondyls w/o myelpath or radiculopathy, sacr/sacrocygl rgn	Back Pain Low Back	Nociceptive Pain
M47819	Spondylosis without myelopathy or radiculopathy, site unsp	Back Pain Other/Unspecified	Nociceptive Pain
M4800	Spinal stenosis, site unspecified	Back Pain Other/Unspecified	Nociceptive Pain
M4801	Spinal stenosis, occipito-atlanto-axial region	Neck Pain	Nociceptive Pain
M4802	Spinal stenosis, cervical region	Neck Pain	Nociceptive Pain
M4803	Spinal stenosis, cervicothoracic region	Neck Pain	Nociceptive Pain
M4804	Spinal stenosis, thoracic region	Back Pain Mid Back	Nociceptive Pain
M4805	Spinal stenosis, thoracolumbar region	Back Pain Mid Back	Nociceptive Pain
M4806	Spinal stenosis, lumbar region	Back Pain Low Back	Nociceptive Pain
M48061	Spinal stenosis, lumbar region without neurogenic claud	Back Pain Low Back	Nociceptive Pain
M48062	Spinal stenosis, lumbar region with neurogenic claudication	Back Pain Low Back	Nociceptive Pain
M4807	Spinal stenosis, lumbosacral region	Back Pain Low Back	Nociceptive Pain
M4808	Spinal stenosis, sacral and sacrococcygeal region	Back Pain Low Back	Nociceptive Pain
M4810	Ankylosing hyperostosis [Forestier], site unspecified	Back Pain Other/Unspecified	Nociceptive Pain
M4814	Ankylosing hyperostosis [Forestier], thoracic region	Back Pain Mid Back	Nociceptive Pain
M4815	Ankylosing hyperostosis [Forestier], thoracolumbar region	Back Pain Mid Back	Nociceptive Pain
M4816	Ankylosing hyperostosis [Forestier], lumbar region	Back Pain Low Back	Nociceptive Pain
M4817	Ankylosing hyperostosis [Forestier], lumbosacral region	Back Pain Low Back	Nociceptive Pain
M4818	Ankylosing hyperostosis, sacral and sacrococcygeal region	Back Pain Low Back	Nociceptive Pain
M4819	Ankylosing hyperostosis [Forestier], multiple sites in spine	Back Pain Other/Unspecified	Nociceptive Pain
M4820	Kissing spine, site unspecified	Back Pain Other/Unspecified	Nociceptive Pain
M4824	Kissing spine, thoracic region	Back Pain Mid Back	Nociceptive Pain
M4825	Kissing spine, thoracolumbar region	Back Pain Mid Back	Nociceptive Pain

© 2024 Peterson MD et al. *JAMA Neurology.*

M4826	Kissing spine, lumbar region	Back Pain Low Back	Nociceptive Pain
M4827	Kissing spine, lumbosacral region	Back Pain Low Back	Nociceptive Pain
M4830	Traumatic spondylopathy, site unspecified	Back Pain Other/Unspecified	Nociceptive Pain
M4834	Traumatic spondylopathy, thoracic region	Back Pain Mid Back	Nociceptive Pain
M4835	Traumatic spondylopathy, thoracolumbar region	Back Pain Mid Back	Nociceptive Pain
M4836	Traumatic spondylopathy, lumbar region	Back Pain Low Back	Nociceptive Pain
M4837	Traumatic spondylopathy, lumbosacral region	Back Pain Low Back	Nociceptive Pain
M4838	Traumatic spondylopathy, sacral and sacrococcygeal region	Back Pain Low Back	Nociceptive Pain
M4840XA	Fatigue fracture of vertebra, site unsp, init for fx	Arthritis Joint Spine and Hips	Nociceptive Pain
M4841XA	Fatigue fracture of vertebra, occipt-atlan-ax region, init	Arthritis Joint Spine and Hips	Nociceptive Pain
M4842XA	Fatigue fracture of vertebra, cervical region, init for fx	Arthritis Joint Spine and Hips	Nociceptive Pain
M4843XA	Fatigue fracture of vertebra, cervicothoracic region, init	Arthritis Joint Spine and Hips	Nociceptive Pain
M4844XA	Fatigue fracture of vertebra, thoracic region, init for fx	Arthritis Joint Spine and Hips	Nociceptive Pain
M4845XA	Fatigue fracture of vertebra, thoracolumbar region, init	Arthritis Joint Spine and Hips	Nociceptive Pain
M4846XA	Fatigue fracture of vertebra, lumbar region, init for fx	Arthritis Joint Spine and Hips	Nociceptive Pain
M4847XA	Fatigue fracture of vertebra, lumbosacral region, init	Arthritis Joint Spine and Hips	Nociceptive Pain
M4848XA	Fatigue fracture of vertebra, sacr/sacrocygl region, init	Arthritis Joint Spine and Hips	Nociceptive Pain
M4850XA	Collapsed vertebra, NEC, site unsp, init	Arthritis Joint Spine and Hips	Nociceptive Pain
M4851XA	Collapsed vertebra, NEC, occipito-atlanto-axial region, init	Arthritis Joint Spine and Hips	Nociceptive Pain
M4852XA	Collapsed vertebra, NEC, cervical region, init	Arthritis Joint Spine and Hips	Nociceptive Pain
M4853XA	Collapsed vertebra, NEC, cervicothoracic region, init	Arthritis Joint Spine and Hips	Nociceptive Pain
M4854XA	Collapsed vertebra, NEC, thoracic region, init	Arthritis Joint Spine and Hips	Nociceptive Pain
M4855XA	Collapsed vertebra, NEC, thoracolumbar region, init	Arthritis Joint Spine and Hips	Nociceptive Pain
M4856XA	Collapsed vertebra, NEC, lumbar region, init	Arthritis Joint Spine and Hips	Nociceptive Pain
M4857XA	Collapsed vertebra, NEC, lumbosacral region, init	Arthritis Joint Spine and Hips	Nociceptive Pain
M4858XA	Collapsed vertebra, NEC, sacr/sacrocygl region, init	Arthritis Joint Spine and Hips	Nociceptive Pain
M489	Spondylopathy, unspecified	Back Pain Other/Unspecified	Nociceptive Pain
M500	Cervical disc disorder with myelopathy	Neck Pain	Nociceptive Pain
M502	Other cervical disc displacement	Neck Pain	Nociceptive Pain
M508	Other cervical disc disorders	Neck Pain	Nociceptive Pain
M509	Cervical disc disorder, unspecified	Neck Pain	Nociceptive Pain
M5104	Intervertebral disc disorders w myelopathy, thoracic region	Back Pain Mid Back	Nociceptive Pain
M5105	Intvrt disc disorders w myelopathy, thoracolumbar region	Back Pain Mid Back	Nociceptive Pain
M5106	Intervertebral disc disorders with myelopathy, lumbar region	Back Pain Low Back	Nociceptive Pain
M5144	Schmorl's nodes, thoracic region	Back Pain Mid Back	Nociceptive Pain
M5145	Schmorl's nodes, thoracolumbar region	Back Pain Mid Back	Nociceptive Pain
M5146	Schmorl's nodes, lumbar region	Back Pain Low Back	Nociceptive Pain

© 2024 Peterson MD et al. *JAMA Neurology.*

M5147	Schmorl's nodes, lumbosacral region	Back Pain Low Back	Nociceptive Pain
M5184	Other intervertebral disc disorders, thoracic region	Back Pain Mid Back	Nociceptive Pain
M5185	Other intervertebral disc disorders, thoracolumbar region	Back Pain Mid Back	Nociceptive Pain
M5186	Other intervertebral disc disorders, lumbar region	Back Pain Low Back	Nociceptive Pain
M5187	Other intervertebral disc disorders, lumbosacral region	Back Pain Low Back	Nociceptive Pain
M519	Unsp thoracic, thoracolum and lumbosacr intvrt disc disorder	Back Pain Mid Back	Nociceptive Pain
M530	Cervicocranial syndrome	Neck Pain	Nociceptive Pain
M531	Cervicobrachial syndrome	Neck Pain	Nociceptive Pain
M532X4	Spinal instabilities, thoracic region	Back Pain Mid Back	Nociceptive Pain
M532X5	Spinal instabilities, thoracolumbar region	Back Pain Mid Back	Nociceptive Pain
M532X6	Spinal instabilities, lumbar region	Back Pain Low Back	Nociceptive Pain
M532X7	Spinal instabilities, lumbosacral region	Back Pain Low Back	Nociceptive Pain
M532X8	Spinal instabilities, sacral and sacrococcygeal region	Back Pain Low Back	Nociceptive Pain
M532X9	Spinal instabilities, site unspecified	Back Pain Other/Unspecified	Nociceptive Pain
M533	Sacrococcygeal disorders, not elsewhere classified	Back Pain Low Back	Nociceptive Pain
M5382	Other specified dorsopathies, cervical region	Neck Pain	Nociceptive Pain
M539	Dorsopathy, unspecified	Back Pain Other/Unspecified	Nociceptive Pain
M5401	Panniculitis aff regions of neck/bk, occipt-atlan-ax region	Neck Pain	Nociceptive Pain
M5402	Panniculitis affecting regions of neck/bk, cervical region	Neck Pain	Nociceptive Pain
M5403	Panniculitis aff regions of neck/bk, cervicothor region	Neck Pain	Nociceptive Pain
M5404	Panniculitis affecting regions of neck/bk, thoracic region	Back Pain Mid Back	Nociceptive Pain
M5405	Panniculitis affecting regions of neck/bk, thoracolum region	Back Pain Mid Back	Nociceptive Pain
M5406	Panniculitis affecting regions of neck/bk, lumbar region	Back Pain Low Back	Nociceptive Pain
M5407	Panniculitis affecting regions of neck/bk, lumbosacr region	Back Pain Low Back	Nociceptive Pain
M5408	Panniculitis aff regions of neck/bk, sacr/sacrocygl region	Back Pain Low Back	Nociceptive Pain
M542	Cervicalgia	Neck Pain	Nociceptive Pain
M543	Sciatica	Back Pain Low Back	Nociceptive Pain
M5430	Sciatica, unspecified side	Back Pain Low Back	Nociceptive Pain
M5431	Sciatica, right side	Back Pain Low Back	Nociceptive Pain
M5432	Sciatica, left side	Back Pain Low Back	Nociceptive Pain
M546	Pain in thoracic spine	Back Pain Mid Back	Nociceptive Pain
M5489	Other dorsalgia	Back Pain Other/Unspecified	Nociceptive Pain
M549	Dorsalgia, unspecified	Back Pain Other/Unspecified	Nociceptive Pain
M60	Myositis	Arthritis Joint Other	Nociceptive Pain
M600	Infective myositis	Arthritis Joint Other	Nociceptive Pain
M6000	Infective myositis, unspecified site	Arthritis Joint Other	Nociceptive Pain
M60000	Infective myositis, unspecified right arm	Arthritis Joint Upper Limb	Nociceptive Pain

© 2024 Peterson MD et al. *JAMA Neurology.*

M60001	Infective myositis, unspecified left arm	Arthritis Joint Upper Limb	Nociceptive Pain
M60002	Infective myositis, unspecified arm	Arthritis Joint Upper Limb	Nociceptive Pain
M60003	Infective myositis, unspecified right leg	Arthritis Joint Lower Limb	Nociceptive Pain
M60004	Infective myositis, unspecified left leg	Arthritis Joint Lower Limb	Nociceptive Pain
M60005	Infective myositis, unspecified leg	Arthritis Joint Lower Limb	Nociceptive Pain
M60009	Infective myositis, unspecified site	Arthritis Joint Other	Nociceptive Pain
M6001	Infective myositis, shoulder	Arthritis Joint Upper Limb	Nociceptive Pain
M60011	Infective myositis, right shoulder	Arthritis Joint Upper Limb	Nociceptive Pain
M60012	Infective myositis, left shoulder	Arthritis Joint Upper Limb	Nociceptive Pain
M60019	Infective myositis, unspecified shoulder	Arthritis Joint Upper Limb	Nociceptive Pain
M6002	Infective myositis, upper arm	Arthritis Joint Upper Limb	Nociceptive Pain
M60021	Infective myositis, right upper arm	Arthritis Joint Upper Limb	Nociceptive Pain
M60022	Infective myositis, left upper arm	Arthritis Joint Upper Limb	Nociceptive Pain
M60029	Infective myositis, unspecified upper arm	Arthritis Joint Upper Limb	Nociceptive Pain
M6003	Infective myositis, forearm	Arthritis Joint Upper Limb	Nociceptive Pain
M60031	Infective myositis, right forearm	Arthritis Joint Upper Limb	Nociceptive Pain
M60032	Infective myositis, left forearm	Arthritis Joint Upper Limb	Nociceptive Pain
M60039	Infective myositis, unspecified forearm	Arthritis Joint Upper Limb	Nociceptive Pain
M6004	Infective myositis, hand and fingers	Arthritis Joint Upper Limb	Nociceptive Pain
M60041	Infective myositis, right hand	Arthritis Joint Upper Limb	Nociceptive Pain
M60042	Infective myositis, left hand	Arthritis Joint Upper Limb	Nociceptive Pain
M60043	Infective myositis, unspecified hand	Arthritis Joint Upper Limb	Nociceptive Pain
M60044	Infective myositis, right finger(s)	Arthritis Joint Upper Limb	Nociceptive Pain
M60045	Infective myositis, left finger(s)	Arthritis Joint Upper Limb	Nociceptive Pain
M60046	Infective myositis, unspecified finger(s)	Arthritis Joint Upper Limb	Nociceptive Pain
M6005	Infective myositis, thigh	Arthritis Joint Lower Limb	Nociceptive Pain
M60051	Infective myositis, right thigh	Arthritis Joint Lower Limb	Nociceptive Pain
M60052	Infective myositis, left thigh	Arthritis Joint Lower Limb	Nociceptive Pain
M60059	Infective myositis, unspecified thigh	Arthritis Joint Lower Limb	Nociceptive Pain
M6006	Infective myositis, lower leg	Arthritis Joint Lower Limb	Nociceptive Pain
M60061	Infective myositis, right lower leg	Arthritis Joint Lower Limb	Nociceptive Pain
M60062	Infective myositis, left lower leg	Arthritis Joint Lower Limb	Nociceptive Pain
M60069	Infective myositis, unspecified lower leg	Arthritis Joint Lower Limb	Nociceptive Pain
M6007	Infective myositis, ankle, foot and toes	Arthritis Joint Lower Limb	Nociceptive Pain
M60070	Infective myositis, right ankle	Arthritis Joint Lower Limb	Nociceptive Pain
M60071	Infective myositis, left ankle	Arthritis Joint Lower Limb	Nociceptive Pain
M60072	Infective myositis, unspecified ankle	Arthritis Joint Lower Limb	Nociceptive Pain

© 2024 Peterson MD et al. *JAMA Neurology.*

M60073	Infective myositis, right foot	Arthritis Joint Lower Limb	Nociceptive Pain
M60074	Infective myositis, left foot	Arthritis Joint Lower Limb	Nociceptive Pain
M60075	Infective myositis, unspecified foot	Arthritis Joint Lower Limb	Nociceptive Pain
M60076	Infective myositis, right toe(s)	Arthritis Joint Lower Limb	Nociceptive Pain
M60077	Infective myositis, left toe(s)	Arthritis Joint Lower Limb	Nociceptive Pain
M60078	Infective myositis, unspecified toe(s)	Arthritis Joint Lower Limb	Nociceptive Pain
M6008	Infective myositis, other site	Arthritis Joint Other	Nociceptive Pain
M6009	Infective myositis, multiple sites	Arthritis Joint Other	Nociceptive Pain
M601	Interstitial myositis	Arthritis Joint Other	Nociceptive Pain
M6010	Interstitial myositis of unspecified site	Arthritis Joint Other	Nociceptive Pain
M6011	Interstitial myositis, shoulder	Arthritis Joint Upper Limb	Nociceptive Pain
M60111	Interstitial myositis, right shoulder	Arthritis Joint Upper Limb	Nociceptive Pain
M60112	Interstitial myositis, left shoulder	Arthritis Joint Upper Limb	Nociceptive Pain
M60119	Interstitial myositis, unspecified shoulder	Arthritis Joint Upper Limb	Nociceptive Pain
M6012	Interstitial myositis, upper arm	Arthritis Joint Upper Limb	Nociceptive Pain
M60121	Interstitial myositis, right upper arm	Arthritis Joint Upper Limb	Nociceptive Pain
M60122	Interstitial myositis, left upper arm	Arthritis Joint Upper Limb	Nociceptive Pain
M60129	Interstitial myositis, unspecified upper arm	Arthritis Joint Upper Limb	Nociceptive Pain
M6013	Interstitial myositis, forearm	Arthritis Joint Upper Limb	Nociceptive Pain
M60131	Interstitial myositis, right forearm	Arthritis Joint Upper Limb	Nociceptive Pain
M60132	Interstitial myositis, left forearm	Arthritis Joint Upper Limb	Nociceptive Pain
M60139	Interstitial myositis, unspecified forearm	Arthritis Joint Upper Limb	Nociceptive Pain
M6014	Interstitial myositis, hand	Arthritis Joint Upper Limb	Nociceptive Pain
M60141	Interstitial myositis, right hand	Arthritis Joint Upper Limb	Nociceptive Pain
M60142	Interstitial myositis, left hand	Arthritis Joint Upper Limb	Nociceptive Pain
M60149	Interstitial myositis, unspecified hand	Arthritis Joint Upper Limb	Nociceptive Pain
M6015	Interstitial myositis, thigh	Arthritis Joint Lower Limb	Nociceptive Pain
M60151	Interstitial myositis, right thigh	Arthritis Joint Lower Limb	Nociceptive Pain
M60152	Interstitial myositis, left thigh	Arthritis Joint Lower Limb	Nociceptive Pain
M60159	Interstitial myositis, unspecified thigh	Arthritis Joint Lower Limb	Nociceptive Pain
M6016	Interstitial myositis, lower leg	Arthritis Joint Lower Limb	Nociceptive Pain
M60161	Interstitial myositis, right lower leg	Arthritis Joint Lower Limb	Nociceptive Pain
M60162	Interstitial myositis, left lower leg	Arthritis Joint Lower Limb	Nociceptive Pain
M60169	Interstitial myositis, unspecified lower leg	Arthritis Joint Lower Limb	Nociceptive Pain
M6017	Interstitial myositis, ankle and foot	Arthritis Joint Lower Limb	Nociceptive Pain
M60171	Interstitial myositis, right ankle and foot	Arthritis Joint Lower Limb	Nociceptive Pain
M60172	Interstitial myositis, left ankle and foot	Arthritis Joint Lower Limb	Nociceptive Pain

© 2024 Peterson MD et al. *JAMA Neurology.*

M60179	Interstitial myositis, unspecified ankle and foot	Arthritis Joint Lower Limb	Nociceptive Pain
M6018	Interstitial myositis, other site	Arthritis Joint Other	Nociceptive Pain
M6019	Interstitial myositis, multiple sites	Arthritis Joint Other	Nociceptive Pain
M602	Foreign body granuloma of soft tissue, NEC	Arthritis Joint Other	Nociceptive Pain
M6020	Foreign body granuloma of soft tissue, NEC, unsp site	Arthritis Joint Other	Nociceptive Pain
M6021	Foreign body granuloma of soft tissue, NEC, shoulder	Arthritis Joint Upper Limb	Nociceptive Pain
M60211	Foreign body granuloma of soft tissue, NEC, right shoulder	Arthritis Joint Upper Limb	Nociceptive Pain
M60212	Foreign body granuloma of soft tissue, NEC, left shoulder	Arthritis Joint Upper Limb	Nociceptive Pain
M60219	Foreign body granuloma of soft tissue, NEC, unsp shoulder	Arthritis Joint Upper Limb	Nociceptive Pain
M6022	Foreign body granuloma of soft tissue, NEC, upper arm	Arthritis Joint Upper Limb	Nociceptive Pain
M60221	Foreign body granuloma of soft tissue, NEC, right upper arm	Arthritis Joint Upper Limb	Nociceptive Pain
M60222	Foreign body granuloma of soft tissue, NEC, left upper arm	Arthritis Joint Upper Limb	Nociceptive Pain
M60229	Foreign body granuloma of soft tissue, NEC, unsp upper arm	Arthritis Joint Upper Limb	Nociceptive Pain
M6023	Foreign body granuloma of soft tissue, NEC, forearm	Arthritis Joint Upper Limb	Nociceptive Pain
M60231	Foreign body granuloma of soft tissue, NEC, right forearm	Arthritis Joint Upper Limb	Nociceptive Pain
M60232	Foreign body granuloma of soft tissue, NEC, left forearm	Arthritis Joint Upper Limb	Nociceptive Pain
M60239	Foreign body granuloma of soft tissue, NEC, unsp forearm	Arthritis Joint Upper Limb	Nociceptive Pain
M6024	Foreign body granuloma of soft tissue, NEC, hand	Arthritis Joint Upper Limb	Nociceptive Pain
M60241	Foreign body granuloma of soft tissue, NEC, right hand	Arthritis Joint Upper Limb	Nociceptive Pain
M60242	Foreign body granuloma of soft tissue, NEC, left hand	Arthritis Joint Upper Limb	Nociceptive Pain
M60249	Foreign body granuloma of soft tissue, NEC, unsp hand	Arthritis Joint Upper Limb	Nociceptive Pain
M6025	Foreign body granuloma of soft tissue, NEC, thigh	Arthritis Joint Lower Limb	Nociceptive Pain
M60251	Foreign body granuloma of soft tissue, NEC, right thigh	Arthritis Joint Lower Limb	Nociceptive Pain
M60252	Foreign body granuloma of soft tissue, NEC, left thigh	Arthritis Joint Lower Limb	Nociceptive Pain
M60259	Foreign body granuloma of soft tissue, NEC, unsp thigh	Arthritis Joint Lower Limb	Nociceptive Pain
M6026	Foreign body granuloma of soft tissue, NEC, lower leg	Arthritis Joint Lower Limb	Nociceptive Pain
M60261	Foreign body granuloma of soft tissue, NEC, right lower leg	Arthritis Joint Lower Limb	Nociceptive Pain
M60262	Foreign body granuloma of soft tissue, NEC, left lower leg	Arthritis Joint Lower Limb	Nociceptive Pain
M60269	Foreign body granuloma of soft tissue, NEC, unsp lower leg	Arthritis Joint Lower Limb	Nociceptive Pain
M6027	Foreign body granuloma of soft tissue, NEC, ankle and foot	Arthritis Joint Lower Limb	Nociceptive Pain
M60271	Foreign body granuloma of soft tissue, NEC, right ank/ft	Arthritis Joint Lower Limb	Nociceptive Pain
M60272	Foreign body granuloma of soft tissue, NEC, left ank/ft	Arthritis Joint Lower Limb	Nociceptive Pain
M60279	Foreign body granuloma of soft tissue, NEC, unsp ank/ft	Arthritis Joint Lower Limb	Nociceptive Pain
M6028	Foreign body granuloma of soft tissue, NEC, oth site	Arthritis Joint Other	Nociceptive Pain
M61	Calcification and ossification of muscle	Arthritis Joint Other	Nociceptive Pain
M610	Myositis ossificans traumatica	Arthritis Joint Other	Nociceptive Pain
M6100	Myositis ossificans traumatica, unspecified site	Arthritis Joint Other	Nociceptive Pain

© 2024 Peterson MD et al. *JAMA Neurology.*

M6101	Myositis ossificans traumatica, shoulder	Arthritis Joint Upper Limb	Nociceptive Pain
M61011	Myositis ossificans traumatica, right shoulder	Arthritis Joint Upper Limb	Nociceptive Pain
M61012	Myositis ossificans traumatica, left shoulder	Arthritis Joint Upper Limb	Nociceptive Pain
M61019	Myositis ossificans traumatica, unspecified shoulder	Arthritis Joint Upper Limb	Nociceptive Pain
M6102	Myositis ossificans traumatica, upper arm	Arthritis Joint Upper Limb	Nociceptive Pain
M61021	Myositis ossificans traumatica, right upper arm	Arthritis Joint Upper Limb	Nociceptive Pain
M61022	Myositis ossificans traumatica, left upper arm	Arthritis Joint Upper Limb	Nociceptive Pain
M61029	Myositis ossificans traumatica, unspecified upper arm	Arthritis Joint Upper Limb	Nociceptive Pain
M6103	Myositis ossificans traumatica, forearm	Arthritis Joint Upper Limb	Nociceptive Pain
M61031	Myositis ossificans traumatica, right forearm	Arthritis Joint Upper Limb	Nociceptive Pain
M61032	Myositis ossificans traumatica, left forearm	Arthritis Joint Upper Limb	Nociceptive Pain
M61039	Myositis ossificans traumatica, unspecified forearm	Arthritis Joint Upper Limb	Nociceptive Pain
M6104	Myositis ossificans traumatica, hand	Arthritis Joint Upper Limb	Nociceptive Pain
M61041	Myositis ossificans traumatica, right hand	Arthritis Joint Upper Limb	Nociceptive Pain
M61042	Myositis ossificans traumatica, left hand	Arthritis Joint Upper Limb	Nociceptive Pain
M61049	Myositis ossificans traumatica, unspecified hand	Arthritis Joint Upper Limb	Nociceptive Pain
M6105	Myositis ossificans traumatica, thigh	Arthritis Joint Lower Limb	Nociceptive Pain
M61051	Myositis ossificans traumatica, right thigh	Arthritis Joint Lower Limb	Nociceptive Pain
M61052	Myositis ossificans traumatica, left thigh	Arthritis Joint Lower Limb	Nociceptive Pain
M61059	Myositis ossificans traumatica, unspecified thigh	Arthritis Joint Lower Limb	Nociceptive Pain
M6106	Myositis ossificans traumatica, lower leg	Arthritis Joint Lower Limb	Nociceptive Pain
M61061	Myositis ossificans traumatica, right lower leg	Arthritis Joint Lower Limb	Nociceptive Pain
M61062	Myositis ossificans traumatica, left lower leg	Arthritis Joint Lower Limb	Nociceptive Pain
M61069	Myositis ossificans traumatica, unspecified lower leg	Arthritis Joint Lower Limb	Nociceptive Pain
M6107	Myositis ossificans traumatica, ankle and foot	Arthritis Joint Lower Limb	Nociceptive Pain
M61071	Myositis ossificans traumatica, right ankle and foot	Arthritis Joint Lower Limb	Nociceptive Pain
M61072	Myositis ossificans traumatica, left ankle and foot	Arthritis Joint Lower Limb	Nociceptive Pain
M61079	Myositis ossificans traumatica, unspecified ankle and foot	Arthritis Joint Lower Limb	Nociceptive Pain
M6108	Myositis ossificans traumatica, other site	Arthritis Joint Other	Nociceptive Pain
M6109	Myositis ossificans traumatica, multiple sites	Arthritis Joint Other	Nociceptive Pain
M611	Myositis ossificans progressiva	Arthritis Joint Other	Nociceptive Pain
M6110	Myositis ossificans progressiva, unspecified site	Arthritis Joint Other	Nociceptive Pain
M6111	Myositis ossificans progressiva, shoulder	Arthritis Joint Upper Limb	Nociceptive Pain
M61111	Myositis ossificans progressiva, right shoulder	Arthritis Joint Upper Limb	Nociceptive Pain
M61112	Myositis ossificans progressiva, left shoulder	Arthritis Joint Upper Limb	Nociceptive Pain
M61119	Myositis ossificans progressiva, unspecified shoulder	Arthritis Joint Upper Limb	Nociceptive Pain
M6112	Myositis ossificans progressiva, upper arm	Arthritis Joint Upper Limb	Nociceptive Pain

© 2024 Peterson MD et al. *JAMA Neurology.*

M61121	Myositis ossificans progressiva, right upper arm	Arthritis Joint Upper Limb	Nociceptive Pain
M61122	Myositis ossificans progressiva, left upper arm	Arthritis Joint Upper Limb	Nociceptive Pain
M61129	Myositis ossificans progressiva, unspecified arm	Arthritis Joint Upper Limb	Nociceptive Pain
M6113	Myositis ossificans progressiva, forearm	Arthritis Joint Upper Limb	Nociceptive Pain
M61131	Myositis ossificans progressiva, right forearm	Arthritis Joint Upper Limb	Nociceptive Pain
M61132	Myositis ossificans progressiva, left forearm	Arthritis Joint Upper Limb	Nociceptive Pain
M61139	Myositis ossificans progressiva, unspecified forearm	Arthritis Joint Upper Limb	Nociceptive Pain
M6114	Myositis ossificans progressiva, hand and finger(s)	Arthritis Joint Upper Limb	Nociceptive Pain
M61141	Myositis ossificans progressiva, right hand	Arthritis Joint Upper Limb	Nociceptive Pain
M61142	Myositis ossificans progressiva, left hand	Arthritis Joint Upper Limb	Nociceptive Pain
M61143	Myositis ossificans progressiva, unspecified hand	Arthritis Joint Upper Limb	Nociceptive Pain
M61144	Myositis ossificans progressiva, right finger(s)	Arthritis Joint Upper Limb	Nociceptive Pain
M61145	Myositis ossificans progressiva, left finger(s)	Arthritis Joint Upper Limb	Nociceptive Pain
M61146	Myositis ossificans progressiva, unspecified finger(s)	Arthritis Joint Upper Limb	Nociceptive Pain
M6115	Myositis ossificans progressiva, thigh	Arthritis Joint Lower Limb	Nociceptive Pain
M61151	Myositis ossificans progressiva, right thigh	Arthritis Joint Lower Limb	Nociceptive Pain
M61152	Myositis ossificans progressiva, left thigh	Arthritis Joint Lower Limb	Nociceptive Pain
M61159	Myositis ossificans progressiva, unspecified thigh	Arthritis Joint Lower Limb	Nociceptive Pain
M6116	Myositis ossificans progressiva, lower leg	Arthritis Joint Lower Limb	Nociceptive Pain
M61161	Myositis ossificans progressiva, right lower leg	Arthritis Joint Lower Limb	Nociceptive Pain
M61162	Myositis ossificans progressiva, left lower leg	Arthritis Joint Lower Limb	Nociceptive Pain
M61169	Myositis ossificans progressiva, unspecified lower leg	Arthritis Joint Lower Limb	Nociceptive Pain
M6117	Myositis ossificans progressiva, ankle, foot and toe(s)	Arthritis Joint Lower Limb	Nociceptive Pain
M61171	Myositis ossificans progressiva, right ankle	Arthritis Joint Lower Limb	Nociceptive Pain
M61172	Myositis ossificans progressiva, left ankle	Arthritis Joint Lower Limb	Nociceptive Pain
M61173	Myositis ossificans progressiva, unspecified ankle	Arthritis Joint Lower Limb	Nociceptive Pain
M61174	Myositis ossificans progressiva, right foot	Arthritis Joint Lower Limb	Nociceptive Pain
M61175	Myositis ossificans progressiva, left foot	Arthritis Joint Lower Limb	Nociceptive Pain
M61176	Myositis ossificans progressiva, unspecified foot	Arthritis Joint Lower Limb	Nociceptive Pain
M61177	Myositis ossificans progressiva, right toe(s)	Arthritis Joint Lower Limb	Nociceptive Pain
M61178	Myositis ossificans progressiva, left toe(s)	Arthritis Joint Lower Limb	Nociceptive Pain
M61179	Myositis ossificans progressiva, unspecified toe(s)	Arthritis Joint Lower Limb	Nociceptive Pain
M6118	Myositis ossificans progressiva, other site	Arthritis Joint Other	Nociceptive Pain
M6119	Myositis ossificans progressiva, multiple sites	Arthritis Joint Other	Nociceptive Pain
M612	Paralytic calcification and ossification of muscle	Arthritis Joint Other	Nociceptive Pain
M6120	Paralytic calcifcn and ossification of muscle, unsp site	Arthritis Joint Other	Nociceptive Pain
M6121	Paralytic calcification and ossification of muscle, shoulder	Arthritis Joint Upper Limb	Nociceptive Pain

© 2024 Peterson MD et al. *JAMA Neurology.*

M61211	Paralytic calcifcn and ossification of muscle, r shoulder	Arthritis Joint Upper Limb	Nociceptive Pain
M61212	Paralytic calcifcn and ossification of muscle, left shoulder	Arthritis Joint Upper Limb	Nociceptive Pain
M61219	Paralytic calcifcn and ossification of muscle, unsp shoulder	Arthritis Joint Upper Limb	Nociceptive Pain
M6122	Paralytic calcifcn and ossification of muscle, upper arm	Arthritis Joint Upper Limb	Nociceptive Pain
M61221	Paralytic calcification and ossification of muscle, r up arm	Arthritis Joint Upper Limb	Nociceptive Pain
M61222	Paralytic calcification and ossification of muscle, l up arm	Arthritis Joint Upper Limb	Nociceptive Pain
M61229	Paralytic calcifcn and ossifictn of muscle, unsp upper arm	Arthritis Joint Upper Limb	Nociceptive Pain
M6123	Paralytic calcification and ossification of muscle, forearm	Arthritis Joint Upper Limb	Nociceptive Pain
M61231	Paralytic calcifcn and ossification of muscle, right forearm	Arthritis Joint Upper Limb	Nociceptive Pain
M61232	Paralytic calcifcn and ossification of muscle, left forearm	Arthritis Joint Upper Limb	Nociceptive Pain
M61239	Paralytic calcifcn and ossification of muscle, unsp forearm	Arthritis Joint Upper Limb	Nociceptive Pain
M6124	Paralytic calcification and ossification of muscle, hand	Arthritis Joint Upper Limb	Nociceptive Pain
M61241	Paralytic calcifcn and ossification of muscle, right hand	Arthritis Joint Upper Limb	Nociceptive Pain
M61242	Paralytic calcifcn and ossification of muscle, left hand	Arthritis Joint Upper Limb	Nociceptive Pain
M61249	Paralytic calcifcn and ossification of muscle, unsp hand	Arthritis Joint Upper Limb	Nociceptive Pain
M6125	Paralytic calcification and ossification of muscle, thigh	Arthritis Joint Lower Limb	Nociceptive Pain
M61251	Paralytic calcifcn and ossification of muscle, right thigh	Arthritis Joint Lower Limb	Nociceptive Pain
M61252	Paralytic calcifcn and ossification of muscle, left thigh	Arthritis Joint Lower Limb	Nociceptive Pain
M61259	Paralytic calcifcn and ossification of muscle, unsp thigh	Arthritis Joint Lower Limb	Nociceptive Pain
M6126	Paralytic calcifcn and ossification of muscle, lower leg	Arthritis Joint Lower Limb	Nociceptive Pain
M61261	Paralytic calcifcn and ossification of muscle, r low leg	Arthritis Joint Lower Limb	Nociceptive Pain
M61262	Paralytic calcifcn and ossification of muscle, l low leg	Arthritis Joint Lower Limb	Nociceptive Pain
M61269	Paralytic calcifcn and ossifictn of muscle, unsp lower leg	Arthritis Joint Lower Limb	Nociceptive Pain
M6127	Paralytic calcification and ossification of muscle, ank/ft	Arthritis Joint Lower Limb	Nociceptive Pain
M61271	Paralytic calcifcn and ossification of muscle, right ank/ft	Arthritis Joint Lower Limb	Nociceptive Pain
M61272	Paralytic calcifcn and ossification of muscle, left ank/ft	Arthritis Joint Lower Limb	Nociceptive Pain
M61279	Paralytic calcifcn and ossification of muscle, unsp ank/ft	Arthritis Joint Lower Limb	Nociceptive Pain
M6128	Paralytic calcification and ossification of muscle, oth site	Arthritis Joint Other	Nociceptive Pain
M6129	Paralytic calcifcn and ossifictn of muscle, multiple sites	Arthritis Joint Other	Nociceptive Pain
M613	Calcification and ossification of muscles associated w burns	Arthritis Joint Other	Nociceptive Pain
M6130	Calcifcn and ossifictn of muscles assoc w burns, unsp site	Arthritis Joint Other	Nociceptive Pain
M6131	Calcifcn and ossifictn of muscles assoc w burns, shoulder	Arthritis Joint Upper Limb	Nociceptive Pain
M61311	Calcifcn and ossifictn of muscles assoc w burns, r shoulder	Arthritis Joint Upper Limb	Nociceptive Pain
M61312	Calcifcn and ossifictn of muscles assoc w burns, l shoulder	Arthritis Joint Upper Limb	Nociceptive Pain
M61319	Calcifcn and ossifictn of muscles assoc w burns, unsp shldr	Arthritis Joint Upper Limb	Nociceptive Pain
M6132	Calcifcn and ossifictn of muscles assoc w burns, upper arm	Arthritis Joint Upper Limb	Nociceptive Pain
M61321	Calcifcn and ossifictn of muscles assoc w burns, r up arm	Arthritis Joint Upper Limb	Nociceptive Pain

© 2024 Peterson MD et al. *JAMA Neurology.*

M61322	Calcifcn and ossifictn of muscles assoc w burns, l up arm	Arthritis Joint Upper Limb	Nociceptive Pain
M61329	Calcifcn and ossifictn of muscles assoc w burns, unsp up arm	Arthritis Joint Upper Limb	Nociceptive Pain
M6133	Calcifcn and ossifictn of muscles assoc w burns, forearm	Arthritis Joint Upper Limb	Nociceptive Pain
M61331	Calcifcn and ossifictn of muscles assoc w burns, r forearm	Arthritis Joint Upper Limb	Nociceptive Pain
M61332	Calcifcn and ossifictn of muscles assoc w burns, l forearm	Arthritis Joint Upper Limb	Nociceptive Pain
M61339	Calcifcn and ossifictn of musc assoc w burns, unsp forearm	Arthritis Joint Upper Limb	Nociceptive Pain
M6134	Calcifcn and ossifictn of muscles associated w burns, hand	Arthritis Joint Upper Limb	Nociceptive Pain
M61341	Calcifcn and ossifictn of muscles assoc w burns, right hand	Arthritis Joint Upper Limb	Nociceptive Pain
M61342	Calcifcn and ossifictn of muscles assoc w burns, left hand	Arthritis Joint Upper Limb	Nociceptive Pain
M61349	Calcifcn and ossifictn of muscles assoc w burns, unsp hand	Arthritis Joint Upper Limb	Nociceptive Pain
M6135	Calcifcn and ossifictn of muscles associated w burns, thigh	Arthritis Joint Lower Limb	Nociceptive Pain
M61351	Calcifcn and ossifictn of muscles assoc w burns, right thigh	Arthritis Joint Lower Limb	Nociceptive Pain
M61352	Calcifcn and ossifictn of muscles assoc w burns, left thigh	Arthritis Joint Lower Limb	Nociceptive Pain
M61359	Calcifcn and ossifictn of muscles assoc w burns, unsp thigh	Arthritis Joint Lower Limb	Nociceptive Pain
M6136	Calcifcn and ossifictn of muscles assoc w burns, lower leg	Arthritis Joint Lower Limb	Nociceptive Pain
M61361	Calcifcn and ossifictn of muscles assoc w burns, r low leg	Arthritis Joint Lower Limb	Nociceptive Pain
M61362	Calcifcn and ossifictn of muscles assoc w burns, l low leg	Arthritis Joint Lower Limb	Nociceptive Pain
M61369	Calcifcn and ossifictn of musc assoc w burns, unsp low leg	Arthritis Joint Lower Limb	Nociceptive Pain
M6137	Calcifcn and ossifictn of muscles associated w burns, ank/ft	Arthritis Joint Lower Limb	Nociceptive Pain
M61371	Calcifcn and ossifictn of musc assoc w burns, right ank/ft	Arthritis Joint Lower Limb	Nociceptive Pain
M61372	Calcifcn and ossifictn of muscles assoc w burns, left ank/ft	Arthritis Joint Lower Limb	Nociceptive Pain
M61379	Calcifcn and ossifictn of muscles assoc w burns, unsp ank/ft	Arthritis Joint Lower Limb	Nociceptive Pain
M6138	Calcifcn and ossifictn of muscles assoc w burns, oth site	Arthritis Joint Other	Nociceptive Pain
M6139	Calcifcn and ossifictn of muscles assoc w burns, mult sites	Arthritis Joint Other	Nociceptive Pain
M614	Other calcification of muscle	Arthritis Joint Other	Nociceptive Pain
M6140	Other calcification of muscle, unspecified site	Arthritis Joint Other	Nociceptive Pain
M6141	Other calcification of muscle, shoulder	Arthritis Joint Upper Limb	Nociceptive Pain
M61411	Other calcification of muscle, right shoulder	Arthritis Joint Upper Limb	Nociceptive Pain
M61412	Other calcification of muscle, left shoulder	Arthritis Joint Upper Limb	Nociceptive Pain
M61419	Other calcification of muscle, unspecified shoulder	Arthritis Joint Upper Limb	Nociceptive Pain
M6142	Other calcification of muscle, upper arm	Arthritis Joint Upper Limb	Nociceptive Pain
M61421	Other calcification of muscle, right upper arm	Arthritis Joint Upper Limb	Nociceptive Pain
M61422	Other calcification of muscle, left upper arm	Arthritis Joint Upper Limb	Nociceptive Pain
M61429	Other calcification of muscle, unspecified upper arm	Arthritis Joint Upper Limb	Nociceptive Pain
M6143	Other calcification of muscle, forearm	Arthritis Joint Upper Limb	Nociceptive Pain
M61431	Other calcification of muscle, right forearm	Arthritis Joint Upper Limb	Nociceptive Pain
M61432	Other calcification of muscle, left forearm	Arthritis Joint Upper Limb	Nociceptive Pain

© 2024 Peterson MD et al. *JAMA Neurology.*

M61439	Other calcification of muscle, unspecified forearm	Arthritis Joint Upper Limb	Nociceptive Pain
M6144	Other calcification of muscle, hand	Arthritis Joint Upper Limb	Nociceptive Pain
M61441	Other calcification of muscle, right hand	Arthritis Joint Upper Limb	Nociceptive Pain
M61442	Other calcification of muscle, left hand	Arthritis Joint Upper Limb	Nociceptive Pain
M61449	Other calcification of muscle, unspecified hand	Arthritis Joint Upper Limb	Nociceptive Pain
M6145	Other calcification of muscle, thigh	Arthritis Joint Lower Limb	Nociceptive Pain
M61451	Other calcification of muscle, right thigh	Arthritis Joint Lower Limb	Nociceptive Pain
M61452	Other calcification of muscle, left thigh	Arthritis Joint Lower Limb	Nociceptive Pain
M61459	Other calcification of muscle, unspecified thigh	Arthritis Joint Lower Limb	Nociceptive Pain
M6146	Other calcification of muscle, lower leg	Arthritis Joint Lower Limb	Nociceptive Pain
M61461	Other calcification of muscle, right lower leg	Arthritis Joint Lower Limb	Nociceptive Pain
M61462	Other calcification of muscle, left lower leg	Arthritis Joint Lower Limb	Nociceptive Pain
M61469	Other calcification of muscle, unspecified lower leg	Arthritis Joint Lower Limb	Nociceptive Pain
M6147	Other calcification of muscle, ankle and foot	Arthritis Joint Lower Limb	Nociceptive Pain
M61471	Other calcification of muscle, right ankle and foot	Arthritis Joint Lower Limb	Nociceptive Pain
M61472	Other calcification of muscle, left ankle and foot	Arthritis Joint Lower Limb	Nociceptive Pain
M61479	Other calcification of muscle, unspecified ankle and foot	Arthritis Joint Lower Limb	Nociceptive Pain
M6148	Other calcification of muscle, other site	Arthritis Joint Other	Nociceptive Pain
M6149	Other calcification of muscle, multiple sites	Arthritis Joint Other	Nociceptive Pain
M615	Other ossification of muscle	Arthritis Joint Other	Nociceptive Pain
M6150	Other ossification of muscle, unspecified site	Arthritis Joint Other	Nociceptive Pain
M6151	Other ossification of muscle, shoulder	Arthritis Joint Upper Limb	Nociceptive Pain
M61511	Other ossification of muscle, right shoulder	Arthritis Joint Upper Limb	Nociceptive Pain
M61512	Other ossification of muscle, left shoulder	Arthritis Joint Upper Limb	Nociceptive Pain
M61519	Other ossification of muscle, unspecified shoulder	Arthritis Joint Upper Limb	Nociceptive Pain
M6152	Other ossification of muscle, upper arm	Arthritis Joint Upper Limb	Nociceptive Pain
M61521	Other ossification of muscle, right upper arm	Arthritis Joint Upper Limb	Nociceptive Pain
M61522	Other ossification of muscle, left upper arm	Arthritis Joint Upper Limb	Nociceptive Pain
M61529	Other ossification of muscle, unspecified upper arm	Arthritis Joint Upper Limb	Nociceptive Pain
M6153	Other ossification of muscle, forearm	Arthritis Joint Upper Limb	Nociceptive Pain
M61531	Other ossification of muscle, right forearm	Arthritis Joint Upper Limb	Nociceptive Pain
M61532	Other ossification of muscle, left forearm	Arthritis Joint Upper Limb	Nociceptive Pain
M61539	Other ossification of muscle, unspecified forearm	Arthritis Joint Upper Limb	Nociceptive Pain
M6154	Other ossification of muscle, hand	Arthritis Joint Upper Limb	Nociceptive Pain
M61541	Other ossification of muscle, right hand	Arthritis Joint Upper Limb	Nociceptive Pain
M61542	Other ossification of muscle, left hand	Arthritis Joint Upper Limb	Nociceptive Pain
M61549	Other ossification of muscle, unspecified hand	Arthritis Joint Upper Limb	Nociceptive Pain

© 2024 Peterson MD et al. *JAMA Neurology.*

M6155	Other ossification of muscle, thigh	Arthritis Joint Lower Limb	Nociceptive Pain
M61551	Other ossification of muscle, right thigh	Arthritis Joint Lower Limb	Nociceptive Pain
M61552	Other ossification of muscle, left thigh	Arthritis Joint Lower Limb	Nociceptive Pain
M61559	Other ossification of muscle, unspecified thigh	Arthritis Joint Lower Limb	Nociceptive Pain
M6156	Other ossification of muscle, lower leg	Arthritis Joint Lower Limb	Nociceptive Pain
M61561	Other ossification of muscle, right lower leg	Arthritis Joint Lower Limb	Nociceptive Pain
M61562	Other ossification of muscle, left lower leg	Arthritis Joint Lower Limb	Nociceptive Pain
M61569	Other ossification of muscle, unspecified lower leg	Arthritis Joint Lower Limb	Nociceptive Pain
M6157	Other ossification of muscle, ankle and foot	Arthritis Joint Lower Limb	Nociceptive Pain
M61571	Other ossification of muscle, right ankle and foot	Arthritis Joint Lower Limb	Nociceptive Pain
M61572	Other ossification of muscle, left ankle and foot	Arthritis Joint Lower Limb	Nociceptive Pain
M61579	Other ossification of muscle, unspecified ankle and foot	Arthritis Joint Lower Limb	Nociceptive Pain
M6158	Other ossification of muscle, other site	Arthritis Joint Other	Nociceptive Pain
M6159	Other ossification of muscle, multiple sites	Arthritis Joint Other	Nociceptive Pain
M619	Calcification and ossification of muscle, unspecified	Arthritis Joint Other	Nociceptive Pain
M62	Other disorders of muscle	Arthritis Joint Other	Nociceptive Pain
M620	Separation of muscle (nontraumatic)	Arthritis Joint Other	Nociceptive Pain
M6200	Separation of muscle (nontraumatic), unspecified site	Arthritis Joint Other	Nociceptive Pain
M6201	Separation of muscle (nontraumatic), shoulder	Arthritis Joint Upper Limb	Nociceptive Pain
M62011	Separation of muscle (nontraumatic), right shoulder	Arthritis Joint Upper Limb	Nociceptive Pain
M62012	Separation of muscle (nontraumatic), left shoulder	Arthritis Joint Upper Limb	Nociceptive Pain
M62019	Separation of muscle (nontraumatic), unspecified shoulder	Arthritis Joint Upper Limb	Nociceptive Pain
M6202	Separation of muscle (nontraumatic), upper arm	Arthritis Joint Upper Limb	Nociceptive Pain
M62021	Separation of muscle (nontraumatic), right upper arm	Arthritis Joint Upper Limb	Nociceptive Pain
M62022	Separation of muscle (nontraumatic), left upper arm	Arthritis Joint Upper Limb	Nociceptive Pain
M62029	Separation of muscle (nontraumatic), unspecified upper arm	Arthritis Joint Upper Limb	Nociceptive Pain
M6203	Separation of muscle (nontraumatic), forearm	Arthritis Joint Upper Limb	Nociceptive Pain
M62031	Separation of muscle (nontraumatic), right forearm	Arthritis Joint Upper Limb	Nociceptive Pain
M62032	Separation of muscle (nontraumatic), left forearm	Arthritis Joint Upper Limb	Nociceptive Pain
M62039	Separation of muscle (nontraumatic), unspecified forearm	Arthritis Joint Upper Limb	Nociceptive Pain
M6204	Separation of muscle (nontraumatic), hand	Arthritis Joint Upper Limb	Nociceptive Pain
M62041	Separation of muscle (nontraumatic), right hand	Arthritis Joint Upper Limb	Nociceptive Pain
M62042	Separation of muscle (nontraumatic), left hand	Arthritis Joint Upper Limb	Nociceptive Pain
M62049	Separation of muscle (nontraumatic), unspecified hand	Arthritis Joint Upper Limb	Nociceptive Pain
M6205	Separation of muscle (nontraumatic), thigh	Arthritis Joint Lower Limb	Nociceptive Pain
M62051	Separation of muscle (nontraumatic), right thigh	Arthritis Joint Lower Limb	Nociceptive Pain
M62052	Separation of muscle (nontraumatic), left thigh	Arthritis Joint Lower Limb	Nociceptive Pain

© 2024 Peterson MD et al. *JAMA Neurology.*

M62059	Separation of muscle (nontraumatic), unspecified thigh	Arthritis Joint Lower Limb	Nociceptive Pain
M6206	Separation of muscle (nontraumatic), lower leg	Arthritis Joint Lower Limb	Nociceptive Pain
M62061	Separation of muscle (nontraumatic), right lower leg	Arthritis Joint Lower Limb	Nociceptive Pain
M62062	Separation of muscle (nontraumatic), left lower leg	Arthritis Joint Lower Limb	Nociceptive Pain
M62069	Separation of muscle (nontraumatic), unspecified lower leg	Arthritis Joint Lower Limb	Nociceptive Pain
M6207	Separation of muscle (nontraumatic), ankle and foot	Arthritis Joint Lower Limb	Nociceptive Pain
M62071	Separation of muscle (nontraumatic), right ankle and foot	Arthritis Joint Lower Limb	Nociceptive Pain
M62072	Separation of muscle (nontraumatic), left ankle and foot	Arthritis Joint Lower Limb	Nociceptive Pain
M62079	Separation of muscle (nontraumatic), unsp ankle and foot	Arthritis Joint Lower Limb	Nociceptive Pain
M6208	Separation of muscle (nontraumatic), other site	Arthritis Joint Other	Nociceptive Pain
M621	Other rupture of muscle (nontraumatic)	Arthritis Joint Other	Nociceptive Pain
M6210	Other rupture of muscle (nontraumatic), unspecified site	Arthritis Joint Other	Nociceptive Pain
M6211	Other rupture of muscle (nontraumatic), shoulder	Arthritis Joint Upper Limb	Nociceptive Pain
M62111	Other rupture of muscle (nontraumatic), right shoulder	Arthritis Joint Upper Limb	Nociceptive Pain
M62112	Other rupture of muscle (nontraumatic), left shoulder	Arthritis Joint Upper Limb	Nociceptive Pain
M62119	Other rupture of muscle (nontraumatic), unspecified shoulder	Arthritis Joint Upper Limb	Nociceptive Pain
M6212	Other rupture of muscle (nontraumatic), upper arm	Arthritis Joint Upper Limb	Nociceptive Pain
M62121	Other rupture of muscle (nontraumatic), right upper arm	Arthritis Joint Upper Limb	Nociceptive Pain
M62122	Other rupture of muscle (nontraumatic), left upper arm	Arthritis Joint Upper Limb	Nociceptive Pain
M62129	Other rupture of muscle (nontraumatic), unsp upper arm	Arthritis Joint Upper Limb	Nociceptive Pain
M6213	Other rupture of muscle (nontraumatic), forearm	Arthritis Joint Upper Limb	Nociceptive Pain
M62131	Other rupture of muscle (nontraumatic), right forearm	Arthritis Joint Upper Limb	Nociceptive Pain
M62132	Other rupture of muscle (nontraumatic), left forearm	Arthritis Joint Upper Limb	Nociceptive Pain
M62139	Other rupture of muscle (nontraumatic), unspecified forearm	Arthritis Joint Upper Limb	Nociceptive Pain
M6214	Other rupture of muscle (nontraumatic), hand	Arthritis Joint Upper Limb	Nociceptive Pain
M62141	Other rupture of muscle (nontraumatic), right hand	Arthritis Joint Upper Limb	Nociceptive Pain
M62142	Other rupture of muscle (nontraumatic), left hand	Arthritis Joint Upper Limb	Nociceptive Pain
M62149	Other rupture of muscle (nontraumatic), unspecified hand	Arthritis Joint Upper Limb	Nociceptive Pain
M6215	Other rupture of muscle (nontraumatic), thigh	Arthritis Joint Lower Limb	Nociceptive Pain
M62151	Other rupture of muscle (nontraumatic), right thigh	Arthritis Joint Lower Limb	Nociceptive Pain
M62152	Other rupture of muscle (nontraumatic), left thigh	Arthritis Joint Lower Limb	Nociceptive Pain
M62159	Other rupture of muscle (nontraumatic), unspecified thigh	Arthritis Joint Lower Limb	Nociceptive Pain
M6216	Other rupture of muscle (nontraumatic), lower leg	Arthritis Joint Lower Limb	Nociceptive Pain
M62161	Other rupture of muscle (nontraumatic), right lower leg	Arthritis Joint Lower Limb	Nociceptive Pain
M62162	Other rupture of muscle (nontraumatic), left lower leg	Arthritis Joint Lower Limb	Nociceptive Pain
M62169	Other rupture of muscle (nontraumatic), unsp lower leg	Arthritis Joint Lower Limb	Nociceptive Pain
M6217	Other rupture of muscle (nontraumatic), ankle and foot	Arthritis Joint Lower Limb	Nociceptive Pain

© 2024 Peterson MD et al. *JAMA Neurology.*

M62171	Other rupture of muscle (nontraumatic), right ankle and foot	Arthritis Joint Lower Limb	Nociceptive Pain
M62172	Other rupture of muscle (nontraumatic), left ankle and foot	Arthritis Joint Lower Limb	Nociceptive Pain
M62179	Other rupture of muscle (nontraumatic), unsp ankle and foot	Arthritis Joint Lower Limb	Nociceptive Pain
M6218	Other rupture of muscle (nontraumatic), other site	Arthritis Joint Other	Nociceptive Pain
M622	Nontraumatic ischemic infarction of muscle	Arthritis Joint Other	Nociceptive Pain
M6220	Nontraumatic ischemic infarction of muscle, unspecified site	Arthritis Joint Other	Nociceptive Pain
M6221	Nontraumatic ischemic infarction of muscle, shoulder	Arthritis Joint Upper Limb	Nociceptive Pain
M62211	Nontraumatic ischemic infarction of muscle, right shoulder	Arthritis Joint Upper Limb	Nociceptive Pain
M62212	Nontraumatic ischemic infarction of muscle, left shoulder	Arthritis Joint Upper Limb	Nociceptive Pain
M62219	Nontraumatic ischemic infarction of muscle, unsp shoulder	Arthritis Joint Upper Limb	Nociceptive Pain
M6222	Nontraumatic ischemic infarction of muscle, upper arm	Arthritis Joint Upper Limb	Nociceptive Pain
M62221	Nontraumatic ischemic infarction of muscle, right upper arm	Arthritis Joint Upper Limb	Nociceptive Pain
M62222	Nontraumatic ischemic infarction of muscle, left upper arm	Arthritis Joint Upper Limb	Nociceptive Pain
M62229	Nontraumatic ischemic infarction of muscle, unsp upper arm	Arthritis Joint Upper Limb	Nociceptive Pain
M6223	Nontraumatic ischemic infarction of muscle, forearm	Arthritis Joint Upper Limb	Nociceptive Pain
M62231	Nontraumatic ischemic infarction of muscle, right forearm	Arthritis Joint Upper Limb	Nociceptive Pain
M62232	Nontraumatic ischemic infarction of muscle, left forearm	Arthritis Joint Upper Limb	Nociceptive Pain
M62239	Nontraumatic ischemic infarction of muscle, unsp forearm	Arthritis Joint Upper Limb	Nociceptive Pain
M6224	Nontraumatic ischemic infarction of muscle, hand	Arthritis Joint Upper Limb	Nociceptive Pain
M62241	Nontraumatic ischemic infarction of muscle, right hand	Arthritis Joint Upper Limb	Nociceptive Pain
M62242	Nontraumatic ischemic infarction of muscle, left hand	Arthritis Joint Upper Limb	Nociceptive Pain
M62249	Nontraumatic ischemic infarction of muscle, unspecified hand	Arthritis Joint Upper Limb	Nociceptive Pain
M6225	Nontraumatic ischemic infarction of muscle, thigh	Arthritis Joint Lower Limb	Nociceptive Pain
M62251	Nontraumatic ischemic infarction of muscle, right thigh	Arthritis Joint Lower Limb	Nociceptive Pain
M62252	Nontraumatic ischemic infarction of muscle, left thigh	Arthritis Joint Lower Limb	Nociceptive Pain
M62259	Nontraumatic ischemic infarction of muscle, unsp thigh	Arthritis Joint Lower Limb	Nociceptive Pain
M6226	Nontraumatic ischemic infarction of muscle, lower leg	Arthritis Joint Lower Limb	Nociceptive Pain
M62261	Nontraumatic ischemic infarction of muscle, right lower leg	Arthritis Joint Lower Limb	Nociceptive Pain
M62262	Nontraumatic ischemic infarction of muscle, left lower leg	Arthritis Joint Lower Limb	Nociceptive Pain
M62269	Nontraumatic ischemic infarction of muscle, unsp lower leg	Arthritis Joint Lower Limb	Nociceptive Pain
M6227	Nontraumatic ischemic infarction of muscle, ankle and foot	Arthritis Joint Lower Limb	Nociceptive Pain
M62271	Nontraumatic ischemic infarction of muscle, right ank/ft	Arthritis Joint Lower Limb	Nociceptive Pain
M62272	Nontraumatic ischemic infarction of muscle, left ank/ft	Arthritis Joint Lower Limb	Nociceptive Pain
M62279	Nontraumatic ischemic infarction of muscle, unsp ank/ft	Arthritis Joint Lower Limb	Nociceptive Pain
M6228	Nontraumatic ischemic infarction of muscle, other site	Arthritis Joint Other	Nociceptive Pain
M623	Immobility syndrome (paraplegic)	Arthritis Joint Lower Limb	Nociceptive Pain
M624	Contracture of muscle	Arthritis Joint Other	Nociceptive Pain

© 2024 Peterson MD et al. *JAMA Neurology.*

M6240	Contracture of muscle, unspecified site	Arthritis Joint Other	Nociceptive Pain
M6241	Contracture of muscle, shoulder	Arthritis Joint Upper Limb	Nociceptive Pain
M62411	Contracture of muscle, right shoulder	Arthritis Joint Upper Limb	Nociceptive Pain
M62412	Contracture of muscle, left shoulder	Arthritis Joint Upper Limb	Nociceptive Pain
M62419	Contracture of muscle, unspecified shoulder	Arthritis Joint Upper Limb	Nociceptive Pain
M6242	Contracture of muscle, upper arm	Arthritis Joint Upper Limb	Nociceptive Pain
M62421	Contracture of muscle, right upper arm	Arthritis Joint Upper Limb	Nociceptive Pain
M62422	Contracture of muscle, left upper arm	Arthritis Joint Upper Limb	Nociceptive Pain
M62429	Contracture of muscle, unspecified upper arm	Arthritis Joint Upper Limb	Nociceptive Pain
M6243	Contracture of muscle, forearm	Arthritis Joint Upper Limb	Nociceptive Pain
M62431	Contracture of muscle, right forearm	Arthritis Joint Upper Limb	Nociceptive Pain
M62432	Contracture of muscle, left forearm	Arthritis Joint Upper Limb	Nociceptive Pain
M62439	Contracture of muscle, unspecified forearm	Arthritis Joint Upper Limb	Nociceptive Pain
M6244	Contracture of muscle, hand	Arthritis Joint Upper Limb	Nociceptive Pain
M62441	Contracture of muscle, right hand	Arthritis Joint Upper Limb	Nociceptive Pain
M62442	Contracture of muscle, left hand	Arthritis Joint Upper Limb	Nociceptive Pain
M62449	Contracture of muscle, unspecified hand	Arthritis Joint Upper Limb	Nociceptive Pain
M6245	Contracture of muscle, thigh	Arthritis Joint Lower Limb	Nociceptive Pain
M62451	Contracture of muscle, right thigh	Arthritis Joint Lower Limb	Nociceptive Pain
M62452	Contracture of muscle, left thigh	Arthritis Joint Lower Limb	Nociceptive Pain
M62459	Contracture of muscle, unspecified thigh	Arthritis Joint Lower Limb	Nociceptive Pain
M6246	Contracture of muscle, lower leg	Arthritis Joint Lower Limb	Nociceptive Pain
M62461	Contracture of muscle, right lower leg	Arthritis Joint Lower Limb	Nociceptive Pain
M62462	Contracture of muscle, left lower leg	Arthritis Joint Lower Limb	Nociceptive Pain
M62469	Contracture of muscle, unspecified lower leg	Arthritis Joint Lower Limb	Nociceptive Pain
M6247	Contracture of muscle, ankle and foot	Arthritis Joint Lower Limb	Nociceptive Pain
M62471	Contracture of muscle, right ankle and foot	Arthritis Joint Lower Limb	Nociceptive Pain
M62472	Contracture of muscle, left ankle and foot	Arthritis Joint Lower Limb	Nociceptive Pain
M62479	Contracture of muscle, unspecified ankle and foot	Arthritis Joint Lower Limb	Nociceptive Pain
M6248	Contracture of muscle, other site	Arthritis Joint Other	Nociceptive Pain
M6249	Contracture of muscle, multiple sites	Arthritis Joint Other	Nociceptive Pain
M625	Muscle wasting and atrophy, not elsewhere classified	Arthritis Joint Other	Nociceptive Pain
M6250	Muscle wasting and atrophy, NEC, unsp site	Arthritis Joint Other	Nociceptive Pain
M6251	Muscle wasting and atrophy, NEC, shoulder	Arthritis Joint Upper Limb	Nociceptive Pain
M62511	Muscle wasting and atrophy, NEC, right shoulder	Arthritis Joint Upper Limb	Nociceptive Pain
M62512	Muscle wasting and atrophy, NEC, left shoulder	Arthritis Joint Upper Limb	Nociceptive Pain
M62519	Muscle wasting and atrophy, NEC, unsp shoulder	Arthritis Joint Upper Limb	Nociceptive Pain

© 2024 Peterson MD et al. *JAMA Neurology.*

M6252	Muscle wasting and atrophy, NEC, upper arm	Arthritis Joint Upper Limb	Nociceptive Pain
M62521	Muscle wasting and atrophy, NEC, right upper arm	Arthritis Joint Upper Limb	Nociceptive Pain
M62522	Muscle wasting and atrophy, NEC, left upper arm	Arthritis Joint Upper Limb	Nociceptive Pain
M62529	Muscle wasting and atrophy, NEC, unsp upper arm	Arthritis Joint Upper Limb	Nociceptive Pain
M6253	Muscle wasting and atrophy, NEC, forearm	Arthritis Joint Upper Limb	Nociceptive Pain
M62531	Muscle wasting and atrophy, NEC, right forearm	Arthritis Joint Upper Limb	Nociceptive Pain
M62532	Muscle wasting and atrophy, NEC, left forearm	Arthritis Joint Upper Limb	Nociceptive Pain
M62539	Muscle wasting and atrophy, NEC, unsp forearm	Arthritis Joint Upper Limb	Nociceptive Pain
M6254	Muscle wasting and atrophy, not elsewhere classified, hand	Arthritis Joint Upper Limb	Nociceptive Pain
M62541	Muscle wasting and atrophy, NEC, right hand	Arthritis Joint Upper Limb	Nociceptive Pain
M62542	Muscle wasting and atrophy, NEC, left hand	Arthritis Joint Upper Limb	Nociceptive Pain
M62549	Muscle wasting and atrophy, NEC, unsp hand	Arthritis Joint Upper Limb	Nociceptive Pain
M6255	Muscle wasting and atrophy, not elsewhere classified, thigh	Arthritis Joint Lower Limb	Nociceptive Pain
M62551	Muscle wasting and atrophy, NEC, right thigh	Arthritis Joint Lower Limb	Nociceptive Pain
M62552	Muscle wasting and atrophy, NEC, left thigh	Arthritis Joint Lower Limb	Nociceptive Pain
M62559	Muscle wasting and atrophy, NEC, unsp thigh	Arthritis Joint Lower Limb	Nociceptive Pain
M6256	Muscle wasting and atrophy, NEC, lower leg	Arthritis Joint Lower Limb	Nociceptive Pain
M62561	Muscle wasting and atrophy, NEC, right lower leg	Arthritis Joint Lower Limb	Nociceptive Pain
M62562	Muscle wasting and atrophy, NEC, left lower leg	Arthritis Joint Lower Limb	Nociceptive Pain
M62569	Muscle wasting and atrophy, NEC, unsp lower leg	Arthritis Joint Lower Limb	Nociceptive Pain
M6257	Muscle wasting and atrophy, NEC, ankle and foot	Arthritis Joint Lower Limb	Nociceptive Pain
M62571	Muscle wasting and atrophy, NEC, right ankle and foot	Arthritis Joint Lower Limb	Nociceptive Pain
M62572	Muscle wasting and atrophy, NEC, left ankle and foot	Arthritis Joint Lower Limb	Nociceptive Pain
M62579	Muscle wasting and atrophy, NEC, unsp ankle and foot	Arthritis Joint Lower Limb	Nociceptive Pain
M6258	Muscle wasting and atrophy, NEC, oth site	Arthritis Joint Other	Nociceptive Pain
M6259	Muscle wasting and atrophy, NEC, multiple sites	Arthritis Joint Other	Nociceptive Pain
M625A	Muscle wasting and atrophy, not elsewhere classified, back	Arthritis Joint Spine and Hips	Nociceptive Pain
M625A0	Muscle wasting and atrophy, NEC, back, cervical	Arthritis Joint Spine and Hips	Nociceptive Pain
M625A1	Muscle wasting and atrophy, NEC, back, thoracic	Arthritis Joint Spine and Hips	Nociceptive Pain
M625A2	Muscle wasting and atrophy, NEC, back, lumbosacral	Arthritis Joint Spine and Hips	Nociceptive Pain
M625A9	Muscle wasting and atrophy, NEC, back, unspecified level	Arthritis Joint Spine and Hips	Nociceptive Pain
M628	Other specified disorders of muscle	Arthritis Joint Other	Nociceptive Pain
M6281	Muscle weakness (generalized)	Arthritis Joint Other	Nociceptive Pain
M6282	Rhabdomyolysis	Arthritis Joint Other	Nociceptive Pain
M6283	Muscle spasm	Arthritis Joint Other	Nociceptive Pain
M62830	Muscle spasm of back	Arthritis Joint Spine and Hips	Nociceptive Pain
M62831	Muscle spasm of calf	Arthritis Joint Other	Nociceptive Pain

© 2024 Peterson MD et al. *JAMA Neurology.*

M62838	Other muscle spasm	Arthritis Joint Other	Nociceptive Pain
M6284	Sarcopenia	Arthritis Joint Other	Nociceptive Pain
M6289	Other specified disorders of muscle	Arthritis Joint Other	Nociceptive Pain
M629	Disorder of muscle, unspecified	Arthritis Joint Other	Nociceptive Pain
M65	Synovitis and tenosynovitis	Arthritis Joint Other	Nociceptive Pain
M650	Abscess of tendon sheath	Arthritis Joint Other	Nociceptive Pain
M6500	Abscess of tendon sheath, unspecified site	Arthritis Joint Other	Nociceptive Pain
M6501	Abscess of tendon sheath, shoulder	Arthritis Joint Upper Limb	Nociceptive Pain
M65011	Abscess of tendon sheath, right shoulder	Arthritis Joint Upper Limb	Nociceptive Pain
M65012	Abscess of tendon sheath, left shoulder	Arthritis Joint Upper Limb	Nociceptive Pain
M65019	Abscess of tendon sheath, unspecified shoulder	Arthritis Joint Upper Limb	Nociceptive Pain
M6502	Abscess of tendon sheath, upper arm	Arthritis Joint Upper Limb	Nociceptive Pain
M65021	Abscess of tendon sheath, right upper arm	Arthritis Joint Upper Limb	Nociceptive Pain
M65022	Abscess of tendon sheath, left upper arm	Arthritis Joint Upper Limb	Nociceptive Pain
M65029	Abscess of tendon sheath, unspecified upper arm	Arthritis Joint Upper Limb	Nociceptive Pain
M6503	Abscess of tendon sheath, forearm	Arthritis Joint Upper Limb	Nociceptive Pain
M65031	Abscess of tendon sheath, right forearm	Arthritis Joint Upper Limb	Nociceptive Pain
M65032	Abscess of tendon sheath, left forearm	Arthritis Joint Upper Limb	Nociceptive Pain
M65039	Abscess of tendon sheath, unspecified forearm	Arthritis Joint Upper Limb	Nociceptive Pain
M6504	Abscess of tendon sheath, hand	Arthritis Joint Upper Limb	Nociceptive Pain
M65041	Abscess of tendon sheath, right hand	Arthritis Joint Upper Limb	Nociceptive Pain
M65042	Abscess of tendon sheath, left hand	Arthritis Joint Upper Limb	Nociceptive Pain
M65049	Abscess of tendon sheath, unspecified hand	Arthritis Joint Upper Limb	Nociceptive Pain
M6505	Abscess of tendon sheath, thigh	Arthritis Joint Lower Limb	Nociceptive Pain
M65051	Abscess of tendon sheath, right thigh	Arthritis Joint Lower Limb	Nociceptive Pain
M65052	Abscess of tendon sheath, left thigh	Arthritis Joint Lower Limb	Nociceptive Pain
M65059	Abscess of tendon sheath, unspecified thigh	Arthritis Joint Lower Limb	Nociceptive Pain
M6506	Abscess of tendon sheath, lower leg	Arthritis Joint Lower Limb	Nociceptive Pain
M65061	Abscess of tendon sheath, right lower leg	Arthritis Joint Lower Limb	Nociceptive Pain
M65062	Abscess of tendon sheath, left lower leg	Arthritis Joint Lower Limb	Nociceptive Pain
M65069	Abscess of tendon sheath, unspecified lower leg	Arthritis Joint Lower Limb	Nociceptive Pain
M6507	Abscess of tendon sheath, ankle and foot	Arthritis Joint Lower Limb	Nociceptive Pain
M65071	Abscess of tendon sheath, right ankle and foot	Arthritis Joint Lower Limb	Nociceptive Pain
M65072	Abscess of tendon sheath, left ankle and foot	Arthritis Joint Lower Limb	Nociceptive Pain
M65079	Abscess of tendon sheath, unspecified ankle and foot	Arthritis Joint Lower Limb	Nociceptive Pain
M6508	Abscess of tendon sheath, other site	Arthritis Joint Other	Nociceptive Pain
M651	Other infective (teno)synovitis	Arthritis Joint Other	Nociceptive Pain

© 2024 Peterson MD et al. *JAMA Neurology.*

M6510	Other infective (teno)synovitis, unspecified site	Arthritis Joint Other	Nociceptive Pain
M6511	Other infective (teno)synovitis, shoulder	Arthritis Joint Upper Limb	Nociceptive Pain
M65111	Other infective (teno)synovitis, right shoulder	Arthritis Joint Upper Limb	Nociceptive Pain
M65112	Other infective (teno)synovitis, left shoulder	Arthritis Joint Upper Limb	Nociceptive Pain
M65119	Other infective (teno)synovitis, unspecified shoulder	Arthritis Joint Upper Limb	Nociceptive Pain
M6512	Other infective (teno)synovitis, elbow	Arthritis Joint Upper Limb	Nociceptive Pain
M65121	Other infective (teno)synovitis, right elbow	Arthritis Joint Upper Limb	Nociceptive Pain
M65122	Other infective (teno)synovitis, left elbow	Arthritis Joint Upper Limb	Nociceptive Pain
M65129	Other infective (teno)synovitis, unspecified elbow	Arthritis Joint Upper Limb	Nociceptive Pain
M6513	Other infective (teno)synovitis, wrist	Arthritis Joint Upper Limb	Nociceptive Pain
M65131	Other infective (teno)synovitis, right wrist	Arthritis Joint Upper Limb	Nociceptive Pain
M65132	Other infective (teno)synovitis, left wrist	Arthritis Joint Upper Limb	Nociceptive Pain
M65139	Other infective (teno)synovitis, unspecified wrist	Arthritis Joint Upper Limb	Nociceptive Pain
M6514	Other infective (teno)synovitis, hand	Arthritis Joint Upper Limb	Nociceptive Pain
M65141	Other infective (teno)synovitis, right hand	Arthritis Joint Upper Limb	Nociceptive Pain
M65142	Other infective (teno)synovitis, left hand	Arthritis Joint Upper Limb	Nociceptive Pain
M65149	Other infective (teno)synovitis, unspecified hand	Arthritis Joint Upper Limb	Nociceptive Pain
M6515	Other infective (teno)synovitis, hip	Arthritis Joint Spine and Hips	Nociceptive Pain
M65151	Other infective (teno)synovitis, right hip	Arthritis Joint Spine and Hips	Nociceptive Pain
M65152	Other infective (teno)synovitis, left hip	Arthritis Joint Spine and Hips	Nociceptive Pain
M65159	Other infective (teno)synovitis, unspecified hip	Arthritis Joint Spine and Hips	Nociceptive Pain
M6516	Other infective (teno)synovitis, knee	Arthritis Joint Lower Limb	Nociceptive Pain
M65161	Other infective (teno)synovitis, right knee	Arthritis Joint Lower Limb	Nociceptive Pain
M65162	Other infective (teno)synovitis, left knee	Arthritis Joint Lower Limb	Nociceptive Pain
M65169	Other infective (teno)synovitis, unspecified knee	Arthritis Joint Lower Limb	Nociceptive Pain
M6517	Other infective (teno)synovitis, ankle and foot	Arthritis Joint Lower Limb	Nociceptive Pain
M65171	Other infective (teno)synovitis, right ankle and foot	Arthritis Joint Lower Limb	Nociceptive Pain
M65172	Other infective (teno)synovitis, left ankle and foot	Arthritis Joint Lower Limb	Nociceptive Pain
M65179	Other infective (teno)synovitis, unspecified ankle and foot	Arthritis Joint Lower Limb	Nociceptive Pain
M6518	Other infective (teno)synovitis, other site	Arthritis Joint Other	Nociceptive Pain
M6519	Other infective (teno)synovitis, multiple sites	Arthritis Joint Other	Nociceptive Pain
M652	Calcific tendinitis	Arthritis Joint Other	Nociceptive Pain
M6520	Calcific tendinitis, unspecified site	Arthritis Joint Other	Nociceptive Pain
M6522	Calcific tendinitis, upper arm	Arthritis Joint Upper Limb	Nociceptive Pain
M65221	Calcific tendinitis, right upper arm	Arthritis Joint Upper Limb	Nociceptive Pain
M65222	Calcific tendinitis, left upper arm	Arthritis Joint Upper Limb	Nociceptive Pain
M65229	Calcific tendinitis, unspecified upper arm	Arthritis Joint Upper Limb	Nociceptive Pain

© 2024 Peterson MD et al. *JAMA Neurology.*

M6523	Calcific tendinitis, forearm	Arthritis Joint Upper Limb	Nociceptive Pain
M65231	Calcific tendinitis, right forearm	Arthritis Joint Upper Limb	Nociceptive Pain
M65232	Calcific tendinitis, left forearm	Arthritis Joint Upper Limb	Nociceptive Pain
M65239	Calcific tendinitis, unspecified forearm	Arthritis Joint Upper Limb	Nociceptive Pain
M6524	Calcific tendinitis, hand	Arthritis Joint Upper Limb	Nociceptive Pain
M65241	Calcific tendinitis, right hand	Arthritis Joint Upper Limb	Nociceptive Pain
M65242	Calcific tendinitis, left hand	Arthritis Joint Upper Limb	Nociceptive Pain
M65249	Calcific tendinitis, unspecified hand	Arthritis Joint Upper Limb	Nociceptive Pain
M6525	Calcific tendinitis, thigh	Arthritis Joint Lower Limb	Nociceptive Pain
M65251	Calcific tendinitis, right thigh	Arthritis Joint Lower Limb	Nociceptive Pain
M65252	Calcific tendinitis, left thigh	Arthritis Joint Lower Limb	Nociceptive Pain
M65259	Calcific tendinitis, unspecified thigh	Arthritis Joint Lower Limb	Nociceptive Pain
M6526	Calcific tendinitis, lower leg	Arthritis Joint Lower Limb	Nociceptive Pain
M65261	Calcific tendinitis, right lower leg	Arthritis Joint Lower Limb	Nociceptive Pain
M65262	Calcific tendinitis, left lower leg	Arthritis Joint Lower Limb	Nociceptive Pain
M65269	Calcific tendinitis, unspecified lower leg	Arthritis Joint Lower Limb	Nociceptive Pain
M6527	Calcific tendinitis, ankle and foot	Arthritis Joint Lower Limb	Nociceptive Pain
M65271	Calcific tendinitis, right ankle and foot	Arthritis Joint Lower Limb	Nociceptive Pain
M65272	Calcific tendinitis, left ankle and foot	Arthritis Joint Lower Limb	Nociceptive Pain
M65279	Calcific tendinitis, unspecified ankle and foot	Arthritis Joint Lower Limb	Nociceptive Pain
M6528	Calcific tendinitis, other site	Arthritis Joint Other	Nociceptive Pain
M6529	Calcific tendinitis, multiple sites	Arthritis Joint Other	Nociceptive Pain
M653	Trigger finger	Arthritis Joint Upper Limb	Nociceptive Pain
M6530	Trigger finger, unspecified finger	Arthritis Joint Upper Limb	Nociceptive Pain
M6531	Trigger thumb	Arthritis Joint Upper Limb	Nociceptive Pain
M65311	Trigger thumb, right thumb	Arthritis Joint Upper Limb	Nociceptive Pain
M65312	Trigger thumb, left thumb	Arthritis Joint Upper Limb	Nociceptive Pain
M65319	Trigger thumb, unspecified thumb	Arthritis Joint Upper Limb	Nociceptive Pain
M6532	Trigger finger, index finger	Arthritis Joint Upper Limb	Nociceptive Pain
M65321	Trigger finger, right index finger	Arthritis Joint Upper Limb	Nociceptive Pain
M65322	Trigger finger, left index finger	Arthritis Joint Upper Limb	Nociceptive Pain
M65329	Trigger finger, unspecified index finger	Arthritis Joint Upper Limb	Nociceptive Pain
M6533	Trigger finger, middle finger	Arthritis Joint Upper Limb	Nociceptive Pain
M65331	Trigger finger, right middle finger	Arthritis Joint Upper Limb	Nociceptive Pain
M65332	Trigger finger, left middle finger	Arthritis Joint Upper Limb	Nociceptive Pain
M65339	Trigger finger, unspecified middle finger	Arthritis Joint Upper Limb	Nociceptive Pain
M6534	Trigger finger, ring finger	Arthritis Joint Upper Limb	Nociceptive Pain

© 2024 Peterson MD et al. *JAMA Neurology.*

M65341	Trigger finger, right ring finger	Arthritis Joint Upper Limb	Nociceptive Pain
M65342	Trigger finger, left ring finger	Arthritis Joint Upper Limb	Nociceptive Pain
M65349	Trigger finger, unspecified ring finger	Arthritis Joint Upper Limb	Nociceptive Pain
M6535	Trigger finger, little finger	Arthritis Joint Upper Limb	Nociceptive Pain
M65351	Trigger finger, right little finger	Arthritis Joint Upper Limb	Nociceptive Pain
M65352	Trigger finger, left little finger	Arthritis Joint Upper Limb	Nociceptive Pain
M65359	Trigger finger, unspecified little finger	Arthritis Joint Upper Limb	Nociceptive Pain
M654	Radial styloid tenosynovitis [de Quervain]	Arthritis Joint Upper Limb	Nociceptive Pain
M658	Other synovitis and tenosynovitis	Arthritis Joint Other	Nociceptive Pain
M6580	Other synovitis and tenosynovitis, unspecified site	Arthritis Joint Other	Nociceptive Pain
M6581	Other synovitis and tenosynovitis, shoulder	Arthritis Joint Upper Limb	Nociceptive Pain
M65811	Other synovitis and tenosynovitis, right shoulder	Arthritis Joint Upper Limb	Nociceptive Pain
M65812	Other synovitis and tenosynovitis, left shoulder	Arthritis Joint Upper Limb	Nociceptive Pain
M65819	Other synovitis and tenosynovitis, unspecified shoulder	Arthritis Joint Upper Limb	Nociceptive Pain
M6582	Other synovitis and tenosynovitis, upper arm	Arthritis Joint Upper Limb	Nociceptive Pain
M65821	Other synovitis and tenosynovitis, right upper arm	Arthritis Joint Upper Limb	Nociceptive Pain
M65822	Other synovitis and tenosynovitis, left upper arm	Arthritis Joint Upper Limb	Nociceptive Pain
M65829	Other synovitis and tenosynovitis, unspecified upper arm	Arthritis Joint Upper Limb	Nociceptive Pain
M6583	Other synovitis and tenosynovitis, forearm	Arthritis Joint Upper Limb	Nociceptive Pain
M65831	Other synovitis and tenosynovitis, right forearm	Arthritis Joint Upper Limb	Nociceptive Pain
M65832	Other synovitis and tenosynovitis, left forearm	Arthritis Joint Upper Limb	Nociceptive Pain
M65839	Other synovitis and tenosynovitis, unspecified forearm	Arthritis Joint Upper Limb	Nociceptive Pain
M6584	Other synovitis and tenosynovitis, hand	Arthritis Joint Upper Limb	Nociceptive Pain
M65841	Other synovitis and tenosynovitis, right hand	Arthritis Joint Upper Limb	Nociceptive Pain
M65842	Other synovitis and tenosynovitis, left hand	Arthritis Joint Upper Limb	Nociceptive Pain
M65849	Other synovitis and tenosynovitis, unspecified hand	Arthritis Joint Upper Limb	Nociceptive Pain
M6585	Other synovitis and tenosynovitis, thigh	Arthritis Joint Lower Limb	Nociceptive Pain
M65851	Other synovitis and tenosynovitis, right thigh	Arthritis Joint Lower Limb	Nociceptive Pain
M65852	Other synovitis and tenosynovitis, left thigh	Arthritis Joint Lower Limb	Nociceptive Pain
M65859	Other synovitis and tenosynovitis, unspecified thigh	Arthritis Joint Lower Limb	Nociceptive Pain
M6586	Other synovitis and tenosynovitis, lower leg	Arthritis Joint Lower Limb	Nociceptive Pain
M65861	Other synovitis and tenosynovitis, right lower leg	Arthritis Joint Lower Limb	Nociceptive Pain
M65862	Other synovitis and tenosynovitis, left lower leg	Arthritis Joint Lower Limb	Nociceptive Pain
M65869	Other synovitis and tenosynovitis, unspecified lower leg	Arthritis Joint Lower Limb	Nociceptive Pain
M6587	Other synovitis and tenosynovitis, ankle and foot	Arthritis Joint Lower Limb	Nociceptive Pain
M65871	Other synovitis and tenosynovitis, right ankle and foot	Arthritis Joint Lower Limb	Nociceptive Pain
M65872	Other synovitis and tenosynovitis, left ankle and foot	Arthritis Joint Lower Limb	Nociceptive Pain

© 2024 Peterson MD et al. *JAMA Neurology.*

M65879	Other synovitis and tenosynovitis, unsp ankle and foot	Arthritis Joint Lower Limb	Nociceptive Pain
M6588	Other synovitis and tenosynovitis, other site	Arthritis Joint Other	Nociceptive Pain
M6589	Other synovitis and tenosynovitis, multiple sites	Arthritis Joint Other	Nociceptive Pain
M659	Synovitis and tenosynovitis, unspecified	Arthritis Joint Other	Nociceptive Pain
M66	Spontaneous rupture of synovium and tendon	Arthritis Joint Other	Nociceptive Pain
M660	Rupture of popliteal cyst	Arthritis Joint Other	Nociceptive Pain
M661	Rupture of synovium	Arthritis Joint Other	Nociceptive Pain
M6610	Rupture of synovium, unspecified joint	Arthritis Joint Other	Nociceptive Pain
M6611	Rupture of synovium, shoulder	Arthritis Joint Upper Limb	Nociceptive Pain
M66111	Rupture of synovium, right shoulder	Arthritis Joint Upper Limb	Nociceptive Pain
M66112	Rupture of synovium, left shoulder	Arthritis Joint Upper Limb	Nociceptive Pain
M66119	Rupture of synovium, unspecified shoulder	Arthritis Joint Upper Limb	Nociceptive Pain
M6612	Rupture of synovium, elbow	Arthritis Joint Upper Limb	Nociceptive Pain
M66121	Rupture of synovium, right elbow	Arthritis Joint Upper Limb	Nociceptive Pain
M66122	Rupture of synovium, left elbow	Arthritis Joint Upper Limb	Nociceptive Pain
M66129	Rupture of synovium, unspecified elbow	Arthritis Joint Upper Limb	Nociceptive Pain
M6613	Rupture of synovium, wrist	Arthritis Joint Upper Limb	Nociceptive Pain
M66131	Rupture of synovium, right wrist	Arthritis Joint Upper Limb	Nociceptive Pain
M66132	Rupture of synovium, left wrist	Arthritis Joint Upper Limb	Nociceptive Pain
M66139	Rupture of synovium, unspecified wrist	Arthritis Joint Upper Limb	Nociceptive Pain
M6614	Rupture of synovium, hand and fingers	Arthritis Joint Upper Limb	Nociceptive Pain
M66141	Rupture of synovium, right hand	Arthritis Joint Upper Limb	Nociceptive Pain
M66142	Rupture of synovium, left hand	Arthritis Joint Upper Limb	Nociceptive Pain
M66143	Rupture of synovium, unspecified hand	Arthritis Joint Upper Limb	Nociceptive Pain
M66144	Rupture of synovium, right finger(s)	Arthritis Joint Upper Limb	Nociceptive Pain
M66145	Rupture of synovium, left finger(s)	Arthritis Joint Upper Limb	Nociceptive Pain
M66146	Rupture of synovium, unspecified finger(s)	Arthritis Joint Upper Limb	Nociceptive Pain
M6615	Rupture of synovium, hip	Arthritis Joint Spine and Hips	Nociceptive Pain
M66151	Rupture of synovium, right hip	Arthritis Joint Spine and Hips	Nociceptive Pain
M66152	Rupture of synovium, left hip	Arthritis Joint Spine and Hips	Nociceptive Pain
M66159	Rupture of synovium, unspecified hip	Arthritis Joint Spine and Hips	Nociceptive Pain
M6617	Rupture of synovium, ankle, foot and toes	Arthritis Joint Lower Limb	Nociceptive Pain
M66171	Rupture of synovium, right ankle	Arthritis Joint Lower Limb	Nociceptive Pain
M66172	Rupture of synovium, left ankle	Arthritis Joint Lower Limb	Nociceptive Pain
M66173	Rupture of synovium, unspecified ankle	Arthritis Joint Lower Limb	Nociceptive Pain
M66174	Rupture of synovium, right foot	Arthritis Joint Lower Limb	Nociceptive Pain
M66175	Rupture of synovium, left foot	Arthritis Joint Lower Limb	Nociceptive Pain

© 2024 Peterson MD et al. *JAMA Neurology.*

M66176	Rupture of synovium, unspecified foot	Arthritis Joint Lower Limb	Nociceptive Pain
M66177	Rupture of synovium, right toe(s)	Arthritis Joint Lower Limb	Nociceptive Pain
M66178	Rupture of synovium, left toe(s)	Arthritis Joint Lower Limb	Nociceptive Pain
M66179	Rupture of synovium, unspecified toe(s)	Arthritis Joint Lower Limb	Nociceptive Pain
M6618	Rupture of synovium, other site	Arthritis Joint Other	Nociceptive Pain
M662	Spontaneous rupture of extensor tendons	Arthritis Joint Upper Limb	Nociceptive Pain
M6620	Spontaneous rupture of extensor tendons, unspecified site	Arthritis Joint Upper Limb	Nociceptive Pain
M6621	Spontaneous rupture of extensor tendons, shoulder	Arthritis Joint Upper Limb	Nociceptive Pain
M66211	Spontaneous rupture of extensor tendons, right shoulder	Arthritis Joint Upper Limb	Nociceptive Pain
M66212	Spontaneous rupture of extensor tendons, left shoulder	Arthritis Joint Upper Limb	Nociceptive Pain
M66219	Spontaneous rupture of extensor tendons, unsp shoulder	Arthritis Joint Upper Limb	Nociceptive Pain
M6622	Spontaneous rupture of extensor tendons, upper arm	Arthritis Joint Upper Limb	Nociceptive Pain
M66221	Spontaneous rupture of extensor tendons, right upper arm	Arthritis Joint Upper Limb	Nociceptive Pain
M66222	Spontaneous rupture of extensor tendons, left upper arm	Arthritis Joint Upper Limb	Nociceptive Pain
M66229	Spontaneous rupture of extensor tendons, unsp upper arm	Arthritis Joint Upper Limb	Nociceptive Pain
M6623	Spontaneous rupture of extensor tendons, forearm	Arthritis Joint Upper Limb	Nociceptive Pain
M66231	Spontaneous rupture of extensor tendons, right forearm	Arthritis Joint Upper Limb	Nociceptive Pain
M66232	Spontaneous rupture of extensor tendons, left forearm	Arthritis Joint Upper Limb	Nociceptive Pain
M66239	Spontaneous rupture of extensor tendons, unspecified forearm	Arthritis Joint Upper Limb	Nociceptive Pain
M6624	Spontaneous rupture of extensor tendons, hand	Arthritis Joint Upper Limb	Nociceptive Pain
M66241	Spontaneous rupture of extensor tendons, right hand	Arthritis Joint Upper Limb	Nociceptive Pain
M66242	Spontaneous rupture of extensor tendons, left hand	Arthritis Joint Upper Limb	Nociceptive Pain
M66249	Spontaneous rupture of extensor tendons, unspecified hand	Arthritis Joint Upper Limb	Nociceptive Pain
M6625	Spontaneous rupture of extensor tendons, thigh	Arthritis Joint Lower Limb	Nociceptive Pain
M66251	Spontaneous rupture of extensor tendons, right thigh	Arthritis Joint Lower Limb	Nociceptive Pain
M66252	Spontaneous rupture of extensor tendons, left thigh	Arthritis Joint Lower Limb	Nociceptive Pain
M66259	Spontaneous rupture of extensor tendons, unspecified thigh	Arthritis Joint Lower Limb	Nociceptive Pain
M6626	Spontaneous rupture of extensor tendons, lower leg	Arthritis Joint Lower Limb	Nociceptive Pain
M66261	Spontaneous rupture of extensor tendons, right lower leg	Arthritis Joint Lower Limb	Nociceptive Pain
M66262	Spontaneous rupture of extensor tendons, left lower leg	Arthritis Joint Lower Limb	Nociceptive Pain
M66269	Spontaneous rupture of extensor tendons, unsp lower leg	Arthritis Joint Lower Limb	Nociceptive Pain
M6627	Spontaneous rupture of extensor tendons, ankle and foot	Arthritis Joint Lower Limb	Nociceptive Pain
M66271	Spontaneous rupture of extensor tendons, right ank/ft	Arthritis Joint Lower Limb	Nociceptive Pain
M66272	Spontaneous rupture of extensor tendons, left ankle and foot	Arthritis Joint Lower Limb	Nociceptive Pain
M66279	Spontaneous rupture of extensor tendons, unsp ankle and foot	Arthritis Joint Lower Limb	Nociceptive Pain
M6628	Spontaneous rupture of extensor tendons, other site	Arthritis Joint Upper Limb	Nociceptive Pain
M6629	Spontaneous rupture of extensor tendons, multiple sites	Arthritis Joint Other	Nociceptive Pain

© 2024 Peterson MD et al. *JAMA Neurology.*

M663	Spontaneous rupture of flexor tendons	Arthritis Joint Upper Limb	Nociceptive Pain
M6630	Spontaneous rupture of flexor tendons, unspecified site	Arthritis Joint Upper Limb	Nociceptive Pain
M6631	Spontaneous rupture of flexor tendons, shoulder	Arthritis Joint Upper Limb	Nociceptive Pain
M66311	Spontaneous rupture of flexor tendons, right shoulder	Arthritis Joint Upper Limb	Nociceptive Pain
M66312	Spontaneous rupture of flexor tendons, left shoulder	Arthritis Joint Upper Limb	Nociceptive Pain
M66319	Spontaneous rupture of flexor tendons, unspecified shoulder	Arthritis Joint Upper Limb	Nociceptive Pain
M6632	Spontaneous rupture of flexor tendons, upper arm	Arthritis Joint Upper Limb	Nociceptive Pain
M66321	Spontaneous rupture of flexor tendons, right upper arm	Arthritis Joint Upper Limb	Nociceptive Pain
M66322	Spontaneous rupture of flexor tendons, left upper arm	Arthritis Joint Upper Limb	Nociceptive Pain
M66329	Spontaneous rupture of flexor tendons, unspecified upper arm	Arthritis Joint Upper Limb	Nociceptive Pain
M6633	Spontaneous rupture of flexor tendons, forearm	Arthritis Joint Upper Limb	Nociceptive Pain
M66331	Spontaneous rupture of flexor tendons, right forearm	Arthritis Joint Upper Limb	Nociceptive Pain
M66332	Spontaneous rupture of flexor tendons, left forearm	Arthritis Joint Upper Limb	Nociceptive Pain
M66339	Spontaneous rupture of flexor tendons, unspecified forearm	Arthritis Joint Upper Limb	Nociceptive Pain
M6634	Spontaneous rupture of flexor tendons, hand	Arthritis Joint Upper Limb	Nociceptive Pain
M66341	Spontaneous rupture of flexor tendons, right hand	Arthritis Joint Upper Limb	Nociceptive Pain
M66342	Spontaneous rupture of flexor tendons, left hand	Arthritis Joint Upper Limb	Nociceptive Pain
M66349	Spontaneous rupture of flexor tendons, unspecified hand	Arthritis Joint Upper Limb	Nociceptive Pain
M6635	Spontaneous rupture of flexor tendons, thigh	Arthritis Joint Lower Limb	Nociceptive Pain
M66351	Spontaneous rupture of flexor tendons, right thigh	Arthritis Joint Lower Limb	Nociceptive Pain
M66352	Spontaneous rupture of flexor tendons, left thigh	Arthritis Joint Lower Limb	Nociceptive Pain
M66359	Spontaneous rupture of flexor tendons, unspecified thigh	Arthritis Joint Lower Limb	Nociceptive Pain
M6636	Spontaneous rupture of flexor tendons, lower leg	Arthritis Joint Lower Limb	Nociceptive Pain
M66361	Spontaneous rupture of flexor tendons, right lower leg	Arthritis Joint Lower Limb	Nociceptive Pain
M66362	Spontaneous rupture of flexor tendons, left lower leg	Arthritis Joint Lower Limb	Nociceptive Pain
M66369	Spontaneous rupture of flexor tendons, unspecified lower leg	Arthritis Joint Lower Limb	Nociceptive Pain
M6637	Spontaneous rupture of flexor tendons, ankle and foot	Arthritis Joint Lower Limb	Nociceptive Pain
M66371	Spontaneous rupture of flexor tendons, right ankle and foot	Arthritis Joint Lower Limb	Nociceptive Pain
M66372	Spontaneous rupture of flexor tendons, left ankle and foot	Arthritis Joint Lower Limb	Nociceptive Pain
M66379	Spontaneous rupture of flexor tendons, unsp ankle and foot	Arthritis Joint Lower Limb	Nociceptive Pain
M6638	Spontaneous rupture of flexor tendons, other site	Arthritis Joint Upper Limb	Nociceptive Pain
M6639	Spontaneous rupture of flexor tendons, multiple sites	Arthritis Joint Other	Nociceptive Pain
M668	Spontaneous rupture of other tendons	Arthritis Joint Other	Nociceptive Pain
M6680	Spontaneous rupture of other tendons, unspecified site	Arthritis Joint Other	Nociceptive Pain
M6681	Spontaneous rupture of other tendons, shoulder	Arthritis Joint Upper Limb	Nociceptive Pain
M66811	Spontaneous rupture of other tendons, right shoulder	Arthritis Joint Upper Limb	Nociceptive Pain
M66812	Spontaneous rupture of other tendons, left shoulder	Arthritis Joint Upper Limb	Nociceptive Pain

© 2024 Peterson MD et al. *JAMA Neurology.*

M66819	Spontaneous rupture of other tendons, unspecified shoulder	Arthritis Joint Upper Limb	Nociceptive Pain
M6682	Spontaneous rupture of other tendons, upper arm	Arthritis Joint Upper Limb	Nociceptive Pain
M66821	Spontaneous rupture of other tendons, right upper arm	Arthritis Joint Upper Limb	Nociceptive Pain
M66822	Spontaneous rupture of other tendons, left upper arm	Arthritis Joint Upper Limb	Nociceptive Pain
M66829	Spontaneous rupture of other tendons, unspecified upper arm	Arthritis Joint Upper Limb	Nociceptive Pain
M6683	Spontaneous rupture of other tendons, forearm	Arthritis Joint Upper Limb	Nociceptive Pain
M66831	Spontaneous rupture of other tendons, right forearm	Arthritis Joint Upper Limb	Nociceptive Pain
M66832	Spontaneous rupture of other tendons, left forearm	Arthritis Joint Upper Limb	Nociceptive Pain
M66839	Spontaneous rupture of other tendons, unspecified forearm	Arthritis Joint Upper Limb	Nociceptive Pain
M6684	Spontaneous rupture of other tendons, hand	Arthritis Joint Upper Limb	Nociceptive Pain
M66841	Spontaneous rupture of other tendons, right hand	Arthritis Joint Upper Limb	Nociceptive Pain
M66842	Spontaneous rupture of other tendons, left hand	Arthritis Joint Upper Limb	Nociceptive Pain
M66849	Spontaneous rupture of other tendons, unspecified hand	Arthritis Joint Upper Limb	Nociceptive Pain
M6685	Spontaneous rupture of other tendons, thigh	Arthritis Joint Lower Limb	Nociceptive Pain
M66851	Spontaneous rupture of other tendons, right thigh	Arthritis Joint Lower Limb	Nociceptive Pain
M66852	Spontaneous rupture of other tendons, left thigh	Arthritis Joint Lower Limb	Nociceptive Pain
M66859	Spontaneous rupture of other tendons, unspecified thigh	Arthritis Joint Lower Limb	Nociceptive Pain
M6686	Spontaneous rupture of other tendons, lower leg	Arthritis Joint Lower Limb	Nociceptive Pain
M66861	Spontaneous rupture of other tendons, right lower leg	Arthritis Joint Lower Limb	Nociceptive Pain
M66862	Spontaneous rupture of other tendons, left lower leg	Arthritis Joint Lower Limb	Nociceptive Pain
M66869	Spontaneous rupture of other tendons, unspecified lower leg	Arthritis Joint Lower Limb	Nociceptive Pain
M6687	Spontaneous rupture of other tendons, ankle and foot	Arthritis Joint Lower Limb	Nociceptive Pain
M66871	Spontaneous rupture of other tendons, right ankle and foot	Arthritis Joint Lower Limb	Nociceptive Pain
M66872	Spontaneous rupture of other tendons, left ankle and foot	Arthritis Joint Lower Limb	Nociceptive Pain
M66879	Spontaneous rupture of other tendons, unsp ankle and foot	Arthritis Joint Lower Limb	Nociceptive Pain
M6688	Spontaneous rupture of other tendons, other sites	Arthritis Joint Other	Nociceptive Pain
M6689	Spontaneous rupture of other tendons, multiple sites	Arthritis Joint Other	Nociceptive Pain
M669	Spontaneous rupture of unspecified tendon	Arthritis Joint Other	Nociceptive Pain
M67	Other disorders of synovium and tendon	Arthritis Joint Other	Nociceptive Pain
M670	Short Achilles tendon (acquired)	Arthritis Joint Lower Limb	Nociceptive Pain
M6700	Short Achilles tendon (acquired), unspecified ankle	Arthritis Joint Lower Limb	Nociceptive Pain
M6701	Short Achilles tendon (acquired), right ankle	Arthritis Joint Lower Limb	Nociceptive Pain
M6702	Short Achilles tendon (acquired), left ankle	Arthritis Joint Lower Limb	Nociceptive Pain
M672	Synovial hypertrophy, not elsewhere classified	Arthritis Joint Other	Nociceptive Pain
M6720	Synovial hypertrophy, not elsewhere classified, unsp site	Arthritis Joint Other	Nociceptive Pain
M6721	Synovial hypertrophy, not elsewhere classified, shoulder	Arthritis Joint Upper Limb	Nociceptive Pain
M67211	Synovial hypertrophy, NEC, right shoulder	Arthritis Joint Upper Limb	Nociceptive Pain

© 2024 Peterson MD et al. *JAMA Neurology.*

M67212	Synovial hypertrophy, NEC, left shoulder	Arthritis Joint Upper Limb	Nociceptive Pain
M67219	Synovial hypertrophy, NEC, unsp shoulder	Arthritis Joint Upper Limb	Nociceptive Pain
M6722	Synovial hypertrophy, not elsewhere classified, upper arm	Arthritis Joint Upper Limb	Nociceptive Pain
M67221	Synovial hypertrophy, NEC, right upper arm	Arthritis Joint Upper Limb	Nociceptive Pain
M67222	Synovial hypertrophy, NEC, left upper arm	Arthritis Joint Upper Limb	Nociceptive Pain
M67229	Synovial hypertrophy, NEC, unsp upper arm	Arthritis Joint Upper Limb	Nociceptive Pain
M6723	Synovial hypertrophy, not elsewhere classified, forearm	Arthritis Joint Upper Limb	Nociceptive Pain
M67231	Synovial hypertrophy, NEC, right forearm	Arthritis Joint Upper Limb	Nociceptive Pain
M67232	Synovial hypertrophy, not elsewhere classified, left forearm	Arthritis Joint Upper Limb	Nociceptive Pain
M67239	Synovial hypertrophy, not elsewhere classified, unsp forearm	Arthritis Joint Upper Limb	Nociceptive Pain
M6724	Synovial hypertrophy, not elsewhere classified, hand	Arthritis Joint Upper Limb	Nociceptive Pain
M67241	Synovial hypertrophy, not elsewhere classified, right hand	Arthritis Joint Upper Limb	Nociceptive Pain
M67242	Synovial hypertrophy, not elsewhere classified, left hand	Arthritis Joint Upper Limb	Nociceptive Pain
M67249	Synovial hypertrophy, not elsewhere classified, unsp hand	Arthritis Joint Upper Limb	Nociceptive Pain
M6725	Synovial hypertrophy, not elsewhere classified, thigh	Arthritis Joint Lower Limb	Nociceptive Pain
M67251	Synovial hypertrophy, not elsewhere classified, right thigh	Arthritis Joint Lower Limb	Nociceptive Pain
M67252	Synovial hypertrophy, not elsewhere classified, left thigh	Arthritis Joint Lower Limb	Nociceptive Pain
M67259	Synovial hypertrophy, not elsewhere classified, unsp thigh	Arthritis Joint Lower Limb	Nociceptive Pain
M6726	Synovial hypertrophy, not elsewhere classified, lower leg	Arthritis Joint Lower Limb	Nociceptive Pain
M67261	Synovial hypertrophy, NEC, right lower leg	Arthritis Joint Lower Limb	Nociceptive Pain
M67262	Synovial hypertrophy, NEC, left lower leg	Arthritis Joint Lower Limb	Nociceptive Pain
M67269	Synovial hypertrophy, NEC, unsp lower leg	Arthritis Joint Lower Limb	Nociceptive Pain
M6727	Synovial hypertrophy, NEC, ankle and foot	Arthritis Joint Lower Limb	Nociceptive Pain
M67271	Synovial hypertrophy, NEC, right ankle and foot	Arthritis Joint Lower Limb	Nociceptive Pain
M67272	Synovial hypertrophy, NEC, left ankle and foot	Arthritis Joint Lower Limb	Nociceptive Pain
M67279	Synovial hypertrophy, NEC, unsp ankle and foot	Arthritis Joint Lower Limb	Nociceptive Pain
M6728	Synovial hypertrophy, not elsewhere classified, other site	Arthritis Joint Other	Nociceptive Pain
M6729	Synovial hypertrophy, NEC, multiple sites	Arthritis Joint Other	Nociceptive Pain
M673	Transient synovitis	Arthritis Joint Other	Nociceptive Pain
M6730	Transient synovitis, unspecified site	Arthritis Joint Other	Nociceptive Pain
M6731	Transient synovitis, shoulder	Arthritis Joint Upper Limb	Nociceptive Pain
M67311	Transient synovitis, right shoulder	Arthritis Joint Upper Limb	Nociceptive Pain
M67312	Transient synovitis, left shoulder	Arthritis Joint Upper Limb	Nociceptive Pain
M67319	Transient synovitis, unspecified shoulder	Arthritis Joint Upper Limb	Nociceptive Pain
M6732	Transient synovitis, elbow	Arthritis Joint Upper Limb	Nociceptive Pain
M67321	Transient synovitis, right elbow	Arthritis Joint Upper Limb	Nociceptive Pain
M67322	Transient synovitis, left elbow	Arthritis Joint Upper Limb	Nociceptive Pain

© 2024 Peterson MD et al. *JAMA Neurology.*

M67329	Transient synovitis, unspecified elbow	Arthritis Joint Upper Limb	Nociceptive Pain
M6733	Transient synovitis, wrist	Arthritis Joint Upper Limb	Nociceptive Pain
M67331	Transient synovitis, right wrist	Arthritis Joint Upper Limb	Nociceptive Pain
M67332	Transient synovitis, left wrist	Arthritis Joint Upper Limb	Nociceptive Pain
M67339	Transient synovitis, unspecified wrist	Arthritis Joint Upper Limb	Nociceptive Pain
M6734	Transient synovitis, hand	Arthritis Joint Upper Limb	Nociceptive Pain
M67341	Transient synovitis, right hand	Arthritis Joint Upper Limb	Nociceptive Pain
M67342	Transient synovitis, left hand	Arthritis Joint Upper Limb	Nociceptive Pain
M67349	Transient synovitis, unspecified hand	Arthritis Joint Upper Limb	Nociceptive Pain
M6735	Transient synovitis, hip	Arthritis Joint Spine and Hips	Nociceptive Pain
M67351	Transient synovitis, right hip	Arthritis Joint Spine and Hips	Nociceptive Pain
M67352	Transient synovitis, left hip	Arthritis Joint Spine and Hips	Nociceptive Pain
M67359	Transient synovitis, unspecified hip	Arthritis Joint Spine and Hips	Nociceptive Pain
M6736	Transient synovitis, knee	Arthritis Joint Lower Limb	Nociceptive Pain
M67361	Transient synovitis, right knee	Arthritis Joint Lower Limb	Nociceptive Pain
M67362	Transient synovitis, left knee	Arthritis Joint Lower Limb	Nociceptive Pain
M67369	Transient synovitis, unspecified knee	Arthritis Joint Lower Limb	Nociceptive Pain
M6737	Transient synovitis, ankle and foot	Arthritis Joint Lower Limb	Nociceptive Pain
M67371	Transient synovitis, right ankle and foot	Arthritis Joint Lower Limb	Nociceptive Pain
M67372	Transient synovitis, left ankle and foot	Arthritis Joint Lower Limb	Nociceptive Pain
M67379	Transient synovitis, unspecified ankle and foot	Arthritis Joint Lower Limb	Nociceptive Pain
M6738	Transient synovitis, other site	Arthritis Joint Other	Nociceptive Pain
M6739	Transient synovitis, multiple sites	Arthritis Joint Other	Nociceptive Pain
M674	Ganglion	Arthritis Joint Other	Nociceptive Pain
M6740	Ganglion, unspecified site	Arthritis Joint Other	Nociceptive Pain
M6741	Ganglion, shoulder	Arthritis Joint Upper Limb	Nociceptive Pain
M67411	Ganglion, right shoulder	Arthritis Joint Upper Limb	Nociceptive Pain
M67412	Ganglion, left shoulder	Arthritis Joint Upper Limb	Nociceptive Pain
M67419	Ganglion, unspecified shoulder	Arthritis Joint Upper Limb	Nociceptive Pain
M6742	Ganglion, elbow	Arthritis Joint Upper Limb	Nociceptive Pain
M67421	Ganglion, right elbow	Arthritis Joint Upper Limb	Nociceptive Pain
M67422	Ganglion, left elbow	Arthritis Joint Upper Limb	Nociceptive Pain
M67429	Ganglion, unspecified elbow	Arthritis Joint Upper Limb	Nociceptive Pain
M6743	Ganglion, wrist	Arthritis Joint Upper Limb	Nociceptive Pain
M67431	Ganglion, right wrist	Arthritis Joint Upper Limb	Nociceptive Pain
M67432	Ganglion, left wrist	Arthritis Joint Upper Limb	Nociceptive Pain
M67439	Ganglion, unspecified wrist	Arthritis Joint Upper Limb	Nociceptive Pain

© 2024 Peterson MD et al. *JAMA Neurology.*

M6744	Ganglion, hand	Arthritis Joint Upper Limb	Nociceptive Pain
M67441	Ganglion, right hand	Arthritis Joint Upper Limb	Nociceptive Pain
M67442	Ganglion, left hand	Arthritis Joint Upper Limb	Nociceptive Pain
M67449	Ganglion, unspecified hand	Arthritis Joint Upper Limb	Nociceptive Pain
M6745	Ganglion, hip	Arthritis Joint Spine and Hips	Nociceptive Pain
M67451	Ganglion, right hip	Arthritis Joint Spine and Hips	Nociceptive Pain
M67452	Ganglion, left hip	Arthritis Joint Spine and Hips	Nociceptive Pain
M67459	Ganglion, unspecified hip	Arthritis Joint Spine and Hips	Nociceptive Pain
M6746	Ganglion, knee	Arthritis Joint Lower Limb	Nociceptive Pain
M67461	Ganglion, right knee	Arthritis Joint Lower Limb	Nociceptive Pain
M67462	Ganglion, left knee	Arthritis Joint Lower Limb	Nociceptive Pain
M67469	Ganglion, unspecified knee	Arthritis Joint Lower Limb	Nociceptive Pain
M6747	Ganglion, ankle and foot	Arthritis Joint Lower Limb	Nociceptive Pain
M67471	Ganglion, right ankle and foot	Arthritis Joint Lower Limb	Nociceptive Pain
M67472	Ganglion, left ankle and foot	Arthritis Joint Lower Limb	Nociceptive Pain
M67479	Ganglion, unspecified ankle and foot	Arthritis Joint Lower Limb	Nociceptive Pain
M6748	Ganglion, other site	Arthritis Joint Other	Nociceptive Pain
M6749	Ganglion, multiple sites	Arthritis Joint Other	Nociceptive Pain
M675	Plica syndrome	Arthritis Joint Other	Nociceptive Pain
M6750	Plica syndrome, unspecified knee	Arthritis Joint Lower Limb	Nociceptive Pain
M6751	Plica syndrome, right knee	Arthritis Joint Lower Limb	Nociceptive Pain
M6752	Plica syndrome, left knee	Arthritis Joint Lower Limb	Nociceptive Pain
M678	Other specified disorders of synovium and tendon	Arthritis Joint Other	Nociceptive Pain
M6780	Oth disrd of synovium and tendon, unspecified site	Arthritis Joint Other	Nociceptive Pain
M6781	Other specified disorders of synovium and tendon, shoulder	Arthritis Joint Upper Limb	Nociceptive Pain
M67811	Other specified disorders of synovium, right shoulder	Arthritis Joint Upper Limb	Nociceptive Pain
M67812	Other specified disorders of synovium, left shoulder	Arthritis Joint Upper Limb	Nociceptive Pain
M67813	Other specified disorders of tendon, right shoulder	Arthritis Joint Upper Limb	Nociceptive Pain
M67814	Other specified disorders of tendon, left shoulder	Arthritis Joint Upper Limb	Nociceptive Pain
M67819	Oth disrd of synovium and tendon, unspecified shoulder	Arthritis Joint Upper Limb	Nociceptive Pain
M6782	Other specified disorders of synovium and tendon, elbow	Arthritis Joint Upper Limb	Nociceptive Pain
M67821	Other specified disorders of synovium, right elbow	Arthritis Joint Upper Limb	Nociceptive Pain
M67822	Other specified disorders of synovium, left elbow	Arthritis Joint Upper Limb	Nociceptive Pain
M67823	Other specified disorders of tendon, right elbow	Arthritis Joint Upper Limb	Nociceptive Pain
M67824	Other specified disorders of tendon, left elbow	Arthritis Joint Upper Limb	Nociceptive Pain
M67829	Oth disrd of synovium and tendon, unspecified elbow	Arthritis Joint Upper Limb	Nociceptive Pain
M6783	Other specified disorders of synovium and tendon, wrist	Arthritis Joint Upper Limb	Nociceptive Pain

© 2024 Peterson MD et al. *JAMA Neurology.*

M67831	Other specified disorders of synovium, right wrist	Arthritis Joint Upper Limb	Nociceptive Pain
M67832	Other specified disorders of synovium, left wrist	Arthritis Joint Upper Limb	Nociceptive Pain
M67833	Other specified disorders of tendon, right wrist	Arthritis Joint Upper Limb	Nociceptive Pain
M67834	Other specified disorders of tendon, left wrist	Arthritis Joint Upper Limb	Nociceptive Pain
M67839	Oth disrd of synovium and tendon, unspecified wrist	Arthritis Joint Upper Limb	Nociceptive Pain
M6784	Other specified disorders of synovium and tendon, hand	Arthritis Joint Upper Limb	Nociceptive Pain
M67841	Other specified disorders of synovium, right hand	Arthritis Joint Upper Limb	Nociceptive Pain
M67842	Other specified disorders of synovium, left hand	Arthritis Joint Upper Limb	Nociceptive Pain
M67843	Other specified disorders of tendon, right hand	Arthritis Joint Upper Limb	Nociceptive Pain
M67844	Other specified disorders of tendon, left hand	Arthritis Joint Upper Limb	Nociceptive Pain
M67849	Oth disrd of synovium and tendon, unspecified hand	Arthritis Joint Upper Limb	Nociceptive Pain
M6785	Other specified disorders of synovium and tendon, hip	Arthritis Joint Spine and Hips	Nociceptive Pain
M67851	Other specified disorders of synovium, right hip	Arthritis Joint Spine and Hips	Nociceptive Pain
M67852	Other specified disorders of synovium, left hip	Arthritis Joint Spine and Hips	Nociceptive Pain
M67853	Other specified disorders of tendon, right hip	Arthritis Joint Spine and Hips	Nociceptive Pain
M67854	Other specified disorders of tendon, left hip	Arthritis Joint Spine and Hips	Nociceptive Pain
M67859	Oth disrd of synovium and tendon, unspecified hip	Arthritis Joint Spine and Hips	Nociceptive Pain
M6786	Other specified disorders of synovium and tendon, knee	Arthritis Joint Lower Limb	Nociceptive Pain
M67861	Other specified disorders of synovium, right knee	Arthritis Joint Lower Limb	Nociceptive Pain
M67862	Other specified disorders of synovium, left knee	Arthritis Joint Lower Limb	Nociceptive Pain
M67863	Other specified disorders of tendon, right knee	Arthritis Joint Lower Limb	Nociceptive Pain
M67864	Other specified disorders of tendon, left knee	Arthritis Joint Lower Limb	Nociceptive Pain
M67869	Oth disrd of synovium and tendon, unspecified knee	Arthritis Joint Lower Limb	Nociceptive Pain
M6787	Oth disrd of synovium and tendon, ankle and foot	Arthritis Joint Lower Limb	Nociceptive Pain
M67871	Other specified disorders of synovium, right ankle and foot	Arthritis Joint Lower Limb	Nociceptive Pain
M67872	Other specified disorders of synovium, left ankle and foot	Arthritis Joint Lower Limb	Nociceptive Pain
M67873	Other specified disorders of tendon, right ankle and foot	Arthritis Joint Lower Limb	Nociceptive Pain
M67874	Other specified disorders of tendon, left ankle and foot	Arthritis Joint Lower Limb	Nociceptive Pain
M67879	Oth disrd of synovium and tendon, unspecified ankle and foot	Arthritis Joint Lower Limb	Nociceptive Pain
M6788	Other specified disorders of synovium and tendon, other site	Arthritis Joint Other	Nociceptive Pain
M6789	Oth disrd of synovium and tendon, multiple sites	Arthritis Joint Other	Nociceptive Pain
M679	Unspecified disorder of synovium and tendon	Arthritis Joint Other	Nociceptive Pain
M6790	Unsp disorder of synovium and tendon, unspecified site	Arthritis Joint Other	Nociceptive Pain
M6791	Unspecified disorder of synovium and tendon, shoulder	Arthritis Joint Upper Limb	Nociceptive Pain
M67911	Unspecified disorder of synovium and tendon, right shoulder	Arthritis Joint Upper Limb	Nociceptive Pain
M67912	Unspecified disorder of synovium and tendon, left shoulder	Arthritis Joint Upper Limb	Nociceptive Pain
M67919	Unsp disorder of synovium and tendon, unspecified shoulder	Arthritis Joint Upper Limb	Nociceptive Pain

© 2024 Peterson MD et al. *JAMA Neurology.*

M6792	Unspecified disorder of synovium and tendon, upper arm	Arthritis Joint Upper Limb	Nociceptive Pain
M67921	Unspecified disorder of synovium and tendon, right upper arm	Arthritis Joint Upper Limb	Nociceptive Pain
M67922	Unspecified disorder of synovium and tendon, left upper arm	Arthritis Joint Upper Limb	Nociceptive Pain
M67929	Unsp disorder of synovium and tendon, unspecified upper arm	Arthritis Joint Upper Limb	Nociceptive Pain
M6793	Unspecified disorder of synovium and tendon, forearm	Arthritis Joint Upper Limb	Nociceptive Pain
M67931	Unspecified disorder of synovium and tendon, right forearm	Arthritis Joint Upper Limb	Nociceptive Pain
M67932	Unspecified disorder of synovium and tendon, left forearm	Arthritis Joint Upper Limb	Nociceptive Pain
M67939	Unsp disorder of synovium and tendon, unspecified forearm	Arthritis Joint Upper Limb	Nociceptive Pain
M6794	Unspecified disorder of synovium and tendon, hand	Arthritis Joint Upper Limb	Nociceptive Pain
M67941	Unspecified disorder of synovium and tendon, right hand	Arthritis Joint Upper Limb	Nociceptive Pain
M67942	Unspecified disorder of synovium and tendon, left hand	Arthritis Joint Upper Limb	Nociceptive Pain
M67949	Unsp disorder of synovium and tendon, unspecified hand	Arthritis Joint Upper Limb	Nociceptive Pain
M6795	Unspecified disorder of synovium and tendon, thigh	Arthritis Joint Lower Limb	Nociceptive Pain
M67951	Unspecified disorder of synovium and tendon, right thigh	Arthritis Joint Lower Limb	Nociceptive Pain
M67952	Unspecified disorder of synovium and tendon, left thigh	Arthritis Joint Lower Limb	Nociceptive Pain
M67959	Unsp disorder of synovium and tendon, unspecified thigh	Arthritis Joint Lower Limb	Nociceptive Pain
M6796	Unspecified disorder of synovium and tendon, lower leg	Arthritis Joint Lower Limb	Nociceptive Pain
M67961	Unspecified disorder of synovium and tendon, right lower leg	Arthritis Joint Lower Limb	Nociceptive Pain
M67962	Unspecified disorder of synovium and tendon, left lower leg	Arthritis Joint Lower Limb	Nociceptive Pain
M67969	Unsp disorder of synovium and tendon, unspecified lower leg	Arthritis Joint Lower Limb	Nociceptive Pain
M6797	Unspecified disorder of synovium and tendon, ankle and foot	Arthritis Joint Lower Limb	Nociceptive Pain
M67971	Unsp disorder of synovium and tendon, right ankle and foot	Arthritis Joint Lower Limb	Nociceptive Pain
M67972	Unsp disorder of synovium and tendon, left ankle and foot	Arthritis Joint Lower Limb	Nociceptive Pain
M67979	Unsp disorder of synovium and tendon, unsp ankle and foot	Arthritis Joint Lower Limb	Nociceptive Pain
M6798	Unspecified disorder of synovium and tendon, other site	Arthritis Joint Other	Nociceptive Pain
M6799	Unspecified disorder of synovium and tendon, multiple sites	Arthritis Joint Other	Nociceptive Pain
M70	Soft tissue disorders related to use, overuse and pressure	Arthritis Joint Other	Nociceptive Pain
M700	Crepitant synovitis (acute) (chronic) of hand and wrist	Arthritis Joint Other	Nociceptive Pain
M7003	Crepitant synovitis (acute) (chronic), wrist	Arthritis Joint Upper Limb	Nociceptive Pain
M70031	Crepitant synovitis (acute) (chronic), right wrist	Arthritis Joint Upper Limb	Nociceptive Pain
M70032	Crepitant synovitis (acute) (chronic), left wrist	Arthritis Joint Upper Limb	Nociceptive Pain
M70039	Crepitant synovitis (acute) (chronic), unspecified wrist	Arthritis Joint Upper Limb	Nociceptive Pain
M7004	Crepitant synovitis (acute) (chronic), hand	Arthritis Joint Upper Limb	Nociceptive Pain
M70041	Crepitant synovitis (acute) (chronic), right hand	Arthritis Joint Upper Limb	Nociceptive Pain
M70042	Crepitant synovitis (acute) (chronic), left hand	Arthritis Joint Upper Limb	Nociceptive Pain
M70049	Crepitant synovitis (acute) (chronic), unspecified hand	Arthritis Joint Upper Limb	Nociceptive Pain
M701	Bursitis of hand	Arthritis Joint Upper Limb	Nociceptive Pain

© 2024 Peterson MD et al. *JAMA Neurology.*

M7010	Bursitis, unspecified hand	Arthritis Joint Upper Limb	Nociceptive Pain
M7011	Bursitis, right hand	Arthritis Joint Upper Limb	Nociceptive Pain
M7012	Bursitis, left hand	Arthritis Joint Upper Limb	Nociceptive Pain
M702	Olecranon bursitis	Arthritis Joint Upper Limb	Nociceptive Pain
M7020	Olecranon bursitis, unspecified elbow	Arthritis Joint Upper Limb	Nociceptive Pain
M7021	Olecranon bursitis, right elbow	Arthritis Joint Upper Limb	Nociceptive Pain
M7022	Olecranon bursitis, left elbow	Arthritis Joint Upper Limb	Nociceptive Pain
M703	Other bursitis of elbow	Arthritis Joint Upper Limb	Nociceptive Pain
M7030	Other bursitis of elbow, unspecified elbow	Arthritis Joint Upper Limb	Nociceptive Pain
M7031	Other bursitis of elbow, right elbow	Arthritis Joint Upper Limb	Nociceptive Pain
M7032	Other bursitis of elbow, left elbow	Arthritis Joint Upper Limb	Nociceptive Pain
M704	Prepatellar bursitis	Arthritis Joint Lower Limb	Nociceptive Pain
M7040	Prepatellar bursitis, unspecified knee	Arthritis Joint Lower Limb	Nociceptive Pain
M7041	Prepatellar bursitis, right knee	Arthritis Joint Lower Limb	Nociceptive Pain
M7042	Prepatellar bursitis, left knee	Arthritis Joint Lower Limb	Nociceptive Pain
M705	Other bursitis of knee	Arthritis Joint Lower Limb	Nociceptive Pain
M7050	Other bursitis of knee, unspecified knee	Arthritis Joint Lower Limb	Nociceptive Pain
M7051	Other bursitis of knee, right knee	Arthritis Joint Lower Limb	Nociceptive Pain
M7052	Other bursitis of knee, left knee	Arthritis Joint Lower Limb	Nociceptive Pain
M706	Trochanteric bursitis	Arthritis Joint Lower Limb	Nociceptive Pain
M7060	Trochanteric bursitis, unspecified hip	Arthritis Joint Spine and Hips	Nociceptive Pain
M7061	Trochanteric bursitis, right hip	Arthritis Joint Spine and Hips	Nociceptive Pain
M7062	Trochanteric bursitis, left hip	Arthritis Joint Spine and Hips	Nociceptive Pain
M707	Other bursitis of hip	Arthritis Joint Spine and Hips	Nociceptive Pain
M7070	Other bursitis of hip, unspecified hip	Arthritis Joint Spine and Hips	Nociceptive Pain
M7071	Other bursitis of hip, right hip	Arthritis Joint Spine and Hips	Nociceptive Pain
M7072	Other bursitis of hip, left hip	Arthritis Joint Spine and Hips	Nociceptive Pain
M708	Oth soft tissue disorders related to use/pressure	Arthritis Joint Other	Nociceptive Pain
M7080	Oth soft tissue disord related to use/pressure of unsp site	Arthritis Joint Other	Nociceptive Pain
M7081	Oth soft tissue disord related to use/pressure of shoulder	Arthritis Joint Upper Limb	Nociceptive Pain
M70811	Oth soft tissue disord related to use/pressure, r shoulder	Arthritis Joint Upper Limb	Nociceptive Pain
M70812	Oth soft tissue disord related to use/pressure, l shoulder	Arthritis Joint Upper Limb	Nociceptive Pain
M70819	Oth soft tissue disord related to use/pressure, unsp shldr	Arthritis Joint Upper Limb	Nociceptive Pain
M7082	Oth soft tissue disord related to use/pressure of upper arm	Arthritis Joint Upper Limb	Nociceptive Pain
M70821	Oth soft tissue disorders related to use/pressure, r up arm	Arthritis Joint Upper Limb	Nociceptive Pain
M70822	Oth soft tissue disorders related to use/pressure, l up arm	Arthritis Joint Upper Limb	Nociceptive Pain
M70829	Oth soft tissue disord rel to use/pressure, unsp upper arms	Arthritis Joint Upper Limb	Nociceptive Pain

© 2024 Peterson MD et al. *JAMA Neurology.*

M7083	Oth soft tissue disorders related to use/pressure of forearm	Arthritis Joint Upper Limb	Nociceptive Pain
M70831	Oth soft tissue disorders related to use/pressure, r forearm	Arthritis Joint Upper Limb	Nociceptive Pain
M70832	Oth soft tissue disorders related to use/pressure, l forearm	Arthritis Joint Upper Limb	Nociceptive Pain
M70839	Oth soft tissue disord related to use/pressure, unsp forearm	Arthritis Joint Upper Limb	Nociceptive Pain
M7084	Oth soft tissue disorders related to use/pressure of hand	Arthritis Joint Upper Limb	Nociceptive Pain
M70841	Oth soft tissue disorders related to use/pressure, r hand	Arthritis Joint Upper Limb	Nociceptive Pain
M70842	Oth soft tissue disorders related to use/pressure, left hand	Arthritis Joint Upper Limb	Nociceptive Pain
M70849	Oth soft tissue disorders related to use/pressure, unsp hand	Arthritis Joint Upper Limb	Nociceptive Pain
M7085	Oth soft tissue disorders related to use/pressure of thigh	Arthritis Joint Lower Limb	Nociceptive Pain
M70851	Oth soft tissue disord related to use/pressure, right thigh	Arthritis Joint Lower Limb	Nociceptive Pain
M70852	Oth soft tissue disord related to use/pressure, left thigh	Arthritis Joint Lower Limb	Nociceptive Pain
M70859	Oth soft tissue disord related to use/pressure, unsp thigh	Arthritis Joint Lower Limb	Nociceptive Pain
M7086	Oth soft tissue disorders related to use/pressure lower leg	Arthritis Joint Lower Limb	Nociceptive Pain
M70861	Oth soft tissue disorders related to use/pressure, r low leg	Arthritis Joint Lower Limb	Nociceptive Pain
M70862	Oth soft tissue disorders related to use/pressure, l low leg	Arthritis Joint Lower Limb	Nociceptive Pain
M70869	Oth soft tissue disorders related to use/pressure, unsp leg	Arthritis Joint Lower Limb	Nociceptive Pain
M7087	Oth soft tissue disorders related to use/pressure of ank/ft	Arthritis Joint Lower Limb	Nociceptive Pain
M70871	Oth soft tissue disord related to use/pressure, right ank/ft	Arthritis Joint Lower Limb	Nociceptive Pain
M70872	Oth soft tissue disord related to use/pressure, left ank/ft	Arthritis Joint Lower Limb	Nociceptive Pain
M70879	Oth soft tissue disord related to use/pressure, unsp ank/ft	Arthritis Joint Lower Limb	Nociceptive Pain
M7088	Oth soft tissue disorders related to use/pressure oth site	Arthritis Joint Other	Nociceptive Pain
M7089	Oth soft tissue disord related to use/pressure mult sites	Arthritis Joint Other	Nociceptive Pain
M709	Unsp soft tissue disorder related to use/pressure	Arthritis Joint Other	Nociceptive Pain
M7090	Unsp soft tissue disord related to use/pressure of unsp site	Arthritis Joint Other	Nociceptive Pain
M7091	Unsp soft tissue disord related to use/pressure of shoulder	Arthritis Joint Upper Limb	Nociceptive Pain
M70911	Unsp soft tissue disord related to use/pressure, r shoulder	Arthritis Joint Upper Limb	Nociceptive Pain
M70912	Unsp soft tissue disord related to use/pressure, l shoulder	Arthritis Joint Upper Limb	Nociceptive Pain
M70919	Unsp soft tissue disord related to use/pressure, unsp shldr	Arthritis Joint Upper Limb	Nociceptive Pain
M7092	Unsp soft tissue disorder related to use/pressure of up arm	Arthritis Joint Upper Limb	Nociceptive Pain
M70921	Unsp soft tissue disorder related to use/pressure, r up arm	Arthritis Joint Upper Limb	Nociceptive Pain
M70922	Unsp soft tissue disorder related to use/pressure, l up arm	Arthritis Joint Upper Limb	Nociceptive Pain
M70929	Unsp soft tissue disord related to use/pressure, unsp up arm	Arthritis Joint Upper Limb	Nociceptive Pain
M7093	Unsp soft tissue disorder related to use/pressure of forearm	Arthritis Joint Upper Limb	Nociceptive Pain
M70931	Unsp soft tissue disorder related to use/pressure, r forearm	Arthritis Joint Upper Limb	Nociceptive Pain
M70932	Unsp soft tissue disorder related to use/pressure, l forearm	Arthritis Joint Upper Limb	Nociceptive Pain
M70939	Unsp soft tissue disord rel to use/pressure, unsp forearm	Arthritis Joint Upper Limb	Nociceptive Pain
M7094	Unsp soft tissue disorder related to use/pressure of hand	Arthritis Joint Upper Limb	Nociceptive Pain

© 2024 Peterson MD et al. *JAMA Neurology.*

M70941	Unsp soft tissue disorder related to use/pressure, r hand	Arthritis Joint Upper Limb	Nociceptive Pain
M70942	Unsp soft tissue disorder related to use/pressure, left hand	Arthritis Joint Upper Limb	Nociceptive Pain
M70949	Unsp soft tissue disorder related to use/pressure, unsp hand	Arthritis Joint Upper Limb	Nociceptive Pain
M7095	Unsp soft tissue disorder related to use/pressure of thigh	Arthritis Joint Lower Limb	Nociceptive Pain
M70951	Unsp soft tissue disord related to use/pressure, right thigh	Arthritis Joint Lower Limb	Nociceptive Pain
M70952	Unsp soft tissue disord related to use/pressure, left thigh	Arthritis Joint Lower Limb	Nociceptive Pain
M70959	Unsp soft tissue disord related to use/pressure, unsp thigh	Arthritis Joint Lower Limb	Nociceptive Pain
M7096	Unsp soft tissue disorder related to use/pressure lower leg	Arthritis Joint Lower Limb	Nociceptive Pain
M70961	Unsp soft tissue disorder related to use/pressure, r low leg	Arthritis Joint Lower Limb	Nociceptive Pain
M70962	Unsp soft tissue disorder related to use/pressure, l low leg	Arthritis Joint Lower Limb	Nociceptive Pain
M70969	Unsp soft tissue disord rel to use/pressure, unsp low leg	Arthritis Joint Lower Limb	Nociceptive Pain
M7097	Unsp soft tissue disorder related to use/pressure of ank/ft	Arthritis Joint Lower Limb	Nociceptive Pain
M70971	Unsp soft tissue disord rel to use/pressure, right ank/ft	Arthritis Joint Lower Limb	Nociceptive Pain
M70972	Unsp soft tissue disord related to use/pressure, left ank/ft	Arthritis Joint Lower Limb	Nociceptive Pain
M70979	Unsp soft tissue disord related to use/pressure, unsp ank/ft	Arthritis Joint Lower Limb	Nociceptive Pain
M7098	Unsp soft tissue disorder related to use/pressure oth	Arthritis Joint Other	Nociceptive Pain
M7099	Unsp soft tissue disord related to use/pressure mult sites	Arthritis Joint Other	Nociceptive Pain
M71	Other bursopathies	Arthritis Joint Other	Nociceptive Pain
M710	Abscess of bursa	Arthritis Joint Other	Nociceptive Pain
M7100	Abscess of bursa, unspecified site	Arthritis Joint Other	Nociceptive Pain
M7101	Abscess of bursa, shoulder	Arthritis Joint Upper Limb	Nociceptive Pain
M71011	Abscess of bursa, right shoulder	Arthritis Joint Upper Limb	Nociceptive Pain
M71012	Abscess of bursa, left shoulder	Arthritis Joint Upper Limb	Nociceptive Pain
M71019	Abscess of bursa, unspecified shoulder	Arthritis Joint Upper Limb	Nociceptive Pain
M7102	Abscess of bursa, elbow	Arthritis Joint Upper Limb	Nociceptive Pain
M71021	Abscess of bursa, right elbow	Arthritis Joint Upper Limb	Nociceptive Pain
M71022	Abscess of bursa, left elbow	Arthritis Joint Upper Limb	Nociceptive Pain
M71029	Abscess of bursa, unspecified elbow	Arthritis Joint Upper Limb	Nociceptive Pain
M7103	Abscess of bursa, wrist	Arthritis Joint Upper Limb	Nociceptive Pain
M71031	Abscess of bursa, right wrist	Arthritis Joint Upper Limb	Nociceptive Pain
M71032	Abscess of bursa, left wrist	Arthritis Joint Upper Limb	Nociceptive Pain
M71039	Abscess of bursa, unspecified wrist	Arthritis Joint Upper Limb	Nociceptive Pain
M7104	Abscess of bursa, hand	Arthritis Joint Upper Limb	Nociceptive Pain
M71041	Abscess of bursa, right hand	Arthritis Joint Upper Limb	Nociceptive Pain
M71042	Abscess of bursa, left hand	Arthritis Joint Upper Limb	Nociceptive Pain
M71049	Abscess of bursa, unspecified hand	Arthritis Joint Upper Limb	Nociceptive Pain
M7105	Abscess of bursa, hip	Arthritis Joint Spine and Hips	Nociceptive Pain

© 2024 Peterson MD et al. *JAMA Neurology.*

M71051	Abscess of bursa, right hip	Arthritis Joint Spine and Hips	Nociceptive Pain
M71052	Abscess of bursa, left hip	Arthritis Joint Spine and Hips	Nociceptive Pain
M71059	Abscess of bursa, unspecified hip	Arthritis Joint Spine and Hips	Nociceptive Pain
M7106	Abscess of bursa, knee	Arthritis Joint Lower Limb	Nociceptive Pain
M71061	Abscess of bursa, right knee	Arthritis Joint Lower Limb	Nociceptive Pain
M71062	Abscess of bursa, left knee	Arthritis Joint Lower Limb	Nociceptive Pain
M71069	Abscess of bursa, unspecified knee	Arthritis Joint Lower Limb	Nociceptive Pain
M7107	Abscess of bursa, ankle and foot	Arthritis Joint Lower Limb	Nociceptive Pain
M71071	Abscess of bursa, right ankle and foot	Arthritis Joint Lower Limb	Nociceptive Pain
M71072	Abscess of bursa, left ankle and foot	Arthritis Joint Lower Limb	Nociceptive Pain
M71079	Abscess of bursa, unspecified ankle and foot	Arthritis Joint Lower Limb	Nociceptive Pain
M7108	Abscess of bursa, other site	Arthritis Joint Other	Nociceptive Pain
M7109	Abscess of bursa, multiple sites	Arthritis Joint Other	Nociceptive Pain
M711	Other infective bursitis	Arthritis Joint Other	Nociceptive Pain
M7110	Other infective bursitis, unspecified site	Arthritis Joint Other	Nociceptive Pain
M7111	Other infective bursitis, shoulder	Arthritis Joint Upper Limb	Nociceptive Pain
M71111	Other infective bursitis, right shoulder	Arthritis Joint Upper Limb	Nociceptive Pain
M71112	Other infective bursitis, left shoulder	Arthritis Joint Upper Limb	Nociceptive Pain
M71119	Other infective bursitis, unspecified shoulder	Arthritis Joint Upper Limb	Nociceptive Pain
M7112	Other infective bursitis, elbow	Arthritis Joint Upper Limb	Nociceptive Pain
M71121	Other infective bursitis, right elbow	Arthritis Joint Upper Limb	Nociceptive Pain
M71122	Other infective bursitis, left elbow	Arthritis Joint Upper Limb	Nociceptive Pain
M71129	Other infective bursitis, unspecified elbow	Arthritis Joint Upper Limb	Nociceptive Pain
M7113	Other infective bursitis, wrist	Arthritis Joint Upper Limb	Nociceptive Pain
M71131	Other infective bursitis, right wrist	Arthritis Joint Upper Limb	Nociceptive Pain
M71132	Other infective bursitis, left wrist	Arthritis Joint Upper Limb	Nociceptive Pain
M71139	Other infective bursitis, unspecified wrist	Arthritis Joint Upper Limb	Nociceptive Pain
M7114	Other infective bursitis, hand	Arthritis Joint Upper Limb	Nociceptive Pain
M71141	Other infective bursitis, right hand	Arthritis Joint Upper Limb	Nociceptive Pain
M71142	Other infective bursitis, left hand	Arthritis Joint Upper Limb	Nociceptive Pain
M71149	Other infective bursitis, unspecified hand	Arthritis Joint Upper Limb	Nociceptive Pain
M7115	Other infective bursitis, hip	Arthritis Joint Spine and Hips	Nociceptive Pain
M71151	Other infective bursitis, right hip	Arthritis Joint Spine and Hips	Nociceptive Pain
M71152	Other infective bursitis, left hip	Arthritis Joint Spine and Hips	Nociceptive Pain
M71159	Other infective bursitis, unspecified hip	Arthritis Joint Spine and Hips	Nociceptive Pain
M7116	Other infective bursitis, knee	Arthritis Joint Lower Limb	Nociceptive Pain
M71161	Other infective bursitis, right knee	Arthritis Joint Lower Limb	Nociceptive Pain

© 2024 Peterson MD et al. *JAMA Neurology.*

M71162	Other infective bursitis, left knee	Arthritis Joint Lower Limb	Nociceptive Pain
M71169	Other infective bursitis, unspecified knee	Arthritis Joint Lower Limb	Nociceptive Pain
M7117	Other infective bursitis, ankle and foot	Arthritis Joint Lower Limb	Nociceptive Pain
M71171	Other infective bursitis, right ankle and foot	Arthritis Joint Lower Limb	Nociceptive Pain
M71172	Other infective bursitis, left ankle and foot	Arthritis Joint Lower Limb	Nociceptive Pain
M71179	Other infective bursitis, unspecified ankle and foot	Arthritis Joint Lower Limb	Nociceptive Pain
M7118	Other infective bursitis, other site	Arthritis Joint Other	Nociceptive Pain
M7119	Other infective bursitis, multiple sites	Arthritis Joint Other	Nociceptive Pain
M712	Synovial cyst of popliteal space [Baker]	Arthritis Joint Other	Nociceptive Pain
M7120	Synovial cyst of popliteal space [Baker], unspecified knee	Arthritis Joint Lower Limb	Nociceptive Pain
M7121	Synovial cyst of popliteal space [Baker], right knee	Arthritis Joint Lower Limb	Nociceptive Pain
M7122	Synovial cyst of popliteal space [Baker], left knee	Arthritis Joint Lower Limb	Nociceptive Pain
M713	Other bursal cyst	Arthritis Joint Other	Nociceptive Pain
M7130	Other bursal cyst, unspecified site	Arthritis Joint Other	Nociceptive Pain
M7131	Other bursal cyst, shoulder	Arthritis Joint Upper Limb	Nociceptive Pain
M71311	Other bursal cyst, right shoulder	Arthritis Joint Upper Limb	Nociceptive Pain
M71312	Other bursal cyst, left shoulder	Arthritis Joint Upper Limb	Nociceptive Pain
M71319	Other bursal cyst, unspecified shoulder	Arthritis Joint Upper Limb	Nociceptive Pain
M7132	Other bursal cyst, elbow	Arthritis Joint Upper Limb	Nociceptive Pain
M71321	Other bursal cyst, right elbow	Arthritis Joint Upper Limb	Nociceptive Pain
M71322	Other bursal cyst, left elbow	Arthritis Joint Upper Limb	Nociceptive Pain
M71329	Other bursal cyst, unspecified elbow	Arthritis Joint Upper Limb	Nociceptive Pain
M7133	Other bursal cyst, wrist	Arthritis Joint Upper Limb	Nociceptive Pain
M71331	Other bursal cyst, right wrist	Arthritis Joint Upper Limb	Nociceptive Pain
M71332	Other bursal cyst, left wrist	Arthritis Joint Upper Limb	Nociceptive Pain
M71339	Other bursal cyst, unspecified wrist	Arthritis Joint Upper Limb	Nociceptive Pain
M7134	Other bursal cyst, hand	Arthritis Joint Upper Limb	Nociceptive Pain
M71341	Other bursal cyst, right hand	Arthritis Joint Upper Limb	Nociceptive Pain
M71342	Other bursal cyst, left hand	Arthritis Joint Upper Limb	Nociceptive Pain
M71349	Other bursal cyst, unspecified hand	Arthritis Joint Upper Limb	Nociceptive Pain
M7135	Other bursal cyst, hip	Arthritis Joint Spine and Hips	Nociceptive Pain
M71351	Other bursal cyst, right hip	Arthritis Joint Spine and Hips	Nociceptive Pain
M71352	Other bursal cyst, left hip	Arthritis Joint Spine and Hips	Nociceptive Pain
M71359	Other bursal cyst, unspecified hip	Arthritis Joint Spine and Hips	Nociceptive Pain
M7137	Other bursal cyst, ankle and foot	Arthritis Joint Lower Limb	Nociceptive Pain
M71371	Other bursal cyst, right ankle and foot	Arthritis Joint Lower Limb	Nociceptive Pain
M71372	Other bursal cyst, left ankle and foot	Arthritis Joint Lower Limb	Nociceptive Pain

© 2024 Peterson MD et al. *JAMA Neurology.*

M71379	Other bursal cyst, unspecified ankle and foot	Arthritis Joint Lower Limb	Nociceptive Pain
M7138	Other bursal cyst, other site	Arthritis Joint Other	Nociceptive Pain
M7139	Other bursal cyst, multiple sites	Arthritis Joint Other	Nociceptive Pain
M714	Calcium deposit in bursa	Arthritis Joint Other	Nociceptive Pain
M7140	Calcium deposit in bursa, unspecified site	Arthritis Joint Other	Nociceptive Pain
M7142	Calcium deposit in bursa, elbow	Arthritis Joint Upper Limb	Nociceptive Pain
M71421	Calcium deposit in bursa, right elbow	Arthritis Joint Upper Limb	Nociceptive Pain
M71422	Calcium deposit in bursa, left elbow	Arthritis Joint Upper Limb	Nociceptive Pain
M71429	Calcium deposit in bursa, unspecified elbow	Arthritis Joint Upper Limb	Nociceptive Pain
M7143	Calcium deposit in bursa, wrist	Arthritis Joint Upper Limb	Nociceptive Pain
M71431	Calcium deposit in bursa, right wrist	Arthritis Joint Upper Limb	Nociceptive Pain
M71432	Calcium deposit in bursa, left wrist	Arthritis Joint Upper Limb	Nociceptive Pain
M71439	Calcium deposit in bursa, unspecified wrist	Arthritis Joint Upper Limb	Nociceptive Pain
M7144	Calcium deposit in bursa, hand	Arthritis Joint Upper Limb	Nociceptive Pain
M71441	Calcium deposit in bursa, right hand	Arthritis Joint Upper Limb	Nociceptive Pain
M71442	Calcium deposit in bursa, left hand	Arthritis Joint Upper Limb	Nociceptive Pain
M71449	Calcium deposit in bursa, unspecified hand	Arthritis Joint Upper Limb	Nociceptive Pain
M7145	Calcium deposit in bursa, hip	Arthritis Joint Spine and Hips	Nociceptive Pain
M71451	Calcium deposit in bursa, right hip	Arthritis Joint Spine and Hips	Nociceptive Pain
M71452	Calcium deposit in bursa, left hip	Arthritis Joint Spine and Hips	Nociceptive Pain
M71459	Calcium deposit in bursa, unspecified hip	Arthritis Joint Spine and Hips	Nociceptive Pain
M7146	Calcium deposit in bursa, knee	Arthritis Joint Lower Limb	Nociceptive Pain
M71461	Calcium deposit in bursa, right knee	Arthritis Joint Lower Limb	Nociceptive Pain
M71462	Calcium deposit in bursa, left knee	Arthritis Joint Lower Limb	Nociceptive Pain
M71469	Calcium deposit in bursa, unspecified knee	Arthritis Joint Lower Limb	Nociceptive Pain
M7147	Calcium deposit in bursa, ankle and foot	Arthritis Joint Lower Limb	Nociceptive Pain
M71471	Calcium deposit in bursa, right ankle and foot	Arthritis Joint Lower Limb	Nociceptive Pain
M71472	Calcium deposit in bursa, left ankle and foot	Arthritis Joint Lower Limb	Nociceptive Pain
M71479	Calcium deposit in bursa, unspecified ankle and foot	Arthritis Joint Lower Limb	Nociceptive Pain
M7148	Calcium deposit in bursa, other site	Arthritis Joint Other	Nociceptive Pain
M7149	Calcium deposit in bursa, multiple sites	Arthritis Joint Other	Nociceptive Pain
M715	Other bursitis, not elsewhere classified	Arthritis Joint Other	Nociceptive Pain
M7150	Other bursitis, not elsewhere classified, unspecified site	Arthritis Joint Other	Nociceptive Pain
M7152	Other bursitis, not elsewhere classified, elbow	Arthritis Joint Upper Limb	Nociceptive Pain
M71521	Other bursitis, not elsewhere classified, right elbow	Arthritis Joint Upper Limb	Nociceptive Pain
M71522	Other bursitis, not elsewhere classified, left elbow	Arthritis Joint Upper Limb	Nociceptive Pain
M71529	Other bursitis, not elsewhere classified, unspecified elbow	Arthritis Joint Upper Limb	Nociceptive Pain

© 2024 Peterson MD et al. *JAMA Neurology.*

M7153	Other bursitis, not elsewhere classified, wrist	Arthritis Joint Upper Limb	Nociceptive Pain
M71531	Other bursitis, not elsewhere classified, right wrist	Arthritis Joint Upper Limb	Nociceptive Pain
M71532	Other bursitis, not elsewhere classified, left wrist	Arthritis Joint Upper Limb	Nociceptive Pain
M71539	Other bursitis, not elsewhere classified, unspecified wrist	Arthritis Joint Upper Limb	Nociceptive Pain
M7154	Other bursitis, not elsewhere classified, hand	Arthritis Joint Upper Limb	Nociceptive Pain
M71541	Other bursitis, not elsewhere classified, right hand	Arthritis Joint Upper Limb	Nociceptive Pain
M71542	Other bursitis, not elsewhere classified, left hand	Arthritis Joint Upper Limb	Nociceptive Pain
M71549	Other bursitis, not elsewhere classified, unspecified hand	Arthritis Joint Upper Limb	Nociceptive Pain
M7155	Other bursitis, not elsewhere classified, hip	Arthritis Joint Spine and Hips	Nociceptive Pain
M71551	Other bursitis, not elsewhere classified, right hip	Arthritis Joint Spine and Hips	Nociceptive Pain
M71552	Other bursitis, not elsewhere classified, left hip	Arthritis Joint Spine and Hips	Nociceptive Pain
M71559	Other bursitis, not elsewhere classified, unspecified hip	Arthritis Joint Spine and Hips	Nociceptive Pain
M7156	Other bursitis, not elsewhere classified, knee	Arthritis Joint Lower Limb	Nociceptive Pain
M71561	Other bursitis, not elsewhere classified, right knee	Arthritis Joint Lower Limb	Nociceptive Pain
M71562	Other bursitis, not elsewhere classified, left knee	Arthritis Joint Lower Limb	Nociceptive Pain
M71569	Other bursitis, not elsewhere classified, unspecified knee	Arthritis Joint Lower Limb	Nociceptive Pain
M7157	Other bursitis, not elsewhere classified, ankle and foot	Arthritis Joint Lower Limb	Nociceptive Pain
M71571	Oth bursitis, not elsewhere classified, right ankle and foot	Arthritis Joint Lower Limb	Nociceptive Pain
M71572	Oth bursitis, not elsewhere classified, left ankle and foot	Arthritis Joint Lower Limb	Nociceptive Pain
M71579	Oth bursitis, not elsewhere classified, unsp ankle and foot	Arthritis Joint Lower Limb	Nociceptive Pain
M7158	Other bursitis, not elsewhere classified, other site	Arthritis Joint Other	Nociceptive Pain
M718	Other specified bursopathies	Arthritis Joint Other	Nociceptive Pain
M7180	Other specified bursopathies, unspecified site	Arthritis Joint Other	Nociceptive Pain
M7181	Other specified bursopathies, shoulder	Arthritis Joint Upper Limb	Nociceptive Pain
M71811	Other specified bursopathies, right shoulder	Arthritis Joint Upper Limb	Nociceptive Pain
M71812	Other specified bursopathies, left shoulder	Arthritis Joint Upper Limb	Nociceptive Pain
M71819	Other specified bursopathies, unspecified shoulder	Arthritis Joint Upper Limb	Nociceptive Pain
M7182	Other specified bursopathies, elbow	Arthritis Joint Upper Limb	Nociceptive Pain
M71821	Other specified bursopathies, right elbow	Arthritis Joint Upper Limb	Nociceptive Pain
M71822	Other specified bursopathies, left elbow	Arthritis Joint Upper Limb	Nociceptive Pain
M71829	Other specified bursopathies, unspecified elbow	Arthritis Joint Upper Limb	Nociceptive Pain
M7183	Other specified bursopathies, wrist	Arthritis Joint Upper Limb	Nociceptive Pain
M71831	Other specified bursopathies, right wrist	Arthritis Joint Upper Limb	Nociceptive Pain
M71832	Other specified bursopathies, left wrist	Arthritis Joint Upper Limb	Nociceptive Pain
M71839	Other specified bursopathies, unspecified wrist	Arthritis Joint Upper Limb	Nociceptive Pain
M7184	Other specified bursopathies, hand	Arthritis Joint Upper Limb	Nociceptive Pain
M71841	Other specified bursopathies, right hand	Arthritis Joint Upper Limb	Nociceptive Pain

© 2024 Peterson MD et al. *JAMA Neurology.*

M71842	Other specified bursopathies, left hand	Arthritis Joint Upper Limb	Nociceptive Pain
M71849	Other specified bursopathies, unspecified hand	Arthritis Joint Upper Limb	Nociceptive Pain
M7185	Other specified bursopathies, hip	Arthritis Joint Spine and Hips	Nociceptive Pain
M71851	Other specified bursopathies, right hip	Arthritis Joint Spine and Hips	Nociceptive Pain
M71852	Other specified bursopathies, left hip	Arthritis Joint Spine and Hips	Nociceptive Pain
M71859	Other specified bursopathies, unspecified hip	Arthritis Joint Spine and Hips	Nociceptive Pain
M7186	Other specified bursopathies, knee	Arthritis Joint Lower Limb	Nociceptive Pain
M71861	Other specified bursopathies, right knee	Arthritis Joint Lower Limb	Nociceptive Pain
M71862	Other specified bursopathies, left knee	Arthritis Joint Lower Limb	Nociceptive Pain
M71869	Other specified bursopathies, unspecified knee	Arthritis Joint Lower Limb	Nociceptive Pain
M7187	Other specified bursopathies, ankle and foot	Arthritis Joint Lower Limb	Nociceptive Pain
M71871	Other specified bursopathies, right ankle and foot	Arthritis Joint Lower Limb	Nociceptive Pain
M71872	Other specified bursopathies, left ankle and foot	Arthritis Joint Lower Limb	Nociceptive Pain
M71879	Other specified bursopathies, unspecified ankle and foot	Arthritis Joint Lower Limb	Nociceptive Pain
M7188	Other specified bursopathies, other site	Arthritis Joint Other	Nociceptive Pain
M7189	Other specified bursopathies, multiple sites	Arthritis Joint Other	Nociceptive Pain
M719	Bursopathy, unspecified	Arthritis Joint Other	Nociceptive Pain
M72	Fibroblastic disorders	Arthritis Joint Other	Nociceptive Pain
M720	Palmar fascial fibromatosis [Dupuytren]	Arthritis Joint Lower Limb	Nociceptive Pain
M721	Knuckle pads	Arthritis Joint Upper Limb	Nociceptive Pain
M722	Plantar fascial fibromatosis	Arthritis Joint Lower Limb	Nociceptive Pain
M724	Pseudosarcomatous fibromatosis	Arthritis Joint Other	Nociceptive Pain
M726	Necrotizing fasciitis	Arthritis Joint Other	Nociceptive Pain
M728	Other fibroblastic disorders	Arthritis Joint Other	Nociceptive Pain
M729	Fibroblastic disorder, unspecified	Arthritis Joint Other	Nociceptive Pain
M75	Shoulder lesions	Arthritis Joint Upper Limb	Nociceptive Pain
M750	Adhesive capsulitis of shoulder	Arthritis Joint Upper Limb	Nociceptive Pain
M7500	Adhesive capsulitis of unspecified shoulder	Arthritis Joint Upper Limb	Nociceptive Pain
M7501	Adhesive capsulitis of right shoulder	Arthritis Joint Upper Limb	Nociceptive Pain
M7502	Adhesive capsulitis of left shoulder	Arthritis Joint Upper Limb	Nociceptive Pain
M751	Rotator cuff tear or rupture, not specified as traumatic	Arthritis Joint Upper Limb	Nociceptive Pain
M7510	Unsp rotatr-cuff tear/ruptr, not specified as traumatic	Arthritis Joint Upper Limb	Nociceptive Pain
M75100	Unsp rotatr-cuff tear/ruptr of unsp shoulder, not trauma	Arthritis Joint Upper Limb	Nociceptive Pain
M75101	Unsp rotatr-cuff tear/ruptr of right shoulder, not trauma	Arthritis Joint Upper Limb	Nociceptive Pain
M75102	Unsp rotatr-cuff tear/ruptr of left shoulder, not trauma	Arthritis Joint Upper Limb	Nociceptive Pain
M7511	Incomplete rotatr-cuff tear/ruptr not specified as traumatic	Arthritis Joint Upper Limb	Nociceptive Pain
M75110	Incmpl rotatr-cuff tear/ruptr of unsp shoulder, not trauma	Arthritis Joint Upper Limb	Nociceptive Pain

© 2024 Peterson MD et al. *JAMA Neurology.*

M75111	Incomplete rotatr-cuff tear/ruptr of r shoulder, not trauma	Arthritis Joint Upper Limb	Nociceptive Pain
M75112	Incomplete rotatr-cuff tear/ruptr of l shoulder, not trauma	Arthritis Joint Upper Limb	Nociceptive Pain
M7512	Complete rotatr-cuff tear/ruptr not specified as traumatic	Arthritis Joint Upper Limb	Nociceptive Pain
M75120	Complete rotatr-cuff tear/ruptr of unsp shoulder, not trauma	Arthritis Joint Upper Limb	Nociceptive Pain
M75121	Complete rotatr-cuff tear/ruptr of r shoulder, not trauma	Arthritis Joint Upper Limb	Nociceptive Pain
M75122	Complete rotatr-cuff tear/ruptr of left shoulder, not trauma	Arthritis Joint Upper Limb	Nociceptive Pain
M752	Bicipital tendinitis	Arthritis Joint Upper Limb	Nociceptive Pain
M7520	Bicipital tendinitis, unspecified shoulder	Arthritis Joint Upper Limb	Nociceptive Pain
M7521	Bicipital tendinitis, right shoulder	Arthritis Joint Upper Limb	Nociceptive Pain
M7522	Bicipital tendinitis, left shoulder	Arthritis Joint Upper Limb	Nociceptive Pain
M753	Calcific tendinitis of shoulder	Arthritis Joint Upper Limb	Nociceptive Pain
M7530	Calcific tendinitis of unspecified shoulder	Arthritis Joint Upper Limb	Nociceptive Pain
M7531	Calcific tendinitis of right shoulder	Arthritis Joint Upper Limb	Nociceptive Pain
M7532	Calcific tendinitis of left shoulder	Arthritis Joint Upper Limb	Nociceptive Pain
M754	Impingement syndrome of shoulder	Arthritis Joint Upper Limb	Nociceptive Pain
M7540	Impingement syndrome of unspecified shoulder	Arthritis Joint Upper Limb	Nociceptive Pain
M7541	Impingement syndrome of right shoulder	Arthritis Joint Upper Limb	Nociceptive Pain
M7542	Impingement syndrome of left shoulder	Arthritis Joint Upper Limb	Nociceptive Pain
M755	Bursitis of shoulder	Arthritis Joint Upper Limb	Nociceptive Pain
M7550	Bursitis of unspecified shoulder	Arthritis Joint Upper Limb	Nociceptive Pain
M7551	Bursitis of right shoulder	Arthritis Joint Upper Limb	Nociceptive Pain
M7552	Bursitis of left shoulder	Arthritis Joint Upper Limb	Nociceptive Pain
M758	Other shoulder lesions	Arthritis Joint Upper Limb	Nociceptive Pain
M7580	Other shoulder lesions, unspecified shoulder	Arthritis Joint Upper Limb	Nociceptive Pain
M7581	Other shoulder lesions, right shoulder	Arthritis Joint Upper Limb	Nociceptive Pain
M7582	Other shoulder lesions, left shoulder	Arthritis Joint Upper Limb	Nociceptive Pain
M759	Shoulder lesion, unspecified	Arthritis Joint Upper Limb	Nociceptive Pain
M7590	Shoulder lesion, unspecified, unspecified shoulder	Arthritis Joint Upper Limb	Nociceptive Pain
M7591	Shoulder lesion, unspecified, right shoulder	Arthritis Joint Upper Limb	Nociceptive Pain
M7592	Shoulder lesion, unspecified, left shoulder	Arthritis Joint Upper Limb	Nociceptive Pain
M76	Enthesopathies, lower limb, excluding foot	Arthritis Joint Lower Limb	Nociceptive Pain
M760	Gluteal tendinitis	Arthritis Joint Spine and Hips	Nociceptive Pain
M7600	Gluteal tendinitis, unspecified hip	Arthritis Joint Spine and Hips	Nociceptive Pain
M7601	Gluteal tendinitis, right hip	Arthritis Joint Spine and Hips	Nociceptive Pain
M7602	Gluteal tendinitis, left hip	Arthritis Joint Spine and Hips	Nociceptive Pain
M761	Psoas tendinitis	Arthritis Joint Spine and Hips	Nociceptive Pain
M7610	Psoas tendinitis, unspecified hip	Arthritis Joint Spine and Hips	Nociceptive Pain

© 2024 Peterson MD et al. *JAMA Neurology.*

M7611	Psoas tendinitis, right hip	Arthritis Joint Spine and Hips	Nociceptive Pain
M7612	Psoas tendinitis, left hip	Arthritis Joint Spine and Hips	Nociceptive Pain
M762	Iliac crest spur	Arthritis Joint Spine and Hips	Nociceptive Pain
M7620	Iliac crest spur, unspecified hip	Arthritis Joint Spine and Hips	Nociceptive Pain
M7621	Iliac crest spur, right hip	Arthritis Joint Spine and Hips	Nociceptive Pain
M7622	Iliac crest spur, left hip	Arthritis Joint Spine and Hips	Nociceptive Pain
M763	Iliotibial band syndrome	Arthritis Joint Lower Limb	Nociceptive Pain
M7630	Iliotibial band syndrome, unspecified leg	Arthritis Joint Lower Limb	Nociceptive Pain
M7631	Iliotibial band syndrome, right leg	Arthritis Joint Lower Limb	Nociceptive Pain
M7632	Iliotibial band syndrome, left leg	Arthritis Joint Lower Limb	Nociceptive Pain
M764	Tibial collateral bursitis [Pellegrini-Stieda]	Arthritis Joint Lower Limb	Nociceptive Pain
M7640	Tibial collateral bursitis, unspecified leg	Arthritis Joint Lower Limb	Nociceptive Pain
M7641	Tibial collateral bursitis [Pellegrini-Stieda], right leg	Arthritis Joint Lower Limb	Nociceptive Pain
M7642	Tibial collateral bursitis [Pellegrini-Stieda], left leg	Arthritis Joint Lower Limb	Nociceptive Pain
M765	Patellar tendinitis	Arthritis Joint Lower Limb	Nociceptive Pain
M7650	Patellar tendinitis, unspecified knee	Arthritis Joint Lower Limb	Nociceptive Pain
M7651	Patellar tendinitis, right knee	Arthritis Joint Lower Limb	Nociceptive Pain
M7652	Patellar tendinitis, left knee	Arthritis Joint Lower Limb	Nociceptive Pain
M766	Achilles tendinitis	Arthritis Joint Lower Limb	Nociceptive Pain
M7660	Achilles tendinitis, unspecified leg	Arthritis Joint Lower Limb	Nociceptive Pain
M7661	Achilles tendinitis, right leg	Arthritis Joint Lower Limb	Nociceptive Pain
M7662	Achilles tendinitis, left leg	Arthritis Joint Lower Limb	Nociceptive Pain
M767	Peroneal tendinitis	Arthritis Joint Lower Limb	Nociceptive Pain
M7670	Peroneal tendinitis, unspecified leg	Arthritis Joint Lower Limb	Nociceptive Pain
M7671	Peroneal tendinitis, right leg	Arthritis Joint Lower Limb	Nociceptive Pain
M7672	Peroneal tendinitis, left leg	Arthritis Joint Lower Limb	Nociceptive Pain
M768	Other specified enthesopathies of lower limb, excluding foot	Arthritis Joint Lower Limb	Nociceptive Pain
M7681	Anterior tibial syndrome	Arthritis Joint Lower Limb	Nociceptive Pain
M76811	Anterior tibial syndrome, right leg	Arthritis Joint Lower Limb	Nociceptive Pain
M76812	Anterior tibial syndrome, left leg	Arthritis Joint Lower Limb	Nociceptive Pain
M76819	Anterior tibial syndrome, unspecified leg	Arthritis Joint Lower Limb	Nociceptive Pain
M7682	Posterior tibial tendinitis	Arthritis Joint Lower Limb	Nociceptive Pain
M76821	Posterior tibial tendinitis, right leg	Arthritis Joint Lower Limb	Nociceptive Pain
M76822	Posterior tibial tendinitis, left leg	Arthritis Joint Lower Limb	Nociceptive Pain
M76829	Posterior tibial tendinitis, unspecified leg	Arthritis Joint Lower Limb	Nociceptive Pain
M7689	Other specified enthesopathies of lower limb, excluding foot	Arthritis Joint Lower Limb	Nociceptive Pain
M76891	Oth enthesopathies of right lower limb, excluding foot	Arthritis Joint Lower Limb	Nociceptive Pain

© 2024 Peterson MD et al. *JAMA Neurology.*

M76892	Oth enthesopathies of left lower limb, excluding foot	Arthritis Joint Lower Limb	Nociceptive Pain
M76899	Oth enthesopathies of unspecified lower limb, excluding foot	Arthritis Joint Lower Limb	Nociceptive Pain
M769	Unspecified enthesopathy, lower limb, excluding foot	Arthritis Joint Lower Limb	Nociceptive Pain
M77	Other enthesopathies	Arthritis Joint Other	Nociceptive Pain
M770	Medial epicondylitis	Arthritis Joint Upper Limb	Nociceptive Pain
M7700	Medial epicondylitis, unspecified elbow	Arthritis Joint Upper Limb	Nociceptive Pain
M7701	Medial epicondylitis, right elbow	Arthritis Joint Upper Limb	Nociceptive Pain
M7702	Medial epicondylitis, left elbow	Arthritis Joint Upper Limb	Nociceptive Pain
M771	Lateral epicondylitis	Arthritis Joint Upper Limb	Nociceptive Pain
M7710	Lateral epicondylitis, unspecified elbow	Arthritis Joint Upper Limb	Nociceptive Pain
M7711	Lateral epicondylitis, right elbow	Arthritis Joint Upper Limb	Nociceptive Pain
M7712	Lateral epicondylitis, left elbow	Arthritis Joint Upper Limb	Nociceptive Pain
M772	Periarthritis of wrist	Arthritis Joint Upper Limb	Nociceptive Pain
M7720	Periarthritis, unspecified wrist	Arthritis Joint Upper Limb	Nociceptive Pain
M7721	Periarthritis, right wrist	Arthritis Joint Upper Limb	Nociceptive Pain
M7722	Periarthritis, left wrist	Arthritis Joint Upper Limb	Nociceptive Pain
M773	Calcaneal spur	Arthritis Joint Lower Limb	Nociceptive Pain
M7730	Calcaneal spur, unspecified foot	Arthritis Joint Lower Limb	Nociceptive Pain
M7731	Calcaneal spur, right foot	Arthritis Joint Lower Limb	Nociceptive Pain
M7732	Calcaneal spur, left foot	Arthritis Joint Lower Limb	Nociceptive Pain
M774	Metatarsalgia	Arthritis Joint Lower Limb	Nociceptive Pain
M7740	Metatarsalgia, unspecified foot	Arthritis Joint Lower Limb	Nociceptive Pain
M7741	Metatarsalgia, right foot	Arthritis Joint Lower Limb	Nociceptive Pain
M7742	Metatarsalgia, left foot	Arthritis Joint Lower Limb	Nociceptive Pain
M775	Other enthesopathy of foot and ankle	Arthritis Joint Lower Limb	Nociceptive Pain
M7750	Other enthesopathy of unspecified foot and ankle	Arthritis Joint Lower Limb	Nociceptive Pain
M7751	Other enthesopathy of right foot and ankle	Arthritis Joint Lower Limb	Nociceptive Pain
M7752	Other enthesopathy of left foot and ankle	Arthritis Joint Lower Limb	Nociceptive Pain
M778	Other enthesopathies, not elsewhere classified	Arthritis Joint Other	Nociceptive Pain
M779	Enthesopathy, unspecified	Arthritis Joint Other	Nociceptive Pain
M796	Pain in limb, hand, foot, fingers and toes	Arthritis Joint Other	Nociceptive Pain
M80	Osteoporosis with current pathological fracture	Arthritis Joint Other	Nociceptive Pain
M800	Age-related osteoporosis with current pathological fracture	Arthritis Joint Other	Nociceptive Pain
M8000	Age-related osteopor w current path fracture, unsp site	Arthritis Joint Other	Nociceptive Pain
M8000XA	Age-rel osteopor w current path fracture, unsp site, init	Arthritis Joint Other	Nociceptive Pain
M8000XD	Age-rel osteopor w crnt path fx, unsp site, 7thD	Arthritis Joint Other	Nociceptive Pain
M8000XG	Age-rel osteopor w crnt path fx, unsp site, 7thG	Arthritis Joint Other	Nociceptive Pain

© 2024 Peterson MD et al. *JAMA Neurology.*

M8000XK	Age-rel osteopor w crnt path fx, unsp site, 7thK	Arthritis Joint Other	Nociceptive Pain
M8000XP	Age-rel osteopor w crnt path fx, unsp site, 7thP	Arthritis Joint Other	Nociceptive Pain
M8000XS	Age-rel osteopor w current path fracture, unsp site, sequela	Arthritis Joint Other	Nociceptive Pain
M8001	Age-related osteopor w current path fracture, shoulder	Arthritis Joint Upper Limb	Nociceptive Pain
M80011	Age-related osteopor w current path fracture, r shoulder	Arthritis Joint Upper Limb	Nociceptive Pain
M80011A	Age-rel osteopor w current path fracture, r shoulder, init	Arthritis Joint Upper Limb	Nociceptive Pain
M80011D	Age-rel osteopor w crnt path fx, r shldr, 7thD	Arthritis Joint Upper Limb	Nociceptive Pain
M80011G	Age-rel osteopor w crnt path fx, r shldr, 7thG	Arthritis Joint Upper Limb	Nociceptive Pain
M80011K	Age-rel osteopor w crnt path fx, r shldr, 7thK	Arthritis Joint Upper Limb	Nociceptive Pain
M80011P	Age-rel osteopor w crnt path fx, r shldr, 7thP	Arthritis Joint Upper Limb	Nociceptive Pain
M80011S	Age-rel osteopor w current path fx, r shoulder, sequela	Arthritis Joint Upper Limb	Nociceptive Pain
M80012	Age-related osteopor w current path fracture, l shoulder	Arthritis Joint Upper Limb	Nociceptive Pain
M80012A	Age-rel osteopor w current path fracture, l shoulder, init	Arthritis Joint Upper Limb	Nociceptive Pain
M80012D	Age-rel osteopor w crnt path fx, l shldr, 7thD	Arthritis Joint Upper Limb	Nociceptive Pain
M80012G	Age-rel osteopor w crnt path fx, l shldr, 7thG	Arthritis Joint Upper Limb	Nociceptive Pain
M80012K	Age-rel osteopor w crnt path fx, l shldr, 7thK	Arthritis Joint Upper Limb	Nociceptive Pain
M80012P	Age-rel osteopor w crnt path fx, l shldr, 7thP	Arthritis Joint Upper Limb	Nociceptive Pain
M80012S	Age-rel osteopor w current path fx, l shoulder, sequela	Arthritis Joint Upper Limb	Nociceptive Pain
M80019	Age-related osteopor w current path fracture, unsp shoulder	Arthritis Joint Upper Limb	Nociceptive Pain
M80019A	Age-rel osteopor w current path fx, unsp shoulder, init	Arthritis Joint Upper Limb	Nociceptive Pain
M80019D	Age-rel osteopor w crnt path fx, unsp shldr, 7thD	Arthritis Joint Upper Limb	Nociceptive Pain
M80019G	Age-rel osteopor w crnt path fx, unsp shldr, 7thG	Arthritis Joint Upper Limb	Nociceptive Pain
M80019K	Age-rel osteopor w crnt path fx, unsp shldr, 7thK	Arthritis Joint Upper Limb	Nociceptive Pain
M80019P	Age-rel osteopor w crnt path fx, unsp shldr, 7thP	Arthritis Joint Upper Limb	Nociceptive Pain
M80019S	Age-rel osteopor w current path fx, unsp shoulder, sequela	Arthritis Joint Upper Limb	Nociceptive Pain
M8002	Age-related osteopor w current path fracture, humerus	Arthritis Joint Upper Limb	Nociceptive Pain
M80021	Age-related osteopor w current path fracture, r humerus	Arthritis Joint Upper Limb	Nociceptive Pain
M80021A	Age-rel osteopor w current path fracture, r humerus, init	Arthritis Joint Upper Limb	Nociceptive Pain
M80021D	Age-rel osteopor w crnt path fx, r humer, 7thD	Arthritis Joint Upper Limb	Nociceptive Pain
M80021G	Age-rel osteopor w crnt path fx, r humer, 7thG	Arthritis Joint Upper Limb	Nociceptive Pain
M80021K	Age-rel osteopor w crnt path fx, r humer, 7thK	Arthritis Joint Upper Limb	Nociceptive Pain
M80021P	Age-rel osteopor w crnt path fx, r humer, 7thP	Arthritis Joint Upper Limb	Nociceptive Pain
M80021S	Age-rel osteopor w current path fracture, r humerus, sequela	Arthritis Joint Upper Limb	Nociceptive Pain
M80022	Age-related osteopor w current path fracture, l humerus	Arthritis Joint Upper Limb	Nociceptive Pain
M80022A	Age-rel osteopor w current path fracture, l humerus, init	Arthritis Joint Upper Limb	Nociceptive Pain
M80022D	Age-rel osteopor w crnt path fx, l humer, 7thD	Arthritis Joint Upper Limb	Nociceptive Pain
M80022G	Age-rel osteopor w crnt path fx, l humer, 7thG	Arthritis Joint Upper Limb	Nociceptive Pain

© 2024 Peterson MD et al. *JAMA Neurology.*

M80022K	Age-rel osteopor w crnt path fx, l humer, 7thK	Arthritis Joint Upper Limb	Nociceptive Pain
M80022P	Age-rel osteopor w crnt path fx, l humer, 7thP	Arthritis Joint Upper Limb	Nociceptive Pain
M80022S	Age-rel osteopor w current path fracture, l humerus, sequela	Arthritis Joint Upper Limb	Nociceptive Pain
M80029	Age-related osteopor w current path fracture, unsp humerus	Arthritis Joint Upper Limb	Nociceptive Pain
M80029A	Age-rel osteopor w current path fracture, unsp humerus, init	Arthritis Joint Upper Limb	Nociceptive Pain
M80029D	Age-rel osteopor w crnt path fx, unsp humer, 7thD	Arthritis Joint Upper Limb	Nociceptive Pain
M80029G	Age-rel osteopor w crnt path fx, unsp humer, 7thG	Arthritis Joint Upper Limb	Nociceptive Pain
M80029K	Age-rel osteopor w crnt path fx, unsp humer, 7thK	Arthritis Joint Upper Limb	Nociceptive Pain
M80029P	Age-rel osteopor w crnt path fx, unsp humer, 7thP	Arthritis Joint Upper Limb	Nociceptive Pain
M80029S	Age-rel osteopor w current path fx, unsp humerus, sequela	Arthritis Joint Upper Limb	Nociceptive Pain
M8003	Age-related osteopor w current path fracture, forearm	Arthritis Joint Upper Limb	Nociceptive Pain
M80031	Age-related osteopor w current path fracture, r forearm	Arthritis Joint Upper Limb	Nociceptive Pain
M80031A	Age-rel osteopor w current path fracture, r forearm, init	Arthritis Joint Upper Limb	Nociceptive Pain
M80031D	Age-rel osteopor w crnt path fx, r forearm, 7thD	Arthritis Joint Upper Limb	Nociceptive Pain
M80031G	Age-rel osteopor w crnt path fx, r forearm, 7thG	Arthritis Joint Upper Limb	Nociceptive Pain
M80031K	Age-rel osteopor w crnt path fx, r forearm, 7thK	Arthritis Joint Upper Limb	Nociceptive Pain
M80031P	Age-rel osteopor w crnt path fx, r forearm, 7thP	Arthritis Joint Upper Limb	Nociceptive Pain
M80031S	Age-rel osteopor w current path fracture, r forearm, sequela	Arthritis Joint Upper Limb	Nociceptive Pain
M80032	Age-related osteopor w current path fracture, l forearm	Arthritis Joint Upper Limb	Nociceptive Pain
M80032A	Age-rel osteopor w current path fracture, l forearm, init	Arthritis Joint Upper Limb	Nociceptive Pain
M80032D	Age-rel osteopor w crnt path fx, l forearm, 7thD	Arthritis Joint Upper Limb	Nociceptive Pain
M80032G	Age-rel osteopor w crnt path fx, l forearm, 7thG	Arthritis Joint Upper Limb	Nociceptive Pain
M80032K	Age-rel osteopor w crnt path fx, l forearm, 7thK	Arthritis Joint Upper Limb	Nociceptive Pain
M80032P	Age-rel osteopor w crnt path fx, l forearm, 7thP	Arthritis Joint Upper Limb	Nociceptive Pain
M80032S	Age-rel osteopor w current path fracture, l forearm, sequela	Arthritis Joint Upper Limb	Nociceptive Pain
M80039	Age-related osteopor w current path fracture, unsp forearm	Arthritis Joint Upper Limb	Nociceptive Pain
M80039A	Age-rel osteopor w current path fracture, unsp forearm, init	Arthritis Joint Upper Limb	Nociceptive Pain
M80039D	Age-rel osteopor w crnt path fx, unsp forearm, 7thD	Arthritis Joint Upper Limb	Nociceptive Pain
M80039G	Age-rel osteopor w crnt path fx, unsp forearm, 7thG	Arthritis Joint Upper Limb	Nociceptive Pain
M80039K	Age-rel osteopor w crnt path fx, unsp forearm, 7thK	Arthritis Joint Upper Limb	Nociceptive Pain
M80039P	Age-rel osteopor w crnt path fx, unsp forearm, 7thP	Arthritis Joint Upper Limb	Nociceptive Pain
M80039S	Age-rel osteopor w current path fx, unsp forearm, sequela	Arthritis Joint Upper Limb	Nociceptive Pain
M8004	Age-related osteopor w current pathological fracture, hand	Arthritis Joint Upper Limb	Nociceptive Pain
M80041	Age-related osteopor w current path fracture, right hand	Arthritis Joint Upper Limb	Nociceptive Pain
M80041A	Age-rel osteopor w current path fracture, right hand, init	Arthritis Joint Upper Limb	Nociceptive Pain
M80041D	Age-rel osteopor w crnt path fx, r hand, 7thD	Arthritis Joint Upper Limb	Nociceptive Pain
M80041G	Age-rel osteopor w crnt path fx, r hand, 7thG	Arthritis Joint Upper Limb	Nociceptive Pain

© 2024 Peterson MD et al. *JAMA Neurology.*

M80041K	Age-rel osteopor w crnt path fx, r hand, 7thK	Arthritis Joint Upper Limb	Nociceptive Pain
M80041P	Age-rel osteopor w crnt path fx, r hand, 7thP	Arthritis Joint Upper Limb	Nociceptive Pain
M80041S	Age-rel osteopor w current path fracture, r hand, sequela	Arthritis Joint Upper Limb	Nociceptive Pain
M80042	Age-related osteopor w current path fracture, left hand	Arthritis Joint Upper Limb	Nociceptive Pain
M80042A	Age-rel osteopor w current path fracture, left hand, init	Arthritis Joint Upper Limb	Nociceptive Pain
M80042D	Age-rel osteopor w crnt path fx, l hand, 7thD	Arthritis Joint Upper Limb	Nociceptive Pain
M80042G	Age-rel osteopor w crnt path fx, l hand, 7thG	Arthritis Joint Upper Limb	Nociceptive Pain
M80042K	Age-rel osteopor w crnt path fx, l hand, 7thK	Arthritis Joint Upper Limb	Nociceptive Pain
M80042P	Age-rel osteopor w crnt path fx, l hand, 7thP	Arthritis Joint Upper Limb	Nociceptive Pain
M80042S	Age-rel osteopor w current path fracture, left hand, sequela	Arthritis Joint Upper Limb	Nociceptive Pain
M80049	Age-related osteopor w current path fracture, unsp hand	Arthritis Joint Upper Limb	Nociceptive Pain
M80049A	Age-rel osteopor w current path fracture, unsp hand, init	Arthritis Joint Upper Limb	Nociceptive Pain
M80049D	Age-rel osteopor w crnt path fx, unsp hand, 7thD	Arthritis Joint Upper Limb	Nociceptive Pain
M80049G	Age-rel osteopor w crnt path fx, unsp hand, 7thG	Arthritis Joint Upper Limb	Nociceptive Pain
M80049K	Age-rel osteopor w crnt path fx, unsp hand, 7thK	Arthritis Joint Upper Limb	Nociceptive Pain
M80049P	Age-rel osteopor w crnt path fx, unsp hand, 7thP	Arthritis Joint Upper Limb	Nociceptive Pain
M80049S	Age-rel osteopor w current path fracture, unsp hand, sequela	Arthritis Joint Upper Limb	Nociceptive Pain
M8005	Age-related osteopor w current pathological fracture, femur	Arthritis Joint Lower Limb	Nociceptive Pain
M80051	Age-related osteopor w current path fracture, right femur	Arthritis Joint Lower Limb	Nociceptive Pain
M80051A	Age-rel osteopor w current path fracture, right femur, init	Arthritis Joint Lower Limb	Nociceptive Pain
M80051D	Age-rel osteopor w crnt path fx, r femr, 7thD	Arthritis Joint Lower Limb	Nociceptive Pain
M80051G	Age-rel osteopor w crnt path fx, r femr, 7thG	Arthritis Joint Lower Limb	Nociceptive Pain
M80051K	Age-rel osteopor w crnt path fx, r femr, 7thK	Arthritis Joint Lower Limb	Nociceptive Pain
M80051P	Age-rel osteopor w crnt path fx, r femr, 7thP	Arthritis Joint Lower Limb	Nociceptive Pain
M80051S	Age-rel osteopor w current path fracture, r femur, sequela	Arthritis Joint Lower Limb	Nociceptive Pain
M80052	Age-related osteopor w current path fracture, left femur	Arthritis Joint Lower Limb	Nociceptive Pain
M80052A	Age-rel osteopor w current path fracture, left femur, init	Arthritis Joint Lower Limb	Nociceptive Pain
M80052D	Age-rel osteopor w crnt path fx, l femr, 7thD	Arthritis Joint Lower Limb	Nociceptive Pain
M80052G	Age-rel osteopor w crnt path fx, l femr, 7thG	Arthritis Joint Lower Limb	Nociceptive Pain
M80052K	Age-rel osteopor w crnt path fx, l femr, 7thK	Arthritis Joint Lower Limb	Nociceptive Pain
M80052P	Age-rel osteopor w crnt path fx, l femr, 7thP	Arthritis Joint Lower Limb	Nociceptive Pain
M80052S	Age-rel osteopor w current path fracture, l femur, sequela	Arthritis Joint Lower Limb	Nociceptive Pain
M80059	Age-related osteopor w current path fracture, unsp femur	Arthritis Joint Lower Limb	Nociceptive Pain
M80059A	Age-rel osteopor w current path fracture, unsp femur, init	Arthritis Joint Lower Limb	Nociceptive Pain
M80059D	Age-rel osteopor w crnt path fx, unsp femr, 7thD	Arthritis Joint Lower Limb	Nociceptive Pain
M80059G	Age-rel osteopor w crnt path fx, unsp femr, 7thG	Arthritis Joint Lower Limb	Nociceptive Pain
M80059K	Age-rel osteopor w crnt path fx, unsp femr, 7thK	Arthritis Joint Lower Limb	Nociceptive Pain

© 2024 Peterson MD et al. *JAMA Neurology.*

M80059P	Age-rel osteopor w crnt path fx, unsp femr, 7thP	Arthritis Joint Lower Limb	Nociceptive Pain
M80059S	Age-rel osteopor w current path fx, unsp femur, sequela	Arthritis Joint Lower Limb	Nociceptive Pain
M8006	Age-related osteopor w current path fracture, lower leg	Arthritis Joint Lower Limb	Nociceptive Pain
M80061	Age-related osteopor w current path fracture, r low leg	Arthritis Joint Lower Limb	Nociceptive Pain
M80061A	Age-rel osteopor w current path fracture, r low leg, init	Arthritis Joint Lower Limb	Nociceptive Pain
M80061D	Age-rel osteopor w crnt path fx, r low leg, 7thD	Arthritis Joint Lower Limb	Nociceptive Pain
M80061G	Age-rel osteopor w crnt path fx, r low leg, 7thG	Arthritis Joint Lower Limb	Nociceptive Pain
M80061K	Age-rel osteopor w crnt path fx, r low leg, 7thK	Arthritis Joint Lower Limb	Nociceptive Pain
M80061P	Age-rel osteopor w crnt path fx, r low leg, 7thP	Arthritis Joint Lower Limb	Nociceptive Pain
M80061S	Age-rel osteopor w current path fracture, r low leg, sequela	Arthritis Joint Lower Limb	Nociceptive Pain
M80062	Age-related osteopor w current path fracture, l low leg	Arthritis Joint Lower Limb	Nociceptive Pain
M80062A	Age-rel osteopor w current path fracture, l low leg, init	Arthritis Joint Lower Limb	Nociceptive Pain
M80062D	Age-rel osteopor w crnt path fx, l low leg, 7thD	Arthritis Joint Lower Limb	Nociceptive Pain
M80062G	Age-rel osteopor w crnt path fx, l low leg, 7thG	Arthritis Joint Lower Limb	Nociceptive Pain
M80062K	Age-rel osteopor w crnt path fx, l low leg, 7thK	Arthritis Joint Lower Limb	Nociceptive Pain
M80062P	Age-rel osteopor w crnt path fx, l low leg, 7thP	Arthritis Joint Lower Limb	Nociceptive Pain
M80062S	Age-rel osteopor w current path fracture, l low leg, sequela	Arthritis Joint Lower Limb	Nociceptive Pain
M80069	Age-related osteopor w current path fracture, unsp lower leg	Arthritis Joint Lower Limb	Nociceptive Pain
M80069A	Age-rel osteopor w current path fracture, unsp low leg, init	Arthritis Joint Lower Limb	Nociceptive Pain
M80069D	Age-rel osteopor w crnt path fx, unsp low leg, 7thD	Arthritis Joint Lower Limb	Nociceptive Pain
M80069G	Age-rel osteopor w crnt path fx, unsp low leg, 7thG	Arthritis Joint Lower Limb	Nociceptive Pain
M80069K	Age-rel osteopor w crnt path fx, unsp low leg, 7thK	Arthritis Joint Lower Limb	Nociceptive Pain
M80069P	Age-rel osteopor w crnt path fx, unsp low leg, 7thP	Arthritis Joint Lower Limb	Nociceptive Pain
M80069S	Age-rel osteopor w current path fx, unsp low leg, sequela	Arthritis Joint Lower Limb	Nociceptive Pain
M8007	Age-related osteopor w current pathological fracture, ank/ft	Arthritis Joint Lower Limb	Nociceptive Pain
M80071	Age-related osteopor w current path fracture, right ank/ft	Arthritis Joint Lower Limb	Nociceptive Pain
M80071A	Age-rel osteopor w current path fracture, right ank/ft, init	Arthritis Joint Lower Limb	Nociceptive Pain
M80071D	Age-rel osteopor w crnt path fx, r ank/ft, 7thD	Arthritis Joint Lower Limb	Nociceptive Pain
M80071G	Age-rel osteopor w crnt path fx, r ank/ft, 7thG	Arthritis Joint Lower Limb	Nociceptive Pain
M80071K	Age-rel osteopor w crnt path fx, r ank/ft, 7thK	Arthritis Joint Lower Limb	Nociceptive Pain
M80071P	Age-rel osteopor w crnt path fx, r ank/ft, 7thP	Arthritis Joint Lower Limb	Nociceptive Pain
M80071S	Age-rel osteopor w current path fx, right ank/ft, sequela	Arthritis Joint Lower Limb	Nociceptive Pain
M80072	Age-related osteopor w current path fracture, left ank/ft	Arthritis Joint Lower Limb	Nociceptive Pain
M80072A	Age-rel osteopor w current path fracture, left ank/ft, init	Arthritis Joint Lower Limb	Nociceptive Pain
M80072D	Age-rel osteopor w crnt path fx, l ank/ft, 7thD	Arthritis Joint Lower Limb	Nociceptive Pain
M80072G	Age-rel osteopor w crnt path fx, l ank/ft, 7thG	Arthritis Joint Lower Limb	Nociceptive Pain
M80072K	Age-rel osteopor w crnt path fx, l ank/ft, 7thK	Arthritis Joint Lower Limb	Nociceptive Pain

© 2024 Peterson MD et al. *JAMA Neurology.*

M80072P	Age-rel osteopor w crnt path fx, l ank/ft, 7thP	Arthritis Joint Lower Limb	Nociceptive Pain
M80072S	Age-rel osteopor w current path fx, left ank/ft, sequela	Arthritis Joint Lower Limb	Nociceptive Pain
M80079	Age-related osteopor w current path fracture, unsp ank/ft	Arthritis Joint Lower Limb	Nociceptive Pain
M80079A	Age-rel osteopor w current path fracture, unsp ank/ft, init	Arthritis Joint Lower Limb	Nociceptive Pain
M80079D	Age-rel osteopor w crnt path fx, unsp ank/ft, 7thD	Arthritis Joint Lower Limb	Nociceptive Pain
M80079G	Age-rel osteopor w crnt path fx, unsp ank/ft, 7thG	Arthritis Joint Lower Limb	Nociceptive Pain
M80079K	Age-rel osteopor w crnt path fx, unsp ank/ft, 7thK	Arthritis Joint Lower Limb	Nociceptive Pain
M80079P	Age-rel osteopor w crnt path fx, unsp ank/ft, 7thP	Arthritis Joint Lower Limb	Nociceptive Pain
M80079S	Age-rel osteopor w current path fx, unsp ank/ft, sequela	Arthritis Joint Lower Limb	Nociceptive Pain
M8008	Age-related osteopor w current path fracture, vertebra(e)	Arthritis Joint Spine and Hips	Nociceptive Pain
M8008XA	Age-rel osteopor w current path fracture, vertebra(e), init	Arthritis Joint Spine and Hips	Nociceptive Pain
M8008XD	Age-rel osteopor w crnt path fx, verteb, 7thD	Arthritis Joint Spine and Hips	Nociceptive Pain
M8008XG	Age-rel osteopor w crnt path fx, verteb, 7thG	Arthritis Joint Spine and Hips	Nociceptive Pain
M8008XK	Age-rel osteopor w crnt path fx, verteb, 7thK	Arthritis Joint Spine and Hips	Nociceptive Pain
M8008XP	Age-rel osteopor w crnt path fx, verteb, 7thP	Arthritis Joint Spine and Hips	Nociceptive Pain
M8008XS	Age-rel osteopor w current path fracture, verteb, sequela	Arthritis Joint Spine and Hips	Nociceptive Pain
M800A	Age-related osteopor with current path fracture, other site	Arthritis Joint Other	Nociceptive Pain
M800AXA	Age-rel osteopor with current path fx, other site, init	Arthritis Joint Other	Nociceptive Pain
M800AXD	Age-rel osteopor with current path fx, other site, 7thD	Arthritis Joint Other	Nociceptive Pain
M800AXG	Age-rel osteopor with current path fx, other site, 7thG	Arthritis Joint Other	Nociceptive Pain
M800AXK	Age-rel osteopor with current path fx, other site, 7thK	Arthritis Joint Other	Nociceptive Pain
M800AXP	Age-rel osteopor with current path fx, other site, 7thP	Arthritis Joint Other	Nociceptive Pain
M800AXS	Age-rel osteopor with current path fx, other site, sequela	Arthritis Joint Other	Nociceptive Pain
M800B	Age-related osteopor with current path fracture, pelvis	Arthritis Joint Spine and Hips	Nociceptive Pain
M800B1	Age-rel osteopor with current path fracture, right pelvis	Arthritis Joint Spine and Hips	Nociceptive Pain
M800B1A	Age-rel osteopor with current path fx, right pelvis, init	Arthritis Joint Spine and Hips	Nociceptive Pain
M800B1D	Age-rel osteopor with current path fx, right pelvis, 7thD	Arthritis Joint Spine and Hips	Nociceptive Pain
M800B1G	Age-rel osteopor with current path fx, right pelvis, 7thG	Arthritis Joint Spine and Hips	Nociceptive Pain
M800B1K	Age-rel osteopor with current path fx, right pelvis, 7thK	Arthritis Joint Spine and Hips	Nociceptive Pain
M800B1P	Age-rel osteopor with current path fx, right pelvis, 7thP	Arthritis Joint Spine and Hips	Nociceptive Pain
M800B1S	Age-rel osteopor with current path fx, right pelvis, sequela	Arthritis Joint Spine and Hips	Nociceptive Pain
M800B2	Age-related osteopor with current path fracture, left pelvis	Arthritis Joint Spine and Hips	Nociceptive Pain
M800B2A	Age-rel osteopor with current path fx, left pelvis, init	Arthritis Joint Spine and Hips	Nociceptive Pain
M800B2D	Age-rel osteopor with current path fx, left pelvis, 7thD	Arthritis Joint Spine and Hips	Nociceptive Pain
M800B2G	Age-rel osteopor with current path fx, left pelvis, 7thG	Arthritis Joint Spine and Hips	Nociceptive Pain
M800B2K	Age-rel osteopor with current path fx, left pelvis, 7thK	Arthritis Joint Spine and Hips	Nociceptive Pain
M800B2P	Age-rel osteopor with current path fx, left pelvis, 7thP	Arthritis Joint Spine and Hips	Nociceptive Pain

© 2024 Peterson MD et al. *JAMA Neurology.*

M800B2S	Age-rel osteopor with current path fx, left pelvis, sequela	Arthritis Joint Spine and Hips	Nociceptive Pain
M800B9	Age-rel osteopor with current path fracture, unsp pelvis	Arthritis Joint Spine and Hips	Nociceptive Pain
M800B9A	Age-rel osteopor with current path fx, unsp pelvis, init	Arthritis Joint Spine and Hips	Nociceptive Pain
M800B9D	Age-rel osteopor with current path fx, unsp pelvis, 7thD	Arthritis Joint Spine and Hips	Nociceptive Pain
M800B9G	Age-rel osteopor with current path fx, unsp pelvis, 7thG	Arthritis Joint Spine and Hips	Nociceptive Pain
M800B9K	Age-rel osteopor with current path fx, unsp pelvis, 7thK	Arthritis Joint Spine and Hips	Nociceptive Pain
M800B9P	Age-rel osteopor with current path fx, unsp pelvis, 7thP	Arthritis Joint Spine and Hips	Nociceptive Pain
M800B9S	Age-rel osteopor with current path fx, unsp pelvis, sequela	Arthritis Joint Spine and Hips	Nociceptive Pain
M808	Other osteoporosis with current pathological fracture	Arthritis Joint Other	Nociceptive Pain
M8080	Oth osteoporosis w current pathological fracture, unsp site	Arthritis Joint Other	Nociceptive Pain
M8080XA	Oth osteopor w current path fracture, unsp site, init	Arthritis Joint Other	Nociceptive Pain
M8080XD	Oth osteopor w crnt path fx, unsp site, 7thD	Arthritis Joint Other	Nociceptive Pain
M8080XG	Oth osteopor w crnt path fx, unsp site, 7thG	Arthritis Joint Other	Nociceptive Pain
M8080XK	Oth osteopor w crnt path fx, unsp site, 7thK	Arthritis Joint Other	Nociceptive Pain
M8080XP	Oth osteopor w crnt path fx, unsp site, 7thP	Arthritis Joint Other	Nociceptive Pain
M8080XS	Oth osteopor w current path fracture, unsp site, sequela	Arthritis Joint Other	Nociceptive Pain
M8081	Other osteoporosis with pathological fracture, shoulder	Arthritis Joint Upper Limb	Nociceptive Pain
M80811	Oth osteoporosis w current pathological fracture, r shoulder	Arthritis Joint Upper Limb	Nociceptive Pain
M80811A	Oth osteopor w current path fracture, r shoulder, init	Arthritis Joint Upper Limb	Nociceptive Pain
M80811D	Oth osteopor w crnt path fx, r shldr, 7thD	Arthritis Joint Upper Limb	Nociceptive Pain
M80811G	Oth osteopor w crnt path fx, r shldr, 7thG	Arthritis Joint Upper Limb	Nociceptive Pain
M80811K	Oth osteopor w crnt path fx, r shldr, subs for fx w nonunion	Arthritis Joint Upper Limb	Nociceptive Pain
M80811P	Oth osteopor w crnt path fx, r shldr, subs for fx w malunion	Arthritis Joint Upper Limb	Nociceptive Pain
M80811S	Oth osteopor w current path fracture, r shoulder, sequela	Arthritis Joint Upper Limb	Nociceptive Pain
M80812	Oth osteoporosis w current pathological fracture, l shoulder	Arthritis Joint Upper Limb	Nociceptive Pain
M80812A	Oth osteopor w current path fracture, l shoulder, init	Arthritis Joint Upper Limb	Nociceptive Pain
M80812D	Oth osteopor w crnt path fx, l shldr, 7thD	Arthritis Joint Upper Limb	Nociceptive Pain
M80812G	Oth osteopor w crnt path fx, l shldr, 7thG	Arthritis Joint Upper Limb	Nociceptive Pain
M80812K	Oth osteopor w crnt path fx, l shldr, subs for fx w nonunion	Arthritis Joint Upper Limb	Nociceptive Pain
M80812P	Oth osteopor w crnt path fx, l shldr, subs for fx w malunion	Arthritis Joint Upper Limb	Nociceptive Pain
M80812S	Oth osteopor w current path fracture, l shoulder, sequela	Arthritis Joint Upper Limb	Nociceptive Pain
M80819	Oth osteopor w current pathological fracture, unsp shoulder	Arthritis Joint Upper Limb	Nociceptive Pain
M80819A	Oth osteopor w current path fracture, unsp shoulder, init	Arthritis Joint Upper Limb	Nociceptive Pain
M80819D	Oth osteopor w crnt path fx, unsp shldr, 7thD	Arthritis Joint Upper Limb	Nociceptive Pain
M80819G	Oth osteopor w crnt path fx, unsp shldr, 7thG	Arthritis Joint Upper Limb	Nociceptive Pain
M80819K	Oth osteopor w crnt path fx, unsp shldr, 7thK	Arthritis Joint Upper Limb	Nociceptive Pain
M80819P	Oth osteopor w crnt path fx, unsp shldr, 7thP	Arthritis Joint Upper Limb	Nociceptive Pain

© 2024 Peterson MD et al. *JAMA Neurology.*

M80819S	Oth osteopor w current path fracture, unsp shoulder, sequela	Arthritis Joint Upper Limb	Nociceptive Pain
M8082	Oth osteoporosis with current pathological fracture, humerus	Arthritis Joint Upper Limb	Nociceptive Pain
M80821	Oth osteoporosis w current pathological fracture, r humerus	Arthritis Joint Upper Limb	Nociceptive Pain
M80821A	Oth osteopor w current path fracture, r humerus, init	Arthritis Joint Upper Limb	Nociceptive Pain
M80821D	Oth osteopor w crnt path fx, r humer, 7thD	Arthritis Joint Upper Limb	Nociceptive Pain
M80821G	Oth osteopor w crnt path fx, r humer, 7thG	Arthritis Joint Upper Limb	Nociceptive Pain
M80821K	Oth osteopor w crnt path fx, r humer, subs for fx w nonunion	Arthritis Joint Upper Limb	Nociceptive Pain
M80821P	Oth osteopor w crnt path fx, r humer, subs for fx w malunion	Arthritis Joint Upper Limb	Nociceptive Pain
M80821S	Oth osteopor w current path fracture, r humerus, sequela	Arthritis Joint Upper Limb	Nociceptive Pain
M80822	Oth osteoporosis w current pathological fracture, l humerus	Arthritis Joint Upper Limb	Nociceptive Pain
M80822A	Oth osteopor w current path fracture, l humerus, init	Arthritis Joint Upper Limb	Nociceptive Pain
M80822D	Oth osteopor w crnt path fx, l humer, 7thD	Arthritis Joint Upper Limb	Nociceptive Pain
M80822G	Oth osteopor w crnt path fx, l humer, 7thG	Arthritis Joint Upper Limb	Nociceptive Pain
M80822K	Oth osteopor w crnt path fx, l humer, subs for fx w nonunion	Arthritis Joint Upper Limb	Nociceptive Pain
M80822P	Oth osteopor w crnt path fx, l humer, subs for fx w malunion	Arthritis Joint Upper Limb	Nociceptive Pain
M80822S	Oth osteopor w current path fracture, l humerus, sequela	Arthritis Joint Upper Limb	Nociceptive Pain
M80829	Oth osteopor w current pathological fracture, unsp humerus	Arthritis Joint Upper Limb	Nociceptive Pain
M80829A	Oth osteopor w current path fracture, unsp humerus, init	Arthritis Joint Upper Limb	Nociceptive Pain
M80829D	Oth osteopor w crnt path fx, unsp humer, 7thD	Arthritis Joint Upper Limb	Nociceptive Pain
M80829G	Oth osteopor w crnt path fx, unsp humer, 7thG	Arthritis Joint Upper Limb	Nociceptive Pain
M80829K	Oth osteopor w crnt path fx, unsp humer, 7thK	Arthritis Joint Upper Limb	Nociceptive Pain
M80829P	Oth osteopor w crnt path fx, unsp humer, 7thP	Arthritis Joint Upper Limb	Nociceptive Pain
M80829S	Oth osteopor w current path fracture, unsp humerus, sequela	Arthritis Joint Upper Limb	Nociceptive Pain
M8083	Oth osteoporosis with current pathological fracture, forearm	Arthritis Joint Upper Limb	Nociceptive Pain
M80831	Oth osteoporosis w current pathological fracture, r forearm	Arthritis Joint Upper Limb	Nociceptive Pain
M80831A	Oth osteopor w current path fracture, r forearm, init	Arthritis Joint Upper Limb	Nociceptive Pain
M80831D	Oth osteopor w crnt path fx, r forearm, 7thD	Arthritis Joint Upper Limb	Nociceptive Pain
M80831G	Oth osteopor w crnt path fx, r forearm, 7thG	Arthritis Joint Upper Limb	Nociceptive Pain
M80831K	Oth osteopor w crnt path fx, r forearm, 7thK	Arthritis Joint Upper Limb	Nociceptive Pain
M80831P	Oth osteopor w crnt path fx, r forearm, 7thP	Arthritis Joint Upper Limb	Nociceptive Pain
M80831S	Oth osteopor w current path fracture, r forearm, sequela	Arthritis Joint Upper Limb	Nociceptive Pain
M80832	Oth osteoporosis w current pathological fracture, l forearm	Arthritis Joint Upper Limb	Nociceptive Pain
M80832A	Oth osteopor w current path fracture, l forearm, init	Arthritis Joint Upper Limb	Nociceptive Pain
M80832D	Oth osteopor w crnt path fx, l forearm, 7thD	Arthritis Joint Upper Limb	Nociceptive Pain
M80832G	Oth osteopor w crnt path fx, l forearm, 7thG	Arthritis Joint Upper Limb	Nociceptive Pain
M80832K	Oth osteopor w crnt path fx, l forearm, 7thK	Arthritis Joint Upper Limb	Nociceptive Pain
M80832P	Oth osteopor w crnt path fx, l forearm, 7thP	Arthritis Joint Upper Limb	Nociceptive Pain

© 2024 Peterson MD et al. *JAMA Neurology.*

M80832S	Oth osteopor w current path fracture, l forearm, sequela	Arthritis Joint Upper Limb	Nociceptive Pain
M80839	Oth osteopor w current pathological fracture, unsp forearm	Arthritis Joint Upper Limb	Nociceptive Pain
M80839A	Oth osteopor w current path fracture, unsp forearm, init	Arthritis Joint Upper Limb	Nociceptive Pain
M80839D	Oth osteopor w crnt path fx, unsp forearm, 7thD	Arthritis Joint Upper Limb	Nociceptive Pain
M80839G	Oth osteopor w crnt path fx, unsp forearm, 7thG	Arthritis Joint Upper Limb	Nociceptive Pain
M80839K	Oth osteopor w crnt path fx, unsp forearm, 7thK	Arthritis Joint Upper Limb	Nociceptive Pain
M80839P	Oth osteopor w crnt path fx, unsp forearm, 7thP	Arthritis Joint Upper Limb	Nociceptive Pain
M80839S	Oth osteopor w current path fracture, unsp forearm, sequela	Arthritis Joint Upper Limb	Nociceptive Pain
M8084	Other osteoporosis with current pathological fracture, hand	Arthritis Joint Upper Limb	Nociceptive Pain
M80841	Oth osteoporosis w current pathological fracture, right hand	Arthritis Joint Upper Limb	Nociceptive Pain
M80841A	Oth osteopor w current path fracture, right hand, init	Arthritis Joint Upper Limb	Nociceptive Pain
M80841D	Oth osteopor w crnt path fx, r hand, 7thD	Arthritis Joint Upper Limb	Nociceptive Pain
M80841G	Oth osteopor w crnt path fx, r hand, 7thG	Arthritis Joint Upper Limb	Nociceptive Pain
M80841K	Oth osteopor w crnt path fx, r hand, subs for fx w nonunion	Arthritis Joint Upper Limb	Nociceptive Pain
M80841P	Oth osteopor w crnt path fx, r hand, subs for fx w malunion	Arthritis Joint Upper Limb	Nociceptive Pain
M80841S	Oth osteopor w current path fracture, right hand, sequela	Arthritis Joint Upper Limb	Nociceptive Pain
M80842	Oth osteoporosis w current pathological fracture, left hand	Arthritis Joint Upper Limb	Nociceptive Pain
M80842A	Oth osteopor w current path fracture, left hand, init	Arthritis Joint Upper Limb	Nociceptive Pain
M80842D	Oth osteopor w crnt path fx, l hand, 7thD	Arthritis Joint Upper Limb	Nociceptive Pain
M80842G	Oth osteopor w crnt path fx, l hand, 7thG	Arthritis Joint Upper Limb	Nociceptive Pain
M80842K	Oth osteopor w crnt path fx, l hand, subs for fx w nonunion	Arthritis Joint Upper Limb	Nociceptive Pain
M80842P	Oth osteopor w crnt path fx, l hand, subs for fx w malunion	Arthritis Joint Upper Limb	Nociceptive Pain
M80842S	Oth osteopor w current path fracture, left hand, sequela	Arthritis Joint Upper Limb	Nociceptive Pain
M80849	Oth osteoporosis w current pathological fracture, unsp hand	Arthritis Joint Upper Limb	Nociceptive Pain
M80849A	Oth osteopor w current path fracture, unsp hand, init	Arthritis Joint Upper Limb	Nociceptive Pain
M80849D	Oth osteopor w crnt path fx, unsp hand, 7thD	Arthritis Joint Upper Limb	Nociceptive Pain
M80849G	Oth osteopor w crnt path fx, unsp hand, 7thG	Arthritis Joint Upper Limb	Nociceptive Pain
M80849K	Oth osteopor w crnt path fx, unsp hand, 7thK	Arthritis Joint Upper Limb	Nociceptive Pain
M80849P	Oth osteopor w crnt path fx, unsp hand, 7thP	Arthritis Joint Upper Limb	Nociceptive Pain
M80849S	Oth osteopor w current path fracture, unsp hand, sequela	Arthritis Joint Upper Limb	Nociceptive Pain
M8085	Other osteoporosis with current pathological fracture, femur	Arthritis Joint Lower Limb	Nociceptive Pain
M80851	Oth osteopor w current pathological fracture, right femur	Arthritis Joint Lower Limb	Nociceptive Pain
M80851A	Oth osteopor w current path fracture, right femur, init	Arthritis Joint Lower Limb	Nociceptive Pain
M80851D	Oth osteopor w crnt path fx, r femr, 7thD	Arthritis Joint Lower Limb	Nociceptive Pain
M80851G	Oth osteopor w crnt path fx, r femr, 7thG	Arthritis Joint Lower Limb	Nociceptive Pain
M80851K	Oth osteopor w crnt path fx, r femur, subs for fx w nonunion	Arthritis Joint Lower Limb	Nociceptive Pain
M80851P	Oth osteopor w crnt path fx, r femur, subs for fx w malunion	Arthritis Joint Lower Limb	Nociceptive Pain

© 2024 Peterson MD et al. *JAMA Neurology.*

M80851S	Oth osteopor w current path fracture, right femur, sequela	Arthritis Joint Lower Limb	Nociceptive Pain
M80852	Oth osteoporosis w current pathological fracture, left femur	Arthritis Joint Lower Limb	Nociceptive Pain
M80852A	Oth osteopor w current path fracture, left femur, init	Arthritis Joint Lower Limb	Nociceptive Pain
M80852D	Oth osteopor w crnt path fx, l femr, 7thD	Arthritis Joint Lower Limb	Nociceptive Pain
M80852G	Oth osteopor w crnt path fx, l femr, 7thG	Arthritis Joint Lower Limb	Nociceptive Pain
M80852K	Oth osteopor w crnt path fx, l femur, subs for fx w nonunion	Arthritis Joint Lower Limb	Nociceptive Pain
M80852P	Oth osteopor w crnt path fx, l femur, subs for fx w malunion	Arthritis Joint Lower Limb	Nociceptive Pain
M80852S	Oth osteopor w current path fracture, left femur, sequela	Arthritis Joint Lower Limb	Nociceptive Pain
M80859	Oth osteoporosis w current pathological fracture, unsp femur	Arthritis Joint Lower Limb	Nociceptive Pain
M80859A	Oth osteopor w current path fracture, unsp femur, init	Arthritis Joint Lower Limb	Nociceptive Pain
M80859D	Oth osteopor w crnt path fx, unsp femr, 7thD	Arthritis Joint Lower Limb	Nociceptive Pain
M80859G	Oth osteopor w crnt path fx, unsp femr, 7thG	Arthritis Joint Lower Limb	Nociceptive Pain
M80859K	Oth osteopor w crnt path fx, unsp femr, 7thK	Arthritis Joint Lower Limb	Nociceptive Pain
M80859P	Oth osteopor w crnt path fx, unsp femr, 7thP	Arthritis Joint Lower Limb	Nociceptive Pain
M80859S	Oth osteopor w current path fracture, unsp femur, sequela	Arthritis Joint Lower Limb	Nociceptive Pain
M8086	Oth osteoporosis w current pathological fracture, lower leg	Arthritis Joint Lower Limb	Nociceptive Pain
M80861	Oth osteoporosis w current pathological fracture, r low leg	Arthritis Joint Lower Limb	Nociceptive Pain
M80861A	Oth osteopor w current path fracture, r low leg, init	Arthritis Joint Lower Limb	Nociceptive Pain
M80861D	Oth osteopor w crnt path fx, r low leg, 7thD	Arthritis Joint Lower Limb	Nociceptive Pain
M80861G	Oth osteopor w crnt path fx, r low leg, 7thG	Arthritis Joint Lower Limb	Nociceptive Pain
M80861K	Oth osteopor w crnt path fx, r low leg, 7thK	Arthritis Joint Lower Limb	Nociceptive Pain
M80861P	Oth osteopor w crnt path fx, r low leg, 7thP	Arthritis Joint Lower Limb	Nociceptive Pain
M80861S	Oth osteopor w current path fracture, r low leg, sequela	Arthritis Joint Lower Limb	Nociceptive Pain
M80862	Oth osteoporosis w current pathological fracture, l low leg	Arthritis Joint Lower Limb	Nociceptive Pain
M80862A	Oth osteopor w current path fracture, l low leg, init	Arthritis Joint Lower Limb	Nociceptive Pain
M80862D	Oth osteopor w crnt path fx, l low leg, 7thD	Arthritis Joint Lower Limb	Nociceptive Pain
M80862G	Oth osteopor w crnt path fx, l low leg, 7thG	Arthritis Joint Lower Limb	Nociceptive Pain
M80862K	Oth osteopor w crnt path fx, l low leg, 7thK	Arthritis Joint Lower Limb	Nociceptive Pain
M80862P	Oth osteopor w crnt path fx, l low leg, 7thP	Arthritis Joint Lower Limb	Nociceptive Pain
M80862S	Oth osteopor w current path fracture, l low leg, sequela	Arthritis Joint Lower Limb	Nociceptive Pain
M80869	Oth osteopor w current pathological fracture, unsp lower leg	Arthritis Joint Lower Limb	Nociceptive Pain
M80869A	Oth osteopor w current path fracture, unsp lower leg, init	Arthritis Joint Lower Limb	Nociceptive Pain
M80869D	Oth osteopor w crnt path fx, unsp low leg, 7thD	Arthritis Joint Lower Limb	Nociceptive Pain
M80869G	Oth osteopor w crnt path fx, unsp low leg, 7thG	Arthritis Joint Lower Limb	Nociceptive Pain
M80869K	Oth osteopor w crnt path fx, unsp low leg, 7thK	Arthritis Joint Lower Limb	Nociceptive Pain
M80869P	Oth osteopor w crnt path fx, unsp low leg, 7thP	Arthritis Joint Lower Limb	Nociceptive Pain
M80869S	Oth osteopor w current path fracture, unsp low leg, sequela	Arthritis Joint Lower Limb	Nociceptive Pain

© 2024 Peterson MD et al. *JAMA Neurology.*

M8087	Oth osteoporosis w current pathological fracture, ank/ft	Arthritis Joint Lower Limb	Nociceptive Pain
M80871	Oth osteopor w current pathological fracture, right ank/ft	Arthritis Joint Lower Limb	Nociceptive Pain
M80871A	Oth osteopor w current path fracture, right ank/ft, init	Arthritis Joint Lower Limb	Nociceptive Pain
M80871D	Oth osteopor w crnt path fx, r ank/ft, 7thD	Arthritis Joint Lower Limb	Nociceptive Pain
M80871G	Oth osteopor w crnt path fx, r ank/ft, 7thG	Arthritis Joint Lower Limb	Nociceptive Pain
M80871K	Oth osteopor w crnt path fx, r ank/ft, 7thK	Arthritis Joint Lower Limb	Nociceptive Pain
M80871P	Oth osteopor w crnt path fx, r ank/ft, 7thP	Arthritis Joint Lower Limb	Nociceptive Pain
M80871S	Oth osteopor w current path fracture, right ank/ft, sequela	Arthritis Joint Lower Limb	Nociceptive Pain
M80872	Oth osteopor w current pathological fracture, left ank/ft	Arthritis Joint Lower Limb	Nociceptive Pain
M80872A	Oth osteopor w current path fracture, left ank/ft, init	Arthritis Joint Lower Limb	Nociceptive Pain
M80872D	Oth osteopor w crnt path fx, l ank/ft, 7thD	Arthritis Joint Lower Limb	Nociceptive Pain
M80872G	Oth osteopor w crnt path fx, l ank/ft, 7thG	Arthritis Joint Lower Limb	Nociceptive Pain
M80872K	Oth osteopor w crnt path fx, l ank/ft, 7thK	Arthritis Joint Lower Limb	Nociceptive Pain
M80872P	Oth osteopor w crnt path fx, l ank/ft, 7thP	Arthritis Joint Lower Limb	Nociceptive Pain
M80872S	Oth osteopor w current path fracture, left ank/ft, sequela	Arthritis Joint Lower Limb	Nociceptive Pain
M80879	Oth osteopor w current pathological fracture, unsp ank/ft	Arthritis Joint Lower Limb	Nociceptive Pain
M80879A	Oth osteopor w current path fracture, unsp ank/ft, init	Arthritis Joint Lower Limb	Nociceptive Pain
M80879D	Oth osteopor w crnt path fx, unsp ank/ft, 7thD	Arthritis Joint Lower Limb	Nociceptive Pain
M80879G	Oth osteopor w crnt path fx, unsp ank/ft, 7thG	Arthritis Joint Lower Limb	Nociceptive Pain
M80879K	Oth osteopor w crnt path fx, unsp ank/ft, 7thK	Arthritis Joint Lower Limb	Nociceptive Pain
M80879P	Oth osteopor w crnt path fx, unsp ank/ft, 7thP	Arthritis Joint Lower Limb	Nociceptive Pain
M80879S	Oth osteopor w current path fracture, unsp ank/ft, sequela	Arthritis Joint Lower Limb	Nociceptive Pain
M8088	Oth osteopor w current pathological fracture, vertebra(e)	Arthritis Joint Spine and Hips	Nociceptive Pain
M8088XA	Oth osteopor w current path fracture, vertebra(e), init	Arthritis Joint Spine and Hips	Nociceptive Pain
M8088XD	Oth osteopor w crnt path fx, verteb, 7thD	Arthritis Joint Spine and Hips	Nociceptive Pain
M8088XG	Oth osteopor w crnt path fx, verteb, 7thG	Arthritis Joint Spine and Hips	Nociceptive Pain
M8088XK	Oth osteopor w crnt path fx, verteb, subs for fx w nonunion	Arthritis Joint Spine and Hips	Nociceptive Pain
M8088XP	Oth osteopor w crnt path fx, verteb, subs for fx w malunion	Arthritis Joint Spine and Hips	Nociceptive Pain
M8088XS	Oth osteopor w current path fracture, vertebra(e), sequela	Arthritis Joint Spine and Hips	Nociceptive Pain
M808A	Other osteopor with current path fracture, other site	Arthritis Joint Other	Nociceptive Pain
M808AXA	Other osteopor with current path fracture, other site, init	Arthritis Joint Other	Nociceptive Pain
M808AXD	Other osteopor with current path fracture, other site, 7thD	Arthritis Joint Other	Nociceptive Pain
M808AXG	Other osteopor with current path fracture, other site, 7thG	Arthritis Joint Other	Nociceptive Pain
M808AXK	Other osteopor with current path fracture, other site, 7thK	Arthritis Joint Other	Nociceptive Pain
M808AXP	Other osteopor with current path fracture, other site, 7thP	Arthritis Joint Other	Nociceptive Pain
M808AXS	Other osteopor with current path fx, other site, sequela	Arthritis Joint Other	Nociceptive Pain
M808B	Other osteopor with current pathological fracture, pelvis	Arthritis Joint Spine and Hips	Nociceptive Pain

© 2024 Peterson MD et al. *JAMA Neurology.*

M808B1	Other osteopor with current path fracture, right pelvis	Arthritis Joint Spine and Hips	Nociceptive Pain
M808B1A	Other osteopor with current path fx, right pelvis, init	Arthritis Joint Spine and Hips	Nociceptive Pain
M808B1D	Other osteopor with current path fx, right pelvis, 7thD	Arthritis Joint Spine and Hips	Nociceptive Pain
M808B1G	Other osteopor with current path fx, right pelvis, 7thG	Arthritis Joint Spine and Hips	Nociceptive Pain
M808B1K	Other osteopor with current path fx, right pelvis, 7thK	Arthritis Joint Spine and Hips	Nociceptive Pain
M808B1P	Other osteopor with current path fx, right pelvis, 7thP	Arthritis Joint Spine and Hips	Nociceptive Pain
M808B1S	Other osteopor with current path fx, right pelvis, sequela	Arthritis Joint Spine and Hips	Nociceptive Pain
M808B2	Other osteopor with current path fracture, left pelvis	Arthritis Joint Spine and Hips	Nociceptive Pain
M808B2A	Other osteopor with current path fracture, left pelvis, init	Arthritis Joint Spine and Hips	Nociceptive Pain
M808B2D	Other osteopor with current path fracture, left pelvis, 7thD	Arthritis Joint Spine and Hips	Nociceptive Pain
M808B2G	Other osteopor with current path fracture, left pelvis, 7thG	Arthritis Joint Spine and Hips	Nociceptive Pain
M808B2K	Other osteopor with current path fracture, left pelvis, 7thK	Arthritis Joint Spine and Hips	Nociceptive Pain
M808B2P	Other osteopor with current path fracture, left pelvis, 7thP	Arthritis Joint Spine and Hips	Nociceptive Pain
M808B2S	Other osteopor with current path fx, left pelvis, sequela	Arthritis Joint Spine and Hips	Nociceptive Pain
M808B9	Other osteopor with current path fracture, unsp pelvis	Arthritis Joint Spine and Hips	Nociceptive Pain
M808B9A	Other osteopor with current path fracture, unsp pelvis, init	Arthritis Joint Spine and Hips	Nociceptive Pain
M808B9D	Other osteopor with current path fracture, unsp pelvis, 7thD	Arthritis Joint Spine and Hips	Nociceptive Pain
M808B9G	Other osteopor with current path fracture, unsp pelvis, 7thG	Arthritis Joint Spine and Hips	Nociceptive Pain
M808B9K	Other osteopor with current path fracture, unsp pelvis, 7thK	Arthritis Joint Spine and Hips	Nociceptive Pain
M808B9P	Other osteopor with current path fracture, unsp pelvis, 7thP	Arthritis Joint Spine and Hips	Nociceptive Pain
M808B9S	Other osteopor with current path fx, unsp pelvis, sequela	Arthritis Joint Spine and Hips	Nociceptive Pain
M81	Osteoporosis without current pathological fracture	Arthritis Joint Other	Nociceptive Pain
M810	Age-related osteoporosis w/o current pathological fracture	Arthritis Joint Other	Nociceptive Pain
M816	Localized osteoporosis [Lequesne]	Arthritis Joint Other	Nociceptive Pain
M818	Other osteoporosis without current pathological fracture	Arthritis Joint Other	Nociceptive Pain
M84	Disorder of continuity of bone	Arthritis Joint Other	Nociceptive Pain
M843	Stress fracture	Arthritis Joint Other	Nociceptive Pain
M8430	Stress fracture, unspecified site	Arthritis Joint Other	Nociceptive Pain
M8430XA	Stress fracture, unspecified site, init encntr for fracture	Arthritis Joint Other	Nociceptive Pain
M8430XD	Stress fracture, unsp site, subs for fx w routn heal	Arthritis Joint Other	Nociceptive Pain
M8430XG	Stress fracture, unsp site, subs for fx w delay heal	Arthritis Joint Other	Nociceptive Pain
M8430XK	Stress fracture, unsp site, subs for fx w nonunion	Arthritis Joint Other	Nociceptive Pain
M8430XP	Stress fracture, unsp site, subs for fx w malunion	Arthritis Joint Other	Nociceptive Pain
M8430XS	Stress fracture, unspecified site, sequela	Arthritis Joint Other	Nociceptive Pain
M8431	Stress fracture, shoulder	Arthritis Joint Upper Limb	Nociceptive Pain
M84311	Stress fracture, right shoulder	Arthritis Joint Upper Limb	Nociceptive Pain
M84311A	Stress fracture, right shoulder, init encntr for fracture	Arthritis Joint Upper Limb	Nociceptive Pain

© 2024 Peterson MD et al. *JAMA Neurology.*

M84311D	Stress fracture, right shoulder, subs for fx w routn heal	Arthritis Joint Upper Limb	Nociceptive Pain
M84311G	Stress fracture, right shoulder, subs for fx w delay heal	Arthritis Joint Upper Limb	Nociceptive Pain
M84311K	Stress fracture, right shoulder, subs for fx w nonunion	Arthritis Joint Upper Limb	Nociceptive Pain
M84311P	Stress fracture, right shoulder, subs for fx w malunion	Arthritis Joint Upper Limb	Nociceptive Pain
M84311S	Stress fracture, right shoulder, sequela	Arthritis Joint Upper Limb	Nociceptive Pain
M84312	Stress fracture, left shoulder	Arthritis Joint Upper Limb	Nociceptive Pain
M84312A	Stress fracture, left shoulder, init encntr for fracture	Arthritis Joint Upper Limb	Nociceptive Pain
M84312D	Stress fracture, left shoulder, subs for fx w routn heal	Arthritis Joint Upper Limb	Nociceptive Pain
M84312G	Stress fracture, left shoulder, subs for fx w delay heal	Arthritis Joint Upper Limb	Nociceptive Pain
M84312K	Stress fracture, left shoulder, subs for fx w nonunion	Arthritis Joint Upper Limb	Nociceptive Pain
M84312P	Stress fracture, left shoulder, subs for fx w malunion	Arthritis Joint Upper Limb	Nociceptive Pain
M84312S	Stress fracture, left shoulder, sequela	Arthritis Joint Upper Limb	Nociceptive Pain
M84319	Stress fracture, unspecified shoulder	Arthritis Joint Upper Limb	Nociceptive Pain
M84319A	Stress fracture, unsp shoulder, init encntr for fracture	Arthritis Joint Upper Limb	Nociceptive Pain
M84319D	Stress fracture, unsp shoulder, subs for fx w routn heal	Arthritis Joint Upper Limb	Nociceptive Pain
M84319G	Stress fracture, unsp shoulder, subs for fx w delay heal	Arthritis Joint Upper Limb	Nociceptive Pain
M84319K	Stress fracture, unsp shoulder, subs for fx w nonunion	Arthritis Joint Upper Limb	Nociceptive Pain
M84319P	Stress fracture, unsp shoulder, subs for fx w malunion	Arthritis Joint Upper Limb	Nociceptive Pain
M84319S	Stress fracture, unspecified shoulder, sequela	Arthritis Joint Upper Limb	Nociceptive Pain
M8432	Stress fracture, humerus	Arthritis Joint Upper Limb	Nociceptive Pain
M84321	Stress fracture, right humerus	Arthritis Joint Upper Limb	Nociceptive Pain
M84321A	Stress fracture, right humerus, init encntr for fracture	Arthritis Joint Upper Limb	Nociceptive Pain
M84321D	Stress fracture, right humerus, subs for fx w routn heal	Arthritis Joint Upper Limb	Nociceptive Pain
M84321G	Stress fracture, right humerus, subs for fx w delay heal	Arthritis Joint Upper Limb	Nociceptive Pain
M84321K	Stress fracture, right humerus, subs for fx w nonunion	Arthritis Joint Upper Limb	Nociceptive Pain
M84321P	Stress fracture, right humerus, subs for fx w malunion	Arthritis Joint Upper Limb	Nociceptive Pain
M84321S	Stress fracture, right humerus, sequela	Arthritis Joint Upper Limb	Nociceptive Pain
M84322	Stress fracture, left humerus	Arthritis Joint Upper Limb	Nociceptive Pain
M84322A	Stress fracture, left humerus, init encntr for fracture	Arthritis Joint Upper Limb	Nociceptive Pain
M84322D	Stress fracture, left humerus, subs for fx w routn heal	Arthritis Joint Upper Limb	Nociceptive Pain
M84322G	Stress fracture, left humerus, subs for fx w delay heal	Arthritis Joint Upper Limb	Nociceptive Pain
M84322K	Stress fracture, left humerus, subs for fx w nonunion	Arthritis Joint Upper Limb	Nociceptive Pain
M84322P	Stress fracture, left humerus, subs for fx w malunion	Arthritis Joint Upper Limb	Nociceptive Pain
M84322S	Stress fracture, left humerus, sequela	Arthritis Joint Upper Limb	Nociceptive Pain
M84329	Stress fracture, unspecified humerus	Arthritis Joint Upper Limb	Nociceptive Pain
M84329A	Stress fracture, unsp humerus, init encntr for fracture	Arthritis Joint Upper Limb	Nociceptive Pain
M84329D	Stress fracture, unsp humerus, subs for fx w routn heal	Arthritis Joint Upper Limb	Nociceptive Pain

© 2024 Peterson MD et al. *JAMA Neurology.*

M84329G	Stress fracture, unsp humerus, subs for fx w delay heal	Arthritis Joint Upper Limb	Nociceptive Pain
M84329K	Stress fracture, unsp humerus, subs for fx w nonunion	Arthritis Joint Upper Limb	Nociceptive Pain
M84329P	Stress fracture, unsp humerus, subs for fx w malunion	Arthritis Joint Upper Limb	Nociceptive Pain
M84329S	Stress fracture, unspecified humerus, sequela	Arthritis Joint Upper Limb	Nociceptive Pain
M8433	Stress fracture, ulna and radius	Arthritis Joint Upper Limb	Nociceptive Pain
M84331	Stress fracture, right ulna	Arthritis Joint Upper Limb	Nociceptive Pain
M84331A	Stress fracture, right ulna, initial encounter for fracture	Arthritis Joint Upper Limb	Nociceptive Pain
M84331D	Stress fracture, right ulna, subs for fx w routn heal	Arthritis Joint Upper Limb	Nociceptive Pain
M84331G	Stress fracture, right ulna, subs for fx w delay heal	Arthritis Joint Upper Limb	Nociceptive Pain
M84331K	Stress fracture, right ulna, subs for fx w nonunion	Arthritis Joint Upper Limb	Nociceptive Pain
M84331P	Stress fracture, right ulna, subs for fx w malunion	Arthritis Joint Upper Limb	Nociceptive Pain
M84331S	Stress fracture, right ulna, sequela	Arthritis Joint Upper Limb	Nociceptive Pain
M84332	Stress fracture, left ulna	Arthritis Joint Upper Limb	Nociceptive Pain
M84332A	Stress fracture, left ulna, initial encounter for fracture	Arthritis Joint Upper Limb	Nociceptive Pain
M84332D	Stress fracture, left ulna, subs for fx w routn heal	Arthritis Joint Upper Limb	Nociceptive Pain
M84332G	Stress fracture, left ulna, subs for fx w delay heal	Arthritis Joint Upper Limb	Nociceptive Pain
M84332K	Stress fracture, left ulna, subs for fx w nonunion	Arthritis Joint Upper Limb	Nociceptive Pain
M84332P	Stress fracture, left ulna, subs for fx w malunion	Arthritis Joint Upper Limb	Nociceptive Pain
M84332S	Stress fracture, left ulna, sequela	Arthritis Joint Upper Limb	Nociceptive Pain
M84333	Stress fracture, right radius	Arthritis Joint Upper Limb	Nociceptive Pain
M84333A	Stress fracture, right radius, init encntr for fracture	Arthritis Joint Upper Limb	Nociceptive Pain
M84333D	Stress fracture, right radius, subs for fx w routn heal	Arthritis Joint Upper Limb	Nociceptive Pain
M84333G	Stress fracture, right radius, subs for fx w delay heal	Arthritis Joint Upper Limb	Nociceptive Pain
M84333K	Stress fracture, right radius, subs for fx w nonunion	Arthritis Joint Upper Limb	Nociceptive Pain
M84333P	Stress fracture, right radius, subs for fx w malunion	Arthritis Joint Upper Limb	Nociceptive Pain
M84333S	Stress fracture, right radius, sequela	Arthritis Joint Upper Limb	Nociceptive Pain
M84334	Stress fracture, left radius	Arthritis Joint Upper Limb	Nociceptive Pain
M84334A	Stress fracture, left radius, initial encounter for fracture	Arthritis Joint Upper Limb	Nociceptive Pain
M84334D	Stress fracture, left radius, subs for fx w routn heal	Arthritis Joint Upper Limb	Nociceptive Pain
M84334G	Stress fracture, left radius, subs for fx w delay heal	Arthritis Joint Upper Limb	Nociceptive Pain
M84334K	Stress fracture, left radius, subs for fx w nonunion	Arthritis Joint Upper Limb	Nociceptive Pain
M84334P	Stress fracture, left radius, subs for fx w malunion	Arthritis Joint Upper Limb	Nociceptive Pain
M84334S	Stress fracture, left radius, sequela	Arthritis Joint Upper Limb	Nociceptive Pain
M84339	Stress fracture, unspecified ulna and radius	Arthritis Joint Upper Limb	Nociceptive Pain
M84339A	Stress fracture, unsp ulna and radius, init for fx	Arthritis Joint Upper Limb	Nociceptive Pain
M84339D	Stress fx, unsp ulna and radius, subs for fx w routn heal	Arthritis Joint Upper Limb	Nociceptive Pain
M84339G	Stress fx, unsp ulna and radius, subs for fx w delay heal	Arthritis Joint Upper Limb	Nociceptive Pain

© 2024 Peterson MD et al. *JAMA Neurology.*

M84339K	Stress fx, unsp ulna and radius, subs for fx w nonunion	Arthritis Joint Upper Limb	Nociceptive Pain
M84339P	Stress fx, unsp ulna and radius, subs for fx w malunion	Arthritis Joint Upper Limb	Nociceptive Pain
M84339S	Stress fracture, unspecified ulna and radius, sequela	Arthritis Joint Upper Limb	Nociceptive Pain
M8434	Stress fracture, hand and fingers	Arthritis Joint Upper Limb	Nociceptive Pain
M84341	Stress fracture, right hand	Arthritis Joint Upper Limb	Nociceptive Pain
M84341A	Stress fracture, right hand, initial encounter for fracture	Arthritis Joint Upper Limb	Nociceptive Pain
M84341D	Stress fracture, right hand, subs for fx w routn heal	Arthritis Joint Upper Limb	Nociceptive Pain
M84341G	Stress fracture, right hand, subs for fx w delay heal	Arthritis Joint Upper Limb	Nociceptive Pain
M84341K	Stress fracture, right hand, subs for fx w nonunion	Arthritis Joint Upper Limb	Nociceptive Pain
M84341P	Stress fracture, right hand, subs for fx w malunion	Arthritis Joint Upper Limb	Nociceptive Pain
M84341S	Stress fracture, right hand, sequela	Arthritis Joint Upper Limb	Nociceptive Pain
M84342	Stress fracture, left hand	Arthritis Joint Upper Limb	Nociceptive Pain
M84342A	Stress fracture, left hand, initial encounter for fracture	Arthritis Joint Upper Limb	Nociceptive Pain
M84342D	Stress fracture, left hand, subs for fx w routn heal	Arthritis Joint Upper Limb	Nociceptive Pain
M84342G	Stress fracture, left hand, subs for fx w delay heal	Arthritis Joint Upper Limb	Nociceptive Pain
M84342K	Stress fracture, left hand, subs for fx w nonunion	Arthritis Joint Upper Limb	Nociceptive Pain
M84342P	Stress fracture, left hand, subs for fx w malunion	Arthritis Joint Upper Limb	Nociceptive Pain
M84342S	Stress fracture, left hand, sequela	Arthritis Joint Upper Limb	Nociceptive Pain
M84343	Stress fracture, unspecified hand	Arthritis Joint Upper Limb	Nociceptive Pain
M84343A	Stress fracture, unspecified hand, init encntr for fracture	Arthritis Joint Upper Limb	Nociceptive Pain
M84343D	Stress fracture, unsp hand, subs for fx w routn heal	Arthritis Joint Upper Limb	Nociceptive Pain
M84343G	Stress fracture, unsp hand, subs for fx w delay heal	Arthritis Joint Upper Limb	Nociceptive Pain
M84343K	Stress fracture, unsp hand, subs for fx w nonunion	Arthritis Joint Upper Limb	Nociceptive Pain
M84343P	Stress fracture, unsp hand, subs for fx w malunion	Arthritis Joint Upper Limb	Nociceptive Pain
M84343S	Stress fracture, unspecified hand, sequela	Arthritis Joint Upper Limb	Nociceptive Pain
M84344	Stress fracture, right finger(s)	Arthritis Joint Upper Limb	Nociceptive Pain
M84344A	Stress fracture, right finger(s), init encntr for fracture	Arthritis Joint Upper Limb	Nociceptive Pain
M84344D	Stress fracture, right finger(s), subs for fx w routn heal	Arthritis Joint Upper Limb	Nociceptive Pain
M84344G	Stress fracture, right finger(s), subs for fx w delay heal	Arthritis Joint Upper Limb	Nociceptive Pain
M84344K	Stress fracture, right finger(s), subs for fx w nonunion	Arthritis Joint Upper Limb	Nociceptive Pain
M84344P	Stress fracture, right finger(s), subs for fx w malunion	Arthritis Joint Upper Limb	Nociceptive Pain
M84344S	Stress fracture, right finger(s), sequela	Arthritis Joint Upper Limb	Nociceptive Pain
M84345	Stress fracture, left finger(s)	Arthritis Joint Upper Limb	Nociceptive Pain
M84345A	Stress fracture, left finger(s), init encntr for fracture	Arthritis Joint Upper Limb	Nociceptive Pain
M84345D	Stress fracture, left finger(s), subs for fx w routn heal	Arthritis Joint Upper Limb	Nociceptive Pain
M84345G	Stress fracture, left finger(s), subs for fx w delay heal	Arthritis Joint Upper Limb	Nociceptive Pain
M84345K	Stress fracture, left finger(s), subs for fx w nonunion	Arthritis Joint Upper Limb	Nociceptive Pain

© 2024 Peterson MD et al. *JAMA Neurology.*

M84345P	Stress fracture, left finger(s), subs for fx w malunion	Arthritis Joint Upper Limb	Nociceptive Pain
M84345S	Stress fracture, left finger(s), sequela	Arthritis Joint Upper Limb	Nociceptive Pain
M84346	Stress fracture, unspecified finger(s)	Arthritis Joint Upper Limb	Nociceptive Pain
M84346A	Stress fracture, unsp finger(s), init encntr for fracture	Arthritis Joint Upper Limb	Nociceptive Pain
M84346D	Stress fracture, unsp finger(s), subs for fx w routn heal	Arthritis Joint Upper Limb	Nociceptive Pain
M84346G	Stress fracture, unsp finger(s), subs for fx w delay heal	Arthritis Joint Upper Limb	Nociceptive Pain
M84346K	Stress fracture, unsp finger(s), subs for fx w nonunion	Arthritis Joint Upper Limb	Nociceptive Pain
M84346P	Stress fracture, unsp finger(s), subs for fx w malunion	Arthritis Joint Upper Limb	Nociceptive Pain
M84346S	Stress fracture, unspecified finger(s), sequela	Arthritis Joint Upper Limb	Nociceptive Pain
M8435	Stress fracture, pelvis and femur	Arthritis Joint Lower Limb	Nociceptive Pain
M84350	Stress fracture, pelvis	Arthritis Joint Spine and Hips	Nociceptive Pain
M84350A	Stress fracture, pelvis, initial encounter for fracture	Arthritis Joint Spine and Hips	Nociceptive Pain
M84350D	Stress fracture, pelvis, subs for fx w routn heal	Arthritis Joint Spine and Hips	Nociceptive Pain
M84350G	Stress fracture, pelvis, subs for fx w delay heal	Arthritis Joint Spine and Hips	Nociceptive Pain
M84350K	Stress fracture, pelvis, subs encntr for fracture w nonunion	Arthritis Joint Spine and Hips	Nociceptive Pain
M84350P	Stress fracture, pelvis, subs encntr for fracture w malunion	Arthritis Joint Spine and Hips	Nociceptive Pain
M84350S	Stress fracture, pelvis, sequela	Arthritis Joint Spine and Hips	Nociceptive Pain
M84351	Stress fracture, right femur	Arthritis Joint Lower Limb	Nociceptive Pain
M84351A	Stress fracture, right femur, initial encounter for fracture	Arthritis Joint Lower Limb	Nociceptive Pain
M84351D	Stress fracture, right femur, subs for fx w routn heal	Arthritis Joint Lower Limb	Nociceptive Pain
M84351G	Stress fracture, right femur, subs for fx w delay heal	Arthritis Joint Lower Limb	Nociceptive Pain
M84351K	Stress fracture, right femur, subs for fx w nonunion	Arthritis Joint Lower Limb	Nociceptive Pain
M84351P	Stress fracture, right femur, subs for fx w malunion	Arthritis Joint Lower Limb	Nociceptive Pain
M84351S	Stress fracture, right femur, sequela	Arthritis Joint Lower Limb	Nociceptive Pain
M84352	Stress fracture, left femur	Arthritis Joint Lower Limb	Nociceptive Pain
M84352A	Stress fracture, left femur, initial encounter for fracture	Arthritis Joint Lower Limb	Nociceptive Pain
M84352D	Stress fracture, left femur, subs for fx w routn heal	Arthritis Joint Lower Limb	Nociceptive Pain
M84352G	Stress fracture, left femur, subs for fx w delay heal	Arthritis Joint Lower Limb	Nociceptive Pain
M84352K	Stress fracture, left femur, subs for fx w nonunion	Arthritis Joint Lower Limb	Nociceptive Pain
M84352P	Stress fracture, left femur, subs for fx w malunion	Arthritis Joint Lower Limb	Nociceptive Pain
M84352S	Stress fracture, left femur, sequela	Arthritis Joint Lower Limb	Nociceptive Pain
M84353	Stress fracture, unspecified femur	Arthritis Joint Lower Limb	Nociceptive Pain
M84353A	Stress fracture, unspecified femur, init encntr for fracture	Arthritis Joint Lower Limb	Nociceptive Pain
M84353D	Stress fracture, unsp femur, subs for fx w routn heal	Arthritis Joint Lower Limb	Nociceptive Pain
M84353G	Stress fracture, unsp femur, subs for fx w delay heal	Arthritis Joint Lower Limb	Nociceptive Pain
M84353K	Stress fracture, unsp femur, subs for fx w nonunion	Arthritis Joint Lower Limb	Nociceptive Pain
M84353P	Stress fracture, unsp femur, subs for fx w malunion	Arthritis Joint Lower Limb	Nociceptive Pain

© 2024 Peterson MD et al. *JAMA Neurology.*

M84353S	Stress fracture, unspecified femur, sequela	Arthritis Joint Lower Limb	Nociceptive Pain
M84359	Stress fracture, hip, unspecified	Arthritis Joint Spine and Hips	Nociceptive Pain
M84359A	Stress fracture, hip, unspecified, init encntr for fracture	Arthritis Joint Spine and Hips	Nociceptive Pain
M84359D	Stress fracture, hip, unsp, subs for fx w routn heal	Arthritis Joint Spine and Hips	Nociceptive Pain
M84359G	Stress fracture, hip, unsp, subs for fx w delay heal	Arthritis Joint Spine and Hips	Nociceptive Pain
M84359K	Stress fracture, hip, unsp, subs for fx w nonunion	Arthritis Joint Spine and Hips	Nociceptive Pain
M84359P	Stress fracture, hip, unsp, subs for fx w malunion	Arthritis Joint Spine and Hips	Nociceptive Pain
M84359S	Stress fracture, hip, unspecified, sequela	Arthritis Joint Spine and Hips	Nociceptive Pain
M8436	Stress fracture, tibia and fibula	Arthritis Joint Lower Limb	Nociceptive Pain
M84361	Stress fracture, right tibia	Arthritis Joint Lower Limb	Nociceptive Pain
M84361A	Stress fracture, right tibia, initial encounter for fracture	Arthritis Joint Lower Limb	Nociceptive Pain
M84361D	Stress fracture, right tibia, subs for fx w routn heal	Arthritis Joint Lower Limb	Nociceptive Pain
M84361G	Stress fracture, right tibia, subs for fx w delay heal	Arthritis Joint Lower Limb	Nociceptive Pain
M84361K	Stress fracture, right tibia, subs for fx w nonunion	Arthritis Joint Lower Limb	Nociceptive Pain
M84361P	Stress fracture, right tibia, subs for fx w malunion	Arthritis Joint Lower Limb	Nociceptive Pain
M84361S	Stress fracture, right tibia, sequela	Arthritis Joint Lower Limb	Nociceptive Pain
M84362	Stress fracture, left tibia	Arthritis Joint Lower Limb	Nociceptive Pain
M84362A	Stress fracture, left tibia, initial encounter for fracture	Arthritis Joint Lower Limb	Nociceptive Pain
M84362D	Stress fracture, left tibia, subs for fx w routn heal	Arthritis Joint Lower Limb	Nociceptive Pain
M84362G	Stress fracture, left tibia, subs for fx w delay heal	Arthritis Joint Lower Limb	Nociceptive Pain
M84362K	Stress fracture, left tibia, subs for fx w nonunion	Arthritis Joint Lower Limb	Nociceptive Pain
M84362P	Stress fracture, left tibia, subs for fx w malunion	Arthritis Joint Lower Limb	Nociceptive Pain
M84362S	Stress fracture, left tibia, sequela	Arthritis Joint Lower Limb	Nociceptive Pain
M84363	Stress fracture, right fibula	Arthritis Joint Lower Limb	Nociceptive Pain
M84363A	Stress fracture, right fibula, init encntr for fracture	Arthritis Joint Lower Limb	Nociceptive Pain
M84363D	Stress fracture, right fibula, subs for fx w routn heal	Arthritis Joint Lower Limb	Nociceptive Pain
M84363G	Stress fracture, right fibula, subs for fx w delay heal	Arthritis Joint Lower Limb	Nociceptive Pain
M84363K	Stress fracture, right fibula, subs for fx w nonunion	Arthritis Joint Lower Limb	Nociceptive Pain
M84363P	Stress fracture, right fibula, subs for fx w malunion	Arthritis Joint Lower Limb	Nociceptive Pain
M84363S	Stress fracture, right fibula, sequela	Arthritis Joint Lower Limb	Nociceptive Pain
M84364	Stress fracture, left fibula	Arthritis Joint Lower Limb	Nociceptive Pain
M84364A	Stress fracture, left fibula, initial encounter for fracture	Arthritis Joint Lower Limb	Nociceptive Pain
M84364D	Stress fracture, left fibula, subs for fx w routn heal	Arthritis Joint Lower Limb	Nociceptive Pain
M84364G	Stress fracture, left fibula, subs for fx w delay heal	Arthritis Joint Lower Limb	Nociceptive Pain
M84364K	Stress fracture, left fibula, subs for fx w nonunion	Arthritis Joint Lower Limb	Nociceptive Pain
M84364P	Stress fracture, left fibula, subs for fx w malunion	Arthritis Joint Lower Limb	Nociceptive Pain
M84364S	Stress fracture, left fibula, sequela	Arthritis Joint Lower Limb	Nociceptive Pain

© 2024 Peterson MD et al. *JAMA Neurology.*

M84369	Stress fracture, unspecified tibia and fibula	Arthritis Joint Lower Limb	Nociceptive Pain
M84369A	Stress fracture, unsp tibia and fibula, init for fx	Arthritis Joint Lower Limb	Nociceptive Pain
M84369D	Stress fx, unsp tibia and fibula, subs for fx w routn heal	Arthritis Joint Lower Limb	Nociceptive Pain
M84369G	Stress fx, unsp tibia and fibula, subs for fx w delay heal	Arthritis Joint Lower Limb	Nociceptive Pain
M84369K	Stress fx, unsp tibia and fibula, subs for fx w nonunion	Arthritis Joint Lower Limb	Nociceptive Pain
M84369P	Stress fx, unsp tibia and fibula, subs for fx w malunion	Arthritis Joint Lower Limb	Nociceptive Pain
M84369S	Stress fracture, unspecified tibia and fibula, sequela	Arthritis Joint Lower Limb	Nociceptive Pain
M8437	Stress fracture, ankle, foot and toes	Arthritis Joint Lower Limb	Nociceptive Pain
M84371	Stress fracture, right ankle	Arthritis Joint Lower Limb	Nociceptive Pain
M84371A	Stress fracture, right ankle, initial encounter for fracture	Arthritis Joint Lower Limb	Nociceptive Pain
M84371D	Stress fracture, right ankle, subs for fx w routn heal	Arthritis Joint Lower Limb	Nociceptive Pain
M84371G	Stress fracture, right ankle, subs for fx w delay heal	Arthritis Joint Lower Limb	Nociceptive Pain
M84371K	Stress fracture, right ankle, subs for fx w nonunion	Arthritis Joint Lower Limb	Nociceptive Pain
M84371P	Stress fracture, right ankle, subs for fx w malunion	Arthritis Joint Lower Limb	Nociceptive Pain
M84371S	Stress fracture, right ankle, sequela	Arthritis Joint Lower Limb	Nociceptive Pain
M84372	Stress fracture, left ankle	Arthritis Joint Lower Limb	Nociceptive Pain
M84372A	Stress fracture, left ankle, initial encounter for fracture	Arthritis Joint Lower Limb	Nociceptive Pain
M84372D	Stress fracture, left ankle, subs for fx w routn heal	Arthritis Joint Lower Limb	Nociceptive Pain
M84372G	Stress fracture, left ankle, subs for fx w delay heal	Arthritis Joint Lower Limb	Nociceptive Pain
M84372K	Stress fracture, left ankle, subs for fx w nonunion	Arthritis Joint Lower Limb	Nociceptive Pain
M84372P	Stress fracture, left ankle, subs for fx w malunion	Arthritis Joint Lower Limb	Nociceptive Pain
M84372S	Stress fracture, left ankle, sequela	Arthritis Joint Lower Limb	Nociceptive Pain
M84373	Stress fracture, unspecified ankle	Arthritis Joint Lower Limb	Nociceptive Pain
M84373A	Stress fracture, unspecified ankle, init encntr for fracture	Arthritis Joint Lower Limb	Nociceptive Pain
M84373D	Stress fracture, unsp ankle, subs for fx w routn heal	Arthritis Joint Lower Limb	Nociceptive Pain
M84373G	Stress fracture, unsp ankle, subs for fx w delay heal	Arthritis Joint Lower Limb	Nociceptive Pain
M84373K	Stress fracture, unsp ankle, subs for fx w nonunion	Arthritis Joint Lower Limb	Nociceptive Pain
M84373P	Stress fracture, unsp ankle, subs for fx w malunion	Arthritis Joint Lower Limb	Nociceptive Pain
M84373S	Stress fracture, unspecified ankle, sequela	Arthritis Joint Lower Limb	Nociceptive Pain
M84374	Stress fracture, right foot	Arthritis Joint Lower Limb	Nociceptive Pain
M84374A	Stress fracture, right foot, initial encounter for fracture	Arthritis Joint Lower Limb	Nociceptive Pain
M84374D	Stress fracture, right foot, subs for fx w routn heal	Arthritis Joint Lower Limb	Nociceptive Pain
M84374G	Stress fracture, right foot, subs for fx w delay heal	Arthritis Joint Lower Limb	Nociceptive Pain
M84374K	Stress fracture, right foot, subs for fx w nonunion	Arthritis Joint Lower Limb	Nociceptive Pain
M84374P	Stress fracture, right foot, subs for fx w malunion	Arthritis Joint Lower Limb	Nociceptive Pain
M84374S	Stress fracture, right foot, sequela	Arthritis Joint Lower Limb	Nociceptive Pain
M84375	Stress fracture, left foot	Arthritis Joint Lower Limb	Nociceptive Pain

© 2024 Peterson MD et al. *JAMA Neurology.*

M84375A	Stress fracture, left foot, initial encounter for fracture	Arthritis Joint Lower Limb	Nociceptive Pain
M84375D	Stress fracture, left foot, subs for fx w routn heal	Arthritis Joint Lower Limb	Nociceptive Pain
M84375G	Stress fracture, left foot, subs for fx w delay heal	Arthritis Joint Lower Limb	Nociceptive Pain
M84375K	Stress fracture, left foot, subs for fx w nonunion	Arthritis Joint Lower Limb	Nociceptive Pain
M84375P	Stress fracture, left foot, subs for fx w malunion	Arthritis Joint Lower Limb	Nociceptive Pain
M84375S	Stress fracture, left foot, sequela	Arthritis Joint Lower Limb	Nociceptive Pain
M84376	Stress fracture, unspecified foot	Arthritis Joint Lower Limb	Nociceptive Pain
M84376A	Stress fracture, unspecified foot, init encntr for fracture	Arthritis Joint Lower Limb	Nociceptive Pain
M84376D	Stress fracture, unsp foot, subs for fx w routn heal	Arthritis Joint Lower Limb	Nociceptive Pain
M84376G	Stress fracture, unsp foot, subs for fx w delay heal	Arthritis Joint Lower Limb	Nociceptive Pain
M84376K	Stress fracture, unsp foot, subs for fx w nonunion	Arthritis Joint Lower Limb	Nociceptive Pain
M84376P	Stress fracture, unsp foot, subs for fx w malunion	Arthritis Joint Lower Limb	Nociceptive Pain
M84376S	Stress fracture, unspecified foot, sequela	Arthritis Joint Lower Limb	Nociceptive Pain
M84377	Stress fracture, right toe(s)	Arthritis Joint Lower Limb	Nociceptive Pain
M84377A	Stress fracture, right toe(s), init encntr for fracture	Arthritis Joint Lower Limb	Nociceptive Pain
M84377D	Stress fracture, right toe(s), subs for fx w routn heal	Arthritis Joint Lower Limb	Nociceptive Pain
M84377G	Stress fracture, right toe(s), subs for fx w delay heal	Arthritis Joint Lower Limb	Nociceptive Pain
M84377K	Stress fracture, right toe(s), subs for fx w nonunion	Arthritis Joint Lower Limb	Nociceptive Pain
M84377P	Stress fracture, right toe(s), subs for fx w malunion	Arthritis Joint Lower Limb	Nociceptive Pain
M84377S	Stress fracture, right toe(s), sequela	Arthritis Joint Lower Limb	Nociceptive Pain
M84378	Stress fracture, left toe(s)	Arthritis Joint Lower Limb	Nociceptive Pain
M84378A	Stress fracture, left toe(s), initial encounter for fracture	Arthritis Joint Lower Limb	Nociceptive Pain
M84378D	Stress fracture, left toe(s), subs for fx w routn heal	Arthritis Joint Lower Limb	Nociceptive Pain
M84378G	Stress fracture, left toe(s), subs for fx w delay heal	Arthritis Joint Lower Limb	Nociceptive Pain
M84378K	Stress fracture, left toe(s), subs for fx w nonunion	Arthritis Joint Lower Limb	Nociceptive Pain
M84378P	Stress fracture, left toe(s), subs for fx w malunion	Arthritis Joint Lower Limb	Nociceptive Pain
M84378S	Stress fracture, left toe(s), sequela	Arthritis Joint Lower Limb	Nociceptive Pain
M84379	Stress fracture, unspecified toe(s)	Arthritis Joint Lower Limb	Nociceptive Pain
M84379A	Stress fracture, unsp toe(s), init encntr for fracture	Arthritis Joint Lower Limb	Nociceptive Pain
M84379D	Stress fracture, unsp toe(s), subs for fx w routn heal	Arthritis Joint Lower Limb	Nociceptive Pain
M84379G	Stress fracture, unsp toe(s), subs for fx w delay heal	Arthritis Joint Lower Limb	Nociceptive Pain
M84379K	Stress fracture, unsp toe(s), subs for fx w nonunion	Arthritis Joint Lower Limb	Nociceptive Pain
M84379P	Stress fracture, unsp toe(s), subs for fx w malunion	Arthritis Joint Lower Limb	Nociceptive Pain
M84379S	Stress fracture, unspecified toe(s), sequela	Arthritis Joint Lower Limb	Nociceptive Pain
M8438	Stress fracture, other site	Arthritis Joint Other	Nociceptive Pain
M8438XA	Stress fracture, other site, initial encounter for fracture	Arthritis Joint Other	Nociceptive Pain
M8438XD	Stress fracture, oth site, subs for fx w routn heal	Arthritis Joint Other	Nociceptive Pain

© 2024 Peterson MD et al. *JAMA Neurology.*

M8438XG	Stress fracture, oth site, subs for fx w delay heal	Arthritis Joint Other	Nociceptive Pain
M8438XK	Stress fracture, oth site, subs for fx w nonunion	Arthritis Joint Other	Nociceptive Pain
M8438XP	Stress fracture, oth site, subs for fx w malunion	Arthritis Joint Other	Nociceptive Pain
M8438XS	Stress fracture, other site, sequela	Arthritis Joint Other	Nociceptive Pain
M844	Pathological fracture, not elsewhere classified	Arthritis Joint Other	Nociceptive Pain
M8440	Pathological fracture, unspecified site	Arthritis Joint Other	Nociceptive Pain
M8440XA	Pathological fracture, unsp site, init encntr for fracture	Arthritis Joint Other	Nociceptive Pain
M8440XD	Pathological fracture, unsp site, subs for fx w routn heal	Arthritis Joint Other	Nociceptive Pain
M8440XG	Pathological fracture, unsp site, subs for fx w delay heal	Arthritis Joint Other	Nociceptive Pain
M8440XK	Pathological fracture, unsp site, subs for fx w nonunion	Arthritis Joint Other	Nociceptive Pain
M8440XP	Pathological fracture, unsp site, subs for fx w malunion	Arthritis Joint Other	Nociceptive Pain
M8440XS	Pathological fracture, unspecified site, sequela	Arthritis Joint Other	Nociceptive Pain
M8441	Pathological fracture, shoulder	Arthritis Joint Upper Limb	Nociceptive Pain
M84411	Pathological fracture, right shoulder	Arthritis Joint Upper Limb	Nociceptive Pain
M84411A	Pathological fracture, right shoulder, init for fx	Arthritis Joint Upper Limb	Nociceptive Pain
M84411D	Pathological fracture, r shoulder, subs for fx w routn heal	Arthritis Joint Upper Limb	Nociceptive Pain
M84411G	Pathological fracture, r shoulder, subs for fx w delay heal	Arthritis Joint Upper Limb	Nociceptive Pain
M84411K	Pathological fracture, r shoulder, subs for fx w nonunion	Arthritis Joint Upper Limb	Nociceptive Pain
M84411P	Pathological fracture, r shoulder, subs for fx w malunion	Arthritis Joint Upper Limb	Nociceptive Pain
M84411S	Pathological fracture, right shoulder, sequela	Arthritis Joint Upper Limb	Nociceptive Pain
M84412	Pathological fracture, left shoulder	Arthritis Joint Upper Limb	Nociceptive Pain
M84412A	Pathological fracture, left shoulder, init for fx	Arthritis Joint Upper Limb	Nociceptive Pain
M84412D	Pathological fracture, l shoulder, subs for fx w routn heal	Arthritis Joint Upper Limb	Nociceptive Pain
M84412G	Pathological fracture, l shoulder, subs for fx w delay heal	Arthritis Joint Upper Limb	Nociceptive Pain
M84412K	Pathological fracture, left shoulder, subs for fx w nonunion	Arthritis Joint Upper Limb	Nociceptive Pain
M84412P	Pathological fracture, left shoulder, subs for fx w malunion	Arthritis Joint Upper Limb	Nociceptive Pain
M84412S	Pathological fracture, left shoulder, sequela	Arthritis Joint Upper Limb	Nociceptive Pain
M84419	Pathological fracture, unspecified shoulder	Arthritis Joint Upper Limb	Nociceptive Pain
M84419A	Pathological fracture, unsp shoulder, init for fx	Arthritis Joint Upper Limb	Nociceptive Pain
M84419D	Path fracture, unsp shoulder, subs for fx w routn heal	Arthritis Joint Upper Limb	Nociceptive Pain
M84419G	Path fracture, unsp shoulder, subs for fx w delay heal	Arthritis Joint Upper Limb	Nociceptive Pain
M84419K	Pathological fracture, unsp shoulder, subs for fx w nonunion	Arthritis Joint Upper Limb	Nociceptive Pain
M84419P	Pathological fracture, unsp shoulder, subs for fx w malunion	Arthritis Joint Upper Limb	Nociceptive Pain
M84419S	Pathological fracture, unspecified shoulder, sequela	Arthritis Joint Upper Limb	Nociceptive Pain
M8442	Pathological fracture, humerus	Arthritis Joint Upper Limb	Nociceptive Pain
M84421	Pathological fracture, right humerus	Arthritis Joint Upper Limb	Nociceptive Pain
M84421A	Pathological fracture, right humerus, init for fx	Arthritis Joint Upper Limb	Nociceptive Pain

© 2024 Peterson MD et al. *JAMA Neurology.*

M84421D	Pathological fracture, r humerus, subs for fx w routn heal	Arthritis Joint Upper Limb	Nociceptive Pain
M84421G	Pathological fracture, r humerus, subs for fx w delay heal	Arthritis Joint Upper Limb	Nociceptive Pain
M84421K	Pathological fracture, right humerus, subs for fx w nonunion	Arthritis Joint Upper Limb	Nociceptive Pain
M84421P	Pathological fracture, right humerus, subs for fx w malunion	Arthritis Joint Upper Limb	Nociceptive Pain
M84421S	Pathological fracture, right humerus, sequela	Arthritis Joint Upper Limb	Nociceptive Pain
M84422	Pathological fracture, left humerus	Arthritis Joint Upper Limb	Nociceptive Pain
M84422A	Pathological fracture, left humerus, init for fx	Arthritis Joint Upper Limb	Nociceptive Pain
M84422D	Pathological fracture, l humerus, subs for fx w routn heal	Arthritis Joint Upper Limb	Nociceptive Pain
M84422G	Pathological fracture, l humerus, subs for fx w delay heal	Arthritis Joint Upper Limb	Nociceptive Pain
M84422K	Pathological fracture, left humerus, subs for fx w nonunion	Arthritis Joint Upper Limb	Nociceptive Pain
M84422P	Pathological fracture, left humerus, subs for fx w malunion	Arthritis Joint Upper Limb	Nociceptive Pain
M84422S	Pathological fracture, left humerus, sequela	Arthritis Joint Upper Limb	Nociceptive Pain
M84429	Pathological fracture, unspecified humerus	Arthritis Joint Upper Limb	Nociceptive Pain
M84429A	Pathological fracture, unsp humerus, init for fx	Arthritis Joint Upper Limb	Nociceptive Pain
M84429D	Path fracture, unsp humerus, subs for fx w routn heal	Arthritis Joint Upper Limb	Nociceptive Pain
M84429G	Path fracture, unsp humerus, subs for fx w delay heal	Arthritis Joint Upper Limb	Nociceptive Pain
M84429K	Pathological fracture, unsp humerus, subs for fx w nonunion	Arthritis Joint Upper Limb	Nociceptive Pain
M84429P	Pathological fracture, unsp humerus, subs for fx w malunion	Arthritis Joint Upper Limb	Nociceptive Pain
M84429S	Pathological fracture, unspecified humerus, sequela	Arthritis Joint Upper Limb	Nociceptive Pain
M8443	Pathological fracture, ulna and radius	Arthritis Joint Upper Limb	Nociceptive Pain
M84431	Pathological fracture, right ulna	Arthritis Joint Upper Limb	Nociceptive Pain
M84431A	Pathological fracture, right ulna, init encntr for fracture	Arthritis Joint Upper Limb	Nociceptive Pain
M84431D	Pathological fracture, right ulna, subs for fx w routn heal	Arthritis Joint Upper Limb	Nociceptive Pain
M84431G	Pathological fracture, right ulna, subs for fx w delay heal	Arthritis Joint Upper Limb	Nociceptive Pain
M84431K	Pathological fracture, right ulna, subs for fx w nonunion	Arthritis Joint Upper Limb	Nociceptive Pain
M84431P	Pathological fracture, right ulna, subs for fx w malunion	Arthritis Joint Upper Limb	Nociceptive Pain
M84431S	Pathological fracture, right ulna, sequela	Arthritis Joint Upper Limb	Nociceptive Pain
M84432	Pathological fracture, left ulna	Arthritis Joint Upper Limb	Nociceptive Pain
M84432A	Pathological fracture, left ulna, init encntr for fracture	Arthritis Joint Upper Limb	Nociceptive Pain
M84432D	Pathological fracture, left ulna, subs for fx w routn heal	Arthritis Joint Upper Limb	Nociceptive Pain
M84432G	Pathological fracture, left ulna, subs for fx w delay heal	Arthritis Joint Upper Limb	Nociceptive Pain
M84432K	Pathological fracture, left ulna, subs for fx w nonunion	Arthritis Joint Upper Limb	Nociceptive Pain
M84432P	Pathological fracture, left ulna, subs for fx w malunion	Arthritis Joint Upper Limb	Nociceptive Pain
M84432S	Pathological fracture, left ulna, sequela	Arthritis Joint Upper Limb	Nociceptive Pain
M84433	Pathological fracture, right radius	Arthritis Joint Upper Limb	Nociceptive Pain
M84433A	Pathological fracture, right radius, init for fx	Arthritis Joint Upper Limb	Nociceptive Pain
M84433D	Path fracture, right radius, subs for fx w routn heal	Arthritis Joint Upper Limb	Nociceptive Pain

© 2024 Peterson MD et al. *JAMA Neurology.*

M84433G	Path fracture, right radius, subs for fx w delay heal	Arthritis Joint Upper Limb	Nociceptive Pain
M84433K	Pathological fracture, right radius, subs for fx w nonunion	Arthritis Joint Upper Limb	Nociceptive Pain
M84433P	Pathological fracture, right radius, subs for fx w malunion	Arthritis Joint Upper Limb	Nociceptive Pain
M84433S	Pathological fracture, right radius, sequela	Arthritis Joint Upper Limb	Nociceptive Pain
M84434	Pathological fracture, left radius	Arthritis Joint Upper Limb	Nociceptive Pain
M84434A	Pathological fracture, left radius, init encntr for fracture	Arthritis Joint Upper Limb	Nociceptive Pain
M84434D	Pathological fracture, left radius, subs for fx w routn heal	Arthritis Joint Upper Limb	Nociceptive Pain
M84434G	Pathological fracture, left radius, subs for fx w delay heal	Arthritis Joint Upper Limb	Nociceptive Pain
M84434K	Pathological fracture, left radius, subs for fx w nonunion	Arthritis Joint Upper Limb	Nociceptive Pain
M84434P	Pathological fracture, left radius, subs for fx w malunion	Arthritis Joint Upper Limb	Nociceptive Pain
M84434S	Pathological fracture, left radius, sequela	Arthritis Joint Upper Limb	Nociceptive Pain
M84439	Pathological fracture, unspecified ulna and radius	Arthritis Joint Upper Limb	Nociceptive Pain
M84439A	Pathological fracture, unsp ulna and radius, init for fx	Arthritis Joint Upper Limb	Nociceptive Pain
M84439D	Path fx, unsp ulna and radius, subs for fx w routn heal	Arthritis Joint Upper Limb	Nociceptive Pain
M84439G	Path fx, unsp ulna and radius, subs for fx w delay heal	Arthritis Joint Upper Limb	Nociceptive Pain
M84439K	Path fracture, unsp ulna and radius, subs for fx w nonunion	Arthritis Joint Upper Limb	Nociceptive Pain
M84439P	Path fracture, unsp ulna and radius, subs for fx w malunion	Arthritis Joint Upper Limb	Nociceptive Pain
M84439S	Pathological fracture, unspecified ulna and radius, sequela	Arthritis Joint Upper Limb	Nociceptive Pain
M8444	Pathological fracture, hand and fingers	Arthritis Joint Upper Limb	Nociceptive Pain
M84441	Pathological fracture, right hand	Arthritis Joint Upper Limb	Nociceptive Pain
M84441A	Pathological fracture, right hand, init encntr for fracture	Arthritis Joint Upper Limb	Nociceptive Pain
M84441D	Pathological fracture, right hand, subs for fx w routn heal	Arthritis Joint Upper Limb	Nociceptive Pain
M84441G	Pathological fracture, right hand, subs for fx w delay heal	Arthritis Joint Upper Limb	Nociceptive Pain
M84441K	Pathological fracture, right hand, subs for fx w nonunion	Arthritis Joint Upper Limb	Nociceptive Pain
M84441P	Pathological fracture, right hand, subs for fx w malunion	Arthritis Joint Upper Limb	Nociceptive Pain
M84441S	Pathological fracture, right hand, sequela	Arthritis Joint Upper Limb	Nociceptive Pain
M84442	Pathological fracture, left hand	Arthritis Joint Upper Limb	Nociceptive Pain
M84442A	Pathological fracture, left hand, init encntr for fracture	Arthritis Joint Upper Limb	Nociceptive Pain
M84442D	Pathological fracture, left hand, subs for fx w routn heal	Arthritis Joint Upper Limb	Nociceptive Pain
M84442G	Pathological fracture, left hand, subs for fx w delay heal	Arthritis Joint Upper Limb	Nociceptive Pain
M84442K	Pathological fracture, left hand, subs for fx w nonunion	Arthritis Joint Upper Limb	Nociceptive Pain
M84442P	Pathological fracture, left hand, subs for fx w malunion	Arthritis Joint Upper Limb	Nociceptive Pain
M84442S	Pathological fracture, left hand, sequela	Arthritis Joint Upper Limb	Nociceptive Pain
M84443	Pathological fracture, unspecified hand	Arthritis Joint Upper Limb	Nociceptive Pain
M84443A	Pathological fracture, unsp hand, init encntr for fracture	Arthritis Joint Upper Limb	Nociceptive Pain
M84443D	Pathological fracture, unsp hand, subs for fx w routn heal	Arthritis Joint Upper Limb	Nociceptive Pain
M84443G	Pathological fracture, unsp hand, subs for fx w delay heal	Arthritis Joint Upper Limb	Nociceptive Pain

© 2024 Peterson MD et al. *JAMA Neurology.*

M84443K	Pathological fracture, unsp hand, subs for fx w nonunion	Arthritis Joint Upper Limb	Nociceptive Pain
M84443P	Pathological fracture, unsp hand, subs for fx w malunion	Arthritis Joint Upper Limb	Nociceptive Pain
M84443S	Pathological fracture, unspecified hand, sequela	Arthritis Joint Upper Limb	Nociceptive Pain
M84444	Pathological fracture, right finger(s)	Arthritis Joint Upper Limb	Nociceptive Pain
M84444A	Pathological fracture, right finger(s), init for fx	Arthritis Joint Upper Limb	Nociceptive Pain
M84444D	Path fracture, right finger(s), subs for fx w routn heal	Arthritis Joint Upper Limb	Nociceptive Pain
M84444G	Path fracture, right finger(s), subs for fx w delay heal	Arthritis Joint Upper Limb	Nociceptive Pain
M84444K	Path fracture, right finger(s), subs for fx w nonunion	Arthritis Joint Upper Limb	Nociceptive Pain
M84444P	Path fracture, right finger(s), subs for fx w malunion	Arthritis Joint Upper Limb	Nociceptive Pain
M84444S	Pathological fracture, right finger(s), sequela	Arthritis Joint Upper Limb	Nociceptive Pain
M84445	Pathological fracture, left finger(s)	Arthritis Joint Upper Limb	Nociceptive Pain
M84445A	Pathological fracture, left finger(s), init for fx	Arthritis Joint Upper Limb	Nociceptive Pain
M84445D	Path fracture, left finger(s), subs for fx w routn heal	Arthritis Joint Upper Limb	Nociceptive Pain
M84445G	Path fracture, left finger(s), subs for fx w delay heal	Arthritis Joint Upper Limb	Nociceptive Pain
M84445K	Path fracture, left finger(s), subs for fx w nonunion	Arthritis Joint Upper Limb	Nociceptive Pain
M84445P	Path fracture, left finger(s), subs for fx w malunion	Arthritis Joint Upper Limb	Nociceptive Pain
M84445S	Pathological fracture, left finger(s), sequela	Arthritis Joint Upper Limb	Nociceptive Pain
M84446	Pathological fracture, unspecified finger(s)	Arthritis Joint Upper Limb	Nociceptive Pain
M84446A	Pathological fracture, unsp finger(s), init for fx	Arthritis Joint Upper Limb	Nociceptive Pain
M84446D	Path fracture, unsp finger(s), subs for fx w routn heal	Arthritis Joint Upper Limb	Nociceptive Pain
M84446G	Path fracture, unsp finger(s), subs for fx w delay heal	Arthritis Joint Upper Limb	Nociceptive Pain
M84446K	Path fracture, unsp finger(s), subs for fx w nonunion	Arthritis Joint Upper Limb	Nociceptive Pain
M84446P	Path fracture, unsp finger(s), subs for fx w malunion	Arthritis Joint Upper Limb	Nociceptive Pain
M84446S	Pathological fracture, unspecified finger(s), sequela	Arthritis Joint Upper Limb	Nociceptive Pain
M8445	Pathological fracture, femur and pelvis	Arthritis Joint Lower Limb	Nociceptive Pain
M84451	Pathological fracture, right femur	Arthritis Joint Lower Limb	Nociceptive Pain
M84451A	Pathological fracture, right femur, init encntr for fracture	Arthritis Joint Lower Limb	Nociceptive Pain
M84451D	Pathological fracture, right femur, subs for fx w routn heal	Arthritis Joint Lower Limb	Nociceptive Pain
M84451G	Pathological fracture, right femur, subs for fx w delay heal	Arthritis Joint Lower Limb	Nociceptive Pain
M84451K	Pathological fracture, right femur, subs for fx w nonunion	Arthritis Joint Lower Limb	Nociceptive Pain
M84451P	Pathological fracture, right femur, subs for fx w malunion	Arthritis Joint Lower Limb	Nociceptive Pain
M84451S	Pathological fracture, right femur, sequela	Arthritis Joint Lower Limb	Nociceptive Pain
M84452	Pathological fracture, left femur	Arthritis Joint Lower Limb	Nociceptive Pain
M84452A	Pathological fracture, left femur, init encntr for fracture	Arthritis Joint Lower Limb	Nociceptive Pain
M84452D	Pathological fracture, left femur, subs for fx w routn heal	Arthritis Joint Lower Limb	Nociceptive Pain
M84452G	Pathological fracture, left femur, subs for fx w delay heal	Arthritis Joint Lower Limb	Nociceptive Pain
M84452K	Pathological fracture, left femur, subs for fx w nonunion	Arthritis Joint Lower Limb	Nociceptive Pain

© 2024 Peterson MD et al. *JAMA Neurology.*

M84452P	Pathological fracture, left femur, subs for fx w malunion	Arthritis Joint Lower Limb	Nociceptive Pain
M84452S	Pathological fracture, left femur, sequela	Arthritis Joint Lower Limb	Nociceptive Pain
M84453	Pathological fracture, unspecified femur	Arthritis Joint Lower Limb	Nociceptive Pain
M84453A	Pathological fracture, unsp femur, init encntr for fracture	Arthritis Joint Lower Limb	Nociceptive Pain
M84453D	Pathological fracture, unsp femur, subs for fx w routn heal	Arthritis Joint Lower Limb	Nociceptive Pain
M84453G	Pathological fracture, unsp femur, subs for fx w delay heal	Arthritis Joint Lower Limb	Nociceptive Pain
M84453K	Pathological fracture, unsp femur, subs for fx w nonunion	Arthritis Joint Lower Limb	Nociceptive Pain
M84453P	Pathological fracture, unsp femur, subs for fx w malunion	Arthritis Joint Lower Limb	Nociceptive Pain
M84453S	Pathological fracture, unspecified femur, sequela	Arthritis Joint Lower Limb	Nociceptive Pain
M84454	Pathological fracture, pelvis	Arthritis Joint Spine and Hips	Nociceptive Pain
M84454A	Pathological fracture, pelvis, init encntr for fracture	Arthritis Joint Spine and Hips	Nociceptive Pain
M84454D	Pathological fracture, pelvis, subs for fx w routn heal	Arthritis Joint Spine and Hips	Nociceptive Pain
M84454G	Pathological fracture, pelvis, subs for fx w delay heal	Arthritis Joint Spine and Hips	Nociceptive Pain
M84454K	Pathological fracture, pelvis, subs for fx w nonunion	Arthritis Joint Spine and Hips	Nociceptive Pain
M84454P	Pathological fracture, pelvis, subs for fx w malunion	Arthritis Joint Spine and Hips	Nociceptive Pain
M84454S	Pathological fracture, pelvis, sequela	Arthritis Joint Spine and Hips	Nociceptive Pain
M84459	Pathological fracture, hip, unspecified	Arthritis Joint Spine and Hips	Nociceptive Pain
M84459A	Pathological fracture, hip, unsp, init encntr for fracture	Arthritis Joint Spine and Hips	Nociceptive Pain
M84459D	Pathological fracture, hip, unsp, subs for fx w routn heal	Arthritis Joint Spine and Hips	Nociceptive Pain
M84459G	Pathological fracture, hip, unsp, subs for fx w delay heal	Arthritis Joint Spine and Hips	Nociceptive Pain
M84459K	Pathological fracture, hip, unsp, subs for fx w nonunion	Arthritis Joint Spine and Hips	Nociceptive Pain
M84459P	Pathological fracture, hip, unsp, subs for fx w malunion	Arthritis Joint Spine and Hips	Nociceptive Pain
M84459S	Pathological fracture, hip, unspecified, sequela	Arthritis Joint Spine and Hips	Nociceptive Pain
M8446	Pathological fracture, tibia and fibula	Arthritis Joint Lower Limb	Nociceptive Pain
M84461	Pathological fracture, right tibia	Arthritis Joint Lower Limb	Nociceptive Pain
M84461A	Pathological fracture, right tibia, init encntr for fracture	Arthritis Joint Lower Limb	Nociceptive Pain
M84461D	Pathological fracture, right tibia, subs for fx w routn heal	Arthritis Joint Lower Limb	Nociceptive Pain
M84461G	Pathological fracture, right tibia, subs for fx w delay heal	Arthritis Joint Lower Limb	Nociceptive Pain
M84461K	Pathological fracture, right tibia, subs for fx w nonunion	Arthritis Joint Lower Limb	Nociceptive Pain
M84461P	Pathological fracture, right tibia, subs for fx w malunion	Arthritis Joint Lower Limb	Nociceptive Pain
M84461S	Pathological fracture, right tibia, sequela	Arthritis Joint Lower Limb	Nociceptive Pain
M84462	Pathological fracture, left tibia	Arthritis Joint Lower Limb	Nociceptive Pain
M84462A	Pathological fracture, left tibia, init encntr for fracture	Arthritis Joint Lower Limb	Nociceptive Pain
M84462D	Pathological fracture, left tibia, subs for fx w routn heal	Arthritis Joint Lower Limb	Nociceptive Pain
M84462G	Pathological fracture, left tibia, subs for fx w delay heal	Arthritis Joint Lower Limb	Nociceptive Pain
M84462K	Pathological fracture, left tibia, subs for fx w nonunion	Arthritis Joint Lower Limb	Nociceptive Pain
M84462P	Pathological fracture, left tibia, subs for fx w malunion	Arthritis Joint Lower Limb	Nociceptive Pain

© 2024 Peterson MD et al. *JAMA Neurology.*

M84462S	Pathological fracture, left tibia, sequela	Arthritis Joint Lower Limb	Nociceptive Pain
M84463	Pathological fracture, right fibula	Arthritis Joint Lower Limb	Nociceptive Pain
M84463A	Pathological fracture, right fibula, init for fx	Arthritis Joint Lower Limb	Nociceptive Pain
M84463D	Path fracture, right fibula, subs for fx w routn heal	Arthritis Joint Lower Limb	Nociceptive Pain
M84463G	Path fracture, right fibula, subs for fx w delay heal	Arthritis Joint Lower Limb	Nociceptive Pain
M84463K	Pathological fracture, right fibula, subs for fx w nonunion	Arthritis Joint Lower Limb	Nociceptive Pain
M84463P	Pathological fracture, right fibula, subs for fx w malunion	Arthritis Joint Lower Limb	Nociceptive Pain
M84463S	Pathological fracture, right fibula, sequela	Arthritis Joint Lower Limb	Nociceptive Pain
M84464	Pathological fracture, left fibula	Arthritis Joint Lower Limb	Nociceptive Pain
M84464A	Pathological fracture, left fibula, init encntr for fracture	Arthritis Joint Lower Limb	Nociceptive Pain
M84464D	Pathological fracture, left fibula, subs for fx w routn heal	Arthritis Joint Lower Limb	Nociceptive Pain
M84464G	Pathological fracture, left fibula, subs for fx w delay heal	Arthritis Joint Lower Limb	Nociceptive Pain
M84464K	Pathological fracture, left fibula, subs for fx w nonunion	Arthritis Joint Lower Limb	Nociceptive Pain
M84464P	Pathological fracture, left fibula, subs for fx w malunion	Arthritis Joint Lower Limb	Nociceptive Pain
M84464S	Pathological fracture, left fibula, sequela	Arthritis Joint Lower Limb	Nociceptive Pain
M84469	Pathological fracture, unspecified tibia and fibula	Arthritis Joint Lower Limb	Nociceptive Pain
M84469A	Pathological fracture, unsp tibia and fibula, init for fx	Arthritis Joint Lower Limb	Nociceptive Pain
M84469D	Path fx, unsp tibia and fibula, subs for fx w routn heal	Arthritis Joint Lower Limb	Nociceptive Pain
M84469G	Path fx, unsp tibia and fibula, subs for fx w delay heal	Arthritis Joint Lower Limb	Nociceptive Pain
M84469K	Path fracture, unsp tibia and fibula, subs for fx w nonunion	Arthritis Joint Lower Limb	Nociceptive Pain
M84469P	Path fracture, unsp tibia and fibula, subs for fx w malunion	Arthritis Joint Lower Limb	Nociceptive Pain
M84469S	Pathological fracture, unspecified tibia and fibula, sequela	Arthritis Joint Lower Limb	Nociceptive Pain
M8447	Pathological fracture, ankle, foot and toes	Arthritis Joint Lower Limb	Nociceptive Pain
M84471	Pathological fracture, right ankle	Arthritis Joint Lower Limb	Nociceptive Pain
M84471A	Pathological fracture, right ankle, init encntr for fracture	Arthritis Joint Lower Limb	Nociceptive Pain
M84471D	Pathological fracture, right ankle, subs for fx w routn heal	Arthritis Joint Lower Limb	Nociceptive Pain
M84471G	Pathological fracture, right ankle, subs for fx w delay heal	Arthritis Joint Lower Limb	Nociceptive Pain
M84471K	Pathological fracture, right ankle, subs for fx w nonunion	Arthritis Joint Lower Limb	Nociceptive Pain
M84471P	Pathological fracture, right ankle, subs for fx w malunion	Arthritis Joint Lower Limb	Nociceptive Pain
M84471S	Pathological fracture, right ankle, sequela	Arthritis Joint Lower Limb	Nociceptive Pain
M84472	Pathological fracture, left ankle	Arthritis Joint Lower Limb	Nociceptive Pain
M84472A	Pathological fracture, left ankle, init encntr for fracture	Arthritis Joint Lower Limb	Nociceptive Pain
M84472D	Pathological fracture, left ankle, subs for fx w routn heal	Arthritis Joint Lower Limb	Nociceptive Pain
M84472G	Pathological fracture, left ankle, subs for fx w delay heal	Arthritis Joint Lower Limb	Nociceptive Pain
M84472K	Pathological fracture, left ankle, subs for fx w nonunion	Arthritis Joint Lower Limb	Nociceptive Pain
M84472P	Pathological fracture, left ankle, subs for fx w malunion	Arthritis Joint Lower Limb	Nociceptive Pain
M84472S	Pathological fracture, left ankle, sequela	Arthritis Joint Lower Limb	Nociceptive Pain

© 2024 Peterson MD et al. *JAMA Neurology.*

M84473	Pathological fracture, unspecified ankle	Arthritis Joint Lower Limb	Nociceptive Pain
M84473A	Pathological fracture, unsp ankle, init encntr for fracture	Arthritis Joint Lower Limb	Nociceptive Pain
M84473D	Pathological fracture, unsp ankle, subs for fx w routn heal	Arthritis Joint Lower Limb	Nociceptive Pain
M84473G	Pathological fracture, unsp ankle, subs for fx w delay heal	Arthritis Joint Lower Limb	Nociceptive Pain
M84473K	Pathological fracture, unsp ankle, subs for fx w nonunion	Arthritis Joint Lower Limb	Nociceptive Pain
M84473P	Pathological fracture, unsp ankle, subs for fx w malunion	Arthritis Joint Lower Limb	Nociceptive Pain
M84473S	Pathological fracture, unspecified ankle, sequela	Arthritis Joint Lower Limb	Nociceptive Pain
M84474	Pathological fracture, right foot	Arthritis Joint Lower Limb	Nociceptive Pain
M84474A	Pathological fracture, right foot, init encntr for fracture	Arthritis Joint Lower Limb	Nociceptive Pain
M84474D	Pathological fracture, right foot, subs for fx w routn heal	Arthritis Joint Lower Limb	Nociceptive Pain
M84474G	Pathological fracture, right foot, subs for fx w delay heal	Arthritis Joint Lower Limb	Nociceptive Pain
M84474K	Pathological fracture, right foot, subs for fx w nonunion	Arthritis Joint Lower Limb	Nociceptive Pain
M84474P	Pathological fracture, right foot, subs for fx w malunion	Arthritis Joint Lower Limb	Nociceptive Pain
M84474S	Pathological fracture, right foot, sequela	Arthritis Joint Lower Limb	Nociceptive Pain
M84475	Pathological fracture, left foot	Arthritis Joint Lower Limb	Nociceptive Pain
M84475A	Pathological fracture, left foot, init encntr for fracture	Arthritis Joint Lower Limb	Nociceptive Pain
M84475D	Pathological fracture, left foot, subs for fx w routn heal	Arthritis Joint Lower Limb	Nociceptive Pain
M84475G	Pathological fracture, left foot, subs for fx w delay heal	Arthritis Joint Lower Limb	Nociceptive Pain
M84475K	Pathological fracture, left foot, subs for fx w nonunion	Arthritis Joint Lower Limb	Nociceptive Pain
M84475P	Pathological fracture, left foot, subs for fx w malunion	Arthritis Joint Lower Limb	Nociceptive Pain
M84475S	Pathological fracture, left foot, sequela	Arthritis Joint Lower Limb	Nociceptive Pain
M84476	Pathological fracture, unspecified foot	Arthritis Joint Lower Limb	Nociceptive Pain
M84476A	Pathological fracture, unsp foot, init encntr for fracture	Arthritis Joint Lower Limb	Nociceptive Pain
M84476D	Pathological fracture, unsp foot, subs for fx w routn heal	Arthritis Joint Lower Limb	Nociceptive Pain
M84476G	Pathological fracture, unsp foot, subs for fx w delay heal	Arthritis Joint Lower Limb	Nociceptive Pain
M84476K	Pathological fracture, unsp foot, subs for fx w nonunion	Arthritis Joint Lower Limb	Nociceptive Pain
M84476P	Pathological fracture, unsp foot, subs for fx w malunion	Arthritis Joint Lower Limb	Nociceptive Pain
M84476S	Pathological fracture, unspecified foot, sequela	Arthritis Joint Lower Limb	Nociceptive Pain
M84477	Pathological fracture, right toe(s)	Arthritis Joint Lower Limb	Nociceptive Pain
M84477A	Pathological fracture, right toe(s), init for fx	Arthritis Joint Lower Limb	Nociceptive Pain
M84477D	Path fracture, right toe(s), subs for fx w routn heal	Arthritis Joint Lower Limb	Nociceptive Pain
M84477G	Path fracture, right toe(s), subs for fx w delay heal	Arthritis Joint Lower Limb	Nociceptive Pain
M84477K	Pathological fracture, right toe(s), subs for fx w nonunion	Arthritis Joint Lower Limb	Nociceptive Pain
M84477P	Pathological fracture, right toe(s), subs for fx w malunion	Arthritis Joint Lower Limb	Nociceptive Pain
M84477S	Pathological fracture, right toe(s), sequela	Arthritis Joint Lower Limb	Nociceptive Pain
M84478	Pathological fracture, left toe(s)	Arthritis Joint Lower Limb	Nociceptive Pain
M84478A	Pathological fracture, left toe(s), init encntr for fracture	Arthritis Joint Lower Limb	Nociceptive Pain

© 2024 Peterson MD et al. *JAMA Neurology.*

M84478D	Pathological fracture, left toe(s), subs for fx w routn heal	Arthritis Joint Lower Limb	Nociceptive Pain
M84478G	Pathological fracture, left toe(s), subs for fx w delay heal	Arthritis Joint Lower Limb	Nociceptive Pain
M84478K	Pathological fracture, left toe(s), subs for fx w nonunion	Arthritis Joint Lower Limb	Nociceptive Pain
M84478P	Pathological fracture, left toe(s), subs for fx w malunion	Arthritis Joint Lower Limb	Nociceptive Pain
M84478S	Pathological fracture, left toe(s), sequela	Arthritis Joint Lower Limb	Nociceptive Pain
M84479	Pathological fracture, unspecified toe(s)	Arthritis Joint Lower Limb	Nociceptive Pain
M84479A	Pathological fracture, unsp toe(s), init encntr for fracture	Arthritis Joint Lower Limb	Nociceptive Pain
M84479D	Pathological fracture, unsp toe(s), subs for fx w routn heal	Arthritis Joint Lower Limb	Nociceptive Pain
M84479G	Pathological fracture, unsp toe(s), subs for fx w delay heal	Arthritis Joint Lower Limb	Nociceptive Pain
M84479K	Pathological fracture, unsp toe(s), subs for fx w nonunion	Arthritis Joint Lower Limb	Nociceptive Pain
M84479P	Pathological fracture, unsp toe(s), subs for fx w malunion	Arthritis Joint Lower Limb	Nociceptive Pain
M84479S	Pathological fracture, unspecified toe(s), sequela	Arthritis Joint Lower Limb	Nociceptive Pain
M8448	Pathological fracture, other site	Arthritis Joint Other	Nociceptive Pain
M8448XA	Pathological fracture, other site, init encntr for fracture	Arthritis Joint Other	Nociceptive Pain
M8448XD	Pathological fracture, oth site, subs for fx w routn heal	Arthritis Joint Other	Nociceptive Pain
M8448XG	Pathological fracture, oth site, subs for fx w delay heal	Arthritis Joint Other	Nociceptive Pain
M8448XK	Pathological fracture, oth site, subs for fx w nonunion	Arthritis Joint Other	Nociceptive Pain
M8448XP	Pathological fracture, oth site, subs for fx w malunion	Arthritis Joint Other	Nociceptive Pain
M8448XS	Pathological fracture, other site, sequela	Arthritis Joint Other	Nociceptive Pain
M845	Pathological fracture in neoplastic disease	Arthritis Joint Other	Nociceptive Pain
M8450	Pathological fracture in neoplastic disease, unsp site	Arthritis Joint Other	Nociceptive Pain
M8450XA	Pathological fracture in neoplastic disease, unsp site, init	Arthritis Joint Other	Nociceptive Pain
M8450XD	Path fx in neopltc dis, unsp site, subs for fx w routn heal	Arthritis Joint Other	Nociceptive Pain
M8450XG	Path fx in neopltc dis, unsp site, subs for fx w delay heal	Arthritis Joint Other	Nociceptive Pain
M8450XK	Path fx in neopltc dis, unsp site, subs for fx w nonunion	Arthritis Joint Other	Nociceptive Pain
M8450XP	Path fx in neopltc dis, unsp site, subs for fx w malunion	Arthritis Joint Other	Nociceptive Pain
M8450XS	Path fracture in neoplastic disease, unsp site, sequela	Arthritis Joint Other	Nociceptive Pain
M8451	Pathological fracture in neoplastic disease, shoulder	Arthritis Joint Upper Limb	Nociceptive Pain
M84511	Pathological fracture in neoplastic disease, right shoulder	Arthritis Joint Upper Limb	Nociceptive Pain
M84511A	Path fracture in neoplastic disease, r shoulder, init	Arthritis Joint Upper Limb	Nociceptive Pain
M84511D	Path fx in neopltc dis, r shldr, subs for fx w routn heal	Arthritis Joint Upper Limb	Nociceptive Pain
M84511G	Path fx in neopltc dis, r shldr, subs for fx w delay heal	Arthritis Joint Upper Limb	Nociceptive Pain
M84511K	Path fx in neopltc disease, r shldr, subs for fx w nonunion	Arthritis Joint Upper Limb	Nociceptive Pain
M84511P	Path fx in neopltc disease, r shldr, subs for fx w malunion	Arthritis Joint Upper Limb	Nociceptive Pain
M84511S	Path fracture in neoplastic disease, r shoulder, sequela	Arthritis Joint Upper Limb	Nociceptive Pain
M84512	Pathological fracture in neoplastic disease, left shoulder	Arthritis Joint Upper Limb	Nociceptive Pain
M84512A	Path fracture in neoplastic disease, l shoulder, init	Arthritis Joint Upper Limb	Nociceptive Pain

© 2024 Peterson MD et al. *JAMA Neurology.*

M84512D	Path fx in neopltc dis, l shldr, subs for fx w routn heal	Arthritis Joint Upper Limb	Nociceptive Pain
M84512G	Path fx in neopltc dis, l shldr, subs for fx w delay heal	Arthritis Joint Upper Limb	Nociceptive Pain
M84512K	Path fx in neopltc disease, l shldr, subs for fx w nonunion	Arthritis Joint Upper Limb	Nociceptive Pain
M84512P	Path fx in neopltc disease, l shldr, subs for fx w malunion	Arthritis Joint Upper Limb	Nociceptive Pain
M84512S	Path fracture in neoplastic disease, l shoulder, sequela	Arthritis Joint Upper Limb	Nociceptive Pain
M84519	Pathological fracture in neoplastic disease, unsp shoulder	Arthritis Joint Upper Limb	Nociceptive Pain
M84519A	Path fracture in neoplastic disease, unsp shoulder, init	Arthritis Joint Upper Limb	Nociceptive Pain
M84519D	Path fx in neopltc dis, unsp shldr, subs for fx w routn heal	Arthritis Joint Upper Limb	Nociceptive Pain
M84519G	Path fx in neopltc dis, unsp shldr, subs for fx w delay heal	Arthritis Joint Upper Limb	Nociceptive Pain
M84519K	Path fx in neopltc dis, unsp shldr, subs for fx w nonunion	Arthritis Joint Upper Limb	Nociceptive Pain
M84519P	Path fx in neopltc dis, unsp shldr, subs for fx w malunion	Arthritis Joint Upper Limb	Nociceptive Pain
M84519S	Path fracture in neoplastic disease, unsp shoulder, sequela	Arthritis Joint Upper Limb	Nociceptive Pain
M8452	Pathological fracture in neoplastic disease, humerus	Arthritis Joint Upper Limb	Nociceptive Pain
M84521	Pathological fracture in neoplastic disease, right humerus	Arthritis Joint Upper Limb	Nociceptive Pain
M84521A	Pathological fracture in neoplastic disease, r humerus, init	Arthritis Joint Upper Limb	Nociceptive Pain
M84521D	Path fx in neopltc dis, r humerus, subs for fx w routn heal	Arthritis Joint Upper Limb	Nociceptive Pain
M84521G	Path fx in neopltc dis, r humerus, subs for fx w delay heal	Arthritis Joint Upper Limb	Nociceptive Pain
M84521K	Path fx in neopltc dis, r humerus, subs for fx w nonunion	Arthritis Joint Upper Limb	Nociceptive Pain
M84521P	Path fx in neopltc dis, r humerus, subs for fx w malunion	Arthritis Joint Upper Limb	Nociceptive Pain
M84521S	Path fracture in neoplastic disease, r humerus, sequela	Arthritis Joint Upper Limb	Nociceptive Pain
M84522	Pathological fracture in neoplastic disease, left humerus	Arthritis Joint Upper Limb	Nociceptive Pain
M84522A	Pathological fracture in neoplastic disease, l humerus, init	Arthritis Joint Upper Limb	Nociceptive Pain
M84522D	Path fx in neopltc dis, l humerus, subs for fx w routn heal	Arthritis Joint Upper Limb	Nociceptive Pain
M84522G	Path fx in neopltc dis, l humerus, subs for fx w delay heal	Arthritis Joint Upper Limb	Nociceptive Pain
M84522K	Path fx in neopltc dis, l humerus, subs for fx w nonunion	Arthritis Joint Upper Limb	Nociceptive Pain
M84522P	Path fx in neopltc dis, l humerus, subs for fx w malunion	Arthritis Joint Upper Limb	Nociceptive Pain
M84522S	Path fracture in neoplastic disease, l humerus, sequela	Arthritis Joint Upper Limb	Nociceptive Pain
M84529	Pathological fracture in neoplastic disease, unsp humerus	Arthritis Joint Upper Limb	Nociceptive Pain
M84529A	Path fracture in neoplastic disease, unsp humerus, init	Arthritis Joint Upper Limb	Nociceptive Pain
M84529D	Path fx in neopltc dis, unsp humer, subs for fx w routn heal	Arthritis Joint Other	Nociceptive Pain
M84529G	Path fx in neopltc dis, unsp humer, subs for fx w delay heal	Arthritis Joint Other	Nociceptive Pain
M84529K	Path fx in neopltc dis, unsp humerus, subs for fx w nonunion	Arthritis Joint Upper Limb	Nociceptive Pain
M84529P	Path fx in neopltc dis, unsp humerus, subs for fx w malunion	Arthritis Joint Upper Limb	Nociceptive Pain
M84529S	Path fracture in neoplastic disease, unsp humerus, sequela	Arthritis Joint Upper Limb	Nociceptive Pain
M8453	Pathological fracture in neoplastic disease, ulna and radius	Arthritis Joint Upper Limb	Nociceptive Pain
M84531	Pathological fracture in neoplastic disease, right ulna	Arthritis Joint Upper Limb	Nociceptive Pain
M84531A	Path fracture in neoplastic disease, right ulna, init	Arthritis Joint Upper Limb	Nociceptive Pain

© 2024 Peterson MD et al. *JAMA Neurology.*

M84531D	Path fx in neopltc disease, r ulna, subs for fx w routn heal	Arthritis Joint Upper Limb	Nociceptive Pain
M84531G	Path fx in neopltc disease, r ulna, subs for fx w delay heal	Arthritis Joint Upper Limb	Nociceptive Pain
M84531K	Path fx in neopltc disease, r ulna, subs for fx w nonunion	Arthritis Joint Upper Limb	Nociceptive Pain
M84531P	Path fx in neopltc disease, r ulna, subs for fx w malunion	Arthritis Joint Upper Limb	Nociceptive Pain
M84531S	Path fracture in neoplastic disease, right ulna, sequela	Arthritis Joint Upper Limb	Nociceptive Pain
M84532	Pathological fracture in neoplastic disease, left ulna	Arthritis Joint Upper Limb	Nociceptive Pain
M84532A	Pathological fracture in neoplastic disease, left ulna, init	Arthritis Joint Upper Limb	Nociceptive Pain
M84532D	Path fx in neopltc disease, l ulna, subs for fx w routn heal	Arthritis Joint Upper Limb	Nociceptive Pain
M84532G	Path fx in neopltc disease, l ulna, subs for fx w delay heal	Arthritis Joint Upper Limb	Nociceptive Pain
M84532K	Path fx in neopltc disease, l ulna, subs for fx w nonunion	Arthritis Joint Upper Limb	Nociceptive Pain
M84532P	Path fx in neopltc disease, l ulna, subs for fx w malunion	Arthritis Joint Upper Limb	Nociceptive Pain
M84532S	Path fracture in neoplastic disease, left ulna, sequela	Arthritis Joint Upper Limb	Nociceptive Pain
M84533	Pathological fracture in neoplastic disease, right radius	Arthritis Joint Upper Limb	Nociceptive Pain
M84533A	Path fracture in neoplastic disease, right radius, init	Arthritis Joint Upper Limb	Nociceptive Pain
M84533D	Path fx in neopltc dis, r radius, subs for fx w routn heal	Arthritis Joint Upper Limb	Nociceptive Pain
M84533G	Path fx in neopltc dis, r radius, subs for fx w delay heal	Arthritis Joint Upper Limb	Nociceptive Pain
M84533K	Path fx in neopltc disease, r radius, subs for fx w nonunion	Arthritis Joint Upper Limb	Nociceptive Pain
M84533P	Path fx in neopltc disease, r radius, subs for fx w malunion	Arthritis Joint Upper Limb	Nociceptive Pain
M84533S	Path fracture in neoplastic disease, right radius, sequela	Arthritis Joint Upper Limb	Nociceptive Pain
M84534	Pathological fracture in neoplastic disease, left radius	Arthritis Joint Upper Limb	Nociceptive Pain
M84534A	Path fracture in neoplastic disease, left radius, init	Arthritis Joint Upper Limb	Nociceptive Pain
M84534D	Path fx in neopltc dis, left rad, subs for fx w routn heal	Arthritis Joint Upper Limb	Nociceptive Pain
M84534G	Path fx in neopltc dis, left rad, subs for fx w delay heal	Arthritis Joint Upper Limb	Nociceptive Pain
M84534K	Path fx in neopltc dis, left radius, subs for fx w nonunion	Arthritis Joint Upper Limb	Nociceptive Pain
M84534P	Path fx in neopltc dis, left radius, subs for fx w malunion	Arthritis Joint Upper Limb	Nociceptive Pain
M84534S	Path fracture in neoplastic disease, left radius, sequela	Arthritis Joint Upper Limb	Nociceptive Pain
M84539	Path fracture in neoplastic disease, unsp ulna and radius	Arthritis Joint Upper Limb	Nociceptive Pain
M84539A	Path fracture in neopltc disease, unsp ulna and radius, init	Arthritis Joint Upper Limb	Nociceptive Pain
M84539D	Path fx in neopltc dis, unsp ulna & rad, 7thD	Arthritis Joint Upper Limb	Nociceptive Pain
M84539G	Path fx in neopltc dis, unsp ulna & rad, 7thG	Arthritis Joint Upper Limb	Nociceptive Pain
M84539K	Path fx in neopltc dis, unsp ulna & rad, 7thK	Arthritis Joint Upper Limb	Nociceptive Pain
M84539P	Path fx in neopltc dis, unsp ulna & rad, 7thP	Arthritis Joint Upper Limb	Nociceptive Pain
M84539S	Path fx in neopltc disease, unsp ulna and radius, sequela	Arthritis Joint Upper Limb	Nociceptive Pain
M8454	Pathological fracture in neoplastic disease, hand	Arthritis Joint Upper Limb	Nociceptive Pain
M84541	Pathological fracture in neoplastic disease, right hand	Arthritis Joint Upper Limb	Nociceptive Pain
M84541A	Path fracture in neoplastic disease, right hand, init	Arthritis Joint Upper Limb	Nociceptive Pain
M84541D	Path fx in neopltc disease, r hand, subs for fx w routn heal	Arthritis Joint Upper Limb	Nociceptive Pain

© 2024 Peterson MD et al. *JAMA Neurology.*

M84541G	Path fx in neopltc disease, r hand, subs for fx w delay heal	Arthritis Joint Upper Limb	Nociceptive Pain
M84541K	Path fx in neopltc disease, r hand, subs for fx w nonunion	Arthritis Joint Upper Limb	Nociceptive Pain
M84541P	Path fx in neopltc disease, r hand, subs for fx w malunion	Arthritis Joint Upper Limb	Nociceptive Pain
M84541S	Path fracture in neoplastic disease, right hand, sequela	Arthritis Joint Upper Limb	Nociceptive Pain
M84542	Pathological fracture in neoplastic disease, left hand	Arthritis Joint Upper Limb	Nociceptive Pain
M84542A	Pathological fracture in neoplastic disease, left hand, init	Arthritis Joint Upper Limb	Nociceptive Pain
M84542D	Path fx in neopltc disease, l hand, subs for fx w routn heal	Arthritis Joint Upper Limb	Nociceptive Pain
M84542G	Path fx in neopltc disease, l hand, subs for fx w delay heal	Arthritis Joint Upper Limb	Nociceptive Pain
M84542K	Path fx in neopltc disease, l hand, subs for fx w nonunion	Arthritis Joint Upper Limb	Nociceptive Pain
M84542P	Path fx in neopltc disease, l hand, subs for fx w malunion	Arthritis Joint Upper Limb	Nociceptive Pain
M84542S	Path fracture in neoplastic disease, left hand, sequela	Arthritis Joint Upper Limb	Nociceptive Pain
M84549	Pathological fracture in neoplastic disease, unsp hand	Arthritis Joint Upper Limb	Nociceptive Pain
M84549A	Pathological fracture in neoplastic disease, unsp hand, init	Arthritis Joint Upper Limb	Nociceptive Pain
M84549D	Path fx in neopltc dis, unsp hand, subs for fx w routn heal	Arthritis Joint Upper Limb	Nociceptive Pain
M84549G	Path fx in neopltc dis, unsp hand, subs for fx w delay heal	Arthritis Joint Upper Limb	Nociceptive Pain
M84549K	Path fx in neopltc dis, unsp hand, subs for fx w nonunion	Arthritis Joint Upper Limb	Nociceptive Pain
M84549P	Path fx in neopltc dis, unsp hand, subs for fx w malunion	Arthritis Joint Upper Limb	Nociceptive Pain
M84549S	Path fracture in neoplastic disease, unsp hand, sequela	Arthritis Joint Upper Limb	Nociceptive Pain
M8455	Path fracture in neoplastic disease, pelvis and femur	Arthritis Joint Lower Limb	Nociceptive Pain
M84550	Pathological fracture in neoplastic disease, pelvis	Arthritis Joint Spine and Hips	Nociceptive Pain
M84550A	Pathological fracture in neoplastic disease, pelvis, init	Arthritis Joint Spine and Hips	Nociceptive Pain
M84550D	Path fx in neopltc disease, pelvis, subs for fx w routn heal	Arthritis Joint Spine and Hips	Nociceptive Pain
M84550G	Path fx in neopltc disease, pelvis, subs for fx w delay heal	Arthritis Joint Spine and Hips	Nociceptive Pain
M84550K	Path fx in neopltc disease, pelvis, subs for fx w nonunion	Arthritis Joint Spine and Hips	Nociceptive Pain
M84550P	Path fx in neopltc disease, pelvis, subs for fx w malunion	Arthritis Joint Spine and Hips	Nociceptive Pain
M84550S	Pathological fracture in neoplastic disease, pelvis, sequela	Arthritis Joint Spine and Hips	Nociceptive Pain
M84551	Pathological fracture in neoplastic disease, right femur	Arthritis Joint Lower Limb	Nociceptive Pain
M84551A	Path fracture in neoplastic disease, right femur, init	Arthritis Joint Lower Limb	Nociceptive Pain
M84551D	Path fx in neopltc dis, r femur, subs for fx w routn heal	Arthritis Joint Lower Limb	Nociceptive Pain
M84551G	Path fx in neopltc dis, r femur, subs for fx w delay heal	Arthritis Joint Lower Limb	Nociceptive Pain
M84551K	Path fx in neopltc disease, r femur, subs for fx w nonunion	Arthritis Joint Lower Limb	Nociceptive Pain
M84551P	Path fx in neopltc disease, r femur, subs for fx w malunion	Arthritis Joint Lower Limb	Nociceptive Pain
M84551S	Path fracture in neoplastic disease, right femur, sequela	Arthritis Joint Lower Limb	Nociceptive Pain
M84552	Pathological fracture in neoplastic disease, left femur	Arthritis Joint Lower Limb	Nociceptive Pain
M84552A	Path fracture in neoplastic disease, left femur, init	Arthritis Joint Lower Limb	Nociceptive Pain
M84552D	Path fx in neopltc dis, l femur, subs for fx w routn heal	Arthritis Joint Lower Limb	Nociceptive Pain
M84552G	Path fx in neopltc dis, l femur, subs for fx w delay heal	Arthritis Joint Lower Limb	Nociceptive Pain

© 2024 Peterson MD et al. *JAMA Neurology.*

M84552K	Path fx in neopltc disease, l femur, subs for fx w nonunion	Arthritis Joint Lower Limb	Nociceptive Pain
M84552P	Path fx in neopltc disease, l femur, subs for fx w malunion	Arthritis Joint Lower Limb	Nociceptive Pain
M84552S	Path fracture in neoplastic disease, left femur, sequela	Arthritis Joint Lower Limb	Nociceptive Pain
M84553	Pathological fracture in neoplastic disease, unsp femur	Arthritis Joint Lower Limb	Nociceptive Pain
M84553A	Path fracture in neoplastic disease, unsp femur, init	Arthritis Joint Lower Limb	Nociceptive Pain
M84553D	Path fx in neopltc dis, unsp femur, subs for fx w routn heal	Arthritis Joint Lower Limb	Nociceptive Pain
M84553G	Path fx in neopltc dis, unsp femur, subs for fx w delay heal	Arthritis Joint Lower Limb	Nociceptive Pain
M84553K	Path fx in neopltc dis, unsp femur, subs for fx w nonunion	Arthritis Joint Lower Limb	Nociceptive Pain
M84553P	Path fx in neopltc dis, unsp femur, subs for fx w malunion	Arthritis Joint Lower Limb	Nociceptive Pain
M84553S	Path fracture in neoplastic disease, unsp femur, sequela	Arthritis Joint Lower Limb	Nociceptive Pain
M84559	Pathological fracture in neoplastic disease, hip, unsp	Arthritis Joint Spine and Hips	Nociceptive Pain
M84559A	Pathological fracture in neoplastic disease, hip, unsp, init	Arthritis Joint Spine and Hips	Nociceptive Pain
M84559D	Path fx in neopltc dis, hip, unsp, subs for fx w routn heal	Arthritis Joint Spine and Hips	Nociceptive Pain
M84559G	Path fx in neopltc dis, hip, unsp, subs for fx w delay heal	Arthritis Joint Spine and Hips	Nociceptive Pain
M84559K	Path fx in neopltc dis, hip, unsp, subs for fx w nonunion	Arthritis Joint Spine and Hips	Nociceptive Pain
M84559P	Path fx in neopltc dis, hip, unsp, subs for fx w malunion	Arthritis Joint Spine and Hips	Nociceptive Pain
M84559S	Path fracture in neoplastic disease, hip, unsp, sequela	Arthritis Joint Spine and Hips	Nociceptive Pain
M8456	Path fracture in neoplastic disease, tibia and fibula	Arthritis Joint Lower Limb	Nociceptive Pain
M84561	Pathological fracture in neoplastic disease, right tibia	Arthritis Joint Lower Limb	Nociceptive Pain
M84561A	Path fracture in neoplastic disease, right tibia, init	Arthritis Joint Lower Limb	Nociceptive Pain
M84561D	Path fx in neopltc dis, r tibia, subs for fx w routn heal	Arthritis Joint Lower Limb	Nociceptive Pain
M84561G	Path fx in neopltc dis, r tibia, subs for fx w delay heal	Arthritis Joint Lower Limb	Nociceptive Pain
M84561K	Path fx in neopltc disease, r tibia, subs for fx w nonunion	Arthritis Joint Lower Limb	Nociceptive Pain
M84561P	Path fx in neopltc disease, r tibia, subs for fx w malunion	Arthritis Joint Lower Limb	Nociceptive Pain
M84561S	Path fracture in neoplastic disease, right tibia, sequela	Arthritis Joint Lower Limb	Nociceptive Pain
M84562	Pathological fracture in neoplastic disease, left tibia	Arthritis Joint Lower Limb	Nociceptive Pain
M84562A	Path fracture in neoplastic disease, left tibia, init	Arthritis Joint Lower Limb	Nociceptive Pain
M84562D	Path fx in neopltc dis, l tibia, subs for fx w routn heal	Arthritis Joint Lower Limb	Nociceptive Pain
M84562G	Path fx in neopltc dis, l tibia, subs for fx w delay heal	Arthritis Joint Lower Limb	Nociceptive Pain
M84562K	Path fx in neopltc disease, l tibia, subs for fx w nonunion	Arthritis Joint Lower Limb	Nociceptive Pain
M84562P	Path fx in neopltc disease, l tibia, subs for fx w malunion	Arthritis Joint Lower Limb	Nociceptive Pain
M84562S	Path fracture in neoplastic disease, left tibia, sequela	Arthritis Joint Lower Limb	Nociceptive Pain
M84563	Pathological fracture in neoplastic disease, right fibula	Arthritis Joint Lower Limb	Nociceptive Pain
M84563A	Path fracture in neoplastic disease, right fibula, init	Arthritis Joint Lower Limb	Nociceptive Pain
M84563D	Path fx in neopltc dis, r fibula, subs for fx w routn heal	Arthritis Joint Lower Limb	Nociceptive Pain
M84563G	Path fx in neopltc dis, r fibula, subs for fx w delay heal	Arthritis Joint Lower Limb	Nociceptive Pain
M84563K	Path fx in neopltc disease, r fibula, subs for fx w nonunion	Arthritis Joint Lower Limb	Nociceptive Pain

© 2024 Peterson MD et al. *JAMA Neurology.*

M84563P	Path fx in neopltc disease, r fibula, subs for fx w malunion	Arthritis Joint Lower Limb	Nociceptive Pain
M84563S	Path fracture in neoplastic disease, right fibula, sequela	Arthritis Joint Lower Limb	Nociceptive Pain
M84564	Pathological fracture in neoplastic disease, left fibula	Arthritis Joint Lower Limb	Nociceptive Pain
M84564A	Path fracture in neoplastic disease, left fibula, init	Arthritis Joint Lower Limb	Nociceptive Pain
M84564D	Path fx in neopltc dis, l fibula, subs for fx w routn heal	Arthritis Joint Lower Limb	Nociceptive Pain
M84564G	Path fx in neopltc dis, l fibula, subs for fx w delay heal	Arthritis Joint Lower Limb	Nociceptive Pain
M84564K	Path fx in neopltc disease, l fibula, subs for fx w nonunion	Arthritis Joint Lower Limb	Nociceptive Pain
M84564P	Path fx in neopltc disease, l fibula, subs for fx w malunion	Arthritis Joint Lower Limb	Nociceptive Pain
M84564S	Path fracture in neoplastic disease, left fibula, sequela	Arthritis Joint Lower Limb	Nociceptive Pain
M84569	Path fracture in neoplastic disease, unsp tibia and fibula	Arthritis Joint Lower Limb	Nociceptive Pain
M84569A	Path fx in neopltc disease, unsp tibia and fibula, init	Arthritis Joint Lower Limb	Nociceptive Pain
M84569D	Path fx in neopltc dis, unsp tibia & fibula, 7thD	Arthritis Joint Lower Limb	Nociceptive Pain
M84569G	Path fx in neopltc dis, unsp tibia & fibula, 7thG	Arthritis Joint Lower Limb	Nociceptive Pain
M84569K	Path fx in neopltc dis, unsp tibia & fibula, 7thK	Arthritis Joint Lower Limb	Nociceptive Pain
M84569P	Path fx in neopltc dis, unsp tibia & fibula, 7thP	Arthritis Joint Lower Limb	Nociceptive Pain
M84569S	Path fx in neopltc disease, unsp tibia and fibula, sequela	Arthritis Joint Lower Limb	Nociceptive Pain
M8457	Pathological fracture in neoplastic disease, ankle and foot	Arthritis Joint Lower Limb	Nociceptive Pain
M84571	Pathological fracture in neoplastic disease, right ankle	Arthritis Joint Lower Limb	Nociceptive Pain
M84571A	Path fracture in neoplastic disease, right ankle, init	Arthritis Joint Lower Limb	Nociceptive Pain
M84571D	Path fx in neopltc dis, r ankle, subs for fx w routn heal	Arthritis Joint Lower Limb	Nociceptive Pain
M84571G	Path fx in neopltc dis, r ankle, subs for fx w delay heal	Arthritis Joint Lower Limb	Nociceptive Pain
M84571K	Path fx in neopltc disease, r ankle, subs for fx w nonunion	Arthritis Joint Lower Limb	Nociceptive Pain
M84571P	Path fx in neopltc disease, r ankle, subs for fx w malunion	Arthritis Joint Lower Limb	Nociceptive Pain
M84571S	Path fracture in neoplastic disease, right ankle, sequela	Arthritis Joint Lower Limb	Nociceptive Pain
M84572	Pathological fracture in neoplastic disease, left ankle	Arthritis Joint Lower Limb	Nociceptive Pain
M84572A	Path fracture in neoplastic disease, left ankle, init	Arthritis Joint Lower Limb	Nociceptive Pain
M84572D	Path fx in neopltc dis, l ankle, subs for fx w routn heal	Arthritis Joint Lower Limb	Nociceptive Pain
M84572G	Path fx in neopltc dis, l ankle, subs for fx w delay heal	Arthritis Joint Lower Limb	Nociceptive Pain
M84572K	Path fx in neopltc disease, l ankle, subs for fx w nonunion	Arthritis Joint Lower Limb	Nociceptive Pain
M84572P	Path fx in neopltc disease, l ankle, subs for fx w malunion	Arthritis Joint Lower Limb	Nociceptive Pain
M84572S	Path fracture in neoplastic disease, left ankle, sequela	Arthritis Joint Lower Limb	Nociceptive Pain
M84573	Pathological fracture in neoplastic disease, unsp ankle	Arthritis Joint Lower Limb	Nociceptive Pain
M84573A	Path fracture in neoplastic disease, unsp ankle, init	Arthritis Joint Lower Limb	Nociceptive Pain
M84573D	Path fx in neopltc dis, unsp ankle, subs for fx w routn heal	Arthritis Joint Lower Limb	Nociceptive Pain
M84573G	Path fx in neopltc dis, unsp ankle, subs for fx w delay heal	Arthritis Joint Lower Limb	Nociceptive Pain
M84573K	Path fx in neopltc dis, unsp ankle, subs for fx w nonunion	Arthritis Joint Lower Limb	Nociceptive Pain
M84573P	Path fx in neopltc dis, unsp ankle, subs for fx w malunion	Arthritis Joint Lower Limb	Nociceptive Pain

© 2024 Peterson MD et al. *JAMA Neurology.*

M84573S	Path fracture in neoplastic disease, unsp ankle, sequela	Arthritis Joint Lower Limb	Nociceptive Pain
M84574	Pathological fracture in neoplastic disease, right foot	Arthritis Joint Lower Limb	Nociceptive Pain
M84574A	Path fracture in neoplastic disease, right foot, init	Arthritis Joint Lower Limb	Nociceptive Pain
M84574D	Path fx in neopltc disease, r foot, subs for fx w routn heal	Arthritis Joint Lower Limb	Nociceptive Pain
M84574G	Path fx in neopltc disease, r foot, subs for fx w delay heal	Arthritis Joint Lower Limb	Nociceptive Pain
M84574K	Path fx in neopltc disease, r foot, subs for fx w nonunion	Arthritis Joint Lower Limb	Nociceptive Pain
M84574P	Path fx in neopltc disease, r foot, subs for fx w malunion	Arthritis Joint Lower Limb	Nociceptive Pain
M84574S	Path fracture in neoplastic disease, right foot, sequela	Arthritis Joint Lower Limb	Nociceptive Pain
M84575	Pathological fracture in neoplastic disease, left foot	Arthritis Joint Lower Limb	Nociceptive Pain
M84575A	Pathological fracture in neoplastic disease, left foot, init	Arthritis Joint Lower Limb	Nociceptive Pain
M84575D	Path fx in neopltc disease, l foot, subs for fx w routn heal	Arthritis Joint Lower Limb	Nociceptive Pain
M84575G	Path fx in neopltc disease, l foot, subs for fx w delay heal	Arthritis Joint Lower Limb	Nociceptive Pain
M84575K	Path fx in neopltc disease, l foot, subs for fx w nonunion	Arthritis Joint Lower Limb	Nociceptive Pain
M84575P	Path fx in neopltc disease, l foot, subs for fx w malunion	Arthritis Joint Lower Limb	Nociceptive Pain
M84575S	Path fracture in neoplastic disease, left foot, sequela	Arthritis Joint Lower Limb	Nociceptive Pain
M84576	Pathological fracture in neoplastic disease, unsp foot	Arthritis Joint Lower Limb	Nociceptive Pain
M84576A	Pathological fracture in neoplastic disease, unsp foot, init	Arthritis Joint Lower Limb	Nociceptive Pain
M84576D	Path fx in neopltc dis, unsp foot, subs for fx w routn heal	Arthritis Joint Lower Limb	Nociceptive Pain
M84576G	Path fx in neopltc dis, unsp foot, subs for fx w delay heal	Arthritis Joint Lower Limb	Nociceptive Pain
M84576K	Path fx in neopltc dis, unsp foot, subs for fx w nonunion	Arthritis Joint Lower Limb	Nociceptive Pain
M84576P	Path fx in neopltc dis, unsp foot, subs for fx w malunion	Arthritis Joint Lower Limb	Nociceptive Pain
M84576S	Path fracture in neoplastic disease, unsp foot, sequela	Arthritis Joint Lower Limb	Nociceptive Pain
M8458	Pathological fracture in neoplastic disease, oth site	Arthritis Joint Other	Nociceptive Pain
M8458XA	Pathological fracture in neoplastic disease, oth site, init	Arthritis Joint Other	Nociceptive Pain
M8458XD	Path fx in neopltc dis, oth site, subs for fx w routn heal	Arthritis Joint Other	Nociceptive Pain
M8458XG	Path fx in neopltc dis, oth site, subs for fx w delay heal	Arthritis Joint Other	Nociceptive Pain
M8458XK	Path fx in neopltc disease, oth site, subs for fx w nonunion	Arthritis Joint Other	Nociceptive Pain
M8458XP	Path fx in neopltc disease, oth site, subs for fx w malunion	Arthritis Joint Other	Nociceptive Pain
M8458XS	Path fracture in neoplastic disease, oth site, sequela	Arthritis Joint Other	Nociceptive Pain
M846	Pathological fracture in other disease	Arthritis Joint Other	Nociceptive Pain
M8460	Pathological fracture in other disease, unspecified site	Arthritis Joint Other	Nociceptive Pain
M8460XA	Pathological fracture in oth disease, unsp site, init for fx	Arthritis Joint Other	Nociceptive Pain
M8460XD	Path fx in oth disease, unsp site, subs for fx w routn heal	Arthritis Joint Other	Nociceptive Pain
M8460XG	Path fx in oth disease, unsp site, subs for fx w delay heal	Arthritis Joint Other	Nociceptive Pain
M8460XK	Path fx in oth disease, unsp site, subs for fx w nonunion	Arthritis Joint Other	Nociceptive Pain
M8460XP	Path fx in oth disease, unsp site, subs for fx w malunion	Arthritis Joint Other	Nociceptive Pain
M8460XS	Pathological fracture in other disease, unsp site, sequela	Arthritis Joint Other	Nociceptive Pain

© 2024 Peterson MD et al. *JAMA Neurology.*

M8461	Pathological fracture in other disease, shoulder	Arthritis Joint Upper Limb	Nociceptive Pain
M84611	Pathological fracture in other disease, right shoulder	Arthritis Joint Upper Limb	Nociceptive Pain
M84611A	Pathological fracture in oth disease, right shoulder, init	Arthritis Joint Upper Limb	Nociceptive Pain
M84611D	Path fx in oth disease, r shoulder, subs for fx w routn heal	Arthritis Joint Upper Limb	Nociceptive Pain
M84611G	Path fx in oth disease, r shoulder, subs for fx w delay heal	Arthritis Joint Upper Limb	Nociceptive Pain
M84611K	Path fx in oth disease, r shoulder, subs for fx w nonunion	Arthritis Joint Upper Limb	Nociceptive Pain
M84611P	Path fx in oth disease, r shoulder, subs for fx w malunion	Arthritis Joint Upper Limb	Nociceptive Pain
M84611S	Pathological fracture in oth disease, r shoulder, sequela	Arthritis Joint Upper Limb	Nociceptive Pain
M84612	Pathological fracture in other disease, left shoulder	Arthritis Joint Upper Limb	Nociceptive Pain
M84612A	Pathological fracture in oth disease, left shoulder, init	Arthritis Joint Upper Limb	Nociceptive Pain
M84612D	Path fx in oth disease, l shoulder, subs for fx w routn heal	Arthritis Joint Upper Limb	Nociceptive Pain
M84612G	Path fx in oth disease, l shoulder, subs for fx w delay heal	Arthritis Joint Upper Limb	Nociceptive Pain
M84612K	Path fx in oth disease, l shoulder, subs for fx w nonunion	Arthritis Joint Upper Limb	Nociceptive Pain
M84612P	Path fx in oth disease, l shoulder, subs for fx w malunion	Arthritis Joint Upper Limb	Nociceptive Pain
M84612S	Pathological fracture in oth disease, left shoulder, sequela	Arthritis Joint Upper Limb	Nociceptive Pain
M84619	Pathological fracture in other disease, unspecified shoulder	Arthritis Joint Upper Limb	Nociceptive Pain
M84619A	Pathological fracture in oth disease, unsp shoulder, init	Arthritis Joint Upper Limb	Nociceptive Pain
M84619D	Path fx in oth disease, unsp shldr, subs for fx w routn heal	Arthritis Joint Upper Limb	Nociceptive Pain
M84619G	Path fx in oth disease, unsp shldr, subs for fx w delay heal	Arthritis Joint Upper Limb	Nociceptive Pain
M84619K	Path fx in oth disease, unsp shldr, subs for fx w nonunion	Arthritis Joint Upper Limb	Nociceptive Pain
M84619P	Path fx in oth disease, unsp shldr, subs for fx w malunion	Arthritis Joint Upper Limb	Nociceptive Pain
M84619S	Pathological fracture in oth disease, unsp shoulder, sequela	Arthritis Joint Upper Limb	Nociceptive Pain
M8462	Pathological fracture in other disease, humerus	Arthritis Joint Upper Limb	Nociceptive Pain
M84621	Pathological fracture in other disease, right humerus	Arthritis Joint Upper Limb	Nociceptive Pain
M84621A	Pathological fracture in oth disease, right humerus, init	Arthritis Joint Upper Limb	Nociceptive Pain
M84621D	Path fx in oth disease, r humerus, subs for fx w routn heal	Arthritis Joint Upper Limb	Nociceptive Pain
M84621G	Path fx in oth disease, r humerus, subs for fx w delay heal	Arthritis Joint Upper Limb	Nociceptive Pain
M84621K	Path fx in oth disease, r humerus, subs for fx w nonunion	Arthritis Joint Upper Limb	Nociceptive Pain
M84621P	Path fx in oth disease, r humerus, subs for fx w malunion	Arthritis Joint Upper Limb	Nociceptive Pain
M84621S	Pathological fracture in oth disease, right humerus, sequela	Arthritis Joint Upper Limb	Nociceptive Pain
M84622	Pathological fracture in other disease, left humerus	Arthritis Joint Upper Limb	Nociceptive Pain
M84622A	Pathological fracture in oth disease, left humerus, init	Arthritis Joint Upper Limb	Nociceptive Pain
M84622D	Path fx in oth disease, l humerus, subs for fx w routn heal	Arthritis Joint Upper Limb	Nociceptive Pain
M84622G	Path fx in oth disease, l humerus, subs for fx w delay heal	Arthritis Joint Upper Limb	Nociceptive Pain
M84622K	Path fx in oth disease, l humerus, subs for fx w nonunion	Arthritis Joint Upper Limb	Nociceptive Pain
M84622P	Path fx in oth disease, l humerus, subs for fx w malunion	Arthritis Joint Upper Limb	Nociceptive Pain
M84622S	Pathological fracture in oth disease, left humerus, sequela	Arthritis Joint Upper Limb	Nociceptive Pain

© 2024 Peterson MD et al. *JAMA Neurology.*

M84629	Pathological fracture in other disease, unspecified humerus	Arthritis Joint Upper Limb	Nociceptive Pain
M84629A	Pathological fracture in oth disease, unsp humerus, init	Arthritis Joint Upper Limb	Nociceptive Pain
M84629D	Path fx in oth dis, unsp humerus, subs for fx w routn heal	Arthritis Joint Upper Limb	Nociceptive Pain
M84629G	Path fx in oth dis, unsp humerus, subs for fx w delay heal	Arthritis Joint Upper Limb	Nociceptive Pain
M84629K	Path fx in oth disease, unsp humerus, subs for fx w nonunion	Arthritis Joint Upper Limb	Nociceptive Pain
M84629P	Path fx in oth disease, unsp humerus, subs for fx w malunion	Arthritis Joint Upper Limb	Nociceptive Pain
M84629S	Pathological fracture in oth disease, unsp humerus, sequela	Arthritis Joint Upper Limb	Nociceptive Pain
M8463	Pathological fracture in other disease, ulna and radius	Arthritis Joint Upper Limb	Nociceptive Pain
M84631	Pathological fracture in other disease, right ulna	Arthritis Joint Upper Limb	Nociceptive Pain
M84631A	Pathological fracture in oth disease, right ulna, init	Arthritis Joint Upper Limb	Nociceptive Pain
M84631D	Path fx in oth disease, r ulna, subs for fx w routn heal	Arthritis Joint Upper Limb	Nociceptive Pain
M84631G	Path fx in oth disease, r ulna, subs for fx w delay heal	Arthritis Joint Upper Limb	Nociceptive Pain
M84631K	Path fracture in oth disease, r ulna, subs for fx w nonunion	Arthritis Joint Upper Limb	Nociceptive Pain
M84631P	Path fracture in oth disease, r ulna, subs for fx w malunion	Arthritis Joint Upper Limb	Nociceptive Pain
M84631S	Pathological fracture in other disease, right ulna, sequela	Arthritis Joint Upper Limb	Nociceptive Pain
M84632	Pathological fracture in other disease, left ulna	Arthritis Joint Upper Limb	Nociceptive Pain
M84632A	Pathological fracture in oth disease, left ulna, init for fx	Arthritis Joint Upper Limb	Nociceptive Pain
M84632D	Path fx in oth disease, l ulna, subs for fx w routn heal	Arthritis Joint Upper Limb	Nociceptive Pain
M84632G	Path fx in oth disease, l ulna, subs for fx w delay heal	Arthritis Joint Upper Limb	Nociceptive Pain
M84632K	Path fracture in oth disease, l ulna, subs for fx w nonunion	Arthritis Joint Upper Limb	Nociceptive Pain
M84632P	Path fracture in oth disease, l ulna, subs for fx w malunion	Arthritis Joint Upper Limb	Nociceptive Pain
M84632S	Pathological fracture in other disease, left ulna, sequela	Arthritis Joint Upper Limb	Nociceptive Pain
M84633	Pathological fracture in other disease, right radius	Arthritis Joint Upper Limb	Nociceptive Pain
M84633A	Pathological fracture in oth disease, right radius, init	Arthritis Joint Upper Limb	Nociceptive Pain
M84633D	Path fx in oth disease, r radius, subs for fx w routn heal	Arthritis Joint Upper Limb	Nociceptive Pain
M84633G	Path fx in oth disease, r radius, subs for fx w delay heal	Arthritis Joint Upper Limb	Nociceptive Pain
M84633K	Path fx in oth disease, r radius, subs for fx w nonunion	Arthritis Joint Upper Limb	Nociceptive Pain
M84633P	Path fx in oth disease, r radius, subs for fx w malunion	Arthritis Joint Upper Limb	Nociceptive Pain
M84633S	Pathological fracture in oth disease, right radius, sequela	Arthritis Joint Upper Limb	Nociceptive Pain
M84634	Pathological fracture in other disease, left radius	Arthritis Joint Upper Limb	Nociceptive Pain
M84634A	Pathological fracture in oth disease, left radius, init	Arthritis Joint Upper Limb	Nociceptive Pain
M84634D	Path fx in oth dis, left radius, subs for fx w routn heal	Arthritis Joint Upper Limb	Nociceptive Pain
M84634G	Path fx in oth dis, left radius, subs for fx w delay heal	Arthritis Joint Upper Limb	Nociceptive Pain
M84634K	Path fx in oth disease, left radius, subs for fx w nonunion	Arthritis Joint Upper Limb	Nociceptive Pain
M84634P	Path fx in oth disease, left radius, subs for fx w malunion	Arthritis Joint Upper Limb	Nociceptive Pain
M84634S	Pathological fracture in other disease, left radius, sequela	Arthritis Joint Upper Limb	Nociceptive Pain
M84639	Pathological fracture in other disease, unsp ulna and radius	Arthritis Joint Upper Limb	Nociceptive Pain

© 2024 Peterson MD et al. *JAMA Neurology.*

M84639A	Path fracture in oth disease, unsp ulna and radius, init	Arthritis Joint Upper Limb	Nociceptive Pain
M84639D	Path fx in oth dis, unsp ulna & rad, 7thD	Arthritis Joint Upper Limb	Nociceptive Pain
M84639G	Path fx in oth dis, unsp ulna & rad, 7thG	Arthritis Joint Upper Limb	Nociceptive Pain
M84639K	Path fx in oth dis, unsp ulna & rad, subs for fx w nonunion	Arthritis Joint Upper Limb	Nociceptive Pain
M84639P	Path fx in oth dis, unsp ulna & rad, subs for fx w malunion	Arthritis Joint Upper Limb	Nociceptive Pain
M84639S	Path fracture in oth disease, unsp ulna and radius, sequela	Arthritis Joint Upper Limb	Nociceptive Pain
M8464	Pathological fracture in other disease, hand	Arthritis Joint Upper Limb	Nociceptive Pain
M84641	Pathological fracture in other disease, right hand	Arthritis Joint Upper Limb	Nociceptive Pain
M84641A	Pathological fracture in oth disease, right hand, init	Arthritis Joint Upper Limb	Nociceptive Pain
M84641D	Path fx in oth disease, r hand, subs for fx w routn heal	Arthritis Joint Upper Limb	Nociceptive Pain
M84641G	Path fx in oth disease, r hand, subs for fx w delay heal	Arthritis Joint Upper Limb	Nociceptive Pain
M84641K	Path fracture in oth disease, r hand, subs for fx w nonunion	Arthritis Joint Upper Limb	Nociceptive Pain
M84641P	Path fracture in oth disease, r hand, subs for fx w malunion	Arthritis Joint Upper Limb	Nociceptive Pain
M84641S	Pathological fracture in other disease, right hand, sequela	Arthritis Joint Upper Limb	Nociceptive Pain
M84642	Pathological fracture in other disease, left hand	Arthritis Joint Upper Limb	Nociceptive Pain
M84642A	Pathological fracture in oth disease, left hand, init for fx	Arthritis Joint Upper Limb	Nociceptive Pain
M84642D	Path fx in oth disease, l hand, subs for fx w routn heal	Arthritis Joint Upper Limb	Nociceptive Pain
M84642G	Path fx in oth disease, l hand, subs for fx w delay heal	Arthritis Joint Upper Limb	Nociceptive Pain
M84642K	Path fracture in oth disease, l hand, subs for fx w nonunion	Arthritis Joint Upper Limb	Nociceptive Pain
M84642P	Path fracture in oth disease, l hand, subs for fx w malunion	Arthritis Joint Upper Limb	Nociceptive Pain
M84642S	Pathological fracture in other disease, left hand, sequela	Arthritis Joint Upper Limb	Nociceptive Pain
M84649	Pathological fracture in other disease, unspecified hand	Arthritis Joint Upper Limb	Nociceptive Pain
M84649A	Pathological fracture in oth disease, unsp hand, init for fx	Arthritis Joint Upper Limb	Nociceptive Pain
M84649D	Path fx in oth disease, unsp hand, subs for fx w routn heal	Arthritis Joint Upper Limb	Nociceptive Pain
M84649G	Path fx in oth disease, unsp hand, subs for fx w delay heal	Arthritis Joint Upper Limb	Nociceptive Pain
M84649K	Path fx in oth disease, unsp hand, subs for fx w nonunion	Arthritis Joint Upper Limb	Nociceptive Pain
M84649P	Path fx in oth disease, unsp hand, subs for fx w malunion	Arthritis Joint Upper Limb	Nociceptive Pain
M84649S	Pathological fracture in other disease, unsp hand, sequela	Arthritis Joint Upper Limb	Nociceptive Pain
M8465	Pathological fracture in other disease, pelvis and femur	Arthritis Joint Lower Limb	Nociceptive Pain
M84650	Pathological fracture in other disease, pelvis	Arthritis Joint Spine and Hips	Nociceptive Pain
M84650A	Pathological fracture in oth disease, pelvis, init for fx	Arthritis Joint Spine and Hips	Nociceptive Pain
M84650D	Path fx in oth disease, pelvis, subs for fx w routn heal	Arthritis Joint Spine and Hips	Nociceptive Pain
M84650G	Path fx in oth disease, pelvis, subs for fx w delay heal	Arthritis Joint Spine and Hips	Nociceptive Pain
M84650K	Path fracture in oth disease, pelvis, subs for fx w nonunion	Arthritis Joint Spine and Hips	Nociceptive Pain
M84650P	Path fracture in oth disease, pelvis, subs for fx w malunion	Arthritis Joint Spine and Hips	Nociceptive Pain
M84650S	Pathological fracture in other disease, pelvis, sequela	Arthritis Joint Spine and Hips	Nociceptive Pain
M84651	Pathological fracture in other disease, right femur	Arthritis Joint Lower Limb	Nociceptive Pain

© 2024 Peterson MD et al. *JAMA Neurology.*

M84651A	Pathological fracture in oth disease, right femur, init	Arthritis Joint Lower Limb	Nociceptive Pain
M84651D	Path fx in oth disease, r femur, subs for fx w routn heal	Arthritis Joint Lower Limb	Nociceptive Pain
M84651G	Path fx in oth disease, r femur, subs for fx w delay heal	Arthritis Joint Lower Limb	Nociceptive Pain
M84651K	Path fx in oth disease, r femur, subs for fx w nonunion	Arthritis Joint Lower Limb	Nociceptive Pain
M84651P	Path fx in oth disease, r femur, subs for fx w malunion	Arthritis Joint Lower Limb	Nociceptive Pain
M84651S	Pathological fracture in other disease, right femur, sequela	Arthritis Joint Lower Limb	Nociceptive Pain
M84652	Pathological fracture in other disease, left femur	Arthritis Joint Lower Limb	Nociceptive Pain
M84652A	Pathological fracture in oth disease, left femur, init	Arthritis Joint Lower Limb	Nociceptive Pain
M84652D	Path fx in oth disease, l femur, subs for fx w routn heal	Arthritis Joint Lower Limb	Nociceptive Pain
M84652G	Path fx in oth disease, l femur, subs for fx w delay heal	Arthritis Joint Lower Limb	Nociceptive Pain
M84652K	Path fx in oth disease, l femur, subs for fx w nonunion	Arthritis Joint Lower Limb	Nociceptive Pain
M84652P	Path fx in oth disease, l femur, subs for fx w malunion	Arthritis Joint Lower Limb	Nociceptive Pain
M84652S	Pathological fracture in other disease, left femur, sequela	Arthritis Joint Lower Limb	Nociceptive Pain
M84653	Pathological fracture in other disease, unspecified femur	Arthritis Joint Lower Limb	Nociceptive Pain
M84653A	Pathological fracture in oth disease, unsp femur, init	Arthritis Joint Lower Limb	Nociceptive Pain
M84653D	Path fx in oth disease, unsp femur, subs for fx w routn heal	Arthritis Joint Lower Limb	Nociceptive Pain
M84653G	Path fx in oth disease, unsp femur, subs for fx w delay heal	Arthritis Joint Lower Limb	Nociceptive Pain
M84653K	Path fx in oth disease, unsp femur, subs for fx w nonunion	Arthritis Joint Lower Limb	Nociceptive Pain
M84653P	Path fx in oth disease, unsp femur, subs for fx w malunion	Arthritis Joint Lower Limb	Nociceptive Pain
M84653S	Pathological fracture in other disease, unsp femur, sequela	Arthritis Joint Lower Limb	Nociceptive Pain
M84659	Pathological fracture in other disease, hip, unspecified	Arthritis Joint Spine and Hips	Nociceptive Pain
M84659A	Pathological fracture in oth disease, hip, unsp, init for fx	Arthritis Joint Spine and Hips	Nociceptive Pain
M84659D	Path fx in oth disease, hip, unsp, subs for fx w routn heal	Arthritis Joint Spine and Hips	Nociceptive Pain
M84659G	Path fx in oth disease, hip, unsp, subs for fx w delay heal	Arthritis Joint Spine and Hips	Nociceptive Pain
M84659K	Path fx in oth disease, hip, unsp, subs for fx w nonunion	Arthritis Joint Spine and Hips	Nociceptive Pain
M84659P	Path fx in oth disease, hip, unsp, subs for fx w malunion	Arthritis Joint Spine and Hips	Nociceptive Pain
M84659S	Pathological fracture in other disease, hip, unsp, sequela	Arthritis Joint Spine and Hips	Nociceptive Pain
M8466	Pathological fracture in other disease, tibia and fibula	Arthritis Joint Lower Limb	Nociceptive Pain
M84661	Pathological fracture in other disease, right tibia	Arthritis Joint Lower Limb	Nociceptive Pain
M84661A	Pathological fracture in oth disease, right tibia, init	Arthritis Joint Lower Limb	Nociceptive Pain
M84661D	Path fx in oth disease, r tibia, subs for fx w routn heal	Arthritis Joint Lower Limb	Nociceptive Pain
M84661G	Path fx in oth disease, r tibia, subs for fx w delay heal	Arthritis Joint Lower Limb	Nociceptive Pain
M84661K	Path fx in oth disease, r tibia, subs for fx w nonunion	Arthritis Joint Lower Limb	Nociceptive Pain
M84661P	Path fx in oth disease, r tibia, subs for fx w malunion	Arthritis Joint Lower Limb	Nociceptive Pain
M84661S	Pathological fracture in other disease, right tibia, sequela	Arthritis Joint Lower Limb	Nociceptive Pain
M84662	Pathological fracture in other disease, left tibia	Arthritis Joint Lower Limb	Nociceptive Pain
M84662A	Pathological fracture in oth disease, left tibia, init	Arthritis Joint Lower Limb	Nociceptive Pain

© 2024 Peterson MD et al. *JAMA Neurology.*

M84662D	Path fx in oth disease, l tibia, subs for fx w routn heal	Arthritis Joint Lower Limb	Nociceptive Pain
M84662G	Path fx in oth disease, l tibia, subs for fx w delay heal	Arthritis Joint Lower Limb	Nociceptive Pain
M84662K	Path fx in oth disease, l tibia, subs for fx w nonunion	Arthritis Joint Lower Limb	Nociceptive Pain
M84662P	Path fx in oth disease, l tibia, subs for fx w malunion	Arthritis Joint Lower Limb	Nociceptive Pain
M84662S	Pathological fracture in other disease, left tibia, sequela	Arthritis Joint Lower Limb	Nociceptive Pain
M84663	Pathological fracture in other disease, right fibula	Arthritis Joint Lower Limb	Nociceptive Pain
M84663A	Pathological fracture in oth disease, right fibula, init	Arthritis Joint Lower Limb	Nociceptive Pain
M84663D	Path fx in oth disease, r fibula, subs for fx w routn heal	Arthritis Joint Lower Limb	Nociceptive Pain
M84663G	Path fx in oth disease, r fibula, subs for fx w delay heal	Arthritis Joint Lower Limb	Nociceptive Pain
M84663K	Path fx in oth disease, r fibula, subs for fx w nonunion	Arthritis Joint Lower Limb	Nociceptive Pain
M84663P	Path fx in oth disease, r fibula, subs for fx w malunion	Arthritis Joint Lower Limb	Nociceptive Pain
M84663S	Pathological fracture in oth disease, right fibula, sequela	Arthritis Joint Lower Limb	Nociceptive Pain
M84664	Pathological fracture in other disease, left fibula	Arthritis Joint Lower Limb	Nociceptive Pain
M84664A	Pathological fracture in oth disease, left fibula, init	Arthritis Joint Lower Limb	Nociceptive Pain
M84664D	Path fx in oth disease, l fibula, subs for fx w routn heal	Arthritis Joint Lower Limb	Nociceptive Pain
M84664G	Path fx in oth disease, l fibula, subs for fx w delay heal	Arthritis Joint Lower Limb	Nociceptive Pain
M84664K	Path fx in oth disease, l fibula, subs for fx w nonunion	Arthritis Joint Lower Limb	Nociceptive Pain
M84664P	Path fx in oth disease, l fibula, subs for fx w malunion	Arthritis Joint Lower Limb	Nociceptive Pain
M84664S	Pathological fracture in other disease, left fibula, sequela	Arthritis Joint Lower Limb	Nociceptive Pain
M84669	Pathological fracture in oth disease, unsp tibia and fibula	Arthritis Joint Lower Limb	Nociceptive Pain
M84669A	Path fracture in oth disease, unsp tibia and fibula, init	Arthritis Joint Lower Limb	Nociceptive Pain
M84669D	Path fx in oth dis, unsp tibia & fibula, 7thD	Arthritis Joint Lower Limb	Nociceptive Pain
M84669G	Path fx in oth dis, unsp tibia & fibula, 7thG	Arthritis Joint Lower Limb	Nociceptive Pain
M84669K	Path fx in oth dis, unsp tibia & fibula, 7thK	Arthritis Joint Lower Limb	Nociceptive Pain
M84669P	Path fx in oth dis, unsp tibia & fibula, 7thP	Arthritis Joint Lower Limb	Nociceptive Pain
M84669S	Path fracture in oth disease, unsp tibia and fibula, sequela	Arthritis Joint Lower Limb	Nociceptive Pain
M8467	Pathological fracture in other disease, ankle and foot	Arthritis Joint Lower Limb	Nociceptive Pain
M84671	Pathological fracture in other disease, right ankle	Arthritis Joint Lower Limb	Nociceptive Pain
M84671A	Pathological fracture in oth disease, right ankle, init	Arthritis Joint Lower Limb	Nociceptive Pain
M84671D	Path fx in oth disease, r ankle, subs for fx w routn heal	Arthritis Joint Lower Limb	Nociceptive Pain
M84671G	Path fx in oth disease, r ankle, subs for fx w delay heal	Arthritis Joint Lower Limb	Nociceptive Pain
M84671K	Path fx in oth disease, r ankle, subs for fx w nonunion	Arthritis Joint Lower Limb	Nociceptive Pain
M84671P	Path fx in oth disease, r ankle, subs for fx w malunion	Arthritis Joint Lower Limb	Nociceptive Pain
M84671S	Pathological fracture in other disease, right ankle, sequela	Arthritis Joint Lower Limb	Nociceptive Pain
M84672	Pathological fracture in other disease, left ankle	Arthritis Joint Lower Limb	Nociceptive Pain
M84672A	Pathological fracture in oth disease, left ankle, init	Arthritis Joint Lower Limb	Nociceptive Pain
M84672D	Path fx in oth disease, l ankle, subs for fx w routn heal	Arthritis Joint Lower Limb	Nociceptive Pain

© 2024 Peterson MD et al. *JAMA Neurology.*

M84672G	Path fx in oth disease, l ankle, subs for fx w delay heal	Arthritis Joint Lower Limb	Nociceptive Pain
M84672K	Path fx in oth disease, l ankle, subs for fx w nonunion	Arthritis Joint Lower Limb	Nociceptive Pain
M84672P	Path fx in oth disease, l ankle, subs for fx w malunion	Arthritis Joint Lower Limb	Nociceptive Pain
M84672S	Pathological fracture in other disease, left ankle, sequela	Arthritis Joint Lower Limb	Nociceptive Pain
M84673	Pathological fracture in other disease, unspecified ankle	Arthritis Joint Lower Limb	Nociceptive Pain
M84673A	Pathological fracture in oth disease, unsp ankle, init	Arthritis Joint Lower Limb	Nociceptive Pain
M84673D	Path fx in oth disease, unsp ankle, subs for fx w routn heal	Arthritis Joint Lower Limb	Nociceptive Pain
M84673G	Path fx in oth disease, unsp ankle, subs for fx w delay heal	Arthritis Joint Lower Limb	Nociceptive Pain
M84673K	Path fx in oth disease, unsp ankle, subs for fx w nonunion	Arthritis Joint Lower Limb	Nociceptive Pain
M84673P	Path fx in oth disease, unsp ankle, subs for fx w malunion	Arthritis Joint Lower Limb	Nociceptive Pain
M84673S	Pathological fracture in other disease, unsp ankle, sequela	Arthritis Joint Lower Limb	Nociceptive Pain
M84674	Pathological fracture in other disease, right foot	Arthritis Joint Lower Limb	Nociceptive Pain
M84674A	Pathological fracture in oth disease, right foot, init	Arthritis Joint Lower Limb	Nociceptive Pain
M84674D	Path fx in oth disease, r foot, subs for fx w routn heal	Arthritis Joint Lower Limb	Nociceptive Pain
M84674G	Path fx in oth disease, r foot, subs for fx w delay heal	Arthritis Joint Lower Limb	Nociceptive Pain
M84674K	Path fracture in oth disease, r foot, subs for fx w nonunion	Arthritis Joint Lower Limb	Nociceptive Pain
M84674P	Path fracture in oth disease, r foot, subs for fx w malunion	Arthritis Joint Lower Limb	Nociceptive Pain
M84674S	Pathological fracture in other disease, right foot, sequela	Arthritis Joint Lower Limb	Nociceptive Pain
M84675	Pathological fracture in other disease, left foot	Arthritis Joint Lower Limb	Nociceptive Pain
M84675A	Pathological fracture in oth disease, left foot, init for fx	Arthritis Joint Lower Limb	Nociceptive Pain
M84675D	Path fx in oth disease, l foot, subs for fx w routn heal	Arthritis Joint Lower Limb	Nociceptive Pain
M84675G	Path fx in oth disease, l foot, subs for fx w delay heal	Arthritis Joint Lower Limb	Nociceptive Pain
M84675K	Path fracture in oth disease, l foot, subs for fx w nonunion	Arthritis Joint Lower Limb	Nociceptive Pain
M84675P	Path fracture in oth disease, l foot, subs for fx w malunion	Arthritis Joint Lower Limb	Nociceptive Pain
M84675S	Pathological fracture in other disease, left foot, sequela	Arthritis Joint Lower Limb	Nociceptive Pain
M84676	Pathological fracture in other disease, unspecified foot	Arthritis Joint Lower Limb	Nociceptive Pain
M84676A	Pathological fracture in oth disease, unsp foot, init for fx	Arthritis Joint Lower Limb	Nociceptive Pain
M84676D	Path fx in oth disease, unsp foot, subs for fx w routn heal	Arthritis Joint Lower Limb	Nociceptive Pain
M84676G	Path fx in oth disease, unsp foot, subs for fx w delay heal	Arthritis Joint Lower Limb	Nociceptive Pain
M84676K	Path fx in oth disease, unsp foot, subs for fx w nonunion	Arthritis Joint Lower Limb	Nociceptive Pain
M84676P	Path fx in oth disease, unsp foot, subs for fx w malunion	Arthritis Joint Lower Limb	Nociceptive Pain
M84676S	Pathological fracture in other disease, unsp foot, sequela	Arthritis Joint Lower Limb	Nociceptive Pain
M8468	Pathological fracture in other disease, other site	Arthritis Joint Other	Nociceptive Pain
M8468XA	Pathological fracture in oth disease, oth site, init for fx	Arthritis Joint Other	Nociceptive Pain
M8468XD	Path fx in oth disease, oth site, subs for fx w routn heal	Arthritis Joint Other	Nociceptive Pain
M8468XG	Path fx in oth disease, oth site, subs for fx w delay heal	Arthritis Joint Other	Nociceptive Pain
M8468XK	Path fx in oth disease, oth site, subs for fx w nonunion	Arthritis Joint Other	Nociceptive Pain

© 2024 Peterson MD et al. *JAMA Neurology.*

M8468XP	Path fx in oth disease, oth site, subs for fx w malunion	Arthritis Joint Other	Nociceptive Pain
M8468XS	Pathological fracture in other disease, other site, sequela	Arthritis Joint Other	Nociceptive Pain
M847	Nontraumatic fracture, not elsewhere classified	Arthritis Joint Other	Nociceptive Pain
M8475	Atypical femoral fracture	Arthritis Joint Lower Limb	Nociceptive Pain
M84750	Atypical femoral fracture, unspecified	Arthritis Joint Lower Limb	Nociceptive Pain
M84750A	Atypical femoral fracture, unspecified, init	Arthritis Joint Lower Limb	Nociceptive Pain
M84750D	Atypical femoral fracture, unspecified, 7thD	Arthritis Joint Lower Limb	Nociceptive Pain
M84750G	Atypical femoral fracture, unspecified, 7thG	Arthritis Joint Lower Limb	Nociceptive Pain
M84750K	Atypical femoral fracture, unspecified, 7thK	Arthritis Joint Lower Limb	Nociceptive Pain
M84750P	Atypical femoral fracture, unspecified, 7thP	Arthritis Joint Lower Limb	Nociceptive Pain
M84750S	Atypical femoral fracture, unspecified, sequela	Arthritis Joint Lower Limb	Nociceptive Pain
M84751	Incomplete atypical femoral fracture, right leg	Arthritis Joint Lower Limb	Nociceptive Pain
M84751A	Incomplete atypical femoral fracture, right leg, init	Arthritis Joint Lower Limb	Nociceptive Pain
M84751D	Incomplete atypical femoral fracture, right leg, 7thD	Arthritis Joint Lower Limb	Nociceptive Pain
M84751G	Incomplete atypical femoral fracture, right leg, 7thG	Arthritis Joint Lower Limb	Nociceptive Pain
M84751K	Incomplete atypical femoral fracture, right leg, 7thK	Arthritis Joint Lower Limb	Nociceptive Pain
M84751P	Incomplete atypical femoral fracture, right leg, 7thP	Arthritis Joint Lower Limb	Nociceptive Pain
M84751S	Incomplete atypical femoral fracture, right leg, sequela	Arthritis Joint Lower Limb	Nociceptive Pain
M84752	Incomplete atypical femoral fracture, left leg	Arthritis Joint Lower Limb	Nociceptive Pain
M84752A	Incomplete atypical femoral fracture, left leg, init	Arthritis Joint Lower Limb	Nociceptive Pain
M84752D	Incomplete atypical femoral fracture, left leg, 7thD	Arthritis Joint Lower Limb	Nociceptive Pain
M84752G	Incomplete atypical femoral fracture, left leg, 7thG	Arthritis Joint Lower Limb	Nociceptive Pain
M84752K	Incomplete atypical femoral fracture, left leg, 7thK	Arthritis Joint Lower Limb	Nociceptive Pain
M84752P	Incomplete atypical femoral fracture, left leg, 7thP	Arthritis Joint Lower Limb	Nociceptive Pain
M84752S	Incomplete atypical femoral fracture, left leg, sequela	Arthritis Joint Lower Limb	Nociceptive Pain
M84753	Incomplete atypical femoral fracture, unspecified leg	Arthritis Joint Lower Limb	Nociceptive Pain
M84753A	Incomplete atypical femoral fracture, unspecified leg, init	Arthritis Joint Lower Limb	Nociceptive Pain
M84753D	Incomplete atypical femoral fracture, unspecified leg, 7thD	Arthritis Joint Lower Limb	Nociceptive Pain
M84753G	Incomplete atypical femoral fracture, unspecified leg, 7thG	Arthritis Joint Lower Limb	Nociceptive Pain
M84753K	Incomplete atypical femoral fracture, unspecified leg, 7thK	Arthritis Joint Lower Limb	Nociceptive Pain
M84753P	Incomplete atypical femoral fracture, unspecified leg, 7thP	Arthritis Joint Lower Limb	Nociceptive Pain
M84753S	Incomplete atypical femoral fracture, unsp leg, sequela	Arthritis Joint Lower Limb	Nociceptive Pain
M84754	Complete transverse atypical femoral fracture, right leg	Arthritis Joint Lower Limb	Nociceptive Pain
M84754A	Complete transverse atyp femoral fracture, right leg, init	Arthritis Joint Lower Limb	Nociceptive Pain
M84754D	Complete transverse atyp femoral fracture, right leg, 7thD	Arthritis Joint Lower Limb	Nociceptive Pain
M84754G	Complete transverse atyp femoral fracture, right leg, 7thG	Arthritis Joint Lower Limb	Nociceptive Pain
M84754K	Complete transverse atyp femoral fracture, right leg, 7thK	Arthritis Joint Lower Limb	Nociceptive Pain

© 2024 Peterson MD et al. *JAMA Neurology.*

M84754P	Complete transverse atyp femoral fracture, right leg, 7thP	Arthritis Joint Lower Limb	Nociceptive Pain
M84754S	Complete transverse atyp femoral fx, right leg, sequela	Arthritis Joint Lower Limb	Nociceptive Pain
M84755	Complete transverse atypical femoral fracture, left leg	Arthritis Joint Lower Limb	Nociceptive Pain
M84755A	Complete transverse atyp femoral fracture, left leg, init	Arthritis Joint Lower Limb	Nociceptive Pain
M84755D	Complete transverse atyp femoral fracture, left leg, 7thD	Arthritis Joint Lower Limb	Nociceptive Pain
M84755G	Complete transverse atyp femoral fracture, left leg, 7thG	Arthritis Joint Lower Limb	Nociceptive Pain
M84755K	Complete transverse atyp femoral fracture, left leg, 7thK	Arthritis Joint Lower Limb	Nociceptive Pain
M84755P	Complete transverse atyp femoral fracture, left leg, 7thP	Arthritis Joint Lower Limb	Nociceptive Pain
M84755S	Complete transverse atyp femoral fracture, left leg, sequela	Arthritis Joint Lower Limb	Nociceptive Pain
M84756	Complete transverse atypical femoral fracture, unsp leg	Arthritis Joint Lower Limb	Nociceptive Pain
M84756A	Complete transverse atyp femoral fracture, unsp leg, init	Arthritis Joint Lower Limb	Nociceptive Pain
M84756D	Complete transverse atyp femoral fracture, unsp leg, 7thD	Arthritis Joint Lower Limb	Nociceptive Pain
M84756G	Complete transverse atyp femoral fracture, unsp leg, 7thG	Arthritis Joint Lower Limb	Nociceptive Pain
M84756K	Complete transverse atyp femoral fracture, unsp leg, 7thK	Arthritis Joint Lower Limb	Nociceptive Pain
M84756P	Complete transverse atyp femoral fracture, unsp leg, 7thP	Arthritis Joint Lower Limb	Nociceptive Pain
M84756S	Complete transverse atyp femoral fracture, unsp leg, sequela	Arthritis Joint Lower Limb	Nociceptive Pain
M84757	Complete oblique atypical femoral fracture, right leg	Arthritis Joint Lower Limb	Nociceptive Pain
M84757A	Complete oblique atypical femoral fracture, right leg, init	Arthritis Joint Lower Limb	Nociceptive Pain
M84757D	Complete oblique atypical femoral fracture, right leg, 7thD	Arthritis Joint Lower Limb	Nociceptive Pain
M84757G	Complete oblique atypical femoral fracture, right leg, 7thG	Arthritis Joint Lower Limb	Nociceptive Pain
M84757K	Complete oblique atypical femoral fracture, right leg, 7thK	Arthritis Joint Lower Limb	Nociceptive Pain
M84757P	Complete oblique atypical femoral fracture, right leg, 7thP	Arthritis Joint Lower Limb	Nociceptive Pain
M84757S	Complete oblique atyp femoral fracture, right leg, sequela	Arthritis Joint Lower Limb	Nociceptive Pain
M84758	Complete oblique atypical femoral fracture, left leg	Arthritis Joint Lower Limb	Nociceptive Pain
M84758A	Complete oblique atypical femoral fracture, left leg, init	Arthritis Joint Lower Limb	Nociceptive Pain
M84758D	Complete oblique atypical femoral fracture, left leg, 7thD	Arthritis Joint Lower Limb	Nociceptive Pain
M84758G	Complete oblique atypical femoral fracture, left leg, 7thG	Arthritis Joint Lower Limb	Nociceptive Pain
M84758K	Complete oblique atypical femoral fracture, left leg, 7thK	Arthritis Joint Lower Limb	Nociceptive Pain
M84758P	Complete oblique atypical femoral fracture, left leg, 7thP	Arthritis Joint Lower Limb	Nociceptive Pain
M84758S	Complete oblique atyp femoral fracture, left leg, sequela	Arthritis Joint Lower Limb	Nociceptive Pain
M84759	Complete oblique atypical femoral fracture, unspecified leg	Arthritis Joint Lower Limb	Nociceptive Pain
M84759A	Complete oblique atypical femoral fracture, unsp leg, init	Arthritis Joint Lower Limb	Nociceptive Pain
M84759D	Complete oblique atypical femoral fracture, unsp leg, 7thD	Arthritis Joint Lower Limb	Nociceptive Pain
M84759G	Complete oblique atypical femoral fracture, unsp leg, 7thG	Arthritis Joint Lower Limb	Nociceptive Pain
M84759K	Complete oblique atypical femoral fracture, unsp leg, 7thK	Arthritis Joint Lower Limb	Nociceptive Pain
M84759P	Complete oblique atypical femoral fracture, unsp leg, 7thP	Arthritis Joint Lower Limb	Nociceptive Pain
M84759S	Complete oblique atyp femoral fracture, unsp leg, sequela	Arthritis Joint Lower Limb	Nociceptive Pain

© 2024 Peterson MD et al. *JAMA Neurology.*

M848	Other disorders of continuity of bone	Arthritis Joint Other	Nociceptive Pain
M8480	Other disorders of continuity of bone, unspecified site	Arthritis Joint Other	Nociceptive Pain
M8481	Other disorders of continuity of bone, shoulder	Arthritis Joint Upper Limb	Nociceptive Pain
M84811	Other disorders of continuity of bone, right shoulder	Arthritis Joint Upper Limb	Nociceptive Pain
M84812	Other disorders of continuity of bone, left shoulder	Arthritis Joint Upper Limb	Nociceptive Pain
M84819	Other disorders of continuity of bone, unspecified shoulder	Arthritis Joint Upper Limb	Nociceptive Pain
M8482	Other disorders of continuity of bone, humerus	Arthritis Joint Upper Limb	Nociceptive Pain
M84821	Other disorders of continuity of bone, right humerus	Arthritis Joint Upper Limb	Nociceptive Pain
M84822	Other disorders of continuity of bone, left humerus	Arthritis Joint Upper Limb	Nociceptive Pain
M84829	Other disorders of continuity of bone, unspecified humerus	Arthritis Joint Upper Limb	Nociceptive Pain
M8483	Other disorders of continuity of bone, ulna and radius	Arthritis Joint Upper Limb	Nociceptive Pain
M84831	Other disorders of continuity of bone, right ulna	Arthritis Joint Upper Limb	Nociceptive Pain
M84832	Other disorders of continuity of bone, left ulna	Arthritis Joint Upper Limb	Nociceptive Pain
M84833	Other disorders of continuity of bone, right radius	Arthritis Joint Upper Limb	Nociceptive Pain
M84834	Other disorders of continuity of bone, left radius	Arthritis Joint Upper Limb	Nociceptive Pain
M84839	Other disorders of continuity of bone, unsp ulna and radius	Arthritis Joint Upper Limb	Nociceptive Pain
M8484	Other disorders of continuity of bone, hand	Arthritis Joint Upper Limb	Nociceptive Pain
M84841	Other disorders of continuity of bone, right hand	Arthritis Joint Upper Limb	Nociceptive Pain
M84842	Other disorders of continuity of bone, left hand	Arthritis Joint Upper Limb	Nociceptive Pain
M84849	Other disorders of continuity of bone, unspecified hand	Arthritis Joint Upper Limb	Nociceptive Pain
M8485	Oth disorders of continuity of bone, pelvic region and thigh	Arthritis Joint Lower Limb	Nociceptive Pain
M84851	Oth disord of continuity of bone, right pelv rgn and thigh	Arthritis Joint Lower Limb	Nociceptive Pain
M84852	Oth disord of continuity of bone, left pelv region and thigh	Arthritis Joint Lower Limb	Nociceptive Pain
M84859	Oth disord of continuity of bone, unsp pelv region and thigh	Arthritis Joint Lower Limb	Nociceptive Pain
M8486	Other disorders of continuity of bone, tibia and fibula	Arthritis Joint Lower Limb	Nociceptive Pain
M84861	Other disorders of continuity of bone, right tibia	Arthritis Joint Lower Limb	Nociceptive Pain
M84862	Other disorders of continuity of bone, left tibia	Arthritis Joint Lower Limb	Nociceptive Pain
M84863	Other disorders of continuity of bone, right fibula	Arthritis Joint Lower Limb	Nociceptive Pain
M84864	Other disorders of continuity of bone, left fibula	Arthritis Joint Lower Limb	Nociceptive Pain
M84869	Other disorders of continuity of bone, unsp tibia and fibula	Arthritis Joint Lower Limb	Nociceptive Pain
M8487	Other disorders of continuity of bone, ankle and foot	Arthritis Joint Lower Limb	Nociceptive Pain
M84871	Other disorders of continuity of bone, right ankle and foot	Arthritis Joint Lower Limb	Nociceptive Pain
M84872	Other disorders of continuity of bone, left ankle and foot	Arthritis Joint Lower Limb	Nociceptive Pain
M84879	Other disorders of continuity of bone, unsp ankle and foot	Arthritis Joint Lower Limb	Nociceptive Pain
M8488	Other disorders of continuity of bone, other site	Arthritis Joint Other	Nociceptive Pain
M849	Disorder of continuity of bone, unspecified	Arthritis Joint Other	Nociceptive Pain
M85	Other disorders of bone density and structure	Arthritis Joint Other	Nociceptive Pain

© 2024 Peterson MD et al. *JAMA Neurology.*

M850	Fibrous dysplasia (monostotic)	Arthritis Joint Other	Nociceptive Pain
M8500	Fibrous dysplasia (monostotic), unspecified site	Arthritis Joint Other	Nociceptive Pain
M8501	Fibrous dysplasia (monostotic), shoulder	Arthritis Joint Upper Limb	Nociceptive Pain
M85011	Fibrous dysplasia (monostotic), right shoulder	Arthritis Joint Upper Limb	Nociceptive Pain
M85012	Fibrous dysplasia (monostotic), left shoulder	Arthritis Joint Upper Limb	Nociceptive Pain
M85019	Fibrous dysplasia (monostotic), unspecified shoulder	Arthritis Joint Upper Limb	Nociceptive Pain
M8502	Fibrous dysplasia (monostotic), upper arm	Arthritis Joint Upper Limb	Nociceptive Pain
M85021	Fibrous dysplasia (monostotic), right upper arm	Arthritis Joint Upper Limb	Nociceptive Pain
M85022	Fibrous dysplasia (monostotic), left upper arm	Arthritis Joint Upper Limb	Nociceptive Pain
M85029	Fibrous dysplasia (monostotic), unspecified upper arm	Arthritis Joint Upper Limb	Nociceptive Pain
M8503	Fibrous dysplasia (monostotic), forearm	Arthritis Joint Upper Limb	Nociceptive Pain
M85031	Fibrous dysplasia (monostotic), right forearm	Arthritis Joint Upper Limb	Nociceptive Pain
M85032	Fibrous dysplasia (monostotic), left forearm	Arthritis Joint Upper Limb	Nociceptive Pain
M85039	Fibrous dysplasia (monostotic), unspecified forearm	Arthritis Joint Upper Limb	Nociceptive Pain
M8504	Fibrous dysplasia (monostotic), hand	Arthritis Joint Upper Limb	Nociceptive Pain
M85041	Fibrous dysplasia (monostotic), right hand	Arthritis Joint Upper Limb	Nociceptive Pain
M85042	Fibrous dysplasia (monostotic), left hand	Arthritis Joint Upper Limb	Nociceptive Pain
M85049	Fibrous dysplasia (monostotic), unspecified hand	Arthritis Joint Upper Limb	Nociceptive Pain
M8505	Fibrous dysplasia (monostotic), thigh	Arthritis Joint Lower Limb	Nociceptive Pain
M85051	Fibrous dysplasia (monostotic), right thigh	Arthritis Joint Lower Limb	Nociceptive Pain
M85052	Fibrous dysplasia (monostotic), left thigh	Arthritis Joint Lower Limb	Nociceptive Pain
M85059	Fibrous dysplasia (monostotic), unspecified thigh	Arthritis Joint Lower Limb	Nociceptive Pain
M8506	Fibrous dysplasia (monostotic), lower leg	Arthritis Joint Lower Limb	Nociceptive Pain
M85061	Fibrous dysplasia (monostotic), right lower leg	Arthritis Joint Lower Limb	Nociceptive Pain
M85062	Fibrous dysplasia (monostotic), left lower leg	Arthritis Joint Lower Limb	Nociceptive Pain
M85069	Fibrous dysplasia (monostotic), unspecified lower leg	Arthritis Joint Lower Limb	Nociceptive Pain
M8507	Fibrous dysplasia (monostotic), ankle and foot	Arthritis Joint Lower Limb	Nociceptive Pain
M85071	Fibrous dysplasia (monostotic), right ankle and foot	Arthritis Joint Lower Limb	Nociceptive Pain
M85072	Fibrous dysplasia (monostotic), left ankle and foot	Arthritis Joint Lower Limb	Nociceptive Pain
M85079	Fibrous dysplasia (monostotic), unspecified ankle and foot	Arthritis Joint Lower Limb	Nociceptive Pain
M8508	Fibrous dysplasia (monostotic), other site	Arthritis Joint Other	Nociceptive Pain
M8509	Fibrous dysplasia (monostotic), multiple sites	Arthritis Joint Other	Nociceptive Pain
M851	Skeletal fluorosis	Arthritis Joint Other	Nociceptive Pain
M8510	Skeletal fluorosis, unspecified site	Arthritis Joint Other	Nociceptive Pain
M8511	Skeletal fluorosis, shoulder	Arthritis Joint Upper Limb	Nociceptive Pain
M85111	Skeletal fluorosis, right shoulder	Arthritis Joint Upper Limb	Nociceptive Pain
M85112	Skeletal fluorosis, left shoulder	Arthritis Joint Upper Limb	Nociceptive Pain

© 2024 Peterson MD et al. *JAMA Neurology.*

M85119	Skeletal fluorosis, unspecified shoulder	Arthritis Joint Upper Limb	Nociceptive Pain
M8512	Skeletal fluorosis, upper arm	Arthritis Joint Upper Limb	Nociceptive Pain
M85121	Skeletal fluorosis, right upper arm	Arthritis Joint Upper Limb	Nociceptive Pain
M85122	Skeletal fluorosis, left upper arm	Arthritis Joint Upper Limb	Nociceptive Pain
M85129	Skeletal fluorosis, unspecified upper arm	Arthritis Joint Upper Limb	Nociceptive Pain
M8513	Skeletal fluorosis, forearm	Arthritis Joint Upper Limb	Nociceptive Pain
M85131	Skeletal fluorosis, right forearm	Arthritis Joint Upper Limb	Nociceptive Pain
M85132	Skeletal fluorosis, left forearm	Arthritis Joint Upper Limb	Nociceptive Pain
M85139	Skeletal fluorosis, unspecified forearm	Arthritis Joint Upper Limb	Nociceptive Pain
M8514	Skeletal fluorosis, hand	Arthritis Joint Upper Limb	Nociceptive Pain
M85141	Skeletal fluorosis, right hand	Arthritis Joint Upper Limb	Nociceptive Pain
M85142	Skeletal fluorosis, left hand	Arthritis Joint Upper Limb	Nociceptive Pain
M85149	Skeletal fluorosis, unspecified hand	Arthritis Joint Upper Limb	Nociceptive Pain
M8515	Skeletal fluorosis, thigh	Arthritis Joint Lower Limb	Nociceptive Pain
M85151	Skeletal fluorosis, right thigh	Arthritis Joint Lower Limb	Nociceptive Pain
M85152	Skeletal fluorosis, left thigh	Arthritis Joint Lower Limb	Nociceptive Pain
M85159	Skeletal fluorosis, unspecified thigh	Arthritis Joint Lower Limb	Nociceptive Pain
M8516	Skeletal fluorosis, lower leg	Arthritis Joint Lower Limb	Nociceptive Pain
M85161	Skeletal fluorosis, right lower leg	Arthritis Joint Lower Limb	Nociceptive Pain
M85162	Skeletal fluorosis, left lower leg	Arthritis Joint Lower Limb	Nociceptive Pain
M85169	Skeletal fluorosis, unspecified lower leg	Arthritis Joint Lower Limb	Nociceptive Pain
M8517	Skeletal fluorosis, ankle and foot	Arthritis Joint Lower Limb	Nociceptive Pain
M85171	Skeletal fluorosis, right ankle and foot	Arthritis Joint Lower Limb	Nociceptive Pain
M85172	Skeletal fluorosis, left ankle and foot	Arthritis Joint Lower Limb	Nociceptive Pain
M85179	Skeletal fluorosis, unspecified ankle and foot	Arthritis Joint Lower Limb	Nociceptive Pain
M8518	Skeletal fluorosis, other site	Arthritis Joint Other	Nociceptive Pain
M8519	Skeletal fluorosis, multiple sites	Arthritis Joint Other	Nociceptive Pain
M852	Hyperostosis of skull	Arthritis Joint Other	Nociceptive Pain
M853	Osteitis condensans	Arthritis Joint Other	Nociceptive Pain
M8530	Osteitis condensans, unspecified site	Arthritis Joint Other	Nociceptive Pain
M8531	Osteitis condensans, shoulder	Arthritis Joint Upper Limb	Nociceptive Pain
M85311	Osteitis condensans, right shoulder	Arthritis Joint Upper Limb	Nociceptive Pain
M85312	Osteitis condensans, left shoulder	Arthritis Joint Upper Limb	Nociceptive Pain
M85319	Osteitis condensans, unspecified shoulder	Arthritis Joint Upper Limb	Nociceptive Pain
M8532	Osteitis condensans, upper arm	Arthritis Joint Upper Limb	Nociceptive Pain
M85321	Osteitis condensans, right upper arm	Arthritis Joint Upper Limb	Nociceptive Pain
M85322	Osteitis condensans, left upper arm	Arthritis Joint Upper Limb	Nociceptive Pain

© 2024 Peterson MD et al. *JAMA Neurology.*

M85329	Osteitis condensans, unspecified upper arm	Arthritis Joint Upper Limb	Nociceptive Pain
M8533	Osteitis condensans, forearm	Arthritis Joint Upper Limb	Nociceptive Pain
M85331	Osteitis condensans, right forearm	Arthritis Joint Upper Limb	Nociceptive Pain
M85332	Osteitis condensans, left forearm	Arthritis Joint Upper Limb	Nociceptive Pain
M85339	Osteitis condensans, unspecified forearm	Arthritis Joint Upper Limb	Nociceptive Pain
M8534	Osteitis condensans, hand	Arthritis Joint Upper Limb	Nociceptive Pain
M85341	Osteitis condensans, right hand	Arthritis Joint Upper Limb	Nociceptive Pain
M85342	Osteitis condensans, left hand	Arthritis Joint Upper Limb	Nociceptive Pain
M85349	Osteitis condensans, unspecified hand	Arthritis Joint Upper Limb	Nociceptive Pain
M8535	Osteitis condensans, thigh	Arthritis Joint Lower Limb	Nociceptive Pain
M85351	Osteitis condensans, right thigh	Arthritis Joint Lower Limb	Nociceptive Pain
M85352	Osteitis condensans, left thigh	Arthritis Joint Lower Limb	Nociceptive Pain
M85359	Osteitis condensans, unspecified thigh	Arthritis Joint Lower Limb	Nociceptive Pain
M8536	Osteitis condensans, lower leg	Arthritis Joint Lower Limb	Nociceptive Pain
M85361	Osteitis condensans, right lower leg	Arthritis Joint Lower Limb	Nociceptive Pain
M85362	Osteitis condensans, left lower leg	Arthritis Joint Lower Limb	Nociceptive Pain
M85369	Osteitis condensans, unspecified lower leg	Arthritis Joint Lower Limb	Nociceptive Pain
M8537	Osteitis condensans, ankle and foot	Arthritis Joint Lower Limb	Nociceptive Pain
M85371	Osteitis condensans, right ankle and foot	Arthritis Joint Lower Limb	Nociceptive Pain
M85372	Osteitis condensans, left ankle and foot	Arthritis Joint Lower Limb	Nociceptive Pain
M85379	Osteitis condensans, unspecified ankle and foot	Arthritis Joint Lower Limb	Nociceptive Pain
M8538	Osteitis condensans, other site	Arthritis Joint Other	Nociceptive Pain
M8539	Osteitis condensans, multiple sites	Arthritis Joint Other	Nociceptive Pain
M854	Solitary bone cyst	Arthritis Joint Other	Nociceptive Pain
M8540	Solitary bone cyst, unspecified site	Arthritis Joint Other	Nociceptive Pain
M8541	Solitary bone cyst, shoulder	Arthritis Joint Upper Limb	Nociceptive Pain
M85411	Solitary bone cyst, right shoulder	Arthritis Joint Upper Limb	Nociceptive Pain
M85412	Solitary bone cyst, left shoulder	Arthritis Joint Upper Limb	Nociceptive Pain
M85419	Solitary bone cyst, unspecified shoulder	Arthritis Joint Upper Limb	Nociceptive Pain
M8542	Solitary bone cyst, humerus	Arthritis Joint Upper Limb	Nociceptive Pain
M85421	Solitary bone cyst, right humerus	Arthritis Joint Upper Limb	Nociceptive Pain
M85422	Solitary bone cyst, left humerus	Arthritis Joint Upper Limb	Nociceptive Pain
M85429	Solitary bone cyst, unspecified humerus	Arthritis Joint Upper Limb	Nociceptive Pain
M8543	Solitary bone cyst, ulna and radius	Arthritis Joint Upper Limb	Nociceptive Pain
M85431	Solitary bone cyst, right ulna and radius	Arthritis Joint Upper Limb	Nociceptive Pain
M85432	Solitary bone cyst, left ulna and radius	Arthritis Joint Upper Limb	Nociceptive Pain
M85439	Solitary bone cyst, unspecified ulna and radius	Arthritis Joint Upper Limb	Nociceptive Pain

© 2024 Peterson MD et al. *JAMA Neurology.*

M8544	Solitary bone cyst, hand	Arthritis Joint Upper Limb	Nociceptive Pain
M85441	Solitary bone cyst, right hand	Arthritis Joint Upper Limb	Nociceptive Pain
M85442	Solitary bone cyst, left hand	Arthritis Joint Upper Limb	Nociceptive Pain
M85449	Solitary bone cyst, unspecified hand	Arthritis Joint Upper Limb	Nociceptive Pain
M8545	Solitary bone cyst, pelvis	Arthritis Joint Spine and Hips	Nociceptive Pain
M85451	Solitary bone cyst, right pelvis	Arthritis Joint Spine and Hips	Nociceptive Pain
M85452	Solitary bone cyst, left pelvis	Arthritis Joint Spine and Hips	Nociceptive Pain
M85459	Solitary bone cyst, unspecified pelvis	Arthritis Joint Spine and Hips	Nociceptive Pain
M8546	Solitary bone cyst, tibia and fibula	Arthritis Joint Lower Limb	Nociceptive Pain
M85461	Solitary bone cyst, right tibia and fibula	Arthritis Joint Lower Limb	Nociceptive Pain
M85462	Solitary bone cyst, left tibia and fibula	Arthritis Joint Lower Limb	Nociceptive Pain
M85469	Solitary bone cyst, unspecified tibia and fibula	Arthritis Joint Lower Limb	Nociceptive Pain
M8547	Solitary bone cyst, ankle and foot	Arthritis Joint Lower Limb	Nociceptive Pain
M85471	Solitary bone cyst, right ankle and foot	Arthritis Joint Lower Limb	Nociceptive Pain
M85472	Solitary bone cyst, left ankle and foot	Arthritis Joint Lower Limb	Nociceptive Pain
M85479	Solitary bone cyst, unspecified ankle and foot	Arthritis Joint Lower Limb	Nociceptive Pain
M8548	Solitary bone cyst, other site	Arthritis Joint Other	Nociceptive Pain
M855	Aneurysmal bone cyst	Arthritis Joint Other	Nociceptive Pain
M8550	Aneurysmal bone cyst, unspecified site	Arthritis Joint Other	Nociceptive Pain
M8551	Aneurysmal bone cyst, shoulder	Arthritis Joint Upper Limb	Nociceptive Pain
M85511	Aneurysmal bone cyst, right shoulder	Arthritis Joint Upper Limb	Nociceptive Pain
M85512	Aneurysmal bone cyst, left shoulder	Arthritis Joint Upper Limb	Nociceptive Pain
M85519	Aneurysmal bone cyst, unspecified shoulder	Arthritis Joint Upper Limb	Nociceptive Pain
M8552	Aneurysmal bone cyst, upper arm	Arthritis Joint Upper Limb	Nociceptive Pain
M85521	Aneurysmal bone cyst, right upper arm	Arthritis Joint Upper Limb	Nociceptive Pain
M85522	Aneurysmal bone cyst, left upper arm	Arthritis Joint Upper Limb	Nociceptive Pain
M85529	Aneurysmal bone cyst, unspecified upper arm	Arthritis Joint Upper Limb	Nociceptive Pain
M8553	Aneurysmal bone cyst, forearm	Arthritis Joint Upper Limb	Nociceptive Pain
M85531	Aneurysmal bone cyst, right forearm	Arthritis Joint Upper Limb	Nociceptive Pain
M85532	Aneurysmal bone cyst, left forearm	Arthritis Joint Upper Limb	Nociceptive Pain
M85539	Aneurysmal bone cyst, unspecified forearm	Arthritis Joint Upper Limb	Nociceptive Pain
M8554	Aneurysmal bone cyst, hand	Arthritis Joint Upper Limb	Nociceptive Pain
M85541	Aneurysmal bone cyst, right hand	Arthritis Joint Upper Limb	Nociceptive Pain
M85542	Aneurysmal bone cyst, left hand	Arthritis Joint Upper Limb	Nociceptive Pain
M85549	Aneurysmal bone cyst, unspecified hand	Arthritis Joint Upper Limb	Nociceptive Pain
M8555	Aneurysmal bone cyst, thigh	Arthritis Joint Lower Limb	Nociceptive Pain
M85551	Aneurysmal bone cyst, right thigh	Arthritis Joint Lower Limb	Nociceptive Pain

© 2024 Peterson MD et al. *JAMA Neurology.*

M85552	Aneurysmal bone cyst, left thigh	Arthritis Joint Lower Limb	Nociceptive Pain
M85559	Aneurysmal bone cyst, unspecified thigh	Arthritis Joint Lower Limb	Nociceptive Pain
M8556	Aneurysmal bone cyst, lower leg	Arthritis Joint Lower Limb	Nociceptive Pain
M85561	Aneurysmal bone cyst, right lower leg	Arthritis Joint Lower Limb	Nociceptive Pain
M85562	Aneurysmal bone cyst, left lower leg	Arthritis Joint Lower Limb	Nociceptive Pain
M85569	Aneurysmal bone cyst, unspecified lower leg	Arthritis Joint Lower Limb	Nociceptive Pain
M8557	Aneurysmal bone cyst, ankle and foot	Arthritis Joint Lower Limb	Nociceptive Pain
M85571	Aneurysmal bone cyst, right ankle and foot	Arthritis Joint Lower Limb	Nociceptive Pain
M85572	Aneurysmal bone cyst, left ankle and foot	Arthritis Joint Lower Limb	Nociceptive Pain
M85579	Aneurysmal bone cyst, unspecified ankle and foot	Arthritis Joint Lower Limb	Nociceptive Pain
M8558	Aneurysmal bone cyst, other site	Arthritis Joint Other	Nociceptive Pain
M8559	Aneurysmal bone cyst, multiple sites	Arthritis Joint Other	Nociceptive Pain
M856	Other cyst of bone	Arthritis Joint Other	Nociceptive Pain
M8560	Other cyst of bone, unspecified site	Arthritis Joint Other	Nociceptive Pain
M8561	Other cyst of bone, shoulder	Arthritis Joint Upper Limb	Nociceptive Pain
M85611	Other cyst of bone, right shoulder	Arthritis Joint Upper Limb	Nociceptive Pain
M85612	Other cyst of bone, left shoulder	Arthritis Joint Upper Limb	Nociceptive Pain
M85619	Other cyst of bone, unspecified shoulder	Arthritis Joint Upper Limb	Nociceptive Pain
M8562	Other cyst of bone, upper arm	Arthritis Joint Upper Limb	Nociceptive Pain
M85621	Other cyst of bone, right upper arm	Arthritis Joint Upper Limb	Nociceptive Pain
M85622	Other cyst of bone, left upper arm	Arthritis Joint Upper Limb	Nociceptive Pain
M85629	Other cyst of bone, unspecified upper arm	Arthritis Joint Upper Limb	Nociceptive Pain
M8563	Other cyst of bone, forearm	Arthritis Joint Upper Limb	Nociceptive Pain
M85631	Other cyst of bone, right forearm	Arthritis Joint Upper Limb	Nociceptive Pain
M85632	Other cyst of bone, left forearm	Arthritis Joint Upper Limb	Nociceptive Pain
M85639	Other cyst of bone, unspecified forearm	Arthritis Joint Upper Limb	Nociceptive Pain
M8564	Other cyst of bone, hand	Arthritis Joint Upper Limb	Nociceptive Pain
M85641	Other cyst of bone, right hand	Arthritis Joint Upper Limb	Nociceptive Pain
M85642	Other cyst of bone, left hand	Arthritis Joint Upper Limb	Nociceptive Pain
M85649	Other cyst of bone, unspecified hand	Arthritis Joint Upper Limb	Nociceptive Pain
M8565	Other cyst of bone, thigh	Arthritis Joint Lower Limb	Nociceptive Pain
M85651	Other cyst of bone, right thigh	Arthritis Joint Lower Limb	Nociceptive Pain
M85652	Other cyst of bone, left thigh	Arthritis Joint Lower Limb	Nociceptive Pain
M85659	Other cyst of bone, unspecified thigh	Arthritis Joint Lower Limb	Nociceptive Pain
M8566	Other cyst of bone, lower leg	Arthritis Joint Lower Limb	Nociceptive Pain
M85661	Other cyst of bone, right lower leg	Arthritis Joint Lower Limb	Nociceptive Pain
M85662	Other cyst of bone, left lower leg	Arthritis Joint Lower Limb	Nociceptive Pain

© 2024 Peterson MD et al. *JAMA Neurology.*

M85669	Other cyst of bone, unspecified lower leg	Arthritis Joint Lower Limb	Nociceptive Pain
M8567	Other cyst of bone, ankle and foot	Arthritis Joint Lower Limb	Nociceptive Pain
M85671	Other cyst of bone, right ankle and foot	Arthritis Joint Lower Limb	Nociceptive Pain
M85672	Other cyst of bone, left ankle and foot	Arthritis Joint Lower Limb	Nociceptive Pain
M85679	Other cyst of bone, unspecified ankle and foot	Arthritis Joint Lower Limb	Nociceptive Pain
M8568	Other cyst of bone, other site	Arthritis Joint Other	Nociceptive Pain
M8569	Other cyst of bone, multiple sites	Arthritis Joint Other	Nociceptive Pain
M858	Other specified disorders of bone density and structure	Arthritis Joint Other	Nociceptive Pain
M8580	Oth disrd of bone density and structure, unspecified site	Arthritis Joint Other	Nociceptive Pain
M8581	Oth disrd of bone density and structure, shoulder	Arthritis Joint Upper Limb	Nociceptive Pain
M85811	Oth disrd of bone density and structure, right shoulder	Arthritis Joint Upper Limb	Nociceptive Pain
M85812	Oth disrd of bone density and structure, left shoulder	Arthritis Joint Upper Limb	Nociceptive Pain
M85819	Oth disrd of bone density and structure, unsp shoulder	Arthritis Joint Upper Limb	Nociceptive Pain
M8582	Oth disrd of bone density and structure, upper arm	Arthritis Joint Upper Limb	Nociceptive Pain
M85821	Oth disrd of bone density and structure, right upper arm	Arthritis Joint Upper Limb	Nociceptive Pain
M85822	Oth disrd of bone density and structure, left upper arm	Arthritis Joint Upper Limb	Nociceptive Pain
M85829	Oth disrd of bone density and structure, unsp upper arm	Arthritis Joint Upper Limb	Nociceptive Pain
M8583	Oth disrd of bone density and structure, forearm	Arthritis Joint Upper Limb	Nociceptive Pain
M85831	Oth disrd of bone density and structure, right forearm	Arthritis Joint Upper Limb	Nociceptive Pain
M85832	Oth disrd of bone density and structure, left forearm	Arthritis Joint Upper Limb	Nociceptive Pain
M85839	Oth disrd of bone density and structure, unspecified forearm	Arthritis Joint Upper Limb	Nociceptive Pain
M8584	Oth disrd of bone density and structure, hand	Arthritis Joint Upper Limb	Nociceptive Pain
M85841	Oth disrd of bone density and structure, right hand	Arthritis Joint Upper Limb	Nociceptive Pain
M85842	Oth disrd of bone density and structure, left hand	Arthritis Joint Upper Limb	Nociceptive Pain
M85849	Oth disrd of bone density and structure, unspecified hand	Arthritis Joint Upper Limb	Nociceptive Pain
M8585	Oth disrd of bone density and structure, thigh	Arthritis Joint Lower Limb	Nociceptive Pain
M85851	Oth disrd of bone density and structure, right thigh	Arthritis Joint Lower Limb	Nociceptive Pain
M85852	Oth disrd of bone density and structure, left thigh	Arthritis Joint Lower Limb	Nociceptive Pain
M85859	Oth disrd of bone density and structure, unspecified thigh	Arthritis Joint Lower Limb	Nociceptive Pain
M8586	Oth disrd of bone density and structure, lower leg	Arthritis Joint Lower Limb	Nociceptive Pain
M85861	Oth disrd of bone density and structure, right lower leg	Arthritis Joint Lower Limb	Nociceptive Pain
M85862	Oth disrd of bone density and structure, left lower leg	Arthritis Joint Lower Limb	Nociceptive Pain
M85869	Oth disrd of bone density and structure, unsp lower leg	Arthritis Joint Lower Limb	Nociceptive Pain
M8587	Oth disrd of bone density and structure, ankle and foot	Arthritis Joint Lower Limb	Nociceptive Pain
M85871	Oth disrd of bone density and structure, right ank/ft	Arthritis Joint Lower Limb	Nociceptive Pain
M85872	Oth disrd of bone density and structure, left ankle and foot	Arthritis Joint Lower Limb	Nociceptive Pain
M85879	Oth disrd of bone density and structure, unsp ankle and foot	Arthritis Joint Lower Limb	Nociceptive Pain

© 2024 Peterson MD et al. *JAMA Neurology.*

M8588	Oth disrd of bone density and structure, other site	Arthritis Joint Other	Nociceptive Pain
M8589	Oth disrd of bone density and structure, multiple sites	Arthritis Joint Other	Nociceptive Pain
M859	Disorder of bone density and structure, unspecified	Arthritis Joint Other	Nociceptive Pain
M86	Osteomyelitis	Arthritis Joint Other	Nociceptive Pain
M860	Acute hematogenous osteomyelitis	Arthritis Joint Other	Nociceptive Pain
M8600	Acute hematogenous osteomyelitis, unspecified site	Arthritis Joint Other	Nociceptive Pain
M8601	Acute hematogenous osteomyelitis, shoulder	Arthritis Joint Upper Limb	Nociceptive Pain
M86011	Acute hematogenous osteomyelitis, right shoulder	Arthritis Joint Upper Limb	Nociceptive Pain
M86012	Acute hematogenous osteomyelitis, left shoulder	Arthritis Joint Upper Limb	Nociceptive Pain
M86019	Acute hematogenous osteomyelitis, unspecified shoulder	Arthritis Joint Upper Limb	Nociceptive Pain
M8602	Acute hematogenous osteomyelitis, humerus	Arthritis Joint Upper Limb	Nociceptive Pain
M86021	Acute hematogenous osteomyelitis, right humerus	Arthritis Joint Upper Limb	Nociceptive Pain
M86022	Acute hematogenous osteomyelitis, left humerus	Arthritis Joint Upper Limb	Nociceptive Pain
M86029	Acute hematogenous osteomyelitis, unspecified humerus	Arthritis Joint Upper Limb	Nociceptive Pain
M8603	Acute hematogenous osteomyelitis, radius and ulna	Arthritis Joint Upper Limb	Nociceptive Pain
M86031	Acute hematogenous osteomyelitis, right radius and ulna	Arthritis Joint Upper Limb	Nociceptive Pain
M86032	Acute hematogenous osteomyelitis, left radius and ulna	Arthritis Joint Upper Limb	Nociceptive Pain
M86039	Acute hematogenous osteomyelitis, unsp radius and ulna	Arthritis Joint Upper Limb	Nociceptive Pain
M8604	Acute hematogenous osteomyelitis, hand	Arthritis Joint Upper Limb	Nociceptive Pain
M86041	Acute hematogenous osteomyelitis, right hand	Arthritis Joint Upper Limb	Nociceptive Pain
M86042	Acute hematogenous osteomyelitis, left hand	Arthritis Joint Upper Limb	Nociceptive Pain
M86049	Acute hematogenous osteomyelitis, unspecified hand	Arthritis Joint Upper Limb	Nociceptive Pain
M8605	Acute hematogenous osteomyelitis, femur	Arthritis Joint Lower Limb	Nociceptive Pain
M86051	Acute hematogenous osteomyelitis, right femur	Arthritis Joint Lower Limb	Nociceptive Pain
M86052	Acute hematogenous osteomyelitis, left femur	Arthritis Joint Lower Limb	Nociceptive Pain
M86059	Acute hematogenous osteomyelitis, unspecified femur	Arthritis Joint Lower Limb	Nociceptive Pain
M8606	Acute hematogenous osteomyelitis, tibia and fibula	Arthritis Joint Lower Limb	Nociceptive Pain
M86061	Acute hematogenous osteomyelitis, right tibia and fibula	Arthritis Joint Lower Limb	Nociceptive Pain
M86062	Acute hematogenous osteomyelitis, left tibia and fibula	Arthritis Joint Lower Limb	Nociceptive Pain
M86069	Acute hematogenous osteomyelitis, unsp tibia and fibula	Arthritis Joint Lower Limb	Nociceptive Pain
M8607	Acute hematogenous osteomyelitis, ankle and foot	Arthritis Joint Lower Limb	Nociceptive Pain
M86071	Acute hematogenous osteomyelitis, right ankle and foot	Arthritis Joint Lower Limb	Nociceptive Pain
M86072	Acute hematogenous osteomyelitis, left ankle and foot	Arthritis Joint Lower Limb	Nociceptive Pain
M86079	Acute hematogenous osteomyelitis, unspecified ankle and foot	Arthritis Joint Lower Limb	Nociceptive Pain
M8608	Acute hematogenous osteomyelitis, other sites	Arthritis Joint Other	Nociceptive Pain
M8609	Acute hematogenous osteomyelitis, multiple sites	Arthritis Joint Other	Nociceptive Pain
M861	Other acute osteomyelitis	Arthritis Joint Other	Nociceptive Pain

© 2024 Peterson MD et al. *JAMA Neurology.*

M8610	Other acute osteomyelitis, unspecified site	Arthritis Joint Other	Nociceptive Pain
M8611	Other acute osteomyelitis, shoulder	Arthritis Joint Upper Limb	Nociceptive Pain
M86111	Other acute osteomyelitis, right shoulder	Arthritis Joint Upper Limb	Nociceptive Pain
M86112	Other acute osteomyelitis, left shoulder	Arthritis Joint Upper Limb	Nociceptive Pain
M86119	Other acute osteomyelitis, unspecified shoulder	Arthritis Joint Upper Limb	Nociceptive Pain
M8612	Other acute osteomyelitis, humerus	Arthritis Joint Upper Limb	Nociceptive Pain
M86121	Other acute osteomyelitis, right humerus	Arthritis Joint Upper Limb	Nociceptive Pain
M86122	Other acute osteomyelitis, left humerus	Arthritis Joint Upper Limb	Nociceptive Pain
M86129	Other acute osteomyelitis, unspecified humerus	Arthritis Joint Upper Limb	Nociceptive Pain
M8613	Other acute osteomyelitis, radius and ulna	Arthritis Joint Upper Limb	Nociceptive Pain
M86131	Other acute osteomyelitis, right radius and ulna	Arthritis Joint Upper Limb	Nociceptive Pain
M86132	Other acute osteomyelitis, left radius and ulna	Arthritis Joint Upper Limb	Nociceptive Pain
M86139	Other acute osteomyelitis, unspecified radius and ulna	Arthritis Joint Upper Limb	Nociceptive Pain
M8614	Other acute osteomyelitis, hand	Arthritis Joint Upper Limb	Nociceptive Pain
M86141	Other acute osteomyelitis, right hand	Arthritis Joint Upper Limb	Nociceptive Pain
M86142	Other acute osteomyelitis, left hand	Arthritis Joint Upper Limb	Nociceptive Pain
M86149	Other acute osteomyelitis, unspecified hand	Arthritis Joint Upper Limb	Nociceptive Pain
M8615	Other acute osteomyelitis, femur	Arthritis Joint Lower Limb	Nociceptive Pain
M86151	Other acute osteomyelitis, right femur	Arthritis Joint Lower Limb	Nociceptive Pain
M86152	Other acute osteomyelitis, left femur	Arthritis Joint Lower Limb	Nociceptive Pain
M86159	Other acute osteomyelitis, unspecified femur	Arthritis Joint Lower Limb	Nociceptive Pain
M8616	Other acute osteomyelitis, tibia and fibula	Arthritis Joint Lower Limb	Nociceptive Pain
M86161	Other acute osteomyelitis, right tibia and fibula	Arthritis Joint Lower Limb	Nociceptive Pain
M86162	Other acute osteomyelitis, left tibia and fibula	Arthritis Joint Lower Limb	Nociceptive Pain
M86169	Other acute osteomyelitis, unspecified tibia and fibula	Arthritis Joint Lower Limb	Nociceptive Pain
M8617	Other acute osteomyelitis, ankle and foot	Arthritis Joint Lower Limb	Nociceptive Pain
M86171	Other acute osteomyelitis, right ankle and foot	Arthritis Joint Lower Limb	Nociceptive Pain
M86172	Other acute osteomyelitis, left ankle and foot	Arthritis Joint Lower Limb	Nociceptive Pain
M86179	Other acute osteomyelitis, unspecified ankle and foot	Arthritis Joint Lower Limb	Nociceptive Pain
M8618	Other acute osteomyelitis, other site	Arthritis Joint Other	Nociceptive Pain
M8619	Other acute osteomyelitis, multiple sites	Arthritis Joint Other	Nociceptive Pain
M862	Subacute osteomyelitis	Arthritis Joint Other	Nociceptive Pain
M8620	Subacute osteomyelitis, unspecified site	Arthritis Joint Other	Nociceptive Pain
M8621	Subacute osteomyelitis, shoulder	Arthritis Joint Upper Limb	Nociceptive Pain
M86211	Subacute osteomyelitis, right shoulder	Arthritis Joint Upper Limb	Nociceptive Pain
M86212	Subacute osteomyelitis, left shoulder	Arthritis Joint Upper Limb	Nociceptive Pain
M86219	Subacute osteomyelitis, unspecified shoulder	Arthritis Joint Upper Limb	Nociceptive Pain

© 2024 Peterson MD et al. *JAMA Neurology.*

M8622	Subacute osteomyelitis, humerus	Arthritis Joint Upper Limb	Nociceptive Pain
M86221	Subacute osteomyelitis, right humerus	Arthritis Joint Upper Limb	Nociceptive Pain
M86222	Subacute osteomyelitis, left humerus	Arthritis Joint Upper Limb	Nociceptive Pain
M86229	Subacute osteomyelitis, unspecified humerus	Arthritis Joint Upper Limb	Nociceptive Pain
M8623	Subacute osteomyelitis, radius and ulna	Arthritis Joint Upper Limb	Nociceptive Pain
M86231	Subacute osteomyelitis, right radius and ulna	Arthritis Joint Upper Limb	Nociceptive Pain
M86232	Subacute osteomyelitis, left radius and ulna	Arthritis Joint Upper Limb	Nociceptive Pain
M86239	Subacute osteomyelitis, unspecified radius and ulna	Arthritis Joint Upper Limb	Nociceptive Pain
M8624	Subacute osteomyelitis, hand	Arthritis Joint Upper Limb	Nociceptive Pain
M86241	Subacute osteomyelitis, right hand	Arthritis Joint Upper Limb	Nociceptive Pain
M86242	Subacute osteomyelitis, left hand	Arthritis Joint Upper Limb	Nociceptive Pain
M86249	Subacute osteomyelitis, unspecified hand	Arthritis Joint Upper Limb	Nociceptive Pain
M8625	Subacute osteomyelitis, femur	Arthritis Joint Lower Limb	Nociceptive Pain
M86251	Subacute osteomyelitis, right femur	Arthritis Joint Lower Limb	Nociceptive Pain
M86252	Subacute osteomyelitis, left femur	Arthritis Joint Lower Limb	Nociceptive Pain
M86259	Subacute osteomyelitis, unspecified femur	Arthritis Joint Lower Limb	Nociceptive Pain
M8626	Subacute osteomyelitis, tibia and fibula	Arthritis Joint Lower Limb	Nociceptive Pain
M86261	Subacute osteomyelitis, right tibia and fibula	Arthritis Joint Lower Limb	Nociceptive Pain
M86262	Subacute osteomyelitis, left tibia and fibula	Arthritis Joint Lower Limb	Nociceptive Pain
M86269	Subacute osteomyelitis, unspecified tibia and fibula	Arthritis Joint Lower Limb	Nociceptive Pain
M8627	Subacute osteomyelitis, ankle and foot	Arthritis Joint Lower Limb	Nociceptive Pain
M86271	Subacute osteomyelitis, right ankle and foot	Arthritis Joint Lower Limb	Nociceptive Pain
M86272	Subacute osteomyelitis, left ankle and foot	Arthritis Joint Lower Limb	Nociceptive Pain
M86279	Subacute osteomyelitis, unspecified ankle and foot	Arthritis Joint Lower Limb	Nociceptive Pain
M8628	Subacute osteomyelitis, other site	Arthritis Joint Other	Nociceptive Pain
M8629	Subacute osteomyelitis, multiple sites	Arthritis Joint Other	Nociceptive Pain
M863	Chronic multifocal osteomyelitis	Arthritis Joint Other	Nociceptive Pain
M8630	Chronic multifocal osteomyelitis, unspecified site	Arthritis Joint Other	Nociceptive Pain
M8631	Chronic multifocal osteomyelitis, shoulder	Arthritis Joint Upper Limb	Nociceptive Pain
M86311	Chronic multifocal osteomyelitis, right shoulder	Arthritis Joint Upper Limb	Nociceptive Pain
M86312	Chronic multifocal osteomyelitis, left shoulder	Arthritis Joint Upper Limb	Nociceptive Pain
M86319	Chronic multifocal osteomyelitis, unspecified shoulder	Arthritis Joint Upper Limb	Nociceptive Pain
M8632	Chronic multifocal osteomyelitis, humerus	Arthritis Joint Upper Limb	Nociceptive Pain
M86321	Chronic multifocal osteomyelitis, right humerus	Arthritis Joint Upper Limb	Nociceptive Pain
M86322	Chronic multifocal osteomyelitis, left humerus	Arthritis Joint Upper Limb	Nociceptive Pain
M86329	Chronic multifocal osteomyelitis, unspecified humerus	Arthritis Joint Upper Limb	Nociceptive Pain
M8633	Chronic multifocal osteomyelitis, radius and ulna	Arthritis Joint Upper Limb	Nociceptive Pain

© 2024 Peterson MD et al. *JAMA Neurology.*

M86331	Chronic multifocal osteomyelitis, right radius and ulna	Arthritis Joint Upper Limb	Nociceptive Pain
M86332	Chronic multifocal osteomyelitis, left radius and ulna	Arthritis Joint Upper Limb	Nociceptive Pain
M86339	Chronic multifocal osteomyelitis, unsp radius and ulna	Arthritis Joint Upper Limb	Nociceptive Pain
M8634	Chronic multifocal osteomyelitis, hand	Arthritis Joint Upper Limb	Nociceptive Pain
M86341	Chronic multifocal osteomyelitis, right hand	Arthritis Joint Upper Limb	Nociceptive Pain
M86342	Chronic multifocal osteomyelitis, left hand	Arthritis Joint Upper Limb	Nociceptive Pain
M86349	Chronic multifocal osteomyelitis, unspecified hand	Arthritis Joint Upper Limb	Nociceptive Pain
M8635	Chronic multifocal osteomyelitis, femur	Arthritis Joint Lower Limb	Nociceptive Pain
M86351	Chronic multifocal osteomyelitis, right femur	Arthritis Joint Lower Limb	Nociceptive Pain
M86352	Chronic multifocal osteomyelitis, left femur	Arthritis Joint Lower Limb	Nociceptive Pain
M86359	Chronic multifocal osteomyelitis, unspecified femur	Arthritis Joint Lower Limb	Nociceptive Pain
M8636	Chronic multifocal osteomyelitis, tibia and fibula	Arthritis Joint Lower Limb	Nociceptive Pain
M86361	Chronic multifocal osteomyelitis, right tibia and fibula	Arthritis Joint Lower Limb	Nociceptive Pain
M86362	Chronic multifocal osteomyelitis, left tibia and fibula	Arthritis Joint Lower Limb	Nociceptive Pain
M86369	Chronic multifocal osteomyelitis, unsp tibia and fibula	Arthritis Joint Lower Limb	Nociceptive Pain
M8637	Chronic multifocal osteomyelitis, ankle and foot	Arthritis Joint Lower Limb	Nociceptive Pain
M86371	Chronic multifocal osteomyelitis, right ankle and foot	Arthritis Joint Lower Limb	Nociceptive Pain
M86372	Chronic multifocal osteomyelitis, left ankle and foot	Arthritis Joint Lower Limb	Nociceptive Pain
M86379	Chronic multifocal osteomyelitis, unspecified ankle and foot	Arthritis Joint Lower Limb	Nociceptive Pain
M8638	Chronic multifocal osteomyelitis, other site	Arthritis Joint Other	Nociceptive Pain
M8639	Chronic multifocal osteomyelitis, multiple sites	Arthritis Joint Other	Nociceptive Pain
M864	Chronic osteomyelitis with draining sinus	Arthritis Joint Other	Nociceptive Pain
M8640	Chronic osteomyelitis with draining sinus, unspecified site	Arthritis Joint Other	Nociceptive Pain
M8641	Chronic osteomyelitis with draining sinus, shoulder	Arthritis Joint Upper Limb	Nociceptive Pain
M86411	Chronic osteomyelitis with draining sinus, right shoulder	Arthritis Joint Upper Limb	Nociceptive Pain
M86412	Chronic osteomyelitis with draining sinus, left shoulder	Arthritis Joint Upper Limb	Nociceptive Pain
M86419	Chronic osteomyelitis with draining sinus, unsp shoulder	Arthritis Joint Upper Limb	Nociceptive Pain
M8642	Chronic osteomyelitis with draining sinus, humerus	Arthritis Joint Upper Limb	Nociceptive Pain
M86421	Chronic osteomyelitis with draining sinus, right humerus	Arthritis Joint Upper Limb	Nociceptive Pain
M86422	Chronic osteomyelitis with draining sinus, left humerus	Arthritis Joint Upper Limb	Nociceptive Pain
M86429	Chronic osteomyelitis with draining sinus, unsp humerus	Arthritis Joint Upper Limb	Nociceptive Pain
M8643	Chronic osteomyelitis with draining sinus, radius and ulna	Arthritis Joint Upper Limb	Nociceptive Pain
M86431	Chronic osteomyelit w draining sinus, right radius and ulna	Arthritis Joint Upper Limb	Nociceptive Pain
M86432	Chronic osteomyelitis w draining sinus, left radius and ulna	Arthritis Joint Upper Limb	Nociceptive Pain
M86439	Chronic osteomyelitis w draining sinus, unsp radius and ulna	Arthritis Joint Upper Limb	Nociceptive Pain
M8644	Chronic osteomyelitis with draining sinus, hand	Arthritis Joint Upper Limb	Nociceptive Pain
M86441	Chronic osteomyelitis with draining sinus, right hand	Arthritis Joint Upper Limb	Nociceptive Pain

© 2024 Peterson MD et al. *JAMA Neurology.*

M86442	Chronic osteomyelitis with draining sinus, left hand	Arthritis Joint Upper Limb	Nociceptive Pain
M86449	Chronic osteomyelitis with draining sinus, unspecified hand	Arthritis Joint Upper Limb	Nociceptive Pain
M8645	Chronic osteomyelitis with draining sinus, femur	Arthritis Joint Lower Limb	Nociceptive Pain
M86451	Chronic osteomyelitis with draining sinus, right femur	Arthritis Joint Lower Limb	Nociceptive Pain
M86452	Chronic osteomyelitis with draining sinus, left femur	Arthritis Joint Lower Limb	Nociceptive Pain
M86459	Chronic osteomyelitis with draining sinus, unspecified femur	Arthritis Joint Lower Limb	Nociceptive Pain
M8646	Chronic osteomyelitis with draining sinus, tibia and fibula	Arthritis Joint Lower Limb	Nociceptive Pain
M86461	Chronic osteomyelit w draining sinus, right tibia and fibula	Arthritis Joint Lower Limb	Nociceptive Pain
M86462	Chronic osteomyelit w draining sinus, left tibia and fibula	Arthritis Joint Lower Limb	Nociceptive Pain
M86469	Chronic osteomyelit w draining sinus, unsp tibia and fibula	Arthritis Joint Lower Limb	Nociceptive Pain
M8647	Chronic osteomyelitis with draining sinus, ankle and foot	Arthritis Joint Lower Limb	Nociceptive Pain
M86471	Chronic osteomyelitis w draining sinus, right ankle and foot	Arthritis Joint Lower Limb	Nociceptive Pain
M86472	Chronic osteomyelitis w draining sinus, left ankle and foot	Arthritis Joint Lower Limb	Nociceptive Pain
M86479	Chronic osteomyelitis w draining sinus, unsp ankle and foot	Arthritis Joint Lower Limb	Nociceptive Pain
M8648	Chronic osteomyelitis with draining sinus, other site	Arthritis Joint Other	Nociceptive Pain
M8649	Chronic osteomyelitis with draining sinus, multiple sites	Arthritis Joint Other	Nociceptive Pain
M865	Other chronic hematogenous osteomyelitis	Arthritis Joint Other	Nociceptive Pain
M8650	Other chronic hematogenous osteomyelitis, unspecified site	Arthritis Joint Other	Nociceptive Pain
M8651	Other chronic hematogenous osteomyelitis, shoulder	Arthritis Joint Upper Limb	Nociceptive Pain
M86511	Other chronic hematogenous osteomyelitis, right shoulder	Arthritis Joint Upper Limb	Nociceptive Pain
M86512	Other chronic hematogenous osteomyelitis, left shoulder	Arthritis Joint Upper Limb	Nociceptive Pain
M86519	Other chronic hematogenous osteomyelitis, unsp shoulder	Arthritis Joint Upper Limb	Nociceptive Pain
M8652	Other chronic hematogenous osteomyelitis, humerus	Arthritis Joint Upper Limb	Nociceptive Pain
M86521	Other chronic hematogenous osteomyelitis, right humerus	Arthritis Joint Upper Limb	Nociceptive Pain
M86522	Other chronic hematogenous osteomyelitis, left humerus	Arthritis Joint Upper Limb	Nociceptive Pain
M86529	Other chronic hematogenous osteomyelitis, unsp humerus	Arthritis Joint Upper Limb	Nociceptive Pain
M8653	Other chronic hematogenous osteomyelitis, radius and ulna	Arthritis Joint Upper Limb	Nociceptive Pain
M86531	Oth chronic hematogenous osteomyelit, right radius and ulna	Arthritis Joint Upper Limb	Nociceptive Pain
M86532	Oth chronic hematogenous osteomyelitis, left radius and ulna	Arthritis Joint Upper Limb	Nociceptive Pain
M86539	Oth chronic hematogenous osteomyelitis, unsp radius and ulna	Arthritis Joint Upper Limb	Nociceptive Pain
M8654	Other chronic hematogenous osteomyelitis, hand	Arthritis Joint Upper Limb	Nociceptive Pain
M86541	Other chronic hematogenous osteomyelitis, right hand	Arthritis Joint Upper Limb	Nociceptive Pain
M86542	Other chronic hematogenous osteomyelitis, left hand	Arthritis Joint Upper Limb	Nociceptive Pain
M86549	Other chronic hematogenous osteomyelitis, unspecified hand	Arthritis Joint Upper Limb	Nociceptive Pain
M8655	Other chronic hematogenous osteomyelitis, femur	Arthritis Joint Lower Limb	Nociceptive Pain
M86551	Other chronic hematogenous osteomyelitis, right femur	Arthritis Joint Lower Limb	Nociceptive Pain
M86552	Other chronic hematogenous osteomyelitis, left femur	Arthritis Joint Lower Limb	Nociceptive Pain

© 2024 Peterson MD et al. *JAMA Neurology.*

M86559	Other chronic hematogenous osteomyelitis, unspecified femur	Arthritis Joint Lower Limb	Nociceptive Pain
M8656	Other chronic hematogenous osteomyelitis, tibia and fibula	Arthritis Joint Lower Limb	Nociceptive Pain
M86561	Oth chronic hematogenous osteomyelit, right tibia and fibula	Arthritis Joint Lower Limb	Nociceptive Pain
M86562	Oth chronic hematogenous osteomyelit, left tibia and fibula	Arthritis Joint Lower Limb	Nociceptive Pain
M86569	Oth chronic hematogenous osteomyelit, unsp tibia and fibula	Arthritis Joint Lower Limb	Nociceptive Pain
M8657	Other chronic hematogenous osteomyelitis, ankle and foot	Arthritis Joint Lower Limb	Nociceptive Pain
M86571	Oth chronic hematogenous osteomyelitis, right ankle and foot	Arthritis Joint Lower Limb	Nociceptive Pain
M86572	Oth chronic hematogenous osteomyelitis, left ankle and foot	Arthritis Joint Lower Limb	Nociceptive Pain
M86579	Oth chronic hematogenous osteomyelitis, unsp ankle and foot	Arthritis Joint Lower Limb	Nociceptive Pain
M8658	Other chronic hematogenous osteomyelitis, other site	Arthritis Joint Other	Nociceptive Pain
M8659	Other chronic hematogenous osteomyelitis, multiple sites	Arthritis Joint Other	Nociceptive Pain
M866	Other chronic osteomyelitis	Arthritis Joint Other	Nociceptive Pain
M8660	Other chronic osteomyelitis, unspecified site	Arthritis Joint Other	Nociceptive Pain
M8661	Other chronic osteomyelitis, shoulder	Arthritis Joint Upper Limb	Nociceptive Pain
M86611	Other chronic osteomyelitis, right shoulder	Arthritis Joint Upper Limb	Nociceptive Pain
M86612	Other chronic osteomyelitis, left shoulder	Arthritis Joint Upper Limb	Nociceptive Pain
M86619	Other chronic osteomyelitis, unspecified shoulder	Arthritis Joint Upper Limb	Nociceptive Pain
M8662	Other chronic osteomyelitis, humerus	Arthritis Joint Upper Limb	Nociceptive Pain
M86621	Other chronic osteomyelitis, right humerus	Arthritis Joint Upper Limb	Nociceptive Pain
M86622	Other chronic osteomyelitis, left humerus	Arthritis Joint Upper Limb	Nociceptive Pain
M86629	Other chronic osteomyelitis, unspecified humerus	Arthritis Joint Upper Limb	Nociceptive Pain
M8663	Other chronic osteomyelitis, radius and ulna	Arthritis Joint Upper Limb	Nociceptive Pain
M86631	Other chronic osteomyelitis, right radius and ulna	Arthritis Joint Upper Limb	Nociceptive Pain
M86632	Other chronic osteomyelitis, left radius and ulna	Arthritis Joint Upper Limb	Nociceptive Pain
M86639	Other chronic osteomyelitis, unspecified radius and ulna	Arthritis Joint Upper Limb	Nociceptive Pain
M8664	Other chronic osteomyelitis, hand	Arthritis Joint Upper Limb	Nociceptive Pain
M86641	Other chronic osteomyelitis, right hand	Arthritis Joint Upper Limb	Nociceptive Pain
M86642	Other chronic osteomyelitis, left hand	Arthritis Joint Upper Limb	Nociceptive Pain
M86649	Other chronic osteomyelitis, unspecified hand	Arthritis Joint Upper Limb	Nociceptive Pain
M8665	Other chronic osteomyelitis, thigh	Arthritis Joint Lower Limb	Nociceptive Pain
M86651	Other chronic osteomyelitis, right thigh	Arthritis Joint Lower Limb	Nociceptive Pain
M86652	Other chronic osteomyelitis, left thigh	Arthritis Joint Lower Limb	Nociceptive Pain
M86659	Other chronic osteomyelitis, unspecified thigh	Arthritis Joint Lower Limb	Nociceptive Pain
M8666	Other chronic osteomyelitis, tibia and fibula	Arthritis Joint Lower Limb	Nociceptive Pain
M86661	Other chronic osteomyelitis, right tibia and fibula	Arthritis Joint Lower Limb	Nociceptive Pain
M86662	Other chronic osteomyelitis, left tibia and fibula	Arthritis Joint Lower Limb	Nociceptive Pain
M86669	Other chronic osteomyelitis, unspecified tibia and fibula	Arthritis Joint Lower Limb	Nociceptive Pain

© 2024 Peterson MD et al. *JAMA Neurology.*

M8667	Other chronic osteomyelitis, ankle and foot	Arthritis Joint Lower Limb	Nociceptive Pain
M86671	Other chronic osteomyelitis, right ankle and foot	Arthritis Joint Lower Limb	Nociceptive Pain
M86672	Other chronic osteomyelitis, left ankle and foot	Arthritis Joint Lower Limb	Nociceptive Pain
M86679	Other chronic osteomyelitis, unspecified ankle and foot	Arthritis Joint Lower Limb	Nociceptive Pain
M8668	Other chronic osteomyelitis, other site	Arthritis Joint Other	Nociceptive Pain
M8669	Other chronic osteomyelitis, multiple sites	Arthritis Joint Other	Nociceptive Pain
M868	Other osteomyelitis	Arthritis Joint Other	Nociceptive Pain
M868X	Other osteomyelitis	Arthritis Joint Other	Nociceptive Pain
M868X0	Other osteomyelitis, multiple sites	Arthritis Joint Other	Nociceptive Pain
M868X1	Other osteomyelitis, shoulder	Arthritis Joint Upper Limb	Nociceptive Pain
M868X2	Other osteomyelitis, upper arm	Arthritis Joint Upper Limb	Nociceptive Pain
M868X3	Other osteomyelitis, forearm	Arthritis Joint Upper Limb	Nociceptive Pain
M868X4	Other osteomyelitis, hand	Arthritis Joint Upper Limb	Nociceptive Pain
M868X5	Other osteomyelitis, thigh	Arthritis Joint Lower Limb	Nociceptive Pain
M868X6	Other osteomyelitis, lower leg	Arthritis Joint Lower Limb	Nociceptive Pain
M868X7	Other osteomyelitis, ankle and foot	Arthritis Joint Lower Limb	Nociceptive Pain
M868X8	Other osteomyelitis, other site	Arthritis Joint Other	Nociceptive Pain
M868X9	Other osteomyelitis, unspecified sites	Arthritis Joint Other	Nociceptive Pain
M869	Osteomyelitis, unspecified	Arthritis Joint Other	Nociceptive Pain
M87	Osteonecrosis	Arthritis Joint Other	Nociceptive Pain
M870	Idiopathic aseptic necrosis of bone	Arthritis Joint Other	Nociceptive Pain
M8700	Idiopathic aseptic necrosis of unspecified bone	Arthritis Joint Other	Nociceptive Pain
M8701	Idiopathic aseptic necrosis of shoulder	Arthritis Joint Upper Limb	Nociceptive Pain
M87011	Idiopathic aseptic necrosis of right shoulder	Arthritis Joint Upper Limb	Nociceptive Pain
M87012	Idiopathic aseptic necrosis of left shoulder	Arthritis Joint Upper Limb	Nociceptive Pain
M87019	Idiopathic aseptic necrosis of unspecified shoulder	Arthritis Joint Upper Limb	Nociceptive Pain
M8702	Idiopathic aseptic necrosis of humerus	Arthritis Joint Upper Limb	Nociceptive Pain
M87021	Idiopathic aseptic necrosis of right humerus	Arthritis Joint Upper Limb	Nociceptive Pain
M87022	Idiopathic aseptic necrosis of left humerus	Arthritis Joint Upper Limb	Nociceptive Pain
M87029	Idiopathic aseptic necrosis of unspecified humerus	Arthritis Joint Upper Limb	Nociceptive Pain
M8703	Idiopathic aseptic necrosis of radius, ulna and carpus	Arthritis Joint Upper Limb	Nociceptive Pain
M87031	Idiopathic aseptic necrosis of right radius	Arthritis Joint Upper Limb	Nociceptive Pain
M87032	Idiopathic aseptic necrosis of left radius	Arthritis Joint Upper Limb	Nociceptive Pain
M87033	Idiopathic aseptic necrosis of unspecified radius	Arthritis Joint Upper Limb	Nociceptive Pain
M87034	Idiopathic aseptic necrosis of right ulna	Arthritis Joint Upper Limb	Nociceptive Pain
M87035	Idiopathic aseptic necrosis of left ulna	Arthritis Joint Upper Limb	Nociceptive Pain
M87036	Idiopathic aseptic necrosis of unspecified ulna	Arthritis Joint Upper Limb	Nociceptive Pain

© 2024 Peterson MD et al. *JAMA Neurology.*

M87037	Idiopathic aseptic necrosis of right carpus	Arthritis Joint Upper Limb	Nociceptive Pain
M87038	Idiopathic aseptic necrosis of left carpus	Arthritis Joint Upper Limb	Nociceptive Pain
M87039	Idiopathic aseptic necrosis of unspecified carpus	Arthritis Joint Upper Limb	Nociceptive Pain
M8704	Idiopathic aseptic necrosis of hand and fingers	Arthritis Joint Upper Limb	Nociceptive Pain
M87041	Idiopathic aseptic necrosis of right hand	Arthritis Joint Upper Limb	Nociceptive Pain
M87042	Idiopathic aseptic necrosis of left hand	Arthritis Joint Upper Limb	Nociceptive Pain
M87043	Idiopathic aseptic necrosis of unspecified hand	Arthritis Joint Upper Limb	Nociceptive Pain
M87044	Idiopathic aseptic necrosis of right finger(s)	Arthritis Joint Upper Limb	Nociceptive Pain
M87045	Idiopathic aseptic necrosis of left finger(s)	Arthritis Joint Upper Limb	Nociceptive Pain
M87046	Idiopathic aseptic necrosis of unspecified finger(s)	Arthritis Joint Upper Limb	Nociceptive Pain
M8705	Idiopathic aseptic necrosis of pelvis and femur	Arthritis Joint Lower Limb	Nociceptive Pain
M87050	Idiopathic aseptic necrosis of pelvis	Arthritis Joint Spine and Hips	Nociceptive Pain
M87051	Idiopathic aseptic necrosis of right femur	Arthritis Joint Lower Limb	Nociceptive Pain
M87052	Idiopathic aseptic necrosis of left femur	Arthritis Joint Lower Limb	Nociceptive Pain
M87059	Idiopathic aseptic necrosis of unspecified femur	Arthritis Joint Lower Limb	Nociceptive Pain
M8706	Idiopathic aseptic necrosis of tibia and fibula	Arthritis Joint Lower Limb	Nociceptive Pain
M87061	Idiopathic aseptic necrosis of right tibia	Arthritis Joint Lower Limb	Nociceptive Pain
M87062	Idiopathic aseptic necrosis of left tibia	Arthritis Joint Lower Limb	Nociceptive Pain
M87063	Idiopathic aseptic necrosis of unspecified tibia	Arthritis Joint Lower Limb	Nociceptive Pain
M87064	Idiopathic aseptic necrosis of right fibula	Arthritis Joint Lower Limb	Nociceptive Pain
M87065	Idiopathic aseptic necrosis of left fibula	Arthritis Joint Lower Limb	Nociceptive Pain
M87066	Idiopathic aseptic necrosis of unspecified fibula	Arthritis Joint Lower Limb	Nociceptive Pain
M8707	Idiopathic aseptic necrosis of ankle, foot and toes	Arthritis Joint Lower Limb	Nociceptive Pain
M87071	Idiopathic aseptic necrosis of right ankle	Arthritis Joint Lower Limb	Nociceptive Pain
M87072	Idiopathic aseptic necrosis of left ankle	Arthritis Joint Lower Limb	Nociceptive Pain
M87073	Idiopathic aseptic necrosis of unspecified ankle	Arthritis Joint Lower Limb	Nociceptive Pain
M87074	Idiopathic aseptic necrosis of right foot	Arthritis Joint Lower Limb	Nociceptive Pain
M87075	Idiopathic aseptic necrosis of left foot	Arthritis Joint Lower Limb	Nociceptive Pain
M87076	Idiopathic aseptic necrosis of unspecified foot	Arthritis Joint Lower Limb	Nociceptive Pain
M87077	Idiopathic aseptic necrosis of right toe(s)	Arthritis Joint Lower Limb	Nociceptive Pain
M87078	Idiopathic aseptic necrosis of left toe(s)	Arthritis Joint Lower Limb	Nociceptive Pain
M87079	Idiopathic aseptic necrosis of unspecified toe(s)	Arthritis Joint Lower Limb	Nociceptive Pain
M8708	Idiopathic aseptic necrosis of bone, other site	Arthritis Joint Other	Nociceptive Pain
M8709	Idiopathic aseptic necrosis of bone, multiple sites	Arthritis Joint Other	Nociceptive Pain
M871	Osteonecrosis due to drugs	Arthritis Joint Other	Nociceptive Pain
M8710	Osteonecrosis due to drugs, unspecified bone	Arthritis Joint Other	Nociceptive Pain
M8711	Osteonecrosis due to drugs, shoulder	Arthritis Joint Upper Limb	Nociceptive Pain

© 2024 Peterson MD et al. *JAMA Neurology.*

M87111	Osteonecrosis due to drugs, right shoulder	Arthritis Joint Upper Limb	Nociceptive Pain
M87112	Osteonecrosis due to drugs, left shoulder	Arthritis Joint Upper Limb	Nociceptive Pain
M87119	Osteonecrosis due to drugs, unspecified shoulder	Arthritis Joint Upper Limb	Nociceptive Pain
M8712	Osteonecrosis due to drugs, humerus	Arthritis Joint Upper Limb	Nociceptive Pain
M87121	Osteonecrosis due to drugs, right humerus	Arthritis Joint Upper Limb	Nociceptive Pain
M87122	Osteonecrosis due to drugs, left humerus	Arthritis Joint Upper Limb	Nociceptive Pain
M87129	Osteonecrosis due to drugs, unspecified humerus	Arthritis Joint Upper Limb	Nociceptive Pain
M8713	Osteonecrosis due to drugs of radius, ulna and carpus	Arthritis Joint Upper Limb	Nociceptive Pain
M87131	Osteonecrosis due to drugs of right radius	Arthritis Joint Upper Limb	Nociceptive Pain
M87132	Osteonecrosis due to drugs of left radius	Arthritis Joint Upper Limb	Nociceptive Pain
M87133	Osteonecrosis due to drugs of unspecified radius	Arthritis Joint Upper Limb	Nociceptive Pain
M87134	Osteonecrosis due to drugs of right ulna	Arthritis Joint Upper Limb	Nociceptive Pain
M87135	Osteonecrosis due to drugs of left ulna	Arthritis Joint Upper Limb	Nociceptive Pain
M87136	Osteonecrosis due to drugs of unspecified ulna	Arthritis Joint Upper Limb	Nociceptive Pain
M87137	Osteonecrosis due to drugs of right carpus	Arthritis Joint Upper Limb	Nociceptive Pain
M87138	Osteonecrosis due to drugs of left carpus	Arthritis Joint Upper Limb	Nociceptive Pain
M87139	Osteonecrosis due to drugs of unspecified carpus	Arthritis Joint Upper Limb	Nociceptive Pain
M8714	Osteonecrosis due to drugs, hand and fingers	Arthritis Joint Upper Limb	Nociceptive Pain
M87141	Osteonecrosis due to drugs, right hand	Arthritis Joint Upper Limb	Nociceptive Pain
M87142	Osteonecrosis due to drugs, left hand	Arthritis Joint Upper Limb	Nociceptive Pain
M87143	Osteonecrosis due to drugs, unspecified hand	Arthritis Joint Upper Limb	Nociceptive Pain
M87144	Osteonecrosis due to drugs, right finger(s)	Arthritis Joint Upper Limb	Nociceptive Pain
M87145	Osteonecrosis due to drugs, left finger(s)	Arthritis Joint Upper Limb	Nociceptive Pain
M87146	Osteonecrosis due to drugs, unspecified finger(s)	Arthritis Joint Upper Limb	Nociceptive Pain
M8715	Osteonecrosis due to drugs, pelvis and femur	Arthritis Joint Lower Limb	Nociceptive Pain
M87150	Osteonecrosis due to drugs, pelvis	Arthritis Joint Spine and Hips	Nociceptive Pain
M87151	Osteonecrosis due to drugs, right femur	Arthritis Joint Lower Limb	Nociceptive Pain
M87152	Osteonecrosis due to drugs, left femur	Arthritis Joint Lower Limb	Nociceptive Pain
M87159	Osteonecrosis due to drugs, unspecified femur	Arthritis Joint Lower Limb	Nociceptive Pain
M8716	Osteonecrosis due to drugs, tibia and fibula	Arthritis Joint Lower Limb	Nociceptive Pain
M87161	Osteonecrosis due to drugs, right tibia	Arthritis Joint Lower Limb	Nociceptive Pain
M87162	Osteonecrosis due to drugs, left tibia	Arthritis Joint Lower Limb	Nociceptive Pain
M87163	Osteonecrosis due to drugs, unspecified tibia	Arthritis Joint Lower Limb	Nociceptive Pain
M87164	Osteonecrosis due to drugs, right fibula	Arthritis Joint Lower Limb	Nociceptive Pain
M87165	Osteonecrosis due to drugs, left fibula	Arthritis Joint Lower Limb	Nociceptive Pain
M87166	Osteonecrosis due to drugs, unspecified fibula	Arthritis Joint Lower Limb	Nociceptive Pain
M8717	Osteonecrosis due to drugs, ankle, foot and toes	Arthritis Joint Lower Limb	Nociceptive Pain

© 2024 Peterson MD et al. *JAMA Neurology.*

M87171	Osteonecrosis due to drugs, right ankle	Arthritis Joint Lower Limb	Nociceptive Pain
M87172	Osteonecrosis due to drugs, left ankle	Arthritis Joint Lower Limb	Nociceptive Pain
M87173	Osteonecrosis due to drugs, unspecified ankle	Arthritis Joint Lower Limb	Nociceptive Pain
M87174	Osteonecrosis due to drugs, right foot	Arthritis Joint Lower Limb	Nociceptive Pain
M87175	Osteonecrosis due to drugs, left foot	Arthritis Joint Lower Limb	Nociceptive Pain
M87176	Osteonecrosis due to drugs, unspecified foot	Arthritis Joint Lower Limb	Nociceptive Pain
M87177	Osteonecrosis due to drugs, right toe(s)	Arthritis Joint Lower Limb	Nociceptive Pain
M87178	Osteonecrosis due to drugs, left toe(s)	Arthritis Joint Lower Limb	Nociceptive Pain
M87179	Osteonecrosis due to drugs, unspecified toe(s)	Arthritis Joint Lower Limb	Nociceptive Pain
M8718	Osteonecrosis due to drugs, other site	Arthritis Joint Other	Nociceptive Pain
M87180	Osteonecrosis due to drugs, jaw	Arthritis Joint Other	Nociceptive Pain
M87188	Osteonecrosis due to drugs, other site	Arthritis Joint Other	Nociceptive Pain
M8719	Osteonecrosis due to drugs, multiple sites	Arthritis Joint Other	Nociceptive Pain
M872	Osteonecrosis due to previous trauma	Arthritis Joint Other	Nociceptive Pain
M8720	Osteonecrosis due to previous trauma, unspecified bone	Arthritis Joint Other	Nociceptive Pain
M8721	Osteonecrosis due to previous trauma, shoulder	Arthritis Joint Upper Limb	Nociceptive Pain
M87211	Osteonecrosis due to previous trauma, right shoulder	Arthritis Joint Upper Limb	Nociceptive Pain
M87212	Osteonecrosis due to previous trauma, left shoulder	Arthritis Joint Upper Limb	Nociceptive Pain
M87219	Osteonecrosis due to previous trauma, unspecified shoulder	Arthritis Joint Upper Limb	Nociceptive Pain
M8722	Osteonecrosis due to previous trauma, humerus	Arthritis Joint Upper Limb	Nociceptive Pain
M87221	Osteonecrosis due to previous trauma, right humerus	Arthritis Joint Upper Limb	Nociceptive Pain
M87222	Osteonecrosis due to previous trauma, left humerus	Arthritis Joint Upper Limb	Nociceptive Pain
M87229	Osteonecrosis due to previous trauma, unspecified humerus	Arthritis Joint Upper Limb	Nociceptive Pain
M8723	Osteonecrs due to previous trauma of radius, ulna and carpus	Arthritis Joint Upper Limb	Nociceptive Pain
M87231	Osteonecrosis due to previous trauma of right radius	Arthritis Joint Upper Limb	Nociceptive Pain
M87232	Osteonecrosis due to previous trauma of left radius	Arthritis Joint Upper Limb	Nociceptive Pain
M87233	Osteonecrosis due to previous trauma of unspecified radius	Arthritis Joint Upper Limb	Nociceptive Pain
M87234	Osteonecrosis due to previous trauma of right ulna	Arthritis Joint Upper Limb	Nociceptive Pain
M87235	Osteonecrosis due to previous trauma of left ulna	Arthritis Joint Upper Limb	Nociceptive Pain
M87236	Osteonecrosis due to previous trauma of unspecified ulna	Arthritis Joint Upper Limb	Nociceptive Pain
M87237	Osteonecrosis due to previous trauma of right carpus	Arthritis Joint Upper Limb	Nociceptive Pain
M87238	Osteonecrosis due to previous trauma of left carpus	Arthritis Joint Upper Limb	Nociceptive Pain
M87239	Osteonecrosis due to previous trauma of unspecified carpus	Arthritis Joint Upper Limb	Nociceptive Pain
M8724	Osteonecrosis due to previous trauma, hand and fingers	Arthritis Joint Upper Limb	Nociceptive Pain
M87241	Osteonecrosis due to previous trauma, right hand	Arthritis Joint Upper Limb	Nociceptive Pain
M87242	Osteonecrosis due to previous trauma, left hand	Arthritis Joint Upper Limb	Nociceptive Pain
M87243	Osteonecrosis due to previous trauma, unspecified hand	Arthritis Joint Upper Limb	Nociceptive Pain

© 2024 Peterson MD et al. *JAMA Neurology.*

M87244	Osteonecrosis due to previous trauma, right finger(s)	Arthritis Joint Upper Limb	Nociceptive Pain
M87245	Osteonecrosis due to previous trauma, left finger(s)	Arthritis Joint Upper Limb	Nociceptive Pain
M87246	Osteonecrosis due to previous trauma, unspecified finger(s)	Arthritis Joint Upper Limb	Nociceptive Pain
M8725	Osteonecrosis due to previous trauma, pelvis and femur	Arthritis Joint Lower Limb	Nociceptive Pain
M87250	Osteonecrosis due to previous trauma, pelvis	Arthritis Joint Spine and Hips	Nociceptive Pain
M87251	Osteonecrosis due to previous trauma, right femur	Arthritis Joint Lower Limb	Nociceptive Pain
M87252	Osteonecrosis due to previous trauma, left femur	Arthritis Joint Lower Limb	Nociceptive Pain
M87256	Osteonecrosis due to previous trauma, unspecified femur	Arthritis Joint Lower Limb	Nociceptive Pain
M8726	Osteonecrosis due to previous trauma, tibia and fibula	Arthritis Joint Lower Limb	Nociceptive Pain
M87261	Osteonecrosis due to previous trauma, right tibia	Arthritis Joint Lower Limb	Nociceptive Pain
M87262	Osteonecrosis due to previous trauma, left tibia	Arthritis Joint Lower Limb	Nociceptive Pain
M87263	Osteonecrosis due to previous trauma, unspecified tibia	Arthritis Joint Lower Limb	Nociceptive Pain
M87264	Osteonecrosis due to previous trauma, right fibula	Arthritis Joint Lower Limb	Nociceptive Pain
M87265	Osteonecrosis due to previous trauma, left fibula	Arthritis Joint Lower Limb	Nociceptive Pain
M87266	Osteonecrosis due to previous trauma, unspecified fibula	Arthritis Joint Lower Limb	Nociceptive Pain
M8727	Osteonecrosis due to previous trauma, ankle, foot and toes	Arthritis Joint Lower Limb	Nociceptive Pain
M87271	Osteonecrosis due to previous trauma, right ankle	Arthritis Joint Lower Limb	Nociceptive Pain
M87272	Osteonecrosis due to previous trauma, left ankle	Arthritis Joint Lower Limb	Nociceptive Pain
M87273	Osteonecrosis due to previous trauma, unspecified ankle	Arthritis Joint Lower Limb	Nociceptive Pain
M87274	Osteonecrosis due to previous trauma, right foot	Arthritis Joint Lower Limb	Nociceptive Pain
M87275	Osteonecrosis due to previous trauma, left foot	Arthritis Joint Lower Limb	Nociceptive Pain
M87276	Osteonecrosis due to previous trauma, unspecified foot	Arthritis Joint Lower Limb	Nociceptive Pain
M87277	Osteonecrosis due to previous trauma, right toe(s)	Arthritis Joint Lower Limb	Nociceptive Pain
M87278	Osteonecrosis due to previous trauma, left toe(s)	Arthritis Joint Lower Limb	Nociceptive Pain
M87279	Osteonecrosis due to previous trauma, unspecified toe(s)	Arthritis Joint Lower Limb	Nociceptive Pain
M8728	Osteonecrosis due to previous trauma, other site	Arthritis Joint Other	Nociceptive Pain
M8729	Osteonecrosis due to previous trauma, multiple sites	Arthritis Joint Other	Nociceptive Pain
M873	Other secondary osteonecrosis	Arthritis Joint Other	Nociceptive Pain
M8730	Other secondary osteonecrosis, unspecified bone	Arthritis Joint Other	Nociceptive Pain
M8731	Other secondary osteonecrosis, shoulder	Arthritis Joint Upper Limb	Nociceptive Pain
M87311	Other secondary osteonecrosis, right shoulder	Arthritis Joint Upper Limb	Nociceptive Pain
M87312	Other secondary osteonecrosis, left shoulder	Arthritis Joint Upper Limb	Nociceptive Pain
M87319	Other secondary osteonecrosis, unspecified shoulder	Arthritis Joint Upper Limb	Nociceptive Pain
M8732	Other secondary osteonecrosis, humerus	Arthritis Joint Upper Limb	Nociceptive Pain
M87321	Other secondary osteonecrosis, right humerus	Arthritis Joint Upper Limb	Nociceptive Pain
M87322	Other secondary osteonecrosis, left humerus	Arthritis Joint Upper Limb	Nociceptive Pain
M87329	Other secondary osteonecrosis, unspecified humerus	Arthritis Joint Upper Limb	Nociceptive Pain

© 2024 Peterson MD et al. *JAMA Neurology.*

M8733	Other secondary osteonecrosis of radius, ulna and carpus	Arthritis Joint Upper Limb	Nociceptive Pain
M87331	Other secondary osteonecrosis of right radius	Arthritis Joint Upper Limb	Nociceptive Pain
M87332	Other secondary osteonecrosis of left radius	Arthritis Joint Upper Limb	Nociceptive Pain
M87333	Other secondary osteonecrosis of unspecified radius	Arthritis Joint Upper Limb	Nociceptive Pain
M87334	Other secondary osteonecrosis of right ulna	Arthritis Joint Upper Limb	Nociceptive Pain
M87335	Other secondary osteonecrosis of left ulna	Arthritis Joint Upper Limb	Nociceptive Pain
M87336	Other secondary osteonecrosis of unspecified ulna	Arthritis Joint Upper Limb	Nociceptive Pain
M87337	Other secondary osteonecrosis of right carpus	Arthritis Joint Upper Limb	Nociceptive Pain
M87338	Other secondary osteonecrosis of left carpus	Arthritis Joint Upper Limb	Nociceptive Pain
M87339	Other secondary osteonecrosis of unspecified carpus	Arthritis Joint Upper Limb	Nociceptive Pain
M8734	Other secondary osteonecrosis, hand and fingers	Arthritis Joint Upper Limb	Nociceptive Pain
M87341	Other secondary osteonecrosis, right hand	Arthritis Joint Upper Limb	Nociceptive Pain
M87342	Other secondary osteonecrosis, left hand	Arthritis Joint Upper Limb	Nociceptive Pain
M87343	Other secondary osteonecrosis, unspecified hand	Arthritis Joint Upper Limb	Nociceptive Pain
M87344	Other secondary osteonecrosis, right finger(s)	Arthritis Joint Upper Limb	Nociceptive Pain
M87345	Other secondary osteonecrosis, left finger(s)	Arthritis Joint Upper Limb	Nociceptive Pain
M87346	Other secondary osteonecrosis, unspecified finger(s)	Arthritis Joint Upper Limb	Nociceptive Pain
M8735	Other secondary osteonecrosis, pelvis and femur	Arthritis Joint Lower Limb	Nociceptive Pain
M87350	Other secondary osteonecrosis, pelvis	Arthritis Joint Spine and Hips	Nociceptive Pain
M87351	Other secondary osteonecrosis, right femur	Arthritis Joint Lower Limb	Nociceptive Pain
M87352	Other secondary osteonecrosis, left femur	Arthritis Joint Lower Limb	Nociceptive Pain
M87353	Other secondary osteonecrosis, unspecified femur	Arthritis Joint Lower Limb	Nociceptive Pain
M8736	Other secondary osteonecrosis, tibia and fibula	Arthritis Joint Lower Limb	Nociceptive Pain
M87361	Other secondary osteonecrosis, right tibia	Arthritis Joint Lower Limb	Nociceptive Pain
M87362	Other secondary osteonecrosis, left tibia	Arthritis Joint Lower Limb	Nociceptive Pain
M87363	Other secondary osteonecrosis, unspecified tibia	Arthritis Joint Lower Limb	Nociceptive Pain
M87364	Other secondary osteonecrosis, right fibula	Arthritis Joint Lower Limb	Nociceptive Pain
M87365	Other secondary osteonecrosis, left fibula	Arthritis Joint Lower Limb	Nociceptive Pain
M87366	Other secondary osteonecrosis, unspecified fibula	Arthritis Joint Lower Limb	Nociceptive Pain
M8737	Other secondary osteonecrosis, ankle and foot	Arthritis Joint Lower Limb	Nociceptive Pain
M87371	Other secondary osteonecrosis, right ankle	Arthritis Joint Lower Limb	Nociceptive Pain
M87372	Other secondary osteonecrosis, left ankle	Arthritis Joint Lower Limb	Nociceptive Pain
M87373	Other secondary osteonecrosis, unspecified ankle	Arthritis Joint Lower Limb	Nociceptive Pain
M87374	Other secondary osteonecrosis, right foot	Arthritis Joint Lower Limb	Nociceptive Pain
M87375	Other secondary osteonecrosis, left foot	Arthritis Joint Lower Limb	Nociceptive Pain
M87376	Other secondary osteonecrosis, unspecified foot	Arthritis Joint Lower Limb	Nociceptive Pain
M87377	Other secondary osteonecrosis, right toe(s)	Arthritis Joint Lower Limb	Nociceptive Pain

© 2024 Peterson MD et al. *JAMA Neurology.*

M87378	Other secondary osteonecrosis, left toe(s)	Arthritis Joint Lower Limb	Nociceptive Pain
M87379	Other secondary osteonecrosis, unspecified toe(s)	Arthritis Joint Lower Limb	Nociceptive Pain
M8738	Other secondary osteonecrosis, other site	Arthritis Joint Other	Nociceptive Pain
M8739	Other secondary osteonecrosis, multiple sites	Arthritis Joint Other	Nociceptive Pain
M878	Other osteonecrosis	Arthritis Joint Other	Nociceptive Pain
M8780	Other osteonecrosis, unspecified bone	Arthritis Joint Other	Nociceptive Pain
M8781	Other osteonecrosis, shoulder	Arthritis Joint Upper Limb	Nociceptive Pain
M87811	Other osteonecrosis, right shoulder	Arthritis Joint Upper Limb	Nociceptive Pain
M87812	Other osteonecrosis, left shoulder	Arthritis Joint Upper Limb	Nociceptive Pain
M87819	Other osteonecrosis, unspecified shoulder	Arthritis Joint Upper Limb	Nociceptive Pain
M8782	Other osteonecrosis, humerus	Arthritis Joint Upper Limb	Nociceptive Pain
M87821	Other osteonecrosis, right humerus	Arthritis Joint Upper Limb	Nociceptive Pain
M87822	Other osteonecrosis, left humerus	Arthritis Joint Upper Limb	Nociceptive Pain
M87829	Other osteonecrosis, unspecified humerus	Arthritis Joint Upper Limb	Nociceptive Pain
M8783	Other osteonecrosis of radius, ulna and carpus	Arthritis Joint Upper Limb	Nociceptive Pain
M87831	Other osteonecrosis of right radius	Arthritis Joint Upper Limb	Nociceptive Pain
M87832	Other osteonecrosis of left radius	Arthritis Joint Upper Limb	Nociceptive Pain
M87833	Other osteonecrosis of unspecified radius	Arthritis Joint Upper Limb	Nociceptive Pain
M87834	Other osteonecrosis of right ulna	Arthritis Joint Upper Limb	Nociceptive Pain
M87835	Other osteonecrosis of left ulna	Arthritis Joint Upper Limb	Nociceptive Pain
M87836	Other osteonecrosis of unspecified ulna	Arthritis Joint Upper Limb	Nociceptive Pain
M87837	Other osteonecrosis of right carpus	Arthritis Joint Upper Limb	Nociceptive Pain
M87838	Other osteonecrosis of left carpus	Arthritis Joint Upper Limb	Nociceptive Pain
M87839	Other osteonecrosis of unspecified carpus	Arthritis Joint Upper Limb	Nociceptive Pain
M8784	Other osteonecrosis, hand and fingers	Arthritis Joint Upper Limb	Nociceptive Pain
M87841	Other osteonecrosis, right hand	Arthritis Joint Upper Limb	Nociceptive Pain
M87842	Other osteonecrosis, left hand	Arthritis Joint Upper Limb	Nociceptive Pain
M87843	Other osteonecrosis, unspecified hand	Arthritis Joint Upper Limb	Nociceptive Pain
M87844	Other osteonecrosis, right finger(s)	Arthritis Joint Upper Limb	Nociceptive Pain
M87845	Other osteonecrosis, left finger(s)	Arthritis Joint Upper Limb	Nociceptive Pain
M87849	Other osteonecrosis, unspecified finger(s)	Arthritis Joint Upper Limb	Nociceptive Pain
M8785	Other osteonecrosis, pelvis and femur	Arthritis Joint Lower Limb	Nociceptive Pain
M87850	Other osteonecrosis, pelvis	Arthritis Joint Spine and Hips	Nociceptive Pain
M87851	Other osteonecrosis, right femur	Arthritis Joint Lower Limb	Nociceptive Pain
M87852	Other osteonecrosis, left femur	Arthritis Joint Lower Limb	Nociceptive Pain
M87859	Other osteonecrosis, unspecified femur	Arthritis Joint Lower Limb	Nociceptive Pain
M8786	Other osteonecrosis, tibia and fibula	Arthritis Joint Lower Limb	Nociceptive Pain

© 2024 Peterson MD et al. *JAMA Neurology.*

M87861	Other osteonecrosis, right tibia	Arthritis Joint Lower Limb	Nociceptive Pain
M87862	Other osteonecrosis, left tibia	Arthritis Joint Lower Limb	Nociceptive Pain
M87863	Other osteonecrosis, unspecified tibia	Arthritis Joint Lower Limb	Nociceptive Pain
M87864	Other osteonecrosis, right fibula	Arthritis Joint Lower Limb	Nociceptive Pain
M87865	Other osteonecrosis, left fibula	Arthritis Joint Lower Limb	Nociceptive Pain
M87869	Other osteonecrosis, unspecified fibula	Arthritis Joint Lower Limb	Nociceptive Pain
M8787	Other osteonecrosis, ankle, foot and toes	Arthritis Joint Lower Limb	Nociceptive Pain
M87871	Other osteonecrosis, right ankle	Arthritis Joint Lower Limb	Nociceptive Pain
M87872	Other osteonecrosis, left ankle	Arthritis Joint Lower Limb	Nociceptive Pain
M87873	Other osteonecrosis, unspecified ankle	Arthritis Joint Lower Limb	Nociceptive Pain
M87874	Other osteonecrosis, right foot	Arthritis Joint Lower Limb	Nociceptive Pain
M87875	Other osteonecrosis, left foot	Arthritis Joint Lower Limb	Nociceptive Pain
M87876	Other osteonecrosis, unspecified foot	Arthritis Joint Lower Limb	Nociceptive Pain
M87877	Other osteonecrosis, right toe(s)	Arthritis Joint Lower Limb	Nociceptive Pain
M87878	Other osteonecrosis, left toe(s)	Arthritis Joint Lower Limb	Nociceptive Pain
M87879	Other osteonecrosis, unspecified toe(s)	Arthritis Joint Lower Limb	Nociceptive Pain
M8788	Other osteonecrosis, other site	Arthritis Joint Other	Nociceptive Pain
M8789	Other osteonecrosis, multiple sites	Arthritis Joint Other	Nociceptive Pain
M879	Osteonecrosis, unspecified	Arthritis Joint Other	Nociceptive Pain
M88	Osteitis deformans [Paget's disease of bone]	Arthritis Joint Other	Nociceptive Pain
M880	Osteitis deformans of skull	Arthritis Joint Other	Nociceptive Pain
M881	Osteitis deformans of vertebrae	Arthritis Joint Spine and Hips	Nociceptive Pain
M888	Osteitis deformans of other bones	Arthritis Joint Other	Nociceptive Pain
M8881	Osteitis deformans of shoulder	Arthritis Joint Upper Limb	Nociceptive Pain
M88811	Osteitis deformans of right shoulder	Arthritis Joint Upper Limb	Nociceptive Pain
M88812	Osteitis deformans of left shoulder	Arthritis Joint Upper Limb	Nociceptive Pain
M88819	Osteitis deformans of unspecified shoulder	Arthritis Joint Upper Limb	Nociceptive Pain
M8882	Osteitis deformans of upper arm	Arthritis Joint Upper Limb	Nociceptive Pain
M88821	Osteitis deformans of right upper arm	Arthritis Joint Upper Limb	Nociceptive Pain
M88822	Osteitis deformans of left upper arm	Arthritis Joint Upper Limb	Nociceptive Pain
M88829	Osteitis deformans of unspecified upper arm	Arthritis Joint Upper Limb	Nociceptive Pain
M8883	Osteitis deformans of forearm	Arthritis Joint Upper Limb	Nociceptive Pain
M88831	Osteitis deformans of right forearm	Arthritis Joint Upper Limb	Nociceptive Pain
M88832	Osteitis deformans of left forearm	Arthritis Joint Upper Limb	Nociceptive Pain
M88839	Osteitis deformans of unspecified forearm	Arthritis Joint Upper Limb	Nociceptive Pain
M8884	Osteitis deformans of hand	Arthritis Joint Upper Limb	Nociceptive Pain
M88841	Osteitis deformans of right hand	Arthritis Joint Upper Limb	Nociceptive Pain

© 2024 Peterson MD et al. *JAMA Neurology.*

M88842	Osteitis deformans of left hand	Arthritis Joint Upper Limb	Nociceptive Pain
M88849	Osteitis deformans of unspecified hand	Arthritis Joint Upper Limb	Nociceptive Pain
M8885	Osteitis deformans of thigh	Arthritis Joint Lower Limb	Nociceptive Pain
M88851	Osteitis deformans of right thigh	Arthritis Joint Lower Limb	Nociceptive Pain
M88852	Osteitis deformans of left thigh	Arthritis Joint Lower Limb	Nociceptive Pain
M88859	Osteitis deformans of unspecified thigh	Arthritis Joint Lower Limb	Nociceptive Pain
M8886	Osteitis deformans of lower leg	Arthritis Joint Lower Limb	Nociceptive Pain
M88861	Osteitis deformans of right lower leg	Arthritis Joint Lower Limb	Nociceptive Pain
M88862	Osteitis deformans of left lower leg	Arthritis Joint Lower Limb	Nociceptive Pain
M88869	Osteitis deformans of unspecified lower leg	Arthritis Joint Lower Limb	Nociceptive Pain
M8887	Osteitis deformans of ankle and foot	Arthritis Joint Lower Limb	Nociceptive Pain
M88871	Osteitis deformans of right ankle and foot	Arthritis Joint Lower Limb	Nociceptive Pain
M88872	Osteitis deformans of left ankle and foot	Arthritis Joint Lower Limb	Nociceptive Pain
M88879	Osteitis deformans of unspecified ankle and foot	Arthritis Joint Lower Limb	Nociceptive Pain
M8888	Osteitis deformans of other bones	Arthritis Joint Other	Nociceptive Pain
M8889	Osteitis deformans of multiple sites	Arthritis Joint Other	Nociceptive Pain
M889	Osteitis deformans of unspecified bone	Arthritis Joint Other	Nociceptive Pain
M89	Other disorders of bone	Arthritis Joint Other	Nociceptive Pain
M890	Algoneurodystrophy	Arthritis Joint Other	Nociceptive Pain
M8900	Algoneurodystrophy, unspecified site	Arthritis Joint Other	Nociceptive Pain
M8901	Algoneurodystrophy, shoulder	Arthritis Joint Upper Limb	Nociceptive Pain
M89011	Algoneurodystrophy, right shoulder	Arthritis Joint Upper Limb	Nociceptive Pain
M89012	Algoneurodystrophy, left shoulder	Arthritis Joint Upper Limb	Nociceptive Pain
M89019	Algoneurodystrophy, unspecified shoulder	Arthritis Joint Upper Limb	Nociceptive Pain
M8902	Algoneurodystrophy, upper arm	Arthritis Joint Upper Limb	Nociceptive Pain
M89021	Algoneurodystrophy, right upper arm	Arthritis Joint Upper Limb	Nociceptive Pain
M89022	Algoneurodystrophy, left upper arm	Arthritis Joint Upper Limb	Nociceptive Pain
M89029	Algoneurodystrophy, unspecified upper arm	Arthritis Joint Upper Limb	Nociceptive Pain
M8903	Algoneurodystrophy, forearm	Arthritis Joint Upper Limb	Nociceptive Pain
M89031	Algoneurodystrophy, right forearm	Arthritis Joint Upper Limb	Nociceptive Pain
M89032	Algoneurodystrophy, left forearm	Arthritis Joint Upper Limb	Nociceptive Pain
M89039	Algoneurodystrophy, unspecified forearm	Arthritis Joint Upper Limb	Nociceptive Pain
M8904	Algoneurodystrophy, hand	Arthritis Joint Upper Limb	Nociceptive Pain
M89041	Algoneurodystrophy, right hand	Arthritis Joint Upper Limb	Nociceptive Pain
M89042	Algoneurodystrophy, left hand	Arthritis Joint Upper Limb	Nociceptive Pain
M89049	Algoneurodystrophy, unspecified hand	Arthritis Joint Upper Limb	Nociceptive Pain
M8905	Algoneurodystrophy, thigh	Arthritis Joint Lower Limb	Nociceptive Pain

© 2024 Peterson MD et al. *JAMA Neurology.*

M89051	Algoneurodystrophy, right thigh	Arthritis Joint Lower Limb	Nociceptive Pain
M89052	Algoneurodystrophy, left thigh	Arthritis Joint Lower Limb	Nociceptive Pain
M89059	Algoneurodystrophy, unspecified thigh	Arthritis Joint Lower Limb	Nociceptive Pain
M8906	Algoneurodystrophy, lower leg	Arthritis Joint Lower Limb	Nociceptive Pain
M89061	Algoneurodystrophy, right lower leg	Arthritis Joint Lower Limb	Nociceptive Pain
M89062	Algoneurodystrophy, left lower leg	Arthritis Joint Lower Limb	Nociceptive Pain
M89069	Algoneurodystrophy, unspecified lower leg	Arthritis Joint Lower Limb	Nociceptive Pain
M8907	Algoneurodystrophy, ankle and foot	Arthritis Joint Lower Limb	Nociceptive Pain
M89071	Algoneurodystrophy, right ankle and foot	Arthritis Joint Lower Limb	Nociceptive Pain
M89072	Algoneurodystrophy, left ankle and foot	Arthritis Joint Lower Limb	Nociceptive Pain
M89079	Algoneurodystrophy, unspecified ankle and foot	Arthritis Joint Lower Limb	Nociceptive Pain
M8908	Algoneurodystrophy, other site	Arthritis Joint Other	Nociceptive Pain
M8909	Algoneurodystrophy, multiple sites	Arthritis Joint Other	Nociceptive Pain
M891	Physeal arrest	Arthritis Joint Other	Nociceptive Pain
M8912	Physeal arrest, humerus	Arthritis Joint Upper Limb	Nociceptive Pain
M89121	Complete physeal arrest, right proximal humerus	Arthritis Joint Upper Limb	Nociceptive Pain
M89122	Complete physeal arrest, left proximal humerus	Arthritis Joint Upper Limb	Nociceptive Pain
M89123	Partial physeal arrest, right proximal humerus	Arthritis Joint Upper Limb	Nociceptive Pain
M89124	Partial physeal arrest, left proximal humerus	Arthritis Joint Upper Limb	Nociceptive Pain
M89125	Complete physeal arrest, right distal humerus	Arthritis Joint Upper Limb	Nociceptive Pain
M89126	Complete physeal arrest, left distal humerus	Arthritis Joint Upper Limb	Nociceptive Pain
M89127	Partial physeal arrest, right distal humerus	Arthritis Joint Upper Limb	Nociceptive Pain
M89128	Partial physeal arrest, left distal humerus	Arthritis Joint Upper Limb	Nociceptive Pain
M89129	Physeal arrest, humerus, unspecified	Arthritis Joint Upper Limb	Nociceptive Pain
M8913	Physeal arrest, forearm	Arthritis Joint Upper Limb	Nociceptive Pain
M89131	Complete physeal arrest, right distal radius	Arthritis Joint Upper Limb	Nociceptive Pain
M89132	Complete physeal arrest, left distal radius	Arthritis Joint Upper Limb	Nociceptive Pain
M89133	Partial physeal arrest, right distal radius	Arthritis Joint Upper Limb	Nociceptive Pain
M89134	Partial physeal arrest, left distal radius	Arthritis Joint Upper Limb	Nociceptive Pain
M89138	Other physeal arrest of forearm	Arthritis Joint Upper Limb	Nociceptive Pain
M89139	Physeal arrest, forearm, unspecified	Arthritis Joint Upper Limb	Nociceptive Pain
M8915	Physeal arrest, femur	Arthritis Joint Lower Limb	Nociceptive Pain
M89151	Complete physeal arrest, right proximal femur	Arthritis Joint Lower Limb	Nociceptive Pain
M89152	Complete physeal arrest, left proximal femur	Arthritis Joint Lower Limb	Nociceptive Pain
M89153	Partial physeal arrest, right proximal femur	Arthritis Joint Lower Limb	Nociceptive Pain
M89154	Partial physeal arrest, left proximal femur	Arthritis Joint Lower Limb	Nociceptive Pain
M89155	Complete physeal arrest, right distal femur	Arthritis Joint Lower Limb	Nociceptive Pain

© 2024 Peterson MD et al. *JAMA Neurology.*

M89156	Complete physeal arrest, left distal femur	Arthritis Joint Lower Limb	Nociceptive Pain
M89157	Partial physeal arrest, right distal femur	Arthritis Joint Lower Limb	Nociceptive Pain
M89158	Partial physeal arrest, left distal femur	Arthritis Joint Lower Limb	Nociceptive Pain
M89159	Physeal arrest, femur, unspecified	Arthritis Joint Lower Limb	Nociceptive Pain
M8916	Physeal arrest, lower leg	Arthritis Joint Lower Limb	Nociceptive Pain
M89160	Complete physeal arrest, right proximal tibia	Arthritis Joint Lower Limb	Nociceptive Pain
M89161	Complete physeal arrest, left proximal tibia	Arthritis Joint Lower Limb	Nociceptive Pain
M89162	Partial physeal arrest, right proximal tibia	Arthritis Joint Lower Limb	Nociceptive Pain
M89163	Partial physeal arrest, left proximal tibia	Arthritis Joint Lower Limb	Nociceptive Pain
M89164	Complete physeal arrest, right distal tibia	Arthritis Joint Lower Limb	Nociceptive Pain
M89165	Complete physeal arrest, left distal tibia	Arthritis Joint Lower Limb	Nociceptive Pain
M89166	Partial physeal arrest, right distal tibia	Arthritis Joint Lower Limb	Nociceptive Pain
M89167	Partial physeal arrest, left distal tibia	Arthritis Joint Lower Limb	Nociceptive Pain
M89168	Other physeal arrest of lower leg	Arthritis Joint Lower Limb	Nociceptive Pain
M89169	Physeal arrest, lower leg, unspecified	Arthritis Joint Lower Limb	Nociceptive Pain
M8918	Physeal arrest, other site	Arthritis Joint Other	Nociceptive Pain
M892	Other disorders of bone development and growth	Arthritis Joint Other	Nociceptive Pain
M8920	Other disorders of bone development and growth, unsp site	Arthritis Joint Other	Nociceptive Pain
M8921	Other disorders of bone development and growth, shoulder	Arthritis Joint Upper Limb	Nociceptive Pain
M89211	Oth disorders of bone development and growth, right shoulder	Arthritis Joint Upper Limb	Nociceptive Pain
M89212	Oth disorders of bone development and growth, left shoulder	Arthritis Joint Upper Limb	Nociceptive Pain
M89219	Oth disorders of bone development and growth, unsp shoulder	Arthritis Joint Upper Limb	Nociceptive Pain
M8922	Other disorders of bone development and growth, humerus	Arthritis Joint Upper Limb	Nociceptive Pain
M89221	Oth disorders of bone development and growth, right humerus	Arthritis Joint Upper Limb	Nociceptive Pain
M89222	Other disorders of bone development and growth, left humerus	Arthritis Joint Upper Limb	Nociceptive Pain
M89229	Other disorders of bone development and growth, unsp humerus	Arthritis Joint Upper Limb	Nociceptive Pain
M8923	Oth disorders of bone dev and growth, ulna and radius	Arthritis Joint Upper Limb	Nociceptive Pain
M89231	Other disorders of bone development and growth, right ulna	Arthritis Joint Upper Limb	Nociceptive Pain
M89232	Other disorders of bone development and growth, left ulna	Arthritis Joint Upper Limb	Nociceptive Pain
M89233	Other disorders of bone development and growth, right radius	Arthritis Joint Upper Limb	Nociceptive Pain
M89234	Other disorders of bone development and growth, left radius	Arthritis Joint Upper Limb	Nociceptive Pain
M89239	Oth disorders of bone dev and growth, unsp ulna and radius	Arthritis Joint Upper Limb	Nociceptive Pain
M8924	Other disorders of bone development and growth, hand	Arthritis Joint Upper Limb	Nociceptive Pain
M89241	Other disorders of bone development and growth, right hand	Arthritis Joint Upper Limb	Nociceptive Pain
M89242	Other disorders of bone development and growth, left hand	Arthritis Joint Upper Limb	Nociceptive Pain
M89249	Other disorders of bone development and growth, unsp hand	Arthritis Joint Upper Limb	Nociceptive Pain

© 2024 Peterson MD et al. *JAMA Neurology.*

M8925	Other disorders of bone development and growth, femur	Arthritis Joint Lower Limb	Nociceptive Pain
M89251	Other disorders of bone development and growth, right femur	Arthritis Joint Lower Limb	Nociceptive Pain
M89252	Other disorders of bone development and growth, left femur	Arthritis Joint Lower Limb	Nociceptive Pain
M89259	Other disorders of bone development and growth, unsp femur	Arthritis Joint Lower Limb	Nociceptive Pain
M8926	Oth disorders of bone dev and growth, tibia and fibula	Arthritis Joint Lower Limb	Nociceptive Pain
M89261	Other disorders of bone development and growth, right tibia	Arthritis Joint Lower Limb	Nociceptive Pain
M89262	Other disorders of bone development and growth, left tibia	Arthritis Joint Lower Limb	Nociceptive Pain
M89263	Other disorders of bone development and growth, right fibula	Arthritis Joint Lower Limb	Nociceptive Pain
M89264	Other disorders of bone development and growth, left fibula	Arthritis Joint Lower Limb	Nociceptive Pain
M89269	Oth disorders of bone development and growth, unsp lower leg	Arthritis Joint Lower Limb	Nociceptive Pain
M8927	Oth disorders of bone development and growth, ankle and foot	Arthritis Joint Lower Limb	Nociceptive Pain
M89271	Oth disorders of bone development and growth, right ank/ft	Arthritis Joint Lower Limb	Nociceptive Pain
M89272	Oth disorders of bone development and growth, left ank/ft	Arthritis Joint Lower Limb	Nociceptive Pain
M89279	Oth disorders of bone development and growth, unsp ank/ft	Arthritis Joint Lower Limb	Nociceptive Pain
M8928	Other disorders of bone development and growth, other site	Arthritis Joint Other	Nociceptive Pain
M8929	Oth disorders of bone development and growth, multiple sites	Arthritis Joint Other	Nociceptive Pain
M893	Hypertrophy of bone	Arthritis Joint Other	Nociceptive Pain
M8930	Hypertrophy of bone, unspecified site	Arthritis Joint Other	Nociceptive Pain
M8931	Hypertrophy of bone, shoulder	Arthritis Joint Upper Limb	Nociceptive Pain
M89311	Hypertrophy of bone, right shoulder	Arthritis Joint Upper Limb	Nociceptive Pain
M89312	Hypertrophy of bone, left shoulder	Arthritis Joint Upper Limb	Nociceptive Pain
M89319	Hypertrophy of bone, unspecified shoulder	Arthritis Joint Upper Limb	Nociceptive Pain
M8932	Hypertrophy of bone, humerus	Arthritis Joint Upper Limb	Nociceptive Pain
M89321	Hypertrophy of bone, right humerus	Arthritis Joint Upper Limb	Nociceptive Pain
M89322	Hypertrophy of bone, left humerus	Arthritis Joint Upper Limb	Nociceptive Pain
M89329	Hypertrophy of bone, unspecified humerus	Arthritis Joint Upper Limb	Nociceptive Pain
M8933	Hypertrophy of bone, ulna and radius	Arthritis Joint Upper Limb	Nociceptive Pain
M89331	Hypertrophy of bone, right ulna	Arthritis Joint Upper Limb	Nociceptive Pain
M89332	Hypertrophy of bone, left ulna	Arthritis Joint Upper Limb	Nociceptive Pain
M89333	Hypertrophy of bone, right radius	Arthritis Joint Upper Limb	Nociceptive Pain
M89334	Hypertrophy of bone, left radius	Arthritis Joint Upper Limb	Nociceptive Pain
M89339	Hypertrophy of bone, unspecified ulna and radius	Arthritis Joint Upper Limb	Nociceptive Pain
M8934	Hypertrophy of bone, hand	Arthritis Joint Upper Limb	Nociceptive Pain
M89341	Hypertrophy of bone, right hand	Arthritis Joint Upper Limb	Nociceptive Pain
M89342	Hypertrophy of bone, left hand	Arthritis Joint Upper Limb	Nociceptive Pain
M89349	Hypertrophy of bone, unspecified hand	Arthritis Joint Upper Limb	Nociceptive Pain

© 2024 Peterson MD et al. *JAMA Neurology.*

M8935	Hypertrophy of bone, femur	Arthritis Joint Lower Limb	Nociceptive Pain
M89351	Hypertrophy of bone, right femur	Arthritis Joint Lower Limb	Nociceptive Pain
M89352	Hypertrophy of bone, left femur	Arthritis Joint Lower Limb	Nociceptive Pain
M89359	Hypertrophy of bone, unspecified femur	Arthritis Joint Lower Limb	Nociceptive Pain
M8936	Hypertrophy of bone, tibia and fibula	Arthritis Joint Lower Limb	Nociceptive Pain
M89361	Hypertrophy of bone, right tibia	Arthritis Joint Lower Limb	Nociceptive Pain
M89362	Hypertrophy of bone, left tibia	Arthritis Joint Lower Limb	Nociceptive Pain
M89363	Hypertrophy of bone, right fibula	Arthritis Joint Lower Limb	Nociceptive Pain
M89364	Hypertrophy of bone, left fibula	Arthritis Joint Lower Limb	Nociceptive Pain
M89369	Hypertrophy of bone, unspecified tibia and fibula	Arthritis Joint Lower Limb	Nociceptive Pain
M8937	Hypertrophy of bone, ankle and foot	Arthritis Joint Lower Limb	Nociceptive Pain
M89371	Hypertrophy of bone, right ankle and foot	Arthritis Joint Lower Limb	Nociceptive Pain
M89372	Hypertrophy of bone, left ankle and foot	Arthritis Joint Lower Limb	Nociceptive Pain
M89379	Hypertrophy of bone, unspecified ankle and foot	Arthritis Joint Lower Limb	Nociceptive Pain
M8938	Hypertrophy of bone, other site	Arthritis Joint Other	Nociceptive Pain
M8939	Hypertrophy of bone, multiple sites	Arthritis Joint Other	Nociceptive Pain
M894	Other hypertrophic osteoarthropathy	Arthritis Joint Other	Nociceptive Pain
M8940	Other hypertrophic osteoarthropathy, unspecified site	Arthritis Joint Other	Nociceptive Pain
M8941	Other hypertrophic osteoarthropathy, shoulder	Arthritis Joint Upper Limb	Nociceptive Pain
M89411	Other hypertrophic osteoarthropathy, right shoulder	Arthritis Joint Upper Limb	Nociceptive Pain
M89412	Other hypertrophic osteoarthropathy, left shoulder	Arthritis Joint Upper Limb	Nociceptive Pain
M89419	Other hypertrophic osteoarthropathy, unspecified shoulder	Arthritis Joint Upper Limb	Nociceptive Pain
M8942	Other hypertrophic osteoarthropathy, upper arm	Arthritis Joint Upper Limb	Nociceptive Pain
M89421	Other hypertrophic osteoarthropathy, right upper arm	Arthritis Joint Upper Limb	Nociceptive Pain
M89422	Other hypertrophic osteoarthropathy, left upper arm	Arthritis Joint Upper Limb	Nociceptive Pain
M89429	Other hypertrophic osteoarthropathy, unspecified upper arm	Arthritis Joint Upper Limb	Nociceptive Pain
M8943	Other hypertrophic osteoarthropathy, forearm	Arthritis Joint Upper Limb	Nociceptive Pain
M89431	Other hypertrophic osteoarthropathy, right forearm	Arthritis Joint Upper Limb	Nociceptive Pain
M89432	Other hypertrophic osteoarthropathy, left forearm	Arthritis Joint Upper Limb	Nociceptive Pain
M89439	Other hypertrophic osteoarthropathy, unspecified forearm	Arthritis Joint Upper Limb	Nociceptive Pain
M8944	Other hypertrophic osteoarthropathy, hand	Arthritis Joint Upper Limb	Nociceptive Pain
M89441	Other hypertrophic osteoarthropathy, right hand	Arthritis Joint Upper Limb	Nociceptive Pain
M89442	Other hypertrophic osteoarthropathy, left hand	Arthritis Joint Upper Limb	Nociceptive Pain
M89449	Other hypertrophic osteoarthropathy, unspecified hand	Arthritis Joint Upper Limb	Nociceptive Pain
M8945	Other hypertrophic osteoarthropathy, thigh	Arthritis Joint Lower Limb	Nociceptive Pain
M89451	Other hypertrophic osteoarthropathy, right thigh	Arthritis Joint Lower Limb	Nociceptive Pain
M89452	Other hypertrophic osteoarthropathy, left thigh	Arthritis Joint Lower Limb	Nociceptive Pain

© 2024 Peterson MD et al. *JAMA Neurology.*

M89459	Other hypertrophic osteoarthropathy, unspecified thigh	Arthritis Joint Lower Limb	Nociceptive Pain
M8946	Other hypertrophic osteoarthropathy, lower leg	Arthritis Joint Lower Limb	Nociceptive Pain
M89461	Other hypertrophic osteoarthropathy, right lower leg	Arthritis Joint Lower Limb	Nociceptive Pain
M89462	Other hypertrophic osteoarthropathy, left lower leg	Arthritis Joint Lower Limb	Nociceptive Pain
M89469	Other hypertrophic osteoarthropathy, unspecified lower leg	Arthritis Joint Lower Limb	Nociceptive Pain
M8947	Other hypertrophic osteoarthropathy, ankle and foot	Arthritis Joint Lower Limb	Nociceptive Pain
M89471	Other hypertrophic osteoarthropathy, right ankle and foot	Arthritis Joint Lower Limb	Nociceptive Pain
M89472	Other hypertrophic osteoarthropathy, left ankle and foot	Arthritis Joint Lower Limb	Nociceptive Pain
M89479	Other hypertrophic osteoarthropathy, unsp ankle and foot	Arthritis Joint Lower Limb	Nociceptive Pain
M8948	Other hypertrophic osteoarthropathy, other site	Arthritis Joint Other	Nociceptive Pain
M8949	Other hypertrophic osteoarthropathy, multiple sites	Arthritis Joint Other	Nociceptive Pain
M895	Osteolysis	Arthritis Joint Other	Nociceptive Pain
M8950	Osteolysis, unspecified site	Arthritis Joint Other	Nociceptive Pain
M8951	Osteolysis, shoulder	Arthritis Joint Upper Limb	Nociceptive Pain
M89511	Osteolysis, right shoulder	Arthritis Joint Upper Limb	Nociceptive Pain
M89512	Osteolysis, left shoulder	Arthritis Joint Upper Limb	Nociceptive Pain
M89519	Osteolysis, unspecified shoulder	Arthritis Joint Upper Limb	Nociceptive Pain
M8952	Osteolysis, upper arm	Arthritis Joint Upper Limb	Nociceptive Pain
M89521	Osteolysis, right upper arm	Arthritis Joint Upper Limb	Nociceptive Pain
M89522	Osteolysis, left upper arm	Arthritis Joint Upper Limb	Nociceptive Pain
M89529	Osteolysis, unspecified upper arm	Arthritis Joint Upper Limb	Nociceptive Pain
M8953	Osteolysis, forearm	Arthritis Joint Upper Limb	Nociceptive Pain
M89531	Osteolysis, right forearm	Arthritis Joint Upper Limb	Nociceptive Pain
M89532	Osteolysis, left forearm	Arthritis Joint Upper Limb	Nociceptive Pain
M89539	Osteolysis, unspecified forearm	Arthritis Joint Upper Limb	Nociceptive Pain
M8954	Osteolysis, hand	Arthritis Joint Upper Limb	Nociceptive Pain
M89541	Osteolysis, right hand	Arthritis Joint Upper Limb	Nociceptive Pain
M89542	Osteolysis, left hand	Arthritis Joint Upper Limb	Nociceptive Pain
M89549	Osteolysis, unspecified hand	Arthritis Joint Upper Limb	Nociceptive Pain
M8955	Osteolysis, thigh	Arthritis Joint Lower Limb	Nociceptive Pain
M89551	Osteolysis, right thigh	Arthritis Joint Lower Limb	Nociceptive Pain
M89552	Osteolysis, left thigh	Arthritis Joint Lower Limb	Nociceptive Pain
M89559	Osteolysis, unspecified thigh	Arthritis Joint Lower Limb	Nociceptive Pain
M8956	Osteolysis, lower leg	Arthritis Joint Lower Limb	Nociceptive Pain
M89561	Osteolysis, right lower leg	Arthritis Joint Lower Limb	Nociceptive Pain
M89562	Osteolysis, left lower leg	Arthritis Joint Lower Limb	Nociceptive Pain
M89569	Osteolysis, unspecified lower leg	Arthritis Joint Lower Limb	Nociceptive Pain

© 2024 Peterson MD et al. *JAMA Neurology.*

M8957	Osteolysis, ankle and foot	Arthritis Joint Lower Limb	Nociceptive Pain
M89571	Osteolysis, right ankle and foot	Arthritis Joint Lower Limb	Nociceptive Pain
M89572	Osteolysis, left ankle and foot	Arthritis Joint Lower Limb	Nociceptive Pain
M89579	Osteolysis, unspecified ankle and foot	Arthritis Joint Lower Limb	Nociceptive Pain
M8958	Osteolysis, other site	Arthritis Joint Other	Nociceptive Pain
M8959	Osteolysis, multiple sites	Arthritis Joint Other	Nociceptive Pain
M896	Osteopathy after poliomyelitis	Arthritis Joint Other	Nociceptive Pain
M8960	Osteopathy after poliomyelitis, unspecified site	Arthritis Joint Other	Nociceptive Pain
M8961	Osteopathy after poliomyelitis, shoulder	Arthritis Joint Upper Limb	Nociceptive Pain
M89611	Osteopathy after poliomyelitis, right shoulder	Arthritis Joint Upper Limb	Nociceptive Pain
M89612	Osteopathy after poliomyelitis, left shoulder	Arthritis Joint Upper Limb	Nociceptive Pain
M89619	Osteopathy after poliomyelitis, unspecified shoulder	Arthritis Joint Upper Limb	Nociceptive Pain
M8962	Osteopathy after poliomyelitis, upper arm	Arthritis Joint Upper Limb	Nociceptive Pain
M89621	Osteopathy after poliomyelitis, right upper arm	Arthritis Joint Upper Limb	Nociceptive Pain
M89622	Osteopathy after poliomyelitis, left upper arm	Arthritis Joint Upper Limb	Nociceptive Pain
M89629	Osteopathy after poliomyelitis, unspecified upper arm	Arthritis Joint Upper Limb	Nociceptive Pain
M8963	Osteopathy after poliomyelitis, forearm	Arthritis Joint Upper Limb	Nociceptive Pain
M89631	Osteopathy after poliomyelitis, right forearm	Arthritis Joint Upper Limb	Nociceptive Pain
M89632	Osteopathy after poliomyelitis, left forearm	Arthritis Joint Upper Limb	Nociceptive Pain
M89639	Osteopathy after poliomyelitis, unspecified forearm	Arthritis Joint Upper Limb	Nociceptive Pain
M8964	Osteopathy after poliomyelitis, hand	Arthritis Joint Upper Limb	Nociceptive Pain
M89641	Osteopathy after poliomyelitis, right hand	Arthritis Joint Upper Limb	Nociceptive Pain
M89642	Osteopathy after poliomyelitis, left hand	Arthritis Joint Upper Limb	Nociceptive Pain
M89649	Osteopathy after poliomyelitis, unspecified hand	Arthritis Joint Upper Limb	Nociceptive Pain
M8965	Osteopathy after poliomyelitis, thigh	Arthritis Joint Lower Limb	Nociceptive Pain
M89651	Osteopathy after poliomyelitis, right thigh	Arthritis Joint Lower Limb	Nociceptive Pain
M89652	Osteopathy after poliomyelitis, left thigh	Arthritis Joint Lower Limb	Nociceptive Pain
M89659	Osteopathy after poliomyelitis, unspecified thigh	Arthritis Joint Lower Limb	Nociceptive Pain
M8966	Osteopathy after poliomyelitis, lower leg	Arthritis Joint Lower Limb	Nociceptive Pain
M89661	Osteopathy after poliomyelitis, right lower leg	Arthritis Joint Lower Limb	Nociceptive Pain
M89662	Osteopathy after poliomyelitis, left lower leg	Arthritis Joint Lower Limb	Nociceptive Pain
M89669	Osteopathy after poliomyelitis, unspecified lower leg	Arthritis Joint Lower Limb	Nociceptive Pain
M8967	Osteopathy after poliomyelitis, ankle and foot	Arthritis Joint Lower Limb	Nociceptive Pain
M89671	Osteopathy after poliomyelitis, right ankle and foot	Arthritis Joint Lower Limb	Nociceptive Pain
M89672	Osteopathy after poliomyelitis, left ankle and foot	Arthritis Joint Lower Limb	Nociceptive Pain
M89679	Osteopathy after poliomyelitis, unspecified ankle and foot	Arthritis Joint Lower Limb	Nociceptive Pain
M8968	Osteopathy after poliomyelitis, other site	Arthritis Joint Other	Nociceptive Pain

© 2024 Peterson MD et al. *JAMA Neurology.*

M8969	Osteopathy after poliomyelitis, multiple sites	Arthritis Joint Other	Nociceptive Pain
M897	Major osseous defect	Arthritis Joint Other	Nociceptive Pain
M8970	Major osseous defect, unspecified site	Arthritis Joint Other	Nociceptive Pain
M8971	Major osseous defect, shoulder region	Arthritis Joint Upper Limb	Nociceptive Pain
M89711	Major osseous defect, right shoulder region	Arthritis Joint Upper Limb	Nociceptive Pain
M89712	Major osseous defect, left shoulder region	Arthritis Joint Upper Limb	Nociceptive Pain
M89719	Major osseous defect, unspecified shoulder region	Arthritis Joint Upper Limb	Nociceptive Pain
M8972	Major osseous defect, humerus	Arthritis Joint Upper Limb	Nociceptive Pain
M89721	Major osseous defect, right humerus	Arthritis Joint Upper Limb	Nociceptive Pain
M89722	Major osseous defect, left humerus	Arthritis Joint Upper Limb	Nociceptive Pain
M89729	Major osseous defect, unspecified humerus	Arthritis Joint Upper Limb	Nociceptive Pain
M8973	Major osseous defect, forearm	Arthritis Joint Upper Limb	Nociceptive Pain
M89731	Major osseous defect, right forearm	Arthritis Joint Upper Limb	Nociceptive Pain
M89732	Major osseous defect, left forearm	Arthritis Joint Upper Limb	Nociceptive Pain
M89739	Major osseous defect, unspecified forearm	Arthritis Joint Upper Limb	Nociceptive Pain
M8974	Major osseous defect, hand	Arthritis Joint Upper Limb	Nociceptive Pain
M89741	Major osseous defect, right hand	Arthritis Joint Upper Limb	Nociceptive Pain
M89742	Major osseous defect, left hand	Arthritis Joint Upper Limb	Nociceptive Pain
M89749	Major osseous defect, unspecified hand	Arthritis Joint Upper Limb	Nociceptive Pain
M8975	Major osseous defect, pelvic region and thigh	Arthritis Joint Lower Limb	Nociceptive Pain
M89751	Major osseous defect, right pelvic region and thigh	Arthritis Joint Lower Limb	Nociceptive Pain
M89752	Major osseous defect, left pelvic region and thigh	Arthritis Joint Lower Limb	Nociceptive Pain
M89759	Major osseous defect, unspecified pelvic region and thigh	Arthritis Joint Lower Limb	Nociceptive Pain
M8976	Major osseous defect, lower leg	Arthritis Joint Lower Limb	Nociceptive Pain
M89761	Major osseous defect, right lower leg	Arthritis Joint Lower Limb	Nociceptive Pain
M89762	Major osseous defect, left lower leg	Arthritis Joint Lower Limb	Nociceptive Pain
M89769	Major osseous defect, unspecified lower leg	Arthritis Joint Lower Limb	Nociceptive Pain
M8977	Major osseous defect, ankle and foot	Arthritis Joint Lower Limb	Nociceptive Pain
M89771	Major osseous defect, right ankle and foot	Arthritis Joint Lower Limb	Nociceptive Pain
M89772	Major osseous defect, left ankle and foot	Arthritis Joint Lower Limb	Nociceptive Pain
M89779	Major osseous defect, unspecified ankle and foot	Arthritis Joint Lower Limb	Nociceptive Pain
M8978	Major osseous defect, other site	Arthritis Joint Other	Nociceptive Pain
M8979	Major osseous defect, multiple sites	Arthritis Joint Other	Nociceptive Pain
M898	Other specified disorders of bone	Arthritis Joint Other	Nociceptive Pain
M898X	Other specified disorders of bone	Arthritis Joint Other	Nociceptive Pain
M898X0	Other specified disorders of bone, multiple sites	Arthritis Joint Other	Nociceptive Pain
M898X1	Other specified disorders of bone, shoulder	Arthritis Joint Upper Limb	Nociceptive Pain

© 2024 Peterson MD et al. *JAMA Neurology.*

M898X2	Other specified disorders of bone, upper arm	Arthritis Joint Upper Limb	Nociceptive Pain
M898X3	Other specified disorders of bone, forearm	Arthritis Joint Upper Limb	Nociceptive Pain
M898X4	Other specified disorders of bone, hand	Arthritis Joint Upper Limb	Nociceptive Pain
M898X5	Other specified disorders of bone, thigh	Arthritis Joint Lower Limb	Nociceptive Pain
M898X6	Other specified disorders of bone, lower leg	Arthritis Joint Lower Limb	Nociceptive Pain
M898X7	Other specified disorders of bone, ankle and foot	Arthritis Joint Lower Limb	Nociceptive Pain
M898X8	Other specified disorders of bone, other site	Arthritis Joint Other	Nociceptive Pain
M898X9	Other specified disorders of bone, unspecified site	Arthritis Joint Other	Nociceptive Pain
M899	Disorder of bone, unspecified	Arthritis Joint Other	Nociceptive Pain
M90	Osteopathies in diseases classified elsewhere	Arthritis Joint Other	Nociceptive Pain
M905	Osteonecrosis in diseases classified elsewhere	Arthritis Joint Other	Nociceptive Pain
M9050	Osteonecrosis in diseases classified elsewhere, unsp site	Arthritis Joint Other	Nociceptive Pain
M9051	Osteonecrosis in diseases classified elsewhere, shoulder	Arthritis Joint Upper Limb	Nociceptive Pain
M90511	Osteonecrosis in diseases classd elswhr, right shoulder	Arthritis Joint Upper Limb	Nociceptive Pain
M90512	Osteonecrosis in diseases classd elswhr, left shoulder	Arthritis Joint Upper Limb	Nociceptive Pain
M90519	Osteonecrosis in diseases classd elswhr, unsp shoulder	Arthritis Joint Upper Limb	Nociceptive Pain
M9052	Osteonecrosis in diseases classified elsewhere, upper arm	Arthritis Joint Upper Limb	Nociceptive Pain
M90521	Osteonecrosis in diseases classd elswhr, right upper arm	Arthritis Joint Upper Limb	Nociceptive Pain
M90522	Osteonecrosis in diseases classd elswhr, left upper arm	Arthritis Joint Upper Limb	Nociceptive Pain
M90529	Osteonecrosis in diseases classd elswhr, unsp upper arm	Arthritis Joint Upper Limb	Nociceptive Pain
M9053	Osteonecrosis in diseases classified elsewhere, forearm	Arthritis Joint Upper Limb	Nociceptive Pain
M90531	Osteonecrosis in diseases classd elswhr, right forearm	Arthritis Joint Upper Limb	Nociceptive Pain
M90532	Osteonecrosis in diseases classified elsewhere, left forearm	Arthritis Joint Upper Limb	Nociceptive Pain
M90539	Osteonecrosis in diseases classified elsewhere, unsp forearm	Arthritis Joint Upper Limb	Nociceptive Pain
M9054	Osteonecrosis in diseases classified elsewhere, hand	Arthritis Joint Upper Limb	Nociceptive Pain
M90541	Osteonecrosis in diseases classified elsewhere, right hand	Arthritis Joint Upper Limb	Nociceptive Pain
M90542	Osteonecrosis in diseases classified elsewhere, left hand	Arthritis Joint Upper Limb	Nociceptive Pain
M90549	Osteonecrosis in diseases classified elsewhere, unsp hand	Arthritis Joint Upper Limb	Nociceptive Pain
M9055	Osteonecrosis in diseases classified elsewhere, thigh	Arthritis Joint Lower Limb	Nociceptive Pain
M90551	Osteonecrosis in diseases classified elsewhere, right thigh	Arthritis Joint Lower Limb	Nociceptive Pain
M90552	Osteonecrosis in diseases classified elsewhere, left thigh	Arthritis Joint Lower Limb	Nociceptive Pain
M90559	Osteonecrosis in diseases classified elsewhere, unsp thigh	Arthritis Joint Lower Limb	Nociceptive Pain
M9056	Osteonecrosis in diseases classified elsewhere, lower leg	Arthritis Joint Lower Limb	Nociceptive Pain
M90561	Osteonecrosis in diseases classd elswhr, right lower leg	Arthritis Joint Lower Limb	Nociceptive Pain
M90562	Osteonecrosis in diseases classd elswhr, left lower leg	Arthritis Joint Lower Limb	Nociceptive Pain
M90569	Osteonecrosis in diseases classd elswhr, unsp lower leg	Arthritis Joint Lower Limb	Nociceptive Pain
M9057	Osteonecrosis in diseases classd elswhr, ankle and foot	Arthritis Joint Lower Limb	Nociceptive Pain

© 2024 Peterson MD et al. *JAMA Neurology.*

M90571	Osteonecrosis in diseases classd elswhr, right ank/ft	Arthritis Joint Lower Limb	Nociceptive Pain
M90572	Osteonecrosis in diseases classd elswhr, left ankle and foot	Arthritis Joint Lower Limb	Nociceptive Pain
M90579	Osteonecrosis in diseases classd elswhr, unsp ankle and foot	Arthritis Joint Lower Limb	Nociceptive Pain
M9058	Osteonecrosis in diseases classified elsewhere, other site	Arthritis Joint Other	Nociceptive Pain
M9059	Osteonecrosis in diseases classd elswhr, multiple sites	Arthritis Joint Other	Nociceptive Pain
M906	Osteitis deformans in neoplastic diseases	Arthritis Joint Other	Nociceptive Pain
M9060	Osteitis deformans in neoplastic diseases, unspecified site	Arthritis Joint Other	Nociceptive Pain
M9061	Osteitis deformans in neoplastic diseases, shoulder	Arthritis Joint Upper Limb	Nociceptive Pain
M90611	Osteitis deformans in neoplastic diseases, right shoulder	Arthritis Joint Upper Limb	Nociceptive Pain
M90612	Osteitis deformans in neoplastic diseases, left shoulder	Arthritis Joint Upper Limb	Nociceptive Pain
M90619	Osteitis deformans in neoplastic diseases, unsp shoulder	Arthritis Joint Upper Limb	Nociceptive Pain
M9062	Osteitis deformans in neoplastic diseases, upper arm	Arthritis Joint Upper Limb	Nociceptive Pain
M90621	Osteitis deformans in neoplastic diseases, right upper arm	Arthritis Joint Upper Limb	Nociceptive Pain
M90622	Osteitis deformans in neoplastic diseases, left upper arm	Arthritis Joint Upper Limb	Nociceptive Pain
M90629	Osteitis deformans in neoplastic diseases, unsp upper arm	Arthritis Joint Upper Limb	Nociceptive Pain
M9063	Osteitis deformans in neoplastic diseases, forearm	Arthritis Joint Upper Limb	Nociceptive Pain
M90631	Osteitis deformans in neoplastic diseases, right forearm	Arthritis Joint Upper Limb	Nociceptive Pain
M90632	Osteitis deformans in neoplastic diseases, left forearm	Arthritis Joint Upper Limb	Nociceptive Pain
M90639	Osteitis deformans in neoplastic diseases, unsp forearm	Arthritis Joint Upper Limb	Nociceptive Pain
M9064	Osteitis deformans in neoplastic diseases, hand	Arthritis Joint Upper Limb	Nociceptive Pain
M90641	Osteitis deformans in neoplastic diseases, right hand	Arthritis Joint Upper Limb	Nociceptive Pain
M90642	Osteitis deformans in neoplastic diseases, left hand	Arthritis Joint Upper Limb	Nociceptive Pain
M90649	Osteitis deformans in neoplastic diseases, unspecified hand	Arthritis Joint Upper Limb	Nociceptive Pain
M9065	Osteitis deformans in neoplastic diseases, thigh	Arthritis Joint Lower Limb	Nociceptive Pain
M90651	Osteitis deformans in neoplastic diseases, right thigh	Arthritis Joint Lower Limb	Nociceptive Pain
M90652	Osteitis deformans in neoplastic diseases, left thigh	Arthritis Joint Lower Limb	Nociceptive Pain
M90659	Osteitis deformans in neoplastic diseases, unspecified thigh	Arthritis Joint Lower Limb	Nociceptive Pain
M9066	Osteitis deformans in neoplastic diseases, lower leg	Arthritis Joint Lower Limb	Nociceptive Pain
M90661	Osteitis deformans in neoplastic diseases, right lower leg	Arthritis Joint Lower Limb	Nociceptive Pain
M90662	Osteitis deformans in neoplastic diseases, left lower leg	Arthritis Joint Lower Limb	Nociceptive Pain
M90669	Osteitis deformans in neoplastic diseases, unsp lower leg	Arthritis Joint Lower Limb	Nociceptive Pain
M9067	Osteitis deformans in neoplastic diseases, ankle and foot	Arthritis Joint Lower Limb	Nociceptive Pain
M90671	Osteitis deformans in neoplastic diseases, right ank/ft	Arthritis Joint Lower Limb	Nociceptive Pain
M90672	Osteitis deformans in neoplastic diseases, left ank/ft	Arthritis Joint Lower Limb	Nociceptive Pain
M90679	Osteitis deformans in neoplastic diseases, unsp ank/ft	Arthritis Joint Lower Limb	Nociceptive Pain
M9068	Osteitis deformans in neoplastic diseases, other site	Arthritis Joint Other	Nociceptive Pain
M9069	Osteitis deformans in neoplastic diseases, multiple sites	Arthritis Joint Other	Nociceptive Pain

© 2024 Peterson MD et al. *JAMA Neurology.*

M908	Osteopathy in diseases classified elsewhere	Arthritis Joint Other	Nociceptive Pain
M9080	Osteopathy in diseases classified elsewhere, unsp site	Arthritis Joint Other	Nociceptive Pain
M9081	Osteopathy in diseases classified elsewhere, shoulder	Arthritis Joint Upper Limb	Nociceptive Pain
M90811	Osteopathy in diseases classified elsewhere, right shoulder	Arthritis Joint Upper Limb	Nociceptive Pain
M90812	Osteopathy in diseases classified elsewhere, left shoulder	Arthritis Joint Upper Limb	Nociceptive Pain
M90819	Osteopathy in diseases classified elsewhere, unsp shoulder	Arthritis Joint Upper Limb	Nociceptive Pain
M9082	Osteopathy in diseases classified elsewhere, upper arm	Arthritis Joint Upper Limb	Nociceptive Pain
M90821	Osteopathy in diseases classified elsewhere, right upper arm	Arthritis Joint Upper Limb	Nociceptive Pain
M90822	Osteopathy in diseases classified elsewhere, left upper arm	Arthritis Joint Upper Limb	Nociceptive Pain
M90829	Osteopathy in diseases classified elsewhere, unsp upper arm	Arthritis Joint Upper Limb	Nociceptive Pain
M9083	Osteopathy in diseases classified elsewhere, forearm	Arthritis Joint Upper Limb	Nociceptive Pain
M90831	Osteopathy in diseases classified elsewhere, right forearm	Arthritis Joint Upper Limb	Nociceptive Pain
M90832	Osteopathy in diseases classified elsewhere, left forearm	Arthritis Joint Upper Limb	Nociceptive Pain
M90839	Osteopathy in diseases classified elsewhere, unsp forearm	Arthritis Joint Upper Limb	Nociceptive Pain
M9084	Osteopathy in diseases classified elsewhere, hand	Arthritis Joint Upper Limb	Nociceptive Pain
M90841	Osteopathy in diseases classified elsewhere, right hand	Arthritis Joint Upper Limb	Nociceptive Pain
M90842	Osteopathy in diseases classified elsewhere, left hand	Arthritis Joint Upper Limb	Nociceptive Pain
M90849	Osteopathy in diseases classified elsewhere, unsp hand	Arthritis Joint Upper Limb	Nociceptive Pain
M9085	Osteopathy in diseases classified elsewhere, thigh	Arthritis Joint Lower Limb	Nociceptive Pain
M90851	Osteopathy in diseases classified elsewhere, right thigh	Arthritis Joint Lower Limb	Nociceptive Pain
M90852	Osteopathy in diseases classified elsewhere, left thigh	Arthritis Joint Lower Limb	Nociceptive Pain
M90859	Osteopathy in diseases classified elsewhere, unsp thigh	Arthritis Joint Lower Limb	Nociceptive Pain
M9086	Osteopathy in diseases classified elsewhere, lower leg	Arthritis Joint Lower Limb	Nociceptive Pain
M90861	Osteopathy in diseases classified elsewhere, right lower leg	Arthritis Joint Lower Limb	Nociceptive Pain
M90862	Osteopathy in diseases classified elsewhere, left lower leg	Arthritis Joint Lower Limb	Nociceptive Pain
M90869	Osteopathy in diseases classified elsewhere, unsp lower leg	Arthritis Joint Lower Limb	Nociceptive Pain
M9087	Osteopathy in diseases classified elsewhere, ankle and foot	Arthritis Joint Lower Limb	Nociceptive Pain
M90871	Osteopathy in diseases classd elswhr, right ankle and foot	Arthritis Joint Lower Limb	Nociceptive Pain
M90872	Osteopathy in diseases classd elswhr, left ankle and foot	Arthritis Joint Lower Limb	Nociceptive Pain
M90879	Osteopathy in diseases classd elswhr, unsp ankle and foot	Arthritis Joint Lower Limb	Nociceptive Pain
M9088	Osteopathy in diseases classified elsewhere, other site	Arthritis Joint Other	Nociceptive Pain
M9089	Osteopathy in diseases classified elsewhere, multiple sites	Arthritis Joint Other	Nociceptive Pain
M91	Juvenile osteochondrosis of hip and pelvis	Arthritis Joint Spine and Hips	Nociceptive Pain
M910	Juvenile osteochondrosis of pelvis	Arthritis Joint Spine and Hips	Nociceptive Pain
M911	Juvenile osteochondrosis of head of femur	Arthritis Joint Lower Limb	Nociceptive Pain
M9110	Juvenile osteochondrosis of head of femur, unspecified leg	Arthritis Joint Lower Limb	Nociceptive Pain
M9111	Juvenile osteochondrosis of head of femur, right leg	Arthritis Joint Lower Limb	Nociceptive Pain

© 2024 Peterson MD et al. *JAMA Neurology.*

M9112	Juvenile osteochondrosis of head of femur, left leg	Arthritis Joint Lower Limb	Nociceptive Pain
M912	Coxa plana	Arthritis Joint Spine and Hips	Nociceptive Pain
M9120	Coxa plana, unspecified hip	Arthritis Joint Spine and Hips	Nociceptive Pain
M9121	Coxa plana, right hip	Arthritis Joint Spine and Hips	Nociceptive Pain
M9122	Coxa plana, left hip	Arthritis Joint Spine and Hips	Nociceptive Pain
M913	Pseudocoxalgia	Arthritis Joint Spine and Hips	Nociceptive Pain
M9130	Pseudocoxalgia, unspecified hip	Arthritis Joint Spine and Hips	Nociceptive Pain
M9131	Pseudocoxalgia, right hip	Arthritis Joint Spine and Hips	Nociceptive Pain
M9132	Pseudocoxalgia, left hip	Arthritis Joint Spine and Hips	Nociceptive Pain
M914	Coxa magna	Arthritis Joint Spine and Hips	Nociceptive Pain
M9140	Coxa magna, unspecified hip	Arthritis Joint Spine and Hips	Nociceptive Pain
M9141	Coxa magna, right hip	Arthritis Joint Spine and Hips	Nociceptive Pain
M9142	Coxa magna, left hip	Arthritis Joint Spine and Hips	Nociceptive Pain
M918	Other juvenile osteochondrosis of hip and pelvis	Arthritis Joint Spine and Hips	Nociceptive Pain
M9180	Other juvenile osteochondrosis of hip and pelvis, unsp leg	Arthritis Joint Spine and Hips	Nociceptive Pain
M9181	Other juvenile osteochondrosis of hip and pelvis, right leg	Arthritis Joint Spine and Hips	Nociceptive Pain
M9182	Other juvenile osteochondrosis of hip and pelvis, left leg	Arthritis Joint Spine and Hips	Nociceptive Pain
M919	Juvenile osteochondrosis of hip and pelvis, unspecified	Arthritis Joint Spine and Hips	Nociceptive Pain
M9190	Juvenile osteochondrosis of hip and pelvis, unsp, unsp leg	Arthritis Joint Spine and Hips	Nociceptive Pain
M9191	Juvenile osteochondrosis of hip and pelvis, unsp, right leg	Arthritis Joint Spine and Hips	Nociceptive Pain
M9192	Juvenile osteochondrosis of hip and pelvis, unsp, left leg	Arthritis Joint Spine and Hips	Nociceptive Pain
M92	Other juvenile osteochondrosis	Arthritis Joint Other	Nociceptive Pain
M920	Juvenile osteochondrosis of humerus	Arthritis Joint Upper Limb	Nociceptive Pain
M9200	Juvenile osteochondrosis of humerus, unspecified arm	Arthritis Joint Upper Limb	Nociceptive Pain
M9201	Juvenile osteochondrosis of humerus, right arm	Arthritis Joint Upper Limb	Nociceptive Pain
M9202	Juvenile osteochondrosis of humerus, left arm	Arthritis Joint Upper Limb	Nociceptive Pain
M921	Juvenile osteochondrosis of radius and ulna	Arthritis Joint Upper Limb	Nociceptive Pain
M9210	Juvenile osteochondrosis of radius and ulna, unspecified arm	Arthritis Joint Upper Limb	Nociceptive Pain
M9211	Juvenile osteochondrosis of radius and ulna, right arm	Arthritis Joint Upper Limb	Nociceptive Pain
M9212	Juvenile osteochondrosis of radius and ulna, left arm	Arthritis Joint Upper Limb	Nociceptive Pain
M922	Juvenile osteochondrosis, hand	Arthritis Joint Upper Limb	Nociceptive Pain
M9220	Unspecified juvenile osteochondrosis, hand	Arthritis Joint Upper Limb	Nociceptive Pain
M92201	Unspecified juvenile osteochondrosis, right hand	Arthritis Joint Upper Limb	Nociceptive Pain
M92202	Unspecified juvenile osteochondrosis, left hand	Arthritis Joint Upper Limb	Nociceptive Pain
M92209	Unspecified juvenile osteochondrosis, unspecified hand	Arthritis Joint Upper Limb	Nociceptive Pain
M9221	Osteochondrosis (juvenile) of carpal lunate [Kienbock]	Arthritis Joint Other	Nociceptive Pain
M92211	Osteochondrosis (juvenile) of carpal lunate, right hand	Arthritis Joint Upper Limb	Nociceptive Pain

© 2024 Peterson MD et al. *JAMA Neurology.*

M92212	Osteochondrosis (juvenile) of carpal lunate, left hand	Arthritis Joint Upper Limb	Nociceptive Pain
M92219	Osteochondrosis (juvenile) of carpal lunate, unsp hand	Arthritis Joint Upper Limb	Nociceptive Pain
M9222	Osteochondrosis (juvenile) of metacarpal heads [Mauclaire]	Arthritis Joint Upper Limb	Nociceptive Pain
M92221	Osteochondrosis (juvenile) of metacarpal heads, right hand	Arthritis Joint Upper Limb	Nociceptive Pain
M92222	Osteochondrosis (juvenile) of metacarpal heads, left hand	Arthritis Joint Upper Limb	Nociceptive Pain
M92229	Osteochondrosis (juvenile) of metacarpal heads, unsp hand	Arthritis Joint Upper Limb	Nociceptive Pain
M9229	Other juvenile osteochondrosis, hand	Arthritis Joint Upper Limb	Nociceptive Pain
M92291	Other juvenile osteochondrosis, right hand	Arthritis Joint Upper Limb	Nociceptive Pain
M92292	Other juvenile osteochondrosis, left hand	Arthritis Joint Upper Limb	Nociceptive Pain
M92299	Other juvenile osteochondrosis, unspecified hand	Arthritis Joint Upper Limb	Nociceptive Pain
M923	Other juvenile osteochondrosis, upper limb	Arthritis Joint Upper Limb	Nociceptive Pain
M9230	Other juvenile osteochondrosis, unspecified upper limb	Arthritis Joint Upper Limb	Nociceptive Pain
M9231	Other juvenile osteochondrosis, right upper limb	Arthritis Joint Upper Limb	Nociceptive Pain
M9232	Other juvenile osteochondrosis, left upper limb	Arthritis Joint Upper Limb	Nociceptive Pain
M924	Juvenile osteochondrosis of patella	Arthritis Joint Lower Limb	Nociceptive Pain
M9240	Juvenile osteochondrosis of patella, unspecified knee	Arthritis Joint Lower Limb	Nociceptive Pain
M9241	Juvenile osteochondrosis of patella, right knee	Arthritis Joint Lower Limb	Nociceptive Pain
M9242	Juvenile osteochondrosis of patella, left knee	Arthritis Joint Lower Limb	Nociceptive Pain
M925	Juvenile osteochondrosis of tibia and fibula	Arthritis Joint Lower Limb	Nociceptive Pain
M9250	Unspecified juvenile osteochondrosis of tibia and fibula	Arthritis Joint Lower Limb	Nociceptive Pain
M92501	Unspecified juvenile osteochondrosis, right leg	Arthritis Joint Lower Limb	Nociceptive Pain
M92502	Unspecified juvenile osteochondrosis, left leg	Arthritis Joint Lower Limb	Nociceptive Pain
M92503	Unspecified juvenile osteochondrosis, bilateral leg	Arthritis Joint Lower Limb	Nociceptive Pain
M92509	Unspecified juvenile osteochondrosis, unspecified leg	Arthritis Joint Lower Limb	Nociceptive Pain
M9251	Juvenile osteochondrosis of proximal tibia	Arthritis Joint Lower Limb	Nociceptive Pain
M92511	Juvenile osteochondrosis of proximal tibia, right leg	Arthritis Joint Lower Limb	Nociceptive Pain
M92512	Juvenile osteochondrosis of proximal tibia, left leg	Arthritis Joint Lower Limb	Nociceptive Pain
M92513	Juvenile osteochondrosis of proximal tibia, bilateral	Arthritis Joint Lower Limb	Nociceptive Pain
M92519	Juvenile osteochondrosis of proximal tibia, unspecified leg	Arthritis Joint Lower Limb	Nociceptive Pain
M9252	Juvenile osteochondrosis of tibia tubercle	Arthritis Joint Lower Limb	Nociceptive Pain
M92521	Juvenile osteochondrosis of tibia tubercle, right leg	Arthritis Joint Lower Limb	Nociceptive Pain
M92522	Juvenile osteochondrosis of tibia tubercle, left leg	Arthritis Joint Lower Limb	Nociceptive Pain
M92523	Juvenile osteochondrosis of tibia tubercle, bilateral	Arthritis Joint Lower Limb	Nociceptive Pain
M92529	Juvenile osteochondrosis of tibia tubercle, unspecified leg	Arthritis Joint Lower Limb	Nociceptive Pain
M9259	Other juvenile osteochondrosis of tibia and fibula	Arthritis Joint Lower Limb	Nociceptive Pain
M92591	Other juvenile osteochon of tibia and fibula, right leg	Arthritis Joint Lower Limb	Nociceptive Pain
M92592	Other juvenile osteochondrosis of tibia and fibula, left leg	Arthritis Joint Lower Limb	Nociceptive Pain

© 2024 Peterson MD et al. *JAMA Neurology.*

M92593	Other juvenile osteochon of tibia and fibula, bilateral	Arthritis Joint Lower Limb	Nociceptive Pain
M92599	Other juvenile osteochon of tibia and fibula, unsp leg	Arthritis Joint Lower Limb	Nociceptive Pain
M926	Juvenile osteochondrosis of tarsus	Arthritis Joint Lower Limb	Nociceptive Pain
M9260	Juvenile osteochondrosis of tarsus, unspecified ankle	Arthritis Joint Lower Limb	Nociceptive Pain
M9261	Juvenile osteochondrosis of tarsus, right ankle	Arthritis Joint Lower Limb	Nociceptive Pain
M9262	Juvenile osteochondrosis of tarsus, left ankle	Arthritis Joint Lower Limb	Nociceptive Pain
M927	Juvenile osteochondrosis of metatarsus	Arthritis Joint Lower Limb	Nociceptive Pain
M9270	Juvenile osteochondrosis of metatarsus, unspecified foot	Arthritis Joint Lower Limb	Nociceptive Pain
M9271	Juvenile osteochondrosis of metatarsus, right foot	Arthritis Joint Lower Limb	Nociceptive Pain
M9272	Juvenile osteochondrosis of metatarsus, left foot	Arthritis Joint Lower Limb	Nociceptive Pain
M928	Other specified juvenile osteochondrosis	Arthritis Joint Other	Nociceptive Pain
M929	Juvenile osteochondrosis, unspecified	Arthritis Joint Other	Nociceptive Pain
M93	Other osteochondropathies	Arthritis Joint Other	Nociceptive Pain
M930	Slipped upper femoral epiphysis (nontraumatic)	Arthritis Joint Lower Limb	Nociceptive Pain
M9300	Unspecified slipped upper femoral epiphysis (nontraumatic)	Arthritis Joint Lower Limb	Nociceptive Pain
M93001	Unsp slipped upper femoral epiphysis, right hip	Arthritis Joint Spine and Hips	Nociceptive Pain
M93002	Unsp slipped upper femoral epiphysis, left hip	Arthritis Joint Spine and Hips	Nociceptive Pain
M93003	Unsp slipped upper femoral epiphysis, unsp hip	Arthritis Joint Spine and Hips	Nociceptive Pain
M93004	Unspecified slipped upper femoral epiphysis, bilateral hips	Arthritis Joint Spine and Hips	Nociceptive Pain
M9301	Acute slipped upper femoral epiphysis, stable (nontraumatic)	Arthritis Joint Lower Limb	Nociceptive Pain
M93011	Acute slipped upper femoral epiphysis, stable, right hip	Arthritis Joint Spine and Hips	Nociceptive Pain
M93012	Acute slipped upper femoral epiphysis, stable, left hip	Arthritis Joint Spine and Hips	Nociceptive Pain
M93013	Acute slipped upper femoral epiphysis, stable, unsp hip	Arthritis Joint Spine and Hips	Nociceptive Pain
M93014	Acute slipped upper femoral epiphysis, stable, bi hips	Arthritis Joint Spine and Hips	Nociceptive Pain
M9302	Chronic slipped upper femoral epiphysis, stable	Arthritis Joint Lower Limb	Nociceptive Pain
M93021	Chronic slipped upper femoral epiphysis, stable, right hip	Arthritis Joint Spine and Hips	Nociceptive Pain
M93022	Chronic slipped upper femoral epiphysis, stable, left hip	Arthritis Joint Spine and Hips	Nociceptive Pain
M93023	Chronic slipped upper femoral epiphysis, stable, unsp hip	Arthritis Joint Spine and Hips	Nociceptive Pain
M93024	Chronic slipped upper femoral epiphysis, stable, bi hips	Arthritis Joint Spine and Hips	Nociceptive Pain
M9303	Acute on chronic slipped upper femoral epiphysis, stable	Arthritis Joint Lower Limb	Nociceptive Pain
M93031	Acute on chr slipped upper femoral epiphy, stable, r hip	Arthritis Joint Spine and Hips	Nociceptive Pain
M93032	Acute on chr slipped upper femoral epiphy, stable, l hip	Arthritis Joint Spine and Hips	Nociceptive Pain
M93033	Acute on chr slipped upper femoral epiphy, stable, unsp hip	Arthritis Joint Spine and Hips	Nociceptive Pain
M93034	Acute on chr slipped upper femoral epiphy, stable, bi hips	Arthritis Joint Spine and Hips	Nociceptive Pain
M9304	Acute slipped upper femoral epiphysis, unstable	Arthritis Joint Lower Limb	Nociceptive Pain
M93041	Acute slipped upper femoral epiphysis, unstable, right hip	Arthritis Joint Spine and Hips	Nociceptive Pain
M93042	Acute slipped upper femoral epiphysis, unstable, left hip	Arthritis Joint Spine and Hips	Nociceptive Pain

© 2024 Peterson MD et al. *JAMA Neurology.*

M93043	Acute slipped upper femoral epiphysis, unstable, unsp hip	Arthritis Joint Spine and Hips	Nociceptive Pain
M93044	Acute slipped upper femoral epiphysis, unstable, bi hips	Arthritis Joint Spine and Hips	Nociceptive Pain
M9305	Acute on chronic slipped upper femoral epiphysis, unstable	Arthritis Joint Lower Limb	Nociceptive Pain
M93051	Acute on chr slipped upper femoral epiphy, unstbl, r hip	Arthritis Joint Spine and Hips	Nociceptive Pain
M93052	Acute on chr slipped upper femoral epiphy, unstbl, l hip	Arthritis Joint Spine and Hips	Nociceptive Pain
M93053	Acute on chr slipped upper femoral epiphy, unstbl, unsp hip	Arthritis Joint Spine and Hips	Nociceptive Pain
M93054	Acute on chr slipped upper femoral epiphy, unstbl, bi hips	Arthritis Joint Spine and Hips	Nociceptive Pain
M9306	Acute slipped upper femoral epiphysis, unsp stability	Arthritis Joint Lower Limb	Nociceptive Pain
M93061	Acute slipped upper femoral epiphy, unsp stability, r hip	Arthritis Joint Spine and Hips	Nociceptive Pain
M93062	Acute slipped upper femoral epiphy, unsp stability, l hip	Arthritis Joint Spine and Hips	Nociceptive Pain
M93063	Acute slipped upper fem epiphy, unsp stability, unsp hip	Arthritis Joint Spine and Hips	Nociceptive Pain
M93064	Acute slipped upper femoral epiphy, unsp stability, bi hips	Arthritis Joint Spine and Hips	Nociceptive Pain
M9307	Acute on chr slipped upper femoral epiphy, unsp stability	Arthritis Joint Lower Limb	Nociceptive Pain
M93071	Acute on chr slip upper fem epiphy, unsp stability, r hip	Arthritis Joint Spine and Hips	Nociceptive Pain
M93072	Acute on chr slip upper fem epiphy, unsp stability, l hip	Arthritis Joint Spine and Hips	Nociceptive Pain
M93073	Ac on chr slip upper fem epiphy, unsp stability, unsp hip	Arthritis Joint Spine and Hips	Nociceptive Pain
M93074	Acute on chr slip upper fem epiphy, unsp stability, bi hips	Arthritis Joint Spine and Hips	Nociceptive Pain
M931	Kienbock's disease of adults	Arthritis Joint Upper Limb	Nociceptive Pain
M932	Osteochondritis dissecans	Arthritis Joint Other	Nociceptive Pain
M9320	Osteochondritis dissecans of unspecified site	Arthritis Joint Other	Nociceptive Pain
M9321	Osteochondritis dissecans of shoulder	Arthritis Joint Upper Limb	Nociceptive Pain
M93211	Osteochondritis dissecans, right shoulder	Arthritis Joint Upper Limb	Nociceptive Pain
M93212	Osteochondritis dissecans, left shoulder	Arthritis Joint Upper Limb	Nociceptive Pain
M93219	Osteochondritis dissecans, unspecified shoulder	Arthritis Joint Upper Limb	Nociceptive Pain
M9322	Osteochondritis dissecans of elbow	Arthritis Joint Upper Limb	Nociceptive Pain
M93221	Osteochondritis dissecans, right elbow	Arthritis Joint Upper Limb	Nociceptive Pain
M93222	Osteochondritis dissecans, left elbow	Arthritis Joint Upper Limb	Nociceptive Pain
M93229	Osteochondritis dissecans, unspecified elbow	Arthritis Joint Upper Limb	Nociceptive Pain
M9323	Osteochondritis dissecans of wrist	Arthritis Joint Upper Limb	Nociceptive Pain
M93231	Osteochondritis dissecans, right wrist	Arthritis Joint Upper Limb	Nociceptive Pain
M93232	Osteochondritis dissecans, left wrist	Arthritis Joint Upper Limb	Nociceptive Pain
M93239	Osteochondritis dissecans, unspecified wrist	Arthritis Joint Upper Limb	Nociceptive Pain
M9324	Osteochondritis dissecans of joints of hand	Arthritis Joint Upper Limb	Nociceptive Pain
M93241	Osteochondritis dissecans, joints of right hand	Arthritis Joint Upper Limb	Nociceptive Pain
M93242	Osteochondritis dissecans, joints of left hand	Arthritis Joint Upper Limb	Nociceptive Pain
M93249	Osteochondritis dissecans, joints of unspecified hand	Arthritis Joint Upper Limb	Nociceptive Pain
M9325	Osteochondritis dissecans of hip	Arthritis Joint Spine and Hips	Nociceptive Pain

© 2024 Peterson MD et al. *JAMA Neurology.*

M93251	Osteochondritis dissecans, right hip	Arthritis Joint Spine and Hips	Nociceptive Pain
M93252	Osteochondritis dissecans, left hip	Arthritis Joint Spine and Hips	Nociceptive Pain
M93259	Osteochondritis dissecans, unspecified hip	Arthritis Joint Spine and Hips	Nociceptive Pain
M9326	Osteochondritis dissecans knee	Arthritis Joint Lower Limb	Nociceptive Pain
M93261	Osteochondritis dissecans, right knee	Arthritis Joint Lower Limb	Nociceptive Pain
M93262	Osteochondritis dissecans, left knee	Arthritis Joint Lower Limb	Nociceptive Pain
M93269	Osteochondritis dissecans, unspecified knee	Arthritis Joint Lower Limb	Nociceptive Pain
M9327	Osteochondritis dissecans of ankle and joints of foot	Arthritis Joint Lower Limb	Nociceptive Pain
M93271	Osteochondritis dissecans, r ankle and joints of right foot	Arthritis Joint Lower Limb	Nociceptive Pain
M93272	Osteochondritis dissecans, l ankle and joints of left foot	Arthritis Joint Lower Limb	Nociceptive Pain
M93279	Osteochondritis dissecans, unsp ankle and joints of foot	Arthritis Joint Lower Limb	Nociceptive Pain
M9328	Osteochondritis dissecans other site	Arthritis Joint Other	Nociceptive Pain
M9329	Osteochondritis dissecans multiple sites	Arthritis Joint Other	Nociceptive Pain
M938	Other specified osteochondropathies	Arthritis Joint Other	Nociceptive Pain
M9380	Other specified osteochondropathies of unspecified site	Arthritis Joint Other	Nociceptive Pain
M9381	Other specified osteochondropathies of shoulder	Arthritis Joint Upper Limb	Nociceptive Pain
M93811	Other specified osteochondropathies, right shoulder	Arthritis Joint Upper Limb	Nociceptive Pain
M93812	Other specified osteochondropathies, left shoulder	Arthritis Joint Upper Limb	Nociceptive Pain
M93819	Other specified osteochondropathies, unspecified shoulder	Arthritis Joint Upper Limb	Nociceptive Pain
M9382	Other specified osteochondropathies of upper arm	Arthritis Joint Upper Limb	Nociceptive Pain
M93821	Other specified osteochondropathies, right upper arm	Arthritis Joint Upper Limb	Nociceptive Pain
M93822	Other specified osteochondropathies, left upper arm	Arthritis Joint Upper Limb	Nociceptive Pain
M93829	Other specified osteochondropathies, unspecified upper arm	Arthritis Joint Upper Limb	Nociceptive Pain
M9383	Other specified osteochondropathies of forearm	Arthritis Joint Upper Limb	Nociceptive Pain
M93831	Other specified osteochondropathies, right forearm	Arthritis Joint Upper Limb	Nociceptive Pain
M93832	Other specified osteochondropathies, left forearm	Arthritis Joint Upper Limb	Nociceptive Pain
M93839	Other specified osteochondropathies, unspecified forearm	Arthritis Joint Upper Limb	Nociceptive Pain
M9384	Other specified osteochondropathies of hand	Arthritis Joint Upper Limb	Nociceptive Pain
M93841	Other specified osteochondropathies, right hand	Arthritis Joint Upper Limb	Nociceptive Pain
M93842	Other specified osteochondropathies, left hand	Arthritis Joint Upper Limb	Nociceptive Pain
M93849	Other specified osteochondropathies, unspecified hand	Arthritis Joint Upper Limb	Nociceptive Pain
M9385	Other specified osteochondropathies of thigh	Arthritis Joint Lower Limb	Nociceptive Pain
M93851	Other specified osteochondropathies, right thigh	Arthritis Joint Lower Limb	Nociceptive Pain
M93852	Other specified osteochondropathies, left thigh	Arthritis Joint Lower Limb	Nociceptive Pain
M93859	Other specified osteochondropathies, unspecified thigh	Arthritis Joint Lower Limb	Nociceptive Pain
M9386	Other specified osteochondropathies lower leg	Arthritis Joint Lower Limb	Nociceptive Pain
M93861	Other specified osteochondropathies, right lower leg	Arthritis Joint Lower Limb	Nociceptive Pain

© 2024 Peterson MD et al. *JAMA Neurology.*

M93862	Other specified osteochondropathies, left lower leg	Arthritis Joint Lower Limb	Nociceptive Pain
M93869	Other specified osteochondropathies, unspecified lower leg	Arthritis Joint Lower Limb	Nociceptive Pain
M9387	Other specified osteochondropathies of ankle and foot	Arthritis Joint Lower Limb	Nociceptive Pain
M93871	Other specified osteochondropathies, right ankle and foot	Arthritis Joint Lower Limb	Nociceptive Pain
M93872	Other specified osteochondropathies, left ankle and foot	Arthritis Joint Lower Limb	Nociceptive Pain
M93879	Oth osteochondropathies, unspecified ankle and foot	Arthritis Joint Lower Limb	Nociceptive Pain
M9388	Other specified osteochondropathies other	Arthritis Joint Other	Nociceptive Pain
M9389	Other specified osteochondropathies multiple sites	Arthritis Joint Other	Nociceptive Pain
M939	Osteochondropathy, unspecified	Arthritis Joint Other	Nociceptive Pain
M9390	Osteochondropathy, unspecified of unspecified site	Arthritis Joint Other	Nociceptive Pain
M9391	Osteochondropathy, unspecified of shoulder	Arthritis Joint Upper Limb	Nociceptive Pain
M93911	Osteochondropathy, unspecified, right shoulder	Arthritis Joint Upper Limb	Nociceptive Pain
M93912	Osteochondropathy, unspecified, left shoulder	Arthritis Joint Upper Limb	Nociceptive Pain
M93919	Osteochondropathy, unspecified, unspecified shoulder	Arthritis Joint Upper Limb	Nociceptive Pain
M9392	Osteochondropathy, unspecified of upper arm	Arthritis Joint Upper Limb	Nociceptive Pain
M93921	Osteochondropathy, unspecified, right upper arm	Arthritis Joint Upper Limb	Nociceptive Pain
M93922	Osteochondropathy, unspecified, left upper arm	Arthritis Joint Upper Limb	Nociceptive Pain
M93929	Osteochondropathy, unspecified, unspecified upper arm	Arthritis Joint Upper Limb	Nociceptive Pain
M9393	Osteochondropathy, unspecified of forearm	Arthritis Joint Upper Limb	Nociceptive Pain
M93931	Osteochondropathy, unspecified, right forearm	Arthritis Joint Upper Limb	Nociceptive Pain
M93932	Osteochondropathy, unspecified, left forearm	Arthritis Joint Upper Limb	Nociceptive Pain
M93939	Osteochondropathy, unspecified, unspecified forearm	Arthritis Joint Upper Limb	Nociceptive Pain
M9394	Osteochondropathy, unspecified of hand	Arthritis Joint Upper Limb	Nociceptive Pain
M93941	Osteochondropathy, unspecified, right hand	Arthritis Joint Upper Limb	Nociceptive Pain
M93942	Osteochondropathy, unspecified, left hand	Arthritis Joint Upper Limb	Nociceptive Pain
M93949	Osteochondropathy, unspecified, unspecified hand	Arthritis Joint Upper Limb	Nociceptive Pain
M9395	Osteochondropathy, unspecified of thigh	Arthritis Joint Lower Limb	Nociceptive Pain
M93951	Osteochondropathy, unspecified, right thigh	Arthritis Joint Lower Limb	Nociceptive Pain
M93952	Osteochondropathy, unspecified, left thigh	Arthritis Joint Lower Limb	Nociceptive Pain
M93959	Osteochondropathy, unspecified, unspecified thigh	Arthritis Joint Lower Limb	Nociceptive Pain
M9396	Osteochondropathy, unspecified lower leg	Arthritis Joint Lower Limb	Nociceptive Pain
M93961	Osteochondropathy, unspecified, right lower leg	Arthritis Joint Lower Limb	Nociceptive Pain
M93962	Osteochondropathy, unspecified, left lower leg	Arthritis Joint Lower Limb	Nociceptive Pain
M93969	Osteochondropathy, unspecified, unspecified lower leg	Arthritis Joint Lower Limb	Nociceptive Pain
M9397	Osteochondropathy, unspecified of ankle and foot	Arthritis Joint Lower Limb	Nociceptive Pain
M93971	Osteochondropathy, unspecified, right ankle and foot	Arthritis Joint Lower Limb	Nociceptive Pain
M93972	Osteochondropathy, unspecified, left ankle and foot	Arthritis Joint Lower Limb	Nociceptive Pain

© 2024 Peterson MD et al. *JAMA Neurology.*

M93979	Osteochondropathy, unspecified, unspecified ankle and foot	Arthritis Joint Lower Limb	Nociceptive Pain
M9398	Osteochondropathy, unspecified other	Arthritis Joint Other	Nociceptive Pain
M9399	Osteochondropathy, unspecified multiple sites	Arthritis Joint Other	Nociceptive Pain
M94	Other disorders of cartilage	Arthritis Joint Other	Nociceptive Pain
M940	Chondrocostal junction syndrome [Tietze]	Arthritis Joint Other	Nociceptive Pain
M941	Relapsing polychondritis	Arthritis Joint Other	Nociceptive Pain
M942	Chondromalacia	Arthritis Joint Other	Nociceptive Pain
M9420	Chondromalacia, unspecified site	Arthritis Joint Other	Nociceptive Pain
M9421	Chondromalacia, shoulder	Arthritis Joint Upper Limb	Nociceptive Pain
M94211	Chondromalacia, right shoulder	Arthritis Joint Upper Limb	Nociceptive Pain
M94212	Chondromalacia, left shoulder	Arthritis Joint Upper Limb	Nociceptive Pain
M94219	Chondromalacia, unspecified shoulder	Arthritis Joint Upper Limb	Nociceptive Pain
M9422	Chondromalacia, elbow	Arthritis Joint Upper Limb	Nociceptive Pain
M94221	Chondromalacia, right elbow	Arthritis Joint Upper Limb	Nociceptive Pain
M94222	Chondromalacia, left elbow	Arthritis Joint Upper Limb	Nociceptive Pain
M94229	Chondromalacia, unspecified elbow	Arthritis Joint Upper Limb	Nociceptive Pain
M9423	Chondromalacia, wrist	Arthritis Joint Upper Limb	Nociceptive Pain
M94231	Chondromalacia, right wrist	Arthritis Joint Upper Limb	Nociceptive Pain
M94232	Chondromalacia, left wrist	Arthritis Joint Upper Limb	Nociceptive Pain
M94239	Chondromalacia, unspecified wrist	Arthritis Joint Upper Limb	Nociceptive Pain
M9424	Chondromalacia, joints of hand	Arthritis Joint Upper Limb	Nociceptive Pain
M94241	Chondromalacia, joints of right hand	Arthritis Joint Upper Limb	Nociceptive Pain
M94242	Chondromalacia, joints of left hand	Arthritis Joint Upper Limb	Nociceptive Pain
M94249	Chondromalacia, joints of unspecified hand	Arthritis Joint Upper Limb	Nociceptive Pain
M9425	Chondromalacia, hip	Arthritis Joint Spine and Hips	Nociceptive Pain
M94251	Chondromalacia, right hip	Arthritis Joint Spine and Hips	Nociceptive Pain
M94252	Chondromalacia, left hip	Arthritis Joint Spine and Hips	Nociceptive Pain
M94259	Chondromalacia, unspecified hip	Arthritis Joint Spine and Hips	Nociceptive Pain
M9426	Chondromalacia, knee	Arthritis Joint Lower Limb	Nociceptive Pain
M94261	Chondromalacia, right knee	Arthritis Joint Lower Limb	Nociceptive Pain
M94262	Chondromalacia, left knee	Arthritis Joint Lower Limb	Nociceptive Pain
M94269	Chondromalacia, unspecified knee	Arthritis Joint Lower Limb	Nociceptive Pain
M9427	Chondromalacia, ankle and joints of foot	Arthritis Joint Lower Limb	Nociceptive Pain
M94271	Chondromalacia, right ankle and joints of right foot	Arthritis Joint Lower Limb	Nociceptive Pain
M94272	Chondromalacia, left ankle and joints of left foot	Arthritis Joint Lower Limb	Nociceptive Pain
M94279	Chondromalacia, unspecified ankle and joints of foot	Arthritis Joint Lower Limb	Nociceptive Pain
M9428	Chondromalacia, other site	Arthritis Joint Other	Nociceptive Pain

© 2024 Peterson MD et al. *JAMA Neurology.*

M9429	Chondromalacia, multiple sites	Arthritis Joint Other	Nociceptive Pain
M943	Chondrolysis	Arthritis Joint Other	Nociceptive Pain
M9435	Chondrolysis, hip	Arthritis Joint Spine and Hips	Nociceptive Pain
M94351	Chondrolysis, right hip	Arthritis Joint Spine and Hips	Nociceptive Pain
M94352	Chondrolysis, left hip	Arthritis Joint Spine and Hips	Nociceptive Pain
M94359	Chondrolysis, unspecified hip	Arthritis Joint Spine and Hips	Nociceptive Pain
M948	Other specified disorders of cartilage	Arthritis Joint Other	Nociceptive Pain
M948X	Other specified disorders of cartilage	Arthritis Joint Other	Nociceptive Pain
M948X0	Other specified disorders of cartilage, multiple sites	Arthritis Joint Other	Nociceptive Pain
M948X1	Other specified disorders of cartilage, shoulder	Arthritis Joint Upper Limb	Nociceptive Pain
M948X2	Other specified disorders of cartilage, upper arm	Arthritis Joint Upper Limb	Nociceptive Pain
M948X3	Other specified disorders of cartilage, forearm	Arthritis Joint Upper Limb	Nociceptive Pain
M948X4	Other specified disorders of cartilage, hand	Arthritis Joint Upper Limb	Nociceptive Pain
M948X5	Other specified disorders of cartilage, thigh	Arthritis Joint Lower Limb	Nociceptive Pain
M948X6	Other specified disorders of cartilage, lower leg	Arthritis Joint Lower Limb	Nociceptive Pain
M948X7	Other specified disorders of cartilage, ankle and foot	Arthritis Joint Lower Limb	Nociceptive Pain
M948X8	Other specified disorders of cartilage, other site	Arthritis Joint Other	Nociceptive Pain
M948X9	Other specified disorders of cartilage, unspecified sites	Arthritis Joint Other	Nociceptive Pain
M949	Disorder of cartilage, unspecified	Arthritis Joint Other	Nociceptive Pain
M95	Oth acquired deformities of ms sys and connective tissue	Arthritis Joint Other	Nociceptive Pain
M950	Acquired deformity of nose	Arthritis Joint Other	Nociceptive Pain
M951	Cauliflower ear	Arthritis Joint Other	Nociceptive Pain
M9510	Cauliflower ear, unspecified ear	Arthritis Joint Other	Nociceptive Pain
M9511	Cauliflower ear, right ear	Arthritis Joint Other	Nociceptive Pain
M9512	Cauliflower ear, left ear	Arthritis Joint Other	Nociceptive Pain
M952	Other acquired deformity of head	Arthritis Joint Other	Nociceptive Pain
M953	Acquired deformity of neck	Arthritis Joint Spine and Hips	Nociceptive Pain
M954	Acquired deformity of chest and rib	Arthritis Joint Other	Nociceptive Pain
M955	Acquired deformity of pelvis	Arthritis Joint Spine and Hips	Nociceptive Pain
M958	Oth acquired deformities of musculoskeletal system	Arthritis Joint Other	Nociceptive Pain
M959	Acquired deformity of musculoskeletal system, unspecified	Arthritis Joint Other	Nociceptive Pain
M961	Postlaminectomy syndrome, not elsewhere classified	Back Pain Other/Unspecified	Nociceptive Pain
M962	Postradiation kyphosis	Back Pain Other/Unspecified	Nociceptive Pain
M963	Postlaminectomy kyphosis	Back Pain Other/Unspecified	Nociceptive Pain
M964	Postsurgical lordosis	Arthritis Joint Other	Nociceptive Pain
M965	Postradiation scoliosis	Back Pain Other/Unspecified	Nociceptive Pain
M9901	Segmental and somatic dysfunction of cervical region	Neck Pain	Nociceptive Pain

© 2024 Peterson MD et al. *JAMA Neurology.*

M9902	Segmental and somatic dysfunction of thoracic region	Back Pain Mid Back	Nociceptive Pain
M9903	Segmental and somatic dysfunction of lumbar region	Back Pain Low Back	Nociceptive Pain
M9904	Segmental and somatic dysfunction of sacral region	Back Pain Low Back	Nociceptive Pain
M9983	Other biomechanical lesions of lumbar region	Back Pain Low Back	Nociceptive Pain
M9984	Other biomechanical lesions of sacral region	Back Pain Low Back	Nociceptive Pain
Q762	Congenital spondylolisthesis	Back Pain Other/Unspecified	Nociceptive Pain
Q764	Oth congenital malform of spine, not associated w scoliosis	Back Pain Other/Unspecified	Nociceptive Pain
R252	Cramp and spasm	Arthritis Joint Other	Nociceptive Pain
R262	Difficulty in walking, not elsewhere classified	Arthritis Joint Other	Nociceptive Pain
R294	Clicking hip	Arthritis Joint Spine and Hips	Nociceptive Pain
R29898	Oth symptoms and signs involving the musculoskeletal system	Arthritis Joint Other	Nociceptive Pain
S0291XK	Unsp fracture of skull, subs encntr for fracture w nonunion	Arthritis Joint Other	Nociceptive Pain
S0292XK	Unsp fracture of facial bones, subs for fx w nonunion	Arthritis Joint Other	Nociceptive Pain
S12000K	Unsp disp fx of first cervcal vert, subs for fx w nonunion	Arthritis Joint Spine and Hips	Nociceptive Pain
S12001K	Unsp nondisp fx of 1st cervcal vert, subs for fx w nonunion	Arthritis Joint Spine and Hips	Nociceptive Pain
S12100K	Unsp disp fx of second cervcal vert, subs for fx w nonunion	Arthritis Joint Spine and Hips	Nociceptive Pain
S12101K	Unsp nondisp fx of 2nd cervcal vert, subs for fx w nonunion	Arthritis Joint Spine and Hips	Nociceptive Pain
S12200K	Unsp disp fx of third cervcal vert, subs for fx w nonunion	Arthritis Joint Spine and Hips	Nociceptive Pain
S12201K	Unsp nondisp fx of 3rd cervcal vert, subs for fx w nonunion	Arthritis Joint Spine and Hips	Nociceptive Pain
S12300K	Unsp disp fx of fourth cervcal vert, subs for fx w nonunion	Arthritis Joint Spine and Hips	Nociceptive Pain
S12301K	Unsp nondisp fx of 4th cervcal vert, subs for fx w nonunion	Arthritis Joint Spine and Hips	Nociceptive Pain
S12400K	Unsp disp fx of fifth cervcal vert, subs for fx w nonunion	Arthritis Joint Spine and Hips	Nociceptive Pain
S12401K	Unsp nondisp fx of 5th cervcal vert, subs for fx w nonunion	Arthritis Joint Spine and Hips	Nociceptive Pain
S12500K	Unsp disp fx of sixth cervcal vert, subs for fx w nonunion	Arthritis Joint Spine and Hips	Nociceptive Pain
S12501K	Unsp nondisp fx of sixth cervcal vert, 7thK	Arthritis Joint Spine and Hips	Nociceptive Pain
S12600K	Unsp disp fx of seventh cervcal vert, subs for fx w nonunion	Arthritis Joint Spine and Hips	Nociceptive Pain
S12601K	Unsp nondisp fx of 7th cervcal vert, subs for fx w nonunion	Arthritis Joint Spine and Hips	Nociceptive Pain
S13101A	Dislocation of unspecified cervical vertebrae, init encntr	Neck Pain	Nociceptive Pain
S13111A	Dislocation of C0/C1 cervical vertebrae, initial encounter	Neck Pain	Nociceptive Pain
S13121A	Dislocation of C1/C2 cervical vertebrae, initial encounter	Neck Pain	Nociceptive Pain
S13131A	Dislocation of C2/C3 cervical vertebrae, initial encounter	Neck Pain	Nociceptive Pain
S13141A	Dislocation of C3/C4 cervical vertebrae, initial encounter	Neck Pain	Nociceptive Pain
S13151A	Dislocation of C4/C5 cervical vertebrae, initial encounter	Neck Pain	Nociceptive Pain
S13161A	Dislocation of C5/C6 cervical vertebrae, initial encounter	Neck Pain	Nociceptive Pain
S13171A	Dislocation of C6/C7 cervical vertebrae, initial encounter	Neck Pain	Nociceptive Pain
S13181A	Dislocation of C7/T1 cervical vertebrae, initial encounter	Neck Pain	Nociceptive Pain
S134XXA	Sprain of ligaments of cervical spine, initial encounter	Neck Pain	Nociceptive Pain

© 2024 Peterson MD et al. *JAMA Neurology.*

S138XXA	Sprain of joints and ligaments of oth prt neck, init encntr	Neck Pain	Nociceptive Pain
S22009A	Unsp fracture of unsp thoracic vertebra, init for clos fx	Back Pain Mid Back	Nociceptive Pain
S229XXK	Fracture of bony thorax, part unsp, subs for fx w nonunion	Arthritis Joint Other	Nociceptive Pain
S23101A	Dislocation of unspecified thoracic vertebra, init encntr	Back Pain Mid Back	Nociceptive Pain
S23111A	Dislocation of T1/T2 thoracic vertebra, initial encounter	Back Pain Mid Back	Nociceptive Pain
S23121A	Dislocation of T2/T3 thoracic vertebra, initial encounter	Back Pain Mid Back	Nociceptive Pain
S23123A	Dislocation of T3/T4 thoracic vertebra, initial encounter	Back Pain Mid Back	Nociceptive Pain
S23131A	Dislocation of T4/T5 thoracic vertebra, initial encounter	Back Pain Mid Back	Nociceptive Pain
S23133A	Dislocation of T5/T6 thoracic vertebra, initial encounter	Back Pain Mid Back	Nociceptive Pain
S23141A	Dislocation of T6/T7 thoracic vertebra, initial encounter	Back Pain Mid Back	Nociceptive Pain
S23143A	Dislocation of T7/T8 thoracic vertebra, initial encounter	Back Pain Mid Back	Nociceptive Pain
S23151A	Dislocation of T8/T9 thoracic vertebra, initial encounter	Back Pain Mid Back	Nociceptive Pain
S23153A	Dislocation of T9/T10 thoracic vertebra, initial encounter	Back Pain Mid Back	Nociceptive Pain
S23161A	Dislocation of T10/T11 thoracic vertebra, initial encounter	Back Pain Mid Back	Nociceptive Pain
S23163A	Dislocation of T11/T12 thoracic vertebra, initial encounter	Back Pain Mid Back	Nociceptive Pain
S23171A	Dislocation of T12/L1 thoracic vertebra, initial encounter	Back Pain Mid Back	Nociceptive Pain
S233XXA	Sprain of ligaments of thoracic spine, initial encounter	Back Pain Mid Back	Nociceptive Pain
S238XXA	Sprain of other specified parts of thorax, initial encounter	Back Pain Mid Back	Nociceptive Pain
S239XXA	Sprain of unspecified parts of thorax, initial encounter	Back Pain Mid Back	Nociceptive Pain
S32009A	Unsp fracture of unsp lumbar vertebra, init for clos fx	Back Pain Low Back	Nociceptive Pain
S32010A	Wedge compression fracture of first lumbar vertebra, init	Back Pain Low Back	Nociceptive Pain
S322XXA	Fracture of coccyx, initial encounter for closed fracture	Back Pain Low Back	Nociceptive Pain
S329XXK	Fx unsp parts of lumbosacr spin & pelv, 7thK	Arthritis Joint Spine and Hips	Nociceptive Pain
S33101A	Dislocation of unspecified lumbar vertebra, init encntr	Back Pain Low Back	Nociceptive Pain
S332XXA	Dislocation of sacroiliac and sacrococcygeal joint, init	Back Pain Low Back	Nociceptive Pain
S335XXA	Sprain of ligaments of lumbar spine, initial encounter	Back Pain Low Back	Nociceptive Pain
S336XXA	Sprain of sacroiliac joint, initial encounter	Back Pain Low Back	Nociceptive Pain
S338XXA	Sprain of oth parts of lumbar spine and pelvis, init encntr	Back Pain Low Back	Nociceptive Pain
S339XXA	Sprain of unsp parts of lumbar spine and pelvis, init encntr	Back Pain Low Back	Nociceptive Pain
S42009K	Fx unsp part of unsp clavicle, subs for fx w nonunion	Arthritis Joint Spine and Hips	Nociceptive Pain
S42009P	Fx unsp part of unsp clavicle, subs for fx w malunion	Arthritis Joint Spine and Hips	Nociceptive Pain
S42209K	Unsp fx upper end of unsp humerus, subs for fx w nonunion	Arthritis Joint Upper Limb	Nociceptive Pain
S42209P	Unsp fx upper end of unsp humerus, subs for fx w malunion	Arthritis Joint Upper Limb	Nociceptive Pain
S4290XK	Fx unsp shoulder girdle, part unsp, subs for fx w nonunion	Arthritis Joint Upper Limb	Nociceptive Pain
S4290XP	Fx unsp shoulder girdle, part unsp, subs for fx w malunion	Arthritis Joint Upper Limb	Nociceptive Pain
S5290XK	Unsp fracture of unsp forearm, subs for clos fx w nonunion	Arthritis Joint Upper Limb	Nociceptive Pain
S5290XM	Unsp fx unsp forearm, subs for opn fx type I/2 w nonunion	Arthritis Joint Upper Limb	Nociceptive Pain

© 2024 Peterson MD et al. *JAMA Neurology.*

S5290XN	Unsp fx unsp forearm, subs for opn fx type 3A/B/C w nonunion	Arthritis Joint Upper Limb	Nociceptive Pain
S5290XP	Unsp fracture of unsp forearm, subs for clos fx w malunion	Arthritis Joint Upper Limb	Nociceptive Pain
S5290XQ	Unsp fx unsp forearm, subs for opn fx type I/2 w malunion	Arthritis Joint Upper Limb	Nociceptive Pain
S5290XR	Unsp fx unsp forearm, subs for opn fx type 3A/B/C w malunion	Arthritis Joint Upper Limb	Nociceptive Pain
S6290XK	Unsp fracture of unsp wrist and hand, subs for fx w nonunion	Arthritis Joint Upper Limb	Nociceptive Pain
S6290XP	Unsp fracture of unsp wrist and hand, subs for fx w malunion	Arthritis Joint Upper Limb	Nociceptive Pain
S7290XK	Unsp fracture of unsp femur, subs for clos fx w nonunion	Arthritis Joint Lower Limb	Nociceptive Pain
S7290XM	Unsp fx unsp femur, subs for opn fx type I/2 w nonunion	Arthritis Joint Lower Limb	Nociceptive Pain
S7290XN	Unsp fx unsp femur, subs for opn fx type 3A/B/C w nonunion	Arthritis Joint Lower Limb	Nociceptive Pain
S7290XP	Unsp fracture of unsp femur, subs for clos fx w malunion	Arthritis Joint Lower Limb	Nociceptive Pain
S7290XQ	Unsp fx unsp femur, subs for opn fx type I/2 w malunion	Arthritis Joint Lower Limb	Nociceptive Pain
S7290XR	Unsp fx unsp femur, subs for opn fx type 3A/B/C w malunion	Arthritis Joint Lower Limb	Nociceptive Pain
S82009P	Unsp fracture of unsp patella, subs for clos fx w malunion	Arthritis Joint Lower Limb	Nociceptive Pain
S82009Q	Unsp fx unsp patella, subs for opn fx type I/2 w malunion	Arthritis Joint Lower Limb	Nociceptive Pain
S82009R	Unsp fx unsp patella, subs for opn fx type 3A/B/C w malunion	Arthritis Joint Lower Limb	Nociceptive Pain
S8290XK	Unsp fracture of unsp lower leg, subs for clos fx w nonunion	Arthritis Joint Lower Limb	Nociceptive Pain
S8290XM	Unsp fx unsp lower leg, subs for opn fx type I/2 w nonunion	Arthritis Joint Lower Limb	Nociceptive Pain
S8290XN	Unsp fx unsp low leg, subs for opn fx type 3A/B/C w nonunion	Arthritis Joint Lower Limb	Nociceptive Pain
S8290XP	Unsp fracture of unsp lower leg, subs for clos fx w malunion	Arthritis Joint Lower Limb	Nociceptive Pain
S8290XQ	Unsp fx unsp lower leg, subs for opn fx type I/2 w malunion	Arthritis Joint Lower Limb	Nociceptive Pain
S8290XR	Unsp fx unsp low leg, subs for opn fx type 3A/B/C w malunion	Arthritis Joint Lower Limb	Nociceptive Pain
S92819K	Other fracture of unspecified foot, 7thK	Arthritis Joint Lower Limb	Nociceptive Pain
S92819P	Other fracture of unspecified foot, 7thP	Arthritis Joint Lower Limb	Nociceptive Pain
S92909K	Unsp fracture of unsp foot, subs for fx w nonunion	Arthritis Joint Lower Limb	Nociceptive Pain
S92909P	Unsp fracture of unsp foot, subs for fx w malunion	Arthritis Joint Lower Limb	Nociceptive Pain
S92919K	Unsp fracture of unsp toe(s), subs for fx w nonunion	Arthritis Joint Lower Limb	Nociceptive Pain
S92919P	Unsp fracture of unsp toe(s), subs for fx w malunion	Arthritis Joint Lower Limb	Nociceptive Pain
S99209K	Unspecified physeal fx phalanx of unspecified toe, 7thK	Arthritis Joint Lower Limb	Nociceptive Pain
S99209P	Unspecified physeal fx phalanx of unspecified toe, 7thP	Arthritis Joint Lower Limb	Nociceptive Pain
S99219K	Sltr-haris Type I physeal fx phalanx of unsp toe, 7thK	Arthritis Joint Lower Limb	Nociceptive Pain
S99219P	Sltr-haris Type I physeal fx phalanx of unsp toe, 7thP	Arthritis Joint Lower Limb	Nociceptive Pain
S99229K	Sltr-haris Type II physeal fx phalanx of unsp toe, 7thK	Arthritis Joint Lower Limb	Nociceptive Pain
S99229P	Sltr-haris Type II physeal fx phalanx of unsp toe, 7thP	Arthritis Joint Lower Limb	Nociceptive Pain
S99239K	Sltr-haris Type III physeal fx phalanx of unsp toe, 7thK	Arthritis Joint Lower Limb	Nociceptive Pain

© 2024 Peterson MD et al. *JAMA Neurology.*

S99239P	Sltr-haris Type III physeal fx phalanx of unsp toe, 7thP	Arthritis Joint Lower Limb	Nociceptive Pain
S99249K	Sltr-haris Type IV physeal fx phalanx of unsp toe, 7thK	Arthritis Joint Lower Limb	Nociceptive Pain
S99249P	Sltr-haris Type IV physeal fx phalanx of unsp toe, 7thP	Arthritis Joint Lower Limb	Nociceptive Pain
S99299K	Other physeal fracture of phalanx of unspecified toe, 7thK	Arthritis Joint Lower Limb	Nociceptive Pain
S99299P	Other physeal fracture of phalanx of unspecified toe, 7thP	Arthritis Joint Lower Limb	Nociceptive Pain
G43	Migraine	Migrane	Nociplastic Pain
G430	Migraine without aura	Migrane	Nociplastic Pain
G4300	Migraine without aura, not intractable	Migrane	Nociplastic Pain
G43001	Migraine w/o aura, not intractable, with status migrainosus	Migrane	Nociplastic Pain
G43009	Migraine w/o aura, not intractable, w/o status migrainosus	Migrane	Nociplastic Pain
G4301	Migraine without aura, intractable	Migrane	Nociplastic Pain
G43011	Migraine without aura, intractable, with status migrainosus	Migrane	Nociplastic Pain
G43019	Migraine w/o aura, intractable, without status migrainosus	Migrane	Nociplastic Pain
G431	Migraine with aura	Migrane	Nociplastic Pain
G4310	Migraine with aura, not intractable	Migrane	Nociplastic Pain
G43101	Migraine with aura, not intractable, with status migrainosus	Migrane	Nociplastic Pain
G43109	Migraine with aura, not intractable, w/o status migrainosus	Migrane	Nociplastic Pain
G4311	Migraine with aura, intractable	Migrane	Nociplastic Pain
G43111	Migraine with aura, intractable, with status migrainosus	Migrane	Nociplastic Pain
G43119	Migraine with aura, intractable, without status migrainosus	Migrane	Nociplastic Pain
G434	Hemiplegic migraine	Migrane	Nociplastic Pain
G4340	Hemiplegic migraine, not intractable	Migrane	Nociplastic Pain
G43401	Hemiplegic migraine, not intractable, w status migrainosus	Migrane	Nociplastic Pain
G43409	Hemiplegic migraine, not intractable, w/o status migrainosus	Migrane	Nociplastic Pain
G4341	Hemiplegic migraine, intractable	Migrane	Nociplastic Pain
G43411	Hemiplegic migraine, intractable, with status migrainosus	Migrane	Nociplastic Pain
G43419	Hemiplegic migraine, intractable, without status migrainosus	Migrane	Nociplastic Pain
G44201	Tension-type headache, unspecified, intractable	Tension Headache	Nociplastic Pain
G44209	Tension-type headache, unspecified, not intractable	Tension Headache	Nociplastic Pain
G905	Complex regional pain syndrome I (CRPS I)	Myalgia and myositis, unspecified (Fibromyalgia)	Nociplastic Pain
K224	Dyskinesia of esophagus	Dyskinesia of esophagus	Nociplastic Pain
K30	Functional dyspepsia	Dyspepsia	Nociplastic Pain
K58	Irritable bowel syndrome	Functional bowel IBS	Nociplastic Pain
K580	Irritable bowel syndrome with diarrhea	Functional bowel IBS	Nociplastic Pain
K581	Irritable bowel syndrome with constipation	Functional bowel IBS	Nociplastic Pain
K582	Mixed irritable bowel syndrome	Functional bowel IBS	Nociplastic Pain
K588	Other irritable bowel syndrome	Functional bowel IBS	Nociplastic Pain

© 2024 Peterson MD et al. *JAMA Neurology.*

K589	Irritable bowel syndrome without diarrhea	Functional bowel IBS	Nociplastic Pain
M266	Temporomandibular joint disorders	TMJD	Nociplastic Pain
M544	Lumbago with sciatica	Back Pain Low Back	Nociplastic Pain
M5440	Lumbago with sciatica, unspecified side	Back Pain Low Back	Nociplastic Pain
M5441	Lumbago with sciatica, right side	Back Pain Low Back	Nociplastic Pain
M5442	Lumbago with sciatica, left side	Back Pain Low Back	Nociplastic Pain
M545	Low back pain	Back Pain Low Back	Nociplastic Pain
M5450	Low back pain, unspecified	Back Pain Low Back	Nociplastic Pain
M5451	Vertebrogenic low back pain	Back Pain Low Back	Nociplastic Pain
M5459	Other low back pain	Back Pain Low Back	Nociplastic Pain
M5481	Occipital neuralgia	Occipital neuralgia	Nociplastic Pain
M791	Myalgia	Myalgia and myositis, unspecified (Fibromyalgia)	Nociplastic Pain
M797	Fibromyalgia	Myalgia and myositis, unspecified (Fibromyalgia)	Nociplastic Pain
N3010	Interstitial cystitis (chronic) without hematuria	Interstitial cystitis	Nociplastic Pain
N3011	Interstitial cystitis (chronic) with hematuria	Interstitial cystitis	Nociplastic Pain
N80	Endometriosis	Endometriosis	Nociplastic Pain
N800	Endometriosis of uterus	Endometriosis	Nociplastic Pain
N8000	Endometriosis of the uterus, unspecified	Endometriosis	Nociplastic Pain
N8001	Superficial endometriosis of the uterus	Endometriosis	Nociplastic Pain
N8002	Deep endometriosis of the uterus	Endometriosis	Nociplastic Pain
N8003	Adenomyosis of the uterus	Endometriosis	Nociplastic Pain
N801	Endometriosis of ovary	Endometriosis	Nociplastic Pain
N8010	Endometriosis of ovary, unspecified depth	Endometriosis	Nociplastic Pain
N80101	Endometriosis of right ovary, unspecified depth	Endometriosis	Nociplastic Pain
N80102	Endometriosis of left ovary, unspecified depth	Endometriosis	Nociplastic Pain
N80103	Endometriosis of bilateral ovaries, unspecified depth	Endometriosis	Nociplastic Pain
N80109	Endometriosis of ovary, unspecified side, unspecified depth	Endometriosis	Nociplastic Pain
N8011	Superficial endometriosis of the ovary	Endometriosis	Nociplastic Pain
N80111	Superficial endometriosis of right ovary	Endometriosis	Nociplastic Pain
N80112	Superficial endometriosis of left ovary	Endometriosis	Nociplastic Pain
N80113	Superficial endometriosis of bilateral ovaries	Endometriosis	Nociplastic Pain
N80119	Superficial endometriosis of ovary, unspecified ovary	Endometriosis	Nociplastic Pain
N8012	Deep endometriosis of ovary	Endometriosis	Nociplastic Pain
N80121	Deep endometriosis of right ovary	Endometriosis	Nociplastic Pain
N80122	Deep endometriosis of left ovary	Endometriosis	Nociplastic Pain
N80123	Deep endometriosis of bilateral ovaries	Endometriosis	Nociplastic Pain
N80129	Deep endometriosis of ovary, unspecified ovary	Endometriosis	Nociplastic Pain

© 2024 Peterson MD et al. *JAMA Neurology.*

N802	Endometriosis of fallopian tube	Endometriosis	Nociplastic Pain
N8020	Endometriosis of fallopian tube, unspecified depth	Endometriosis	Nociplastic Pain
N80201	Endometriosis of right fallopian tube, unspecified depth	Endometriosis	Nociplastic Pain
N80202	Endometriosis of left fallopian tube, unspecified depth	Endometriosis	Nociplastic Pain
N80203	Endometriosis of bilateral fallops, unspecified depth	Endometriosis	Nociplastic Pain
N80209	Endometriosis of unspecified fallop, unspecified depth	Endometriosis	Nociplastic Pain
N8021	Superficial endometriosis of fallopian tube	Endometriosis	Nociplastic Pain
N80211	Superficial endometriosis of right fallopian tube	Endometriosis	Nociplastic Pain
N80212	Superficial endometriosis of left fallopian tube	Endometriosis	Nociplastic Pain
N80213	Superficial endometriosis of bilateral fallopian tubes	Endometriosis	Nociplastic Pain
N80219	Superficial endometriosis of unspecified fallopian tube	Endometriosis	Nociplastic Pain
N8022	Deep endometriosis of the fallopian tube	Endometriosis	Nociplastic Pain
N80221	Deep endometriosis of right fallopian tube	Endometriosis	Nociplastic Pain
N80222	Deep endometriosis of left fallopian tube	Endometriosis	Nociplastic Pain
N80223	Deep endometriosis of bilateral fallopian tubes	Endometriosis	Nociplastic Pain
N80229	Deep endometriosis of unspecified fallopian tube	Endometriosis	Nociplastic Pain
N803	Endometriosis of pelvic peritoneum	Endometriosis	Nociplastic Pain
N8030	Endometriosis of pelvic peritoneum, unspecified	Endometriosis	Nociplastic Pain
N8031	Endometriosis of the anterior cul-de-sac	Endometriosis	Nociplastic Pain
N80311	Superficial endometriosis of the anterior cul-de-sac	Endometriosis	Nociplastic Pain
N80312	Deep endometriosis of the anterior cul-de-sac	Endometriosis	Nociplastic Pain
N80319	Endometriosis of the anterior cul-de-sac, unspecified depth	Endometriosis	Nociplastic Pain
N8032	Endometriosis of the posterior cul-de-sac	Endometriosis	Nociplastic Pain
N80321	Superficial endometriosis of the posterior cul-de-sac	Endometriosis	Nociplastic Pain
N80322	Deep endometriosis of the posterior cul-de-sac	Endometriosis	Nociplastic Pain
N80329	Endometriosis of the posterior cul-de-sac, unspecified depth	Endometriosis	Nociplastic Pain
N8033	Superficial endometriosis of the pelvic sidewall	Endometriosis	Nociplastic Pain
N80331	Superficial endometriosis of the right pelvic sidewall	Endometriosis	Nociplastic Pain
N80332	Superficial endometriosis of the left pelvic sidewall	Endometriosis	Nociplastic Pain
N80333	Superficial endometriosis of bilateral pelvic sidewall	Endometriosis	Nociplastic Pain
N80339	Superfic endometriosis of pelvic sidewall, unspecified side	Endometriosis	Nociplastic Pain
N8034	Deep endometriosis of the pelvic sidewall	Endometriosis	Nociplastic Pain
N80341	Deep endometriosis of the right pelvic sidewall	Endometriosis	Nociplastic Pain
N80342	Deep endometriosis of the left pelvic sidewall	Endometriosis	Nociplastic Pain
N80343	Deep endometriosis of the bilateral pelvic sidewall	Endometriosis	Nociplastic Pain
N80349	Deep endometriosis of the pelvic sidewall, unspecified side	Endometriosis	Nociplastic Pain
N8035	Endometriosis of the pelvic sidewall, unspecified depth	Endometriosis	Nociplastic Pain

© 2024 Peterson MD et al. *JAMA Neurology.*

N80351	Endometriosis of the right pelvic sidewall, unsp depth	Endometriosis	Nociplastic Pain
N80352	Endometriosis of the left pelvic sidewall, unspecified depth	Endometriosis	Nociplastic Pain
N80353	Endometriosis of bilateral pelvic sidewall, unsp depth	Endometriosis	Nociplastic Pain
N80359	Endometriosis of pelvic sidewall, unsp side, unsp depth	Endometriosis	Nociplastic Pain
N8036	Superficial endometriosis of the pelvic brim	Endometriosis	Nociplastic Pain
N80361	Superficial endometriosis of the right pelvic brim	Endometriosis	Nociplastic Pain
N80362	Superficial endometriosis of the left pelvic brim	Endometriosis	Nociplastic Pain
N80363	Superficial endometriosis of bilateral pelvic brim	Endometriosis	Nociplastic Pain
N80369	Superfic endometriosis of the pelvic brim, unspecified side	Endometriosis	Nociplastic Pain
N8037	Deep endometriosis of the pelvic brim	Endometriosis	Nociplastic Pain
N80371	Deep endometriosis of the right pelvic brim	Endometriosis	Nociplastic Pain
N80372	Deep endometriosis of the left pelvic brim	Endometriosis	Nociplastic Pain
N80373	Deep endometriosis of bilateral pelvic brim	Endometriosis	Nociplastic Pain
N80379	Deep endometriosis of the pelvic brim, unspecified side	Endometriosis	Nociplastic Pain
N8038	Endometriosis of the pelvic brim, unspecified depth	Endometriosis	Nociplastic Pain
N80381	Endometriosis of the right pelvic brim, unspecified depth	Endometriosis	Nociplastic Pain
N80382	Endometriosis of the left pelvic brim, unspecified depth	Endometriosis	Nociplastic Pain
N80383	Endometriosis of bilateral pelvic brim, unspecified depth	Endometriosis	Nociplastic Pain
N80389	Endometriosis of the pelvic brim, unsp side, unsp depth	Endometriosis	Nociplastic Pain
N8039	Endometriosis of other pelvic peritoneum	Endometriosis	Nociplastic Pain
N80391	Superfic endometriosis of the pelvic peritoneum, oth sites	Endometriosis	Nociplastic Pain
N80392	Deep endometriosis of the pelvic peritoneum, oth sites	Endometriosis	Nociplastic Pain
N80399	Endometriosis of pelvic peritoneum, oth sites, unsp depth	Endometriosis	Nociplastic Pain
N803A	Superficial endometriosis of the uterosacral ligament(s)	Endometriosis	Nociplastic Pain
N803A1	Superficial endometriosis of the right uterosacral ligament	Endometriosis	Nociplastic Pain
N803A2	Superficial endometriosis of the left uterosacral ligament	Endometriosis	Nociplastic Pain
N803A3	Superfic endometriosis of the bi uterosacral ligament(s)	Endometriosis	Nociplastic Pain
N803A9	Superfic endometriosis of uterosacral ligmt(s), unsp side	Endometriosis	Nociplastic Pain
N803B	Deep endometriosis of the uterosacral ligament(s)	Endometriosis	Nociplastic Pain
N803B1	Deep endometriosis of the right uterosacral ligament	Endometriosis	Nociplastic Pain
N803B2	Deep endometriosis of the left uterosacral ligament	Endometriosis	Nociplastic Pain
N803B3	Deep endometriosis of bilateral uterosacral ligament(s)	Endometriosis	Nociplastic Pain
N803B9	Deep endometriosis of the uterosacral ligament(s), unsp side	Endometriosis	Nociplastic Pain
N803C	Endometriosis of the uterosacral ligament(s), unsp depth	Endometriosis	Nociplastic Pain
N803C1	Endometriosis of the right uterosacral ligament, unsp depth	Endometriosis	Nociplastic Pain
N803C2	Endometriosis of the left uterosacral ligament, unsp depth	Endometriosis	Nociplastic Pain
N803C3	Endometriosis of bi uterosacral ligament(s), unsp depth	Endometriosis	Nociplastic Pain

© 2024 Peterson MD et al. *JAMA Neurology.*

N803C9	Endometriosis of uterosacral ligmt(s), unsp side, unsp depth	Endometriosis	Nociplastic Pain
N804	Endometriosis of rectovaginal septum and vagina	Endometriosis	Nociplastic Pain
N8040	Endometriosis of rectovaginal septum, unsp invl of vagina	Endometriosis	Nociplastic Pain
N8041	Endometriosis of rectovaginal septum without invl of vagina	Endometriosis	Nociplastic Pain
N8042	Endometriosis of rectovaginal septum with invl of vagina	Endometriosis	Nociplastic Pain
N805	Endometriosis of intestine	Endometriosis	Nociplastic Pain
N8050	Endometriosis of intestine, unspecified	Endometriosis	Nociplastic Pain
N8051	Endometriosis of the rectum	Endometriosis	Nociplastic Pain
N80511	Superficial endometriosis of the rectum	Endometriosis	Nociplastic Pain
N80512	Deep endometriosis of the rectum	Endometriosis	Nociplastic Pain
N80519	Endometriosis of the rectum, unspecified depth	Endometriosis	Nociplastic Pain
N8052	Endometriosis of the sigmoid colon	Endometriosis	Nociplastic Pain
N80521	Superficial endometriosis of the sigmoid colon	Endometriosis	Nociplastic Pain
N80522	Deep endometriosis of the sigmoid colon	Endometriosis	Nociplastic Pain
N80529	Endometriosis of the sigmoid colon, unspecified depth	Endometriosis	Nociplastic Pain
N8053	Endometriosis of the cecum	Endometriosis	Nociplastic Pain
N80531	Superficial endometriosis of the cecum	Endometriosis	Nociplastic Pain
N80532	Deep endometriosis of the cecum	Endometriosis	Nociplastic Pain
N80539	Endometriosis of the cecum, unspecified depth	Endometriosis	Nociplastic Pain
N8054	Endometriosis of the appendix	Endometriosis	Nociplastic Pain
N80541	Superficial endometriosis of the appendix	Endometriosis	Nociplastic Pain
N80542	Deep endometriosis of the appendix	Endometriosis	Nociplastic Pain
N80549	Endometriosis of the appendix, unspecified depth	Endometriosis	Nociplastic Pain
N8055	Endometriosis of other parts of the colon	Endometriosis	Nociplastic Pain
N80551	Superficial endometriosis of other parts of the colon	Endometriosis	Nociplastic Pain
N80552	Deep endometriosis of other parts of the colon	Endometriosis	Nociplastic Pain
N80559	Endometriosis of other parts of the colon, unspecified depth	Endometriosis	Nociplastic Pain
N8056	Endometriosis of the small intestine	Endometriosis	Nociplastic Pain
N80561	Superficial endometriosis of the small intestine	Endometriosis	Nociplastic Pain
N80562	Deep endometriosis of the small intestine	Endometriosis	Nociplastic Pain
N80569	Endometriosis of the small intestine, unspecified depth	Endometriosis	Nociplastic Pain
N806	Endometriosis in cutaneous scar	Endometriosis	Nociplastic Pain
N808	Other endometriosis	Endometriosis	Nociplastic Pain
N809	Endometriosis, unspecified	Endometriosis	Nociplastic Pain
N80A	Endometriosis of bladder and ureters	Endometriosis	Nociplastic Pain
N80A0	Endometriosis of bladder, unspecified depth	Endometriosis	Nociplastic Pain
N80A1	Superficial endometriosis of bladder	Endometriosis	Nociplastic Pain

© 2024 Peterson MD et al. *JAMA Neurology.*

N80A2	Deep endometriosis of bladder	Endometriosis	Nociplastic Pain
N80A4	Superficial endometriosis of ureter	Endometriosis	Nociplastic Pain
N80A41	Superficial endometriosis of right ureter	Endometriosis	Nociplastic Pain
N80A42	Superficial endometriosis of left ureter	Endometriosis	Nociplastic Pain
N80A43	Superficial endometriosis of bilateral ureters	Endometriosis	Nociplastic Pain
N80A49	Superficial endometriosis of unspecified ureter	Endometriosis	Nociplastic Pain
N80A5	Deep endometriosis of ureter	Endometriosis	Nociplastic Pain
N80A51	Deep endometriosis of right ureter	Endometriosis	Nociplastic Pain
N80A52	Deep endometriosis of left ureter	Endometriosis	Nociplastic Pain
N80A53	Deep endometriosis of bilateral ureters	Endometriosis	Nociplastic Pain
N80A59	Deep endometriosis of unspecified ureter	Endometriosis	Nociplastic Pain
N80A6	Endometriosis of ureter, unspecified depth	Endometriosis	Nociplastic Pain
N80A61	Endometriosis of right ureter, unspecified depth	Endometriosis	Nociplastic Pain
N80A62	Endometriosis of left ureter, unspecified depth	Endometriosis	Nociplastic Pain
N80A63	Endometriosis of bilateral ureters, unspecified depth	Endometriosis	Nociplastic Pain
N80A69	Endometriosis of unspecified ureter, unspecified depth	Endometriosis	Nociplastic Pain
N80B	Endometriosis of cardiothoracic space	Endometriosis	Nociplastic Pain
N80B1	Endometriosis of pleura	Endometriosis	Nociplastic Pain
N80B2	Endometriosis of lung	Endometriosis	Nociplastic Pain
N80B3	Endometriosis of diaphragm	Endometriosis	Nociplastic Pain
N80B31	Superficial endometriosis of diaphragm	Endometriosis	Nociplastic Pain
N80B32	Deep endometriosis of diaphragm	Endometriosis	Nociplastic Pain
N80B39	Endometriosis of diaphragm, unspecified depth	Endometriosis	Nociplastic Pain
N80B4	Endometriosis of the pericardial space	Endometriosis	Nociplastic Pain
N80B5	Endometriosis of the mediastinal space	Endometriosis	Nociplastic Pain
N80B6	Endometriosis of cardiothoracic space	Endometriosis	Nociplastic Pain
N80C	Endometriosis of the abdomen	Endometriosis	Nociplastic Pain
N80C0	Endometriosis of the abdomen, unspecified	Endometriosis	Nociplastic Pain
N80C1	Endometriosis of the anterior abdominal wall	Endometriosis	Nociplastic Pain
N80C10	Endometriosis of the anterior abd wall, subcutaneous tissue	Endometriosis	Nociplastic Pain
N80C11	Endometriosis of ant abd wall, fascia and muscular layers	Endometriosis	Nociplastic Pain
N80C19	Endometriosis of the anterior abd wall, unspecified depth	Endometriosis	Nociplastic Pain
N80C2	Endometriosis of the umbilicus	Endometriosis	Nociplastic Pain
N80C3	Endometriosis of the inguinal canal	Endometriosis	Nociplastic Pain
N80C4	Endometriosis of extra-pelvic abdominal peritoneum	Endometriosis	Nociplastic Pain
N80C9	Endometriosis of other site of abdomen	Endometriosis	Nociplastic Pain
N80D	Endometriosis of the pelvic nerves	Endometriosis	Nociplastic Pain

© 2024 Peterson MD et al. *JAMA Neurology.*

N80D0	Endometriosis of the pelvic nerves, unspecified	Endometriosis	Nociplastic Pain
N80D1	Endometriosis of the sacral splanchnic nerves	Endometriosis	Nociplastic Pain
N80D2	Endometriosis of the sacral nerve roots	Endometriosis	Nociplastic Pain
N80D3	Endometriosis of the obturator nerve	Endometriosis	Nociplastic Pain
N80D4	Endometriosis of the sciatic nerve	Endometriosis	Nociplastic Pain
N80D5	Endometriosis of the pudendal nerve	Endometriosis	Nociplastic Pain
N80D6	Endometriosis of the femoral nerve	Endometriosis	Nociplastic Pain
N80D9	Endometriosis of other pelvic nerve	Endometriosis	Nociplastic Pain
N9481	Vulvodynia	Vulvodynia	Nociplastic Pain
N94810	Vulvar vestibulitis	Vulvodynia	Nociplastic Pain
N94818	Other vulvodynia	Vulvodynia	Nociplastic Pain
N94819	Vulvodynia, unspecified	Vulvodynia	Nociplastic Pain
R0782	Intercostal pain	Noncardiac or musculoskeletal chest pain	Nociplastic Pain
R0789	Other chest pain	Noncardiac or musculoskeletal chest pain	Nociplastic Pain
R51	Headache	Headache	Nociplastic Pain
R5382	Chronic fatigue, unspecified	Chronic Fatigue	Nociplastic Pain

© 2024 Peterson MD et al. *JAMA Neurology.*
